# Regulatory mechanisms link phenotypic plasticity to evolvability

**DOI:** 10.1038/srep24524

**Published:** 2016-04-18

**Authors:** Jordi van Gestel, Franz J. Weissing

**Affiliations:** 1 Groningen Institute for Evolutionary Life Sciences, University of Groningen, P.O. Box 11103, Groningen 9700 CC, The Netherlands

## Abstract

Organisms have a remarkable capacity to respond to environmental change. They can either respond directly, by means of phenotypic plasticity, or they can slowly adapt through evolution. Yet, how phenotypic plasticity links to evolutionary adaptability is largely unknown. Current studies of plasticity tend to adopt a phenomenological reaction norm (RN) approach, which neglects the mechanisms underlying plasticity. Focusing on a concrete question – the optimal timing of bacterial sporulation – we here also consider a mechanistic approach, the evolution of a gene regulatory network (GRN) underlying plasticity. Using individual-based simulations, we compare the RN and GRN approach and find a number of striking differences. Most importantly, the GRN model results in a much higher diversity of responsive strategies than the RN model. We show that each of the evolved strategies is pre-adapted to a unique set of unseen environmental conditions. The regulatory mechanisms that control plasticity therefore critically link phenotypic plasticity to the adaptive potential of biological populations.

Life is adaptable. Organisms can slowly adapt by means of evolution or they can directly respond to changing environmental conditions^1^. The capacity of an organism to express different phenotypes in response to the environment is referred to as phenotypic plasticity^2^. All organisms express plasticity^3^. Yet, despite its prevalence, it often remains difficult to understand how plasticity evolved. First, one needs to have a good understanding of the possible response strategies. Which information can an individual obtain from the environment? What are the available response options? Second, one needs a firm understanding of how changes in phenotypic responsivity are related to changes at the genetic level. How do mutations affect the plastic responsiveness of an individual? A question like this may be difficult to answer, since the genotype-to-phenotype mapping (i.e., the relation between an organism’s genotype and phenotype) is often very complex and largely unknown^4^,^5^. Third, individuals not only respond to the environment, they also shape their environment^6^. For example, many bacteria influence their environment by secreting products like antimicrobials, communicative signals and waste products^7^,^8^,^9^,^10^. Moreover, in social interactions the phenotype of an individual typically influences the social environment to which it is exposed^11^,^12^. The dual role of the environment as both the cause and consequence of an individual’s phenotype makes it difficult to study the evolution of plasticity.

In this study, we examine the evolution of plasticity using a mechanistic model, in which we explicitly account for the genotype-to-phenotype mapping and the interaction between individuals and their environment, without making the model intractable. Our goal is to understand how a mechanistic implementation of plasticity affects the outcome of evolution. To this end, we focus on a specific system: bacterial sporulation. The regulatory underpinnings of bacterial sporulation have intensely been studied^13^,^14^,^15^. Sporulation forms the ultimate survival strategy that is triggered when cells face harsh environmental conditions^16^. Sporulation is costly, involving both time and energy, and results in the production of a metabolically inactive spore^16^. Spores can survive long periods of environmental stress, such as starvation, desiccation and radiation. The sporulation process is controlled by a gene regulatory network that integrates multiple cues, such as starvation cues, communicative signals and physiological cues^14^. Together these cues determine when and where a cell sporulates. This is particularly apparent in bacterial colonies, where cells – in response to their environment – trigger sporulation in specific regions of the colony^17^,^18^.

Using individual-based simulations, we study the evolution of sporulation. We compare two alternative implementations of the genotype-to-phenotype mapping: (1) a classical reaction norm approach and (2) a simple mechanistic implementation of a gene regulatory network. Reaction norms give a phenomenological description of plasticity, by directly considering the response of an organism to its local environment^19^. Reaction norms are a valuable tool to compare the plastic responses of different genotypes, but they often ignore the regulatory mechanisms that underlie plasticity^19^,^20^,^21^. The question therefore arises whether, and to what extent, an explicit consideration of these mechanisms would affect predictions concerning the evolution of plasticity. To address this question, we compare a reaction norm approach to a more mechanistic gene regulatory network approach. In both model implementations, cells grow in colonies, where they interact with the environment by consuming nutrients and secreting products. Over time, colonies go through consecutive rounds of colony growth and dispersal. Spores are more likely to survive dispersal and thereby to found new colonies, yet spores cannot divide and therefore hamper colony growth. The fitness of a sporulation strategy is determined by the timing of sporulation: when a cell sporulates too early it forgoes cell division and when it sporulates too late it increases the chance to die when conditions are getting harsh. Cells can evolve the timing of sporulation by changing their responsiveness to the environment.

For each model implementation, we investigate if and how the cell’s responsiveness evolves by running 500 replicate simulations. In addition, we perform a detailed evaluation of the evolved responsive strategies, by examining their differences and by exposing each strategy to a set of novel environmental conditions.

## Structure of the model

**Figure 1:**
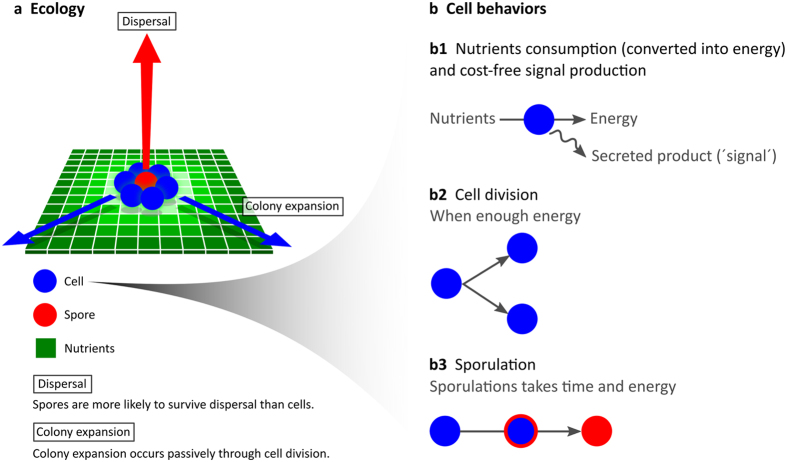
Colony growth and cell behaviours. (**a**) Individuals divide and differentiate on a two dimensional grid (blue, cells; red, spores). Cells sense the local environmental conditions, determined by the quadrant on which a cell is placed. Nutrients (green) and signal (not shown) diffuse in space. Colony growth occurs for a fixed number of time steps, after which all individuals can disperse. Spores are ten times more likely to survive dispersal than cells. (**b**) Cells can express three behaviours: (b1) cells can consume nutrients, which are converted to energy, and secrete molecular products in the local environment (called ‘signal’); (b2) cells with sufficient energy have a fixed probability to divide; (b3) cells can sporulate, which requires time and energy. Only cells that finish the sporulation process are more likely to survive dispersal than cells.

We model a population of cells that grow inside a colony. The colony is placed on a two-dimensional surface that contains nutrients (Fig. 1a). Cells consume these nutrients, convert them to energy, and subsequently use this energy to divide. Hence, while colony growth progresses, nutrients get depleted. The colony grows for a fixed time period. At the end, cells can disperse and colonize a new nutrient surface, which forms the beginning of a new cycle of colony growth. We assume that cells only interact with their local environment. Thus, cells only consume nutrients from the grid element on which they are placed (see Material and Methods). These consumed nutrients are partly replenished by diffusion. We also assume that cells secrete a product into their local environment, which they can sense as well (Fig. 1b). We do not specify the nature of this product, so it can be anything from a waste product to a quorum-sensing signal. Take for example the soil bacterium *Bacillus subtilis*, it secretes numerous products during colony growth, these range from antimicrobials (e.g. surfactin)^22^ to pentapeptides (e.g. PhrC)^23^,^14^,^24^. Even though these molecules might not primarily function as communicative signals, they are secreted and sensed by the cells and can affect the timing of sporulation^18^,^25^. Henceforth we will refer to the secreted product in our model as ‘signal’. During colony growth, cells can decide to allocate their energy to cell division or sporulation (Fig. 1b). Sporulation requires time and energy and at end of sporulation a cell transforms into a spore. Spores are metabolically inactive and cannot divide, yet spores are ten times more likely to survive dispersal than cells. Thus, there is a trade-off between cell division and survival. The fitness of a genotype is largely determined by the number of spores it produces by the end of colony growth, which – as explained above – depends on the timing of sporulation. In fact, spore production forms an accurate proxy of fitness (Supplementary Fig. S1), even though cell production also contributes weakly to the reproductive success of a colony.

**Figure 2:**
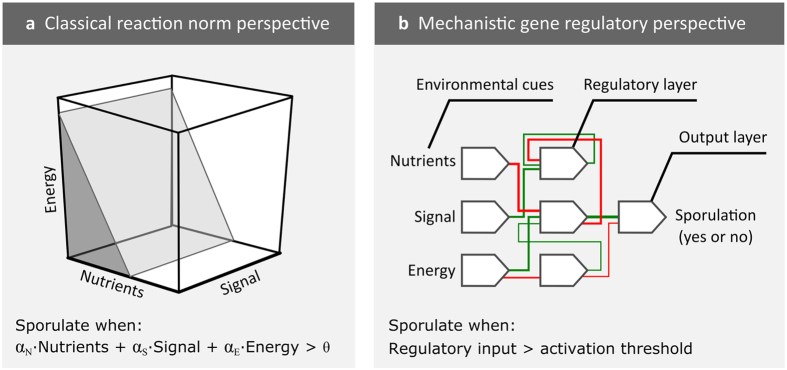
The genotype-to-phenotype mapping. Two different implementations of the genotype-to-phenotype mapping are considered: (**a**) a reaction norm (RN) and (**b**) a gene regulatory network (GRN). (**a**) In the RN model the environmental cues directly determine if a cell sporulates or not, as shown by the inequality below the three-dimensional reaction norm. (**b**) In the GRN model the environmental cues affect gene expression. The GRN consists of three layers: input layer, regulatory layer and output layer. The input layer processes the three environmental cues. These cues subsequently affect the gene expression in the regulatory layer, which affect the expression of the gene in the output layer. Only when the ‘output’ gene is expressed a cell sporulates. A gene is expressed when the regulatory input exceeds the gene’s activation threshold.

In both model implementations – the reaction norm (RN) model and the gene regulatory network (GRN) model – cells can trigger sporulation in response to three environmental cues (Fig. 2): nutrient concentration (N), signal concentration (S) and energy level (E). Both the nutrient and signal concentration are sensed from the local environment of a cell, while the energy level is associated with the physiological state of a cell. In the RN model, these cues directly determine if a cell sporulates or not. Each cue is multiplied by a certain weighting factor and a cell sporulates when the sum of regulatory input exceeds the activation threshold (Fig. 2a, see Material and Methods). The weighting factors (α) and activation threshold (θ) are heritable and subject to evolution. Every time a cell divides, these parameters are transmitted to the offspring, subject to rare mutations of small effect size. In the GRN model, sporulation is not triggered directly, but determined by the output of a gene regulatory network. The network consists of three layers: input layer, regulatory layer and output layer (Fig. 2b, see Material and Methods). The cues are processed by the input layer of the GRN and can affect gene expression. We assume that gene expression is Boolean, so genes are either expressed or not. The expression of a gene is determined by the regulatory input it receives and its activation threshold. The regulatory input depends on the connection weights in the GRN. When the sum of regulatory input exceeds the activation threshold a gene is expressed. When the gene in the output layer is expressed a cell sporulates. The connection weights and activation thresholds are heritable and subject to evolution. Every time a cell divides, they have a small probability to mutate.

In both models, cells evolve their responsiveness to the environment, either by changes in the RN or GRN. This responsiveness in turn determines the timing of sporulation. All simulations were initiated by a population of cells that could not sporulate – i.e. all evolvable variables were set to zero. The population could subsequently evolve for 400 consecutive colony growth cycles, to which we refer as ‘generations’.

## Results

### The evolution of plasticity in the reaction norm model

**Figure 3:**
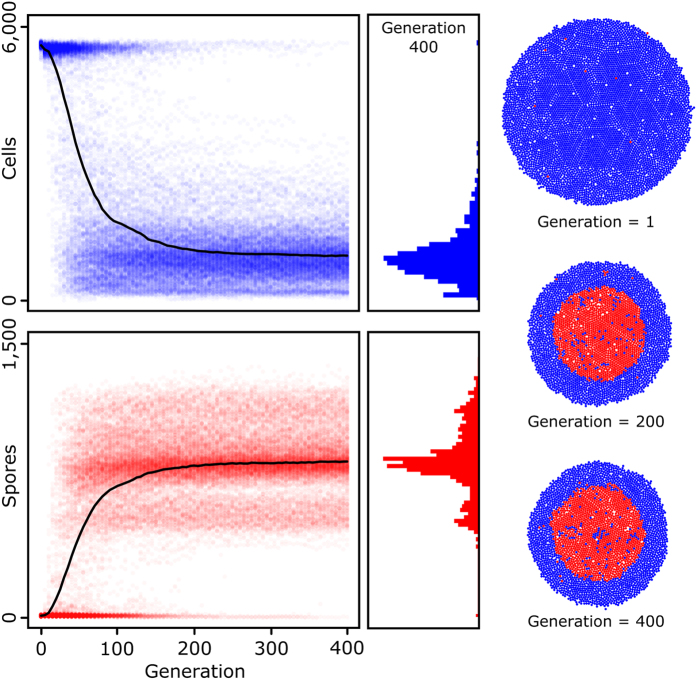
Evolution of sporulation in the RN model. (Left) Number of cells (blue) and spores (red) in 500 replicate simulations over the course of 400 generations. At each generation, cell and spore counts are collected at the end of colony growth. The black lines show the average number of cells and spores. (middle) Distributions of the number of cells and spores over the 500 replicate simulations at the end of evolution. (right) Colonies of the most productive genotype at generation 1, 200 and 400.

We first examine the evolution of plasticity in the RN model. Figure 3 shows the number of cells and spores in the 500 replicate simulations over the course of 400 generations. Since at the onset of evolution cells are unable to sporulate, the colonies in the first generation do not produce spores, with the exception of some mutants. Over evolutionary time, sporulation evolves and the number of spores that are present at the end of colony growth increases (Fig. 3). At the same time the colony size decreases, because energy that is allocated to sporulation cannot be used for cell division. Despite the smaller colonies, the evolved genotypes have a higher fitness than their non-sporulating ancestors, because spores are more likely to survive dispersal than cells. The colony at generation 200 is characterized by two radial zones: the center and the edge. Spores mostly occur in the colony center, where nutrients are depleted first, while dividing cells occur at the colony edge. From generation 200 onwards, the colonies of most replicate simulations produce a constant number of spores until the end of evolution. Interestingly, when examining the distribution of spore production among the 500 replicate simulations, one can discriminate three phenotypic groups: colonies that produce a low (~600 spores), intermediate (~800) and high (~1,200) number of spores. Since the colonies belong to separate replicate simulations, we hypothesized that three phenotypic groups belong to separate evolutionary trajectories, each trajectory leading to another level of spore production.

#### Diversity

**Figure 4:**
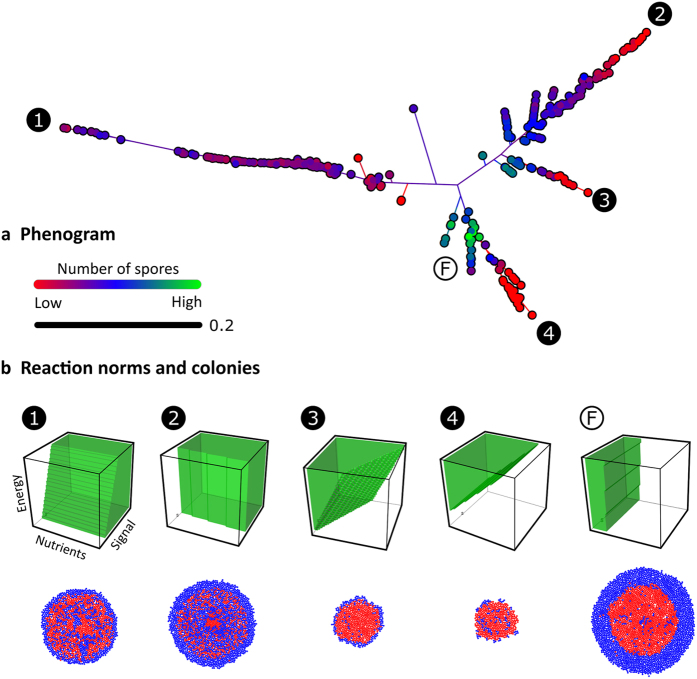
The diversity in evolved reaction norms in the RN model. (**a**) Phenogram based on the distance between reaction norms of the most frequent genotypes in the 500 replicate simulations at the end of evolution. The distance between two reaction norms is given by the fraction of conditions at which they prescribe a different response. Colours indicate spore production of genotypes: low (red), intermediate (blue) and high (green). (**b**) The reaction norms and corresponding colonies that are associated with the tips of the branches in the phenogram (numbered 1–4) and the most productive genotype (F).

In order to characterize the diversity of reaction norms at generation 400, we compared the reaction norms of all 500 replicate simulations in a pairwise fashion. For each pairwise combination, we determined the fraction of conditions (N, S and E) for which cells with the associated reaction norms would take different decisions: one cell would sporulate, while the other cell would not. Using a hierarchical cluster analysis (see Material and Methods), the pairwise differences were converted into a phenogram, which illustrates the diversity of reaction norms present at the end of evolution (Fig. 4a). Each dot is associated with a single evolved genotype and its associated reaction norm (corresponding to one simulation) and the branch lengths correspond to the differences between the reaction norms. The colors of the dots correspond to the productivity of the given genotypes, which is defined by the number of spores present at the end of colony growth (an accurate proxy for a genotype’s fitness, see Supplementary Fig. S1).

The phenogram consists of four main branches (Fig. 4a; numbered 1–4). The most productive genotypes all occur in the same branch (see label F in branch 4). The reaction norms and colonies that correspond to the tip of the branches are shown below the phenogram (Fig. 4b; label 1–4). Figure 4b also shows the reaction norm and colony of the most productive genotype (label F; see Supplementary Figs S2 and S4 for the twenty most productive reaction norms and the associated genotypes). Interestingly, only the reaction norm of branch 3 is sensitive to all cues, the rest only responds to two of the three cues. The reaction norms of branch 1, 2 and 4 are insensitive to respectively the nutrient concentration, energy level and signal concentration. The reaction norm of the most productive genotype is insensitive to the signal concentration and mostly affected by the nutrient concentration: at low nutrient concentrations sporulation is triggered and spores were confined to the colony center. The genotypes that are associated with the other four reaction norms produce a low number of spores, as is apparent from their colonies, which are either small or show low sporulation efficiencies. The branched structure of the phenogram explains why evolution resulted in three more-or-less discrete phenotypic groups (Fig. 3). Once a genotype belongs to a certain branch, it is expected to climb the local fitness gradient that is present in this branch. This genotype is unlikely to switch between branches, because this requires the accumulation of multiple mutations, many of which may be deleterious. Thus, genotypes follow alternative evolutionary trajectories that lead to different levels of spore production.

### The evolution of plasticity in the gene regulatory network model

In the GRN model sporulation evolves quickly as well and, like in the RN model, results in colonies with spores in the center and dividing cells at the edge (Supplementary Fig. S6). However, in contrast to the RN model (Fig. 3), there are no clear groups differing in spore production at the end of evolution (Supplementary Fig. S6): most of the evolved colonies produce between 900 and 1,200 spores. Moreover, on average, the evolved genotypes in the GRN model produce more spores (median = 1,060, interquartile range = [968; 1,173]) than those from the RN model (median = 849, interquartile range = [788;936]). This is also the case when only comparing the twenty most productive genotypes, which produce approximately 250 spores more in the GRN model (mean and standard deviation = 1,491 ± 66) than in the RN model (1,229 ± 43).

#### Information processing and cellular memory

**Figure 5:**
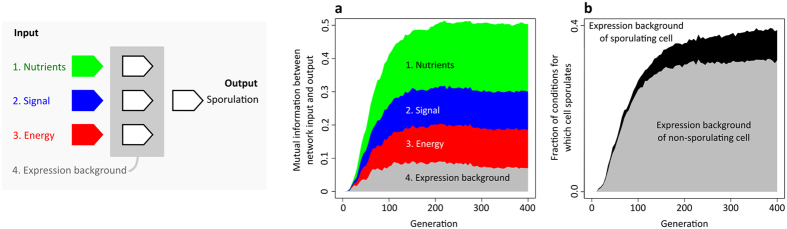
Mutual information between network input and output in the GRN model. (**a**) Average mutual information between network input – (1) nutrients (green area), (2) signal (blue area), (3) energy (red area), (4) expression background (grey area) – and network output (i.e. sporulation). The gene regulatory network on the left shows the relation between network input and output. The mutual information values were calculated for the most frequent genotype in each replicate simulation and averaged over all 500 replicate simulations. (**b**) Fraction of environmental conditions for which cells sporulate when having the expression background of a sporulating or a non-sporulating cell. The black area indicates the difference in the fraction of sporulation conditions between the two expression backgrounds.

In the GRN model, the environmental cues do not directly determine the phenotype of a cell, but are first processed by the GRN. We use the mutual information metric (see Material and Methods) to quantify the extent to which the output of a network depends on a given input: when the mutual information that is associated with a network input is high, the output of the network is to a large extent determined by this input. Figure 5a shows for each network input – N, S and E – how, on average, the mutual information value increase over evolutionary time, especially within the first 200 generations (see Supplementary Text S1 for the mutual information values in the RN model). Thus, on average, the output of an evolved GRN depends on all network inputs.

In addition to the three cues, the output of a GRN can also be affected by the expression state of genes in the regulatory layer. Gene expression in the regulatory layer is inherited from one time step to the next and, depending on the regulatory interactions inside the network, can affect the output of the network. For example, suppose that one of the genes in the regulatory layer strongly stimulates its own expression. Once this gene is expressed, it will continue to be expressed, even if the initial conditions that triggered its expression are absent. In fact, feedback interactions can lead to the inheritance of gene expression between generations, a phenomenon known as epigenetic inheritance through self-sustaining loops^26^. We will refer to the expression state of genes in the regulatory layer as the ‘expression background’. It is important to realize that the expression background is a product of past environment conditions and the regulatory interactions inside the network, which can ´memorize´ these conditions (e.g. possible feedback interactions in the regulatory layer of the network). Figure 5a shows that the mutual information between the expression background and the network´s output increased over time (grey area). Thus, the evolved GRNs depend on the nutrient concentration, signal concentration, energy level and expression background.

Even though the mutual information shows that the expression background affects a cell´s decision to sporulate or not, it does not show how this decision is affected. We therefore determined how the expression background affects the fraction of conditions – possible combinations of N, S and E – for which a cell sporulates. Each evolved GRN was exposed to a large set of environmental conditions using different expression backgrounds: the expression background of a sporulating and a non-sporulating cell. The typical expression background of a sporulating and non-sporulating cell was determined separately for each evolved GRN (see Material and Methods). When cells have the expression background of a sporulating cell, they sporulate for a larger fraction of conditions than when they have the expression background of a non-sporulating cell (Fig. 5b). Thus, cells that sporulate change their sensitivity to the environment such that they are more likely to continue sporulation in the presence of small environmental perturbations. In this way, no time or energy is wasted on failed sporulation attempts.

#### Diversity

**Figure 6:**
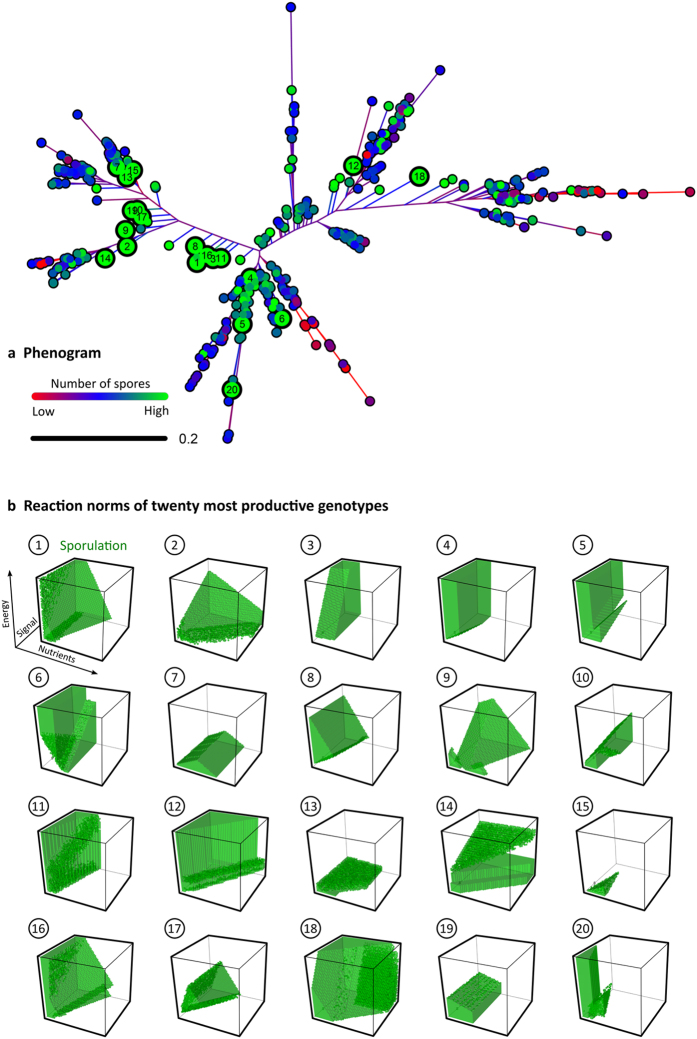
Diversity in reaction norms in the GRN model. (**a**) Phenogram based on the distance between reaction norms of the most frequent genotypes in the 500 replicate simulations at the end of evolution. The distance between two reaction norms is given by the fraction of conditions at which they prescribe a different response. Colours indicate spore production of genotypes: low (red), intermediate (blue) and high (green). The twenty most productive genotypes are shown by larger dots. (**b**) The reaction norms associated with the twenty most productive genotypes ranked from the genotype that produces the largest number of spores (1) to the one that produces the smallest number of spores (20). For the twenty most productive reaction norms in the RN model see Supplementary Fig. S2.

In the previous section, the *average* properties of the evolved GRNs were examined. In order to characterize the *individual* properties, we determined the reaction norms associated with the evolved GRNs by exposing each GRN to all possible combinations of N, S and E (see Material and Methods). Figure 6 shows the phenogram based on these reaction norms as well as the reaction norms of the twenty most productive GRNs at the end of evolution (see Supplementary Fig. S10 for the underlying genotypes). In contrast to the RN model (Fig. 4), the phenogram of the GRN model shows a much higher diversity in the evolved reaction norms. Even the twenty most productive genotypes, which produce approximately the same number of spores (Supplementary Fig. S7), differ considerably in their reaction norms: some genotypes only sporulate for a small set of conditions (e.g. genotype 15), while others sporulate for the majority of conditions (e.g. genotype 18). Moreover, while some genotypes are insensitive to a certain environmental cue (e.g. genotype 7 is insensitive to the amount of signal), others almost entirely base their decision to differentiate on that same cue (e.g. genotype 12 strongly depends on the amount of signal for its decision to sporulate). The diversity in reaction norms is a product of the diverse regulatory interactions that are present in the evolved genotypes (see Supplementary Fig. S10).

Notice that the reaction norm of a GRN can only be determined for a given expression background. Supplementary Fig. S8 shows that the reaction norms of the evolved GRNs for different expression background. While the reaction norm of some genotypes is insensitive to the expression background (e.g. genotype 17), other genotypes are strongly affected (e.g. genotype 15). The diversity in GRNs is also apparent by comparing the mutual information values of individual GRNs (Supplementary Text S1) or when comparing the twenty most productive genotypes directly (Supplementary Text S2). Thus, in conclusion, evolution in the GRN model results in a remarkably diverse set of responsive strategies. Even the twenty most productive genotypes are scattered throughout nearly the entire phenogram. This strongly contrasts the twenty fittest genotypes in the RN model, which cluster together in the phenogram and look nearly identical (Supplementary Fig. S2 and Supplementary Text S1 and S2).

#### Informational redundancy and irrelevant parts of the reaction norms

How can it be that GRNs produce similar colonies, with nearly the same number of spores, while having such different reaction norms? First, the GRNs might respond to different cues, but still sporulate at approximately the same time, thereby forming similar colonies. In other words, the same response pattern might be based on different sources of information. Second, reaction norms might differ for conditions that are only rarely encountered during colony growth. The diversity in reaction norms might therefore result from the accumulation of mutations that are effectively neutral, since they only affect irrelevant parts of the reaction norm. The two possible causes of diversity are not mutually exclusive; GRNs might respond to different cues and differ in their responsiveness to conditions that they never encounter in the colony.

**Figure 7:**
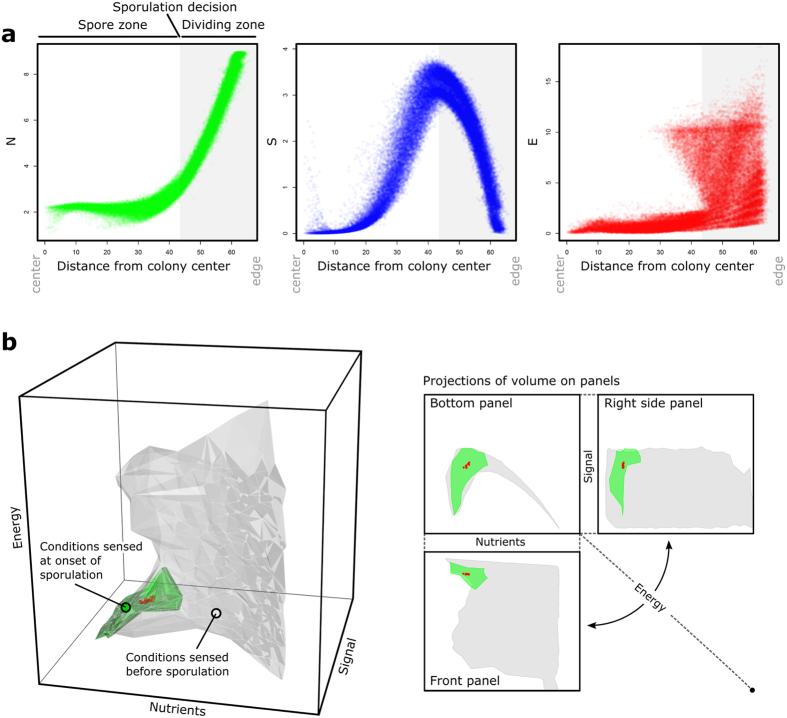
Environmental cues and informational redundancy in the GRN model. The environmental conditions in ten replicate colonies of the most productive genotype. (**a**) Environmental cues sensed by cells as a function of their distance to the colony center: N = nutrients (green), S = signal (blue), E = energy (red). Each dot corresponds to the conditions sensed by a single cell. The colonies are divided in two zones: spore zone and dividing zone. (**b**) Subset of conditions that cells sense before (grey volume) and at the onset of sporulation (green volume). The red dots correspond to the *average* conditions that cells experience at onset of sporulation in the ten replicate colonies.

As illustrated in Supplementary Figs S6 and S7, evolved colonies are characterized by two radial zones: the center (the ‘spore zone’) and the edge (the ‘dividing zone’). Evidently, cells in the dividing zone experience other conditions than cells in the spore zone (Fig. 7a). Nutrients are abundant at the colony edge and gradually decrease in abundance towards the center. Signal is produced by dividing cells at the colony edge, but not by the spores. Consequently, there is a peak in the signal concentration that reaches its maximum in the transition between both zones. Cells in the dividing zone show a wide variety of energy levels, while the spores in the colony center are depleted of energy. Given their strong gradients, both the nutrient and the signal concentration can be used by cells to determine their position in the colony. In addition, there is a strong negative correlation between the nutrient and signal concentration in the dividing zone. Thus, the nutrient and signal concentration give redundant information about each other and the location of cells inside the colony. As a consequence, evolved GRNs can respond to different cues, but still express the same behavior: a GRN that is sensitive to the nutrient concentration can extract essentially the same environmental information as a GRN that is sensitive to the signal concentration.

The environmental gradients also make it evident that cells only experience a small subset of conditions. Figure 7b shows the conditions cells experience before (grey volume) and at (green volume) the onset of sporulation in the space of environmental conditions that is also used for the reaction norms in Fig. 6. Cells clearly experience only a limited subset of conditions within the colony. Conditionally neutral mutations that alter the reaction norms for conditions that cells rarely or never encounter can increase the diversity of responsive strategies, without having an immediate effect on the phenotypes that are expressed. This, together with the informational redundancy between environmental cues, can explain the high diversity of responsive strategies that evolved among the twenty most productive genotypes in the GRN model (Fig. 6).

### Pre-adaptation to novel environments

Even though the diverse responsive strategies behave nearly identical in the environmental conditions that they encountered during evolution, they might express differences in novel environmental conditions, which can expose previously hidden parts of the reaction norms. To examine how the evolved genotypes respond to unseen environmental conditions, we exposed the twenty most productive genotypes from both the RN and GRN model to 250 randomly generated novel environments.

**Figure 8:**
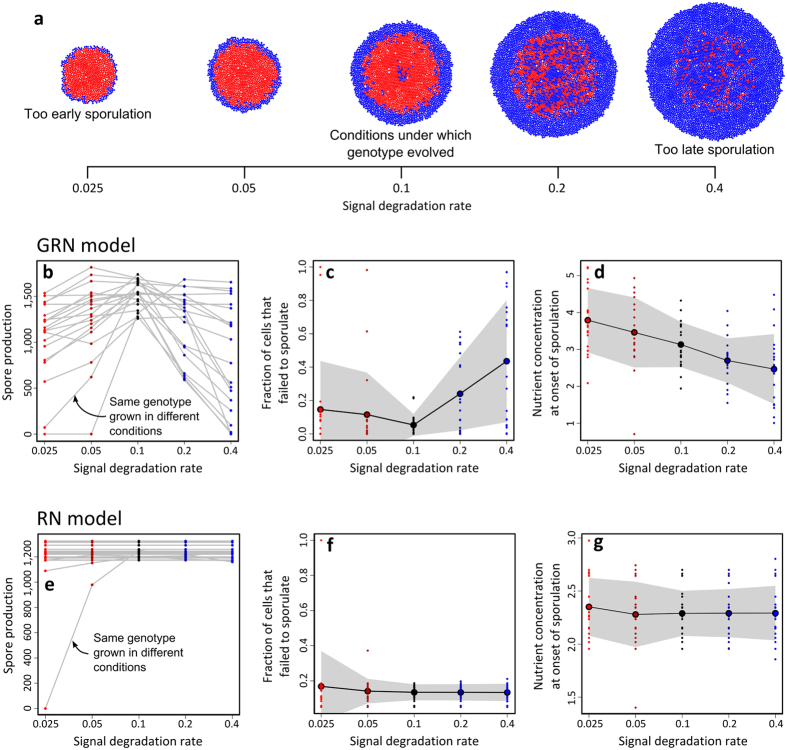
Colonies and the signal degradation rate in the RN and GRN model. For each of the twenty most productive genotypes (see Fig. 6b for their reaction norms), 10 replicate colonies are grown at five signal degradation rates (δ): 0.025, 0.05, 0.1, 0.2 and 0.4. Cells evolved at a signal degradation rate of 0.1. (**a**) Colonies of the most productive genotype in the GRN model at different signal degradation rate (blue = cells and red = spores). (**b**) Average spore production of twenty most productive genotypes over the different signal degradation rates. (**c**) Fraction of cells that failed to sporulate (i.e. having insufficient energy to sporulate). (**d**) Average nutrient concentration at which cells initiate sporulation. The lowest row of graphs show (**e**) the number of spores, (**f**) fraction of failed sporulation attempts and (**g**) average nutrient concentration at onset of sporulation for the twenty most productive genotypes of the RN model.

Before looking at the overall picture, we first will focus on five environments that were generated by changing one parameter only: the signal degradation rate. In contrast to nutrients and energy, signal is not required for the sporulation process, but the local signal concentration can be used by cells to time the onset of sporulation. Thus, by varying the signal degradation rate, the environment that cells perceive is altered, but the selection pressures on the timing of sporulation remain the same (Supplementary Text S3). Figure 8a shows colonies of the most productive genotype from the GRN model at different signal degradation rates. In comparison to the signal degradation rate at which genotypes evolved (δ = 0.1), the spore production of the twenty most productive genotypes of the GRN model varies strongly with the new environment (Fig. 8b–d): whereas some genotypes robustly produce the same high number of spores under all signal degradation rates, others produce much fewer spores when encountering a new signal degradation rate. On average, genotypes tend to postpone sporulation at high signal degradation rates and advance sporulation at low signal degradation rates (e.g. Fig. 8a). High signal degradation rates result in low signal concentrations, which cells associate with high nutrient concentrations (see the negative correlation between the signal and nutrient concentration in the dividing zone of Fig. 7a). By the same token, low signal degradation rates result in high signal concentrations, which cells associate with low nutrient concentrations. Thus, by changing the signal degradation rate, cells get the ‘illusion’ that less or more nutrients are present in the environment and thereby falsely advance or postpone sporulation, which goes at the expense of spore production (Fig. 8b–d; see also Supplementary Text S4 and Supplementary Fig. S13). The most productive genotypes of the RN model are largely insensitive to the signal concentration (Supplementary Fig. S2 and Supplementary Text S1). Accordingly, these genotypes are hardly affected by a change in the signal degradation rate (Fig. 8e–g).

**Figure 9:**
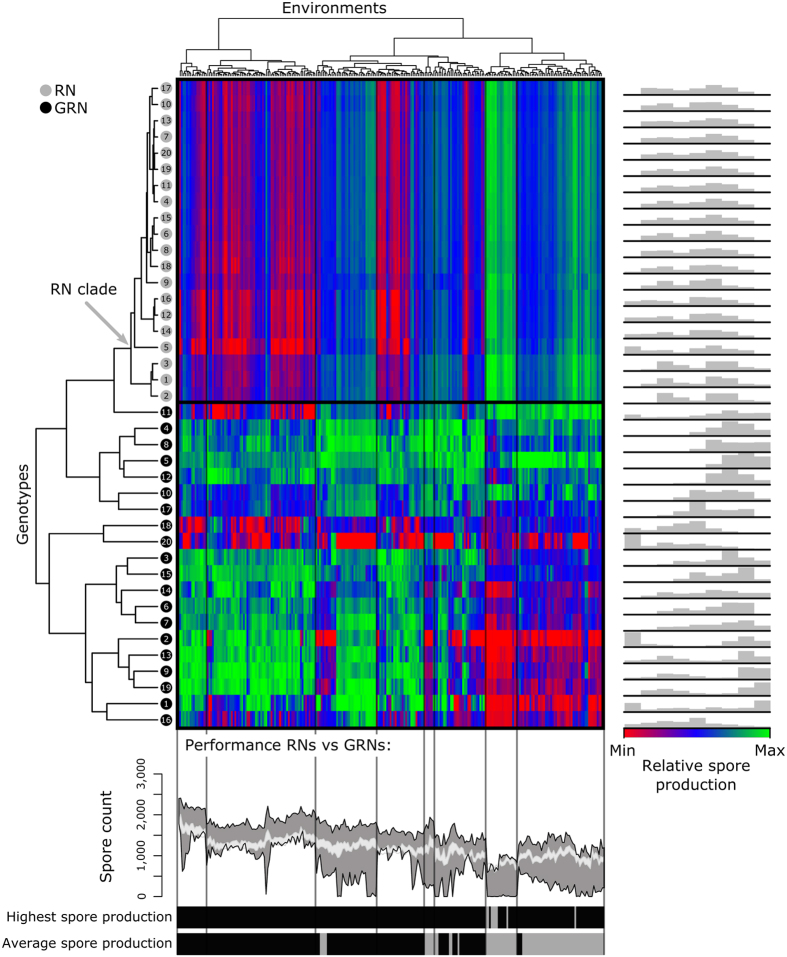
Spore production of GRNs in randomly-generated novel environments. The twenty most productive genotypes of the RN model (grey circles) and GRN model (black circles) were exposed to 250 randomly-generated novel environments (Supplementary Table S2 and Supplementary Data S1). For each environment ten replicate colonies were grown per genotype and the average number of spores at the end of colony growth was used for the cluster analysis see Material and Methods). The colours indicate the relative spore production of genotypes in each novel environment: red = relative low spore production, green = relative high spore production. Histrograms on the right show the distribution of relative spore production for the genotypes. Graph on the bottom shows range of absolute spore production in RN model (light grey area) and GRN model (dark grey area) over all 250 environments. The two bars at the bottom compare genotypes from the RN and GRN model directly, by showing where the most productive genotype comes from (grey = a genotype from RN model, black = a genotype from GRN model) and which set of genotypes produce most spores on average (grey = genotypes from RN model, black = genotypes from GRN model).

Next, we exposed the twenty most productive RN and GRN genotypes to 250 novel environments, which were generated by randomly varying seven model parameters: (1) nutrient diffusion rate, (2) signal diffusion rate, (3) signal degradation rate, (4) nutrient consumption rate, (5) probability of cell division, (6) duration of sporulation and (7) energy consumption during sporulation (Supplementary Table S2 and Supplementary Data S1). Although many of these parameters are not strictly environmental, since they can also be influenced by cellular or molecular factors (e.g. the signal diffusion rate depends on the molecular weight of the signal and the ambient temperature), changes in these parameters do affect the environment to which cells are exposed and the optimal timing of sporulation. As such, they confront cells with unseen environmental conditions. The parameters are varied such that colony growth would not exceed the surface area. In each environment, we determined the relative and absolute spore production of a genotype by growing ten replicate colonies. To evaluate the responses of the evolved genotypes over the 250 environmental conditions, we performed a cluster analysis (see Material and Methods). Genotypes were clustered with respect to their relative spore production over the 250 environments and environments were clustered with respect to the absolute spore production of the 40 genotypes. Genotypes that appear close in the cluster analysis have approximately the same relative spore production – i.e. relative fitness with respect to the other genotypes – in all novel environments. Environments that cluster together have a similar effect on the absolute spore production of all genotypes. Figure 9 shows that the relative spore production of each genotype varies strongly between the novel environments (see histograms on the right of the cluster diagram in Fig. 9). Moreover, the differences in spore production between genotypes from the GRN model are much more pronounced than the differences between genotypes from the RN model. Thus, the higher diversity of responsive strategies in the GRN model (Figs 4 and 6) is translated to a higher diversity in spore production among the tested novel environments (Figs 8 and 9). Even though there are many novel environments for which genotypes from the RN model *on average* perform better than the genotypes from the GRN model, the best performing genotypes nearly always come from the GRN model. In the GRN model, each genotype has a distinct profile along the 250 novel environments. In other words, each responsive strategy is pre-adapted to unique set of environmental conditions. Some genotypes perform relatively well under most novel environments, but never have the highest relative spore production (e.g. genotype 10 from the GRN model). Others perform very well in some environments, but badly in others (e.g. genotype 1 from the GRN model). Importantly, none of the genotypes performs best in all environmental conditions. The diversity in responsive strategy is therefore important for the capacity of a population to cope with many potential future environments.

## Discussion

Plasticity is prominent in all forms of life, but it often remains hard to explain how the mechanisms underlying plasticity evolved^3^. We examined the evolution of plasticity in the context of bacterial sporulation by comparing two alternative model implementations: a classical reaction norm (RN) model and a more mechanistic gene regulatory network (GRN) model. Even though the GRN model accounts for diverse regulatory interactions, the GRN is a strong simplification of the signal transduction cascade that actually underlies sporulation^14^. In both model implementations, cells rapidly evolved the capacity to sense, interpret and respond to the environment by triggering sporulation. The evolved GRNs typically produce more spores than the evolved RNs, in part because many RNs got stuck on local fitness peaks. In the RN model, three distinct evolutionary trajectories were apparent, each making use of different combinations of environmental cues. Apparently, switching from one trajectory to another one is unlikely, because gaining sensitivity to a new cue will typically perturb the current responsiveness of a cell. Such constraints play a minor role in the GRN model, because of the distributed robustness of the network^27^. In the GRN, the decision to sporulate is controlled by three regulatory genes, which together determine the expression of a gene in the output layer. When only one of the regulatory genes is perturbed, due to a mutation, the other genes can still guarantee the accurate timing of sporulation. In the extreme case, when the regulatory wiring of genes is identical, they can even be fully redundant. In that case, loss-of-function mutations of one gene have no consequences for organismal functioning. The GRN therefore allows for mutational robustness, in which cells can accumulate mutations that do not immediately affect their phenotype (i.e. conditionally neutral mutations), but can form a gateway to new adaptive mutations. This form of mutational robustness has been suggested to facilitate the evolvability for a number of biological properties (e.g. RNA molecules, proteins, metabolic networks)^28^,^29^,^30^,^31^,^32^,^33^.

The evolved RNs and GRNs not only differ in their capacity to process direct environmental information, they also differ in their capacity to transmit epigenetic information. Only in the GRN model positive feedback interactions could evolve that allow for the transmission of epigenetic information^26^,^34^,^35^. This form of epigenetic inheritance is common in multicellular development (e.g. heterocycts in *Cyanobacteria*^36^) and distinct from DNA methylation or histone modification, because it relies on self-sustaining regulatory feedback loops that are inherited in time. In our model, epigenetic information is used by cells to adjust their sensitivity to the environment such that they are more likely to continue the sporulation process once initiated. Sporulation is a costly process; switching back and forth is therefore a waste of time and energy. In the absence of the epigenetic feedback, small environmental perturbations during sporulation are sufficient to stop the sporulation process. The epigenetic switch that evolved in the GRN model makes the sporulation process largely irreversible and prevents cells from prematurely stopping sporulation^37^. In rare cases, the epigenetic switch can also be inherited across generations; if a cell divides after its decision to sporulate, both daughter cells will continue the sporulation process. Similar feedback mechanisms also underlie other forms of terminal cell differentiation^38^,^39^. We hypothesize that, quite generally, epigenetic inheritance through self-sustaining loops will be an adaptive strategy when switching back and forth between states is costly: when organisms encounter environmental ‘borderline’ conditions, epigenetics can prevent them from responding in a too sensitive manner to environmental fluctuations, which could otherwise induce repeated switches between states.

A particularly striking difference between the RN and GRN model is the much higher diversity of responsive strategies in the GRN model. Two factors contributed the evolution of this diversity: (i) informational redundancy and (ii) the limited set of conditions cells experience in the colony. Two cues provide redundant information, when the value of one cue can be inferred from the other, which is the case when cues strongly correlate. In our model, the nutrient and signal concentration strongly co-varied in the dividing zone (see Fig. 7). In the presence of informational redundancy, cues become interchangeable, which allows for the evolution of various responsive strategies that express the same adaptive phenotype, but respond to different environmental cues^40^,^41^. The second factor that underlies the diversity in response strategies is the small set of conditions cells encounter inside the colony. Since cells only sense a small number of conditions, only a part of their reaction norm gets exposed, which allows for the accumulation of conditionally neutral mutations elsewhere in the reaction norm (i.e. the so-called ‘hidden reaction norm’ 42). A large fraction of the diversity in responsive strategies remains hidden under the conditions in which organisms evolved. Yet, under novel conditions, this diversity gets exposed^42^,^43^,^44^,^45^. We showed that each responsive strategies is pre-adapted to a specific set of potential future environments^46^. The capacity of a population to cope with environmental change therefore strongly depends on the diversity of responsive strategies that is present in the population^29^,^42^,^43^,^47^,^48^,^49^,^50^. Like previous studies^51^,^52^,^53^, our model emphasizes the important role of environmental information for the evolutionary process. The capacity of organisms to acquire accurate environmental information allows for the evolution of responsive strategies, which can diversify when organisms can sense multiple information sources simultaneously. In reality, bacterial cells sense many more environmental cues than the ones considered in our model^54^,^55^. This poses an interesting question: how much do organisms actually ‘know’ about their environment and how does this affect their evolution?

In our analysis, we focused on the most frequent genotypes that evolved in different replicate simulations. We therefore neglected the stable coexistence of genotypes, while previous studies have shown that the coexistence of responsive strategies can be a likely outcome, especially when individuals engage in social interactions^56^,^57^,^58^. In our model, such coexistence is of marginal importance, since populations repeatedly experience severe bottlenecks during the dispersal phase (Supplementary Text S2). We suspect that, in model implementations with a larger effective population size, a variety of genotypes with similar fitness will coexist, in particular if their responsive strategies are somewhat complementary^57^. Under these conditions, we therefore expect a higher degree of within-population diversity in the GRN model than in the RN model, resulting in a higher adaptive capacity when encountering novel environmental challenges.

## Material and Methods

### Model

#### Colony growth

Cells are placed on a continuous two-dimensional surface with fixed boundaries, which is placed on top of a discrete grid (the model therefore combines continuous and discrete spacing^59^). The grid consists of 200 by 200 equally-sized elements. The radius of a cell (*r*_*cell*_) is 0.8, which makes the ‘surface area’ of a cell (cells are depicted as two dimensional circles in the model) about twice the size of a grid element (which is one by one unit). The grid element on which a cell is placed forms its local environment. The nutrients and signal that are associated with this grid element can be sensed by the cell. In addition, any exchange in the form of nutrient consumption or signal production occurs through the local environment. Cells consume the locally available nutrients with a fixed rate, *V* (see Supplementary Table S1 for model parameters). Nutrients (N) are converted into energy (E); assuming that one unit of nutrients is converted in one unit of energy. When the energy level of a cell passes the threshold level *E*_*d*_, the cell divides with a fixed probability *P*_*d*_. If cell division occurs, the energy necessary for cell division is consumed (*E*_*d*_) and left-over energy is divided equally among the two daughter cells. One of the daughter cells takes in the position of the mother cell and the other one is placed in a random direction next to it. When other cells overlap with the daughter cell, they are pushed aside. Cell pushing is an iterative process, in which cells are randomly selected and examined for their overlap with neighboring cells. If a cell overlaps with its neighbor it is moved such that the overlap between cells disappears. This might result in overlap with other cells. Effectively, cell pushing therefore leads to colony expansion, in which cells continuously push each other aside until cells at the colony edge move outwards and all overlap between cells disappeared (for growth dynamics of colony see^60^). Colonies can never expand beyond the border of the nutrient surface, because individuals will enter dispersal before colony growth can occupy the entire space (*t*_*colony*_).

At the onset of colony growth 100 cells are placed in the spatial center of the surface. Each grid element on the surface is supplied with the same nutrient concentration, *N*_*init*_. During colony growth, cells consume these nutrients, resulting in a gradient with a high nutrient concentration at the colony edge and a low nutrients concentration in the colony center. Nutrients diffuse with a given rate, *D*_*N*_. Hence, the nutrient gradient results in a net flux of nutrients from the colony edge to the center. Like nutrients, signal produced by the cells can also diffuse (*D*_*S*_ = *D*_*N*_, with the exception of Fig. 9). Signal is produced by cells before the onset of sporulation and is secreted in the local environment (Fig. 1). In addition, signal is degraded with a given degradation rate, δ.

A colony can grow for a fixed number of time steps (*t*_*colony*_). After this, cells and spores migrate. We assume that spores are ten times more likely to survive migration than cells. From the individuals that survive migration 100 individuals are randomly chosen for the onset of the next growth cycle. Given that the migratory pool can consist of thousands of individuals, dispersal forms a severe genetic bottleneck. The 100 individuals that initiate a colony might nevertheless have genetic differences.

#### Sporulation

Sporulation requires time (*t*_*spore*_) and energy. In *B. subtilis* sporulation requires for example 6 to 8 hours and involves the expression of hundreds of genes^16^,^61^. In the model, we assume that a fixed amount of energy (*E*_*s*_) is consumed at every time step. When cells have insufficient energy, the sporulation process is stopped. Sporulation can also be prematurely stopped when a cell reverts its sporulation decision, which could follow from a change in the environmental conditions. This has as well been shown in *B. subtilis*, where the first stages of the sporulation process are reversible^37^. Only after forming an asymmetrically positioned polar septum, a cell is irreversibly committed to become a spore^62^. In our model, we assume there are no consequences of a premature stop in the sporulation process, apart from the time and energy that already has been wasted on sporulation. During sporulation a cell continues to consume nutrients, so a part of the energy that is consumed during sporulation gets immediately replenished. When sporulation finishes, a cell is transformed into a mature spore. Mature spores do not consume nutrients and cannot divide, but they are ten times more likely to survive dispersal than cells. In addition, we assume that spores do not respond to the environment. At the onset of a new growth cycles, spores are assumed to germinate and transform back to dividing cells. Thus, all cells are phenotypically identical at the onset of colony growth.

#### Reaction norm model

In the RN model, the cues – nutrient concentration (N), signal concentration (S) and energy level (E) – directly determine whether or not a cell sporulates (Fig. 2a). A cell sporulates when α_N_·N + α_S_·S +  α_E_·E > θ, where θ is the cell’s activation threshold while *α*_N_, *α*_S_, *α*_E_ are the cell’s weighting factors for the corresponding cues. A weighting factor can take on positive and negative values: in case of a positive value, the corresponding cue stimulates sporulation, while it inhibits sporulation in case of a negative value. When the sum of regulatory inputs exceeds the activation threshold, θ, a cell triggers or continues sporulation. The three weighting factors and the activation threshold are encoded by four heritable loci, which evolve under the influence of mutation and selection. At the onset of evolution, we assume that all weighting factors and the activation threshold are 0, hence there is no sporulation. At every cell division, each locus can mutate with probability *μ*. When a mutation occurs, a value is added to the connection weight or activation threshold; this mutational step size is drawn from a normal distribution with mean 0 and standard deviation 0.1 (*σ*).

#### Gene regulatory network model

We consider a GRN that consists of three layers (see Fig. 2b): (i) input layer, (ii) regulatory layer, (iii) output layer. The input layer processes the three cues that cell can sense: N, S and E. The regulatory layer consists of three genes that encode for products that can affect one or several genes in the regulatory or output layer. The output layer consists of one effector gene that determines whether a cell sporulates or not. Genes have a Boolean expression: a gene is either expressed or not, G ∈ {0, 1} (for alternative implementations of the GRN model see Supplementary Text S4). A gene is expressed (G = 1) when the sum of inputs it receives is higher than the genes activation threshold (θ). A gene in the regulatory layer can receive inputs from both the input and regulatory layer, while the gene in the output layer can only receive inputs from the regulatory layer. The input a gene receives is weighted by the connection weights, *w*_*i*_. For example, in case a gene is affected by the nutrient concentration (connection weight *w*_*1*_) and the expression of a gene in the regulatory layer (connection weight *w*_*2*_), the input towards the gene is determined by: *w*_*1*_·N + *w*_*2*_·G, where N is the nutrient concentration and G is the expression state of a regulatory gene. This gene would therefore be expressed if *w*_*1*_·N + *w*_*2*_·G > θ.

Connection weights and activation thresholds are real-numbered values that can be negative or positive. When a connection weight is negative (positive) gene expression is inhibited (stimulated). When the activation threshold of a gene is negative, a gene is expressed by default and its expression can only be prevented through inhibition. In total, the network contains 21 connection weights (9 from the input to the regulatory layer, 9 between genes in the regulatory layer and 3 from the regulatory to the output layer) and 4 activation thresholds (one for each gene). All the connection weights and activation thresholds can mutate, therefore a genotype consists of 25 evolvable loci. At the onset of evolution, we assume that all connection weights and activation thresholds are 0. At every cell division, each locus can mutate with probability *μ*. When a mutation occurs, a value is added to the connection weight or activation threshold that is taken from a normal distribution with mean 0 and standard deviation 0.1 (*σ*).

### Analysis

#### Mutual information

Different response strategies could evolve in both the RN and GRN model. In the RN model the response strategies can easily be determined by examining the weighting factors and activation threshold. In contrast, for the GRNs it is very difficult to determine the response strategy by solely examining the connection weights and activation thresholds. Instead, one can make use of the concept of ‘mutual information’^63^ to understand how a GRN processes environmental information (Fig. 5 and Supplementary Fig. S9). Consider (i) a probability distribution ***p*** over the set of input states *I*, where *p*_*i*_ is the probability of input *i*; (ii) a probability distribution ***q*** over the set of output states *O*, where *q*_*j*_ is the probability of output *j*; and (iii) the joint distribution ***P*** describing the probability *p*_*ij*_ that output *j* co-occurs with input *i*. If inputs and outputs were statistically independent (*p*_*ij*_  = *p*_*i*_*q*_*j*_), knowing the input would not provide any information about the output and *vice versa*. If *p*_*ij*_  ≠ *p*_*i*_*q*_*j*_, knowing the input does provide information on the output. Mutual information, which is defined as


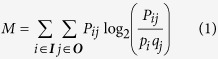


measures the information that inputs and output share, that is, it measures how much knowing the input (resp. the output) reduces uncertainty about the output (resp. the input).

In order to calculate the mutual information for our GRNs, we first discretized the network inputs by dividing the range of possible input values into 50 equally-sized intervals, yielding an input set with 50 discrete intervals: *I = *{1, 2…, 50} Take for example the nutrient concentration, when dividing the range of possible nutrient concentrations (N) into 50 equally-sized intervals, we have all nutrient values between 0 and 10 (*N*_*init*_) with incremental steps of 0.2. Thus, the first interval (*I *= 1) corresponds to N = 0.2, the second interval (*I* = 2) corresponds to N = 0.4, all the way up until the last interval (*I* = 50), which corresponds to N = 10. The output set is already discrete: *O*  = {0, 1} (i.e. sporulation or not). Since the input set is larger than the output set, the maximum information content of inputs (log_2_(50) = 5.64 bits) is larger than the information content of outputs (log_2_(2) = 1 bit). For that reason, the mutual information values are more likely to be constrained by the output of a GRN than by the network’s inputs. Besides the three cues that are processed by the input layer of a GRN (N, S and E), the genetic background of a GRN could also be viewed as an input. There are three regulatory genes, allowing for 8 possible expression backgrounds. The information content of the expression background (log_2_(8) = 3 bits) is therefore lower than that of the three cues.

#### Expression background

For Fig. 5 and Supplementary Fig. S8, we evaluate the responses of GRNs with different expression backgrounds. We discriminated between the expression background of a sporulating and non-sporulating cell. These backgrounds were acquired for each genotype separately, by evaluating the gene expression of cells at the end of colony growth. The expression background (i.e. gene expression in the regulatory layer) of the majority of dividing cells at the end of colony growth was considered to be the typical expression background of a non-sporulating cell. By the same token, the expression background of sporulating cells at the end of colony growth was considered the typical expression background of a sporulating cell. These expression backgrounds were subsequently used to evaluate the performance of a given GRN. In Fig. 5 we evaluated how the fraction of conditions at which a GRN would sporulate depends on the expression background. In Supplementary Fig. S8, we evaluated how the reaction norm generated by a GRN depends on the expression background.

#### Reaction norms and phenograms

In order to assess the diversity of reaction norms that evolved in the 500 replicate simulations, we constructed phenograms. This was done for both the RN model (Fig. 4) and the GNR model (Fig. 6). The phenograms were based on the reaction norms that were associated with the most frequent genotypes present in the 500 replicate simulations at the end of evolution. In case of the GRN model, the reaction norm was determined by exposing each evolved GRN to all possible combinations of network inputs – nutrient concentration (N = [0, 10]), signal concentration (S = [0, 10]) and energy level (E = [0, 15]) – and determining if the GRN would sporulate or not. Since feedback interactions in the regulatory layer of a GRN can result in cyclic changes in gene expression, it would be misleading to evaluate the GRNs at one specific time point. Instead, GRNs were updated 2 to 5 times when evaluating their response to the environment. Finally, one should note that is not possible to associate a unique reaction norm to each GRN, because the GRN’s response to the environment also depends on the expression background of a cell (i.e. gene expression in the regulatory layer). For the reaction norms evaluated in Fig. 6, we simply assume that none of the regulatory genes were expressed before exposure to the environment.

For each pairwise combination of reaction norms, we determined the fraction of conditions at which their associated reaction norms would give a different outcome. This distance measure ranges from 0 (i.e. identical reaction norms) to 1 (i.e. opposite reaction norms). All pairwise differences were combined into a distance matrix, a symmetric matrix with zeros on its diagonal. A neighbor-joining algorithm was used to convert the distance matrix into a phenogram: a tree-like diagram that visualizes the phenotypic differences between the evolved genotypes (using the ‘ape’ library in R version 3.1.1.).

#### Cluster analysis

The cluster analysis of Fig. 9 is based on a different distance measure than the phenograms shown in Figs 4 and 6. The distance between genotypes was measured in terms of relative spore production among the 250 novel environments. The relative spore production of the genotypes was determined for each environment separately (e.g. does a genotype produces the most or the fewest spores in a certain environment?) and compared between the genotypes over all novel environments. The distance between environments was measured in terms of absolute spore production of the 40 genotypes. The distance matrix of both the genotypes and environments were converted in cluster diagrams using a standard hierarchical clustering algorithm (using the ‘ape’ library in R version 3.1.1.).

#### Software

All individual-based simulations are performed in C++ (Microsoft Visual Studio 2010, version 10.0.30319.1). The data is analyzed in R version 3.1.1. Figures are made in inkscape 0.91.

## Additional Information

**How to cite this article**: van Gestel, J. and Weissing, F. J. Regulatory mechanisms link phenotypic plasticity to evolvability. *Sci. Rep.* **6**, 24524; doi: 10.1038/srep24524 (2016).

## Supplementary Material

Supplementary Information

Supplementary Data S1

Supplementary Data S2

Supplementary Data S3
