# Supplementary material for: Regulatory mechanisms link phenotypic plasticity to evolvability
Source: Sci Rep. 2016 Apr 18;6:24524. doi: 10.1038/srep24524 (PMC4834480; doi:10.1038/srep24524)
Supplement: Supplementary Information [file srep24524-s1.pdf]

Supplementary Information

Regulatory mechanisms link phenotypic plasticity to evolvability

J. van Gestel and F.J. Weissing

Content Page

Supplementary Text

Text S1. Diversity in mutual information values in RN and GRN model 2

Text S2. Genotypic diversity in the twenty most productive simulations 6

Text S3. Timing of sporulation 8

Text S4. Alternative modelling implementations of GRN model 10

Supplementary Tables

Table S1. Parameter settings of model 14

Table S2. Parameter ranges used for generating novel environments in Fig. 9. 14

Supplementary Figures

Figures of the RN model

Fig. S1. Spore production and fitness. 15

Fig. S2. Diversity of reaction norms in the RN model. 16

Fig. S3. Mutual information in the RN model. 17

Fig. S4. Twenty most productive genotypes in RN model. 18

Fig. S5. Genetic diversity within and between simulations in the RN model. 19

Figures of the GRN model

Fig. S6. Evolution of sporulation in the GRN model. 20

Fig. S7. Colonies of the twenty most productive genotypes in the GRN model. 21

Fig. S8. Diversity and expression background in the GRN model. 22

Fig. S9. Diversity in mutual information values in evolved GRNs. 23

Fig. S10. Twenty most productive genotypes in GRN model. 24

Fig. S11. Genetic diversity within and between simulations in the GRN model. 25

Fig. S12. Environmental conditions at the onset of sporulation in the GRN model. 26

Fig. S13. Model variants and the accumulation of hidden diversity in GRNs . 27

Supplementary Text S1. Diversity in mutual information values in RN and GRN model.

In Fig. 5 of the main text, we showed the *average* mutual information values of the most frequent genotypes in the GRN model (see also Material and Methods). For comparison, we here determined the same mutual information values for the RN model. Supplementary Fig. S3a shows the *average* mutual information values of the most frequent genotypes in the 500 replicate simulations of the RN model. Since the expression background plays no role in the RN model, Supplementary Fig. S3a only shows the mutual information values with respect to the nutrient concentration, signal concentration and energy level. As for Fig. 5, in the RN model, the *average* genotype depends on all environmental cues for its decision to sporulate or not. The sensitivity to the environment increases within the first 200 generations of evolution.

The mutual information values in Supplementary Fig. S3a do not show the individual differences between genotypes. Yet, from the diversity in reaction norms observed at the end of evolution (Fig. 4), one would expect that genotypes strongly differ in their sensitivity to the environment. This diversity should be reflected as well in the mutual information values that are associated with the *individual* genotypes. Therefore, we also examined the diversity of mutual information values associated with *individual* genotypes in both the RN and GRN model. Before showing this analysis, we first outline a few expectations that one can formulate in advance. First, we know that the space of possible mutual information values is constraint. The mutual information can never be higher than the amount of information (i.e. entropy) present in the output of a reaction norm or gene regulatory network (see Material and Methods). Low mutual information values could indicate that a genotype sporulates for a small fraction or large fraction of conditions and high mutual information values indicate that a genotype sporulates for an intermediate fraction of conditions. Second, we know that there are tradeoffs between the mutual information values of different inputs: a genotype cannot have high mutual information values for two environmental cues

simultaneously. When a genotype's decision depends on multiple cues, it will decrease the mutual information values that are associated with each one of them. Thus, low mutual information values can indicate that the evolved genotype is insensitive to the environment or that it is sensitive to many independent cues. In contrast, high mutual information values indicate that a genotype predominantly responses to one cue only.

Supplementary Fig. S3b shows the mutual information values for the 500 most frequent genotypes at generation 400 in the RN model. Each dot corresponds to one genotype and its placement in the three dimensional volume shows the associated mutual information values. The color of a dot corresponds to the spore production of the associated genotype: red, blue and green indicate a low, intermediate and high spore production respectively. There is a high diversity of mutual information values associated with the genotypes. Most genotypes depend on one or two environmental cues for their decision to sporulate and hardly any genotype is sensitive to all three environmental cues. The twenty most productive genotypes cluster together in space and show high sensitivity to the amount of nutrients in the environment (associated mutual information values are high), while being (nearly) insensitive to the signal concentration and energy level. This fits with the reaction norms and their associated genotypes shown in Supplementary Fig. S2 and S4.

For the GRN model, it is impossible to plot the diversity of mutual information values in a three dimensional volume, while a genotype's decision can also depend on the expression background. Instead, we therefore applied a principal component analysis (PCA) on the mutual information values associated with the evolved genotypes. We not only included genotypes from generation 400, but from the entire time course of evolution (from generation 1 till 400, at intervals of 5 generations). Supplementary Fig. S9 shows the outcome of the PCA. Each data point corresponds to a single genotype and the colors indicate the productivity of the genotypes in terms of spore

production. Supplementary Fig. S9a shows the most frequent genotypes at generation 1, 100, 200, 300 and 400 in the PCA plot. The arrows indicate how the mutual information values are projected on the first two principal components of the PCA. At the onset of evolution (generation = 1), none of the GRNs sporulates, therefore all having mutual information values of zero. Afterwards, some GRNs evolve the capacity to sporulate and predominantly occur at the edges of the PCA plot, indicating that there are high mutual information values between the network inputs and output in these GRNs. Towards the end of evolution, more and more GRNs evolve towards the center of the PCA plot, which suggests that the GRNs respond to multiple environmental cues or sporulate for a small fraction of conditions. When considering the complete PCA (Supplementary Fig. S9b), it becomes apparent that genotypes that produce an intermediate number of spores predominantly occur at the edge of the PCA and genotypes that produce either many or a few spores occur closer to the center of the PCA analysis (Supplementary Fig. S9c). This confirms the above trend (Supplementary Fig. S9a) that over the course of evolution, genotypes first evolve a strong dependency towards one cue for triggering sporulation (resulting in a high mutual information value), but over time integrate information from multiple cues. In addition, the low mutual information values of the most productive genotypes indicate that GRNs decrease the number of conditions for which they sporulate relative to other sporulating genotypes. Supplementary Fig. S9d indeed confirms this view, by showing that the genotypes with an intermediate fitness sporulate for the largest fraction of conditions (this fraction is determined from their associated reaction norms, in which each genotype is evaluated with respect to all possible environmental conditions). One of the most striking contrasts between the RN and GRN model can be found when examining the twenty most productive genotypes. Above we showed that the twenty most productive genotypes in the RN model display nearly identical mutual information values (Supplementary Fig. S3b), in the GRN this is not the case. The twenty most productive genotypes differ strongly in their sensitivity to the environmental cues (Supplementary Fig. S9b). Moreover, most of these genotypes rely on more than one environmental cue for their decision to sporulate, while the genotypes in the RN model were

predominantly influenced by the nutrient concentration in the environment. Thus, as indicated by the associated reaction norms (Fig. 6 and Supplementary Fig. S2), the most productive genotypes in the GRN model display a much wider range of sporulation strategies than the most productive genotypes in the RN model.

Supplementary Text S2. Genotypic diversity in the twenty most productive simulations

In the main text we examined the most frequent genotypes present at the end of evolution, thereby ignoring genotypic variation that is present within the colony. In this section, we characterize genotypic variation both within and between simulations, by focusing on the simulations that are associated with the twenty most productive genotypes (as depicted by Fig. 6, S2, S4 and S10). For both the RN and GRN model, we selected all genotypes present in at least two copies in the colony at the end of evolution. These genotypes were subsequently compared in a pairwise fashion. The genotypic difference between each pair of genotypes, both within and between simulations, was determined by the sum of absolute differences between the evolvable variables. The larger the genotypic difference between two genotypes the more genetic mutations are required to change from one genotype into the other. The genotypic differences were used to make a distance matrix, from which a cladogram could be constructed. Supplementary Fig. S5 and S11 show the cladograms associated with respectively the RN model and GRN model. Genotypes that belong to the same simulation are shown by the same half-transparent color, so in total twenty different colors are present in each cladogram. The size of the dot shows the number of individuals that have a given genotype.

When comparing the cladograms of the RN model and GRN model a few differences become apparent. First, the genotypic variation in the GRN model is much bigger than in the RN model. This is not surprising, because there are more evolvable loci in the GRN model (25 loci) than in the RN model (4 loci). The lower genetic variation in the RN model is also apparent when directly comparing the evolved genotypes (Supplementary Fig. S4 and S10). Second, whereas genotypes from the same simulation strongly cluster together in the GRN model, their clustering is less apparent in the RN model. This implies that the independent evolutionary simulation in the RN model lead to

approximately the same genotypes (i.e. small genetic differences between simulations), whereas different genotypes evolve in the GRN model (i.e. large genetic differences between simulations).

In both the RN and GRN model, the genotypic variation within each simulation is small. This can be explained by the strongly bottleneck that occurs at the onset of each colony growth cycle. Only one hundred individuals (spores or cells) survive migration and can initiate a new colony. Thus, at most one hundred genotypes can survive from one colony growth cycle to the next. Every cycle, a large fraction of the genetic variation is therefore lost. Since the genetic variation within each simulation is fairly small, one can characterize the differences between evolutionary simulations by focusing on the most frequent genotypes only.

Supplementary Text S3. Timing of sporulation

Sporulation requires both time and energy. In order to time the onset of sporulation, cells have to account for both the energy level and nutrient concentration. When cells have insufficient energy the sporulation process is stopped. The amount of energy that cells need to store before the onset of sporulation depends on the nutrient concentration. Cells continue to consume nutrients during the sporulation process. A fraction of the energy that is required for sporulation is therefore directly provided by the environment during sporulation. The signal concentration has no influence on the optimal timing of sporulation, because signal is not required nor consumed during the sporulation process.

Supplementary Fig. S12 shows the average energy level and nutrient concentration at the onset of sporulation for the most-abundant genotypes in 500 replicate simulations. The colors indicate the spore production of the genotypes: a low (red), intermediate (blue) and high (green) spore production. The onset of sporulation is delineated by two zones: (1) a minimal nutrient concentration below which cells should not divide, since otherwise their daughter cells have too little energy to finish sporulation; (2) a nutrient-dependent level of minimal energy, below which cells have insufficient energy to sporulate. The most productive genotypes (green) sporulate at low energy levels and low nutrient concentrations, thereby maximizing the amount of nutrients that are allocated to cell division, while maintaining an efficient sporulation program. Genotypes that produce intermediate numbers of spores (blue) sporulate at either high nutrient concentrations or high energy levels. Genotypes that sporulate at high nutrient concentrations, sporulate relatively early and therefore leave many nutrients in the environment that could have been used for cell division. Genotypes that sporulate at high energy levels, consume a lot of nutrients for nothing, because they accumulate more energy than needed for sporulation. The consumed nutrients cannot be used by other cells for either sporulation or cell division. Thus, overall, the most productive

genotypes postpone sporulation as long as possible, by sporulating at low nutrient concentrations, but they initiate sporulation as soon as there is no further potential for cell division.

Supplementary Text S4. Alternative modelling implementations of GRN model

Different model variants

There are many ways to implement a GRN in a model [1]. In this section, we compare five alternative implementations. One key property of the GRN is gene expression. For the model implementation in the main text, we assumed a Boolean gene expression: a gene is either expressed or not. This assumption greatly simplifies the analysis of evolved networks, because there is a limited set of possible gene expression patterns. In reality, gene expression is typically continuous. Therefore, we examine the robustness of our conclusions in case genes have a continuous gene expression. We assumed that the expression of a gene is described by the function: $G(x) = 1/\left(1 + e^{\,b\,(\theta - x)}\right)$. x is the sum of regulatory input towards a gene, θ determines inflection point of the sigmoidal curve (corresponding to a gene's activation threshold in the Boolean implementation of the GRN) and *b* is proportional to the slope of the sigmoidal curve at the inflection point. θ and *b* form heritable loci that are subject to evolution. Thus, the number of evolvable parameters differs between the Boolean (i.e. connection weights and activation thresholds) and continuous implementation of the GRN (i.e. connection weights, inflection points and slopes).

Besides the implementation of gene expression – Boolean or continuous – we also varied the number of genes in the regulatory layer of the network, the number of parameters that can evolve and the initial conditions of the network. Altogether this resulted in five different model variants.

Model variants

The upper row of graphs in Supplementary Fig. S13a shows the response curves of genes in each of the model variants: the regulatory input to a gene is shown on the x-axis and its response is shown

on the y-axis. The response curves correspond to those at the onset of evolution. On top of the graphs we listed the number of evolvable parameters in the network. Here, a short description of each model variant:

- *Default implementation* – This model variant corresponds to the one we have in the main text. We assume Boolean gene expression and both the connection weights and activation thresholds are subject to evolution.
- *Model variant A* – In this model variant we also assume Boolean gene expression. However, in contrast to the default implementation, we assume that only the connection weights can evolve. In this way, we can examine how the degrees of freedom by which a network can change affect the results.
- *Model variant B* – In this model variant we also assume Boolean gene expression. However, in contrast to the default implementation, we assume that there are four genes in the regulatory layer of the GRN. This is another way to examine how the degrees of freedom affect the evolution of a GRN.
- *Model variant C* – In this model variant we assume continuous gene expression. The connection weights, inflection point (θ) and slope (*b*) can evolve. In addition, we assume the initial response curves of genes to resemble that of genes with Boolean gene expression (Model variant A and B).
- *Model variant D* – In this model variant we assume continuous gene expression. The connection weights, inflection point (θ) and slope (*b*) can evolve. However, in contrast to model variant C, we assume that at the onset of evolution genes show a more gradual response to the regulatory input (Model variant A and B).

Results

For each model variant, we ran 100 replicate simulations for 500 generations. At the end of evolution, we selected the 10 most productive genotypes. These 10 genotypes were grown as mono-clonal colonies at different signal degradation rates. Based on the results in the main text (Fig. 8), we had the following expectations: (i) the variation between the genotypes in terms of spore production is lowest at the signal degradation rate at which cells evolved and higher at alternative signal degradation rates; (ii) the fraction of failed sporulation events is lowest at the signal degradation rate at which cells evolved and higher at alternative signal degradation rates; (iii) at high signal degradation rates cells postpone sporulation (i.e. lower nutrient concentration at onset of sporulation) and at low signal degradation rates cells advance sporulation (i.e. higher nutrient concentration at the onset of sporulation). Model variants A, B and C all satisfied the above expectations (Supplementary Fig. S13b). Only model variant E produced different results. In this model variant, genotypes did express a higher diversity in spore production and failed sporulation attempts at alternative signal degradation rates, but they did not postpone (advance) sporulation at high (low) signal degradation rates. How can these results be explained? Model variant E differs from the other model variants in the initial response curve of genes (Supplementary Fig. S13a). In contrast to the other model variants, genes only weakly change their expression in response to changes in their regulatory input. As a consequence, it is difficult to evolve positive feedback interactions in the regulatory layer. Like explained in the main text, positive feedback interactions are necessary to ensure that cells continue the sporulation process in the presence of small environmental perturbations. As such, they are also necessary for cells that rely on the signal concentration for triggering sporulation. Cells stop producing signal after initiating sporulation. Sporulating cells will therefore experience a drop in the signal concentration, which can trigger cells to stop sporulating. If signal-responsive cells want to continue the sporulation process after its initiation, they need positive feedback interactions in the regulatory layer. Since these positive feedback interactions are difficult to evolve in model variant E, cells cannot evolve a dependency on

the signal concentration, which explains why the evolved genotypes do not change the timing of sporulation when changing the signal degradation rate in model variant E.

References

1. Spirov, A. & Holloway, D. Using evolutionary computations to understand the design and evolution of gene and cell regulatory networks. *Methods* **62,** 39–55 (2013).

Supplementary Tables:

Supplementary Table S1. Parameter settings of model

Parameter	Description	Value
N_{init}	Nutrient concentration at onset	10
r_{cell}	Cell radius	0.8
D_N	Diffusion rate of nutrients	0.1
D_S	Diffusion rate of signal	0.1
δ	Signal degradation rate	0.1
V	Nutrient consumption rate	0.1
P_d	Probability of cell division when sufficient energy	0.5
E_d	Energy level at cell division	10
E_s	Energy sporulation (per time step)	0.5
t_{spore}	Duration of sporulation (in time steps)	5
t_{colony}	Duration of colony growth[1]	125
μ	Mutation rate	0.0015
σ	Standard deviation of mutational step size	0.1

[1] The time duration is chosen such that colony does not exceed the surface boundaries

Supplementary Table S2. Parameter ranges used for generating novel environments in Fig. 9. Parameter conditions are drawn from a uniform distribution between the minimum and maximum value.

Parameter	Default value	Minimum value	Maximum value
D_N	0.1	0.05	0.15
D_S	0.1	0.05	0.15
δ	0.1	0.05	0.15
V	0.1	0.095	0.105
P_d	0.5	0.25	0.75
t_{spore}	5	3	7
E_s	0.5	0.25	0.75

Supplementary Figures:

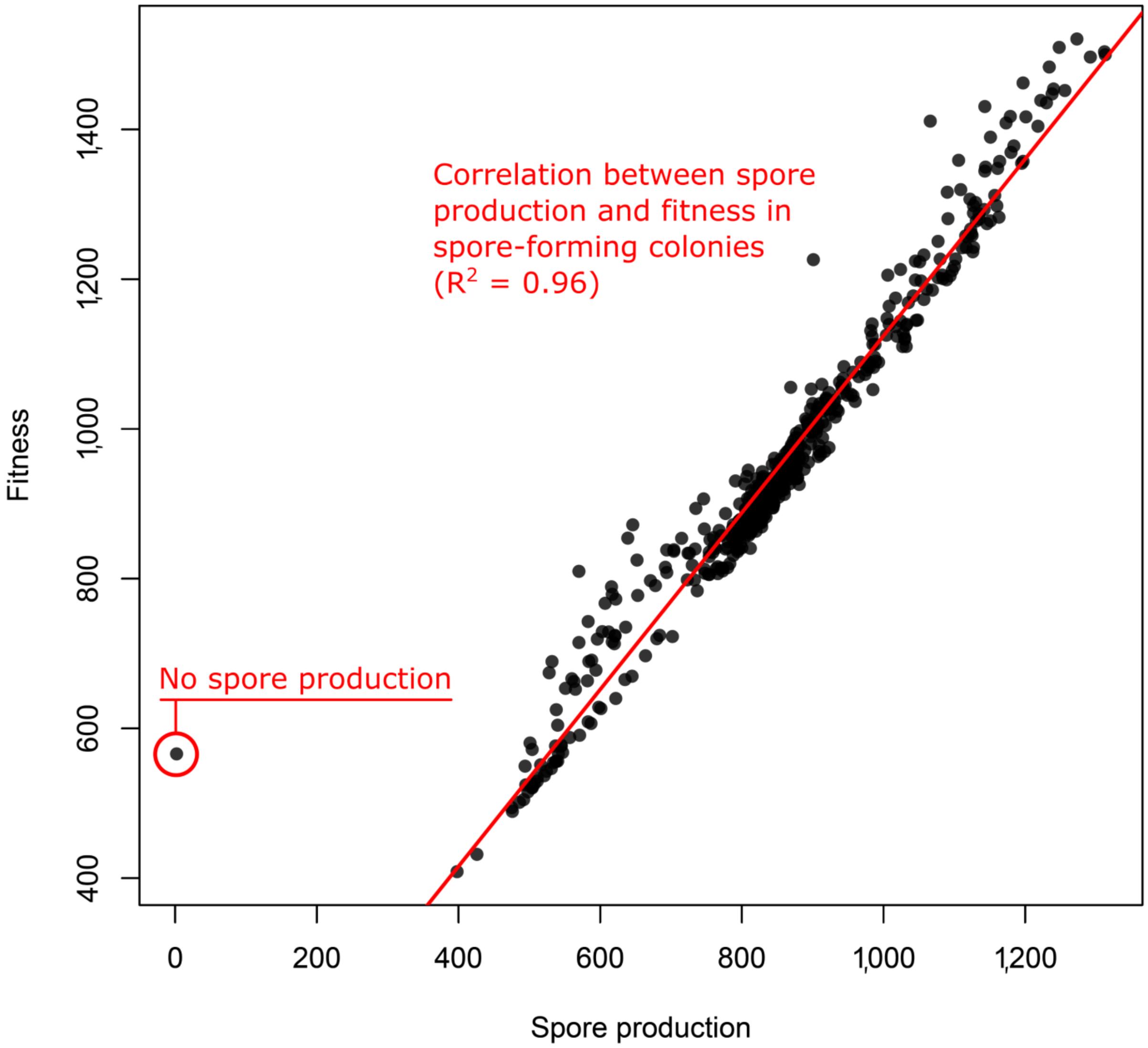

Supplementary Figure S1. Spore production and fitness. The relationship between fitness and spore production for the 500 replicate simulations in the RN model at the end of evolution (generation = 400). Fitness is given by the number of spores plus a 10% fraction of the cell count, because cells have a relative chance of 10% to survive dispersal.

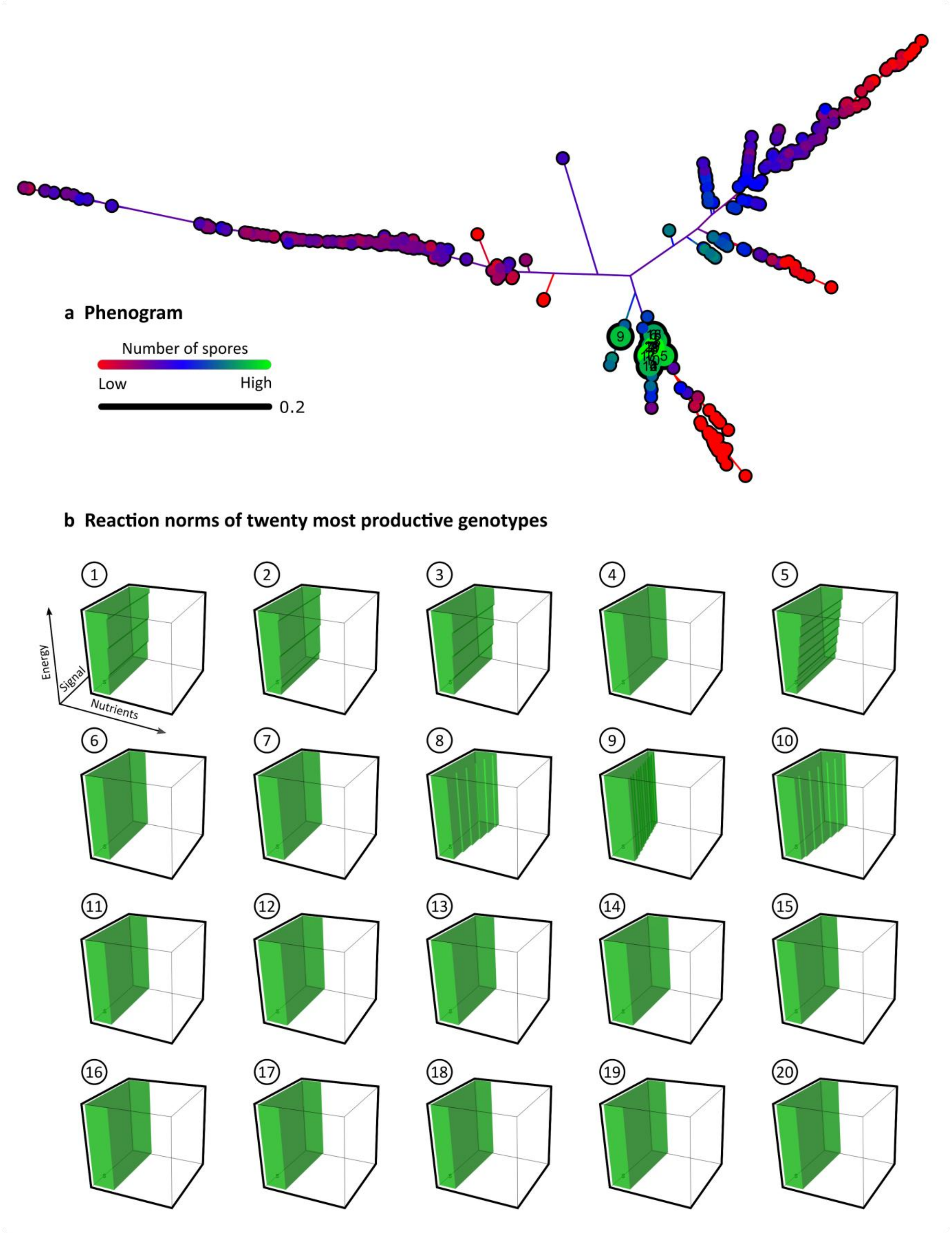

Supplementary Figure S2. Diversity of reaction norms in the RN model. (**a**) Phenogram based on the distance between reaction norms of the most frequent genotypes in the 500 replicate simulations at the end of evolution. The distance between two reaction norms is given by the fraction of conditions at which they prescribe a different response. Colors indicate spore production

of genotypes: low (red), intermediate (blue) and high (green). The twenty most productive genotypes are shown by larger dots. (**b**) The reaction norms associated with the twenty most productive genotypes ranked from the genotype that produces the largest number of spores (1) to the one that produces the smallest number of spores (20).

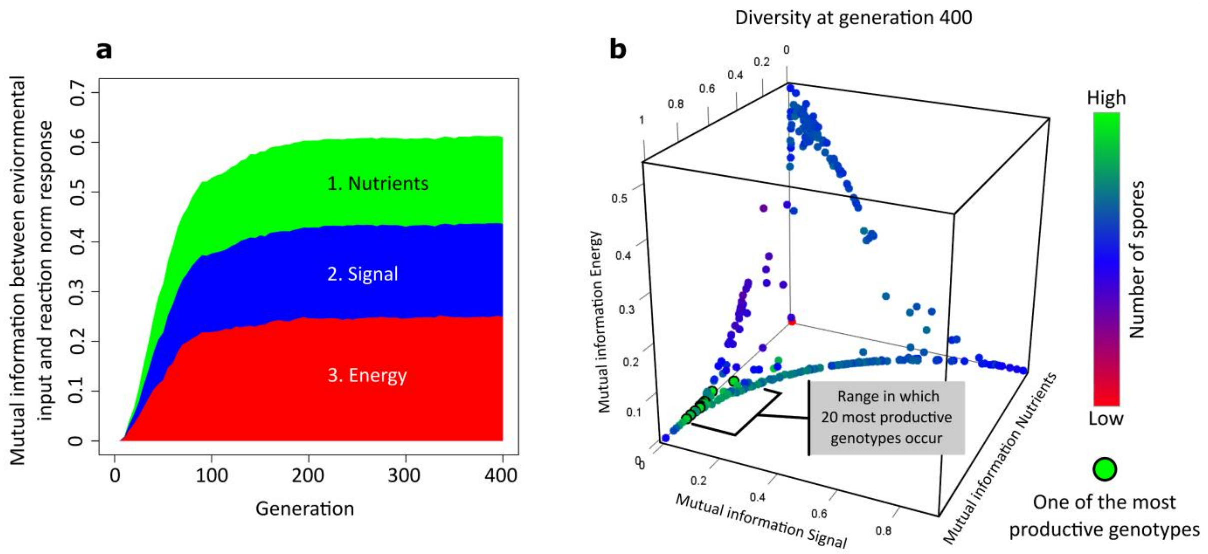

Supplementary Figure S3. Mutual information in the RN model. (**a**) Average mutual information between environmental input – (1) nutrients (green area), (2) signal (blue area), (3) energy (red area) – and a cell's decision to sporulate (see Supplementary Text S1 for details). The mutual information values were calculated for the most frequent genotype in each replicate simulation and averaged over all 500 replicate simulations. (**b**) Diversity in mutual information values at generation 400. Each dot represents a single genotype, associated with one replicate simulation. The color indicates the productivity of this genotype: red indicating no spore production and green indicating a high spore production. The dots associated with the twenty most productive genotypes have a black outline and cluster together in the three dimensional volume, as indicated by the grey box.

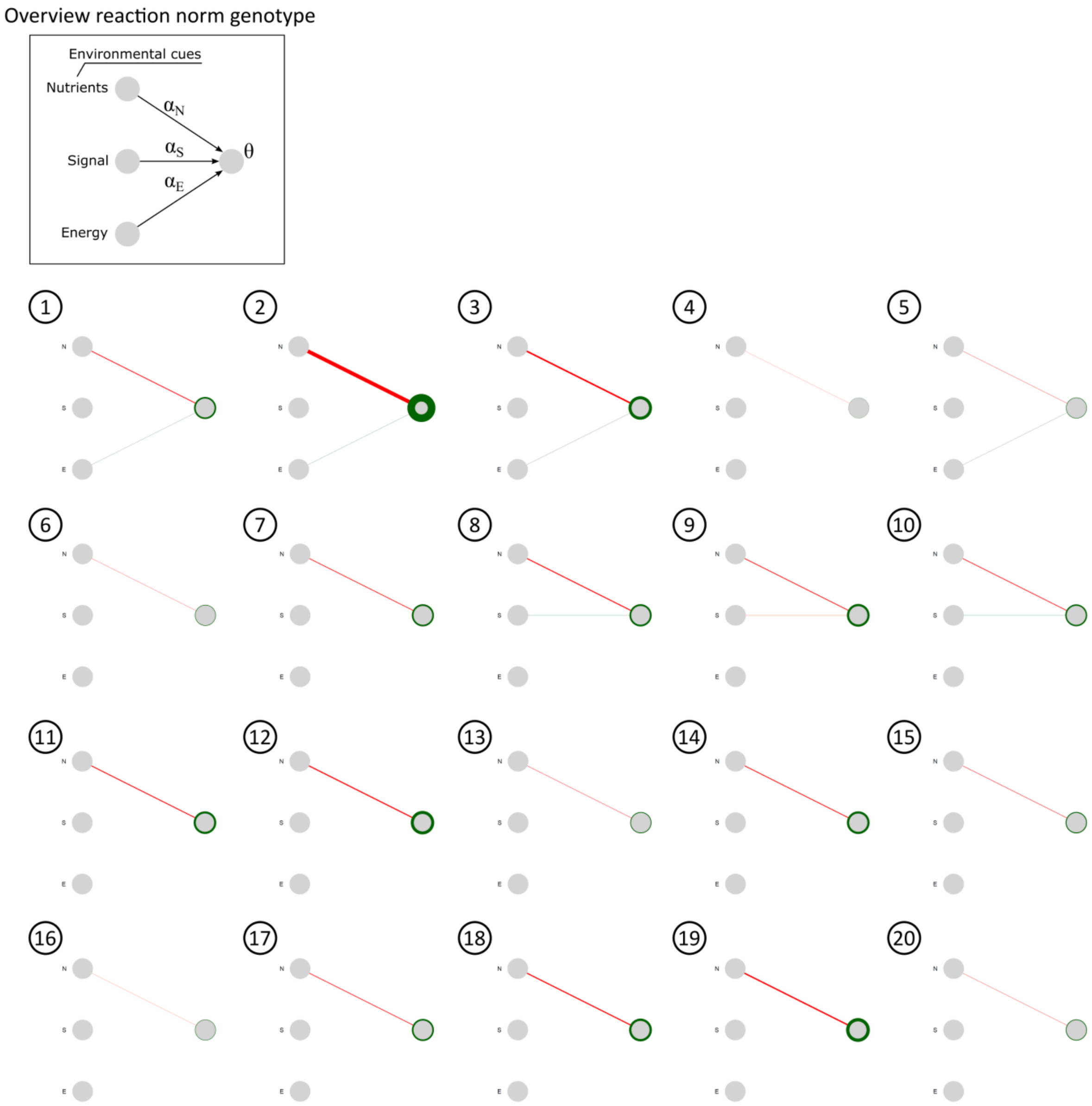

Supplementary Figure S4. Twenty most productive genotypes in RN model. In the RN model there are four evolvable loci: three weighting factors (α_N, α_S, α_E) and one activation threshold (θ). Weighting factors can be inhibitory (red) or stimulatory (green). The strength of the interaction is shown by the width of the line. The activation threshold can either be negative (green) or positive (red). When the activation threshold is positive, a cell does not sporulate unless it receives a stimulatory input. When the activation threshold is negative, a cell sporulates by default and sporulation can only be prevented through inhibition. For genotypic variables see also Supplementary Data S2.

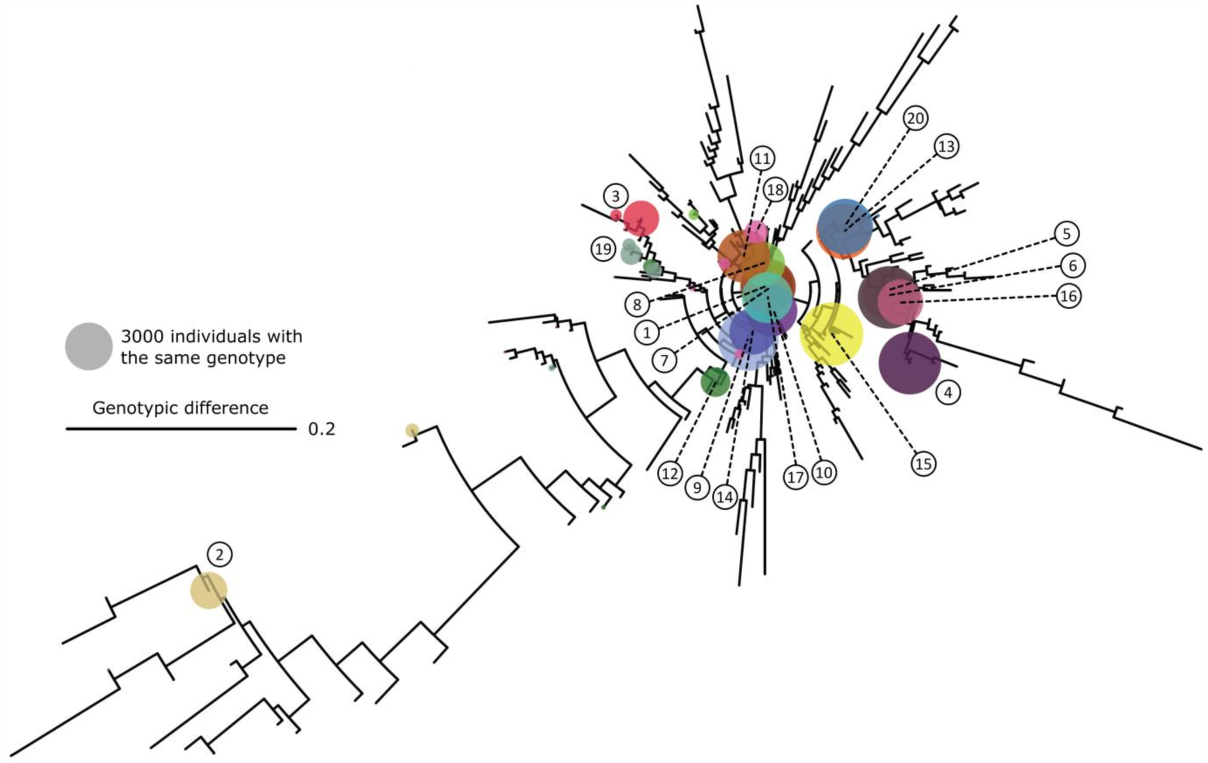

Supplementary Figure S5. Genetic diversity within and between simulations in the RN model. Diversity measured within and between simulations that are associated with the twenty most productive genotypes (Supplementary Fig. S2). The cladogram includes all genotypes that occur in more than one copy at generation 400, thus not only the most frequent genotypes. Each dot corresponds to a single genotype. Genotypes belonging to the same simulation are shown with the same color. The size of the dots indicates how many individuals within the colony have the given genotype. The numbers indicate the twenty simulations (including their most productive genotypes).

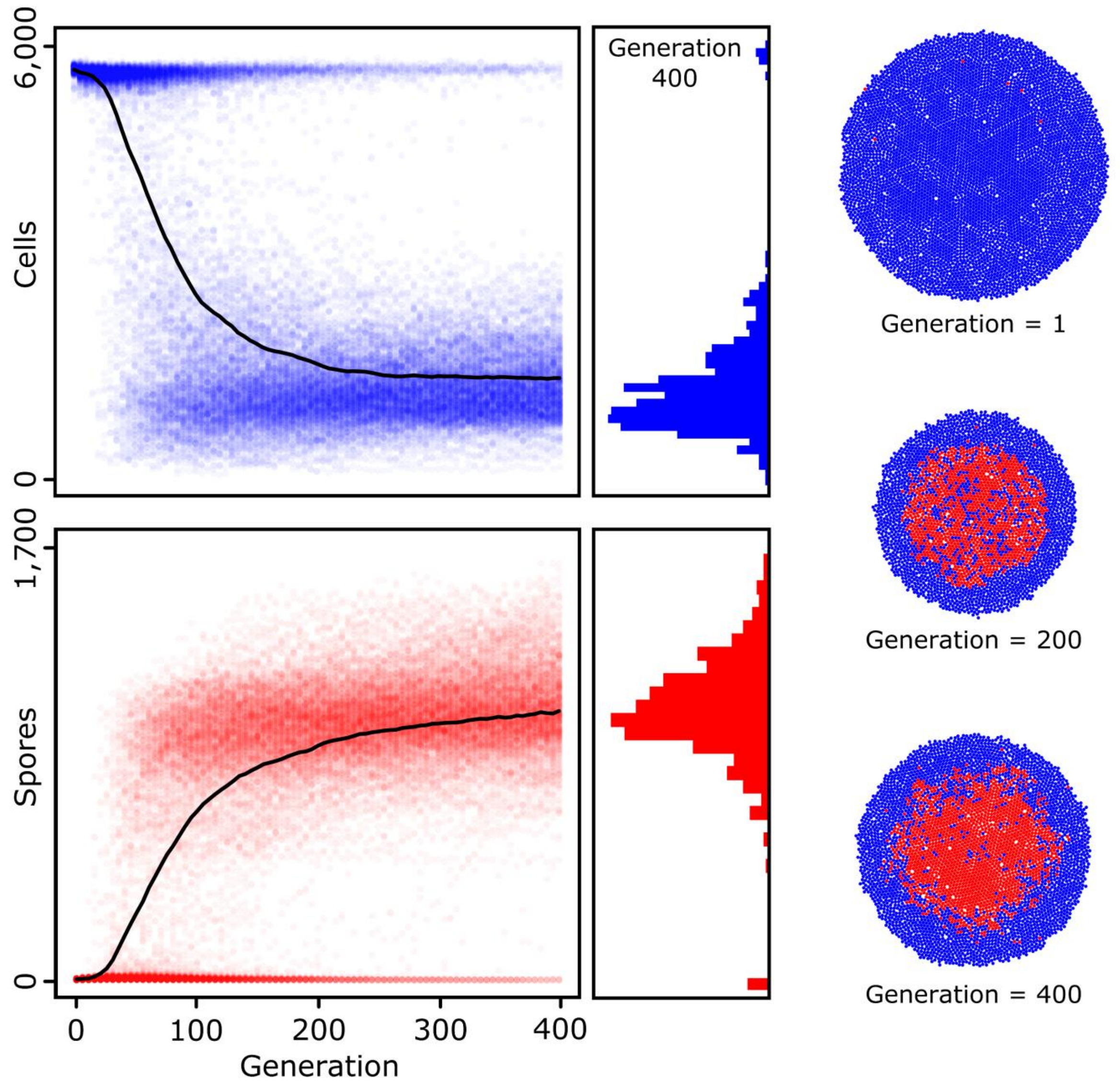

Supplementary Figure S6. Evolution of sporulation in the GRN model. (left) Number of cells (blue) and spores (red) in 500 replicate simulations over the course of 400 generations. At each generation, cell and spore counts are collected at the end of colony growth. The black lines show the average number of cells and spores. (middle) Distributions of the number of cells and spores over the 500 replicate simulations at the end of evolution. (right) Colonies of the most productive genotype at generation 1, 200 and 400.

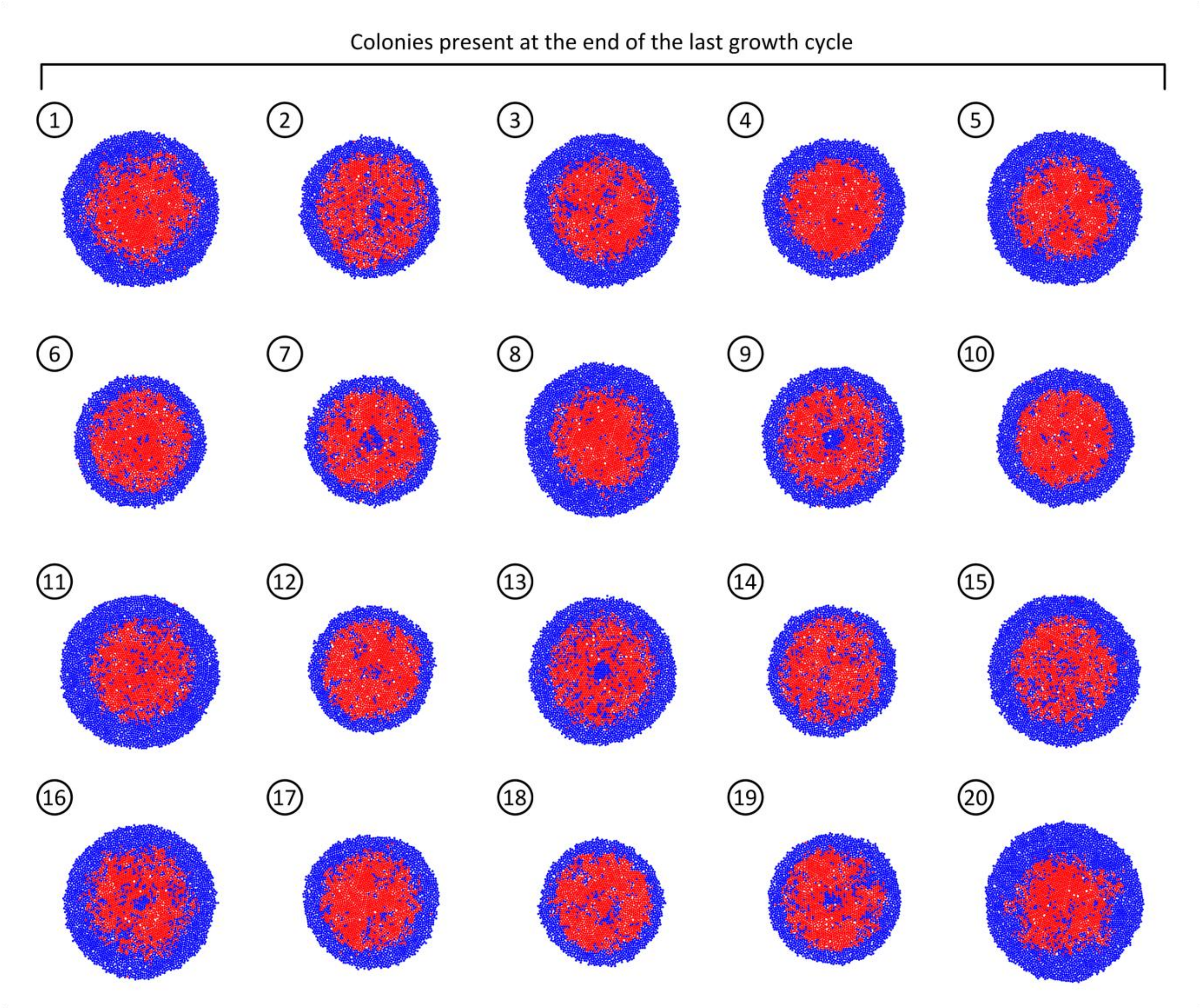

Supplementary Figure S7. Colonies of the twenty most productive genotypes in the GRN model. Colonies at the end of colony growth associated with the twenty most productive genotypes, showing cells (blue) and spores (red). The colonies correspond to the last cycle of the evolutionary process and therefore may contain multiple genotypes.

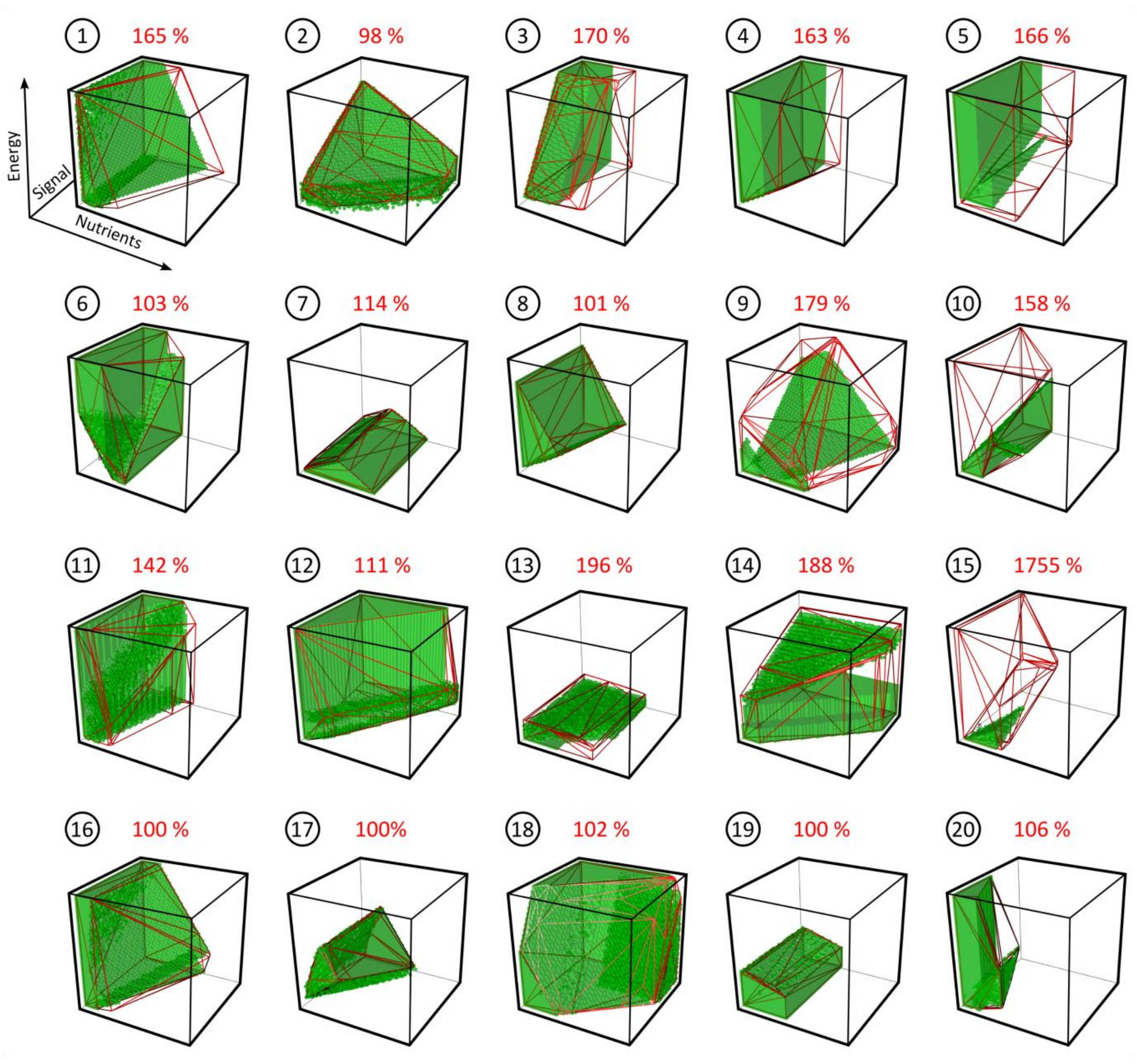

Supplementary Figure S8. Diversity and expression background in the GRN model. Fig. 6b shows the reaction norms that are generated by the twenty most productive genotypes, assuming that none of the genes in the regulatory layer are expressed before cells are evaluated with respect to all combination of N, S and E (i.e. expression background of a non-sporulating cell). In this figure, the same GRNs are evaluated with the expression background of a sporulating cell (see Material and Methods). The new reaction norms are shown as red meshes on top of the original green reaction norms. In most cases, cells sporulate for a larger number of conditions when having the expression background of a sporulating cell. The percentage on top of each reaction norms indicates the relative volume of the red reaction norm (i.e. expression background of sporulating cell) with respect to the green one (i.e. expression background of non-sporulating cell).

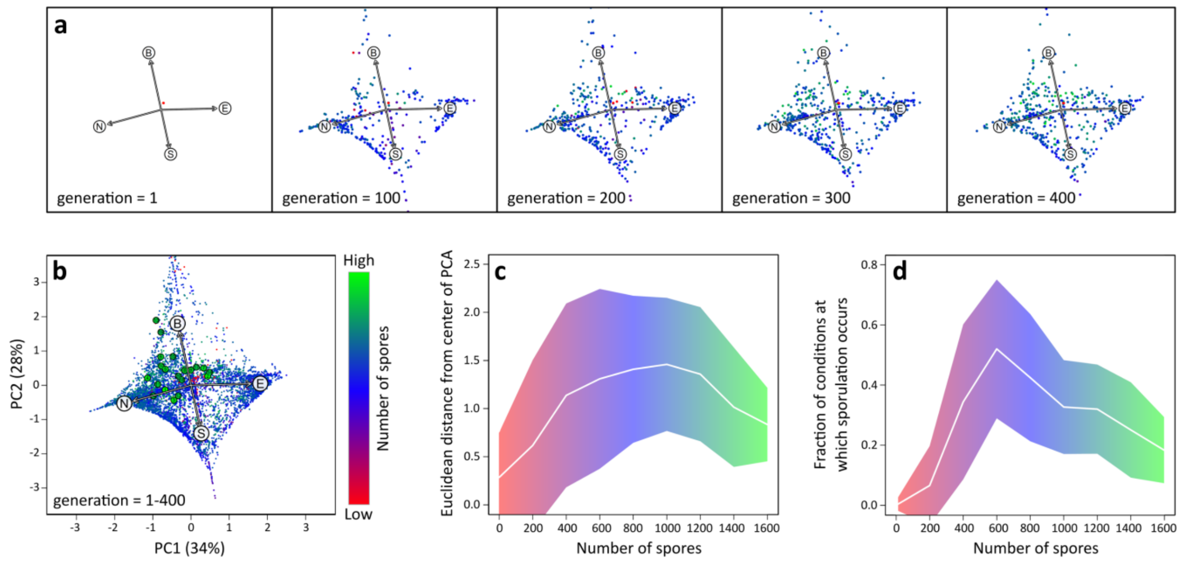

Supplementary Figure S9. Diversity in mutual information values in evolved GRNs. In Fig. 5a we evaluated the *average* mutual information values of the evolved GRNs over the course of 400 generations. Here, we analyse the *individual* mutual information values that are associated with the evolved GRNs using a principal component analysis (PCA). The principle component analysis is based on all GRNs evaluated in Fig. 5a, which includes the most frequent genotypes in the 500 replicate simulations, collected at intervals of 5 generations over the entire course of evolution. The inner axes of the PCA show the relation between the mutual information values and the first two principal components: N = nutrients, S = signal, E = energy, B = gene expression background. Each genotype forms a single data entry (i.e. data point) to the PCA and is associated with four mutual information values (see Supplementary Text S1 for details). The color indicates the relative spore production of a genotype. (**a**) Four PCA plots showing the 500 most frequent genotypes at respectively generation 1, 100, 200, 300 and 400. (**b**) PCA plot showing all data entries. The twenty most productive genotypes at the end of evolution are highlighted by the larger data points. (**b**) The relationship between a genotype's spore production and the Euclidean distance (mean ± SD) of the associated data point in the PCA from the centre of the PCA (see Supplementary Text S1 for details). (**d**) The relationship between a genotype's spore production and the fraction (mean ± SD) of environmental conditions (evaluated using the associated reaction norms, e.g. Fig. 6) at which the associated GRN sporulates.

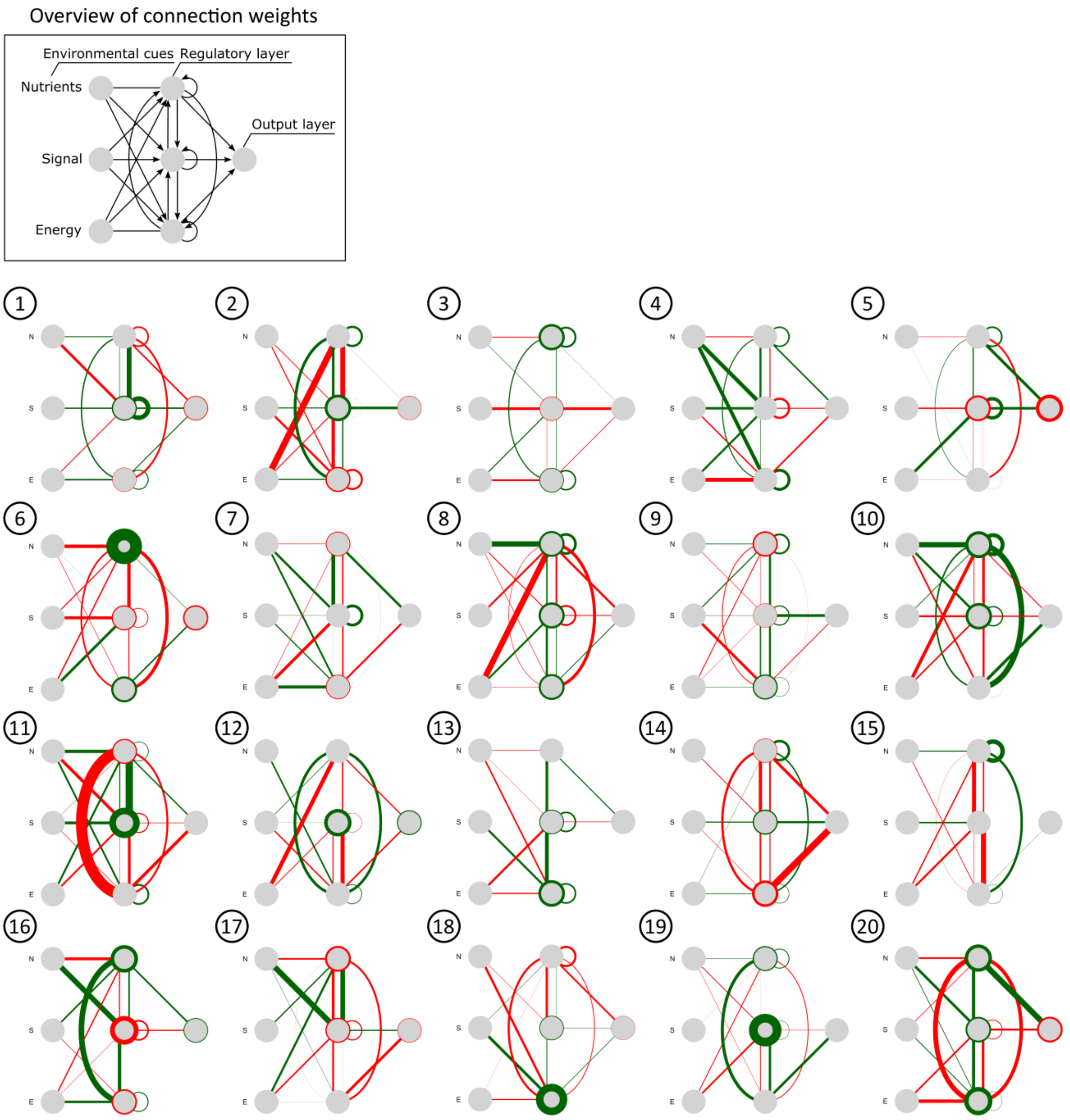

Supplementary Figure S10. Twenty most productive genotypes in GRN model. In the GRN model there are 25 evolvable loci: 21 connection weights and 4 activation thresholds (associated with three genes in the regulatory layer and one in the output layer). Connection weights can be inhibitory (red) or stimulatory (green). The strength of the interaction is shown by the width of the line. The activation threshold can either be negative (green) or positive (red). When the activation threshold is positive, a gene is not expressed unless it receives a stimulatory input. When the activation threshold is negative, a gene is expressed by default and sporulation can only be prevented through inhibition. For genotypic variables see also Supplementary Data S3.

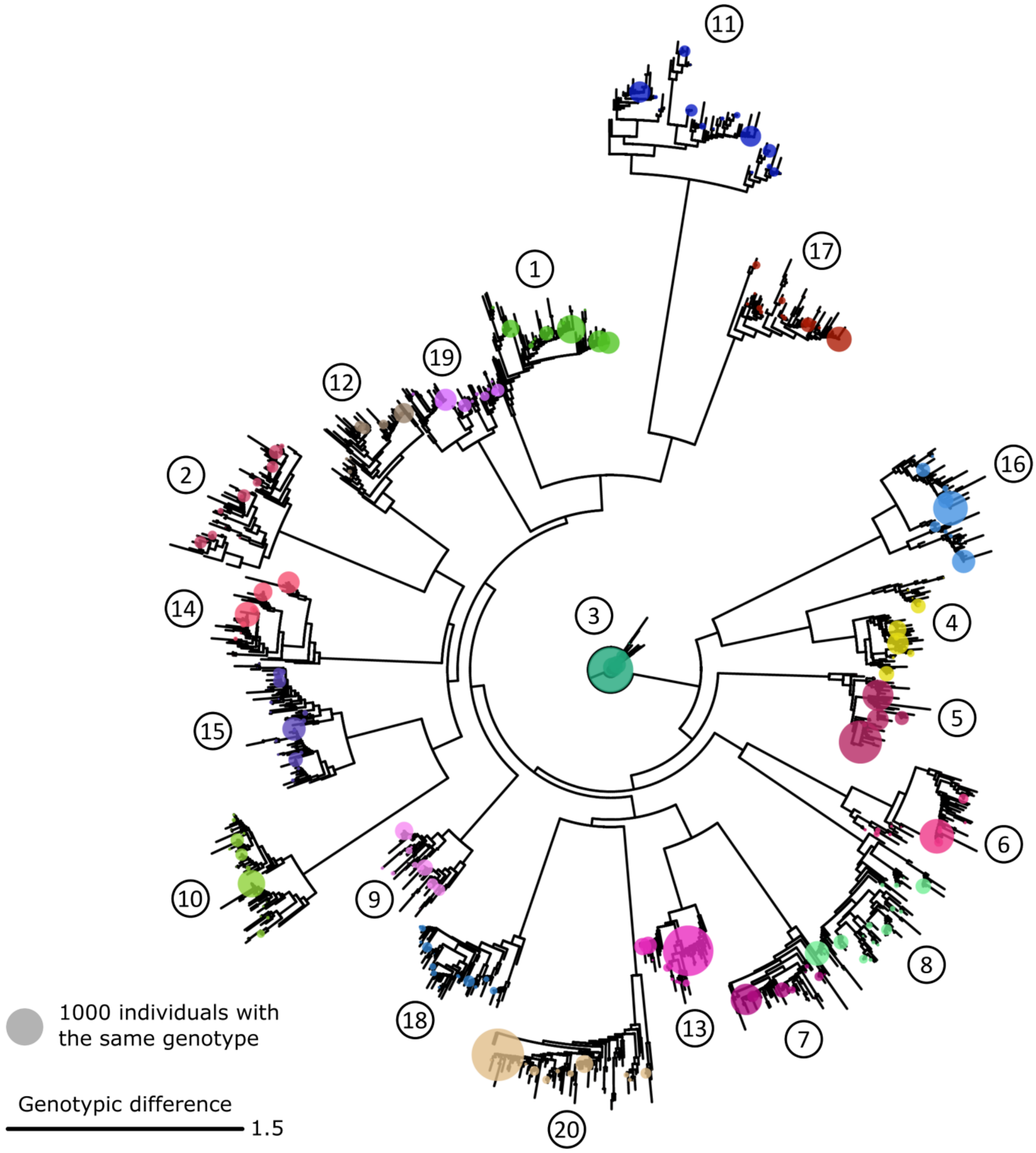

Supplementary Figure S11. Genetic diversity within and between simulations in the GRN model. Diversity measured within and between simulations that are associated with the twenty most productive genotypes (Fig. 6). The cladogram includes all genotypes that occur in more than one copy at generation 400, thus not only the most frequent genotypes. Each dot corresponds to a single genotype. Genotypes belonging to the same simulation are shown with the same color. The size of

the dots indicates how many individuals within the colony have the given genotype. The numbers indicate the twenty simulations (including their most productive genotypes).

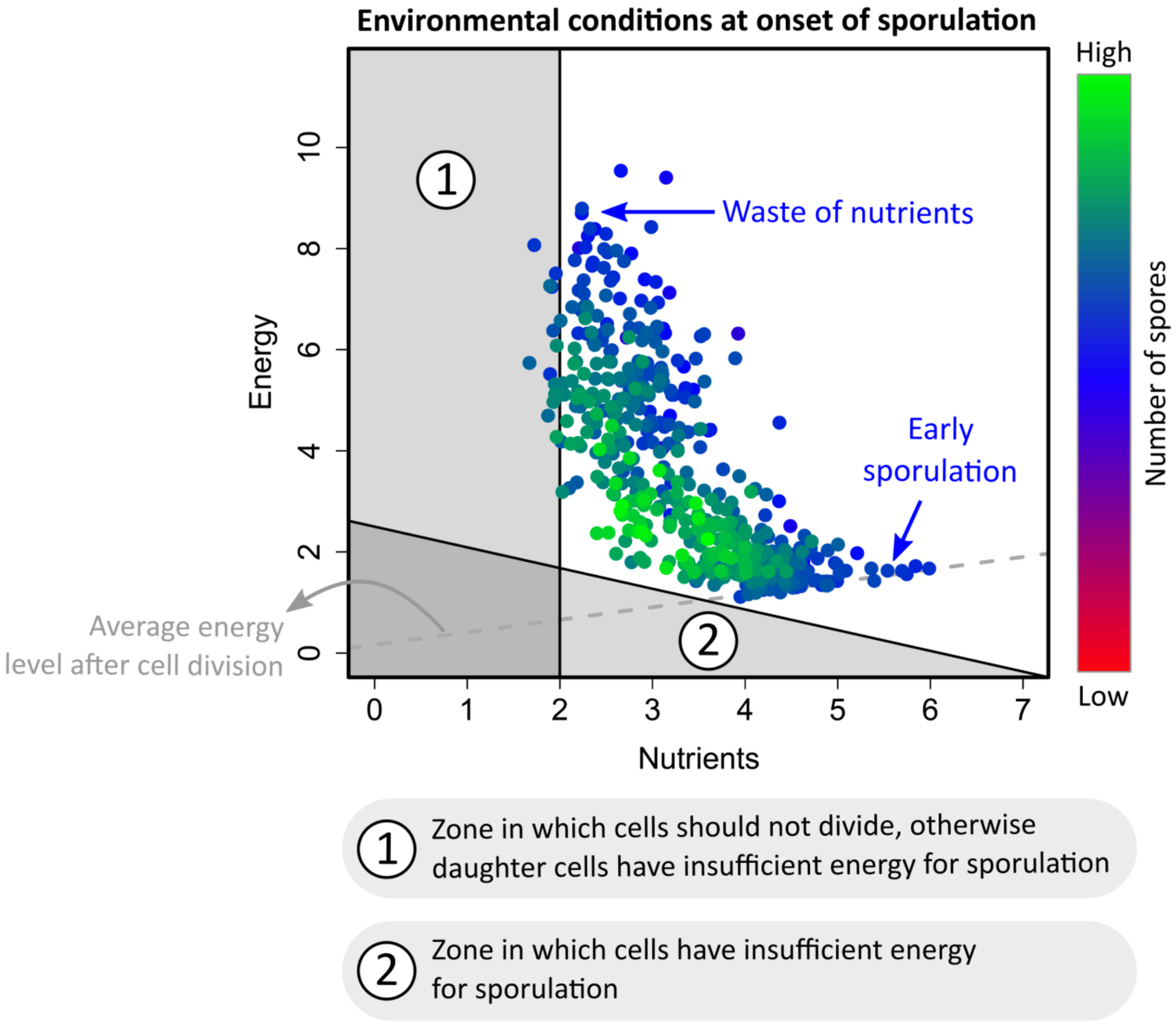

Supplementary Figure S12. Environmental conditions at the onset of sporulation in the GRN model. The average nutrient concentration and energy level at which the most frequent genotypes initiate sporulation at the end of evolution. Colors indicate the relative spore production of the associated GRNs. The range of conditions at which sporulation occurs is delineated by two zones: (1) a minimal nutrient concentration below which cells should not divide, since otherwise their daughter cells have too little energy to finish sporulation; (2) a nutrient-dependent level of minimal energy, below which cells have insufficient energy to sporulate. Dotted line shows the average energy level of daughter cells after cell division (see Supplementary Text S3 for details).

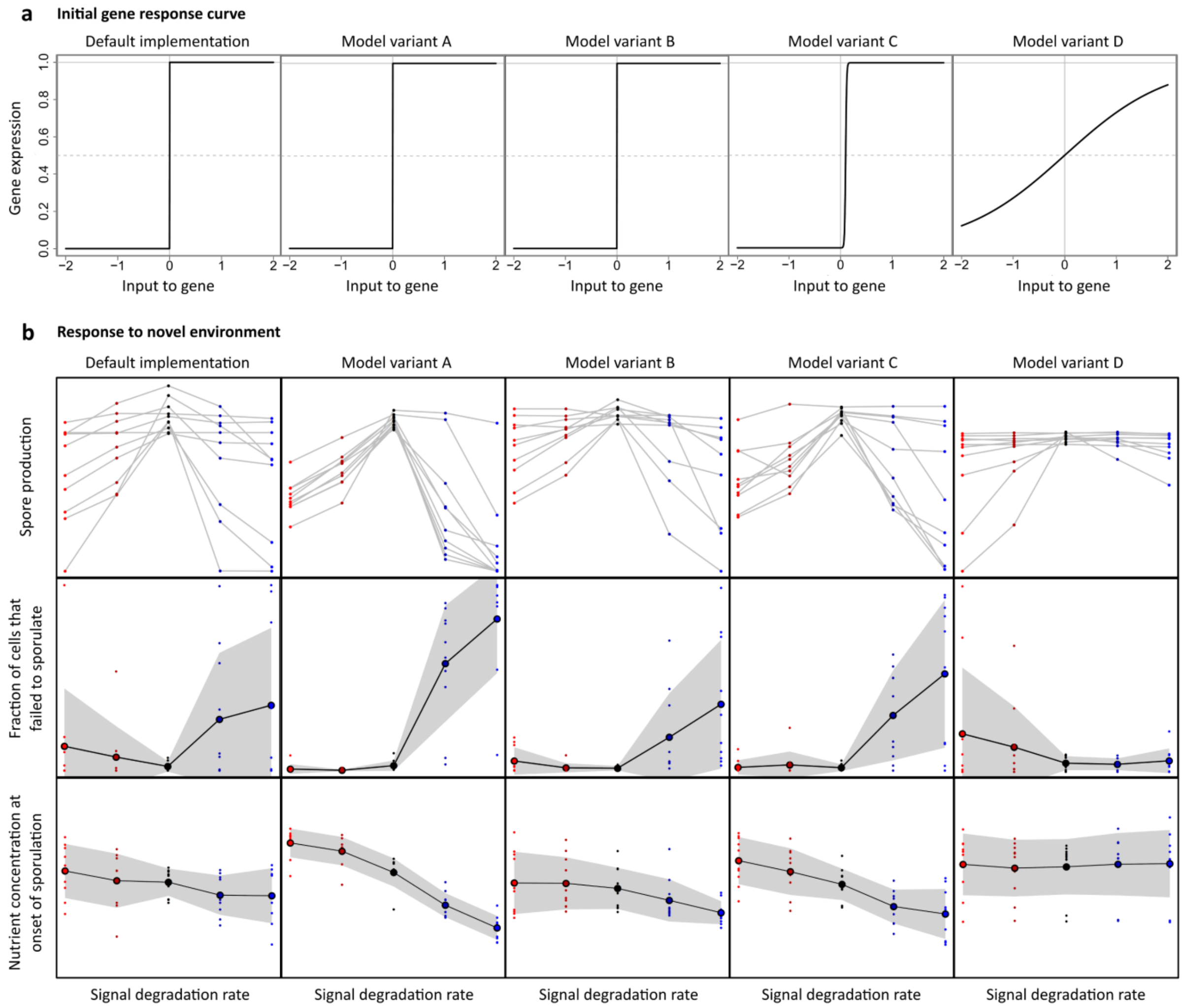

Supplementary Figure S13. Model variants and the accumulation of hidden diversity in GRNs. The exposure of hidden variation in response to changes in signal degradation rate is discussed in the main text for one implementation of the GRN (i.e. default implementation; Fig. 8): Boolean gene expression in which both the connection weights and activation thresholds can evolve. Here we show the results for 4 alternative implementations of the GRN: model variant A-D. (**a**) The initial response curves of genes at the onset of evolution for the different model implementations (model variant A and B have Boolean gene expression and model variant C and D have continuous gene expression, but with different initial conditions). (**b**) The relationship between the signal degradation rate and the (i) spore production, (ii) fraction of failed sporulation attempts and (iii) average nutrient concentration at onset of sporulation for the ten most productive genotypes in the different model

variants (see also Fig. 8). The ten most productive genotypes were collected at the end of evolution among 100 replicate simulations (for details see Supplementary Text S4).
