# Supplementary material for: Regulatory mechanisms link phenotypic plasticity to evolvability
Source: Sci Rep. 2016 Apr 18;6:24524. doi: 10.1038/srep24524 (PMC4834480; doi:10.1038/srep24524)
Supplement: Supplementary Data S1 [file srep24524-s2.doc]

# PARAMETERS USED FOR FIGURE 9

D_N = Diffusion rate of nutrients

D_S = Diffusion rate of signal

delta = Signal degradation rate

V = Nutrient consumption rate

P_d = Probability of cell division when sufficient energy

t_spore = Duration of sporulation (in time steps)

E_s = Energy sporulation (per time step)

D_N D_S delta V P_d t_spore E_s

0.13 0.11 0.13 0.104 0.46 7 0.34

0.07 0.13 0.08 0.102 0.53 5 0.46

0.07 0.05 0.14 0.102 0.6 6 0.6

0.1 0.13 0.08 0.102 0.49 7 0.44

0.11 0.06 0.12 0.096 0.62 5 0.38

0.06 0.14 0.09 0.096 0.48 7 0.46

0.1 0.08 0.12 0.105 0.41 5 0.38

0.12 0.05 0.12 0.105 0.26 3 0.35

0.1 0.11 0.08 0.099 0.37 6 0.68

0.09 0.1 0.11 0.102 0.54 5 0.52

0.09 0.12 0.13 0.102 0.49 7 0.59

0.06 0.09 0.07 0.097 0.31 7 0.34

0.08 0.05 0.15 0.1 0.41 3 0.7

0.09 0.06 0.07 0.101 0.44 6 0.5

0.13 0.12 0.11 0.105 0.59 3 0.48

0.13 0.07 0.12 0.105 0.4 3 0.28

0.1 0.13 0.13 0.103 0.58 4 0.71

0.09 0.11 0.09 0.1 0.34 7 0.45

0.12 0.09 0.07 0.097 0.25 6 0.26

0.12 0.06 0.06 0.098 0.48 5 0.28

0.1 0.15 0.09 0.097 0.59 7 0.75

0.14 0.05 0.06 0.1 0.28 7 0.59

0.06 0.12 0.09 0.104 0.66 7 0.6

0.15 0.06 0.12 0.097 0.48 4 0.38

0.1 0.05 0.1 0.102 0.68 7 0.62

0.08 0.08 0.08 0.105 0.67 5 0.65

0.11 0.08 0.1 0.105 0.57 5 0.37

0.12 0.08 0.13 0.105 0.4 3 0.26

0.06 0.13 0.05 0.096 0.26 5 0.37

0.11 0.11 0.12 0.097 0.75 7 0.64

0.14 0.07 0.11 0.104 0.5 3 0.56

0.14 0.08 0.15 0.105 0.46 3 0.63

0.13 0.12 0.06 0.098 0.55 7 0.42

0.05 0.07 0.1 0.098 0.64 5 0.68

0.13 0.07 0.14 0.102 0.3 5 0.56

0.06 0.1 0.13 0.104 0.44 6 0.3

0.08 0.06 0.05 0.1 0.72 7 0.5

0.14 0.13 0.07 0.1 0.62 7 0.57

0.07 0.13 0.12 0.098 0.67 7 0.44

0.06 0.14 0.11 0.103 0.46 6 0.6

0.12 0.1 0.14 0.103 0.31 4 0.48

0.07 0.12 0.06 0.103 0.25 6 0.72

0.09 0.09 0.15 0.101 0.32 6 0.68

0.1 0.1 0.1 0.104 0.32 7 0.57

0.14 0.05 0.09 0.102 0.39 4 0.71

0.08 0.08 0.08 0.104 0.47 4 0.63

0.11 0.13 0.09 0.103 0.44 3 0.59

0.1 0.14 0.11 0.103 0.63 5 0.38

0.12 0.08 0.1 0.104 0.72 3 0.28

0.12 0.08 0.07 0.099 0.69 7 0.65

0.1 0.09 0.1 0.098 0.43 7 0.58

0.12 0.08 0.1 0.102 0.4 7 0.54

0.12 0.14 0.09 0.102 0.73 6 0.57

0.13 0.14 0.11 0.098 0.4 5 0.3

0.08 0.1 0.14 0.098 0.35 6 0.68

0.07 0.07 0.11 0.096 0.57 5 0.56

0.07 0.07 0.1 0.103 0.64 6 0.27

0.12 0.11 0.08 0.097 0.33 3 0.32

0.1 0.06 0.06 0.097 0.35 7 0.57

0.08 0.05 0.08 0.099 0.7 6 0.71

0.11 0.11 0.1 0.103 0.55 3 0.39

0.1 0.08 0.07 0.102 0.47 5 0.4

0.09 0.12 0.14 0.098 0.48 5 0.55

0.06 0.12 0.15 0.1 0.74 3 0.35

0.07 0.11 0.12 0.096 0.31 4 0.6

0.07 0.13 0.12 0.098 0.62 7 0.56

0.15 0.14 0.14 0.095 0.31 4 0.28

0.1 0.15 0.06 0.102 0.56 5 0.34

0.11 0.11 0.09 0.099 0.32 7 0.56

0.13 0.13 0.1 0.095 0.56 4 0.36

0.06 0.14 0.11 0.096 0.62 3 0.32

0.12 0.06 0.11 0.1 0.32 7 0.5

0.12 0.07 0.11 0.095 0.28 3 0.27

0.1 0.13 0.07 0.104 0.72 4 0.4

0.1 0.11 0.13 0.102 0.6 5 0.55

0.08 0.12 0.14 0.096 0.69 6 0.29

0.12 0.1 0.08 0.101 0.34 5 0.37

0.09 0.06 0.08 0.098 0.48 3 0.5

0.13 0.13 0.06 0.1 0.37 5 0.47

0.07 0.09 0.15 0.096 0.53 5 0.26

0.1 0.13 0.14 0.099 0.32 7 0.3

0.11 0.09 0.09 0.101 0.63 7 0.59

0.13 0.07 0.11 0.097 0.53 7 0.28

0.13 0.14 0.15 0.096 0.39 7 0.73

0.06 0.08 0.05 0.099 0.31 7 0.36

0.06 0.13 0.11 0.096 0.41 5 0.72

0.06 0.14 0.06 0.101 0.7 6 0.55

0.15 0.08 0.12 0.101 0.71 5 0.37

0.11 0.07 0.14 0.1 0.68 7 0.39

0.06 0.07 0.12 0.095 0.29 3 0.68

0.08 0.08 0.09 0.099 0.5 4 0.45

0.05 0.1 0.1 0.099 0.48 3 0.41

0.13 0.05 0.09 0.099 0.5 4 0.59

0.11 0.12 0.06 0.096 0.55 7 0.64

0.07 0.14 0.13 0.096 0.57 7 0.29

0.11 0.13 0.06 0.096 0.48 3 0.51

0.05 0.13 0.07 0.098 0.45 6 0.56

0.12 0.13 0.1 0.102 0.41 3 0.41

0.11 0.11 0.07 0.103 0.36 6 0.36

0.13 0.08 0.1 0.097 0.61 5 0.35

0.09 0.1 0.06 0.099 0.38 3 0.41

0.07 0.15 0.14 0.104 0.5 4 0.71

0.11 0.06 0.09 0.096 0.42 3 0.28

0.08 0.09 0.1 0.102 0.35 3 0.42

0.15 0.08 0.15 0.096 0.45 6 0.31

0.06 0.08 0.09 0.096 0.52 3 0.74

0.14 0.1 0.1 0.097 0.41 7 0.52

0.11 0.09 0.07 0.101 0.31 6 0.44

0.07 0.14 0.13 0.097 0.49 7 0.71

0.12 0.1 0.05 0.1 0.42 5 0.66

0.07 0.12 0.12 0.105 0.29 7 0.48

0.1 0.14 0.1 0.102 0.5 5 0.7

0.06 0.14 0.1 0.097 0.42 3 0.52

0.11 0.13 0.09 0.102 0.25 5 0.3

0.1 0.11 0.08 0.1 0.37 6 0.33

0.07 0.06 0.07 0.098 0.49 7 0.58

0.05 0.11 0.08 0.104 0.49 4 0.54

0.12 0.1 0.11 0.098 0.34 5 0.47

0.09 0.06 0.14 0.098 0.49 6 0.32

0.06 0.07 0.08 0.101 0.37 5 0.66

0.11 0.07 0.11 0.096 0.28 7 0.69

0.11 0.13 0.1 0.1 0.73 6 0.39

0.13 0.14 0.07 0.098 0.54 7 0.49

0.09 0.08 0.11 0.096 0.44 6 0.55

0.08 0.13 0.12 0.101 0.46 6 0.74

0.14 0.12 0.07 0.1 0.33 5 0.52

0.05 0.11 0.1 0.104 0.48 6 0.74

0.06 0.13 0.1 0.097 0.31 7 0.28

0.11 0.13 0.05 0.101 0.49 4 0.69

0.06 0.14 0.07 0.102 0.7 6 0.43

0.09 0.08 0.08 0.096 0.57 7 0.49

0.11 0.12 0.06 0.103 0.68 5 0.67

0.08 0.1 0.11 0.098 0.67 3 0.38

0.13 0.13 0.08 0.102 0.29 5 0.32

0.13 0.07 0.08 0.103 0.48 7 0.64

0.13 0.07 0.1 0.098 0.61 3 0.72

0.11 0.09 0.12 0.097 0.55 6 0.4

0.08 0.05 0.08 0.1 0.45 4 0.75

0.12 0.12 0.14 0.099 0.66 4 0.46

0.13 0.1 0.05 0.105 0.27 6 0.31

0.12 0.11 0.08 0.1 0.75 5 0.62

0.08 0.12 0.11 0.098 0.53 6 0.74

0.1 0.13 0.07 0.098 0.53 3 0.75

0.1 0.14 0.07 0.105 0.69 4 0.39

0.09 0.12 0.11 0.095 0.45 6 0.74

0.08 0.07 0.07 0.104 0.63 7 0.68

0.12 0.11 0.06 0.101 0.25 5 0.72

0.14 0.14 0.14 0.103 0.51 7 0.36

0.15 0.07 0.14 0.101 0.29 6 0.53

0.07 0.13 0.07 0.097 0.51 6 0.31

0.14 0.14 0.12 0.1 0.48 6 0.53

0.14 0.14 0.14 0.105 0.43 7 0.58

0.05 0.13 0.15 0.098 0.27 6 0.26

0.08 0.14 0.11 0.099 0.26 6 0.4

0.13 0.14 0.14 0.099 0.67 5 0.27

0.09 0.15 0.1 0.102 0.32 4 0.32

0.11 0.05 0.13 0.101 0.56 4 0.39

0.07 0.12 0.14 0.098 0.48 4 0.31

0.07 0.13 0.07 0.104 0.74 4 0.45

0.11 0.12 0.11 0.102 0.62 3 0.63

0.12 0.11 0.08 0.1 0.59 6 0.56

0.07 0.14 0.1 0.1 0.34 3 0.66

0.1 0.12 0.11 0.1 0.43 6 0.38

0.08 0.06 0.12 0.096 0.33 5 0.68

0.13 0.11 0.13 0.101 0.55 4 0.69

0.13 0.14 0.08 0.101 0.31 5 0.41

0.07 0.14 0.15 0.096 0.59 4 0.42

0.09 0.11 0.11 0.1 0.28 7 0.52

0.06 0.08 0.06 0.1 0.29 4 0.29

0.05 0.07 0.07 0.097 0.6 3 0.37

0.08 0.13 0.06 0.1 0.53 5 0.35

0.09 0.08 0.13 0.097 0.58 3 0.34

0.11 0.13 0.07 0.098 0.33 7 0.47

0.06 0.13 0.07 0.097 0.55 6 0.29

0.13 0.09 0.05 0.095 0.53 7 0.42

0.06 0.07 0.09 0.098 0.61 6 0.4

0.13 0.07 0.08 0.104 0.33 7 0.49

0.12 0.06 0.08 0.101 0.72 4 0.29

0.06 0.09 0.11 0.095 0.75 5 0.42

0.14 0.05 0.11 0.104 0.46 6 0.68

0.12 0.12 0.07 0.098 0.72 4 0.4

0.14 0.13 0.07 0.104 0.59 6 0.59

0.06 0.07 0.14 0.099 0.36 3 0.57

0.1 0.1 0.09 0.101 0.26 4 0.74

0.06 0.08 0.14 0.1 0.68 4 0.69

0.12 0.08 0.12 0.1 0.67 7 0.37

0.05 0.06 0.14 0.098 0.72 3 0.67

0.13 0.15 0.06 0.097 0.4 3 0.59

0.06 0.06 0.14 0.101 0.52 4 0.3

0.06 0.05 0.14 0.099 0.34 3 0.73

0.07 0.11 0.15 0.103 0.47 3 0.47

0.11 0.11 0.1 0.098 0.49 6 0.55

0.11 0.15 0.07 0.095 0.7 5 0.69

0.09 0.12 0.08 0.101 0.28 5 0.56

0.05 0.08 0.08 0.098 0.31 5 0.3

0.07 0.06 0.12 0.1 0.41 5 0.46

0.11 0.07 0.1 0.104 0.36 4 0.71

0.07 0.05 0.07 0.099 0.43 3 0.39

0.13 0.06 0.12 0.1 0.73 7 0.57

0.08 0.09 0.1 0.096 0.54 4 0.47

0.12 0.15 0.15 0.097 0.59 4 0.46

0.1 0.1 0.08 0.096 0.4 6 0.57

0.09 0.15 0.13 0.105 0.33 6 0.51

0.13 0.14 0.07 0.104 0.59 6 0.33

0.08 0.13 0.09 0.097 0.36 4 0.66

0.08 0.11 0.14 0.096 0.7 7 0.69

0.08 0.13 0.12 0.099 0.33 3 0.32

0.13 0.12 0.07 0.098 0.44 6 0.26

0.1 0.1 0.14 0.099 0.35 3 0.72

0.08 0.1 0.14 0.099 0.4 6 0.57

0.06 0.13 0.08 0.101 0.74 6 0.64

0.05 0.06 0.08 0.096 0.58 4 0.4

0.1 0.13 0.15 0.101 0.63 7 0.56

0.13 0.07 0.15 0.097 0.57 3 0.39

0.14 0.12 0.09 0.097 0.42 7 0.73

0.1 0.14 0.14 0.105 0.63 7 0.44

0.07 0.13 0.09 0.101 0.62 3 0.4

0.14 0.1 0.1 0.104 0.52 4 0.53

0.07 0.11 0.14 0.102 0.41 4 0.34

0.12 0.09 0.12 0.104 0.55 4 0.72

0.05 0.11 0.1 0.096 0.5 7 0.63

0.1 0.11 0.13 0.1 0.44 7 0.66

0.06 0.1 0.11 0.101 0.43 4 0.55

0.13 0.14 0.11 0.097 0.5 6 0.33

0.08 0.07 0.11 0.102 0.35 7 0.5

0.14 0.14 0.1 0.097 0.54 7 0.42

0.06 0.1 0.06 0.096 0.69 3 0.38

0.11 0.09 0.12 0.102 0.38 7 0.59

0.13 0.06 0.09 0.099 0.29 6 0.59

0.13 0.08 0.07 0.1 0.43 6 0.35

0.06 0.07 0.06 0.103 0.38 4 0.3

0.14 0.11 0.08 0.096 0.37 5 0.39

0.1 0.08 0.08 0.098 0.32 4 0.63

0.15 0.12 0.14 0.102 0.72 7 0.65

0.09 0.1 0.07 0.097 0.36 5 0.66

0.14 0.07 0.08 0.103 0.52 3 0.48

0.06 0.12 0.1 0.104 0.32 7 0.31

0.06 0.12 0.12 0.098 0.31 7 0.45

0.11 0.14 0.07 0.104 0.26 3 0.53

0.05 0.11 0.12 0.105 0.49 6 0.46

0.15 0.09 0.09 0.101 0.62 4 0.41

0.12 0.08 0.11 0.103 0.42 7 0.69

0.08 0.06 0.14 0.105 0.41 7 0.56

0.11 0.14 0.1 0.096 0.44 5 0.45

0.11 0.14 0.05 0.097 0.74 7 0.35

0.14 0.14 0.1 0.097 0.4 3 0.32

0.06 0.08 0.11 0.105 0.31 3 0.59

0.13 0.15 0.06 0.104 0.29 5 0.32

0.07 0.13 0.08 0.098 0.42 3 0.42

0.09 0.05 0.07 0.1 0.41 5 0.33
