# Supplementary material for: Regulatory mechanisms link phenotypic plasticity to evolvability
Source: Sci Rep. 2016 Apr 18;6:24524. doi: 10.1038/srep24524 (PMC4834480; doi:10.1038/srep24524)
Supplement: Supplementary Data S2 [file srep24524-s3.doc]

# SIMULATIONS ASSOCIATED WITH TWENTY FITTEST GENOTYPES IN RN MODEL (USED FOR FIGURE S4 AND S5)

i = simulation number

N = number of individuals in the colony with given genotype

alpha_N = weighting factor for nutrients concentration

alpha_S = weighting factor for signal concentration

alpha_E = weighting factor for energy level

theta = activation threshold

i N alpha_N alpha_S alpha_E theta

1 2844 -0.051 0 0.002 -0.107

1 302 -0.039 0 0.002 -0.107

1 6 -0.051 0 0.002 0.004

1 4 -0.051 0 -0.05 -0.107

1 4 -0.051 0 -0.185 -0.107

1 2 -0.073 0 0.002 -0.107

1 2 -0.039 -0.029 0.002 -0.107

1 2 -0.051 0.121 0.002 -0.167

1 1 -0.051 0 0.002 -0.065

1 1 -0.051 0 0.208 -0.107

1 1 -0.051 0 0.047 -0.107

1 1 -0.051 0.109 0.002 -0.107

1 1 -0.149 0 0.002 -0.107

1 1 -0.039 0 0.002 -0.108

1 1 -0.051 0.052 0.002 -0.107

1 1 -0.051 -0.047 0.002 -0.107

1 1 -0.051 0 -0.024 -0.107

1 1 -0.051 0.142 0.002 -0.107

1 1 0.057 0 0.002 -0.107

1 1 -0.051 0 0.002 0.014

1 1 -0.051 -0.215 0.002 -0.107

2 2204 -0.249 0 0.008 -0.498

2 819 -0.164 0 0.008 -0.357

2 110 -0.249 0.122 0.008 -0.498

2 38 -0.333 0 0.008 -0.498

2 11 -0.249 0.077 0.008 -0.498

2 8 -0.249 -0.025 0.008 -0.498

2 5 -0.164 0 0.008 -0.265

2 3 -0.122 0 0.008 -0.498

2 2 -0.082 0 0.008 -0.357

2 2 -0.249 0 0.008 -0.451

2 2 -0.385 0 0.008 -0.498

2 2 -0.249 0 -0.034 -0.498

2 2 -0.249 0 -0.122 -0.498

2 2 -0.19 0 0.008 -0.498

2 2 -0.249 -0.137 0.008 -0.498

2 2 -0.249 0 -0.104 -0.498

2 2 -0.249 0 -0.024 -0.498

2 2 -0.249 0.239 0.008 -0.498

2 2 -0.164 0 0.021 -0.357

2 1 -0.116 0 0.008 -0.498

2 1 -0.112 0 0.008 -0.357

2 1 -0.164 0 0.008 -0.332

2 1 -0.249 0 0.008 -0.384

2 1 -0.147 0 0.008 -0.357

2 1 -0.249 0.169 0.008 -0.498

2 1 -0.249 0 0.008 -0.344

2 1 -0.164 0.114 0.008 -0.357

3 2144 -0.104 0 0.005 -0.196

3 688 -0.114 0 0.005 -0.208

3 139 -0.114 0 0.005 -0.304

3 122 -0.114 0 0.005 -0.254

3 182 -0.113 0 0.005 -0.196

3 26 -0.104 0 0.005 -0.251

3 5 -0.114 0 0.016 -0.254

3 4 -0.104 0 0.005 -0.178

3 4 -0.149 0 0.005 -0.208

3 4 -0.104 0 0.005 -0.286

3 4 -0.104 -0.002 0.005 -0.196

3 4 -0.104 -0.059 0.005 -0.196

3 3 -0.053 0 0.005 -0.196

3 2 -0.104 0 0.005 -0.309

3 1 -0.104 0 0.079 -0.196

3 1 -0.104 -0.005 0.005 -0.196

3 1 -0.104 0 0.005 -0.201

3 1 -0.114 0.077 0.005 -0.208

3 1 -0.104 0 0.005 0.037

3 1 -0.104 0 0.005 -0.27

3 1 -0.104 0 0.005 -0.233

4 3715 -0.003 0 0 -0.007

4 5 -0.003 0 -0.026 -0.007

4 4 -0.012 0 0 -0.007

4 2 -0.004 0 0 -0.007

4 2 -0.003 0 0 0.096

4 1 -0.003 0 0 -0.063

4 1 -0.003 0 0.028 -0.007

4 2 -0.003 -0.028 0 -0.007

4 1 -0.003 0 0.007 -0.007

4 2 -0.003 0 -0.05 -0.007

4 1 0.085 0 0 -0.007

4 1 0.163 0 0 -0.007

4 1 -0.003 0 0 0.028

4 1 -0.003 0 0 -0.057

4 1 -0.003 0 0.011 -0.007

4 1 -0.003 0 0 -0.103

4 1 -0.003 0 0.075 -0.007

4 1 -0.003 0 0 -0.008

4 1 -0.003 0.087 0 -0.007

4 1 -0.003 0 0.078 -0.007

4 1 0.083 0 0 -0.007

4 1 -0.034 0 0 -0.007

4 1 -0.003 0.083 0 -0.007

4 1 -0.003 0 0 0.027

4 1 -0.003 0 0 0.074

5 3108 -0.019 0 0.002 -0.028

5 45 -0.019 -0.067 0.002 -0.028

5 27 -0.019 -0.014 0.002 -0.028

5 11 -0.019 0 0.002 -0.053

5 4 -0.019 0 -0.098 -0.028

5 4 -0.117 0 0.002 -0.028

5 4 -0.081 0 0.002 -0.028

5 2 -0.019 0 -0.116 -0.028

5 2 -0.108 0 0.002 -0.028

5 1 -0.019 0 0.002 -0.149

5 1 -0.019 0 -0.039 -0.028

5 1 -0.019 0 0.002 -0.271

5 1 -0.019 0 0.002 0.002

5 1 -0.019 0 0.002 -0.094

5 1 0.113 0 0.002 -0.028

5 1 -0.019 0 0.002 0.089

5 1 -0.019 0 -0.143 -0.028

6 3713 -0.014 0 0 -0.031

6 34 -0.014 0 0 0.27

6 33 -0.014 0 -0.021 -0.031

6 34 -0.014 0 -0.05 -0.031

6 8 -0.014 0.043 0 -0.031

6 7 -0.047 0 -0.05 -0.031

6 4 -0.064 0 0 -0.031

6 2 -0.014 0 0 0.136

6 2 -0.014 -0.046 0 -0.031

6 2 -0.014 -0.104 0 -0.031

6 2 -0.014 -0.207 0 -0.031

6 2 -0.073 0 0 -0.031

6 2 -0.014 -0.078 0 -0.031

6 2 -0.014 0 -0.031 -0.031

6 1 -0.014 0 0.01 -0.031

6 1 0.109 0 0 -0.031

6 1 0.14 0 0 -0.031

6 1 -0.014 0 0 -0.139

6 1 -0.014 -0.116 0 -0.031

6 1 -0.014 -0.068 0 -0.031

6 1 -0.014 0 0.071 -0.031

6 1 -0.014 0 0 -0.086

6 1 -0.025 0 -0.05 -0.031

6 1 -0.014 0 -0.134 -0.031

6 1 0.013 0 0 -0.031

6 1 -0.014 0.044 0 -0.031

6 1 -0.014 0 -0.292 -0.031

6 1 0.022 0 0 -0.031

6 1 -0.014 0 -0.063 0.108

6 1 -0.089 0 0 -0.031

7 3290 -0.045 0 0 -0.109

7 59 -0.045 0 0 -0.12

7 4 -0.169 0 0 -0.109

7 4 -0.045 0 -0.084 -0.109

7 4 -0.045 -0.115 0 -0.109

7 2 -0.045 0 -0.074 -0.109

7 2 -0.045 0 -0.128 -0.109

7 1 0.072 0 0 -0.109

7 1 -0.045 0 0.078 -0.109

7 1 -0.045 0 0 -0.318

7 1 -0.045 0 0.048 -0.109

7 1 -0.045 0 0.233 -0.109

7 1 -0.045 0.26 0 -0.109

7 1 0.061 0 0 -0.109

7 1 0.096 0 0 -0.109

7 1 -0.103 0 0 -0.109

7 1 -0.045 0.044 0 -0.109

7 1 -0.045 0 0 -0.261

7 1 -0.045 0 0 -0.311

7 1 -0.045 0 0 -0.05

7 1 -0.045 0 -0.01 -0.109

8 2656 -0.063 0.007 0 -0.116

8 623 -0.063 0.052 0 -0.116

8 11 -0.063 0.007 0 -0.146

8 6 -0.063 0.007 -0.154 -0.116

8 5 -0.063 -0.151 0 -0.116

8 3 -0.063 0.007 0 -0.153

8 2 -0.063 -0.033 0 -0.116

8 2 -0.063 0.064 0 -0.116

8 2 -0.063 0.007 -0.074 -0.116

8 2 -0.063 0.052 0 -0.251

8 2 -0.063 0.062 0 -0.116

8 1 -0.063 0.007 0.06 -0.116

8 1 -0.063 0.007 0.051 -0.116

8 1 -0.063 0.007 0.166 -0.116

8 1 -0.063 0.209 0 -0.116

8 1 -0.063 0.007 0 -0.171

8 1 -0.063 0.052 0 -0.101

8 2 -0.063 0.007 0.083 -0.116

8 1 -0.029 0.052 0 -0.116

8 1 -0.034 0.007 0 -0.116

8 1 -0.063 0.145 0 -0.116

8 1 -0.01 0.007 0 -0.116

8 1 -0.063 0.007 0 -0.208

8 1 -0.063 0.018 0 -0.116

8 1 -0.209 0.007 0 -0.116

8 1 -0.063 -0.065 0 -0.116

8 1 -0.063 0.054 0 -0.116

8 1 -0.063 -0.026 0 -0.116

9 3599 -0.048 -0.011 0 -0.145

9 43 -0.209 -0.011 0 -0.145

9 26 -0.048 -0.011 0 -0.063

9 24 -0.048 -0.011 0 -0.015

9 13 -0.048 -0.011 0 -0.116

9 7 -0.048 -0.011 0 -0.188

9 2 -0.124 -0.011 0 -0.145

9 1 -0.048 -0.011 0 -0.229

9 1 -0.048 -0.011 0 -0.219

9 1 -0.048 -0.011 0.036 -0.145

9 1 -0.048 -0.011 0.074 -0.145

9 1 -0.048 -0.011 0.042 -0.145

9 1 -0.048 -0.011 0.251 -0.145

9 1 -0.048 -0.011 0.174 -0.145

9 1 -0.048 -0.011 0.159 -0.145

9 1 -0.048 -0.011 0 -0.12

9 1 -0.279 -0.011 0 -0.145

9 1 -0.048 -0.011 0 -0.17

9 1 -0.048 0.123 0 -0.145

9 1 0.125 -0.011 0 -0.145

10 3047 -0.043 0.005 0 -0.093

10 165 -0.032 0.005 0 -0.093

10 22 -0.043 0.005 0 -0.099

10 14 -0.155 0.005 0 -0.093

10 4 -0.043 0.005 0 -0.174

10 4 -0.043 0.031 0 -0.093

10 4 -0.043 0.005 -0.094 -0.093

10 4 -0.164 0.005 0 -0.093

10 4 -0.183 0.005 0 -0.093

10 2 -0.043 -0.093 0 -0.093

10 2 -0.043 0.005 0 -0.102

10 2 -0.095 0.005 0 -0.093

10 2 -0.073 0.005 0 -0.093

10 2 -0.097 0.005 0 -0.093

10 1 -0.043 0.005 0.101 -0.093

10 1 -0.043 0.005 0.242 -0.093

10 1 -0.043 0.005 0 -0.167

10 1 0.058 0.005 0 -0.093

10 1 -0.043 0.005 0 0.104

10 1 -0.043 -0.078 0 -0.093

10 1 0.074 0.005 0 -0.093

11 3141 -0.059 0 0 -0.138

11 86 -0.059 0.043 0 -0.138

11 76 -0.059 0 0 -0.162

11 40 -0.059 0.107 0 -0.138

11 6 -0.059 -0.03 0 -0.138

11 4 -0.059 0 0 -0.059

11 4 -0.059 0.043 0 -0.171

11 5 -0.059 -0.062 0 -0.138

11 4 -0.059 0.011 0 -0.138

11 3 -0.059 0 0 -0.352

11 2 -0.076 0 0 -0.138

11 2 -0.059 0 -0.061 -0.138

11 2 -0.059 0 -0.054 -0.138

11 2 -0.145 0 0 -0.138

11 2 -0.059 0.093 0 -0.138

11 1 -0.059 0 0 -0.164

11 1 -0.059 0 0 -0.255

11 1 -0.059 0.065 0 -0.138

11 1 -0.059 0.064 0 -0.138

11 1 -0.055 0 0 -0.138

11 1 -0.059 0.152 0 -0.138

11 1 -0.059 0 -0.124 -0.138

11 1 -0.059 -0.062 0.029 -0.138

11 1 -0.059 0 -0.081 -0.138

11 1 -0.059 0 -0.011 -0.138

12 1759 -0.071 0 0 -0.195

12 864 -0.084 0 0 -0.195

12 281 -0.084 0 0 -0.332

12 93 -0.096 0 0 -0.195

12 39 -0.071 0 0 -0.169

12 10 -0.071 -0.03 0 -0.195

12 9 -0.072 0 0 -0.195

12 4 -0.071 -0.049 0 -0.169

12 4 -0.084 0 0 -0.316

12 2 -0.103 0 0 -0.195

12 2 -0.071 0 0 -0.14

12 2 -0.071 0 0 -0.067

12 2 -0.071 0 -0.04 -0.169

12 1 -0.071 0.116 0 -0.195

12 1 0.051 0 0 -0.195

12 1 -0.084 0 0.06 -0.195

12 1 -0.071 0.107 0 -0.195

12 1 -0.135 0 0 -0.195

12 1 -0.03 0 0 -0.195

12 1 -0.071 0 0 -0.131

12 1 -0.093 0 0 -0.195

12 1 0.107 0 0 -0.195

13 3301 -0.02 0 0 -0.049

13 23 -0.02 0 -0.108 -0.049

13 6 -0.02 -0.005 0 -0.049

13 4 -0.02 0 -0.099 -0.049

13 4 -0.044 0 0 -0.049

13 3 -0.02 0 -0.201 -0.049

13 2 -0.07 0 0 -0.049

13 2 -0.263 0 0 -0.049

13 1 -0.02 0 0 -0.206

13 1 0.087 0 0 -0.049

13 1 -0.02 0 0 -0.137

13 1 -0.02 0 0 -0.063

13 1 -0.02 0 0.118 -0.049

13 1 -0.02 -0.055 0 -0.049

13 1 -0.02 0 0 0.059

13 1 -0.02 0 0 0

13 1 -0.02 0 0.087 -0.049

13 1 -0.02 0 -0.102 -0.049

13 1 -0.02 0 0 -0.046

13 1 -0.02 0 0 0.01

13 1 -0.02 0 0 -0.14

14 2757 -0.053 0 0 -0.144

14 7 -0.053 0.073 0 -0.144

14 9 -0.087 0 0 -0.144

14 4 -0.053 0 0 -0.107

14 2 -0.053 0 -0.052 -0.144

14 2 -0.053 -0.077 0 -0.144

14 2 -0.053 0.044 0 -0.144

14 2 -0.053 0.035 0 -0.144

14 1 -0.004 0.073 0 -0.144

14 1 -0.019 0 0 -0.144

14 1 0.142 0 0 -0.144

14 1 -0.053 0 0.125 -0.144

14 1 -0.053 0.093 0 -0.144

14 1 -0.053 0 0.024 -0.144

14 1 -0.053 0 0.103 -0.144

14 1 -0.053 -0.042 0 -0.144

14 1 -0.053 0 0 -0.114

14 1 -0.092 0 0 -0.144

14 1 -0.053 0 0.042 -0.144

14 1 -0.047 0 0 -0.144

14 1 -0.053 0 -0.096 -0.144

14 1 -0.053 0.08 0 -0.144

14 1 -0.053 -0.093 0 -0.144

15 3768 -0.027 0 0 -0.06

15 19 -0.027 0 0.003 -0.06

15 9 -0.027 0 0 -0.065

15 6 -0.027 -0.022 0 -0.06

15 5 -0.027 0 0 -0.152

15 5 -0.027 0 -0.019 -0.06

15 6 -0.027 0 0 -0.035

15 4 -0.096 0 0 -0.06

15 2 -0.027 0 0 -0.067

15 2 -0.027 -0.236 0 -0.06

15 2 -0.027 0.009 0 -0.06

15 2 -0.027 -0.246 0 -0.06

15 2 -0.181 0 0 -0.06

15 1 0.148 0 0 -0.06

15 1 -0.027 0 0.077 -0.06

15 1 -0.027 0 0 -0.143

15 1 0.021 0 0 -0.06

15 1 -0.027 0.091 0 -0.06

15 1 -0.027 0 0.075 -0.06

15 1 -0.027 0 0.048 -0.06

15 1 0.229 0 0 -0.06

15 1 0.079 0 0 -0.06

15 1 -0.209 0 0 -0.06

15 1 -0.027 -0.05 0 -0.06

15 1 -0.093 0 0 -0.06

15 1 0.045 0 0 -0.06

15 1 -0.027 0 -0.022 -0.06

15 1 -0.027 0 0 -0.083

16 2696 -0.009 0 0 -0.025

16 50 -0.009 0 -0.089 -0.025

16 6 -0.009 0 0 -0.063

16 5 -0.009 0 0 0.016

16 4 -0.009 0 0 0.004

16 4 -0.158 0 0 -0.025

16 3 -0.009 0.031 0 -0.025

16 2 -0.009 -0.036 0 -0.025

16 2 -0.009 0 0 0.188

16 2 -0.009 0 0 0.011

16 1 -0.009 0 0.256 -0.025

16 1 -0.009 0.153 0 -0.025

16 1 -0.009 0 0.098 -0.025

16 1 0.126 0 0 -0.025

16 1 -0.009 0 0.066 -0.025

16 1 0.066 0 0 -0.025

16 1 -0.009 -0.077 0 -0.025

16 1 -0.009 0 0.017 -0.025

16 1 -0.009 0 -0.091 -0.025

16 1 0.145 0 0 -0.025

16 1 0.019 0 -0.089 -0.025

16 1 -0.009 0 0 -0.011

16 1 0.019 0 0 -0.025

16 1 -0.009 0 -0.005 -0.025

16 1 -0.012 0 -0.089 -0.025

16 1 -0.148 0 0 -0.025

16 1 -0.009 -0.022 0 -0.025

16 1 -0.009 0 0 -0.083

17 3016 -0.042 0 0 -0.106

17 70 -0.042 0.041 0 -0.106

17 6 -0.152 0 0 -0.106

17 4 -0.065 0 0 -0.106

17 4 -0.042 0 0 -0.055

17 6 -0.017 0 0 -0.106

17 2 -0.042 0 -0.095 -0.106

17 2 -0.042 0.041 0 -0.025

17 2 -0.042 0 0.061 -0.106

17 1 -0.04 0 0 -0.106

17 1 -0.042 0 0 -0.2

17 1 -0.042 0 0.214 -0.106

17 1 0.048 0 0 -0.106

17 1 -0.042 0.182 0 -0.106

17 1 -0.173 0 0 -0.106

17 1 -0.042 -0.019 0 -0.106

17 1 -0.042 0 0.079 -0.106

17 1 -0.042 0 0 -0.1

17 1 -0.042 -0.03 0 -0.106

17 1 -0.042 -0.013 0 -0.106

18 1385 -0.072 0 0 -0.15

18 680 -0.051 0 0 -0.15

18 591 -0.051 0 0 -0.168

18 350 -0.072 0 0 -0.163

18 43 -0.051 -0.007 0 -0.15

18 6 -0.072 0 0 -0.003

18 2 -0.072 0 0 -0.235

18 2 -0.051 -0.031 0 -0.15

18 2 -0.072 -0.224 0 -0.15

18 2 -0.051 -0.106 0 -0.15

18 1 -0.072 0.085 0 -0.15

18 1 -0.051 0.071 0 -0.15

18 1 -0.072 0.048 0 -0.15

18 1 -0.051 0 0 -0.165

18 1 -0.051 0 0.107 -0.15

18 2 -0.061 0 0 -0.168

18 1 -0.072 0 0 -0.181

18 1 -0.051 0.182 0 -0.15

18 1 -0.051 -0.007 0 -0.283

19 1264 -0.09 0 0 -0.21

19 793 -0.09 0 0 -0.214

19 949 -0.09 0 0 -0.185

19 378 -0.09 0 0 -0.29

19 59 -0.09 0 -0.036 -0.185

19 87 -0.094 0 0 -0.214

19 7 -0.09 0 -0.153 -0.21

19 4 -0.09 -0.094 0 -0.21

19 2 -0.1 0 0 -0.29

19 4 -0.028 0 0 -0.185

19 2 -0.09 0 0 -0.201

19 3 -0.046 0 0 -0.21

19 1 -0.017 0 0 -0.185

19 1 -0.09 0 0.053 -0.214

19 1 -0.009 0 0 -0.185

19 1 -0.09 0.032 0 -0.185

19 1 -0.09 0 0.102 -0.185

19 1 0.093 0 0 -0.21

19 1 -0.09 0 -0.162 -0.185

19 1 -0.09 0 0 -0.136

19 1 -0.072 0 0 -0.21

19 1 -0.09 0 0 -0.242

20 3300 -0.019 0 0 -0.046

20 192 -0.019 0 -0.031 -0.046

20 12 -0.019 0 0 -0.099

20 4 -0.019 0 -0.09 -0.046

20 4 -0.019 0 0 -0.004

20 2 -0.241 0 0 -0.046

20 1 -0.019 0 0.015 -0.046

20 1 -0.019 0 0 -0.16

20 1 -0.019 0 0 -0.082

20 1 0.118 0 0 -0.046

20 1 -0.019 0 0.143 -0.046

20 1 -0.019 -0.122 0 -0.046

20 1 0.056 0 0 -0.046

20 1 0.111 0 -0.031 -0.046

20 1 -0.019 0 0.135 -0.046

20 1 0.052 0 0 -0.046

20 1 -0.019 0 -0.031 -0.01

20 1 -0.019 0.084 0 -0.046

20 1 -0.019 0 -0.068 -0.046

20 1 -0.126 0 0 -0.046

20 1 0.01 0 0 -0.046
