# Supplementary material for: Regulatory mechanisms link phenotypic plasticity to evolvability
Source: Sci Rep. 2016 Apr 18;6:24524. doi: 10.1038/srep24524 (PMC4834480; doi:10.1038/srep24524)
Supplement: Supplementary Data S3 [file srep24524-s4.doc]

# SIMULATIONS ASSOCIATED WITH TWENTY FITTEST GENOTYPES IN GRN MODEL (USED FOR FIGURE S10 AND S11)

i = simulation number

N = number of individuals in the colony with given genotype

w_N_G1 = connection weight between nutrient concentration and gene 1 in regulatory layer

w_N_G2 = connection weight between nutrient concentration and gene 2 in regulatory layer

w_N_G3 = connection weight between nutrient concentration and gene 3 in regulatory layer

w_S_G1 = connection weight between signal concentration and gene 1 in regulatory layer

w_S_G2 = connection weight between signal concentration and gene 2 in regulatory layer

w_S_G3 = connection weight between signal concentration and gene 3 in regulatory layer

w_E_G1 = connection weight between energy level and gene 1 in regulatory layer

w_E_G2 = connection weight between energy level and gene 2 in regulatory layer

w_E_G3 = connection weight between energy level and gene 3 in regulatory layer

w_G1_G1 = self-regulatory connection weight of gene 1 in regulatory layer

w_G1_G2 = connection weight between gene 1 and gene 2 in regulatory layer

w_G1_G3 = connection weight between gene 1 and gene 3 in regulatory layer

w_G2_G1 = connection weight between gene 2 and gene 1 in regulatory layer

w_G2_G2 = self-regulatory connection weight of gene 2 in regulatory layer

w_G2_G3 = connection weight between gene 2 and gene 3 in regulatory layer

w_G3_G1 = connection weight between gene 3 and gene 1 in regulatory layer

w_G3_G2 = connection weight between gene 3 and gene 2 in regulatory layer

w_G3_G3 = self-regulatory connection weight of gene 3 in regulatory layer

w_G1_O = connection weight between gene 1 in regulatory layer and gene in output layer

w_G2_O = connection weight between gene 2 in regulatory layer and gene in output layer

w_G3_O = connection weight between gene 3 in regulatory layer and gene in output layer

T_G1 = activation threshold of gene 1 in regulatory layer

T_G2 = activation threshold of gene 2 in regulatory layer

T_G3 = activation threshold of gene 3 in regulatory layer

T_O = activation threshold of gene in output layer

i N w_N_G1 w_N_G2 w_N_G3 w_S_G1 w_S_G2 w_S_G3 w_E_G1 w_E_G2 w_E_G3 w_G1_G1 w_G1_G2 w_G1_G3 w_G2_G1 w_G2_G2 w_G2_G3 w_G3_G1 w_G3_G2 w_G3_G3 w_G1_O w_G2_O w_G3_O T_G1 T_G2 T_G3 T_O

1 847 0.043 -0.147 0 0 0.074 0 0 -0.05 0.079 -0.09 0.264 -0.105 0.027 0.259 0 0.076 0 0.056 -0.087 0.08 0.055 0 -0.073 0.02 0.042

1 656 0.043 -0.098 0 0 0.074 0 0 -0.05 0.079 -0.09 0.194 -0.105 0.027 0.259 0 0.076 0 0.056 -0.087 0.08 0.055 0 -0.073 0.02 0.042

1 652 0.043 -0.098 0 0 0.074 0 0 -0.05 0.079 -0.09 0.194 -0.105 0.027 0.259 0 0.076 0 0.056 -0.087 0.08 0.055 0.026 -0.073 0.02 0.042

1 424 0.043 -0.147 0 0 0.074 0 0 -0.05 0.079 -0.09 0.264 -0.105 0.027 0.259 0 0.076 0 0.056 -0.087 0.08 0.055 0 -0.073 0.02 -0.005

1 530 0.043 -0.098 0 0 0.074 0 0.208 -0.05 0.079 -0.09 0.194 -0.105 0.027 0.259 0 0.076 0 0.056 -0.087 0.08 0.055 0.026 -0.073 0.02 0.042

1 157 0.043 -0.147 0 0 0.074 0 0 -0.05 0.079 -0.09 0.194 -0.105 0.027 0.259 0 0.076 0 0.056 -0.087 0.08 0.055 0 -0.073 0.02 0.042

1 100 0.043 -0.147 0 0 0.074 0 0 -0.05 0.079 -0.09 0.264 -0.105 0.027 0.259 -0.053 0.076 0 0.056 -0.087 0.08 0.055 0 -0.073 0.02 0.042

1 98 0.043 -0.098 0 0 0.074 0 0.208 -0.05 0.079 -0.09 0.194 -0.105 0.027 0.259 -0.14 0.076 0 0.056 -0.087 0.08 0.055 0.026 -0.073 0.02 0.042

1 86 0.043 -0.147 0 0 0.074 0 0 -0.05 0.053 -0.09 0.264 -0.105 0.027 0.259 0 0.076 0 0.056 -0.055 0.08 0.055 0 -0.073 0.02 0.042

1 85 0.043 -0.147 0 0 0.074 0 0 -0.05 0.079 -0.09 0.264 -0.105 0.027 0.259 0 0.076 0 0.056 -0.055 0.08 0.055 0 -0.073 0.02 0.042

1 67 0.043 -0.098 0 0 0.074 0 0 -0.05 0.079 -0.09 0.194 -0.105 0.027 0.259 0 0.076 0 0.056 -0.087 0.08 0.055 0 -0.309 0.02 0.042

1 46 0.043 -0.098 0 0 0.074 0 0 -0.05 0.079 -0.09 0.194 -0.105 0.027 0.259 0 0.076 0.145 0.056 -0.087 0.08 0.055 0 -0.073 0.02 0.042

1 21 0.043 -0.098 0 0 0.074 0 0 -0.05 0.079 -0.09 0.194 -0.105 0.027 0.259 0 0.076 0 0.056 -0.087 0.08 0.055 0 -0.249 0.02 0.042

1 15 0.043 -0.098 0 0 0.074 0 0 -0.144 0.079 -0.09 0.194 -0.105 0.027 0.259 0 0.076 0 0.056 -0.087 0.08 0.055 0 -0.073 0.02 0.042

1 14 0.043 -0.098 0 0 0.074 0 0.208 -0.05 0.079 -0.09 0.194 -0.105 0.027 0.259 0 0.076 0.023 0.056 -0.087 0.08 0.055 0.026 -0.073 0.02 0.042

1 14 0.043 -0.147 0 0 0.074 0 0 -0.05 0.079 -0.09 0.264 -0.105 0.027 0.259 0 0.076 0 0.056 -0.087 0.102 0.055 0 -0.073 0.02 -0.005

1 10 0.07 -0.147 0 0 0.074 0 0 0 0.079 0 0.194 -0.105 0.091 0.259 0 0 0 0.056 -0.077 0.08 0.058 0 0 0 0.042

1 10 0.043 -0.098 0 0 0.074 0 0 -0.05 -0.154 -0.09 0.194 -0.105 0.027 0.259 0 0.076 0 0.056 -0.087 0.08 0.055 0.026 -0.073 0.02 0.042

1 9 0.043 -0.098 0 0 0.074 0 0.208 -0.05 0.079 -0.09 0.194 -0.105 0.027 0.259 -0.14 0.076 0 0.056 -0.087 0.08 0.055 0.026 -0.073 0.02 -0.005

1 9 0.043 -0.098 0 0 0.074 0 0 -0.05 0.079 -0.09 0.194 -0.105 0.027 0.259 0 0.076 0 0.056 -0.087 0.08 0.055 0.026 -0.073 -0.231 0.042

1 8 0.043 -0.147 0 0 -0.134 0 0 -0.05 0.079 -0.09 0.264 -0.105 0.027 0.259 0 0.076 0 0.056 -0.087 0.08 0.055 0 -0.073 0.02 -0.005

1 7 0.043 -0.087 0 0 0.074 0 0 -0.05 0.079 -0.064 0.264 -0.105 0.027 0.259 0 0.076 0 0.056 -0.087 0.08 0.055 0 -0.073 0.02 0.042

1 7 0.043 -0.098 0 0 0.074 0 0 -0.05 0.079 -0.09 0.194 -0.132 0.027 0.259 0 0.076 0 0.056 -0.087 0.08 0.055 0.026 -0.073 0.02 0.042

1 7 0.043 -0.147 0 0 0.074 0 0 -0.05 0.079 -0.09 0.264 -0.105 0.027 0.259 0 0.076 0.049 0.056 -0.087 0.08 0.055 0 -0.073 0.02 0.042

1 6 0.043 -0.098 0 0 0.074 0 0.208 -0.05 0.079 -0.09 0.194 -0.105 0.027 0.259 0 0.076 0 0.056 -0.087 0.08 0.055 0.026 0.017 0.02 0.042

1 6 0.043 -0.147 0 0 0.074 0 0 -0.05 0.079 -0.09 0.245 -0.105 0.027 0.259 0 0.076 0 0.056 -0.087 0.08 0.055 0 -0.073 0.02 0.042

1 6 0.043 -0.098 0 0 0.074 0 0 -0.05 0.079 -0.09 0.194 -0.105 0.027 0.259 0 0.076 0.021 0.056 -0.087 0.08 0.055 0.026 -0.073 0.02 0.042

1 5 0.043 -0.147 0 0 0.074 0 0 -0.05 0.079 -0.09 0.194 -0.183 0.027 0.259 0 0.076 0 0.056 -0.087 0.08 0.055 0 -0.073 0.02 0.042

1 5 0.043 -0.098 0 0 0.074 0 0.208 -0.05 0.079 -0.09 0.194 -0.105 0.027 0.346 0 0.076 0 0.056 -0.087 0.08 0.055 0.026 -0.073 0.02 0.042

1 5 0.043 -0.147 0 0 0.074 0 0 -0.15 0.079 -0.09 0.264 -0.105 0.027 0.259 0 0.076 0 0.056 -0.087 0.08 0.055 0 -0.073 0.02 0.042

1 4 0.043 -0.098 0 0 0.074 0 0 -0.05 0.079 -0.09 0.194 -0.105 -0.059 0.259 0 0.076 0 0.056 -0.087 0.08 0.055 0.026 -0.073 0.02 0.042

1 4 0.043 -0.098 0 0 0.074 0 0 -0.05 0.079 -0.09 0.194 -0.105 0.027 0.259 0 0.076 0 0.071 -0.087 0.08 0.055 0.026 -0.073 0.02 0.042

1 4 0.043 -0.147 0 0 0.074 0 0 -0.05 0.079 -0.09 0.194 -0.105 0.027 0.259 0 0.076 0 0.157 -0.087 0.08 0.055 0 -0.073 0.02 0.042

1 4 0.043 -0.147 0 0 0.074 0 0 -0.05 0.079 -0.09 0.264 -0.105 0.027 0.259 0 0.076 0 0.056 -0.122 0.102 0.055 0 -0.073 0.02 -0.005

1 4 0.043 -0.098 0 0 0.074 0 0 -0.05 0.079 -0.09 0.194 -0.105 0.027 0.259 -0.085 0.076 0 0.056 -0.087 0.08 0.055 0 -0.073 0.02 0.042

1 4 0.043 -0.098 0 0 0.074 0 0 -0.05 0.079 -0.09 0.194 -0.105 0.081 0.259 0 0.076 0 0.056 -0.087 0.08 0.055 0.026 -0.073 0.02 0.042

1 4 0.043 -0.098 0 0 -0.092 -0.193 0 -0.05 0.079 -0.09 0.194 -0.105 0.027 0.259 0 0.076 0 0.056 -0.087 0.08 0.055 0 -0.073 0.02 0.042

1 4 0.043 -0.098 0 0 0.074 0 0.187 -0.05 0.079 -0.09 0.194 -0.105 0.027 0.259 0 0.076 0 0.056 -0.087 0.08 0.055 0.026 -0.073 0.02 0.042

1 4 0.043 -0.147 0 0 0.074 0 0 -0.05 0.079 -0.09 0.264 -0.105 0.027 0.259 0.147 0.076 0 0.056 -0.087 0.08 0.055 0 -0.073 0.02 0.042

1 4 0.043 -0.098 0 0 0.074 0.088 0 -0.05 0.079 -0.09 0.194 -0.105 0.027 0.259 0 0.076 0 0.056 -0.087 0.08 0.055 0 -0.073 0.02 0.042

1 4 0.043 -0.098 0 0 0.074 0 0 -0.05 0.079 -0.09 0.194 -0.105 0.027 0.259 0 0.076 0 0.056 -0.087 0.14 0.055 0 -0.073 0.02 0.042

1 4 0.043 -0.147 0 0 0.074 0 0.083 -0.05 0.079 -0.09 0.264 -0.105 0.027 0.259 0 0.076 0 0.056 -0.087 0.08 0.055 0 -0.073 0.02 0.042

1 4 0.043 -0.147 0 0.133 0.074 0 0 -0.106 0.079 -0.09 0.264 -0.105 0.027 0.259 0 0.076 0 0.056 -0.087 0.08 0.055 0 -0.073 0.02 -0.005

1 4 0.043 -0.147 0 0 0.074 0 0 -0.05 0.079 -0.09 0.264 -0.105 0.027 0.259 0 0.076 0 0.056 -0.087 0.08 0.007 0 -0.073 0.02 0.042

1 4 0.043 -0.147 0 0 0.074 0 0 -0.05 0.053 -0.09 0.264 -0.105 0.027 0.259 0 0.076 0 0.056 -0.13 0.08 0.055 0 -0.073 0.02 0.042

1 4 0.043 -0.098 0 0 0.074 0 0 -0.05 0.079 -0.09 0.194 -0.105 0.076 0.259 0 0.076 0 0.056 -0.087 0.08 0.055 0 -0.073 0.02 0.042

1 4 0.043 -0.098 0 0 0.074 0 0.06 -0.05 0.079 -0.09 0.194 -0.105 0.027 0.259 0 0.076 0 0.056 -0.087 0.08 0.055 0 -0.073 0.02 0.042

1 3 0.043 -0.098 0 0 0.074 0 0.208 -0.05 0.079 -0.09 0.194 -0.105 0.027 0.259 0 0.076 0 0.056 -0.087 0.006 0.055 0.026 -0.073 0.02 0.042

1 3 0.043 -0.147 0 0 0.074 0 0 -0.05 0.079 -0.09 0.194 -0.091 0.027 0.259 0 0.076 0 0.056 -0.087 0.08 0.055 0 -0.073 0.02 0.042

1 3 0.043 -0.098 0 0 0.074 -0.082 0 -0.05 0.079 -0.09 0.194 -0.105 0.027 0.259 0 0.076 0 0.056 -0.087 0.08 0.055 0 -0.249 0.02 0.042

1 3 0.043 -0.147 0 0 0.074 0 0 -0.05 0.079 -0.09 0.264 -0.105 0.027 0.259 0 0.076 0 0.056 -0.087 0.08 0.055 0.024 -0.073 0.02 0.042

1 3 0.043 -0.147 0 0 0.074 0 0 -0.05 0.079 -0.09 0.228 -0.105 0.027 0.259 0 0.076 0 0.056 -0.087 0.08 0.055 0 -0.073 0.02 0.042

1 2 0.043 -0.147 0 0 0.074 0 0 -0.05 0.199 -0.09 0.264 -0.105 0.027 0.259 0 0.076 0 0.056 -0.087 0.08 0.055 0 -0.073 0.02 0.042

1 2 0.043 -0.147 0 0 0.074 0 0 -0.05 0.079 -0.09 0.264 -0.105 0.027 0.259 0 0.076 0 0.056 -0.087 0.094 0.055 0 -0.073 0.02 -0.005

1 2 0.043 -0.098 0 0 0.074 0 0 -0.05 0.079 -0.09 0.194 -0.105 0.027 0.259 0 0.076 0 0.056 -0.087 0.08 0.055 0 -0.073 0.02 0.12

1 2 0.043 -0.147 0 0 0.074 0 0 -0.05 0.079 -0.09 0.264 -0.105 0.027 0.259 0 0.076 0 0.056 -0.055 0.08 0.055 0 -0.073 0.02 0.12

1 2 0.043 -0.098 0 0 0.074 0 0.208 -0.198 0.079 -0.09 0.194 -0.105 0.027 0.259 -0.14 0.076 0 0.056 -0.087 0.08 0.055 0.026 -0.073 0.02 0.042

1 2 0.043 -0.147 0 0 0.009 0 0 -0.05 0.079 -0.09 0.264 -0.105 0.027 0.259 0 0.076 0 0.056 -0.087 0.08 0.055 0 -0.073 0.02 0.042

1 2 0.043 -0.147 0 0 0.074 0 0.041 -0.05 0.079 -0.09 0.264 -0.105 0.027 0.259 0 0.076 0 0.056 -0.087 0.08 0.055 0 -0.073 0.02 0.042

1 2 0.043 -0.147 0 0 0.074 0 0 -0.05 0.079 -0.09 0.088 -0.105 0.027 0.259 -0.053 0.076 0 0.056 -0.087 0.08 0.055 0 -0.073 0.02 0.042

1 2 0.043 -0.098 0 0 0.074 0 0.208 -0.05 0.079 -0.09 0.194 -0.262 0.027 0.259 0 0.076 0 0.056 -0.087 0.08 0.055 0.026 -0.073 0.02 0.042

1 2 0.043 -0.098 0 0 0.074 0 0 -0.05 0.079 -0.09 0.194 -0.105 0.027 0.259 0 0.076 0 0.056 -0.087 0.024 0.055 0.026 -0.073 0.02 0.042

1 2 0.043 -0.147 0 0 0.074 0 0 -0.05 0.079 -0.09 0.194 -0.105 0.027 0.259 0 0.076 0 0.056 -0.255 0.08 0.055 0 -0.073 0.02 0.042

1 2 0.043 -0.098 0 0 0.074 0 0.208 -0.05 0.079 -0.09 0.194 -0.105 0.027 0.259 0 0.076 0 0.056 -0.087 0.08 -0.048 0.026 -0.073 0.02 0.042

1 2 0.043 -0.147 0 0 0.074 0 0 -0.05 0.079 0.089 0.264 -0.105 0.027 0.259 0 0.076 0 0.056 -0.055 0.08 0.055 0 -0.073 0.02 0.042

1 2 0.043 -0.098 0 0 0.074 0 0.208 -0.05 0.079 -0.09 0.194 -0.105 0.027 0.346 0 0.2 0 0.056 -0.087 0.08 0.055 0.026 -0.073 0.02 0.042

1 2 0.043 -0.098 0 0 0.074 0 0 -0.05 0.079 -0.09 0.194 -0.105 0.027 0.259 0 0.076 0 -0.022 -0.087 0.08 0.055 0 -0.073 0.02 0.042

1 2 0.043 -0.147 0 0 0.074 0 0 -0.05 0.079 -0.09 0.264 -0.105 0.027 0.259 0 0.076 0 0.056 -0.087 0.08 0.055 -0.053 -0.073 0.02 -0.005

1 2 0.043 -0.147 0 0 0.074 0 0 -0.05 0.079 -0.09 0.264 -0.105 0.027 0.259 0 0.076 0 0.056 -0.087 0.08 0.055 -0.026 -0.073 0.02 -0.005

1 2 0.043 -0.147 0 0 0.074 0 0 -0.05 0.079 -0.09 0.264 -0.105 0.027 0.259 0 0.076 0 0.056 -0.087 0.08 0.055 0 -0.073 0.02 0.068

1 2 0.043 -0.098 0 0 0.074 0 0 -0.05 0.079 -0.09 0.194 -0.105 0.027 0.259 0 -0.002 0 0.056 -0.087 0.08 0.055 0 -0.073 0.02 0.042

1 2 0.043 -0.147 0 -0.034 0.074 0 0 -0.05 0.079 -0.09 0.194 -0.105 0.027 0.259 0 0.076 0 0.056 -0.087 0.08 0.055 0 -0.073 0.02 0.042

1 2 0.043 -0.147 0 0 0.074 0 0 -0.05 0.079 -0.09 0.245 -0.105 0.027 0.259 0 0.076 0 0.056 -0.087 0.08 0.055 0 0.026 0.02 0.042

1 2 0.043 -0.098 0.103 0 0.074 0 0 -0.05 0.079 -0.09 0.194 -0.105 0.027 0.259 0 0.076 0 0.056 -0.087 0.08 0.055 0.026 -0.073 0.02 0.042

1 2 0.043 -0.098 0 0 0.164 0 0.208 -0.05 0.079 -0.09 0.194 -0.105 0.027 0.259 0 0.076 0 0.056 -0.087 0.08 0.055 0.026 -0.073 0.02 0.042

1 2 0.043 -0.147 0 0 0.074 0 0 -0.05 0.231 -0.09 0.264 -0.105 0.027 0.259 0 0.076 0 0.056 -0.087 0.08 0.055 0 -0.073 0.02 0.042

1 2 0.043 -0.147 0 0 0.074 0 0 -0.05 0.079 -0.09 0.264 -0.105 0.027 0.259 0 0.076 0 0.056 -0.087 0.08 0.055 0 -0.073 0.075 0.042

1 2 0.152 -0.098 0 0 0.074 0 0.208 -0.05 0.079 -0.09 0.194 -0.105 0.027 0.259 -0.14 0.076 0 0.056 -0.087 0.08 0.055 0.026 -0.073 0.02 0.042

1 2 0.043 -0.147 0 0 0.074 0 0 -0.05 0.079 -0.09 0.264 -0.105 0.027 0.259 0 0.025 0 0.056 -0.087 0.08 0.055 0 -0.073 0.02 0.042

1 2 0.095 -0.147 0 0 0.074 0 0 -0.05 0.079 -0.09 0.264 -0.105 0.027 0.259 0 0.076 0 0.056 -0.087 0.08 0.055 0 -0.073 0.02 0.042

1 2 0.043 -0.098 0 0 0.074 0 0 -0.05 0.079 -0.09 0.194 -0.105 -0.053 0.259 0 0.076 0 0.056 -0.087 0.08 0.055 0 -0.073 0.02 0.042

1 2 0.043 -0.098 0 0 0.074 0 0 -0.05 0.079 -0.09 0.194 -0.105 0.027 0.259 0.333 0.076 0 0.056 -0.087 0.08 0.055 0 -0.073 0.02 0.042

1 2 0.043 -0.098 0 0 0.074 0 0.208 -0.05 0.079 -0.09 0.194 -0.105 -0.158 0.259 -0.14 0.076 0 0.056 -0.087 0.08 0.055 0.026 -0.073 0.02 0.042

1 2 0.043 -0.147 0 0 0.074 0 0 -0.05 0.079 -0.09 0.264 -0.105 0.027 0.259 0 0.076 0 0.056 -0.087 0.034 0.055 0 -0.073 0.02 0.042

1 2 0.043 -0.147 0 0 0.074 0 0 -0.05 0.079 -0.09 0.264 -0.105 0.027 0.259 0 0.076 0 0.056 -0.308 0.08 0.055 0 -0.073 0.02 0.042

1 2 0.043 -0.098 0 0 0.074 0 0 -0.05 0.079 -0.09 0.194 -0.105 0.027 0.259 0 0.076 0 0.056 -0.087 0.08 0.055 0.026 -0.073 0.066 0.042

1 2 0.043 -0.098 0 0 0.074 0 0 -0.05 0.079 -0.09 0.194 -0.105 0.026 0.259 0 0.076 0 0.056 -0.087 0.08 0.055 0 -0.073 0.02 0.042

1 2 0.043 -0.147 0 0 0.074 0 0 -0.05 0.103 -0.09 0.264 -0.105 0.027 0.259 0 0.076 0 0.056 -0.087 0.08 0.007 0 -0.073 0.02 0.042

1 2 0.043 -0.098 0 0 0.074 0 0 -0.05 0.079 -0.09 0.194 -0.105 0.027 0.259 0 0.076 0 0.056 -0.087 0.08 0.055 0 -0.073 0.101 0.042

1 2 0.043 -0.098 0 0 0.074 0 0 -0.05 0.079 -0.09 0.194 -0.105 0.027 0.259 0 0.076 -0.01 0.056 -0.087 0.08 0.055 0 -0.073 0.02 0.042

1 2 0.043 -0.147 0 0 0.074 0 0 -0.05 0.079 -0.09 0.264 -0.105 0.027 0.259 -0.077 0.076 0 0.056 -0.087 0.08 0.055 0 -0.073 0.02 0.042

1 1 0.043 -0.098 0 0 0.074 0 0.208 -0.05 0.079 -0.122 0.194 -0.105 0.027 0.259 0 0.076 0 0.056 -0.087 0.08 0.055 0.026 -0.073 0.02 0.042

1 1 0.043 -0.098 0 0 0.074 0 0 -0.05 0.079 -0.09 0.194 -0.105 0.027 0.259 0 0.076 0 0.056 -0.087 0.08 0.055 0.026 -0.042 0.02 0.042

1 1 0.043 -0.098 0.072 0 0.074 0 0 -0.05 0.079 -0.09 0.194 -0.105 0.027 0.259 0 0.076 0 0.056 -0.087 0.08 0.055 0 -0.073 0.02 0.042

1 1 0.043 -0.098 0 0 0.074 0 0 -0.05 0.079 -0.09 0.194 -0.105 0.027 0.259 0 0.076 0 0.056 -0.087 0.08 0.055 0.026 -0.045 0.02 0.042

1 1 0.043 -0.147 0 0 0.074 0 0 -0.05 0.079 -0.09 0.264 -0.105 0.027 0.259 0 0.076 0 0.056 -0.087 0.08 0.055 0 -0.073 0.039 -0.005

1 1 0.043 -0.147 0 0 0.074 0 0 -0.05 0.079 -0.09 0.264 -0.105 0.027 0.259 0 0.076 0 0.114 -0.087 0.08 0.055 0 -0.073 0.02 0.042

1 1 0.043 -0.098 0 0 0.074 0 0.208 -0.05 0.079 -0.09 0.194 -0.105 0.027 0.259 0 0.076 0 0.018 -0.087 0.08 0.055 0.026 -0.073 0.02 0.042

1 1 0.043 -0.147 0 0 0.074 0 0 -0.05 0.079 -0.09 0.264 -0.105 0.027 0.259 0 0.076 0 0.056 -0.087 0.08 0.055 0 -0.073 0.02 -0.037

1 1 0.043 -0.098 0 0 0.074 0 0.208 -0.05 0.079 -0.09 0.219 -0.105 0.027 0.259 0 0.076 0 0.056 -0.087 0.08 0.055 0.026 -0.073 0.02 0.042

1 1 0.043 -0.147 0 0 0.074 0 0 -0.05 0.079 -0.09 0.264 -0.105 0.027 0.359 0 0.076 0 0.056 -0.087 0.08 0.055 0 -0.073 0.02 0.042

1 1 0.043 -0.147 0 0 0.074 0 0 -0.05 0.079 -0.09 0.264 -0.105 0.027 0.259 0 0.076 0.232 0.056 -0.087 0.08 0.055 0 -0.073 0.02 0.042

1 1 0.043 -0.147 0 0 0.239 0 0 -0.05 0.079 -0.09 0.264 -0.105 0.027 0.259 0 0.076 0 0.056 -0.087 0.08 0.055 0 -0.073 0.02 -0.005

1 1 0.043 -0.098 0 0 0.074 0 0.208 -0.05 0.079 -0.09 0.194 -0.105 0.027 0.259 0 0.076 0 0.056 -0.087 0.08 0.055 -0.109 -0.073 0.02 0.042

1 1 0.043 -0.147 0 0 0.074 0 0.038 -0.05 0.079 -0.09 0.264 -0.105 0.027 0.259 0 0.076 0 0.056 -0.087 0.08 0.055 0 -0.073 0.02 -0.005

1 1 0.043 -0.161 0 0 0.074 0 0 -0.05 0.079 -0.09 0.264 -0.105 0.027 0.259 0 0.076 0 0.056 -0.122 0.102 0.055 0 -0.073 0.02 -0.005

1 1 0.043 -0.147 0 0 0.074 0 0 -0.05 0.079 -0.09 0.264 -0.105 0.027 0.259 0 0.076 0 0.056 -0.004 0.08 0.055 0 -0.073 0.02 0.042

1 1 0.043 -0.147 0 0 0.074 0 0 -0.05 0.079 -0.09 0.264 -0.105 0.027 0.259 0 0.076 0 0.056 -0.087 0.08 0.055 0 -0.073 0.098 -0.005

1 1 0.043 -0.147 0 0 0.074 0 0 -0.05 0.079 -0.09 0.264 -0.105 0.027 0.259 0 0.208 0 0.056 -0.087 0.08 0.055 0 -0.073 0.02 0.042

1 1 0.043 -0.147 0 0 0.074 0 0 -0.05 0.079 -0.09 0.264 -0.105 0.027 0.259 0 0.076 0 0.056 -0.087 0.08 0.055 0 -0.051 0.02 -0.005

1 1 0.043 -0.147 0 0 0.074 0 0 -0.05 0.008 -0.09 0.264 -0.105 0.027 0.259 0 0.076 0 0.056 -0.087 0.08 0.055 0 -0.073 0.02 -0.005

1 1 -0.235 -0.098 0 0 0.074 0 0.208 -0.05 0.079 -0.09 0.194 -0.105 0.027 0.259 0 0.076 0 0.056 -0.087 0.08 0.055 0.026 -0.073 0.02 0.042

1 1 0.043 -0.098 0 0 0.074 0 0.208 -0.05 0.079 -0.09 0.194 -0.105 0.027 0.259 0 0.076 0 0.056 0.031 0.08 0.055 0.026 -0.073 0.02 0.042

1 1 0.043 -0.098 0 0 0.074 0 0 -0.05 0.079 -0.09 0.194 -0.105 0.027 0.259 0 0.076 0 0.056 -0.087 0.08 0.138 0 -0.073 0.02 0.042

1 1 0.043 -0.098 0 0 0.074 0 0 -0.05 0.079 -0.09 0.194 -0.105 0.027 0.259 0 0.076 0 0.056 -0.087 0.08 0.137 0.026 -0.073 0.02 0.042

1 1 0.043 -0.098 0 0 0.074 0 0 -0.05 0.079 -0.09 0.194 -0.105 0.027 0.259 -0.006 0.076 0 0.056 -0.087 0.08 0.055 0 -0.073 0.02 0.042

1 1 0.043 -0.147 0 0 0.074 0 0 -0.05 0.079 -0.09 0.264 -0.105 0.027 0.259 0 0.076 0 0.056 -0.013 0.08 0.055 0 -0.073 0.02 -0.005

1 1 0.043 -0.098 0 0 0.074 0 0.208 -0.05 0.096 -0.09 0.194 -0.105 0.027 0.259 0 0.076 0 0.056 -0.087 0.08 0.055 0.026 -0.073 0.02 0.042

1 1 0.043 -0.098 0 0 0.074 0 -0.05 -0.05 0.079 -0.09 0.194 -0.105 0.027 0.259 0 0.076 0 0.056 -0.087 0.08 0.055 0 -0.073 0.02 0.042

1 1 0.043 -0.098 0 0 0.074 0 0 -0.05 0.079 -0.09 0.194 -0.105 0.027 0.188 0 0.076 0 0.056 -0.087 0.08 0.055 0.026 -0.073 0.02 0.042

1 1 0.043 -0.147 0 0 0.074 0 0 -0.05 0.079 -0.195 0.264 -0.105 0.027 0.259 0 0.076 0 0.056 -0.087 0.08 0.055 0 -0.073 0.02 0.042

1 1 0.043 -0.07 0 0 0.074 0 0 -0.05 0.079 -0.09 0.194 -0.105 0.027 0.259 0 0.076 0 0.056 -0.087 0.08 0.055 0 -0.072 0.02 0.042

1 1 0.043 -0.098 0 0 0.074 0 0 -0.05 0.079 -0.09 0.044 -0.105 0.027 0.259 0 0.076 0 0.056 -0.087 0.08 0.055 0 -0.073 0.02 0.042

1 1 0.043 -0.039 0 0 0.074 0 0 -0.05 0.079 -0.09 0.194 -0.105 0.027 0.259 0 0.076 0 0.056 -0.087 0.08 0.055 0.026 -0.073 0.02 0.042

1 1 0.043 -0.098 0 0 0.074 0 0 -0.05 -0.021 -0.09 0.194 -0.105 0.027 0.259 0 0.076 0 0.056 -0.087 0.08 0.055 0 -0.073 0.02 0.042

1 1 0.043 -0.098 0 0 0.074 0 0.208 -0.05 0.079 -0.09 0.194 -0.115 0.027 0.259 -0.14 0.076 0 0.056 -0.087 0.08 0.055 0.026 -0.073 0.02 0.042

1 1 0.322 -0.098 0 0 0.074 0 0 -0.05 0.079 -0.09 0.194 -0.105 0.027 0.259 0 0.076 0 0.056 -0.087 0.08 0.055 0 -0.073 0.02 0.042

1 1 0.043 -0.098 0 -0.04 0.074 0 0.208 -0.05 0.079 -0.09 0.194 -0.105 0.027 0.259 0 0.076 0 0.056 -0.087 0.08 0.055 0.026 -0.073 0.02 0.042

1 1 0.043 -0.147 0 0 0.114 0 0 -0.05 0.079 -0.09 0.264 -0.105 0.027 0.259 0 0.076 0 0.056 -0.087 0.08 0.055 0 -0.073 0.02 0.042

1 1 0.043 -0.147 0 0 0.074 0 0 -0.05 0.079 -0.09 0.194 -0.105 0.027 0.259 0 0.076 -0.046 0.056 -0.087 0.08 0.055 0 -0.073 0.02 0.042

1 1 0.043 -0.147 0 0 0.074 0 0 -0.05 0.079 -0.09 0.194 -0.105 0.027 0.259 -0.216 0.076 0 0.056 -0.087 0.08 0.055 0 -0.073 0.02 0.042

1 1 0.043 -0.098 0 0 0.074 0 0 -0.05 0.079 -0.09 0.194 -0.105 0.027 0.259 0 0.076 0 0.056 -0.087 0.08 0.159 0.026 -0.073 0.02 0.042

1 1 0.043 -0.147 0 -0.009 0.074 0 0 -0.05 0.079 -0.09 0.264 -0.105 0.027 0.259 0 0.076 0 0.056 -0.055 0.08 0.055 0 -0.073 0.02 0.042

1 1 0.043 -0.098 0 0 0.074 -0.126 0.208 -0.05 0.079 -0.09 0.194 -0.105 0.027 0.259 -0.14 0.076 0 0.056 -0.087 0.08 0.055 0.026 -0.073 0.02 0.042

1 1 0.043 -0.098 0 0 0.074 0 0.208 -0.05 0.079 -0.09 0.194 -0.205 0.027 0.259 -0.14 0.076 0 0.056 -0.087 0.08 0.055 0.026 -0.073 0.02 0.042

1 1 0.043 -0.147 0 0 0.074 0 0 -0.05 0.079 -0.09 0.264 -0.105 0.027 0.259 -0.053 -0.142 0 0.056 -0.087 0.08 0.055 0 -0.073 0.02 0.042

1 1 -0.125 -0.098 0 0 0.074 0 0 -0.05 0.079 -0.09 0.194 -0.105 0.027 0.259 0 0.076 0 0.056 -0.087 0.08 0.055 0.026 -0.073 0.02 0.042

1 1 0.043 -0.098 0 0 0.074 0 0 -0.05 0.079 -0.09 0.194 -0.105 0.027 0.259 0 0.076 0 0.056 -0.087 0.114 0.055 0 -0.073 0.02 0.042

1 1 0.043 -0.098 0 0 0.074 0 0.208 -0.05 0.079 -0.09 0.194 -0.105 0.027 0.38 0 0.076 0 0.056 -0.087 0.08 0.055 0.026 -0.073 0.02 0.042

1 1 0.043 -0.098 0 0 0.074 0 0.027 -0.05 0.079 -0.09 0.194 -0.105 0.027 0.259 0 0.076 0 0.056 -0.087 0.08 0.055 0 -0.073 0.02 0.042

1 1 0.043 -0.147 0 0 0.074 0 0 -0.05 0.079 -0.09 0.264 -0.105 0.027 0.259 0 0.076 0 0.056 -0.087 0.08 0.055 0 -0.086 0.02 0.042

1 1 0.043 -0.147 0 0 0.074 0 0 -0.05 0.079 -0.09 0.264 -0.105 0.027 0.259 0 0.076 0 0.056 -0.087 0.08 -0.061 0 -0.073 0.02 0.042

1 1 0.043 -0.147 0 0 0.074 0 0 -0.05 0.079 -0.09 0.264 -0.105 0.027 0.259 0 0.076 0 0.056 -0.055 0.08 0.055 -0.041 -0.073 0.02 0.042

1 1 0.043 -0.162 0 0 0.074 0 0 -0.05 0.079 -0.09 0.264 -0.105 0.027 0.259 0 0.076 0 0.056 -0.087 0.08 0.055 0 -0.073 0.02 0.042

1 1 0.043 -0.098 0 0.124 0.074 0 0.208 -0.05 0.079 -0.09 0.194 -0.105 0.027 0.259 0 0.076 0 0.056 -0.087 0.08 0.055 0.026 -0.073 0.02 0.042

1 1 0.043 -0.098 0 0 0.074 0 0 -0.05 0.079 -0.09 0.194 -0.105 0.027 0.259 0 0.076 0 0.056 -0.087 0.08 0.055 0.026 -0.073 0.02 0.021

1 1 0.043 -0.098 0 0 0.074 0 0 -0.05 0.079 -0.09 -0.048 -0.105 0.027 0.259 0 0.076 0 0.056 -0.087 0.08 0.055 0 -0.073 0.02 0.042

1 1 0.043 -0.147 0 0 0.074 0 0 -0.05 0.079 -0.09 0.264 -0.105 0.027 0.259 0 0.076 -0.057 0.056 -0.087 0.08 0.055 0 -0.073 0.02 0.042

1 1 0.043 -0.147 0 0 0.074 0 0 -0.05 0.079 -0.09 0.264 -0.105 0.027 0.259 0 0.076 0 0.056 -0.077 0.08 0.055 0 -0.073 0.02 0.042

1 1 0.043 -0.147 0 0 0.074 0 0 -0.05 0.079 -0.09 0.264 -0.105 0.027 0.259 0 0.076 0 0.056 -0.087 0.08 0.055 -0.115 -0.073 0.02 0.042

1 1 0.043 -0.098 0 0 0.074 0 0.208 -0.05 0.079 -0.09 0.194 -0.105 0.027 0.259 0 0.076 0 0.056 -0.087 0.08 0.055 0.026 -0.073 -0.04 0.042

1 1 0.043 -0.098 0 0 0.074 0 0 -0.05 0.079 -0.09 0.194 -0.105 0.027 0.188 0 0.076 0 0.056 -0.026 0.08 0.055 0.026 -0.073 0.02 0.042

1 1 0.043 -0.147 0 0 0.074 0 0 0.134 0.079 -0.09 0.264 -0.105 0.027 0.259 0 0.076 0 0.056 -0.087 0.08 0.055 0 -0.073 0.02 0.042

1 1 0.043 -0.098 0 0 0.074 0 0 -0.05 0.079 -0.09 0.194 -0.105 0.027 0.259 0 0.076 0 0.056 -0.087 0.08 0.16 0 -0.073 0.02 0.042

1 1 0.043 -0.147 0 0 0.074 0 0 -0.05 0.079 -0.027 0.264 -0.105 0.027 0.259 0 0.076 0 0.056 -0.087 0.08 0.055 0 -0.073 0.02 0.042

1 1 0.043 -0.147 0 0 0.074 0 0.002 -0.05 0.079 -0.09 0.264 -0.105 0.027 0.259 0 0.076 0 0.056 -0.087 0.08 0.055 0 -0.073 0.02 0.042

1 1 0.043 -0.098 0 0 0.074 0 -0.065 -0.05 0.079 -0.09 0.194 -0.105 0.027 0.259 0 0.076 0 0.056 -0.087 0.08 0.055 0 -0.073 0.02 0.042

1 1 0.043 -0.098 0 0 0.074 -0.096 0 -0.05 0.079 -0.09 0.194 -0.105 0.027 0.259 0 0.076 0 0.056 -0.087 0.08 0.055 0.026 -0.073 0.02 0.042

1 1 0.043 -0.147 0 0 0.074 0 0 -0.05 0.079 -0.09 0.264 -0.105 -0.016 0.259 0 0.076 0 0.056 -0.055 0.08 0.055 0 -0.073 0.02 0.042

1 1 0.043 -0.098 0 0 0.074 0 0.208 -0.05 0.079 -0.09 0.194 -0.105 0.027 0.259 0 0.076 0 0.056 -0.087 0.08 0.055 0.026 -0.073 0.02 -0.036

1 1 0.043 -0.147 0 0 0.074 0 -0.008 -0.05 0.079 -0.09 0.264 -0.105 0.027 0.259 0 0.076 0 0.056 -0.087 0.08 0.055 0 -0.073 0.02 -0.005

1 1 0.043 -0.147 0 0 0.074 0 0 -0.05 0.079 -0.09 0.264 -0.105 0.062 0.259 0 0.076 0 0.056 -0.087 0.08 0.007 0 -0.073 0.02 0.042

1 1 0.043 -0.098 0 0 0.074 0 0 -0.05 0.079 -0.09 0.194 -0.105 0.027 0.259 0 0.076 0.018 0.056 -0.087 0.08 0.055 0 -0.073 0.02 0.042

1 1 0.043 -0.147 0 0 0.074 0 -0.113 -0.05 0.079 -0.09 0.264 -0.105 0.027 0.259 0 0.076 0 0.056 -0.055 0.08 0.055 0 -0.073 0.02 0.042

1 1 0.043 -0.098 0 0 0.074 0 0 -0.05 0.079 -0.09 0.194 -0.105 0.027 0.259 0 0.076 0 0.056 -0.087 0.08 0.055 0.026 -0.073 0.02 0.066

1 1 0.043 -0.147 0 0 0.074 0 0 -0.05 0.079 -0.09 0.264 -0.105 0.027 0.259 0 0.076 0.072 0.056 -0.087 0.08 0.055 0 -0.073 0.02 0.042

1 1 0.043 -0.098 0 0 0.074 0 -0.061 -0.05 0.079 -0.09 0.194 -0.105 0.027 0.259 0 0.076 0 0.056 -0.087 0.08 0.055 0.026 -0.073 0.02 0.042

1 1 0.043 -0.098 0 0 0.074 0 0.208 -0.05 0.079 -0.09 0.194 -0.105 0.027 0.259 0 0.076 0.014 0.056 -0.087 0.08 0.055 0.026 -0.073 0.02 0.042

1 1 -0.039 -0.147 0 0 0.074 0 0 -0.05 0.079 -0.09 0.264 -0.105 0.027 0.259 0 0.076 0 0.056 -0.087 0.08 0.055 0 -0.073 0.02 0.042

1 1 0.043 -0.098 0 0 0.074 0 0 -0.05 0.079 -0.109 0.194 -0.105 0.027 0.259 0 0.076 0 0.056 -0.087 0.08 0.055 0.026 -0.073 0.02 0.042

1 1 0.043 -0.098 0 0 0.074 0 0.208 -0.05 0.079 -0.09 0.194 -0.102 0.027 0.259 0 0.076 0 0.056 -0.087 0.08 0.055 0.026 -0.073 0.02 0.042

1 1 0.043 -0.147 0 -0.128 0.074 0 0 -0.05 0.079 -0.09 0.264 -0.105 0.027 0.259 0 0.076 0 0.056 -0.055 0.08 0.055 0 -0.073 0.02 0.042

2 418 0 -0.044 -0.061 0 0.051 -0.122 -0.356 -0.054 0.06 0.125 -0.278 0 0.136 -0.005 0.067 0.16 -0.185 -0.116 0.006 0.142 0 0 -0.156 0.077 0.023

2 371 0 -0.044 0 0 0.051 -0.33 -0.396 -0.02 0.06 0.125 -0.194 0.014 0.136 0.048 0.12 0.16 -0.204 -0.116 0.006 0.142 0 0 -0.156 0.077 0.023

2 350 0 -0.044 0 0 0.051 -0.122 -0.356 -0.054 0.06 0.125 -0.278 0 0.136 -0.005 0.067 0.16 -0.185 -0.116 0.006 0.142 0 0 -0.156 0.077 0.023

2 274 0 -0.044 0 0 0.051 -0.33 -0.396 -0.02 0.06 0.125 -0.194 0 0.136 -0.005 0.067 0.16 -0.204 -0.116 0.006 0.142 0 0 -0.156 0.077 0.023

2 383 0 -0.044 0 0 0.051 -0.121 -0.356 -0.054 0.06 0.125 -0.194 0 0.274 -0.005 0.067 0.16 -0.204 -0.116 0.006 0.142 0 -0.013 -0.156 0.077 0.023

2 207 0 -0.044 0 0 0.051 -0.121 -0.356 -0.054 0.06 0.125 -0.278 0 0.136 -0.005 0.067 0.16 -0.185 -0.116 0.006 0.142 0 0 -0.156 0.077 0.023

2 173 0 -0.044 0 0 0.051 -0.121 -0.356 -0.054 0.06 0.125 -0.194 -0.199 0.274 -0.005 0.067 0.16 -0.204 -0.116 0.006 0.142 0 -0.013 -0.156 0.077 0.023

2 153 0 -0.044 0 0 0.051 -0.122 -0.356 -0.054 0.06 0.125 -0.278 0 0.136 0.082 0.067 0.16 -0.185 -0.116 0.006 0.142 0 0 -0.156 0.077 0.023

2 146 0 -0.044 0 0 0.051 -0.121 -0.356 -0.054 0.06 0.125 -0.194 0 0.274 -0.005 0.067 0.172 -0.204 -0.116 0.006 0.142 0 -0.013 -0.156 0.077 0.023

2 259 0 -0.044 0 0 0.051 -0.121 -0.356 -0.054 0.06 0.125 -0.278 -0.062 0.136 -0.005 0.067 0.16 -0.204 -0.116 0.006 0.142 0 0 -0.156 0.077 0.042

2 65 0.06 -0.044 0 0 0.051 -0.121 -0.356 -0.054 0.06 0.125 -0.194 0 0.136 -0.005 0.067 0.176 -0.204 -0.116 0.006 0.142 0 -0.013 -0.156 0.077 0.023

2 54 0 -0.044 0 0 0.051 -0.33 -0.396 -0.02 0.06 0.125 -0.194 0.014 0.136 0.048 0.067 0.16 -0.204 -0.116 0.006 0.142 0 0 -0.156 0.077 0.023

2 41 0 -0.044 0 0 0.051 -0.33 -0.396 -0.02 0.06 0.125 -0.194 0 0.136 -0.005 0.067 0.16 -0.204 -0.116 0.006 0.142 0 0.128 -0.156 0.077 0.023

2 39 0.144 -0.044 0 0 0.051 -0.122 -0.356 -0.054 0.06 0.125 -0.278 0 0.136 -0.005 0.067 0.16 -0.185 -0.116 0.006 0.142 0 0 -0.156 0.077 0.023

2 27 0 -0.044 0 0 0.051 -0.121 -0.399 -0.054 0.06 0.125 -0.278 0 0.136 -0.005 0.067 0.16 -0.185 -0.116 0.006 0.142 0 0 -0.156 0.077 0.023

2 25 0 -0.044 0 0 0.051 -0.121 -0.356 -0.054 0.06 0.125 -0.278 -0.062 0.136 -0.005 0.067 0.16 -0.204 -0.116 -0.111 0.142 0 0 -0.156 0.077 0.042

2 25 0 -0.044 0 0 0.051 -0.122 -0.356 -0.054 0.06 0.125 -0.278 0 0.136 -0.005 -0.127 0.16 -0.185 -0.116 0.006 0.142 0 0 -0.156 0.077 0.023

2 22 0 -0.044 0 0 0.051 -0.122 -0.356 -0.054 0.06 0.052 -0.278 0 0.136 -0.005 0.067 0.16 -0.185 -0.116 0.006 0.142 0 0 -0.156 0.077 0.023

2 12 0 -0.044 -0.061 0 0.051 -0.122 -0.356 -0.054 0.06 0.125 -0.278 0.074 0.136 -0.005 0.067 0.16 -0.185 -0.116 0.006 0.142 0 0 -0.156 0.077 0.023

2 11 -0.008 -0.044 0 0 0.051 -0.33 -0.396 -0.02 0.06 0.125 -0.194 0.014 0.136 0.048 0.12 0.16 -0.204 -0.116 0.006 0.142 0 0 -0.156 0.077 0.023

2 8 0 -0.112 0 0 0.051 -0.121 -0.356 -0.054 0.06 0.125 -0.278 0 0.136 -0.005 0.067 0.16 -0.185 -0.116 0.006 0.142 0 0 -0.156 0.077 0.023

2 8 0 -0.044 0 0 0.051 -0.122 -0.356 -0.054 0.052 0.125 -0.278 0 0.136 0.082 0.067 0.16 -0.185 -0.116 0.006 0.142 0 0 -0.156 0.077 0.023

2 8 0 -0.044 0 0 0.051 -0.121 -0.356 -0.054 0.06 0.125 -0.194 0 0.274 -0.005 0.067 0.172 -0.204 -0.116 0.006 0.117 0 -0.013 -0.156 0.077 0.023

2 7 0 -0.044 0 0 0.051 -0.33 -0.462 -0.02 0.06 0.125 -0.194 0 0.136 -0.005 0.067 0.16 -0.204 -0.116 0.006 0.142 0 0 -0.156 0.077 0.023

2 7 0 -0.044 0 0 0.051 -0.122 -0.356 -0.054 0.06 0.125 -0.278 0 0.136 0.057 0.067 0.16 -0.185 -0.116 0.006 0.142 0 0 -0.156 0.077 0.023

2 6 0.144 -0.044 0 0 0.051 -0.122 -0.356 -0.054 0.06 0.125 -0.183 0 0.136 -0.005 0.067 0.16 -0.185 -0.116 0.006 0.142 0 0 -0.156 0.077 0.023

2 6 0.144 -0.044 0 0 0.051 -0.122 -0.356 -0.054 0.06 0.221 -0.278 0 0.136 -0.005 0.067 0.16 -0.185 -0.116 0.006 0.142 0 0 -0.156 0.077 0.023

2 6 0 -0.044 0 0 0.051 -0.121 -0.34 -0.054 0.06 0.125 -0.194 0 0.274 -0.005 0.067 0.172 -0.204 -0.116 0.006 0.142 0 -0.013 -0.156 0.077 0.023

2 6 0 -0.044 0 0 0.051 -0.33 -0.396 -0.02 0.06 0.125 -0.194 0 0.1 -0.005 0.067 0.16 -0.204 -0.116 0.006 0.142 0 0 -0.091 0.077 0.023

2 6 0 -0.044 0 0 0.051 -0.121 -0.356 -0.054 0.06 0.125 -0.194 0 0.274 -0.005 0.067 0.16 -0.204 -0.116 0.006 0.142 0 -0.013 -0.194 0.077 0.023

2 5 0 -0.044 0 0 0.051 -0.121 -0.356 -0.054 0.127 0.125 -0.278 0 0.136 -0.005 0.067 0.071 -0.185 -0.116 0.006 0.142 0 0 -0.156 0.077 0.023

2 5 0 -0.044 0 0 0.051 -0.121 -0.356 -0.054 0.06 0.125 -0.194 0 0.274 -0.005 0.067 0.16 -0.163 -0.116 0.006 0.142 0 -0.013 -0.156 0.077 0.023

2 4 0.232 -0.044 -0.061 0 0.051 -0.122 -0.356 -0.054 0.06 0.125 -0.278 0 0.136 -0.005 0.067 0.16 -0.185 -0.116 0.006 0.142 0 0 -0.156 0.077 0.023

2 4 0 -0.044 0 0 0.051 -0.121 -0.356 -0.054 0.06 0.125 -0.278 0 0.136 -0.005 0.067 0.071 -0.185 -0.116 0.006 0.142 0 0 -0.156 0.077 0.023

2 4 0 -0.044 0 0 0.051 -0.121 -0.356 -0.054 0.06 0.125 -0.278 0 0.136 -0.005 0.067 0.16 -0.185 -0.153 0.006 0.142 0 0 -0.156 0.077 0.023

2 4 0 -0.044 0 0 0.051 -0.121 -0.356 -0.054 0.06 0.125 -0.278 0 0.136 -0.005 0.067 0.16 -0.204 -0.021 0.057 0.142 0 0 -0.156 0.077 0.023

2 4 0 -0.044 0 0 0.051 -0.121 -0.356 -0.054 0.06 0.125 -0.194 0 0.182 -0.005 0.067 0.16 -0.204 -0.116 0.006 0.142 0 -0.013 -0.156 0.077 0.023

2 4 0 -0.044 0 0 0.012 -0.121 -0.356 -0.054 0.06 0.125 -0.278 0 0.136 -0.005 0.067 0.16 -0.185 -0.116 0.006 0.142 0 0 -0.156 0.077 0.023

2 4 0 -0.044 0 0 0.051 -0.121 -0.356 -0.188 0.06 0.125 -0.194 0 0.274 -0.005 0.067 0.16 -0.204 -0.116 0.006 0.142 0 -0.013 -0.156 0.077 0.023

2 4 0 -0.044 -0.061 0 0.051 -0.336 -0.356 -0.054 0.06 0.125 -0.278 0 0.136 -0.005 0.067 0.16 -0.185 -0.116 0.006 0.142 0 0 -0.156 0.077 0.023

2 4 0 -0.044 0 0 0.051 -0.33 -0.396 -0.02 0.06 0.125 -0.194 0 0.136 -0.005 0.067 0.16 -0.204 -0.116 0.006 0.142 0 0 -0.091 0.077 0.023

2 4 0 -0.044 0 0 0.051 -0.33 -0.396 -0.02 0.06 0.125 -0.194 0.014 0.136 0.048 0.067 0.16 -0.256 -0.116 0.006 0.142 0 0 -0.156 0.077 0.023

2 4 0 -0.044 0 0 0.051 -0.121 -0.356 -0.054 0.06 0.125 -0.278 -0.062 0.136 -0.005 0.164 0.16 -0.204 -0.116 -0.111 0.142 0 0 -0.156 0.077 0.042

2 4 0 -0.044 0 0 0.051 -0.33 -0.396 -0.02 0.06 0.125 -0.194 0.014 0.136 0.048 0.12 0.16 -0.199 -0.116 0.006 0.142 0 0 -0.156 0.077 0.023

2 4 0 -0.044 0 0 0.051 -0.121 -0.356 -0.054 0.06 0.125 -0.278 0 0.136 -0.005 0.067 0.16 -0.185 -0.116 0.006 0.142 0 0 -0.153 0.077 0.023

2 4 0 -0.044 0 -0.075 0.051 -0.33 -0.396 -0.02 0.06 0.125 -0.194 0.014 0.136 0.048 0.12 0.16 -0.204 -0.116 0.006 0.142 0 0 -0.156 0.077 0.023

2 3 0 -0.044 0 0 0.051 -0.121 -0.356 -0.054 0.06 0.125 -0.194 0 0.274 -0.005 0.067 0.16 -0.316 -0.116 0.006 0.142 0 -0.013 -0.156 0.077 0.023

2 3 0 -0.044 0 0 0.051 -0.122 -0.356 -0.054 0.06 0.125 -0.325 0 0.136 -0.005 0.067 0.16 -0.185 -0.116 0.006 0.142 0 0 -0.156 0.077 0.023

2 3 0 -0.044 0 0 0.051 -0.121 -0.356 -0.054 0.06 0.125 -0.194 0 0.274 -0.005 0.192 0.172 -0.204 -0.116 0.006 0.142 0 -0.013 -0.156 0.077 0.023

2 3 0 -0.044 -0.061 0 0.051 -0.122 -0.356 -0.054 0.06 0.125 -0.278 0 0.136 -0.088 0.067 0.16 -0.185 -0.116 0.006 0.142 0 0 -0.156 0.077 0.023

2 2 0 -0.044 -0.061 0 0.051 -0.122 -0.356 -0.054 0.06 0.125 -0.278 0 0.136 0.033 0.067 0.16 -0.185 -0.116 0.006 0.142 0 0 -0.156 0.077 0.023

2 2 0 -0.044 -0.096 0 0.051 -0.121 -0.399 -0.054 0.06 0.125 -0.278 0 0.136 -0.005 0.067 0.16 -0.185 -0.116 0.006 0.142 0 0 -0.156 0.077 0.023

2 2 0 -0.044 0 0 0.051 -0.116 -0.356 -0.054 0.06 0.125 -0.278 0 0.136 -0.005 0.067 0.16 -0.185 -0.116 0.006 0.142 0 0 -0.156 0.077 0.023

2 2 0 -0.044 0 0 0.051 -0.121 -0.356 -0.054 0.06 0.125 -0.194 0 0.274 -0.005 0.067 0.16 -0.163 -0.116 0.006 0.142 0 0.04 -0.156 0.077 0.023

2 2 0 -0.044 0 0 0.056 -0.121 -0.356 -0.054 0.06 0.125 -0.278 -0.062 0.136 -0.005 0.067 0.16 -0.204 -0.116 0.006 0.142 0 0 -0.156 0.077 0.042

2 2 0 -0.044 0 0 0.051 -0.122 -0.356 -0.054 0.06 0.125 -0.278 0 0.136 -0.008 0.067 0.16 -0.185 -0.116 0.006 0.142 0 0 -0.156 0.077 0.023

2 2 0 -0.044 0 0 0.051 -0.121 -0.356 -0.054 0.06 0.125 -0.087 0 0.274 -0.005 0.067 0.16 -0.204 -0.116 0.006 0.142 0 -0.013 -0.156 0.077 0.023

2 2 0 -0.044 0 0 0.051 -0.121 -0.356 -0.054 0.06 0.125 -0.282 0 0.136 -0.005 0.067 0.16 -0.185 -0.116 0.006 0.142 0 0 -0.156 0.077 0.023

2 2 0 -0.044 0 0 0.051 -0.33 -0.396 -0.02 0.06 0.125 -0.194 0 0.136 -0.005 0.067 0.16 -0.204 -0.116 0.006 0.142 0 -0.022 -0.156 0.077 0.023

2 2 0.144 -0.044 0.038 0 0.051 -0.122 -0.356 -0.054 0.06 0.221 -0.278 0 0.136 -0.005 0.067 0.16 -0.185 -0.116 0.006 0.142 0 0 -0.156 0.077 0.023

2 2 0 -0.044 0 0 0.051 -0.121 -0.356 -0.054 0.06 0.125 -0.278 0 0.136 -0.005 0.067 0.16 -0.185 -0.116 0.062 0.142 0 0 -0.156 0.077 0.023

2 2 0 -0.044 0 0 0.051 -0.121 -0.356 -0.054 0.06 0.37 -0.194 0 0.274 -0.005 0.067 0.16 -0.204 -0.116 0.006 0.142 0 -0.013 -0.156 0.077 0.023

2 2 0 -0.044 0 0 0.051 -0.176 -0.396 -0.02 0.06 0.125 -0.194 0 0.136 -0.005 0.067 0.16 -0.204 -0.116 0.006 0.142 0 0 -0.156 0.077 0.023

2 2 0 -0.044 0 0 0.051 -0.121 -0.356 -0.054 0.06 0.125 -0.194 0 0.274 -0.005 0.067 0.16 -0.204 -0.116 0.006 0.142 0 -0.145 -0.156 0.077 0.023

2 2 0 -0.044 -0.061 0 0.051 -0.122 -0.356 -0.054 0.06 0.125 -0.278 0 0.136 -0.005 0.067 0.16 -0.185 -0.116 0.006 0.142 0 0 -0.156 -0.09 0.023

2 2 0 -0.044 0 0 0.051 -0.121 -0.356 -0.054 0.06 0.125 -0.194 -0.199 0.274 -0.005 0.067 0.16 -0.204 -0.116 0.006 0.142 0 -0.013 -0.156 0.112 0.023

2 2 0 -0.044 0 0 0.051 -0.33 -0.396 -0.02 0.06 0.125 -0.194 0.014 0.136 0.048 0.136 0.16 -0.204 -0.116 0.006 0.142 0 0 -0.156 0.077 0.023

2 2 0 -0.044 0 0 0.051 -0.33 -0.396 -0.02 0.06 0.125 -0.194 0.014 0.136 0.048 0.12 0.16 -0.204 -0.116 0.245 0.142 0 0 -0.156 0.077 0.023

2 2 0 -0.044 0 0 0.051 -0.121 -0.356 -0.054 0.06 0.125 -0.151 -0.062 0.136 -0.005 0.067 0.16 -0.204 -0.116 0.006 0.142 0 0 -0.156 0.077 0.042

2 2 0 -0.044 0.071 0 0.051 -0.121 -0.356 -0.054 0.06 0.125 -0.194 -0.199 0.274 -0.005 0.067 0.16 -0.204 -0.116 0.006 0.142 0 -0.013 -0.156 0.077 0.023

2 2 0 -0.044 0 0 0.051 -0.121 -0.356 -0.054 0.06 0.125 -0.194 0 0.274 -0.005 0.067 0.172 -0.204 -0.116 0.006 0.142 0 0.098 -0.156 0.077 0.023

2 2 0 -0.044 0 0 0.051 -0.121 -0.356 -0.143 0.06 0.125 -0.194 0 0.274 -0.005 0.067 0.172 -0.204 -0.116 0.006 0.142 0 -0.013 -0.156 0.077 0.023

2 2 0 -0.044 0 0.147 0.051 -0.121 -0.356 -0.054 0.06 0.125 -0.194 0 0.274 -0.005 0.067 0.172 -0.204 -0.116 0.006 0.142 0 -0.013 -0.156 0.077 0.023

2 2 0 -0.044 -0.061 -0.116 0.051 -0.122 -0.356 -0.054 0.06 0.125 -0.278 0 0.136 -0.005 0.067 0.16 -0.185 -0.116 0.006 0.142 0 0 -0.156 0.077 0.023

2 2 0 -0.044 0 0 0.051 -0.131 -0.356 -0.054 0.06 0.052 -0.278 0 0.136 -0.005 0.067 0.16 -0.185 -0.116 0.006 0.142 0 0 -0.156 0.077 0.023

2 2 0 -0.044 0 0 0.051 -0.152 -0.356 -0.054 0.06 0.125 -0.278 0 0.136 0.082 0.067 0.16 -0.185 -0.116 0.006 0.142 0 0 -0.156 0.077 0.023

2 2 0 -0.044 0 0 0.048 -0.121 -0.356 -0.054 0.06 0.125 -0.194 0 0.274 -0.005 0.067 0.172 -0.204 -0.116 0.006 0.142 0 -0.013 -0.156 0.077 0.023

2 2 0 -0.044 -0.061 0 0.051 -0.122 -0.356 -0.054 0.06 0.125 -0.278 0 0.136 -0.005 0.067 0.16 -0.185 -0.116 -0.12 0.142 0 0 -0.156 0.077 0.023

2 2 0 -0.044 0 0 0.051 -0.33 -0.396 -0.02 0.06 0.125 -0.194 0.014 0.136 0.048 0.12 0.16 -0.204 -0.116 0.006 0.142 0 0 -0.018 0.077 0.023

2 2 0 -0.044 0 0 0.051 -0.121 -0.356 -0.054 0.06 0.125 -0.194 0 0.274 -0.005 0.067 0.16 -0.264 -0.116 0.006 0.142 0 -0.013 -0.156 0.077 0.023

2 2 0 -0.044 0 0 0.051 -0.121 -0.356 -0.054 0.06 0.125 -0.194 -0.199 0.232 -0.005 0.067 0.16 -0.204 -0.116 0.006 0.142 0 -0.013 -0.156 0.077 0.023

2 2 0 -0.044 0 0 0.051 -0.33 -0.448 -0.02 0.06 0.125 -0.194 0.014 0.136 0.048 0.12 0.16 -0.204 -0.116 0.006 0.142 0 0 -0.156 0.077 0.023

2 2 0 -0.044 0 0 0.051 -0.122 -0.356 -0.054 0.035 0.125 -0.278 0 0.136 0.082 0.067 0.16 -0.185 -0.116 0.006 0.142 0 0 -0.156 0.077 0.023

2 2 0 -0.044 -0.077 0 0.051 -0.121 -0.356 -0.143 0.06 0.125 -0.194 0 0.274 -0.005 0.067 0.172 -0.204 -0.116 0.006 0.142 0 -0.013 -0.156 0.077 0.023

2 1 0 -0.044 0 0 0.051 -0.33 -0.396 -0.02 0.06 0.125 -0.194 0 0.136 -0.005 0.094 0.16 -0.061 -0.116 0.006 0.142 0 0 -0.156 0.077 0.023

2 1 0 -0.044 0 0 0.051 -0.121 -0.356 -0.054 0.06 0.125 -0.194 0 0.274 -0.005 0.067 0.16 -0.316 -0.116 -0.005 0.142 0 -0.013 -0.156 0.077 0.023

2 1 0 -0.044 0 0 0.051 -0.121 -0.356 -0.054 0.06 0.125 -0.194 0 0.274 -0.005 0.067 0.16 -0.204 -0.024 0.006 0.142 0 -0.013 -0.156 0.077 0.023

2 1 0 -0.044 0 0 0.051 -0.121 -0.356 -0.054 0.06 0.125 -0.194 0 0.274 -0.005 0.067 0.172 -0.204 -0.116 -0.137 0.142 0 -0.013 -0.156 0.077 0.023

2 1 0 -0.044 -0.061 0 0.051 -0.178 -0.356 -0.054 0.06 0.125 -0.278 0 0.136 -0.005 0.067 0.16 -0.185 -0.116 0.006 0.142 0 0 -0.156 0.077 0.023

2 1 0 -0.044 0 0 0.051 -0.33 -0.396 -0.02 0.06 0.125 -0.194 0.014 0.267 0.048 0.12 0.16 -0.204 -0.116 0.006 0.142 0 0 -0.156 0.077 0.023

2 1 0 -0.044 -0.061 0 0.051 -0.122 -0.356 -0.054 0.06 0.125 -0.278 -0.019 0.136 -0.005 0.067 0.16 -0.185 -0.116 0.006 0.142 0 0 -0.156 0.077 0.023

2 1 0 -0.044 0 0 0.051 -0.122 -0.356 -0.054 0.06 0.125 -0.278 0 0.136 -0.005 0.067 0.16 -0.185 -0.116 0.006 0.142 -0.05 0 -0.156 0.077 0.023

2 1 0 -0.044 0 0 0.051 -0.121 -0.356 -0.054 0.06 0.125 -0.278 -0.062 0.136 -0.005 0.067 0.16 -0.204 -0.116 0.006 0.142 0 0 -0.232 0.077 0.042

2 1 0 -0.044 0 0 0.051 -0.33 -0.396 -0.02 0.06 0.125 -0.194 0.014 0.136 0.048 0.12 0.16 -0.204 -0.116 0.006 0.142 0 0 -0.156 0.077 -0.149

2 1 0 -0.044 0 0 0.051 -0.121 -0.356 -0.054 0.06 0.125 -0.278 0 0.136 -0.116 0.067 0.16 -0.185 -0.116 0.006 0.142 0 0 -0.156 0.077 0.023

2 1 0 -0.044 0 0 0.051 -0.121 -0.442 -0.054 0.06 0.125 -0.278 0 0.136 -0.005 0.067 0.16 -0.185 -0.116 0.006 0.142 0 0 -0.156 0.077 0.023

2 1 0 -0.044 0 0 0.051 -0.33 -0.396 -0.02 0.06 0.14 -0.194 0.014 0.136 0.048 0.12 0.16 -0.204 -0.116 0.006 0.142 0 0 -0.156 0.077 0.023

2 1 0 -0.044 0 0 0.051 -0.122 -0.356 -0.054 0.06 0.125 -0.278 0 0.136 -0.005 0.067 0.16 -0.185 -0.116 -0.126 0.142 0 0 -0.156 0.077 0.023

2 1 0 -0.044 -0.061 0 0.137 -0.122 -0.356 -0.054 0.06 0.125 -0.278 0 0.136 -0.005 0.067 0.16 -0.185 -0.116 0.006 0.142 0 0 -0.156 0.077 0.023

2 1 0 -0.044 0 0 0.051 -0.122 -0.356 -0.054 0.06 0.197 -0.278 0 0.136 0.082 0.067 0.16 -0.185 -0.116 0.006 0.142 0 0 -0.156 0.077 0.023

2 1 0 -0.044 0 0 0.051 -0.33 -0.396 -0.02 0.06 0.125 -0.194 -0.174 0.136 -0.005 0.067 0.16 -0.204 -0.116 0.006 0.142 0 0 -0.156 0.077 0.023

2 1 0 -0.044 -0.061 -0.098 0.051 -0.122 -0.356 -0.054 0.06 0.125 -0.278 0 0.136 -0.005 0.067 0.16 -0.185 -0.116 0.006 0.142 0 0 -0.156 0.077 0.023

2 1 0 -0.044 0 0 0.051 -0.122 -0.356 -0.054 0.06 0.125 -0.278 0 0.136 0.082 0.067 0.16 -0.185 -0.116 0.006 0.142 0 0 -0.156 -0.078 0.023

2 1 0 -0.044 -0.061 0 0.051 -0.122 -0.356 -0.054 0.06 0.125 -0.278 0 0.136 -0.005 0.067 0.16 -0.185 -0.116 0.006 0.142 0 0 -0.096 0.077 0.023

2 1 0 -0.044 0 0 -0.017 -0.121 -0.356 -0.054 0.06 0.125 -0.194 0 0.274 -0.005 0.067 0.16 -0.204 -0.116 0.006 0.142 0 -0.013 -0.156 0.077 0.023

2 1 0 -0.044 0 0 0.051 -0.33 -0.398 -0.02 0.06 0.14 -0.194 0.014 0.136 0.048 0.12 0.16 -0.204 -0.116 0.006 0.142 0 0 -0.156 0.077 0.023

2 1 0 -0.044 0 0 0.051 -0.33 -0.396 -0.02 0.06 0.125 -0.194 0.014 0.136 0.048 0.104 0.16 -0.204 -0.116 0.006 0.142 0 0 -0.156 0.077 0.023

2 1 0 -0.044 0 0 0.051 -0.33 -0.396 -0.02 0.06 0.125 -0.194 0 0.136 -0.005 -0.092 0.16 -0.204 -0.116 0.006 0.142 0 0 -0.156 0.077 0.023

2 1 0 -0.044 -0.061 0 0.051 -0.041 -0.356 -0.054 0.06 0.125 -0.278 0 0.136 -0.005 0.067 0.16 -0.185 -0.116 0.006 0.142 0 0 -0.156 0.077 0.023

2 1 0 -0.044 0 0 0.051 -0.121 -0.356 -0.054 0.06 0.125 -0.278 -0.062 0.136 -0.015 0.067 0.16 -0.204 -0.116 0.006 0.142 0 0 -0.156 0.077 0.042

2 1 0 -0.044 0 0 0.051 -0.121 -0.185 -0.054 0.06 0.125 -0.278 -0.062 0.136 -0.005 0.067 0.16 -0.204 -0.116 0.006 0.142 0 0 -0.156 0.077 0.042

2 1 0 -0.044 0 0 0.051 -0.121 -0.356 -0.054 0.06 0.125 -0.194 -0.052 0.274 -0.005 0.067 0.16 -0.204 -0.116 0.006 0.142 0 -0.013 -0.156 0.077 0.023

2 1 -0.007 -0.044 -0.061 0 0.051 -0.122 -0.356 -0.054 0.06 0.125 -0.278 0 0.136 -0.005 0.067 0.16 -0.185 -0.116 0.006 0.142 0 0 -0.156 0.077 0.023

2 1 0 -0.044 0 -0.07 0.051 -0.121 -0.356 -0.054 0.06 0.125 -0.194 0 0.274 -0.005 0.067 0.172 -0.204 -0.116 0.006 0.142 0 -0.013 -0.156 0.077 0.023

2 1 0 -0.044 0 0 0.051 -0.121 -0.356 -0.054 0.06 0.125 -0.194 0 0.262 -0.005 0.067 0.16 -0.204 -0.116 0.006 0.142 0 -0.013 -0.156 0.077 0.023

2 1 0.06 -0.044 0 0 -0.156 -0.121 -0.356 -0.054 0.06 0.125 -0.194 0 0.136 -0.005 0.067 0.176 -0.204 -0.116 0.006 0.142 0 -0.013 -0.156 0.077 0.023

2 1 0 -0.044 0 0 0.051 -0.33 -0.396 -0.156 0.06 0.125 -0.194 0 0.136 -0.005 0.067 0.16 -0.204 -0.116 0.006 0.142 0 0 -0.156 0.077 0.023

2 1 0 -0.044 0 0 0.051 -0.33 -0.396 -0.02 0.045 0.125 -0.194 0.014 0.136 0.048 0.12 0.16 -0.204 -0.116 0.006 0.142 0 0 -0.156 0.077 0.023

2 1 0 -0.044 0 0 0.051 -0.121 -0.356 -0.054 0.06 0.125 -0.194 0 0.274 -0.005 0.158 0.16 -0.204 -0.116 0.006 0.142 0 -0.013 -0.156 0.077 0.023

2 1 0 -0.044 -0.061 0 0.051 -0.122 -0.356 -0.054 0.06 0.125 -0.278 0 0.136 -0.005 0.067 0.16 -0.185 -0.116 -0.133 0.142 0 0 -0.156 0.077 0.023

2 1 0 -0.044 0 0 0.051 -0.33 -0.396 -0.02 0.06 0.125 -0.194 0 0.136 -0.005 0.067 0.16 -0.204 -0.116 0.246 0.142 0 0.128 -0.156 0.077 0.023

2 1 0 -0.044 0 0 0.051 -0.33 -0.396 -0.02 0.06 0.125 -0.194 0.014 0.136 0.048 0.12 0.16 -0.204 -0.109 0.006 0.142 0 0 -0.156 0.077 0.023

2 1 0 -0.044 -0.061 0 0.051 -0.122 -0.356 -0.054 0.06 0.125 -0.278 0 0.136 -0.005 0.067 0.16 -0.185 0.02 0.006 0.142 0 0 -0.156 0.077 0.023

2 1 0 -0.044 0 0 0.051 -0.33 -0.396 -0.02 0.06 0.125 -0.276 0 0.136 -0.005 0.067 0.16 -0.204 -0.116 0.006 0.142 0 0.128 -0.156 0.077 0.023

2 1 0 -0.044 0 0 0.051 -0.121 -0.356 -0.054 0.06 0.125 -0.278 0 0.136 -0.005 0.067 0.16 -0.185 -0.116 0.006 0.142 0 0 -0.156 0.077 -0.096

2 1 0 -0.044 0 0 0.051 -0.122 -0.356 -0.054 0.06 0.125 -0.278 0 0.136 -0.005 0.067 0.16 -0.185 -0.116 0.006 0.142 -0.04 0 -0.156 0.077 0.023

2 1 0 -0.044 0 0 0.051 -0.121 -0.356 -0.054 0.06 0.125 -0.278 -0.062 0.136 -0.005 0.067 0.16 -0.204 -0.116 -0.137 0.142 0 0 -0.156 0.077 0.042

2 1 0 -0.044 -0.061 0 0.051 -0.122 -0.356 -0.054 0.06 0.125 -0.278 0 0.136 -0.005 0.067 0.16 -0.185 -0.116 0.006 0.142 0 0.009 -0.156 0.077 0.023

2 1 0 -0.044 0 0 0.051 -0.33 -0.396 -0.02 0.06 0.125 -0.194 0.014 0.136 0.048 0.12 0.16 -0.204 -0.116 0.114 0.142 0 0 -0.156 0.077 0.023

2 1 0 -0.044 0 0 0.051 -0.33 -0.396 -0.02 0.06 0.125 -0.194 0 0.136 -0.005 0.067 0.109 -0.204 -0.116 0.006 0.142 0 0 -0.156 0.077 0.023

2 1 0 -0.044 -0.061 0 0.051 -0.122 -0.356 -0.054 0.06 0.125 -0.278 0 0.136 -0.005 0.067 0.16 -0.149 -0.116 0.006 0.142 0 0 -0.156 -0.09 0.023

2 1 0 -0.044 0 0 0.051 -0.33 -0.396 -0.02 0.06 0.125 -0.194 0.014 0.136 0.048 0.12 0.16 -0.204 -0.116 0.006 0.142 0 0 -0.156 0.079 0.023

3 1347 -0.045 0.023 0 0 -0.149 0 0 0.059 -0.09 0.112 0.029 0 0 0 0.024 0.063 -0.016 0.085 0.01 -0.152 -0.037 -0.139 0.031 -0.041 0

3 517 -0.045 0.023 0 0 -0.149 0 0 0.059 -0.09 0.112 0.029 0 0 0 0 0.063 -0.016 0.085 0.01 -0.152 -0.037 -0.139 0.066 -0.041 0

3 578 -0.045 0.023 0 0 -0.149 0 0 0.059 -0.09 0.112 0.029 0 0 0 0 0.063 -0.016 0.085 0.01 -0.152 -0.037 -0.139 0.031 -0.041 0

3 314 -0.045 0.023 0 0 -0.149 0 0 0.059 -0.09 0.112 0.029 0 0 0 0 0.063 0.083 0.085 0.01 -0.152 -0.037 -0.139 0.031 -0.041 0

3 253 -0.045 0.023 0 0 -0.149 0 0 0.059 -0.09 0.112 0.029 0 0.012 0 0 0.063 -0.016 0.085 0.01 -0.152 -0.037 -0.139 0.031 -0.041 0

3 246 -0.045 0.023 0 0 -0.149 0 0 0.059 -0.086 0.112 0.029 0 0 0 0 0.063 -0.016 0.085 0.01 -0.152 -0.012 -0.139 0.031 -0.031 0

3 164 -0.045 0.023 0 0 -0.149 -0.073 0 0.059 -0.09 0.112 0.029 0 0 0 0 0.063 -0.016 0.085 0.01 -0.152 -0.037 -0.139 0.031 -0.041 0

3 152 -0.045 0.003 0 0 -0.149 0 0 0.059 -0.09 0.112 0.029 0 0 0 0 0.063 -0.016 0.085 0.01 -0.152 -0.037 -0.139 0.031 -0.041 0

3 62 -0.045 0.023 0 0 -0.149 0 0 0.138 -0.09 0.112 0.029 0 0 0 0 0.063 -0.016 0.085 0.01 -0.152 -0.037 -0.139 0.031 -0.041 0

3 51 -0.045 0.003 0 0 0.015 0 0 0.059 -0.09 0.112 0.029 0 0 0 0 0.063 -0.016 0.085 0.01 -0.152 -0.037 -0.139 0.031 -0.041 0

3 18 -0.073 0.023 0 0 -0.149 0 0 0.059 -0.09 0.112 0.029 0 0 0 0 0.063 -0.016 0.085 0.01 -0.152 -0.037 -0.139 0.031 -0.041 0

3 18 0 0.023 0 0 -0.149 0 0 0.092 -0.09 0.112 0.029 0 0 0 0 0.063 -0.016 0.085 0.01 -0.152 -0.037 -0.139 0.066 -0.041 0

3 15 -0.045 0.023 0 0 -0.149 0 0 0.23 -0.09 0.112 0.029 0 0 0 0 0.063 -0.016 0.085 0.01 -0.152 -0.037 -0.139 0.066 -0.041 0

3 12 -0.045 0.023 0.111 0 -0.149 0 0 0.059 -0.09 0.112 0.029 0 0 0 0 0.063 -0.016 0.085 0.01 -0.152 -0.037 -0.139 0.066 -0.041 0

3 12 -0.045 0.023 0 0.049 -0.149 0 0 0.059 -0.09 0.112 0.029 0 0 0 0 0.063 -0.016 0.085 0.01 -0.152 -0.037 -0.139 0.031 -0.041 0

3 9 -0.045 0.023 0 0 -0.149 0 0 0.059 -0.09 0.112 0.029 0 0 0 0 0.063 -0.016 0.311 0.01 -0.152 -0.037 -0.139 0.031 -0.041 0

3 9 -0.045 0.023 0 0 -0.149 0 0 0.149 -0.086 0.112 0.029 0 0 0 0 0.063 -0.016 0.085 0.01 -0.152 -0.012 -0.139 0.031 -0.031 0

3 8 -0.045 0.023 0 0 -0.149 0 0 0.059 -0.09 0.112 0.029 0 0 0 0 0.063 -0.016 0.085 0.01 -0.261 -0.037 -0.139 0.031 -0.041 0

3 8 -0.045 0.023 0 0 -0.149 0 0 0.059 0.062 0.112 0.029 0 0 0 0.024 0.063 -0.016 0.085 0.01 -0.152 -0.037 -0.139 0.031 -0.041 0

3 8 -0.045 0.023 0 0 -0.149 0 0 0.059 -0.09 0.112 0.024 0 0 0 0 0.063 0.083 0.085 0.01 -0.152 -0.037 -0.139 0.031 -0.041 0

3 8 -0.045 0.023 0 0 -0.149 0 0 0.059 -0.09 0.112 0.029 0 0.037 0 0 0.063 0.083 0.085 0.01 -0.152 -0.037 -0.139 0.031 -0.041 0

3 7 0 0.023 0 0 -0.149 0 0 0.092 -0.09 0.112 0.029 0 0 0 0 0.063 -0.016 -0.036 0.01 -0.152 -0.037 -0.139 0.066 -0.041 0

3 6 -0.045 0.023 0 0 -0.149 0 0 0.059 -0.09 0.112 0.029 -0.214 0 0 0.024 0.063 -0.016 0.085 0.01 -0.152 -0.037 -0.139 0.031 -0.041 0

3 6 -0.045 0.003 0 0 -0.149 0 0 0.059 -0.09 0.112 0.029 0 0 0 0 0.063 -0.016 0.267 0.01 -0.152 -0.037 -0.139 0.031 -0.041 0

3 5 -0.045 0.023 0 0 -0.149 0 0 0.059 0.059 0.112 0.029 0 0 0 0 0.063 -0.016 0.085 0.01 -0.152 -0.037 -0.139 0.066 -0.041 0

3 5 -0.045 0.023 0.167 0 -0.149 0 0 0.059 -0.09 0.112 0.029 0 0 0 0 0.063 -0.016 0.085 0.01 -0.152 -0.037 -0.139 0.031 -0.041 0

3 5 -0.045 0.023 0 0 -0.149 0 0 0.059 -0.09 0.112 0.029 0 0 0 0 0.063 0.069 0.085 0.01 -0.152 -0.037 -0.139 0.031 -0.041 0

3 5 -0.045 0.023 0.118 0 -0.149 0 0 0.059 -0.09 0.112 0.029 0 0 0 0 0.063 -0.016 0.085 0.01 -0.152 -0.037 -0.139 0.031 -0.041 0.015

3 4 -0.045 0.011 0 0 -0.149 0 0 0.059 -0.09 0.112 0.029 0 0 0 0 0.063 -0.016 0.085 0.01 -0.152 -0.037 -0.139 0.066 -0.041 0

3 4 -0.045 0.023 0 0 -0.149 0 0 0.059 -0.086 0.195 0.029 0 0 0 0 0.063 -0.016 0.085 0.01 -0.152 -0.012 -0.139 0.031 -0.031 0

3 4 -0.045 0.023 0 0 -0.149 0 0 0.059 -0.09 0.112 0.029 0 0 0 0.024 0.063 -0.016 0.085 0.01 -0.152 -0.037 -0.051 0.031 -0.041 0

3 4 -0.045 0.023 0 0 -0.149 0 0 0.059 -0.09 0.112 -0.063 0 0.012 0 0 0.063 -0.016 0.085 0.01 -0.152 -0.037 -0.139 0.031 -0.041 0

3 4 -0.045 0.023 0 0 -0.149 0 0 -0.022 -0.09 0.112 0.029 0 0 0 0.024 0.063 -0.016 0.085 0.01 -0.152 -0.037 -0.139 0.031 -0.041 0

3 4 -0.045 0.023 0 0 -0.149 0 -0.126 0.059 -0.09 0.112 0.029 0 0 0 0 0.063 0.083 0.085 0.01 -0.152 -0.037 -0.139 0.031 -0.041 0

3 4 -0.045 0.003 0.051 0 0.015 0 0 0.059 -0.09 0.112 0.029 0 0 0 0 0.063 -0.016 0.085 0.01 -0.152 -0.037 -0.139 0.031 -0.041 0

3 4 -0.045 0.023 0 0 -0.149 0 -0.008 0.059 -0.09 0.112 0.029 0 0 0 0 0.063 -0.016 0.085 0.01 -0.152 -0.037 -0.139 0.066 -0.041 0

3 4 -0.045 0.023 0 0 -0.149 0 0 0.059 -0.09 0.112 0.029 0 0 0 0.024 0.063 -0.068 0.085 0.01 -0.152 -0.003 -0.139 0.031 -0.041 0

3 4 -0.045 0.023 0 0 -0.149 0 -0.187 0.059 -0.09 0.112 0.029 0 0 0 0.024 0.063 -0.016 0.085 0.01 -0.152 -0.037 -0.139 0.031 -0.041 0

3 4 -0.045 0.023 0 0 -0.149 0 0 0.059 -0.09 0.112 0.029 0 0 0.13 0 0.063 -0.016 0.085 0.01 -0.152 -0.037 -0.139 0.066 -0.041 0

3 4 -0.045 0.023 0 0 -0.149 0 0 0.059 -0.09 0.112 0.029 0 0 0 -0.028 0.063 0.083 0.085 0.01 -0.152 -0.037 -0.139 0.031 -0.041 0

3 4 -0.045 0.023 0 0 -0.149 0 -0.187 0.059 -0.09 0.206 0.029 0 0 0 0.024 0.063 -0.016 0.085 0.01 -0.152 -0.037 -0.139 0.031 -0.041 0

3 4 -0.045 0.023 0 0 -0.149 -0.073 0 0.059 -0.09 0.112 0.029 0 0 0 0 0.063 -0.016 0.116 0.01 -0.152 -0.037 -0.139 0.031 -0.041 0

3 4 -0.045 0.023 0 0 -0.149 0 0 0.059 -0.09 0.112 0.029 0 0 0 0 0.063 -0.016 0.085 0.01 -0.152 -0.037 -0.139 0.002 -0.041 0

3 3 -0.045 0.023 0 0 -0.149 0 0 0.059 -0.09 0.112 0.029 0 0 0 0 0.063 -0.016 0.085 0.01 -0.152 -0.037 -0.139 0.031 -0.19 0

3 3 -0.045 0.023 0 0 -0.149 0 0 0.059 -0.09 0.132 0.029 0 0 0 0.024 0.063 -0.016 0.085 0.01 -0.152 -0.037 -0.139 0.031 -0.041 0

3 3 -0.045 0.023 0 0 -0.149 0 0 0.059 -0.09 0.112 0.029 0 0 0 0.024 0.063 -0.068 0.085 0.01 -0.152 -0.037 -0.139 0.031 -0.041 0

3 3 -0.045 0.023 -0.055 0 -0.149 0 0 0.059 -0.09 0.112 0.029 0 0 0 0 0.063 -0.016 0.085 0.01 -0.152 -0.037 -0.139 0.066 -0.041 0

3 3 -0.045 0.023 0 0 -0.149 0 0 0.059 -0.09 0.112 0.029 0 0.012 0 0 0.063 -0.016 0.085 0.01 -0.152 -0.037 -0.139 0.031 -0.041 0.028

3 2 -0.045 0.023 0 0 -0.149 0 0 0.027 -0.09 0.112 0.029 0 0 0 0 0.063 -0.016 0.085 0.01 -0.152 -0.037 -0.139 0.066 -0.041 0

3 2 -0.045 0.023 0 0 -0.175 0 0 0.059 -0.09 0.112 0.029 0 0 0 0 0.063 0.083 0.085 0.01 -0.152 -0.037 -0.139 0.031 -0.041 0

3 2 -0.045 0.023 0 0 -0.149 0 0 0.059 -0.09 0.112 0.029 0 0 0 0 0.063 -0.135 0.085 0.01 -0.152 -0.037 -0.139 0.031 -0.041 0

3 2 -0.045 0.023 0 0 -0.149 0 0 -0.034 -0.09 0.112 0.029 0 0 0 0.024 0.063 -0.016 0.085 0.01 -0.152 -0.037 -0.139 0.031 -0.041 0

3 2 -0.045 0.023 0 0 -0.098 0 0 0.059 -0.09 0.112 0.029 0 0 0 0 0.063 0.083 0.085 0.01 -0.152 -0.037 -0.139 0.031 -0.041 0

3 2 -0.045 0.023 0.118 0 -0.149 0 0 0.059 -0.09 0.112 0.029 0 0 0 0 0.063 -0.016 0.085 0.01 -0.152 -0.037 -0.139 0.031 -0.041 0

3 2 -0.045 0.023 0 0 -0.149 -0.073 0 0.059 -0.09 0.112 0.029 0 0 0 0 0.063 -0.016 0.085 0.01 -0.152 -0.037 -0.139 0.031 -0.019 0

3 2 -0.045 0.023 0 0 -0.149 0 0 0.059 -0.09 0.112 0.029 0 0 -0.078 0.024 0.063 -0.016 0.085 0.01 -0.152 -0.037 -0.139 0.031 -0.041 0

3 2 -0.045 0.023 0 0 -0.149 0 0 0.059 -0.09 0.112 0.029 0 0.035 0 0 0.063 -0.016 0.085 0.01 -0.152 -0.037 -0.139 0.031 -0.041 0

3 2 -0.045 0.023 0 0 -0.149 0 0 0.059 -0.09 0.112 0.029 0 0.012 0 0 0.063 -0.016 0.085 0.01 -0.152 -0.057 -0.139 0.031 -0.041 0

3 2 -0.045 0.023 0 0 -0.149 0 0 0.059 -0.09 0.112 0.029 0 0 0 0 0.063 -0.016 0.085 0.01 -0.152 -0.037 0.001 0.031 -0.041 0

3 2 -0.045 0.023 0 0 -0.127 0 0 0.059 -0.09 0.112 0.029 0 0 0 0.024 0.063 -0.016 0.085 0.01 -0.152 -0.037 -0.139 0.031 -0.041 0

3 2 -0.045 0.023 0.012 -0.016 -0.149 0 0 0.059 -0.09 0.112 0.029 0 0 0 0 0.063 -0.016 0.085 0.01 -0.152 -0.037 -0.139 0.066 -0.041 0

3 2 -0.045 0.023 0 0 -0.149 0 0 0.059 -0.09 0.112 0.029 0 0 0 0.107 0.063 -0.016 0.085 0.01 -0.152 -0.037 -0.139 0.031 -0.041 0

3 2 -0.045 0.023 0 0 -0.149 0 -0.249 0.059 -0.09 0.112 0.029 0 0 0 0 0.063 -0.016 0.085 0.01 -0.152 -0.037 -0.139 0.031 -0.041 0

3 2 -0.085 0.023 0 0 -0.149 0 0 0.059 -0.09 0.112 0.029 0 0 0 0.024 0.063 -0.016 0.085 0.01 -0.152 -0.037 -0.139 0.031 -0.041 0

3 2 -0.045 0.023 0 0 -0.149 0 0 0.059 -0.09 0.112 0.029 0 0 0 0.024 0.063 -0.016 -0.007 0.01 -0.152 -0.037 -0.139 0.031 -0.041 0

3 2 -0.045 0.023 0 0 -0.149 0 0 0.059 -0.09 0.112 0.029 0 0 0 0.024 0.063 -0.016 0.085 -0.006 -0.152 -0.037 -0.139 0.031 -0.041 0

3 2 -0.045 0.023 -0.034 0 -0.149 0 0 0.059 -0.09 0.132 0.029 0 0 0 0.024 0.063 -0.016 0.085 0.01 -0.152 -0.037 -0.139 0.031 -0.041 0

3 2 -0.045 0.003 0 0 -0.149 0 0 0.059 -0.09 0.112 0.029 0 0 0 0 0.063 -0.016 0.085 -0.063 -0.152 -0.037 -0.139 0.031 -0.041 0

3 2 -0.045 0.023 0 0 -0.149 -0.073 0 0.059 -0.09 0.112 0.029 0 0 0 0 0.063 -0.076 0.085 0.01 -0.152 -0.037 -0.139 0.031 -0.041 0

3 2 -0.045 0.023 0 0 -0.149 -0.073 0 0.059 -0.09 0.112 0.029 0 -0.063 0 0 0.063 -0.016 0.085 0.01 -0.152 -0.037 -0.139 0.031 -0.041 0

3 2 -0.045 0.023 -0.125 0 -0.149 0 0 0.059 -0.09 0.112 0.029 0 0 0 0 0.063 -0.016 0.085 0.01 -0.152 -0.037 -0.139 0.066 -0.041 0

3 2 -0.045 0.023 0 0 -0.149 0 0 0.042 -0.09 0.112 0.029 0 0 0 0 0.063 0.083 0.085 0.01 -0.152 -0.037 -0.139 0.031 -0.041 0

3 2 -0.045 -0.114 0 0 -0.149 0 0 0.059 -0.086 0.112 0.029 0 0 0 0 0.063 -0.016 0.085 0.01 -0.152 -0.012 -0.139 0.031 -0.031 0

3 2 -0.045 -0.108 0 0 -0.149 0 0 0.059 -0.09 0.112 0.029 0 0 0 0.024 0.063 -0.016 0.085 0.01 -0.152 -0.037 -0.139 0.031 -0.041 0

3 2 -0.045 0.023 0 0 -0.149 0 0 0.059 -0.09 0.112 0.029 0 0 0 0.024 0.063 -0.016 0.085 0.097 -0.152 -0.037 -0.139 0.031 -0.041 0

3 2 -0.045 0.023 0 0 -0.149 0 0 0.059 -0.09 0.112 0.029 0 0 0 0.046 0.063 -0.016 0.085 0.01 -0.152 -0.037 -0.139 0.031 -0.041 0

3 2 -0.045 0.023 0 0 -0.149 0 0 0.059 -0.09 0.112 0.029 0 0 0 0 0.063 -0.016 0.085 0.01 -0.152 -0.037 -0.139 0.066 -0.041 -0.112

3 2 -0.045 0.003 0 0 0.015 0 0 0.059 -0.09 0.112 0.029 0 0 0 0 0.063 -0.016 0.085 0.01 -0.152 -0.037 -0.139 0.031 -0.041 0.209

3 2 -0.045 0.023 0 0 -0.149 0 0 0.059 -0.09 0.112 0.029 0 0 0 0.024 0.063 -0.016 0.085 0.01 -0.152 -0.037 -0.139 0.109 -0.041 0

3 2 -0.045 0.023 0 0 -0.149 0 0 0.059 -0.09 0.112 0.029 0 0 0 0 0.063 0.03 0.085 0.01 -0.152 -0.037 -0.139 0.031 -0.041 0

3 2 -0.048 0.023 0 0 -0.149 0 0 0.059 -0.086 0.112 0.029 0 0 0 0 0.063 -0.016 0.085 0.01 -0.152 -0.012 -0.139 0.031 -0.031 0

3 2 -0.045 0.023 0 0 -0.149 0 0 0.059 -0.09 0.112 0.029 0.12 0 0 0.024 0.063 -0.016 0.085 0.01 -0.152 -0.037 -0.139 0.031 -0.041 0

3 2 -0.045 0.023 0 0 -0.149 0 0 0.059 -0.09 0.112 0.029 -0.143 0 0 0.024 0.063 -0.016 0.085 0.01 -0.152 -0.037 -0.139 0.031 -0.041 0

3 2 -0.045 -0.114 0 0 -0.149 0 0 0.059 0.006 0.112 0.029 0 0 0 0 0.063 -0.016 0.085 0.01 -0.152 -0.012 -0.139 0.031 -0.031 0

3 2 -0.045 0.023 0 0 -0.149 0 0 0.059 -0.09 0.112 0.029 0 0.053 0 0.024 0.063 -0.016 0.085 0.01 -0.152 -0.037 -0.139 0.031 -0.041 0

3 1 -0.045 0.023 0 0 -0.149 0 0 0.059 -0.09 0.112 0.029 0 0 0 0.024 0.063 -0.016 0.085 0.01 -0.152 -0.037 -0.139 0.031 -0.041 -0.187

3 1 -0.045 0.023 0.083 0 -0.149 0 0 0.059 -0.09 0.112 0.029 0 0 0 0 0.063 -0.016 0.085 0.01 -0.152 -0.037 -0.139 0.031 -0.041 0

3 1 -0.045 0.023 0 0 -0.149 0 0 0.059 -0.09 0.112 0.029 -0.005 0 0 0 0.063 -0.016 0.085 0.01 -0.152 -0.037 -0.139 0.066 -0.041 0

3 1 -0.045 0.023 0 0 -0.149 0 0 0.059 -0.09 0.112 0.029 0 0 0 0 0.063 0.083 0.085 0.01 -0.152 -0.129 -0.139 0.031 -0.041 0

3 1 0.006 0.023 0 0 -0.149 -0.073 0 0.059 -0.09 0.112 0.029 0 0 0 0 0.063 -0.016 0.085 0.01 -0.152 -0.037 -0.139 0.031 -0.041 0

3 1 -0.045 0.023 0 0 -0.149 0 0 0.059 -0.09 0.112 -0.209 0 0 0 0 0.063 -0.016 0.085 0.01 -0.152 -0.037 -0.139 0.066 -0.041 0

3 1 -0.045 0.023 0 0 -0.149 0 0 0.059 -0.09 0.112 0.029 0 0 0 0.024 0.063 -0.016 0.156 0.01 -0.152 -0.037 -0.139 0.031 -0.041 0

3 1 -0.045 0.023 0 0 -0.149 0 0 0.059 -0.09 0.112 0.029 0 0 0 0 0.063 0.083 0.085 0.01 -0.152 -0.037 -0.139 0.031 0.015 0

3 1 -0.045 0.023 0 0 -0.149 0 0 0.097 -0.09 0.112 0.029 0 0 0 0 0.063 -0.016 0.085 0.01 -0.152 -0.037 -0.139 0.066 -0.041 0

3 1 -0.045 0.023 0 0 -0.149 0 0.099 0.059 -0.09 0.112 0.029 0 0.012 0 0 0.063 -0.016 0.085 0.01 -0.152 -0.037 -0.139 0.031 -0.041 0

3 1 -0.045 0.023 0 0 -0.149 0 0 0.059 -0.09 0.112 0.029 0 0 0 0 0.068 -0.016 0.085 0.01 -0.152 -0.037 -0.139 0.066 -0.041 0

3 1 -0.045 0.023 0 0 -0.149 0 0 0.059 -0.09 0.112 0.029 0 0 0 0 0.063 0.083 0.099 0.01 -0.152 -0.037 -0.139 0.031 -0.041 0

3 1 -0.045 0.023 0 0 -0.149 0 0 0.059 -0.09 0.112 0.029 0 0.012 0 -0.016 0.063 -0.016 0.085 0.01 -0.152 -0.037 -0.139 0.031 -0.041 0

3 1 -0.045 0.023 0 0 -0.149 0 0 0.059 -0.09 0.112 0.029 0 0 0 0 0.063 0.083 0.085 0.01 -0.152 -0.173 -0.139 0.031 -0.041 0

3 1 -0.045 0.023 0 0 -0.149 0 0 0.059 -0.09 0.112 -0.071 0 0 0 0.024 0.063 -0.016 0.085 0.01 -0.152 -0.037 -0.139 0.031 -0.041 0

3 1 -0.045 0.023 0 0 -0.149 -0.073 0 -0.031 -0.09 0.112 0.029 0 0 0 0 0.063 -0.016 0.085 0.01 -0.152 -0.037 -0.139 0.031 -0.041 0

3 1 -0.045 0.003 0 0 0.015 0 0 0.059 -0.09 0.112 0.029 0 0 0 0 0.063 -0.016 0.085 0.01 -0.152 -0.037 -0.139 0.031 -0.156 0

3 1 -0.045 0.023 0 0 -0.149 0.097 0 0.059 -0.09 0.112 0.029 0 0 0 0 0.063 -0.016 0.311 0.01 -0.152 -0.037 -0.139 0.031 -0.041 0

3 1 -0.045 0.023 0 0 -0.149 0 0 0.059 -0.086 0.112 0.029 0 0 0 0 0.063 -0.124 0.085 0.01 -0.152 -0.012 -0.139 0.031 -0.031 0

3 1 -0.045 0.023 0 0 -0.149 0 0 0.059 -0.09 0.112 0.029 0 0 0 -0.036 0.063 -0.016 0.085 0.01 -0.152 -0.037 -0.139 0.031 -0.041 0

3 1 -0.045 0.023 0.111 0 -0.149 0 0 0.059 -0.09 0.112 0.029 0 0 0 0 0.175 -0.016 0.085 0.01 -0.152 -0.037 -0.139 0.066 -0.041 0

3 1 -0.045 0.023 0 0 -0.149 0 0 0.059 -0.09 0.112 0.029 0.185 0 0 0 0.063 -0.016 0.085 0.01 -0.152 -0.037 -0.139 0.031 -0.041 0

3 1 -0.045 0.023 0 0 -0.149 0 0 0.059 -0.09 0.112 0.029 0 0 0 0.024 0.063 -0.016 0.203 0.01 -0.152 -0.037 -0.139 0.031 -0.041 0

3 1 -0.045 0.023 0 -0.125 -0.149 0 0 0.059 -0.09 0.112 0.029 0 0 0 0.024 0.063 -0.016 0.085 0.01 -0.152 -0.037 -0.139 0.031 -0.041 0

3 1 -0.045 0.023 0 0 -0.149 0 0 0.059 -0.09 0.112 0.029 0 0 0 0.024 0.063 -0.016 0.085 0.01 -0.152 -0.037 -0.139 0.048 -0.041 0

3 1 -0.045 0.023 0 0 -0.149 0 0 0.059 -0.09 0.112 0.029 0 0 0 -0.041 0.063 -0.016 0.085 0.01 -0.152 -0.037 -0.139 0.031 -0.041 0

3 1 -0.045 0.023 0 0 -0.149 0.058 0 0.059 -0.09 0.112 0.029 0 0.012 0 0 0.063 -0.016 0.085 0.01 -0.152 -0.037 -0.139 0.031 -0.041 0

3 1 -0.045 0.023 0 0 -0.237 0 0 0.059 -0.09 0.112 0.029 0 0 0 0 0.063 -0.016 0.085 0.01 -0.152 -0.037 -0.139 0.066 -0.041 0

3 1 -0.045 0.023 -0.148 0 -0.149 0 0 0.059 -0.09 0.112 0.029 0 0 0 0 0.063 -0.016 0.085 0.01 -0.152 -0.037 -0.139 0.066 -0.041 0

3 1 -0.045 -0.087 0 0 -0.149 0 0 0.059 -0.09 0.112 0.029 0 0 0 0 0.063 -0.016 0.085 0.01 -0.152 -0.037 -0.139 0.031 -0.041 0

3 1 -0.045 0.084 0 0 -0.149 0 0 0.059 -0.09 0.112 0.029 0 0 0 0.024 0.063 -0.016 0.085 0.01 -0.152 -0.037 -0.139 0.031 -0.041 0

3 1 -0.045 0.023 0 0 -0.149 0 0.01 0.059 -0.09 0.112 0.029 0 0 0 0.024 0.063 -0.016 0.085 0.01 -0.152 -0.037 -0.139 0.031 -0.041 0

3 1 -0.045 0.023 0 0 -0.149 0 0 0.059 -0.09 0.112 0.029 0 0 0 0 0.072 -0.016 0.085 0.01 -0.152 -0.037 -0.139 0.031 -0.041 0

3 1 -0.045 0.023 0 0 -0.149 0 0 0.059 -0.09 0.112 0.029 0 0.012 0 0 0.063 -0.016 0.044 0.01 -0.152 -0.037 -0.139 0.031 -0.041 0

3 1 -0.045 0.023 0 0 -0.149 0 0 0.059 -0.148 0.112 0.029 0 0 0 0.024 0.063 -0.016 0.085 0.01 -0.152 -0.037 -0.139 0.031 -0.041 0

3 1 -0.045 0.023 0 0 -0.149 -0.073 0 0.059 -0.09 0.112 0.029 0 0 0 0 0.063 -0.016 0.085 0.01 -0.152 -0.037 -0.139 0.163 -0.041 0

3 1 -0.045 0.023 0 0 -0.149 0 0 0.138 -0.09 0.112 0.029 0 0 0 0 0.063 -0.016 0.085 0.01 -0.152 -0.208 -0.139 0.031 -0.041 0

3 1 -0.045 0.023 0 0 -0.149 0 -0.044 0.059 -0.09 0.112 0.024 0 0 0 0 0.063 0.083 0.085 0.01 -0.152 -0.037 -0.139 0.031 -0.041 0

3 1 -0.045 0.023 0 0 -0.274 0 0 0.059 -0.09 0.112 0.029 0 0 0 0 0.063 -0.016 0.085 0.01 -0.152 -0.037 -0.139 0.031 -0.041 0

3 1 -0.045 0.023 0 0 -0.149 0 0 0.059 -0.09 0.112 0.029 0 -0.088 0 0 0.063 0.083 0.085 0.01 -0.152 -0.037 -0.139 0.031 -0.041 0

3 1 -0.045 0.023 0.219 0 -0.149 0 0 0.059 -0.09 0.112 0.029 0 0 0 0 0.063 -0.016 0.085 0.01 -0.152 -0.037 -0.139 0.066 -0.041 0

3 1 -0.045 0.003 0 0 0.015 0 0 0.059 -0.09 0.112 -0.142 0 0 0 0 0.063 -0.016 0.085 0.01 -0.152 -0.037 -0.139 0.031 -0.041 0

3 1 -0.045 0.023 0 0 -0.149 0 0 0.059 -0.09 0.112 0.029 0 0 0 0.024 0.063 -0.016 0.085 0.01 -0.152 -0.037 -0.139 0.031 -0.041 0.055

3 1 -0.045 0.023 0 0 -0.149 0 0 0.059 -0.09 0.112 0.029 0 0 0 0 0.063 -0.016 0.085 0.01 -0.278 -0.037 -0.139 0.031 -0.041 0

3 1 -0.045 0.003 0 0 -0.149 0 0 0.059 -0.09 0.112 0.019 0 0 0 0 0.063 -0.016 0.085 0.01 -0.152 -0.037 -0.139 0.031 -0.041 0

3 1 -0.045 0.023 0 0 -0.149 0 0 0.059 -0.09 0.112 0.029 0 0 0 0 0.063 0.053 0.085 0.01 -0.152 -0.037 -0.139 0.066 -0.041 0

3 1 -0.045 0.023 0 0 -0.149 0 0 0.059 -0.09 0.298 0.029 0 0 0 0.024 0.063 -0.016 0.085 0.01 -0.152 -0.037 -0.139 0.031 -0.041 0

3 1 -0.045 0.023 0 0 -0.149 0 0 0.059 -0.09 0.112 0.029 -0.086 0 0 0.024 0.063 -0.016 0.085 0.01 -0.152 -0.037 -0.139 0.031 -0.041 0

3 1 -0.045 0.023 0 0 -0.149 0 0 0.059 -0.09 0.112 0.029 0 0 0 0 0.063 -0.016 0.085 0.01 -0.152 0.039 -0.139 0.031 -0.041 0

3 1 -0.045 0.023 0 0 -0.149 0 0 0.059 -0.09 0.112 0.029 0 0 0 0 0.063 0.013 0.085 0.01 -0.152 -0.037 -0.139 0.031 -0.041 0

3 1 -0.045 0.023 0 0 -0.149 0 0 0.059 -0.09 0.112 0.029 0 0 0 0.024 0.063 -0.016 0.085 0.01 -0.152 -0.037 -0.139 0.031 -0.041 -0.124

3 1 -0.045 0.023 0 0 -0.149 0 0 0.059 -0.09 0.112 0.029 0 0 0 0.024 0.063 -0.016 0.085 0.01 -0.152 -0.037 -0.139 0.031 -0.026 0

3 1 -0.045 0.023 0 0 -0.149 0 0 0.059 -0.09 0.112 0.029 -0.083 0 0 0 0.063 0.083 0.085 0.01 -0.152 -0.037 -0.139 0.031 -0.041 0

3 1 -0.045 0.023 0 0 -0.149 0 0 0.059 -0.09 0.112 0.029 0 0 0 0 0.063 -0.016 0.085 0.01 -0.152 -0.037 -0.139 0.066 -0.041 -0.328

3 1 0.165 0.023 0 0 -0.149 0 0 0.059 -0.09 0.112 0.029 0 0 0 0 0.063 -0.016 0.085 0.01 -0.152 -0.037 -0.139 0.031 -0.041 0

3 1 -0.045 0.023 0 0 -0.149 0 0 0.117 -0.09 0.112 0.029 0 0 0 0.024 0.063 -0.016 0.085 0.01 -0.152 -0.037 -0.139 0.031 -0.041 0

3 1 -0.045 0.023 0 0 -0.149 0 -0.126 0.059 -0.09 0.112 0.029 0 0 0 0 0.063 0.083 0.085 0.01 -0.152 -0.037 -0.293 0.1 -0.041 0

3 1 -0.045 0.023 0 0 -0.149 0 0 0.059 -0.09 0.112 0.029 0 0 0 0.024 0.063 -0.016 0.085 0.01 -0.123 -0.037 -0.139 0.031 -0.041 0

3 1 -0.045 0.023 0 0 -0.149 0 0 0.059 -0.09 0.112 0.029 0 0 0 0 0.063 -0.016 0.085 0.01 -0.152 -0.037 -0.139 0.066 -0.153 0

3 1 -0.045 0.023 0 0 -0.113 0 0 0.059 -0.09 0.112 0.029 0 0 0 0.024 0.063 -0.016 0.085 0.01 -0.152 -0.037 -0.139 0.031 -0.041 0

3 1 -0.045 0.023 0 0 -0.149 0 0 0.059 -0.09 0.112 0.029 0 0 0.338 0 0.063 -0.016 0.085 0.01 -0.152 -0.037 -0.139 0.031 -0.041 0

3 1 -0.045 0.023 0 0 -0.149 0 0 0.059 -0.09 0.112 0.054 0 0 0 0.024 0.063 -0.016 0.085 0.01 -0.152 -0.037 -0.139 0.031 -0.041 0

3 1 -0.045 0.023 0 -0.072 -0.149 0 0 0.059 -0.09 0.112 0.029 0 0 0 0.024 0.063 -0.016 0.085 0.01 -0.152 -0.037 -0.139 0.031 -0.041 0

3 1 -0.045 0.023 0 0 -0.149 0 0 0.059 -0.09 0.112 0.029 0 0 0 0.024 0.063 -0.016 0.085 0.01 -0.152 -0.037 -0.039 0.031 -0.041 0

3 1 -0.045 0.023 0 0 -0.149 0 0 0.059 -0.124 0.112 0.029 0 0 0 0 0.063 -0.016 0.085 0.01 -0.152 -0.037 -0.139 0.031 -0.041 0

3 1 -0.045 0.023 0 0 -0.149 0 0 0.059 -0.086 0.112 0.029 0 0 0 0 -0.087 -0.016 0.085 0.01 -0.152 -0.012 -0.139 0.031 -0.031 0

4 646 -0.044 0.221 0.202 0 0.132 -0.075 0 0.117 -0.218 0.083 -0.051 0 0.111 -0.124 0 0.058 0.027 0.163 0.085 -0.054 -0.091 0 0 -0.004 0

4 535 -0.044 0.221 0.202 0 0.132 -0.075 0 0.117 -0.218 0.083 -0.051 0 0.111 -0.113 0 0.058 0 0.163 0.126 -0.054 -0.091 0 0 -0.004 0

4 455 -0.044 0.137 0.202 0 0.132 -0.075 0 0.117 -0.218 0.083 -0.051 0 0.111 -0.124 0 0.058 0 0.163 0.085 -0.054 -0.091 0 0 -0.031 0

4 439 -0.044 0.137 0.181 0 0.191 -0.075 0 0 -0.215 0.083 -0.051 0 0.111 -0.114 0 0 0 0.163 0.009 -0.007 -0.091 0 0.388 0.013 0

4 216 -0.044 0.221 0.202 0 0.132 -0.075 0 0.117 -0.218 0.083 -0.051 0 0.111 -0.113 0 0.058 0 0.163 0.126 -0.054 -0.091 0 0 -0.156 0

4 155 -0.044 0.221 0.202 0 0.132 -0.075 0 0.117 -0.218 0.083 -0.051 -0.044 0.111 -0.113 0 0.058 0 0.163 0.085 -0.054 -0.091 0 0.01 -0.004 0

4 135 -0.044 0.137 0.187 0 0.191 -0.075 0 0 -0.215 0.083 -0.051 0 0.111 -0.114 0 0 0 0.163 0.009 -0.007 -0.091 0 0.388 0.013 0

4 89 -0.044 0.221 0.202 0 0.132 -0.075 0 0.117 -0.218 0.083 -0.051 0 0.111 -0.113 0 0.058 0 0.073 0.085 -0.054 -0.091 0 0 -0.129 0

4 69 -0.044 0.221 0.202 0 0.132 -0.075 0 0.117 -0.218 0.083 -0.051 0 0.111 -0.113 0 0.058 0 0.163 0.085 -0.054 -0.091 0 0 -0.129 0

4 64 -0.044 0.119 0.181 0 0.191 -0.075 0 0 -0.215 0.083 -0.051 0 0.111 -0.114 0 0 0 0.129 0.009 -0.007 -0.091 0 0.388 0.013 0

4 52 -0.044 0.137 0.187 0 0.191 -0.075 0 0 -0.215 0.083 -0.051 0 0.111 -0.078 0 0 0 0.163 0.009 -0.007 -0.091 0 0.388 0.013 0

4 44 -0.044 0.137 0.181 0 0.191 -0.075 0 0 -0.215 0.344 -0.051 0 0.111 -0.114 0 0 0 0.163 0.009 -0.007 -0.091 0 0.388 0.013 0

4 31 -0.044 0.221 0.202 0 0.132 -0.075 0 0.117 -0.218 0.083 -0.051 0 0.111 -0.113 0 0.058 0 0.163 0.085 -0.054 -0.091 0 0 -0.145 0

4 27 -0.044 0.221 0.202 -0.038 0.132 -0.075 0 0.117 -0.218 0.083 -0.051 0 0.111 -0.124 0 0.058 0.027 0.163 0.085 -0.054 -0.091 0 0 -0.004 0

4 25 -0.044 0.137 0.181 0 0.191 -0.075 0 0 -0.215 0.083 -0.051 -0.18 0.111 -0.114 0 0 0 0.163 0.009 -0.007 -0.091 0 0.388 0.013 0

4 23 -0.044 0.221 0.202 0 0.132 -0.075 0 0.117 -0.172 0.083 -0.051 0 0.111 -0.124 0 0.058 0.027 0.163 0.085 -0.054 -0.091 0 0 -0.004 0

4 22 -0.044 0.119 0.181 0 0.191 -0.019 0 0 -0.215 0.083 -0.051 0 0.111 -0.114 0 0 0 0.129 0.009 -0.007 -0.091 0 0.388 0.013 0

4 21 -0.044 0.221 0.202 0 0.132 -0.075 0 0.135 -0.218 0.083 -0.051 0 0.111 -0.113 0 0.058 0 0.163 0.126 -0.054 -0.091 0 0 -0.004 0

4 18 -0.044 0.137 0.181 0 0.191 -0.075 0 0 -0.215 0.222 -0.051 0 0.111 -0.114 0 0 0 0.163 0.009 -0.007 -0.091 0 0.388 0.013 0

4 18 -0.044 0.221 0.202 0 0.132 -0.075 0 0.117 -0.218 0.083 -0.051 0 0.111 -0.113 -0.158 0.058 0 0.163 0.126 -0.054 -0.091 0 0 -0.156 0

4 14 -0.044 0.221 0.202 0 0.132 -0.075 0 0.117 -0.218 0.083 -0.051 0 0.111 -0.113 0 0.058 0 0.163 0.128 -0.054 -0.091 0 0 -0.004 0

4 11 -0.044 0.091 0.202 0 0.132 -0.075 0 0.117 -0.218 0.083 -0.051 0 0.111 -0.113 0 0.058 0 0.163 0.126 -0.054 -0.091 0 0 -0.004 0

4 11 -0.044 0.221 0.202 0 0.132 -0.075 0 0.117 -0.218 0.083 -0.051 0 0.111 -0.124 0 0.058 0.027 0.058 0.085 -0.054 -0.091 0 0 -0.004 0

4 10 -0.044 0.137 0.181 0 0.191 -0.075 0 0 -0.215 0.083 -0.086 0 0.111 -0.114 0 0 0 0.163 0.009 -0.007 -0.091 0 0.388 0.013 0

4 10 -0.044 0.221 0.202 0 0.132 -0.075 0 0.117 -0.218 0.083 -0.051 0 0.111 -0.113 0 0.058 -0.019 0.163 0.126 -0.054 -0.091 0 0 -0.004 0

4 9 -0.044 0.221 0.202 0 0.132 -0.075 0 0.117 -0.218 0.083 -0.051 0 0.111 -0.113 0 0.058 -0.047 0.073 0.085 -0.054 -0.091 0 0 -0.129 0

4 8 -0.044 0.137 0.181 0 0.191 -0.075 0 0 -0.215 0.083 -0.051 -0.084 0.111 -0.114 0 0 0 0.163 0.009 -0.007 -0.091 0 0.388 0.013 0

4 8 -0.044 0.221 0.202 0 0.132 -0.075 0 0.071 -0.218 0.083 -0.051 -0.044 0.111 -0.113 0 0.058 0 0.163 0.085 -0.054 -0.091 0 0.01 -0.004 0

4 7 -0.044 0.221 0.202 0 0.132 -0.075 0 0.135 -0.298 0.083 -0.051 0 0.111 -0.113 0 0.058 0 0.163 0.126 -0.054 -0.091 0 0 -0.004 0

4 7 -0.044 0.221 0.202 0 0.132 -0.075 0 0.117 -0.218 0.083 -0.155 -0.044 0.111 -0.113 0 0.058 0 0.163 0.085 -0.054 -0.091 0 0.01 -0.004 0

4 6 -0.044 0.137 0.181 0 0.191 -0.075 0 0 -0.215 0.083 -0.051 0 0.111 -0.114 0 0 0 0.163 0.009 -0.007 -0.091 0 0.388 -0.126 0

4 6 -0.044 0.137 0.202 0 0.132 -0.075 0 0.133 -0.218 0.083 -0.051 0 0.111 -0.124 0 0.058 0 0.163 0.085 -0.054 -0.091 0 0 -0.031 0

4 5 -0.044 0.137 0.181 0 0.191 -0.075 0 0 -0.215 0.222 -0.051 0 0.111 -0.114 0 0 0 0.094 0.009 -0.007 -0.091 0 0.388 0.013 0

4 5 -0.044 0.137 0.181 0 0.191 -0.075 -0.095 0 -0.215 0.083 -0.051 -0.084 0.111 -0.114 0 0 0 0.163 0.009 -0.007 -0.091 0 0.388 0.013 0

4 5 -0.044 0.137 0.202 0 0.132 -0.075 0 0.117 -0.218 0.083 -0.051 0 0.111 -0.124 0 0.058 0 0.163 0.085 -0.054 -0.091 0 0.168 -0.031 0

4 5 -0.044 0.221 0.202 0 0.132 -0.075 0 0.117 -0.191 0.083 -0.051 0 0.111 -0.113 0 0.058 0 0.163 0.126 -0.054 -0.091 0 0 -0.004 0

4 4 -0.044 0.119 0.181 0 0.191 -0.075 0 0 -0.215 0.083 -0.051 0 0.111 -0.114 0 0 0 0.163 0.009 -0.007 -0.091 0 0.388 0.013 0

4 4 -0.044 0.201 0.202 0 0.132 -0.075 0 0.117 -0.218 0.083 -0.051 0 0.111 -0.124 0 0.058 0.027 0.163 0.085 -0.054 -0.091 0 0 -0.004 0

4 4 -0.044 0.137 0.181 0 0.191 -0.075 0 0 -0.215 0.083 -0.051 0 0.111 -0.114 0 0 0.028 0.163 0.009 -0.007 -0.091 0 0.388 0.013 0

4 4 -0.044 0.137 0.181 0 0.191 -0.075 0 0 -0.215 0.083 -0.051 0 0.111 0.032 0 0 0 0.163 0.009 -0.007 -0.091 0 0.388 0.013 0

4 4 -0.044 0.221 0.202 0 0.132 -0.075 0 0.117 -0.218 0.009 -0.051 0 0.111 -0.113 0 0.058 0 0.163 0.085 -0.054 -0.091 0 0 -0.129 0

4 4 -0.044 0.119 0.181 0.003 0.191 -0.075 0 0 -0.215 0.083 -0.051 0 0.111 -0.114 0 0 0 0.129 0.009 -0.007 -0.091 0 0.388 0.013 0

4 4 -0.044 0.221 0.202 0 0.132 -0.075 -0.211 0.117 -0.218 0.083 -0.051 0 0.111 -0.124 0 0.058 0.027 0.163 0.085 -0.054 -0.091 0 0 -0.004 0

4 4 -0.044 0.221 0.202 0 0.132 -0.075 0 0.117 -0.218 0.083 -0.051 -0.044 0.111 -0.113 0 0.058 0 0.163 0.085 -0.054 -0.091 0 0.01 -0.004 0.131

4 4 -0.044 0.137 0.202 0 0.132 -0.075 0 0.082 -0.218 0.083 -0.051 0 0.111 -0.124 0 0.058 0 0.163 0.085 -0.054 -0.091 0 0 -0.031 0

4 4 -0.044 0.221 0.202 0 0.132 -0.075 0 0.117 -0.218 0.083 -0.051 -0.175 0.111 -0.124 0 0.058 0.027 0.163 0.085 -0.054 -0.091 0 0 -0.004 0

4 4 -0.044 0.137 0.181 0 0.191 -0.075 0 0 -0.018 0.083 -0.051 0 0.111 -0.114 0 0 0 0.163 0.009 -0.007 -0.091 0 0.388 0.013 0

4 3 -0.044 0.221 0.202 0 0.132 -0.075 0 0.117 -0.063 0.083 -0.051 0 0.111 -0.124 0 0.058 0.027 0.163 0.085 -0.054 -0.091 0 0 -0.004 0

4 3 -0.044 0.221 0.202 0 0.132 -0.075 0 0.117 -0.218 0.083 -0.051 0 0.111 -0.124 0 -0.029 0.027 0.163 0.085 -0.054 -0.091 0 0 -0.004 0

4 3 -0.044 0.137 0.181 0 0.191 -0.068 0 0 -0.215 0.083 -0.051 0 0.111 -0.114 0 0 0 0.163 0.009 -0.007 -0.091 0 0.388 0.013 0

4 3 -0.044 0.221 0.202 0 0.132 -0.075 0 0.117 -0.218 0.083 -0.051 0 0.111 -0.113 0 0.058 0 0.163 0.126 -0.054 -0.091 0 -0.063 -0.156 0

4 3 -0.044 0.23 0.181 0 0.191 -0.075 0 0 -0.215 0.083 -0.051 0 0.111 -0.114 0 0 0 0.163 0.009 -0.007 -0.091 0 0.388 0.013 0

4 2 -0.044 0.137 0.181 0 0.191 -0.075 0 0 -0.215 0.083 -0.051 -0.099 0.111 -0.114 0 0 0 0.163 0.009 -0.007 -0.091 0 0.388 0.013 0

4 2 -0.044 0.137 0.202 0 0.132 -0.075 0 0.117 -0.218 0.083 -0.051 0 0.111 -0.124 0 0.042 0 0.163 0.085 -0.054 -0.091 0 0 -0.031 0

4 2 -0.044 0.303 0.202 0 0.132 -0.075 0 0.117 -0.218 0.083 -0.051 -0.087 0.111 -0.113 0 0.058 0 0.163 0.126 -0.054 -0.091 0 0 -0.004 0

4 2 -0.044 0.221 0.202 0 0.132 -0.075 0 0.135 -0.218 0.083 -0.051 0 0.111 -0.113 0 0.058 0 0.061 0.165 -0.054 -0.091 0 0 -0.004 0

4 2 -0.044 0.221 0.202 0 0.132 -0.075 0 0.117 -0.218 0.083 -0.051 0 0.111 -0.124 0 0.251 0.027 0.163 0.085 -0.054 -0.091 0 0 -0.004 0

4 2 -0.044 0.221 0.202 0 0.132 -0.075 0 0.117 -0.218 0.083 -0.051 0 0.111 -0.124 0 0.058 0.027 0.163 0.171 -0.054 -0.091 0 0 -0.004 0

4 2 -0.044 0.221 0.202 0 0.132 -0.075 0 0.117 -0.218 0.083 -0.051 0 0.111 -0.113 0 0.058 0 0.054 0.126 -0.054 -0.091 0 0 -0.004 0

4 2 -0.044 0.221 0.202 0 0.132 -0.075 0 0.117 -0.218 0.083 -0.051 -0.107 0.111 -0.113 0 0.058 0 0.163 0.126 -0.054 -0.091 0 0 -0.004 0

4 2 -0.044 0.221 0.202 0 0.052 -0.075 0 0.117 -0.218 0.083 -0.051 0 0.111 -0.113 0 0.058 0 0.163 0.126 -0.054 -0.091 0 0 -0.004 0

4 2 -0.044 0.221 0.202 0 0.132 -0.075 0 0.117 -0.218 0.083 -0.051 0 0.111 -0.124 0 0.058 0.027 0.163 0.085 -0.054 0.004 0 0 -0.004 0

4 2 -0.044 0.221 0.202 0 0.132 -0.075 0 0.117 -0.254 0.083 -0.051 0 0.111 -0.113 0 0.058 0 0.163 0.128 -0.054 -0.091 0 0 -0.004 0

4 2 -0.044 0.137 0.181 0 0.191 -0.075 0 0 -0.215 0.344 -0.017 0 0.111 -0.114 0 0 0 0.163 0.009 -0.007 -0.091 0 0.388 -0.025 0

4 2 -0.044 0.137 0.202 0 0.132 -0.075 0 0.117 -0.218 0.083 -0.051 -0.073 0.111 -0.124 0 0.058 0 0.163 0.085 -0.054 -0.091 0 0 -0.031 0

4 2 -0.044 0.221 0.2 -0.097 0.132 -0.075 0 0.117 -0.218 0.083 -0.051 0 0.111 -0.124 0 0.058 0.027 0.163 0.085 -0.054 -0.091 0 0 -0.004 0

4 2 -0.044 0.137 0.187 0 0.191 -0.075 0 0 -0.215 0.083 -0.051 -0.058 0.111 -0.114 0 0 0 0.163 0.009 -0.007 -0.091 0 0.388 0.013 0

4 2 -0.044 0.137 0.202 0 0.132 -0.075 0 0.117 -0.218 0.083 -0.051 0 0.111 -0.124 0 0.058 0 0.163 0.085 -0.054 -0.139 0 0 -0.031 0

4 2 -0.044 0.221 0.202 0 0.132 -0.075 0 0.117 -0.218 0.083 -0.051 0 0.111 -0.113 0 0.058 0 0.163 0.126 -0.039 -0.091 0 0 -0.156 0

4 2 -0.044 0.221 0.202 0 0.132 -0.075 0 0.117 -0.218 0.083 -0.051 0 0.104 -0.113 0 0.058 0 0.163 0.126 -0.054 -0.091 0 0 -0.004 0

4 2 -0.044 0.137 0.202 0 0.132 -0.075 0 0.117 -0.218 0.083 -0.051 0 0.111 -0.124 0 0.058 0 0.163 0.085 -0.054 -0.091 0 0 -0.031 0.08

4 2 -0.044 0.221 0.202 0 0.132 -0.026 0 0.117 -0.218 0.083 -0.051 -0.044 0.111 -0.113 0 0.058 0 0.163 0.085 -0.054 -0.091 0 0.01 -0.004 0

4 3 -0.044 0.221 0.202 0 0.132 -0.075 0 0.117 -0.153 0.083 -0.051 0 0.111 -0.124 0 0.058 0.027 0.163 0.085 -0.054 -0.091 0 0 -0.004 0

4 2 -0.044 0.221 0.202 0 0.132 -0.075 0 0.117 -0.218 0.083 -0.151 0 0.111 -0.124 0 0.058 0.027 0.163 0.085 -0.054 -0.091 0 0 -0.004 0

4 2 -0.044 0.137 0.202 0 0.132 -0.075 -0.043 0.117 -0.218 0.083 -0.051 0 0.111 -0.124 0 0.058 0 0.163 0.085 -0.054 -0.091 0 0 -0.031 0

4 2 -0.044 0.163 0.202 0 0.132 -0.075 0 0.117 -0.218 0.083 -0.051 0 0.111 -0.124 0 0.058 0 0.163 0.085 -0.054 -0.091 0 0 -0.031 0

4 2 -0.044 0.221 0.202 0.02 0.132 -0.075 0 0.117 -0.218 0.083 -0.051 0 0.111 -0.113 0 0.058 0 0.073 0.085 -0.054 -0.091 0 0 -0.129 0

4 1 -0.044 0.221 0.202 0 0.132 -0.075 0.089 0.117 -0.218 0.083 -0.051 0 0.111 -0.124 0 0.058 0.027 0.163 0.085 -0.054 -0.091 0 0 -0.004 0

4 1 -0.044 0.137 0.187 0 0.191 -0.075 0 0 -0.215 0.083 -0.051 0 0.111 -0.078 0 0 0 0.163 0.009 -0.007 -0.091 0 0.388 0.013 -0.089

4 1 -0.044 0.221 0.202 0 0.132 -0.075 0 0.117 -0.218 0.083 -0.051 0 0.111 -0.124 0 0.058 0.027 0.163 0.085 0.113 -0.091 0 0 -0.004 0

4 1 -0.044 0.221 0.202 0 0.132 -0.075 0 0.135 -0.218 0.083 -0.051 0 0.111 -0.113 0 0.058 0 0.163 0.165 -0.054 -0.091 0 0 -0.004 0

4 1 -0.044 0.303 0.202 0 0.132 -0.075 0 0.117 -0.218 0.083 -0.051 0 0.111 -0.113 0 0.058 0 0.163 0.126 -0.054 -0.091 0 0 -0.004 0

4 1 -0.044 0.119 0.181 0 0.191 -0.075 0 0 -0.215 0.083 -0.051 0 0.111 -0.114 0 0 0 0.129 0.009 0.001 -0.091 0 0.388 0.013 0

4 1 -0.044 0.137 0.187 0 0.191 -0.075 0.099 0 -0.215 0.083 -0.051 0 0.111 -0.114 0 0 0 0.163 0.009 -0.007 -0.091 0 0.388 0.013 0

4 1 -0.044 0.137 0.202 0 0.132 -0.075 0 0.117 -0.218 0.083 -0.051 0 0.111 -0.124 0 0.058 0 0.163 0.085 -0.054 -0.091 0 0 -0.031 -0.014

4 1 -0.044 0.221 0.202 0 0.132 -0.075 0 0.117 -0.218 0.083 -0.051 -0.044 0.111 -0.084 0 0.058 0 0.163 0.085 -0.054 -0.091 0 0.01 -0.004 0

4 1 -0.044 0.221 0.202 0 0.132 -0.075 0 0.117 -0.218 0.083 -0.051 0 0.111 -0.124 0 0.058 0.027 0.163 0.085 -0.054 -0.091 0 -0.064 -0.004 0

4 1 -0.044 0.137 0.187 0 0.191 -0.075 0 0 -0.215 0.083 -0.051 0 0.111 -0.114 0 0 0 0.163 0.009 -0.007 -0.11 0 0.388 0.013 0

4 1 -0.044 0.137 0.181 0 0.191 -0.075 0 0 -0.215 0.083 -0.051 -0.18 0.111 -0.114 0 0 0 0.163 0.009 -0.007 -0.2 0 0.388 0.013 0

4 1 -0.044 0.221 0.202 0 0.132 -0.075 0 0.117 -0.218 0.083 -0.051 0 0.111 -0.124 0 0.058 0.027 0.163 0.085 -0.054 -0.091 0 0 -0.065 0

4 1 -0.044 0.13 0.202 0 0.132 -0.075 0 0.117 -0.218 0.083 -0.051 0 0.111 -0.124 0 0.058 0.027 0.163 0.085 -0.054 -0.091 0 0 -0.004 0

4 1 -0.044 0.221 0.202 0 0.132 -0.075 0 0.117 -0.218 0.083 -0.051 0 0.111 -0.124 0.002 0.058 0.027 0.163 0.085 -0.054 -0.091 0 0 -0.004 0

4 1 -0.044 0.137 0.181 0 0.191 -0.075 0 0 -0.215 0.083 -0.051 -0.18 0.111 -0.114 0 0 0 0.163 0.009 -0.007 -0.091 0 0.388 0.038 0

4 1 -0.044 0.359 0.202 0 0.132 -0.075 0 0.117 -0.218 0.083 -0.051 0 0.111 -0.124 0 0.058 0.027 0.163 0.085 -0.054 -0.091 0 0 -0.004 0

4 1 -0.044 0.221 0.202 0 0.132 -0.075 0 0.117 -0.218 0.083 -0.051 -0.044 0.111 -0.113 0 0.058 0 0.124 0.085 -0.054 -0.091 0 0.01 -0.004 0

4 1 -0.044 0.137 0.187 0 0.191 -0.075 0 0 -0.215 0.083 -0.051 0 0.111 -0.114 0 0 0 0.163 0.088 -0.007 -0.091 0 0.388 0.013 0

4 1 -0.044 0.221 0.202 0 0.132 -0.075 0 0.179 -0.218 0.083 -0.051 0 0.111 -0.113 0 0.058 0 0.163 0.126 -0.054 -0.091 0 0 -0.156 0

4 1 -0.044 0.137 0.202 0 0.132 -0.075 0 0.117 -0.218 0.083 -0.051 0 0.111 -0.282 0 0.058 0 0.163 0.085 -0.054 -0.091 0 0 -0.031 0

4 1 -0.044 0.137 0.202 0 0.132 -0.075 0 0.117 -0.218 0.098 -0.051 0 0.111 -0.124 0 0.058 0 0.163 0.085 -0.054 -0.091 0 0 -0.031 0

4 1 -0.008 0.221 0.202 0 0.132 -0.075 0 0.117 -0.218 0.083 -0.051 -0.044 0.111 -0.113 0 0.058 0 0.163 0.085 -0.054 -0.091 0 0.01 -0.004 0

4 1 -0.044 0.137 0.181 0 0.191 -0.075 0 0 -0.215 0.344 -0.051 0 0.111 -0.114 0 0 0 0.163 0.014 -0.007 -0.091 0 0.388 0.013 0

4 1 -0.044 0.137 0.202 0 0.132 -0.075 0 0.117 -0.218 0.083 -0.051 0 0.111 -0.124 0 0.058 0 0.163 0.085 -0.054 -0.198 0 0 -0.031 0

4 1 -0.044 0.221 0.202 0 0.132 -0.075 0 0.117 -0.218 0.083 -0.051 -0.044 0.111 -0.113 0 0.058 0.028 0.163 0.085 -0.054 -0.091 0 0.01 -0.004 0

4 1 -0.044 0.221 0.202 0 0.132 -0.075 0 0.117 -0.254 0.083 -0.051 0 0.111 -0.124 0 0.058 0.027 0.163 0.085 -0.054 -0.091 0 0 -0.004 0

4 1 -0.044 0.221 0.202 0 0.132 -0.075 0 0.117 -0.218 0.083 -0.051 0 0.111 -0.113 0.073 0.058 0 0.163 0.126 -0.054 -0.091 0 0 -0.004 0

4 1 -0.044 0.221 0.202 0 0.132 -0.075 0 0.117 -0.218 0.083 -0.051 0 0.111 -0.113 0 0.058 0 0.163 0.167 -0.054 -0.091 0 0 -0.004 0

4 1 -0.044 0.221 0.202 0 0.132 -0.075 0 0.117 -0.218 0.083 -0.051 0 0.111 -0.113 0 0.058 0 0.073 0.085 -0.054 -0.091 0 0 -0.129 -0.334

4 1 -0.044 0.221 0.202 0 0.132 -0.075 0 0.117 -0.218 0.083 -0.051 0 0.111 -0.113 0 0.058 0 0.163 0.126 -0.054 -0.07 0 0 -0.004 0

4 1 -0.044 0.221 0.202 0 0.132 -0.075 0 0.117 -0.218 0.083 -0.051 0 0.111 -0.113 0 0.058 0 0.163 0.126 0.07 -0.091 0 0 -0.004 0

4 1 -0.044 0.221 0.202 0 0.132 -0.075 0 0.117 -0.218 0.083 -0.051 0 0.111 -0.124 0 0.058 0.027 0.163 0.077 -0.054 -0.091 0 0 -0.004 0

4 1 -0.044 0.137 0.187 0 0.191 -0.075 0 0 -0.215 0.083 -0.05 0 0.111 -0.114 0 0 0 0.163 0.009 -0.007 -0.091 0 0.388 0.013 0

4 1 -0.044 0.137 0.202 0 0.132 -0.075 0 0.117 -0.218 0.083 -0.051 0 0.111 -0.124 0 0.098 0 0.163 0.085 -0.054 -0.091 0 0 -0.031 0

4 1 -0.044 0.221 0.202 0 0.132 -0.075 0 0.117 -0.269 0.083 -0.051 0 0.111 -0.124 0 0.058 0.027 0.163 0.085 -0.054 -0.091 0 0 -0.004 0

4 1 -0.044 0.137 0.181 0 0.191 -0.075 0 0 -0.231 0.083 -0.051 0 0.111 -0.114 0 0 0 0.163 0.009 -0.007 -0.091 0 0.388 0.013 0

4 1 -0.044 0.137 0.202 0 0.132 -0.075 0 0.117 -0.218 -0.187 -0.051 0 0.111 -0.124 0 0.058 0 0.163 0.085 -0.054 -0.091 0 0 -0.031 0

4 1 -0.044 0.221 0.202 0 0.132 -0.075 0 0.117 -0.218 0.083 -0.051 0 0.046 -0.113 0 0.058 0 0.163 0.126 -0.054 -0.091 0 0 -0.156 0

4 1 -0.044 0.137 0.202 -0.016 0.132 -0.075 0 0.117 -0.218 0.083 -0.051 0 0.111 -0.124 0 0.058 0 0.163 0.085 -0.054 -0.091 0 0 -0.031 0

4 1 -0.044 0.221 0.202 0 0.132 -0.075 0 0.117 -0.218 0.083 -0.105 0 0.111 -0.113 0 0.058 0 0.163 0.126 -0.054 -0.091 0 0 -0.004 0

4 1 -0.044 0.169 0.181 0 0.191 -0.075 0 0 -0.215 0.344 -0.051 0 0.111 -0.114 0 0 0 0.163 0.009 -0.007 -0.091 0 0.388 0.013 0

4 1 -0.044 0.221 0.202 0 0.132 -0.075 0 0.117 -0.218 0.083 -0.051 0 0.111 -0.113 0 0.048 0 0.163 0.126 -0.054 -0.091 0 0 -0.004 0

4 1 -0.044 0.221 0.202 0 0.132 -0.075 0 0.117 -0.218 0.083 -0.051 0 0.111 -0.124 0 0.058 0.027 0.163 0.085 -0.054 -0.091 0 0 -0.004 0.031

4 1 -0.044 0.221 0.202 0 0.132 -0.075 0 0.117 -0.218 0.083 -0.051 0 -0.016 -0.124 0 0.058 0.027 0.163 0.085 -0.054 -0.091 0 0 -0.004 0

4 1 -0.044 0.221 0.202 0 0.132 -0.075 0 0.117 -0.218 0.083 -0.051 -0.05 0.111 -0.113 0 0.058 0 0.163 0.085 -0.054 -0.091 0 0.01 -0.004 0

4 1 -0.044 0.221 0.202 -0.08 0.132 -0.075 0 0.117 -0.218 0.083 -0.051 0 0.111 -0.113 0 0.058 0 0.163 0.126 -0.054 -0.091 0 0 -0.156 0

4 1 -0.044 0.137 0.181 0 0.191 -0.075 0 0 -0.215 0.083 -0.051 -0.18 0.111 -0.114 0 0 0 0.163 -0.119 -0.007 -0.091 0 0.388 0.013 0

4 1 -0.044 0.137 0.202 0 0.132 -0.075 0 0.117 -0.218 0.083 -0.051 0 0.111 -0.124 0 0.058 0 0.163 0.085 -0.054 -0.091 0 0.015 -0.031 0

4 1 -0.044 0.137 0.187 0 0.191 -0.075 0 0 -0.215 0.083 -0.051 0 0.111 -0.114 0 0 0 0.163 0.009 -0.007 -0.122 0 0.388 0.013 0

4 1 -0.044 0.137 0.181 0 0.191 -0.075 0 0 -0.215 0.083 -0.051 0 0.111 -0.114 0 0 0 0.163 0.009 -0.007 -0.091 0 0.48 0.013 0

4 1 -0.044 0.137 0.181 0 0.191 -0.075 0 0 -0.215 0.083 -0.051 0 0.111 -0.114 0 -0.041 0 0.163 0.009 -0.007 -0.091 0 0.388 0.013 0

4 1 -0.044 0.137 0.202 0 0.132 -0.075 0 0.117 -0.218 0.083 -0.051 0 0.111 -0.124 0 0.058 0 0.163 0.085 -0.054 -0.091 0 0.007 -0.031 0

4 1 -0.044 0.221 0.202 0 0.132 -0.075 0 0.117 -0.218 0.083 -0.051 0 0.111 -0.124 0 0.092 0.027 0.058 0.085 -0.054 -0.091 0 0 -0.004 0

4 1 -0.044 -0.054 0.181 0 0.191 -0.075 0 0 -0.215 0.083 -0.051 0 0.111 -0.114 0 0 0 0.163 0.009 -0.007 -0.091 0 0.388 0.013 0

4 1 -0.044 0.221 0.202 0 0.132 -0.075 0 0.117 -0.218 0.083 -0.051 0 0.111 -0.113 0 0.058 0 0.163 0.126 -0.054 -0.091 0 0 -0.015 0

4 1 -0.044 0.137 0.187 0 0.191 -0.075 0 0 -0.215 0.083 -0.051 0 0.111 -0.114 0 0 0 0.078 0.009 -0.007 -0.091 0 0.388 0.013 0

4 1 -0.044 0.137 0.202 0 0.132 -0.075 0 0.117 -0.218 0.083 0.001 0 0.111 -0.124 0 0.058 0 0.163 0.085 -0.054 -0.091 0 0 -0.031 0

4 1 -0.044 0.221 0.202 0 0.132 -0.075 0 0.117 -0.218 0.083 -0.051 0 0.111 -0.124 0 0.058 0.027 0.163 0.085 -0.054 -0.091 0 0 -0.004 -0.148

4 1 -0.044 0.137 0.181 0 0.191 -0.075 0 0 -0.215 0.083 -0.051 0 0.111 -0.114 0 -0.021 0 0.163 0.009 -0.007 -0.091 0 0.388 0.013 0

4 1 -0.044 0.221 0.202 0 0.132 -0.075 0 0.117 -0.218 0.083 -0.051 0.094 0.111 -0.124 0 0.058 0.027 0.163 0.085 -0.054 -0.091 0 0 -0.004 0

4 1 -0.044 0.221 0.202 0 0.132 -0.075 -0.066 0.117 -0.218 0.083 -0.051 0 0.111 -0.113 0 0.058 0 0.163 0.126 -0.054 -0.091 0 0 -0.156 0

4 1 -0.044 0.221 0.202 0 0.132 -0.075 0 0.117 -0.218 0.083 -0.051 0 0.111 -0.113 0 0.058 0 0.163 0.126 -0.054 -0.091 0 0 -0.095 0

4 1 -0.044 0.221 0.202 0 0.132 -0.075 0 0.117 -0.218 -0.072 -0.051 0 0.111 -0.124 0 0.058 0.027 0.058 0.085 -0.054 -0.091 0 0 -0.004 0

5 1284 -0.027 0 0.005 0 -0.078 0 0 0.158 -0.003 0.092 0 -0.1 0.059 0.191 -0.004 0.031 -0.017 0.002 0.163 0.139 0 0 0.114 0 0.192

5 924 -0.027 0 0.005 0 -0.078 0 0 0.158 -0.003 0.092 0 -0.1 0.059 0.191 -0.004 0.031 0.092 0.002 0.163 0.139 0 0 0.114 0 0.192

5 647 -0.027 0 0.005 0 -0.078 0 0 0.158 -0.003 0.092 0 -0.1 0.059 0.191 -0.004 0.031 -0.017 0.002 0.267 0.139 0 0 0.114 0 0.192

5 415 -0.027 -0.151 0.005 0 -0.078 0 0 0.158 -0.003 0.092 0 -0.1 0.059 0.191 -0.004 0.031 -0.017 0.002 0.267 0.139 0 0 0.114 0 0.192

5 148 -0.027 0 0.005 0 -0.078 0 0 0.158 -0.003 0.092 0 -0.1 0.056 0.191 -0.004 0.031 -0.017 0.002 0.163 0.139 0 0 0.114 0 0.192

5 72 -0.027 0 0.005 -0.03 -0.078 0 0 0.158 -0.003 0.092 0 -0.1 0.059 0.191 -0.004 0.031 -0.017 0.002 0.163 0.139 0 0 0.114 0 0.192

5 67 -0.027 0 0.005 0 -0.078 0 0 0.158 -0.003 0.092 0 -0.1 0.059 0.191 -0.004 -0.038 -0.017 0.002 0.267 0.139 0 0 0.114 0 0.192

5 34 -0.027 0 0.005 0 -0.078 0 0 0.158 -0.003 0.092 0 -0.1 -0.122 0.191 -0.004 0.031 0.092 0.002 0.163 0.139 0 0 0.114 0 0.192

5 32 -0.027 0 0.005 0 -0.078 0 0 0.158 -0.003 0.092 0 -0.1 0.059 0.191 -0.004 0.031 0.092 0.002 0.163 0.139 0 0 0.043 0 0.192

5 29 -0.027 0 0.005 0 -0.078 0 0 0.158 -0.003 0.092 0 -0.1 0.059 0.191 -0.004 -0.038 -0.017 0.002 0.267 0.139 0 0 0.008 0 0.192

5 24 -0.027 0 0.005 0 -0.078 0 0 0.158 -0.003 0.092 0 -0.1 0.059 0.191 -0.004 0.031 0.092 0.002 0.163 -0.049 0 0 0.114 0 0.192

5 19 -0.027 0 0.005 -0.074 -0.078 0 0 0.158 -0.003 0.092 0 -0.1 0.059 0.191 -0.004 0.031 0.092 0.002 0.163 0.139 0 0 0.114 0 0.192

5 18 -0.027 0 0.005 0 -0.078 0 0 0.158 -0.003 0.092 0 -0.1 0.059 0.191 -0.004 0.031 -0.017 0.002 0.163 0.158 0 0 0.114 0 0.192

5 17 -0.027 0 0.005 0 -0.078 0 0 0.158 -0.003 0.092 0 -0.1 0.059 0.191 -0.004 0.031 -0.017 0.002 0.267 0.139 0 0 0.114 0 0.154

5 17 -0.027 0 0.005 0 -0.078 0 0 0.158 -0.003 0.092 0 -0.1 0.059 0.191 -0.004 0.031 -0.017 0.002 0.163 0.139 0 0 0.114 0 0.198

5 15 -0.027 0 0.005 0 -0.078 0 0 0.158 -0.003 0.092 0 -0.1 0.059 0.191 -0.004 0.031 -0.017 0.002 0.163 0.139 0 0 0.175 0 0.192

5 14 -0.027 0 0.005 0 -0.078 0 0 0.158 -0.003 0.092 0 -0.114 0.059 0.191 -0.004 0.031 -0.017 0.002 0.163 0.139 0 0 0.114 0 0.192

5 12 -0.027 0 0.005 0 -0.078 -0.102 0 0.158 -0.003 0.092 0 -0.1 0.059 0.191 -0.004 0.031 -0.017 0.002 0.267 0.139 0 0 0.114 0 0.192

5 11 -0.027 0 0.005 0 -0.078 0.136 0 0.158 -0.003 0.092 0 -0.1 0.059 0.191 -0.004 0.031 -0.017 0.002 0.163 0.139 0 0 0.114 0 0.192

5 18 -0.027 0 0.005 0 -0.078 0 0 0.158 -0.003 0.092 0 -0.1 0.059 0.191 -0.162 0.031 -0.017 0.002 0.267 0.139 -0.226 0 0.114 0 0.192

5 9 -0.027 -0.151 0.005 0 -0.078 0 0 0.158 -0.003 0.092 -0.029 -0.1 0.059 0.191 -0.004 0.031 -0.017 0.002 0.267 0.139 0 0 0.114 0 0.192

5 9 -0.027 0 0.005 0 -0.078 0 0 0.158 -0.003 0.092 0 -0.1 0.059 0.191 -0.004 0.031 -0.017 0.002 0.161 0.139 0 0 0.114 0 0.154

5 8 -0.027 -0.151 0.005 0 -0.12 0 0 0.158 -0.003 0.092 0 -0.1 0.059 0.191 -0.004 0.031 -0.017 0.002 0.267 0.139 0 0 0.114 0 0.192

5 8 -0.027 0 0.005 0 -0.078 0 0 0.158 -0.003 0.092 0 -0.1 0.059 0.191 -0.004 0.031 -0.017 0.002 0.163 0.139 0 0 0.114 -0.109 0.192

5 8 -0.027 0 0.005 0 -0.078 0 0 0.158 -0.003 0.092 0 -0.1 0.059 0.191 -0.004 0.031 -0.017 0.002 0.163 0.139 0 0 0.155 0 0.192

5 7 -0.027 0 0.005 0 -0.078 0 0 0.158 -0.003 0.092 0 -0.1 0.059 0.191 -0.004 0.031 -0.017 0.002 0.267 0.139 0.046 0 0.114 0 0.192

5 7 -0.027 0 0.005 0 -0.078 0 0 0.158 -0.003 0.092 0 -0.1 0.059 0.191 -0.004 0.031 -0.017 0.002 0.267 0.139 0 0.014 0.114 0 0.192

5 7 -0.027 0 0.005 0 -0.078 0 0 0.158 -0.003 0.092 0 -0.1 0.059 0.191 -0.004 0.031 -0.017 0.002 0.163 0.139 0 0 0.136 0 0.192

5 6 -0.027 0 0.005 0 -0.078 0 0 0.158 -0.003 -0.025 0 -0.1 0.059 0.191 -0.004 0.031 -0.017 0.002 0.163 0.139 0 0 0.114 0 0.192

5 6 -0.027 0 0.005 0 -0.078 0 0 0.158 -0.003 0.092 0 -0.1 -0.014 0.191 -0.004 0.031 0.092 0.002 0.163 0.139 0 0 0.114 0 0.192

5 6 -0.027 0 0.005 0 -0.078 0 0 0.214 -0.003 0.092 0 -0.1 0.059 0.191 -0.004 0.031 -0.017 0.002 0.163 0.139 0 0 0.114 0 0.192

5 5 -0.027 0 0.005 0 -0.078 0 0 0.158 -0.003 0.092 0.02 -0.1 0.059 0.191 -0.004 0.031 -0.017 0.002 0.163 0.139 0 0 0.114 0 0.192

5 4 -0.027 0 0.005 0 -0.078 0 0 0.158 -0.003 0.092 0.055 -0.1 0.059 0.191 -0.004 -0.008 0.243 0.002 0.163 0.139 0 0 0.114 0 0.192

5 4 -0.027 0 0.005 0 -0.11 0 -0.007 0.158 -0.003 0.171 0 -0.1 0.059 0.191 -0.004 0.031 0.092 0.002 0.163 0.139 0 0 0.114 0 0.192

5 4 -0.027 -0.052 0.005 0 -0.078 0 0 0.158 -0.003 0.092 0 -0.1 0.059 0.191 -0.004 0.031 -0.017 0.002 0.161 0.139 0 0 0.114 0 0.154

5 5 -0.027 0 0.005 0 -0.078 0 0 0.158 -0.003 0.092 0 -0.1 0.059 0.139 -0.004 0.031 -0.017 0.002 0.267 0.139 0 0 0.114 0 0.192

5 4 -0.027 0 0.005 0 -0.078 0 0 0.158 -0.003 0.092 0 -0.1 0.059 0.191 -0.004 0.031 0.092 0.002 0.163 0.139 0 0 0.052 0 0.192

5 4 -0.027 0 0.005 0 -0.078 0 0 0.158 -0.003 0.092 0 -0.1 0.059 0.191 -0.004 0.031 0.201 0.002 0.163 0.139 0 0 0.114 0 0.192

5 4 -0.027 0 0.005 0 -0.078 -0.077 0 0.158 -0.003 0.092 0 -0.1 0.059 0.191 -0.004 0.031 -0.017 0.002 0.163 0.139 0 0 0.114 0 0.192

5 4 -0.027 0 0.005 0 -0.078 0 0 0.158 -0.003 0.092 0 -0.1 0.059 0.191 -0.004 0.031 -0.017 0.002 0.359 0.139 0 0 0.114 0 0.192

5 4 -0.027 0 0.005 0 -0.078 0 0 0.158 -0.003 0.092 0 -0.1 0.059 0.191 -0.004 0.031 -0.017 0.002 0.163 0.141 0 0 0.114 0 0.192

5 4 -0.027 0 0.005 0 -0.078 0 0 0.158 -0.003 0.092 0 -0.041 0.059 0.191 -0.004 -0.038 -0.017 0.002 0.267 0.139 0 0 0.114 0 0.192

5 4 -0.027 0 0.005 0 -0.078 0 0 0.158 -0.003 0.092 0 -0.1 0.059 0.191 -0.004 0.031 -0.017 0.002 0.163 0.139 0 0 0.142 0 0.192

5 4 -0.027 0 0.005 0 -0.078 0 0 0.158 -0.003 0.092 0 -0.1 0.059 0.191 0.036 0.031 0.092 0.002 0.163 0.139 0 0 0.114 0 0.192

5 4 -0.027 0 0.005 0 -0.078 0 0 0.187 -0.003 0.092 0 -0.1 0.059 0.191 -0.004 0.031 -0.017 0.002 0.267 0.139 0 0 0.114 0 0.192

5 3 -0.027 0 0.005 0 -0.078 0 0 0.158 -0.003 0.092 0 -0.107 0.059 0.191 -0.004 0.031 0.092 0.002 0.163 0.139 0 0 0.114 0 0.192

5 2 -0.027 0 0.005 0 -0.11 0 0 0.158 -0.003 0.171 0 -0.1 0.059 0.191 -0.004 0.031 0.092 0.002 0.163 0.139 0 0 0.114 0 0.192

5 2 -0.027 -0.151 0.036 0 -0.078 0 0 0.158 -0.003 0.092 0 -0.1 0.059 0.191 -0.004 0.031 -0.017 0.002 0.267 0.139 0 0 0.114 0 0.192

5 2 -0.027 0 0.005 0 0.063 0 0 0.158 -0.003 0.092 0 -0.1 0.059 0.191 -0.004 0.031 -0.017 0.002 0.267 0.139 0 0 0.114 0 0.192

5 2 -0.027 0 0.027 0 -0.078 0 0 0.158 -0.003 0.092 0 -0.1 0.059 0.191 -0.004 0.031 -0.017 0.002 0.163 0.139 0 0 0.114 0 0.192

5 2 -0.027 0 0.005 0 -0.078 -0.008 0 0.158 -0.003 0.092 0 -0.1 0.059 0.191 -0.004 0.031 0.092 0.002 0.163 0.139 0 0 0.114 0 0.192

5 2 -0.027 0 0.005 0 -0.078 0 0 0.158 -0.106 0.092 0 -0.1 0.059 0.191 -0.004 0.031 -0.017 0.002 0.163 0.139 0 0 0.114 0 0.192

5 2 -0.027 -0.151 0.005 0 -0.078 0 0 0.158 -0.003 0.092 0 -0.1 0.059 0.191 -0.004 0.031 -0.017 0.002 0.267 0.139 0 0.099 0.114 0 0.192

5 2 -0.027 0 0.005 0 -0.078 0 0 0.158 -0.003 0.092 0 -0.1 0.059 0.269 -0.004 0.031 -0.017 0.002 0.163 0.139 0 0 0.114 0 0.192

5 2 -0.027 0 0.005 0 -0.078 0 0 0.158 -0.003 0.092 0 -0.1 0.059 0.191 -0.004 0.031 -0.017 0.002 0.267 0.139 0 0 0.114 0 0.24

5 2 -0.027 0 0.005 0 -0.078 0 -0.013 0.158 -0.003 0.092 0 -0.1 0.059 0.191 -0.004 0.031 -0.017 0.002 0.163 0.139 0 0 0.114 0 0.192

5 2 -0.027 0 0.005 0 -0.078 0 0 0.158 -0.003 0.092 0 -0.1 0.059 0.191 -0.004 0.031 0.092 0.002 0.163 0.139 0 0 0.056 0 0.192

5 2 -0.027 0 0.005 0 -0.078 0 0 0.158 -0.003 0.092 0 -0.1 0.059 0.191 -0.004 0.031 -0.017 0.002 0.17 0.139 0 0 0.114 0 0.192

5 2 -0.027 0 0.005 0 -0.078 0 0 0.158 -0.003 0.092 0 -0.1 0.059 0.191 -0.004 0.031 0.092 0.002 0.163 0.139 0 0 0.114 -0.008 0.192

5 2 -0.027 0 0.005 0 -0.078 0 0 0.158 -0.003 0.092 0 -0.1 0.059 0.191 -0.004 0.031 -0.017 0.002 0.163 0.139 0 0 0.265 0 0.192

5 2 -0.027 0 0.005 0 -0.078 0 0 0.158 -0.003 0.092 0 -0.1 0.059 0.191 -0.004 0.031 -0.017 0.002 0.163 0.139 0 0 0.114 -0.109 0.183

5 2 -0.027 0 0.005 0 -0.078 0 0 0.158 -0.003 0.092 0 -0.1 0.059 0.191 -0.004 0.031 -0.017 0.002 0.163 0.139 0 0 0.114 0 0.314

5 2 -0.027 0 0.005 0 -0.078 0 0 0.158 -0.003 0.092 0 -0.1 0.059 0.191 -0.004 0.031 -0.017 0.002 0.163 0.139 0 0 0.114 0 0.279

5 2 -0.027 0 0.005 0 -0.174 0 0 0.158 -0.003 0.092 0 -0.1 0.059 0.191 -0.004 0.031 -0.017 0.002 0.267 0.139 0 0 0.114 0 0.192

5 2 -0.027 0 0.005 0 -0.078 0 0 0.158 -0.003 0.092 0 -0.1 0.059 0.191 -0.004 0.031 -0.017 0.018 0.267 0.139 0 0 0.114 0 0.192

5 2 -0.027 0 0.005 0 -0.078 0 0 0.158 -0.003 0.092 0 -0.1 0.059 0.068 -0.004 0.031 -0.017 0.002 0.163 0.139 0 0 0.114 0 0.192

5 2 -0.027 0 0.005 0 -0.078 0 0 0.158 -0.003 0.092 0 -0.1 0.059 0.191 -0.004 0.031 -0.017 0.025 0.163 0.139 0 0 0.114 0 0.192

5 2 -0.027 0 0.005 0 -0.283 0 0 0.158 -0.003 0.092 0 -0.1 0.059 0.191 -0.004 0.031 -0.017 0.002 0.267 0.139 0 0 0.114 0 0.192

5 2 -0.027 0 0.005 0 -0.078 0 0 0.158 0.009 0.092 0 -0.1 0.059 0.191 -0.004 0.031 0.092 0.002 0.163 0.139 0 0 0.114 0 0.192

5 2 -0.027 0 0.005 0 -0.078 0 0 0.158 -0.003 0.092 0 -0.1 0.059 0.191 -0.004 0.031 0.092 -0.044 0.163 0.139 0 0 0.114 0 0.192

5 1 -0.027 0 0.005 0 -0.078 0 0 0.158 -0.003 0.092 0 -0.1 0.059 0.191 -0.004 0.031 -0.017 0.002 0.163 0.139 0.095 0 0.114 0 0.192

5 1 -0.027 0 0.005 0 -0.078 0 0 0.158 -0.003 0.092 0 -0.1 0.059 0.191 -0.004 0.031 -0.017 0.002 0.267 0.139 0.19 0 0.086 0 0.154

5 1 -0.027 0 0.005 0 -0.078 0 0 0.158 -0.003 0.092 0 -0.1 0.059 0.191 -0.004 0.031 0.341 0.002 0.163 0.139 0 0 0.114 0 0.192

5 1 -0.027 0 0.005 0 -0.078 0 0 0.158 -0.003 0.092 0 -0.1 0.177 0.191 -0.004 0.031 -0.017 0.002 0.267 0.139 0 0 0.114 0 0.154

5 1 -0.027 0 0.005 0 -0.078 0 0 0.158 -0.003 0.092 0 -0.107 0.059 0.191 -0.004 0.031 0.092 0.002 0.163 0.139 0 0 0.029 0 0.192

5 1 -0.027 -0.151 0.005 0 -0.078 0 0 0.158 -0.003 0.092 -0.029 -0.1 0.059 0.191 -0.004 0.089 -0.017 0.002 0.267 0.139 0 0 0.114 0 0.192

5 1 -0.027 0 0.005 0 -0.078 0 0 0.158 -0.285 0.092 0 -0.1 0.059 0.191 -0.004 0.031 0.092 0.002 0.163 0.139 0 0 0.114 0 0.192

5 1 -0.027 0 0.005 0 -0.078 0 0 0.158 -0.003 0.092 0 -0.093 0.059 0.191 -0.004 0.031 -0.017 0.002 0.267 0.139 0 0 0.114 0 0.154

5 1 -0.027 0 0.005 0 -0.078 0 0 0.158 -0.003 0.092 0 -0.1 0.059 0.191 -0.004 0.031 -0.017 0.002 0.327 0.139 0 0 0.114 0 0.154

5 1 -0.027 -0.151 0.005 0 -0.078 0 0 0.158 -0.003 -0.019 0 -0.1 0.059 0.191 -0.004 0.031 -0.017 0.002 0.267 0.139 0 0 0.114 0 0.192

5 1 -0.027 0 0.005 0 -0.078 0 0 0.158 -0.003 0.092 0 -0.1 0.059 0.191 -0.004 0.031 0.092 0.002 0.163 0.139 -0.009 0 0.114 0 0.192

5 1 -0.027 0 0.005 0 -0.078 0 0 0.158 -0.003 0.092 0 -0.248 0.059 0.191 -0.004 0.031 0.092 0.002 0.163 0.139 0 0 0.114 0 0.192

5 1 -0.027 0 0.005 0 -0.078 0 0 0.158 -0.003 0.092 0 -0.1 0.059 0.191 -0.004 0.031 0.092 0.002 0.163 0.139 0.084 0 0.114 0 0.192

5 1 -0.027 0 0.005 0 -0.078 0 0 0.158 -0.003 0.092 0 -0.1 0.059 0.191 -0.033 0.031 -0.017 0.002 0.163 0.139 0 0 0.114 0 0.192

5 1 -0.027 0 0.005 0 -0.078 0 0 0.158 -0.003 0.092 0 -0.1 0.091 0.191 -0.004 0.031 -0.017 0.002 0.163 0.139 0 0 0.114 0 0.192

5 1 -0.027 0 0.005 0 -0.078 0 0.005 0.158 -0.003 0.092 0 -0.1 0.059 0.269 -0.004 0.031 -0.017 0.002 0.163 0.139 0 0 0.114 0 0.192

5 1 -0.027 0 0.005 0 -0.078 0 0 0.158 -0.003 0.092 0 -0.1 0.059 0.191 -0.061 0.031 0.092 0.002 0.163 0.139 0 0 0.114 0 0.192

5 1 -0.027 -0.151 0.005 0 0.046 0 0 0.158 -0.003 0.092 0 -0.1 0.059 0.191 -0.004 0.031 -0.017 0.002 0.267 0.139 0 0 0.114 0 0.192

5 1 -0.027 0 0.005 0 -0.078 0 0 0.158 -0.003 0.092 0 -0.1 0.059 0.191 -0.004 0.031 -0.017 0.002 0.163 0.139 0 0 0.114 0 0.044

5 1 -0.027 0 0.005 0 -0.078 0 0 0.158 0.026 0.092 0 -0.1 0.059 0.191 -0.004 0.031 0.092 0.002 0.163 0.139 0 0 0.114 0 0.192

5 1 -0.027 0 0.005 0 -0.078 0 0 0.158 -0.003 0.092 0 -0.1 0.059 0.191 -0.004 0.031 -0.017 0.002 0.267 0.139 0 -0.068 0.114 0 0.192

5 1 -0.027 0 0.005 0 -0.227 0 0 0.158 -0.003 0.092 0 -0.1 0.059 0.191 -0.004 0.031 -0.017 0.002 0.163 0.139 0 0 0.114 0 0.192

5 1 -0.027 0 0.005 0 -0.078 0 0 0.158 -0.003 0.092 0 -0.1 0.059 0.191 -0.004 0.031 -0.017 0.002 0.163 0.103 0 0 0.114 0 0.192

5 1 -0.027 0 0.005 0 -0.078 0 0 0.158 -0.003 0.092 0 -0.1 0.059 0.191 -0.004 -0.166 -0.017 0.002 0.267 0.139 0 0.014 0.114 0 0.192

5 1 0.078 0 0.005 0 -0.078 0 0 0.158 -0.003 0.092 0 -0.1 0.059 0.191 -0.004 0.031 0.092 0.002 0.163 0.139 0 0 0.114 0 0.192

5 1 -0.027 -0.151 0.005 0 -0.078 0 0 0.158 -0.003 0.092 0 -0.1 0.059 0.191 -0.004 0.031 -0.017 0.002 0.273 0.139 0 0 0.114 0 0.192

5 1 -0.027 -0.151 0.005 0 -0.078 0 0.094 0.158 -0.003 0.092 0 -0.1 0.059 0.191 -0.004 0.031 -0.017 0.002 0.267 0.139 0 0 0.114 0 0.192

5 1 -0.027 0 0.005 0 -0.078 0 0 0.158 -0.003 0.092 0 -0.1 0.059 0.191 -0.004 0.031 0.092 0.135 0.163 0.139 0 0 0.114 0 0.192

5 1 -0.027 -0.151 0.005 0 -0.078 0 0 0.158 -0.003 0.092 0 -0.1 0.059 0.191 -0.004 0.031 -0.017 0.002 0.267 0.139 0 -0.123 0.114 0 0.192

5 1 -0.027 0 0.005 0 -0.078 0 0 0.158 -0.003 0.092 0 -0.1 0.059 0.191 -0.004 0.031 -0.017 0.002 0.163 0.139 0 0 0.114 0 0.216

5 1 -0.027 0 0.005 0 -0.078 0 0.009 0.158 -0.003 0.092 0 -0.1 0.059 0.191 -0.004 0.031 0.092 0.002 0.163 0.139 0 0 0.114 0 0.192

5 1 -0.027 0 0.005 0 -0.078 0 0 0.158 -0.003 0.092 0 -0.1 0.059 0.191 0.167 0.031 -0.017 0.002 0.163 0.139 0 0 0.114 0 0.192

5 1 -0.027 0 0.005 0 -0.078 0 0 0.158 -0.003 0.092 0 -0.1 0.059 0.191 -0.004 -0.062 -0.017 0.002 0.267 0.139 0 0 0.114 0 0.192

5 1 -0.027 0 0.005 0 -0.078 0 0 0.158 -0.003 0.092 0 -0.1 0.059 0.191 -0.004 0.031 -0.013 0.002 0.163 0.139 0 0 0.114 0 0.192

5 1 -0.027 -0.151 0.005 0 -0.128 0 0 0.158 -0.003 0.092 0 -0.1 0.059 0.191 -0.004 0.031 -0.017 0.002 0.267 0.139 0 0 0.114 0 0.192

5 1 -0.027 0 0.005 0 -0.078 0 0 0.158 -0.003 0.092 0 -0.1 0.059 0.191 -0.004 0.031 -0.017 -0.055 0.163 0.139 0 0 0.114 0 0.192

5 1 -0.027 -0.118 0.005 0 -0.078 0 0 0.158 -0.003 0.092 0 -0.1 0.059 0.191 -0.004 0.031 0.092 0.002 0.163 0.139 0 0 0.114 0 0.192

5 1 -0.027 0 0.005 0 -0.078 0 0 0.158 -0.003 0.092 0 -0.1 0.059 0.191 -0.004 0.031 -0.017 0.002 0.163 0.139 0 0 0.114 0 -0.012

5 1 -0.027 0 0.005 0 -0.078 0 0 0.065 -0.003 0.092 0 -0.1 0.059 0.191 -0.004 0.031 -0.017 0.002 0.163 0.139 0 0 0.114 0 0.192

5 1 -0.027 0 -0.064 -0.03 -0.078 0 0 0.158 -0.003 0.092 0 -0.1 0.059 0.191 -0.004 0.031 -0.017 0.002 0.163 0.139 0 0 0.114 0 0.192

5 1 -0.111 0 0.005 0 -0.078 0 0 0.158 -0.003 0.092 0 -0.1 0.059 0.191 -0.004 0.031 0.092 0.002 0.163 0.139 0 0 0.114 0 0.192

5 1 -0.027 0 0.005 0 -0.078 0 0 0.158 -0.003 0.101 0 -0.1 0.056 0.191 -0.004 0.031 -0.017 0.002 0.163 0.139 0 0 0.114 0 0.192

5 1 -0.027 0 0.005 0 -0.078 0 0 0.158 -0.003 0.092 0 -0.1 0.059 0.191 -0.084 0.031 -0.017 0.002 0.163 0.139 0 0 0.114 0 0.192

5 1 -0.027 0 0.005 0 -0.078 0 0 0.158 -0.003 0.092 0 -0.1 0.059 0.202 -0.004 0.031 -0.017 0.002 0.163 0.139 0 0 0.114 0 0.192

5 1 -0.027 0 0.005 0 -0.078 0.065 0 0.158 -0.003 0.092 0 -0.1 0.059 0.191 -0.004 0.031 -0.017 0.002 0.163 0.139 0 0 0.114 0 0.192

5 1 -0.027 0 0.005 0 -0.078 0 0 0.158 -0.003 0.092 0 -0.1 0.059 0.191 -0.004 0.031 0.036 0.002 0.267 0.139 0 0 0.114 0 0.192

5 1 -0.027 0 0.005 0 -0.193 0 0 0.158 -0.003 0.092 0 -0.1 0.059 0.191 -0.004 0.031 -0.017 0.002 0.163 0.139 0 0 0.114 0 0.192

5 1 -0.027 0 0.005 0 -0.078 0 0.069 0.158 -0.003 0.092 0 -0.1 0.059 0.191 -0.004 0.031 0.092 0.002 0.163 0.139 0 0 0.114 0 0.192

5 1 -0.027 -0.151 0.005 0 -0.078 -0.015 0 0.158 -0.003 0.092 0 -0.1 0.059 0.191 -0.004 0.031 -0.017 0.002 0.267 0.139 0 0 0.114 0 0.192

5 1 -0.027 0 0.005 0 -0.078 0 0 0.158 -0.003 0.092 0 -0.1 0.059 0.191 -0.004 0.031 -0.017 0.002 0.267 0.16 0 0 0.114 0 0.192

5 1 -0.027 0 0.005 0 -0.078 0 0 0.158 -0.003 0.092 0 -0.1 0.059 0.191 -0.004 0.031 0.092 0.002 0.163 0.139 0 -0.049 0.114 0 0.192

5 1 -0.027 0 0.005 0 -0.078 0 0 0.158 -0.003 0.072 0 -0.1 0.059 0.191 -0.004 0.031 -0.013 0.002 0.163 0.139 0 0 0.114 0 0.192

5 1 -0.027 0 0.005 0 -0.078 0 -0.088 0.158 -0.003 0.092 0 -0.1 0.059 0.191 -0.004 0.031 -0.017 0.002 0.163 0.139 0 0 0.114 0 0.192

5 1 -0.027 0 0.005 0 -0.085 0 0 0.158 -0.003 0.092 0 -0.1 0.059 0.191 -0.004 0.031 0.092 0.002 0.163 0.139 0 0 0.114 0 0.192

5 1 -0.027 0 0.005 0 -0.078 0 0 0.158 -0.003 0.092 0 -0.1 0.059 0.191 -0.004 0.045 -0.017 0.002 0.267 0.139 0 0 0.114 0 0.192

5 1 -0.027 0 0.005 0 -0.078 0 0 0.141 -0.003 0.092 0 -0.1 0.059 0.191 -0.004 0.031 0.092 0.002 0.163 0.139 0 0 0.114 0 0.192

5 1 -0.027 0 0.005 0 -0.078 -0.102 0 0.158 -0.003 0.092 0 -0.1 0.059 0.142 -0.004 0.031 -0.017 0.002 0.267 0.139 0 0 0.114 0 0.192

5 1 -0.027 0 0.005 0 -0.078 0 0 0.158 -0.003 0.092 0 -0.156 0.056 0.191 -0.004 0.031 -0.017 0.002 0.163 0.139 0 0 0.114 0 0.192

5 1 -0.027 0 0.005 0 -0.078 0 0 0.158 -0.003 0.092 0 -0.13 0.059 0.191 -0.004 0.031 -0.017 0.002 0.267 0.139 0 0 0.114 0 0.192

5 1 -0.027 0 0.005 0 -0.078 0 0 0.158 -0.003 0.092 0 -0.1 0.059 0.191 -0.004 0.031 -0.017 0.002 0.267 0.139 0 0 0.114 0 0.184

5 1 -0.027 0 0.082 0 -0.078 0 0 0.158 -0.003 0.092 0 -0.1 0.059 0.191 -0.004 0.031 -0.017 0.002 0.163 0.158 0 0 0.114 0 0.192

5 1 -0.027 0 0.005 0 -0.128 0 0 0.158 -0.003 0.092 0 -0.1 0.059 0.191 -0.004 0.031 -0.017 0.002 0.163 0.139 0 0 0.114 0 0.192

5 1 -0.027 0 0.005 -0.03 -0.078 0 0 0.158 -0.003 0.092 0 -0.1 0.059 0.191 -0.004 0.031 -0.017 0.002 0.163 0.139 0 0 0.114 0 0.207

5 1 -0.027 -0.151 0.005 0 -0.078 0 0 0.158 0.137 0.092 0 -0.1 0.059 0.191 -0.004 0.031 -0.017 0.002 0.267 0.139 0 0 0.114 0 0.192

5 1 -0.027 -0.151 0.005 0 -0.078 0 0 0.158 -0.003 0.092 0.002 -0.1 0.059 0.191 -0.004 0.031 -0.017 0.002 0.267 0.139 0 0 0.114 0 0.192

5 1 -0.027 0 0.005 0 -0.078 0 -0.085 0.158 -0.003 0.092 0 -0.1 0.059 0.191 -0.004 0.031 -0.017 0.002 0.163 0.139 0 0 0.114 0 0.192

5 1 -0.027 0 0.005 0 -0.078 0 0 0.158 -0.003 0.092 0 -0.1 0.059 0.191 -0.004 0.031 -0.017 0.002 0.163 0.139 0 0 0.114 -0.275 0.192

5 1 -0.027 0 -0.211 0 -0.078 0 0 0.158 -0.003 0.092 0 -0.1 0.059 0.191 -0.004 0.031 0.092 0.002 0.163 0.139 0 0 0.114 0 0.192

5 1 -0.027 -0.122 0.005 0 -0.078 0 0 0.158 -0.003 0.092 0 -0.1 0.059 0.191 -0.004 0.031 -0.017 0.002 0.163 0.139 0 0 0.114 0 0.192

5 1 -0.027 0.054 0.005 0 -0.078 0 0 0.158 -0.003 0.092 0 -0.1 0.056 0.191 -0.004 0.031 -0.017 0.002 0.163 0.139 0 0 0.114 0 0.192

5 1 -0.027 0 0.005 0 -0.078 0 0 0.158 -0.003 0.092 0 -0.1 0.059 0.191 -0.004 0.031 -0.105 0.002 0.163 0.139 0 0 0.114 0 0.192

5 1 -0.027 0 0.005 0 -0.078 0 0 0.158 -0.003 0.092 0 -0.1 0.059 0.191 -0.004 0.031 0.164 0.002 0.163 0.139 0 0 0.114 0 0.192

5 1 -0.027 0 0.005 0 -0.078 0 0 0.158 -0.003 0.092 0 -0.1 0.056 0.191 -0.004 0.031 -0.017 0.002 0.163 0.139 0 0 0.114 0.034 0.192

6 1029 -0.187 0.002 -0.028 -0.06 -0.17 0 -0.075 0.148 0 -0.005 -0.189 -0.187 0 -0.042 -0.048 -0.093 0 0 0.025 0 0.084 -0.661 0.066 -0.122 0.082

6 293 -0.187 0.002 -0.028 -0.06 -0.17 0 -0.075 0.148 0 -0.005 -0.189 -0.187 0 -0.042 -0.048 -0.093 0 0 0.025 -0.096 0.084 -0.661 0.066 -0.122 0.082

6 260 -0.216 0.002 -0.028 -0.06 -0.17 0 -0.075 0.148 0 -0.005 -0.189 -0.187 0 -0.042 -0.048 -0.093 0 0 0.025 0 0.084 -0.661 0.066 -0.122 0.082

6 184 -0.187 0.002 -0.028 -0.06 -0.17 0 -0.075 0.114 0 0 0.057 -0.125 0 -0.005 -0.048 -0.107 0.034 0 0.025 0 0.138 -0.536 -0.039 -0.122 0.055

6 159 -0.187 0.002 -0.028 -0.06 -0.17 0 -0.075 0.114 0 0 0.057 -0.125 0 -0.005 -0.048 -0.107 0.017 0 0.025 0 0.138 -0.536 -0.039 -0.122 0.055

6 150 -0.187 0.002 -0.028 -0.06 -0.17 0 -0.075 0.148 0 -0.005 -0.189 -0.187 -0.013 -0.042 -0.048 -0.093 0 0 0.025 0 0.084 -0.661 0.066 -0.122 0.082

6 123 -0.187 0.002 -0.028 -0.06 -0.17 0 -0.075 0.148 0 -0.005 -0.189 -0.187 0 -0.029 -0.048 -0.093 0 0 0.025 0 0.084 -0.661 0.066 -0.122 0.082

6 100 -0.187 0.002 -0.028 -0.06 -0.17 0 -0.075 0.114 0 0 0.057 -0.125 0 -0.005 -0.048 -0.107 0.017 0 0.025 0 0.138 -0.536 0.12 -0.122 0.055

6 90 -0.187 0.002 -0.028 -0.06 -0.17 0 -0.075 0.148 0 -0.005 -0.189 -0.318 -0.013 -0.042 -0.048 -0.093 0 0 0.025 0 0.084 -0.661 0.066 -0.122 0.082

6 83 -0.187 0.002 -0.028 -0.06 -0.17 0 -0.075 0.114 0 0 -0.189 -0.125 0 -0.042 -0.048 -0.107 0 0 0.025 0 0.084 -0.48 -0.039 -0.122 0.082

6 63 -0.199 0.002 -0.028 -0.06 -0.17 0 -0.075 0.148 0 -0.005 -0.189 -0.187 0 -0.042 -0.048 -0.093 0 0 0.025 0 0.084 -0.661 0.066 -0.122 0.082

6 95 -0.187 0.002 -0.028 -0.06 -0.17 0 -0.075 0.114 0 0 0.057 -0.125 0 -0.005 -0.092 -0.018 0.017 0 0.025 0 0.084 -0.536 -0.039 -0.122 0.055

6 53 -0.187 0.002 -0.028 -0.06 -0.17 0 -0.075 0.114 0 0 -0.189 -0.125 0 -0.042 -0.048 -0.107 0 0.014 0.025 0 0.084 -0.543 -0.039 -0.122 0.082

6 28 -0.187 0.002 -0.028 -0.06 -0.097 0 -0.075 0.148 0 -0.005 -0.189 -0.187 0 -0.042 -0.048 -0.093 0 0 0.025 -0.096 0.084 -0.661 0.066 -0.122 0.082

6 26 -0.187 0.002 -0.028 -0.06 -0.17 0 -0.075 0.114 0 0 -0.189 -0.125 0 -0.042 -0.048 -0.107 0 0 0.025 0 0.084 -0.48 -0.039 -0.122 0.071

6 13 -0.187 0.002 -0.028 -0.06 -0.17 0 -0.075 0.148 0 -0.005 -0.189 -0.187 0 -0.029 -0.048 -0.093 0 0 0.025 0 0.084 -0.76 0.066 -0.122 0.082

6 11 -0.187 0.002 -0.028 -0.06 -0.17 0 -0.075 0.148 0 -0.005 -0.189 -0.187 0 -0.042 0.125 -0.093 0 0 0.025 -0.096 0.084 -0.661 0.066 -0.122 0.082

6 9 -0.187 0.002 -0.028 -0.06 -0.17 0 -0.075 0.134 0 -0.005 -0.189 -0.187 0 -0.042 -0.048 -0.093 0 0 0.025 0 0.084 -0.661 0.066 -0.122 0.082

6 9 -0.187 0.002 -0.028 -0.06 -0.17 0 -0.075 0.148 0 -0.005 -0.189 -0.238 0 -0.029 -0.048 -0.093 0 0 0.025 0 0.084 -0.661 0.066 -0.122 0.082

6 8 -0.187 0.002 -0.028 -0.06 -0.17 0 -0.075 0.114 0 0 0.057 -0.125 0 -0.005 -0.092 -0.018 0.017 0 0.025 0 0.054 -0.536 -0.039 -0.122 0.055

6 6 -0.187 0.002 -0.028 -0.06 -0.17 0 -0.075 0.148 0 -0.005 -0.189 -0.187 0 -0.042 -0.048 -0.093 0 0 0.025 0 0.284 -0.661 0.066 -0.122 0.082

6 6 -0.187 0.002 -0.028 -0.089 -0.17 0 -0.075 0.148 0 -0.005 -0.189 -0.187 0 -0.029 -0.048 -0.093 0 0 0.025 0 0.084 -0.661 0.066 -0.122 0.082

6 5 -0.187 0.002 -0.028 -0.06 -0.17 0 -0.075 0.148 0 -0.005 -0.218 -0.187 -0.013 -0.042 -0.048 -0.093 0 0 0.025 0 0.084 -0.661 0.066 -0.122 0.082

6 5 -0.187 0.002 -0.028 -0.06 -0.17 0 -0.075 0.148 0 0.034 -0.189 -0.187 0 -0.042 -0.048 -0.093 0 0 0.025 0 0.084 -0.661 0.066 -0.122 0.082

6 4 -0.187 0.002 -0.028 -0.06 -0.17 0 -0.075 0.148 0 -0.005 -0.189 -0.187 0 -0.042 -0.14 -0.093 0 0 0.025 0 0.084 -0.661 0.066 -0.122 0.082

6 4 -0.187 0.002 -0.028 -0.06 -0.17 0 -0.075 0.114 0 0 -0.189 -0.125 0 -0.042 -0.048 -0.107 0 0.014 0.025 -0.018 0.084 -0.543 -0.039 -0.122 0.082

6 4 -0.187 0.002 -0.028 -0.06 -0.17 -0.129 -0.075 0.114 0 0 0.057 -0.125 0 -0.005 -0.048 -0.107 0.017 0 0.025 0 0.138 -0.536 0.12 -0.122 0.055

6 4 -0.187 0.002 -0.028 -0.06 -0.17 0 -0.075 0.114 0 0 0.057 -0.125 0 -0.005 -0.048 -0.107 0.017 0 0.025 0 0.263 -0.536 0.12 -0.122 0.055

6 4 -0.187 0.002 -0.028 -0.06 -0.17 0 -0.075 0.148 0 -0.005 -0.189 -0.187 -0.013 -0.042 -0.048 -0.093 0 0 0.025 0 0.191 -0.661 0.066 -0.122 0.082

6 3 -0.187 0.002 -0.028 -0.06 -0.17 0 -0.075 0.148 0 0.188 -0.189 -0.318 -0.013 -0.042 -0.048 -0.093 0 0 0.025 0 0.084 -0.661 0.066 -0.122 0.082

6 3 -0.187 0.002 -0.028 -0.06 -0.17 0 -0.075 0.148 0 -0.005 -0.189 -0.187 0 -0.042 -0.048 -0.093 0 0 0.025 0 0.084 -0.661 0.066 -0.165 0.082

6 3 -0.187 0.002 -0.028 -0.06 -0.17 0 -0.075 0.148 0 -0.005 -0.189 -0.187 0 -0.042 -0.048 -0.093 0 0 0.025 0 0.043 -0.661 0.066 -0.122 0.082

6 2 -0.187 -0.09 -0.028 -0.06 -0.17 0 -0.075 0.148 0 -0.005 -0.189 -0.187 0 -0.042 -0.048 -0.093 0 0 0.025 0 0.084 -0.661 0.066 -0.122 0.082

6 2 -0.187 0.002 -0.028 -0.06 -0.17 0 -0.075 0.148 0 -0.005 -0.189 -0.187 0 -0.042 -0.048 -0.093 0.179 0 0.025 0 0.084 -0.661 0.066 -0.122 0.082

6 2 -0.187 -0.09 -0.028 -0.06 -0.17 0 -0.075 0.148 0 -0.005 -0.189 -0.187 0 -0.042 -0.048 -0.093 0 0 0.115 0 0.084 -0.661 0.066 -0.122 0.082

6 2 -0.187 0.002 -0.028 -0.06 -0.17 0 -0.075 0.114 0 0 -0.189 -0.125 0 -0.042 -0.048 -0.107 0 0 0.025 0 0.084 -0.67 -0.039 -0.122 0.082

6 2 -0.187 0.002 -0.028 -0.06 -0.17 0 -0.046 0.148 0 -0.005 -0.189 -0.187 0 -0.042 -0.048 -0.093 0 0 0.025 0 0.084 -0.661 0.066 -0.122 0.082

6 2 -0.187 0.002 -0.028 -0.06 -0.17 0 -0.075 0.148 0 -0.005 -0.189 -0.318 -0.013 -0.042 -0.048 -0.093 0 0 0.025 0 0.084 -0.661 0.111 -0.122 0.082

6 2 -0.187 0.002 -0.028 0 -0.17 0 -0.075 0.148 0 -0.005 -0.189 -0.187 0 -0.029 -0.048 -0.093 0 0 0.025 0 0.084 -0.661 0.066 -0.122 0.082

6 2 -0.187 0.002 -0.028 -0.06 -0.17 0 -0.068 0.148 0 -0.005 -0.189 -0.187 0 -0.042 -0.048 -0.093 0 0 0.025 -0.096 0.084 -0.661 0.066 -0.122 0.082

6 2 -0.216 0.002 -0.028 -0.06 -0.17 0 -0.075 0.148 0 -0.005 -0.189 -0.087 0 -0.042 -0.048 -0.093 0 0 0.025 0 0.084 -0.661 0.066 -0.122 0.082

6 2 -0.187 0.002 -0.028 -0.06 -0.17 0 -0.075 0.148 0 -0.005 -0.189 -0.187 0 -0.043 -0.048 -0.093 0 0 0.025 -0.096 0.084 -0.661 0.066 -0.122 0.082

6 2 -0.187 0.002 -0.028 -0.06 -0.17 0 -0.075 0.148 0 -0.005 -0.473 -0.187 0 -0.042 -0.048 -0.093 0 0 0.025 0 0.084 -0.661 0.066 -0.122 0.082

6 2 -0.187 0.002 -0.028 -0.06 -0.17 -0.049 -0.075 0.148 0 -0.005 -0.189 -0.187 0 -0.042 -0.048 -0.093 0 0 0.025 0 0.084 -0.661 0.066 -0.122 0.082

6 2 -0.187 0.002 -0.028 -0.06 -0.17 0 -0.266 0.148 0 -0.005 -0.189 -0.187 0 -0.042 -0.048 -0.093 0 0 0.025 0 0.084 -0.661 0.066 -0.122 0.082

6 2 -0.187 0.002 -0.028 -0.06 -0.17 0 -0.075 0.148 0 -0.005 -0.189 -0.187 0 0.003 -0.048 -0.093 0 0 0.025 0 0.084 -0.661 0.066 -0.122 0.082

6 2 -0.187 0.002 -0.028 -0.06 -0.17 0 -0.075 0.148 0 -0.005 -0.189 -0.187 0 -0.042 -0.048 -0.093 0 0 0.025 0 -0.109 -0.661 0.066 -0.122 0.082

6 2 -0.187 0.002 -0.028 -0.06 -0.17 0 -0.075 0.148 0 -0.005 -0.189 -0.187 -0.1 -0.042 -0.048 -0.093 0 0 0.025 -0.096 0.084 -0.661 0.066 -0.122 0.082

6 2 -0.187 0.002 -0.028 -0.06 -0.17 0 -0.075 0.114 0 0 -0.189 -0.125 0 -0.042 -0.048 -0.107 0 0 0.025 0 0.255 -0.48 -0.039 -0.122 0.082

6 2 -0.187 0.002 -0.028 -0.06 -0.17 0 -0.122 0.148 0 -0.005 -0.189 -0.187 0 -0.042 -0.048 -0.093 0 0 0.025 -0.096 0.084 -0.661 0.066 -0.122 0.082

6 2 -0.216 0.002 -0.028 -0.06 -0.17 0 -0.075 0.148 0 -0.005 -0.189 -0.108 0 -0.042 -0.048 -0.093 0 0 0.025 0 0.084 -0.661 0.066 -0.122 0.082

6 2 -0.187 0.014 -0.028 -0.06 -0.17 0.076 -0.075 0.148 0 -0.053 -0.189 -0.187 0 -0.029 -0.048 -0.093 0 0 0.025 0 0.084 -0.661 0.066 -0.122 0.082

6 2 -0.187 0.002 -0.028 -0.06 -0.17 0 -0.075 0.114 0 0 -0.189 -0.125 0 -0.042 -0.048 -0.167 0 0 0.025 0 0.084 -0.48 -0.039 -0.122 0.082

6 2 -0.187 0.002 -0.028 -0.06 -0.17 0 -0.075 0.114 0 0 -0.189 -0.125 0 -0.042 -0.048 -0.139 0 0 0.025 0 0.084 -0.48 -0.039 -0.122 0.082

6 2 -0.187 0.002 -0.028 -0.06 -0.17 0 -0.075 0.28 0 0 0.057 -0.125 0 -0.005 -0.048 -0.107 0.034 0 0.025 0 0.138 -0.536 -0.039 -0.122 0.055

6 2 -0.216 0.002 -0.028 -0.038 -0.17 0 -0.075 0.148 0 -0.005 -0.189 -0.187 0 -0.042 -0.048 -0.093 0 0 0.025 0 0.084 -0.661 0.066 -0.122 0.082

6 2 -0.216 0.103 -0.028 -0.06 -0.17 0 -0.075 0.148 0 -0.005 -0.189 -0.187 0 -0.042 -0.048 -0.093 0 0 0.025 0 0.084 -0.661 0.066 -0.122 0.082

6 2 -0.187 0.002 -0.028 -0.06 -0.17 0 -0.189 0.148 0 -0.005 -0.189 -0.187 0 -0.042 -0.048 -0.093 0 0 0.025 -0.096 0.084 -0.661 0.066 -0.122 0.082

6 2 -0.187 0.002 -0.028 -0.06 -0.17 0 -0.075 0.148 0 -0.005 -0.189 -0.187 -0.1 -0.042 -0.048 -0.093 0 0 0.025 -0.096 0.049 -0.661 0.066 -0.122 0.082

6 2 -0.187 0.002 -0.028 -0.06 -0.17 0 -0.075 0.148 0 0.007 -0.189 -0.187 0 -0.042 -0.048 -0.093 0 0 0.025 0 0.084 -0.661 0.066 -0.122 0.082

6 2 -0.206 0.002 -0.028 -0.06 -0.17 0 -0.075 0.148 0 -0.005 -0.189 -0.187 0 -0.042 -0.048 -0.093 0 0 0.025 0 0.084 -0.661 0.066 -0.122 0.082

6 2 -0.187 0.002 -0.028 -0.06 -0.17 0 -0.075 0.114 0 0 0.064 -0.125 0 -0.005 -0.048 -0.107 0.034 0 0.025 0 0.138 -0.536 -0.039 -0.122 0.055

6 2 -0.187 0.002 -0.028 -0.06 -0.17 0 -0.075 0.148 0 -0.005 -0.189 -0.187 0 -0.042 -0.048 -0.093 0 0 0.025 0 0.084 -0.529 0.066 -0.122 0.082

6 2 -0.1 0.002 -0.028 -0.06 -0.17 0 -0.075 0.114 0 0 0.057 -0.125 0 -0.005 -0.092 -0.018 0.017 0 0.025 0 0.084 -0.536 -0.039 -0.122 0.055

6 1 -0.216 0.002 -0.028 -0.06 -0.17 0 -0.075 0.148 0 -0.005 -0.189 -0.187 0 -0.042 -0.048 -0.093 0 0 0.025 0 0.084 -0.661 0.066 -0.217 0.082

6 1 -0.216 0.002 -0.028 -0.06 -0.17 0 -0.075 0.148 0 0.123 -0.189 -0.187 0 -0.042 -0.048 -0.093 0 0 0.025 0 0.084 -0.661 0.066 -0.122 0.007

6 1 -0.187 0.002 -0.028 -0.06 -0.17 0 -0.075 0.148 0 -0.005 -0.189 -0.187 0 -0.042 -0.048 -0.061 0 0 0.025 0 0.084 -0.661 0.066 -0.122 0.082

6 1 -0.187 0.002 -0.028 -0.06 -0.17 0 -0.075 0.148 0 -0.005 -0.189 -0.187 -0.038 -0.042 -0.048 -0.093 0 0 0.025 0 0.084 -0.661 0.066 -0.122 0.082

6 1 -0.187 0.002 -0.028 -0.06 -0.17 0 -0.075 0.114 0 0 0.057 -0.125 0 -0.005 -0.048 -0.107 0.017 0 0.025 0 0.138 -0.536 0.12 -0.122 -0.163

6 1 -0.187 0.002 -0.028 -0.06 -0.17 0 -0.075 -0.084 0 0 0.057 -0.125 0 -0.005 -0.048 -0.107 0.017 0 0.025 0 0.138 -0.536 0.12 -0.122 0.055

6 1 -0.187 -0.156 -0.028 -0.06 -0.17 0 -0.075 0.148 0 -0.005 -0.189 -0.187 0 -0.042 -0.048 -0.093 0 0 0.025 0 0.084 -0.661 0.066 -0.122 0.082

6 2 -0.187 0.002 -0.028 -0.06 -0.17 0 -0.075 0.114 0 0 0.057 -0.125 0 -0.005 -0.124 -0.107 0.034 0 0.025 0 0.138 -0.536 -0.039 -0.122 0.055

6 1 -0.187 0.002 -0.028 -0.06 -0.17 0 -0.075 0.114 0 0 0.057 -0.125 0 -0.005 -0.048 -0.107 0.034 0 0.025 -0.155 0.138 -0.536 -0.039 -0.122 0.055

6 1 -0.187 0.002 -0.028 -0.06 -0.17 0 -0.075 0.114 0 0 0.057 -0.125 0 -0.005 -0.048 -0.107 0.017 0 0.025 0.123 0.138 -0.536 -0.039 -0.122 0.055

6 1 -0.187 0.002 -0.028 -0.156 -0.17 0 -0.075 0.148 0 -0.005 -0.189 -0.187 0 -0.042 -0.048 -0.093 0 0 0.025 0 0.084 -0.661 0.066 -0.122 0.082

6 1 -0.187 0.002 -0.028 -0.06 -0.17 0 -0.075 0.148 0 -0.005 -0.17 -0.187 0 -0.042 -0.048 -0.093 0 0 0.025 0 0.084 -0.661 0.066 -0.122 0.082

6 1 -0.187 0.054 -0.028 -0.06 -0.17 0 -0.075 0.148 0 -0.005 -0.189 -0.187 0 -0.042 -0.048 -0.093 0 0 0.025 -0.104 0.084 -0.661 0.066 -0.122 0.082

6 1 -0.187 -0.039 -0.028 -0.06 -0.17 0 -0.075 0.114 0 0 0.057 -0.125 0 -0.005 -0.092 -0.018 0.017 0 0.025 0 0.084 -0.536 -0.039 -0.122 0.055

6 1 -0.187 0.002 -0.028 -0.06 -0.17 0 -0.075 0.148 0 -0.005 -0.189 -0.16 0 -0.042 -0.048 -0.093 0 0 0.025 0 0.084 -0.661 0.066 -0.122 0.082

6 1 -0.199 0.002 0.065 -0.06 -0.17 0 -0.075 0.148 0 -0.005 -0.189 -0.187 0 -0.042 -0.048 -0.093 0 0 0.025 0 0.084 -0.661 0.066 -0.122 0.082

6 1 -0.187 0.002 -0.028 -0.06 -0.17 0 -0.075 0.148 0 -0.005 -0.189 -0.187 0 -0.042 -0.048 -0.265 0 0 0.025 0 0.084 -0.661 0.066 -0.122 0.082

6 1 -0.187 0.002 -0.028 -0.06 -0.17 0 -0.075 0.114 0 0 0.057 -0.125 0 -0.005 -0.048 -0.107 0.034 0 0.025 0 0.138 -0.527 -0.039 -0.122 0.055

6 1 -0.194 0.002 -0.028 -0.06 -0.17 0 -0.075 0.148 0 -0.005 -0.189 -0.187 0 -0.042 -0.048 -0.093 0 0 0.025 0 0.084 -0.661 0.066 -0.122 0.082

6 1 -0.187 0.002 -0.028 -0.06 -0.17 0 -0.075 0.148 0 -0.005 -0.189 -0.187 0 0.048 -0.048 -0.093 0 0 0.025 0 0.084 -0.661 0.066 -0.122 0.082

6 1 -0.187 0.002 -0.028 -0.06 -0.17 0 -0.075 0.114 0.08 0 0.057 -0.125 0 -0.005 -0.048 -0.107 0.034 0 0.025 0 0.138 -0.536 -0.039 -0.122 0.055

6 1 -0.187 0.002 0.043 -0.06 -0.17 0 -0.075 0.148 0 -0.005 -0.189 -0.187 0 -0.042 -0.048 -0.093 0 0 0.025 0 0.084 -0.661 0.066 -0.122 0.082

6 1 -0.187 0.002 -0.028 -0.06 -0.17 0 -0.075 0.148 0 -0.005 -0.189 -0.187 0 -0.042 -0.048 -0.285 0 0 0.025 0 0.084 -0.661 0.066 -0.122 0.082

6 1 -0.187 0.002 -0.028 -0.06 -0.247 0 -0.075 0.114 0 0 0.057 -0.125 0 -0.005 -0.048 -0.107 0.034 0 0.025 0 0.138 -0.536 -0.039 -0.122 0.055

6 1 -0.187 0.002 -0.028 -0.06 -0.17 0 -0.075 0.148 0 -0.005 -0.189 -0.187 0 -0.042 -0.048 -0.093 0 0 0.049 0 0.084 -0.661 0.066 -0.122 0.082

6 1 -0.187 0.002 0.134 -0.06 -0.17 0 -0.075 0.148 0 -0.005 -0.189 -0.187 0 -0.042 -0.048 -0.093 0 0 0.025 0 0.084 -0.661 0.066 -0.122 0.082

6 1 -0.216 0.002 -0.028 -0.06 -0.17 0 -0.075 0.148 0 -0.005 -0.189 -0.187 0 -0.042 -0.048 -0.093 0 0 0.025 0 0.084 -0.661 0.066 -0.127 0.082

6 1 -0.187 0.002 -0.028 -0.06 -0.17 0 -0.075 0.148 0 -0.005 -0.189 -0.187 0 -0.042 0.037 -0.093 0 0 0.025 -0.096 0.084 -0.661 0.066 -0.122 0.082

6 1 -0.216 0.002 -0.028 -0.06 -0.17 0 -0.075 0.148 0 -0.005 -0.189 -0.187 0 -0.042 -0.048 -0.093 0 0 0.057 0 0.084 -0.661 0.066 -0.122 0.082

6 1 -0.061 0.002 -0.028 -0.06 -0.17 0 -0.075 0.148 0 -0.005 -0.189 -0.187 0 -0.042 -0.048 -0.093 0 0 0.025 -0.096 0.084 -0.661 0.066 -0.122 0.082

6 1 -0.187 0.002 -0.028 -0.051 -0.17 0 -0.075 0.148 0 -0.005 -0.189 -0.187 0 -0.042 -0.048 -0.093 0 0 0.025 0 0.084 -0.661 0.066 -0.165 0.082

6 1 -0.187 0.002 -0.028 -0.06 -0.17 0 -0.075 0.148 0 -0.005 -0.189 -0.187 0 -0.042 0.077 -0.093 0 0 0.025 -0.096 0.084 -0.661 0.066 -0.122 0.082

6 1 -0.187 0.002 -0.028 -0.06 -0.17 0 -0.075 0.148 0 -0.005 -0.189 -0.187 0 -0.042 -0.048 -0.093 0 0 0.025 0 0.084 -0.66 0.066 -0.122 0.082

6 1 -0.187 0.002 -0.028 -0.06 -0.17 0 -0.075 0.148 0 -0.005 -0.189 -0.187 0 -0.073 -0.048 -0.093 0 0 0.025 0 0.084 -0.661 0.066 -0.122 0.082

6 1 -0.187 0.002 -0.028 -0.06 -0.17 0 -0.075 0.114 0 0 0.057 -0.125 0 -0.005 -0.016 -0.107 0.017 0 0.025 0 0.138 -0.536 0.12 -0.122 0.055

6 1 -0.187 0.002 -0.028 -0.06 -0.17 0 -0.075 -0.18 0 -0.005 -0.189 -0.187 -0.013 -0.042 -0.048 -0.093 0 0 0.025 0 0.084 -0.661 0.066 -0.122 0.082

6 1 -0.187 0.002 -0.028 -0.058 -0.17 0 -0.075 0.148 0 -0.005 -0.189 -0.187 0 -0.042 -0.048 -0.093 0 0 0.025 0 0.084 -0.661 0.066 -0.122 0.082

6 1 -0.187 0.002 -0.028 -0.06 -0.17 0 -0.075 0.079 0 0 0.057 -0.125 0 -0.005 -0.048 -0.107 0.017 0 0.025 0 0.138 -0.536 -0.039 -0.122 0.055

6 1 -0.187 0.002 -0.028 -0.06 -0.17 0 -0.075 0.148 0 -0.005 -0.189 -0.187 0 -0.042 -0.048 -0.093 -0.074 0 0.025 0 0.084 -0.661 0.066 -0.122 0.082

6 1 -0.199 0.002 -0.028 -0.06 -0.17 0 -0.075 0.148 0 -0.005 -0.189 -0.187 0 -0.042 -0.048 -0.093 0 0 -0.111 0 0.084 -0.661 0.066 -0.122 0.082

6 1 -0.187 0.002 -0.028 -0.06 -0.17 0 0.146 0.114 0 0 0.057 -0.125 0 -0.005 -0.048 -0.107 0.017 0 0.025 0 0.138 -0.536 0.12 -0.122 0.055

6 1 -0.187 0.002 -0.028 -0.06 -0.17 0 -0.075 0.148 0 -0.005 -0.189 -0.187 0 -0.042 -0.048 -0.093 0 0 0.025 0 0.084 -0.661 0.066 0.002 0.082

6 1 -0.216 0.002 -0.028 -0.06 -0.17 0 -0.075 0.148 0 -0.005 -0.189 -0.187 0 -0.042 -0.048 -0.093 0 0 0.025 -0.183 0.084 -0.661 0.066 -0.122 0.082

6 1 -0.187 0.002 -0.028 -0.06 -0.17 0 -0.075 0.114 0 0 0.057 -0.397 0 -0.005 -0.048 -0.107 0.034 0 0.025 0 0.138 -0.536 -0.039 -0.122 0.055

6 1 -0.187 0.002 -0.028 -0.06 -0.17 0 -0.075 0.148 0 -0.005 -0.189 -0.187 0 -0.029 -0.048 -0.093 0 0 0.025 0 0.084 -0.661 0.066 -0.122 -0.084

6 1 -0.187 0.002 -0.028 -0.06 -0.17 0 -0.15 0.148 0 -0.005 -0.189 -0.187 0 -0.042 -0.048 -0.093 0 0 0.025 0 0.084 -0.661 0.066 -0.122 0.082

6 1 -0.187 0.002 -0.028 -0.06 -0.17 0 -0.075 0.114 0 0 0.057 -0.125 0 -0.005 -0.048 -0.107 0.017 0 0.088 0 0.138 -0.536 -0.039 -0.122 0.055

6 1 -0.187 0.002 -0.028 -0.06 -0.17 0 -0.075 0.148 0 -0.005 -0.189 -0.187 -0.013 -0.042 -0.201 -0.093 0 0 0.025 0 0.084 -0.661 0.066 -0.122 0.082

6 1 -0.187 0.002 -0.028 -0.06 -0.17 0 -0.075 0.148 0 -0.005 -0.189 -0.187 0 -0.042 -0.048 -0.093 0 0 0.025 0 0.084 -0.661 0.073 -0.122 0.082

6 1 -0.187 0.002 0.07 -0.06 -0.17 0 -0.075 0.148 0 -0.005 -0.189 -0.187 0 -0.042 -0.048 -0.093 0 0 0.025 0 0.084 -0.661 0.066 -0.122 0.082

6 1 -0.187 0.002 -0.028 -0.06 -0.17 0 -0.075 0.114 0 0 0.057 -0.125 0 -0.005 -0.048 0.024 0.034 0 0.025 0 0.138 -0.536 -0.039 -0.122 0.055

6 1 -0.187 0.002 -0.028 -0.06 -0.17 0 -0.075 0.148 0 -0.005 -0.189 -0.331 -0.013 -0.042 -0.048 -0.093 0 0 0.025 0 0.084 -0.661 0.066 -0.122 0.082

6 1 -0.199 0.002 -0.028 -0.112 -0.17 0 -0.075 0.148 0 -0.005 -0.189 -0.187 0 -0.042 -0.048 -0.093 0 0 0.025 0 0.084 -0.661 0.066 -0.122 0.082

6 1 -0.187 0.002 -0.028 -0.06 -0.17 0 -0.075 0.148 0 -0.005 -0.189 -0.187 0 -0.042 -0.198 -0.093 0 0 0.025 -0.096 0.084 -0.661 0.066 -0.122 0.082

6 1 -0.187 0.002 -0.028 -0.06 -0.17 0 -0.075 0.148 0 -0.005 -0.189 -0.187 0 -0.042 -0.048 -0.093 0 0 0.025 0 0.084 -0.661 0.066 -0.131 0.082

6 1 -0.187 0.002 -0.028 -0.06 -0.17 0 -0.075 0.114 0 0 -0.189 -0.125 0 -0.042 -0.048 -0.107 0 0 0.025 0 0.084 -0.48 -0.039 0.02 0.082

6 1 -0.187 0.002 -0.028 -0.06 -0.097 0 -0.099 0.148 0 -0.005 -0.189 -0.187 0 -0.042 -0.048 -0.093 0 0 0.025 -0.096 0.084 -0.661 0.066 -0.122 0.082

6 1 -0.187 0.014 -0.028 -0.06 -0.17 0.076 -0.075 0.148 0 -0.127 -0.189 -0.187 0 -0.029 -0.048 -0.093 0 0 0.025 0 0.084 -0.661 0.066 -0.122 0.082

6 1 -0.187 0.002 -0.028 -0.06 -0.17 0 -0.075 0.148 0 -0.005 -0.189 -0.187 0 -0.042 0.026 -0.093 0 0 0.025 -0.096 0.084 -0.661 0.066 -0.122 0.082

7 932 -0.023 0.129 0.101 0 0.012 0.064 -0.026 -0.169 0.172 0 -0.077 0.004 0.207 0.161 -0.084 0 0.005 0 0.134 0 -0.106 0.043 0 0.043 0

7 450 -0.023 0.129 0.101 0 0.012 0.064 -0.026 -0.169 0.172 0 -0.077 0 0.207 0.161 -0.179 0 0.005 0 0.134 0 -0.106 0.043 0 0.043 0

7 335 -0.023 0.129 0.101 0 0.012 0.064 -0.026 -0.169 0.172 0 -0.077 0 0.207 0.161 -0.084 0 0.005 0 0.134 0 -0.106 0.043 0 0.043 0

7 269 -0.023 0.129 0.19 0 0.012 0.064 -0.026 -0.169 0.172 0 -0.051 0.004 0.207 0.161 -0.084 0 0.005 0 0.134 0 -0.106 0.043 -0.105 0.043 0

7 213 -0.023 0.129 0.101 0 0.012 0.064 -0.026 -0.169 0.172 0 -0.077 0.049 0.207 0.161 -0.179 0 0.005 0 0.134 0 -0.106 0.043 0 0.043 0

7 131 -0.023 0.129 0.101 0 0.012 0.064 -0.026 -0.169 0.172 0 -0.077 0 0.207 0.161 -0.084 0 0.005 0 0.134 0 -0.106 0.043 0 -0.099 0

7 87 -0.023 0.129 0.101 0 0.012 0.064 -0.026 -0.169 0.177 0 -0.077 0.049 0.207 0.161 -0.179 0 0.005 0 0.134 0 -0.106 0.043 0 0.043 0

7 60 -0.023 0.129 0.19 0 0.012 0.064 -0.026 -0.169 0.172 0 -0.051 0.004 0.207 0.161 -0.084 0 -0.036 0 0.134 0 -0.106 0.043 -0.105 0.043 0

7 53 -0.023 0.129 0.101 0 0.012 0.064 -0.026 -0.14 0.172 0 -0.077 0 0.207 0.161 -0.179 0 0.005 0 0.134 0 -0.106 0.043 0 0.043 0

7 38 -0.023 0.129 0.101 0 0.012 0.064 -0.026 -0.169 0.172 0 -0.077 0.004 0.207 0.161 -0.084 0 0.005 0 0.134 0 -0.106 0.043 0.01 0.043 0

7 30 -0.023 0.129 0.101 0 0.012 0.064 -0.026 -0.169 0.172 0 -0.077 0.004 0.207 0.161 -0.084 0 0.005 0 0.134 0 -0.106 0.043 0.015 0.043 0

7 24 -0.023 0.129 0.101 0 0.202 0.064 -0.026 -0.169 0.172 0 -0.077 0 0.207 0.161 -0.179 0 0.005 0 0.134 0 -0.106 0.043 0 0.043 0

7 22 -0.023 0.129 0.101 0 0.012 0.064 -0.026 -0.169 0.172 0 -0.077 0 0.207 0.161 -0.084 0 0.129 0 0.134 0 -0.106 0.043 0 0.043 0

7 17 -0.023 0.129 0.101 0 0.012 0.064 -0.026 -0.169 0.172 0 -0.077 0.004 0.207 0.161 -0.137 0 0.005 0 0.134 0 -0.106 0.043 0 0.043 0

7 15 -0.023 0.129 0.19 0 0.012 0.064 -0.026 -0.169 0.172 0.108 -0.051 0.004 0.207 0.161 -0.084 0 0.005 0 0.134 0 -0.106 0.043 -0.105 0.043 0

7 12 -0.023 0.129 0.101 0 0.012 0.064 -0.026 -0.169 0.166 0 -0.077 0 0.207 0.161 -0.084 0 0.005 0 0.134 0 -0.106 0.043 0 0.043 0

7 8 -0.023 0.129 0.101 0 0.012 0.064 -0.026 -0.169 0.172 0 -0.077 0 0.207 0.161 -0.179 0 0.005 0 0.134 -0.063 -0.106 0.043 0 0.043 0

7 8 -0.023 0.129 0.101 0 0.012 0.064 -0.026 -0.169 0.172 0 -0.077 0.004 0.207 0.161 -0.084 0 0.005 0 0.134 0 -0.106 0.043 0.137 0.043 0

7 8 -0.023 0.129 0.101 0 0.012 0.064 -0.026 -0.169 0.172 0 -0.077 0 0.207 0.161 -0.084 0 0.129 0 0.134 -0.167 -0.106 0.043 0 0.043 0

7 7 -0.023 0.129 0.101 0 0.012 0.064 -0.026 -0.169 0.172 0 -0.077 0 0.207 0.161 -0.179 0 0.005 0 0.134 -0.049 -0.106 0.043 0 0.043 0

7 6 -0.023 0.129 0.101 0 0.012 0.064 -0.026 -0.169 0.172 0 -0.077 0.004 0.207 0.161 -0.059 0 0.005 0 0.134 0 -0.106 0.043 0 0.085 0

7 6 -0.023 0.129 0.19 0 0.012 0.064 -0.026 -0.169 0.172 0 -0.051 0.004 0.207 0.161 -0.084 0 0.005 -0.06 0.134 0 -0.106 0.043 -0.105 0.043 0

7 6 -0.023 0.129 0.101 0 0.012 0.064 -0.026 -0.169 0.177 0 -0.077 0.049 0.207 0.287 -0.179 0 0.005 0 0.134 0 -0.106 0.043 0 0.043 0

7 6 -0.023 0.129 0.101 0 0.012 0.064 -0.026 -0.169 0.172 0 -0.077 0 0.207 0.161 -0.179 0.032 0.005 0 0.134 0 -0.106 0.043 0 0.043 0

7 4 -0.023 0.129 -0.24 0 0.012 0.064 -0.026 -0.169 0.172 0 -0.077 0 0.207 0.161 -0.084 0 0.005 0 0.134 0 -0.106 0.043 0 0.043 0

7 4 -0.023 0.129 0.101 0 0.012 0.064 -0.026 -0.169 0.172 0 -0.077 0.049 0.207 0.161 -0.179 0 0.005 0 0.134 0 -0.106 0.096 0 0.043 0

7 4 -0.023 0.129 0.101 0 0.012 0.064 -0.026 -0.169 0.172 0 -0.077 0 0.11 0.161 -0.084 0 0.129 0 0.134 0 -0.106 0.043 0 0.043 0

7 4 -0.023 0.129 0.101 0 0.012 0.064 -0.026 -0.255 0.172 0 -0.077 0.004 0.207 0.161 -0.084 0 0.005 0 0.134 0 -0.106 0.043 0 0.043 0

7 4 -0.023 0.129 0.101 0 0.012 0.064 -0.026 -0.169 0.172 0 -0.077 0.004 0.207 0.161 -0.084 0 0.005 0 0.138 0 -0.106 0.043 0 0.043 0

7 4 -0.023 0.129 0.101 0 0.012 0.064 -0.026 -0.169 0.172 0 -0.077 0 0.207 0.161 -0.072 0 0.005 0 0.134 0 -0.106 0.043 0 0.043 0

7 4 -0.023 0.129 0.101 0 0.012 0.006 -0.026 -0.169 0.172 0 -0.077 0 0.207 0.161 -0.084 0 0.005 0 0.134 0 -0.106 0.043 0 0.043 0

7 4 -0.023 0.129 0.101 0 0.012 0.064 -0.204 -0.14 0.172 0 -0.077 0 0.207 0.161 -0.179 0 0.005 0 0.134 0 -0.106 0.043 0 0.043 0

7 4 -0.023 0.129 0.101 0 0.012 0.064 -0.026 -0.169 0.172 0 -0.077 0 0.207 0.161 -0.084 0 0.09 0 0.134 0 -0.106 0.043 0 -0.099 0

7 4 -0.023 0.129 0.101 0 0.012 0.064 -0.026 -0.169 0.172 0 -0.077 0 0.207 0.295 -0.179 0 0.005 0 0.134 0 -0.106 0.043 0 0.043 0

7 3 -0.023 0.129 0.101 0 0.012 -0.006 -0.026 -0.169 0.172 0 -0.077 0 0.207 0.161 -0.084 0 0.005 0 0.134 0 -0.106 0.043 0 0.043 0

7 3 -0.023 0.129 0.101 0 0.202 0.064 -0.026 -0.169 0.172 0 -0.077 0 0.207 0.161 -0.179 0 0.005 0 0.134 0 -0.103 0.043 0 0.043 0

7 3 -0.023 0.129 0.101 0 0.012 0.064 -0.026 -0.169 0.172 0 -0.077 0.004 0.207 0.205 -0.084 0 0.005 0 0.134 0 -0.106 0.043 0 0.043 0

7 3 -0.023 0.129 0.101 0 0.012 0.064 -0.026 -0.169 0.172 0 -0.077 0 0.207 0.161 -0.179 0 0.005 0 0.134 0 -0.106 0.043 0 -0.011 0

7 3 -0.023 0.129 0.101 0.08 0.012 0.064 -0.026 -0.169 0.172 0 -0.077 0.004 0.207 0.161 -0.084 0 0.005 0 0.134 0 -0.106 0.043 0 0.043 0

7 2 -0.023 0.129 0.101 0 0.012 0.064 -0.026 -0.169 0.177 0 -0.077 0.049 0.207 0.302 -0.179 0 0.005 0 0.134 0 -0.106 0.043 0 0.043 0

7 2 -0.023 0.129 0.101 0 0.012 0.064 -0.026 -0.169 0.172 0 -0.077 0.004 0.207 0.161 -0.084 0 0.005 -0.022 0.134 0 -0.106 0.043 0 0.043 0

7 2 -0.023 0.129 0.101 0 0.012 0.064 -0.026 -0.169 0.172 0 -0.077 0.004 0.207 0.145 -0.084 0 0.005 0 0.134 0 -0.106 0.043 0 0.043 0

7 2 -0.023 0.129 0.101 0 0.012 0.064 -0.026 -0.169 0.172 0 -0.077 0.004 0.207 0.161 -0.137 0 0.005 0 0.134 0 -0.106 0.134 0 0.043 0

7 2 -0.023 0.129 0.101 0 0.012 0.064 -0.026 -0.169 0.172 0 -0.077 0 0.207 0.161 -0.179 -0.055 0.005 0 0.134 0 -0.106 0.043 0 0.043 0

7 2 -0.023 0.129 0.101 0 0.012 0.064 -0.026 -0.169 0.172 -0.078 -0.077 0.004 0.207 0.161 -0.084 0 0.005 0 0.134 0 -0.106 0.043 0 0.043 0

7 2 -0.023 0.129 0.101 0 0.012 0.064 -0.026 -0.169 0.172 0 -0.077 0.004 0.207 0.161 -0.084 0 0.005 0 0.134 0 -0.106 0.043 0 0.131 0

7 2 -0.023 0.129 0.103 0 0.012 0.064 -0.026 -0.169 0.172 0 -0.077 0 0.207 0.161 -0.179 0 0.005 0 0.134 0 -0.106 0.043 0 0.043 0

7 2 -0.023 0.129 0.19 0 0.012 0.064 -0.026 -0.169 0.172 0 -0.051 0.004 0.207 0.161 -0.084 0 0.005 0 0.134 0 -0.096 0.043 -0.105 0.043 0

7 2 -0.023 0.129 0.101 0 0.012 0.064 -0.026 -0.169 0.172 0 -0.077 0.004 0.207 0.161 -0.084 0 -0.094 0 0.134 0 -0.106 0.043 0 0.043 0

7 2 -0.023 0.129 0.101 0 0.012 0.064 -0.026 -0.169 0.172 0 -0.077 0.004 0.123 0.161 -0.084 0 0.005 0 0.134 0 -0.106 0.043 0 0.043 0

7 2 -0.023 0.215 0.101 0 0.202 0.064 -0.026 -0.169 0.172 0 -0.077 0 0.207 0.161 -0.179 0 0.005 0 0.134 0 -0.106 0.043 0 0.043 0

7 2 -0.023 -0.069 0.101 0 0.012 0.064 -0.026 -0.169 0.172 0 -0.077 0 0.207 0.164 -0.179 0 0.005 0 0.134 0 -0.106 0.043 0 0.043 0

7 2 -0.023 0.129 0.101 0 0.012 0.064 -0.026 -0.169 0.172 0 -0.077 0.004 0.207 0.161 -0.084 0 0.005 0 0.134 0 -0.124 0.043 0 0.043 0

7 2 -0.023 0.129 0.101 0 0.037 0.064 -0.026 -0.169 0.172 0 -0.077 0.004 0.207 0.161 -0.084 0 0.005 0 0.134 0 -0.106 0.043 0 0.043 0

7 2 -0.023 0.129 0.101 0 0.202 0.124 -0.026 -0.169 0.172 0 -0.077 0 0.207 0.161 -0.179 0 0.005 0 0.134 0 -0.106 0.043 0 0.043 0

7 2 -0.023 0.129 0.101 0 0.012 0.064 -0.026 -0.169 0.177 0 -0.077 0.049 0.207 0.161 -0.179 -0.157 0.005 0 0.134 0 -0.106 0.043 0 0.043 0

7 2 -0.023 0.121 0.101 0 0.012 0.064 -0.026 -0.169 0.172 0 -0.077 0 0.207 0.161 -0.179 0 0.005 0 0.134 0 -0.106 0.043 0 0.043 0

7 2 -0.023 0.129 0.101 0 0.012 0.064 -0.026 -0.169 0.172 0 -0.077 0.004 0.207 0.161 -0.084 0.033 0.005 0 0.134 0 -0.106 0.043 0 0.043 0

7 2 -0.023 0.129 0.101 0 0.012 0.064 -0.026 -0.169 0.172 0 -0.077 0.004 0.207 0.161 -0.084 0 0.005 0 0.134 0 -0.106 0.043 0 0.043 0.026

7 2 -0.023 0.129 0.101 0 0.012 0.064 -0.026 -0.169 0.172 -0.066 -0.077 0.004 0.207 0.161 -0.084 0 0.005 0 0.134 0 -0.106 0.043 0.01 0.043 0

7 2 -0.023 0.129 0.101 0 0.012 0.064 -0.026 -0.169 0.172 0 -0.077 0.004 0.207 0.076 -0.084 0 0.005 0 0.134 0 -0.106 0.043 0.01 0.043 0

7 2 -0.023 0.129 0.101 0 -0.05 0.064 -0.026 -0.169 0.172 0 -0.077 0.049 0.207 0.161 -0.179 0 0.005 0 0.134 0 -0.106 0.043 0 0.043 0

7 2 -0.023 0.129 0.101 0 0.012 0.064 -0.026 -0.169 0.172 0 -0.077 0 0.207 0.161 -0.084 0 -0.07 0 0.134 0 -0.106 0.043 0 -0.099 0

7 2 -0.023 0.129 0.101 0 0.012 0.064 -0.097 -0.169 0.172 0 -0.077 0 0.207 0.161 -0.084 0 0.005 0 0.134 0 -0.106 0.043 0 0.043 0

7 2 -0.023 0.129 0.19 0 0.012 0.371 -0.026 -0.169 0.172 0 -0.051 0.004 0.207 0.161 -0.084 0 0.005 0 0.134 0 -0.106 0.043 -0.105 0.043 0

7 2 -0.023 0.129 0.101 0 0.012 0.064 -0.026 -0.169 0.172 0 -0.077 0 0.207 0.161 -0.084 0 0.005 0.033 0.134 0 -0.106 0.043 0 0.043 0

7 3 -0.023 0.129 0.101 0 0.012 0.064 -0.026 -0.169 0.172 0 -0.077 0.004 0.207 0.161 -0.084 0 0.005 0 0.134 0 -0.106 0.158 0 0.043 0

7 2 -0.023 0.129 0.101 0 0.012 0.094 -0.026 -0.169 0.172 0 -0.077 0.049 0.207 0.161 -0.179 0 0.005 0 0.134 0 -0.106 0.043 0 0.043 0

7 1 -0.001 0.129 0.19 0 0.012 0.064 -0.026 -0.169 0.172 0 -0.051 0.004 0.207 0.161 -0.084 0 0.005 0 0.134 0 -0.106 0.043 -0.105 0.043 0

7 1 -0.023 0.129 0.101 0 0.012 0.078 -0.026 -0.169 0.172 0 -0.077 0 0.207 0.161 -0.179 0 0.005 0 0.134 0 -0.106 0.043 0 0.043 0

7 1 -0.023 0.129 -0.041 0 0.012 0.064 -0.026 -0.169 0.172 0 -0.077 0.004 0.207 0.161 -0.059 0 0.005 0 0.134 0 -0.106 0.043 0 0.085 0

7 1 -0.023 0.129 0.101 0 0.012 -0.021 -0.026 -0.169 0.172 0 -0.077 0.004 0.207 0.161 -0.059 0 0.005 0 0.134 0 -0.106 0.043 0 0.085 0

7 1 -0.023 0.129 0.101 0 0.012 0.064 -0.026 -0.169 0.172 0 -0.077 0 0.207 0.161 -0.179 0.045 0.005 0 0.134 0 -0.106 0.043 0 0.043 0

7 1 -0.023 0.129 0.101 0 0.012 0.064 -0.026 -0.169 0.172 0 -0.077 0 0.207 0.161 -0.084 0 0.005 0 0.134 0 -0.106 -0.116 0 0.043 0

7 1 -0.023 0.129 0.101 0 0.012 0.064 -0.026 -0.169 0.172 0 -0.077 0 0.207 0.161 -0.179 0 0.005 0 0.134 0 -0.106 0.043 0 0.043 -0.164

7 1 -0.023 0.129 0.101 0 0.012 0.064 -0.026 -0.169 0.172 0 -0.096 0.004 0.207 0.161 -0.084 0 0.005 0 0.134 0 -0.106 0.043 0 0.043 0

7 1 -0.023 0.129 0.101 0 0.012 0.064 -0.026 -0.169 0.172 0 -0.077 0 0.207 0.161 -0.084 0 0.005 0 0.134 0 0.065 0.043 0 0.043 0

7 1 -0.023 0.129 0.101 0 0.012 0.064 -0.026 -0.169 0.172 0 -0.077 0.004 0.207 0.161 -0.084 0 0.005 0 0.134 0 -0.106 0.054 0 0.043 0

7 1 -0.023 0.129 0.101 0.064 0.012 0.064 -0.026 -0.169 0.172 0 -0.077 0.004 0.207 0.161 -0.084 0 0.005 0 0.134 0 -0.106 0.043 0 0.043 0

7 1 -0.023 0.129 0.101 0 0.202 0.064 0.059 -0.169 0.172 0 -0.077 0 0.207 0.161 -0.179 0 0.005 0 0.134 0 -0.106 0.043 0 0.043 0

7 1 -0.023 0.129 0.101 0 0.012 0.064 -0.026 -0.169 0.172 0 -0.077 -0.077 0.207 0.161 -0.084 0 0.005 0 0.134 0 -0.106 0.043 0 0.043 0

7 1 -0.029 0.129 0.19 0 0.012 0.064 -0.026 -0.169 0.172 0 -0.051 0.004 0.207 0.161 -0.084 0 0.005 0 0.134 0 -0.106 0.043 -0.105 0.043 0

7 1 -0.023 0.129 0.101 0 0.012 0.064 -0.026 -0.169 0.22 0 -0.077 0 0.207 0.161 -0.179 0 0.005 0 0.134 -0.049 -0.106 0.043 0 0.043 0

7 1 -0.023 0.129 0.101 0 0.012 0.064 -0.026 -0.169 0.172 0 -0.077 0 0.207 0.161 -0.084 0 0.005 0 0.134 -0.179 -0.106 0.043 0 0.043 0

7 1 -0.023 0.129 0.19 0 0.012 0.064 -0.026 -0.169 0.172 0 -0.051 0.004 0.207 0.161 -0.316 0 0.005 0 0.134 0 -0.106 0.043 -0.105 0.043 0

7 1 0.057 0.129 0.243 0 0.012 0.064 -0.026 -0.169 0.172 0 -0.077 0 0.207 0.161 -0.084 0 0.005 0 0.134 0 -0.106 0.043 0 0.043 0

7 1 -0.023 0.129 0.101 -0.182 0.012 0.064 -0.026 -0.169 0.172 0 -0.077 0 0.207 0.161 -0.084 0 0.005 0 0.134 0 -0.106 0.043 0 0.043 0

7 1 -0.023 0.129 0.101 0 0.012 0.064 -0.022 -0.169 0.172 0 -0.077 0 0.207 0.161 -0.084 0 0.005 0 0.134 0 -0.106 0.043 0 0.043 0

7 1 -0.023 0.129 0.101 0.061 0.012 0.064 -0.026 -0.169 0.172 0 -0.077 0 0.207 0.161 -0.084 0 0.005 0 0.134 0 -0.106 0.043 0 -0.099 0

7 1 -0.023 0.129 0.101 0 0.012 0.064 -0.026 -0.169 0.172 -0.011 -0.077 0 0.207 0.161 -0.179 0 0.005 0 0.134 0 -0.106 0.043 0 -0.011 0

7 1 -0.023 0.129 0.101 0.136 0.012 0.064 -0.026 -0.169 0.172 0 -0.077 0.004 0.207 0.161 -0.084 0 0.005 0 0.134 0 -0.106 0.043 0 0.043 0

7 1 -0.023 0.129 0.101 0 0.012 0.064 -0.026 -0.169 0.172 0 -0.077 0.004 0.207 0.161 -0.084 0 0.005 0 0.134 0 -0.106 -0.009 0 0.043 0

7 1 -0.023 0.129 0.101 0 0.012 0.064 -0.026 -0.169 0.172 0 -0.077 0 0.207 0.161 -0.179 0 0.005 0 0.134 0 0.134 0.043 0 0.043 0

7 1 -0.023 0.129 0.19 0 0.012 0.064 -0.026 -0.169 0.172 0 -0.051 0.004 0.262 0.161 -0.084 0 -0.036 0 0.134 0 -0.106 0.043 -0.105 0.043 0

7 1 -0.023 0.129 0.19 0 0.012 0.064 -0.026 -0.169 0.172 -0.014 -0.051 0.004 0.207 0.161 -0.084 0 0.005 0 0.134 0 -0.106 0.043 -0.105 0.043 0

7 1 -0.023 0.129 0.101 0 0.012 0.064 -0.026 -0.169 0.172 0 -0.077 0.004 0.207 0.323 -0.084 0 0.005 0 0.134 0 -0.106 0.043 0 0.043 0

7 1 -0.023 0.129 0.101 0 0.012 0.064 -0.026 -0.169 0.172 0 -0.077 0.004 0.207 0.161 -0.084 0 0.005 0 0.134 -0.026 -0.106 0.043 0 0.043 0

7 1 -0.023 0.129 0.101 0 0.012 0.064 -0.026 -0.169 0.172 0 -0.077 0.004 0.207 0.161 -0.084 0 0.005 0 0.134 0 -0.106 0.043 0.098 0.043 0

7 1 -0.023 0.129 0.101 0 0.012 0.064 -0.026 -0.169 0.172 0 -0.077 0.004 0.207 0.161 -0.084 0 0.005 0 0.134 0 -0.106 0.043 0 -0.044 0

7 1 -0.023 0.129 0.101 0 0.012 0.064 -0.026 -0.169 0.172 0 -0.077 0.004 0.207 0.161 -0.084 0 0.005 0 0.134 0 -0.106 0.043 0 -0.04 0

7 1 -0.023 0.129 0.19 0 0.012 0.064 -0.026 -0.169 0.172 0 -0.051 0.004 0.207 0.161 -0.084 0 0.005 0 0.134 0 -0.106 0.043 -0.105 0.043 0.086

7 1 -0.023 0.129 0.101 0 0.012 0.064 -0.026 -0.169 0.172 0 -0.077 0.004 0.207 0.161 -0.084 0 0.005 0.031 0.134 0 -0.106 0.043 0 0.043 0

7 1 -0.023 0.129 0.101 0 0.012 0.064 -0.026 -0.169 0.172 0 -0.077 0 0.207 0.161 -0.179 0 0.005 0 0.134 0 -0.142 0.043 0 0.1 0

7 1 -0.023 0.129 0.101 0 0.012 0.064 -0.026 -0.169 0.172 0 -0.077 0.004 0.207 0.161 -0.084 0 0.005 0 0.134 0 -0.106 0.033 0 0.043 0

7 1 -0.023 0.129 0.101 0 0.012 0.064 -0.026 -0.14 0.172 0 -0.077 0 0.207 0.161 -0.179 0 0.005 0 0.134 0 -0.106 0.168 0 0.043 0

7 1 -0.023 0.129 0.101 0 0.012 0.064 -0.026 -0.169 0.172 0 -0.077 -0.071 0.207 0.161 -0.179 0 0.005 0 0.134 0 -0.106 0.043 0 0.043 0

7 1 -0.023 0.129 0.19 0 0.012 0.064 -0.026 -0.11 0.172 0 -0.051 0.004 0.207 0.161 -0.084 0 -0.036 0 0.134 0 -0.106 0.043 -0.105 0.043 0

7 1 -0.023 0.129 0.101 0 0.012 0.064 -0.026 -0.169 0.172 0 -0.077 0.049 0.207 0.161 -0.179 -0.035 0.005 0 0.134 0 -0.106 0.043 0 0.043 0

7 1 -0.023 0.129 0.19 0 0.012 0.064 -0.026 -0.169 0.031 0 -0.051 0.004 0.207 0.161 -0.084 0 0.005 0 0.134 0 -0.106 0.043 -0.105 0.043 0

8 459 0.291 -0.052 -0.028 -0.105 0 0 -0.331 0.108 -0.012 0.149 -0.088 -0.173 0.07 -0.111 0.034 -0.029 0.124 0 -0.125 -0.042 0.047 -0.111 -0.128 -0.116 0

8 732 0.291 -0.052 -0.028 -0.12 0 0 -0.25 0.108 -0.012 0.157 -0.088 -0.173 0.07 -0.052 0.034 -0.029 0.124 0 0 0 0.047 -0.111 -0.128 -0.116 0

8 441 0.291 -0.052 -0.028 -0.12 0 0 -0.25 0.108 -0.012 0.179 -0.088 -0.173 0.07 -0.111 0.034 -0.029 0.124 0 0 0 0.047 -0.111 -0.087 -0.116 0

8 327 0.291 -0.066 -0.028 -0.105 0 0 -0.331 0.108 -0.012 0.179 -0.088 -0.173 0.07 -0.034 0.034 -0.029 0.124 0 0 0 0.047 -0.111 -0.266 -0.116 0

8 259 0.291 -0.052 -0.028 -0.105 0 0 -0.25 0.108 -0.012 0.179 -0.088 -0.173 0.07 -0.293 0.034 -0.029 0.124 0 0 0 0.047 -0.111 -0.128 -0.103 0

8 193 0.291 -0.066 -0.028 -0.105 0 0 -0.331 0.108 -0.012 0.179 -0.088 -0.173 0.07 -0.034 0.034 -0.029 0.124 0 0 0 0.047 -0.111 -0.128 -0.116 0.012

8 160 0.291 -0.052 -0.028 -0.12 0 0 -0.378 0.108 -0.012 0.179 -0.088 -0.173 0.07 -0.111 0.034 -0.029 0.124 0 0 0 0.047 -0.057 -0.087 -0.116 0

8 157 0.291 -0.052 -0.028 -0.105 0 0 -0.25 0.134 -0.012 0.179 -0.088 -0.129 0.07 -0.111 0.034 -0.029 0.053 0 0 -0.035 0.047 -0.157 -0.128 -0.116 0

8 126 0.291 -0.052 -0.028 -0.12 0 0 -0.25 0.108 -0.012 0.157 0.035 -0.173 0.07 -0.052 0.034 -0.029 0.124 0 0 0 0.047 -0.111 -0.128 -0.116 0

8 114 0.291 -0.066 -0.028 -0.105 0 0 -0.331 0.108 -0.012 0.179 -0.088 -0.176 0.07 -0.111 0.034 -0.029 0.124 0.118 0 0 0.047 -0.111 -0.128 -0.116 0

8 112 0.291 -0.052 -0.028 -0.105 0 0 -0.25 0.15 -0.012 0.179 -0.088 -0.173 0.07 -0.293 0.034 -0.029 0.124 0 0 0 0.047 -0.111 -0.128 -0.103 0

8 118 0.291 -0.052 -0.028 -0.12 0 0 -0.378 0.108 -0.012 0.179 -0.088 -0.173 0.07 -0.111 0.034 0.085 0.124 0 0 0 0.047 -0.111 -0.087 -0.116 0

8 97 0.291 -0.052 -0.028 -0.12 0 0 -0.25 0.108 -0.012 0.157 -0.088 -0.173 0.07 -0.052 0.053 -0.029 0.124 0 -0.009 0 0.047 -0.111 -0.128 -0.116 0

8 90 0.291 -0.066 -0.028 -0.105 0 0 -0.331 0.108 -0.012 0.179 -0.088 -0.176 0.07 -0.111 0.034 -0.029 0.124 0.118 0 0 0.047 -0.111 -0.175 -0.116 0

8 53 0.291 -0.052 -0.028 -0.105 0 0 -0.25 0.134 -0.012 0.179 -0.088 -0.129 0.07 -0.111 0.034 -0.029 0.053 0 0 -0.035 0.023 -0.157 -0.128 -0.116 -0.024

8 70 0.291 -0.066 -0.028 -0.015 0 0 -0.331 0.108 -0.012 0.179 -0.088 -0.176 0.07 -0.111 0.034 -0.029 0.124 0.118 0 0 0.047 -0.111 -0.128 -0.116 0

8 29 0.291 -0.052 -0.028 -0.12 -0.07 0 -0.25 0.264 -0.012 0.157 -0.088 -0.173 0.07 -0.052 0.034 -0.029 0.124 0 0 0 0.047 -0.111 -0.128 -0.116 0

8 29 0.291 -0.052 -0.028 -0.12 0 0 -0.25 0.108 -0.012 0.157 -0.088 -0.173 0.07 -0.052 0.034 -0.029 0.124 0 0 0 0.047 -0.111 -0.128 -0.056 0

8 25 0.291 -0.052 -0.028 -0.12 0 0 -0.25 0.108 -0.012 0.179 -0.088 -0.173 0.07 -0.111 0.034 -0.029 0.124 0.108 0 0 0.047 -0.111 -0.087 -0.116 0

8 23 0.291 -0.052 -0.028 -0.12 -0.112 0 -0.25 0.108 -0.012 0.157 -0.088 -0.173 0.07 -0.052 0.034 -0.029 0.124 0 0 0 0.047 -0.111 -0.128 -0.116 0

8 20 0.291 -0.052 -0.028 -0.12 0 0 -0.25 -0.016 -0.012 0.179 -0.088 -0.173 0.07 -0.111 0.034 -0.029 0.124 0 0 0 0.047 -0.111 -0.087 -0.116 0

8 17 0.291 -0.066 -0.028 -0.105 0 0 -0.331 0.108 -0.012 0.179 -0.088 -0.173 0.07 -0.034 0.034 -0.029 0.124 0 0 0 0.047 -0.111 -0.088 -0.116 0.012

8 16 0.291 -0.052 -0.028 -0.105 0 0 -0.347 0.108 -0.012 0.149 -0.088 -0.173 0.07 -0.111 0.034 -0.029 0.124 0 -0.125 -0.042 0.047 -0.111 -0.128 -0.116 0

8 16 0.291 -0.052 -0.028 -0.12 0 0 -0.25 0.039 -0.012 0.157 -0.088 -0.173 0.07 -0.052 0.034 -0.029 0.124 0 0 0 0.047 -0.111 -0.128 -0.116 0

8 15 0.291 -0.052 -0.028 -0.12 0 0 -0.25 0.108 -0.012 0.157 0.035 -0.173 0.07 -0.052 0.034 -0.029 0.124 0 0 0 0.047 -0.111 -0.128 -0.116 0.232

8 14 0.291 -0.052 -0.028 -0.105 0.019 0 -0.331 0.108 -0.012 0.149 -0.088 -0.173 0.07 -0.111 0.034 -0.029 0.124 0 -0.125 -0.042 0.047 -0.111 -0.128 -0.116 0

8 14 0.291 -0.052 -0.028 -0.12 0 0 -0.327 0.108 -0.012 0.157 -0.088 -0.173 0.07 -0.052 0.034 -0.029 0.124 0 0 0 0.047 -0.111 -0.128 -0.116 0

8 12 0.291 -0.066 -0.028 -0.105 0 0 -0.331 0.108 -0.012 0.179 -0.088 -0.173 0.024 -0.034 0.034 -0.029 0.124 0 0 0 0.047 -0.111 -0.266 -0.116 0

8 11 0.291 -0.052 -0.028 -0.12 0 0 -0.48 0.108 -0.012 0.179 -0.088 -0.173 0.07 -0.111 0.034 -0.029 0.124 0 0 0 0.047 -0.057 -0.087 -0.116 0

8 11 0.291 -0.052 -0.028 -0.12 0 0 -0.25 0.108 -0.012 0.157 -0.088 -0.173 0.07 -0.052 0.034 -0.029 0.124 0.08 0 0 0.047 -0.111 -0.128 -0.116 0

8 10 0.291 -0.052 -0.038 -0.12 0 0 -0.25 0.108 -0.012 0.157 -0.088 -0.173 0.1 -0.052 0.034 -0.029 0.124 0 0 0 0.047 -0.111 -0.128 -0.116 0

8 10 0.291 -0.021 -0.028 -0.105 0 0 -0.331 0.108 -0.012 0.149 -0.088 -0.173 0.07 -0.111 0.034 -0.029 0.124 0 -0.125 -0.042 0.047 -0.111 -0.128 -0.116 0

8 9 0.291 -0.052 -0.038 -0.12 0 0 -0.25 0.108 -0.012 0.157 -0.088 -0.173 0.07 -0.052 0.034 -0.029 0.124 0 0 0 0.047 -0.111 -0.128 -0.116 0

8 8 0.291 -0.066 -0.028 -0.105 0 0 -0.331 0.108 -0.012 0.179 -0.088 -0.176 0.07 -0.111 0.034 -0.029 0.108 0.118 0 0 0.047 -0.111 -0.128 -0.116 0

8 8 0.291 -0.052 -0.028 -0.105 0 0 -0.25 0.134 -0.012 0.179 -0.088 -0.129 0.07 -0.111 0.034 -0.029 0.053 0 0 -0.035 0.047 -0.157 -0.128 -0.116 0.036

8 8 0.291 -0.052 -0.028 -0.105 0 0 -0.25 0.134 -0.012 0.179 -0.088 -0.129 0.07 -0.111 0.034 -0.029 0.053 0 0 -0.035 0.047 -0.157 -0.128 -0.116 0.117

8 7 0.291 -0.052 -0.028 -0.12 0 0 -0.25 0.108 -0.012 0.157 -0.088 -0.173 0.07 -0.199 0.034 -0.029 0.124 0 0 0 0.047 -0.111 -0.128 -0.116 0

8 6 0.291 -0.066 -0.028 -0.105 0 0 -0.331 0.108 -0.012 0.179 -0.103 -0.173 0.07 -0.034 0.034 -0.029 0.124 0 0 0 0.047 -0.111 -0.128 -0.116 0.012

8 6 0.291 -0.052 -0.028 -0.105 0 0 -0.25 0.134 -0.012 0.179 -0.088 -0.129 0.07 -0.111 0.034 -0.029 0.053 0.093 0 -0.035 0.023 -0.157 -0.128 -0.116 -0.024

8 6 0.291 -0.066 -0.028 -0.105 0 0 -0.331 0.108 -0.012 0.179 -0.088 -0.173 0.07 -0.034 0.034 -0.029 0.147 0 0 0 0.047 -0.111 -0.266 -0.116 0

8 6 0.291 -0.052 -0.028 -0.12 0 0 -0.25 0.108 -0.012 0.157 -0.088 -0.173 0.07 -0.052 0.053 -0.029 0.124 0 -0.009 0 0.202 -0.111 -0.128 -0.116 0

8 5 0.291 -0.066 -0.028 -0.105 0 0 -0.331 0.108 -0.012 0.241 -0.088 -0.173 0.07 -0.034 0.034 -0.029 0.124 0 0 0 0.047 -0.11 -0.266 -0.116 0

8 5 0.291 -0.052 -0.028 -0.105 0 0 -0.25 0.134 -0.012 0.179 -0.088 -0.129 0.07 -0.111 0.034 -0.029 0.053 -0.019 0 -0.035 0.047 -0.157 -0.128 -0.116 0

8 5 0.291 -0.052 -0.028 -0.07 0 0 -0.25 0.108 -0.012 0.157 -0.088 -0.173 0.07 -0.052 0.034 -0.029 0.124 0 0 0 0.047 -0.111 -0.128 -0.116 0

8 4 0.291 -0.052 -0.028 -0.105 0 0 -0.25 0.134 -0.012 0.179 -0.088 -0.129 0.07 -0.111 0.034 -0.029 0.053 0 0 -0.035 0.059 -0.157 -0.128 -0.116 0

8 4 0.127 -0.052 -0.028 -0.105 0 0 -0.331 0.108 -0.012 0.149 -0.088 -0.173 0.07 -0.111 0.034 -0.029 0.124 0 -0.125 -0.042 0.047 -0.111 -0.128 -0.116 0

8 4 0.291 -0.052 -0.028 -0.105 0 0 -0.25 0.15 -0.012 0.091 -0.088 -0.173 0.07 -0.293 0.034 -0.029 0.124 0 0 0 0.047 -0.111 -0.128 -0.103 0

8 4 0.291 -0.052 -0.076 -0.12 0 0 -0.327 0.108 -0.012 0.157 -0.088 -0.173 0.07 -0.052 0.034 -0.029 0.124 0 0 0 0.047 -0.111 -0.128 -0.116 0

8 4 0.291 -0.052 -0.028 -0.12 0 0 -0.25 0.108 -0.012 0.157 -0.088 -0.173 0.07 -0.052 0.034 -0.029 0.124 0 0 0 0.047 -0.111 -0.128 -0.165 0

8 4 0.291 -0.052 -0.028 -0.105 0 0 -0.25 0.108 -0.012 0.179 -0.088 -0.173 -0.048 -0.293 0.034 -0.029 0.124 0 0 0 0.047 -0.111 -0.128 -0.103 0

8 4 0.291 -0.052 -0.028 -0.12 0 0 -0.25 0.108 -0.012 0.157 -0.088 -0.173 0.07 -0.052 0.034 -0.029 0.124 0 0 0 0.047 -0.111 -0.128 -0.093 0

8 4 0.291 -0.066 -0.028 -0.105 0 0 -0.331 0.108 -0.012 0.296 -0.088 -0.173 0.07 -0.034 0.034 -0.029 0.124 0 0 0 0.047 -0.111 -0.266 -0.116 0

8 4 0.291 -0.052 -0.028 -0.12 0 0 -0.25 0.108 -0.012 0.157 0.035 -0.173 0.07 -0.052 0.034 -0.121 0.124 0 0 0 0.047 -0.111 -0.128 -0.116 0

8 4 0.291 -0.052 -0.028 -0.105 0 0 -0.331 0.108 -0.012 0.149 -0.088 -0.173 0.07 -0.111 0.034 -0.029 0.124 0 -0.125 -0.191 0.047 -0.111 -0.128 -0.116 0

8 4 0.291 -0.019 -0.028 -0.12 0 0 -0.25 0.108 -0.012 0.179 -0.088 -0.173 0.07 -0.111 0.034 -0.029 0.124 0 0 0 0.047 -0.111 -0.087 -0.116 0

8 4 0.291 -0.052 -0.028 -0.144 0 0 -0.25 0.108 -0.012 0.179 -0.088 -0.173 0.07 -0.111 0.034 -0.029 0.124 0 0 0 0.047 -0.111 -0.087 -0.116 0

8 4 0.291 -0.066 -0.028 -0.015 0 0.027 -0.331 0.108 -0.012 0.179 -0.088 -0.176 0.07 -0.111 0.034 -0.029 0.124 0.118 0 0 0.047 -0.111 -0.128 -0.116 0

8 3 0.291 -0.052 -0.028 -0.105 0 0 -0.258 0.108 -0.012 0.179 -0.088 -0.173 0.07 -0.293 0.034 -0.029 0.124 0 0 0 0.047 -0.111 -0.128 -0.103 0

8 3 0.291 -0.052 -0.028 -0.105 0 0 -0.25 0.134 -0.012 0.179 -0.088 -0.129 0.028 -0.111 0.034 -0.029 0.053 0 0 -0.035 0.047 -0.157 -0.128 -0.116 0

8 3 0.291 -0.052 -0.028 -0.105 0 0 -0.25 0.107 -0.012 0.179 -0.088 -0.173 0.07 -0.293 0.034 -0.029 0.124 0 0 0 0.047 -0.111 -0.128 -0.103 0

8 3 0.291 -0.066 -0.028 -0.105 0 0 -0.331 0.108 -0.012 0.179 -0.088 -0.176 -0.021 -0.111 0.034 -0.029 0.124 0.118 0 0 0.047 -0.111 -0.175 -0.116 0

8 3 0.291 -0.052 -0.028 -0.105 0 0.045 -0.331 0.108 -0.012 0.149 -0.088 -0.173 0.07 -0.111 0.034 -0.029 0.124 0 -0.125 -0.042 0.047 -0.111 -0.128 -0.116 0

8 3 0.291 -0.052 -0.028 -0.12 0 0 -0.25 -0.016 -0.012 0.179 -0.088 -0.173 0.07 -0.111 0.034 -0.029 0.124 0 0 0 0.061 -0.111 -0.087 -0.116 0

8 4 0.291 -0.052 -0.028 -0.12 0 0 -0.25 0.108 -0.012 0.179 -0.088 -0.173 0.14 -0.111 0.034 -0.029 0.124 0 0 0 0.047 -0.111 -0.087 -0.116 0

8 3 0.291 -0.052 -0.028 -0.105 0 0 -0.25 0.134 -0.012 0.179 -0.088 -0.129 0.07 -0.111 0.034 -0.029 0.053 0 0 -0.035 0.047 -0.137 -0.128 -0.116 0

8 2 0.291 -0.052 -0.028 -0.12 0.047 0 -0.378 0.108 -0.012 0.179 -0.088 -0.173 0.07 -0.111 0.034 -0.029 0.124 0 0 0 0.047 -0.111 -0.051 -0.116 0

8 2 0.291 0.034 -0.038 -0.12 0 0 -0.25 0.108 -0.012 0.157 -0.088 -0.173 0.07 -0.052 0.034 -0.029 0.124 0 0 0 0.047 -0.111 -0.128 -0.116 0

8 2 0.291 -0.052 -0.028 -0.105 0 0 -0.331 0.108 -0.012 0.149 -0.088 -0.173 0.07 -0.111 0.01 -0.029 0.124 0 -0.125 -0.042 0.047 -0.111 -0.128 -0.116 0

8 2 0.291 -0.052 -0.028 -0.105 0 0 -0.331 0.108 -0.012 0.149 -0.078 -0.173 0.07 -0.111 0.034 -0.029 0.124 0 -0.125 -0.042 0.047 -0.111 -0.128 -0.116 0

8 2 0.291 -0.052 -0.028 -0.12 0 0 -0.25 0.108 -0.012 0.179 -0.088 -0.173 0.07 -0.111 0.034 -0.009 0.124 0 0 0 0.047 -0.111 -0.087 -0.116 0

8 2 0.291 -0.066 -0.028 -0.105 0 0 -0.331 0.108 -0.059 0.179 -0.088 -0.176 0.07 -0.111 0.034 -0.029 0.124 0.118 0 0 0.047 -0.111 -0.128 -0.116 0

8 2 0.291 -0.052 -0.028 -0.12 0 0 -0.168 0.108 -0.012 0.157 -0.088 -0.173 0.07 -0.052 0.034 -0.029 0.124 0 0 0 0.047 -0.111 -0.128 -0.116 0

8 2 0.291 -0.066 -0.028 -0.105 -0.027 0 -0.331 0.108 -0.012 0.179 -0.088 -0.173 0.07 -0.034 0.034 -0.029 0.124 0 0 0 0.047 -0.111 -0.266 -0.116 0

8 2 0.291 -0.052 -0.028 -0.12 -0.07 0 -0.25 0.264 -0.012 0.157 -0.088 -0.173 0.07 -0.052 0.034 -0.029 0.124 0 0 -0.214 0.047 -0.111 -0.128 -0.116 0

8 2 0.291 -0.066 -0.028 -0.105 0 0 -0.331 0.108 -0.012 0.179 -0.088 -0.173 0.07 -0.034 0.034 -0.029 0.124 0 0 -0.033 0.047 -0.111 -0.266 -0.116 0

8 2 0.291 -0.066 -0.028 -0.105 0 0 -0.331 0.108 -0.012 0.179 -0.088 -0.173 0.07 -0.034 0.034 -0.029 0.124 0 0 0 0.047 -0.111 -0.234 -0.116 0.012

8 2 0.291 -0.052 -0.028 -0.12 0 0 -0.25 0.108 -0.012 0.157 -0.088 -0.173 0.07 -0.052 0.034 -0.029 0.124 0 0 0 0.047 0.065 -0.128 -0.116 0

8 2 0.291 -0.052 -0.028 -0.18 0 0 -0.25 0.108 -0.012 0.179 -0.088 -0.173 0.07 -0.111 0.034 -0.029 0.124 0 0 0 0.047 -0.111 -0.087 -0.116 0

8 2 0.291 -0.052 -0.028 -0.12 0 0 -0.378 0.108 -0.012 0.179 -0.088 -0.173 0.07 -0.111 0.127 0.085 0.124 0 0 0 0.047 -0.111 -0.087 -0.116 0

8 2 0.291 -0.052 -0.028 -0.105 0 0 -0.331 0.108 -0.012 0.149 -0.088 -0.173 0.07 -0.111 0.034 -0.029 0.286 0 -0.125 -0.042 0.047 -0.111 -0.128 -0.116 0

8 2 0.291 -0.052 -0.028 -0.12 0 0 -0.25 0.103 -0.012 0.157 -0.088 -0.173 0.07 -0.052 0.034 -0.029 0.124 0 0 0 0.047 -0.111 -0.128 -0.116 0

8 2 0.291 -0.052 -0.028 -0.12 0 0 -0.378 0.108 -0.012 0.179 -0.088 -0.173 0.07 -0.111 0.034 0.085 0.173 0 0 0 0.047 -0.111 -0.087 -0.116 0

8 2 0.291 -0.066 -0.028 -0.105 0 0 -0.34 0.108 -0.012 0.179 -0.088 -0.173 0.07 -0.034 0.034 -0.029 0.147 0 0 0 0.047 -0.111 -0.266 -0.116 0

8 2 0.291 -0.052 -0.028 -0.12 -0.182 0 -0.48 0.108 -0.012 0.179 -0.088 -0.173 0.07 -0.111 0.034 -0.029 0.124 0 0 0 0.047 -0.057 -0.087 -0.116 0

8 2 0.291 -0.066 -0.028 -0.105 0 0 -0.331 0.108 -0.012 0.179 -0.049 -0.173 0.07 -0.034 0.034 -0.029 0.124 0 0 0 0.047 -0.111 -0.266 -0.116 0

8 2 0.291 -0.052 -0.028 -0.105 0.12 0 -0.25 0.108 -0.012 0.179 -0.088 -0.173 0.07 -0.293 0.034 -0.029 0.124 0 0 0 0.047 -0.111 -0.128 -0.103 0

8 2 0.291 -0.127 -0.028 -0.12 0 0 -0.378 0.108 -0.012 0.179 -0.088 -0.173 0.07 -0.053 0.034 -0.029 0.124 0 0 0 0.047 -0.057 -0.087 -0.116 0

8 2 0.291 -0.066 -0.028 -0.105 0 0 -0.39 0.108 -0.012 0.179 -0.088 -0.173 0.07 -0.034 0.034 -0.029 0.124 0 0 0 0.047 -0.111 -0.128 -0.116 0.012

8 2 0.291 -0.052 -0.028 -0.105 0 0 -0.282 0.15 -0.012 0.179 -0.088 -0.173 0.07 -0.293 0.034 -0.029 0.124 0 0 0 0.047 -0.111 -0.128 -0.103 0

8 2 0.291 -0.052 -0.028 -0.12 0 0 -0.378 0.108 -0.012 0.179 -0.088 -0.173 0.07 -0.111 0.034 0.085 0.124 0 0 0 0.047 -0.111 -0.087 -0.116 0.014

8 2 0.291 -0.052 -0.028 -0.105 0 0 -0.25 0.15 -0.012 0.215 -0.088 -0.173 0.07 -0.293 0.034 -0.029 0.124 0 0 0 0.047 -0.111 -0.128 -0.103 0

8 2 0.115 -0.052 -0.028 -0.12 0 0 -0.25 0.108 -0.012 0.157 -0.088 -0.173 0.07 -0.052 0.034 -0.029 0.124 0 0 0 0.047 -0.111 -0.128 -0.116 0

8 2 0.291 -0.052 -0.028 -0.105 0 0 -0.25 0.134 -0.012 0.179 -0.038 -0.129 0.07 -0.111 0.034 -0.029 0.053 0 0 -0.035 0.047 -0.157 -0.128 -0.116 0

8 2 0.291 -0.066 -0.028 -0.105 0 0 -0.331 0.108 -0.012 0.179 -0.088 -0.173 0.07 -0.034 0.034 -0.029 0.142 0 0 0 0.047 -0.111 -0.266 -0.116 0

8 1 0.291 -0.052 -0.028 -0.12 0.047 0 -0.378 0.108 -0.012 0.179 -0.088 -0.173 0.07 -0.111 0.034 -0.029 0.124 0 0 0 0.047 -0.111 -0.087 -0.116 0

8 1 0.291 -0.052 0.1 -0.12 0 0 -0.25 0.108 -0.012 0.179 -0.088 -0.173 0.07 -0.111 0.034 -0.029 0.124 0 0 0 0.047 -0.111 -0.087 -0.116 0

8 1 0.291 -0.052 -0.028 -0.105 0 0 -0.331 0.108 -0.012 0.149 -0.088 -0.173 0.07 -0.111 0.034 -0.029 0.124 0 -0.125 -0.042 0.047 -0.093 -0.128 -0.116 0

8 1 0.291 -0.052 -0.028 -0.12 0 0 -0.25 0.108 -0.012 0.157 -0.088 -0.173 0.07 -0.052 0.145 -0.029 0.124 0 0 0 0.047 -0.111 -0.128 -0.116 0

8 1 0.291 -0.066 -0.028 -0.105 0 0.195 -0.331 0.108 -0.012 0.179 -0.088 -0.176 0.07 -0.111 0.034 -0.029 0.124 0.118 0 0 0.047 -0.111 -0.128 -0.116 0

8 1 0.291 -0.052 -0.028 -0.105 0 0 -0.331 0.108 -0.012 0.149 -0.088 -0.173 0.07 -0.111 0.034 -0.029 0.124 0 0.053 -0.042 0.047 -0.111 -0.128 -0.116 0

8 1 0.291 -0.052 -0.028 -0.12 0 0 -0.25 0.108 -0.012 0.179 -0.088 -0.173 0.07 -0.111 0.034 -0.029 0.124 0 0 0 0.047 -0.111 -0.097 -0.116 0

8 1 0.127 0.103 -0.028 -0.105 0 0 -0.331 0.108 -0.012 0.149 -0.088 -0.173 0.07 -0.111 0.034 -0.029 0.124 0 -0.125 -0.042 0.047 -0.111 -0.128 -0.116 0

8 1 0.291 -0.066 -0.028 -0.105 0 0 -0.331 0.108 -0.012 0.179 -0.088 -0.176 0.07 -0.111 0.034 -0.029 -0.005 0.118 0 0 0.047 -0.111 -0.128 -0.116 0

8 1 0.291 -0.052 -0.028 -0.12 0 0 -0.25 0.108 -0.012 0.157 -0.088 -0.173 0.07 -0.052 0.034 -0.029 0.124 0 0 0 0.047 -0.111 -0.128 -0.116 -0.242

8 1 0.291 -0.052 -0.028 -0.12 0 0 -0.25 0.108 -0.012 0.157 -0.088 -0.226 0.07 -0.052 0.034 -0.029 0.124 0 0 0 0.047 -0.111 -0.128 -0.116 0

8 1 0.291 -0.066 -0.028 -0.015 0 0 -0.331 0.108 -0.012 0.179 -0.155 -0.176 0.07 -0.111 0.034 -0.029 0.124 0.118 0 0 0.047 -0.111 -0.128 -0.116 0

8 1 0.394 -0.066 -0.017 -0.105 0 0 -0.331 0.108 -0.012 0.179 -0.088 -0.176 0.07 -0.111 0.034 -0.029 0.124 0.118 0 0 0.047 -0.111 -0.128 -0.116 0

8 1 0.291 -0.052 -0.028 -0.105 0 0 -0.25 0.134 -0.012 0.179 -0.088 -0.129 0.07 -0.111 0.034 -0.029 0.053 0 0 -0.035 -0.021 -0.157 -0.128 -0.116 0

8 1 0.291 -0.052 -0.028 -0.105 0.019 0 -0.331 0.108 -0.012 0.149 -0.088 -0.173 0.07 -0.02 0.034 -0.029 0.124 0 -0.125 -0.042 0.047 -0.111 -0.128 -0.116 0

8 1 0.291 -0.052 -0.028 -0.12 0 0.032 -0.25 0.108 -0.012 0.157 -0.088 -0.173 0.07 -0.052 0.034 -0.029 0.124 0 0 0 0.047 -0.111 -0.128 -0.116 0

8 1 0.291 -0.052 -0.028 -0.12 0 0 -0.378 0.108 -0.012 0.179 -0.088 -0.173 0.07 -0.111 0.034 0.085 0.124 0 0 0 0.047 -0.266 -0.087 -0.116 0

8 1 0.291 -0.066 -0.028 -0.105 0 0 -0.331 0.108 -0.012 0.179 -0.088 -0.176 0.07 -0.111 0.034 -0.029 0.124 0.22 0 0 0.047 -0.111 -0.175 -0.116 0

8 1 0.291 -0.052 -0.028 -0.12 0 0 -0.25 0.108 -0.012 0.157 0.035 -0.173 0.07 -0.052 0.034 -0.029 0.124 0 0 0 0.047 -0.111 -0.128 -0.116 -0.193

8 1 0.291 -0.052 -0.028 -0.12 0 0 -0.25 0.108 -0.012 0.157 0.035 -0.173 0.07 -0.052 0.034 -0.029 0.124 0 0 0.016 0.047 -0.111 -0.128 -0.116 0

8 1 0.291 -0.052 -0.038 -0.12 -0.039 0 -0.25 0.108 -0.012 0.157 -0.088 -0.173 0.1 -0.052 0.034 -0.029 0.124 0 0 0 0.047 -0.111 -0.128 -0.116 0

8 1 0.291 -0.052 -0.028 -0.12 0 0 -0.25 0.108 -0.012 0.157 0.035 -0.173 0.07 -0.052 0.034 -0.029 0.124 0 0 0.077 0.047 -0.111 -0.128 -0.116 0

8 1 0.291 -0.066 -0.028 -0.105 0 0 -0.331 0.201 -0.012 0.179 -0.088 -0.176 0.07 -0.111 0.034 -0.029 0.124 0.118 0 0 0.047 -0.111 -0.128 -0.116 0

8 1 0.291 -0.066 -0.028 -0.015 0 0 -0.331 0.108 -0.012 0.068 -0.088 -0.176 0.07 -0.111 0.034 -0.029 0.124 0.118 0 0 0.047 -0.111 -0.128 -0.116 0

8 1 0.291 -0.066 -0.028 0.09 0 0 -0.331 0.108 -0.012 0.179 -0.088 -0.176 0.07 -0.111 0.034 -0.029 0.124 0.118 0 0 0.047 -0.111 -0.128 -0.116 0

8 1 0.291 -0.052 -0.028 -0.105 0 0 -0.25 0.134 -0.012 0.179 -0.088 -0.129 0.07 -0.111 0.034 -0.029 0.053 0 0 -0.035 -0.021 -0.157 -0.126 -0.116 0

8 1 0.291 -0.052 -0.028 -0.12 0 0 -0.25 0.108 -0.012 0.148 0.035 -0.173 0.07 -0.052 0.034 -0.029 0.124 0 0 0 0.047 -0.111 -0.128 -0.116 0

8 1 0.291 -0.045 -0.028 -0.105 0 0 -0.258 0.108 -0.012 0.179 -0.088 -0.173 0.07 -0.293 0.034 -0.029 0.124 0 0 0 0.047 -0.111 -0.128 -0.103 0

8 1 0.291 -0.052 -0.028 -0.12 0 0 -0.25 0.108 -0.012 0.157 -0.088 -0.173 0.07 -0.052 0.034 -0.029 -0.143 0 0 0 0.047 -0.111 -0.128 -0.116 0

8 1 0.291 -0.052 -0.028 -0.12 0 0 -0.25 0.108 -0.012 0.157 -0.088 -0.173 0.07 -0.052 0.053 -0.179 0.124 0 -0.009 0 0.047 -0.111 -0.128 -0.116 0

8 1 0.291 -0.052 -0.028 -0.12 0 0 -0.378 0.108 0.088 0.179 -0.088 -0.173 0.07 -0.111 0.034 0.085 0.124 0 0 0 0.047 -0.111 -0.087 -0.116 0

8 1 0.291 -0.083 -0.028 -0.12 0 0 -0.25 0.108 -0.012 0.157 -0.088 -0.173 0.07 -0.052 0.034 -0.029 0.124 0 0 0 0.047 -0.111 -0.128 -0.116 0

8 1 0.291 -0.052 -0.028 -0.12 0 0 -0.25 0.108 -0.012 0.179 -0.088 -0.173 0.07 -0.111 0.034 -0.029 0.124 0 0 0 0.047 -0.111 -0.087 -0.116 0.188

8 1 0.291 -0.066 0.104 -0.105 0 0 -0.331 0.108 -0.012 0.179 -0.088 -0.173 0.07 -0.034 0.034 -0.029 0.124 0 0 0 0.047 -0.111 -0.128 -0.116 0.012

8 1 0.291 -0.052 -0.028 -0.105 0 -0.168 -0.25 0.107 -0.012 0.179 -0.088 -0.173 0.07 -0.293 0.034 -0.029 0.124 0 0 0 0.047 -0.111 -0.128 -0.103 0

8 1 0.291 -0.066 -0.028 -0.015 0 -0.026 -0.331 0.108 -0.012 0.179 -0.088 -0.176 0.07 -0.111 0.034 -0.029 0.124 0.118 0 0 0.047 -0.111 -0.128 -0.116 0

8 1 0.291 -0.052 -0.028 -0.12 0 0 -0.25 -0.016 -0.012 0.179 -0.088 -0.173 0.07 -0.111 0.034 0.196 0.124 0 0 0 0.061 -0.111 -0.087 -0.116 0

8 1 0.291 -0.066 -0.028 -0.105 0 0 -0.331 0.108 -0.012 0.179 -0.088 -0.173 0.07 -0.034 0.034 -0.029 0.124 0 0 0 0.121 -0.111 -0.128 -0.116 0.012

8 1 0.291 -0.052 -0.028 -0.12 0 0 -0.25 0.108 -0.012 0.157 -0.088 -0.173 0.07 -0.052 0.034 -0.029 0.124 0 -0.076 0 0.047 -0.111 -0.128 -0.116 0

8 1 0.291 -0.052 -0.028 -0.12 0 0 -0.25 0.108 -0.012 0.179 -0.088 -0.173 0.07 -0.111 -0.058 -0.029 0.124 0 0 0 0.047 -0.111 -0.087 -0.116 0

8 1 0.291 -0.052 -0.028 -0.12 0 0 -0.25 0.108 -0.012 0.157 0.035 -0.173 0.07 -0.052 0.034 -0.029 0.124 0 0 0 0.047 -0.111 -0.128 -0.206 0

8 1 0.291 -0.052 -0.028 -0.12 0 0 -0.25 0.108 -0.012 0.157 -0.088 -0.173 0.07 -0.052 0.034 0.091 0.124 0 0 0 0.047 -0.111 -0.128 -0.116 0

8 1 0.291 -0.052 -0.028 -0.105 0 0 -0.25 0.312 -0.198 0.179 -0.088 -0.173 0.07 -0.293 0.034 -0.029 0.124 0 0 0 0.047 -0.111 -0.128 -0.103 0

8 1 0.291 -0.052 -0.028 -0.12 0 0 -0.25 0.108 -0.012 0.179 -0.088 -0.173 0.07 -0.111 0.034 -0.029 0.124 0 0 0 0.005 -0.111 -0.087 -0.116 0

8 1 0.291 -0.052 -0.028 -0.12 0 0 -0.25 0.108 -0.012 0.157 -0.088 -0.173 0.07 -0.052 0.034 -0.029 0.124 -0.076 0 0.133 0.047 -0.111 -0.128 -0.116 0

8 1 0.291 -0.052 -0.028 -0.12 0.199 0 -0.25 0.108 -0.012 0.157 -0.088 -0.173 0.07 -0.052 0.034 -0.029 0.124 0 0 0 0.047 -0.111 -0.128 -0.116 0

8 1 0.291 -0.052 -0.028 -0.105 0 0 -0.25 0.134 -0.012 0.179 -0.088 -0.129 0.07 -0.111 0.034 -0.029 0.053 0 0.011 -0.035 0.047 -0.157 -0.128 -0.116 0

8 1 0.291 -0.052 -0.052 -0.12 0 0 -0.25 0.108 -0.012 0.157 -0.088 -0.173 0.07 -0.052 0.053 -0.029 0.124 0 -0.009 0 0.047 -0.111 -0.128 -0.116 0

8 1 0.291 -0.066 -0.028 -0.105 0 0 -0.331 0.108 -0.012 0.179 -0.088 -0.176 -0.115 -0.111 0.034 -0.029 0.124 0.118 0 0 0.047 -0.111 -0.175 -0.116 0

8 1 0.291 -0.052 -0.028 -0.12 0 0 -0.25 0.108 -0.012 0.157 -0.088 -0.302 0.07 -0.052 0.053 -0.029 0.124 0 -0.009 0 0.047 -0.111 -0.128 -0.116 0

8 1 0.291 -0.052 -0.028 -0.105 0 0 -0.331 0.108 -0.012 0.149 -0.088 -0.173 0.07 -0.111 0.034 -0.029 0.124 0.129 -0.125 -0.042 0.047 -0.111 -0.128 -0.116 0

8 1 0.291 -0.052 -0.028 -0.12 0 0 -0.25 0.108 -0.012 0.157 -0.088 -0.173 0.07 -0.052 0.034 -0.029 0.124 0 0 0 0.047 -0.111 -0.128 -0.128 0

8 1 0.291 -0.033 -0.028 -0.12 0 0 -0.25 0.108 -0.012 0.157 -0.088 -0.173 0.07 -0.052 0.034 -0.029 0.124 0 0 0 0.047 -0.111 -0.128 -0.116 0

8 1 0.291 -0.066 -0.028 -0.015 -0.174 0 -0.331 0.108 -0.012 0.179 -0.088 -0.176 0.07 -0.111 0.034 -0.029 0.124 0.118 0 0 0.047 -0.111 -0.128 -0.116 0

8 1 0.291 -0.052 -0.028 -0.12 0 0 -0.25 0.108 -0.012 0.179 -0.088 -0.173 0.07 -0.111 0.034 -0.029 0.124 0 0 0 0.047 -0.111 -0.087 0.015 0

8 1 0.291 -0.052 -0.028 -0.12 0 0 -0.25 0.108 -0.012 0.179 -0.088 -0.173 0.07 -0.111 0.034 -0.029 0.124 0.267 0 0 0.047 -0.111 -0.087 -0.116 0

8 1 0.291 -0.052 -0.028 -0.105 0 0 -0.331 0.108 -0.012 0.149 -0.088 -0.173 0.07 -0.111 0.034 -0.029 0.124 0 -0.125 -0.042 0.047 -0.111 -0.128 -0.233 0

8 1 0.291 -0.052 -0.028 -0.12 0 0 -0.25 0.108 -0.012 0.157 -0.088 -0.173 -0.008 -0.052 0.034 -0.029 0.124 0 0 0 0.047 -0.111 -0.128 -0.116 0

8 1 0.291 -0.066 -0.028 -0.105 0 0 -0.331 0.108 -0.012 0.179 -0.088 -0.173 0.156 -0.034 0.034 -0.029 0.124 0 0 0 0.047 -0.111 -0.266 -0.116 0

8 1 0.291 -0.052 -0.028 -0.105 0 0 -0.25 0.134 -0.012 0.179 -0.088 -0.129 0.07 -0.111 0.034 -0.029 0.084 0 0 -0.035 0.047 -0.157 -0.128 -0.116 0

8 1 0.291 -0.052 -0.028 -0.12 0 0 -0.25 0.108 -0.012 0.157 -0.088 -0.173 0.07 -0.052 0.034 -0.029 0.124 0 0 0 0.047 -0.111 -0.034 -0.116 0

8 1 0.291 -0.052 0.06 -0.12 0 0 -0.25 0.108 -0.012 0.157 -0.088 -0.173 0.07 -0.052 0.034 -0.029 0.124 0.08 0 0 0.047 -0.111 -0.128 -0.116 0

8 1 0.291 -0.066 -0.028 -0.105 0 0.086 -0.331 0.108 -0.012 0.179 -0.088 -0.173 0.07 -0.034 0.034 -0.029 0.124 0 0 0 0.047 -0.111 -0.266 -0.116 0

8 1 0.291 -0.052 -0.028 -0.12 0 0 -0.378 0.108 -0.01 0.179 -0.088 -0.173 0.07 -0.111 0.034 -0.029 0.124 0 0 0 0.047 -0.057 -0.087 -0.116 0

8 1 0.291 -0.052 -0.028 -0.105 0 0 -0.25 0.134 -0.156 0.179 -0.088 -0.129 0.07 -0.111 0.034 -0.029 0.053 0 0 -0.035 0.023 -0.157 -0.128 -0.116 -0.024

8 1 0.291 -0.052 -0.028 -0.12 0 0 -0.25 0.108 -0.012 0.157 -0.088 -0.173 0.07 -0.052 0.034 -0.029 0.124 0 0 0 0.047 -0.111 -0.128 -0.27 0

8 1 0.291 -0.052 -0.028 -0.12 0 0 -0.237 0.108 -0.012 0.157 -0.088 -0.173 0.07 -0.052 0.034 -0.029 0.124 0 0 0 0.047 -0.111 -0.128 -0.116 0

8 1 0.291 -0.052 -0.028 -0.12 0 0 -0.25 0.108 -0.012 0.157 0.035 -0.173 0.07 -0.052 0.034 -0.029 0.124 0 0 0 0.047 -0.064 -0.128 -0.116 0

8 1 0.291 -0.052 -0.028 -0.12 0 0 -0.25 0.108 -0.088 0.179 -0.088 -0.173 0.07 -0.111 0.034 -0.029 0.124 0 0 0 0.047 -0.111 -0.087 -0.116 0

8 1 0.291 -0.052 -0.028 -0.12 0 0 -0.25 0.108 -0.012 0.179 -0.088 -0.173 0.07 -0.111 -0.048 -0.029 0.124 0 0 0 0.047 -0.111 -0.087 -0.116 0

8 1 0.291 -0.052 -0.028 -0.12 0 0 -0.25 0.108 -0.012 0.179 -0.088 -0.173 0.144 -0.111 0.034 -0.029 0.124 0 0 0 0.047 -0.111 -0.087 -0.116 0

8 1 0.291 -0.052 0.057 -0.12 0 0 -0.25 0.108 -0.012 0.157 -0.088 -0.173 0.07 -0.052 0.034 -0.029 0.124 0 0 0 0.047 -0.111 -0.128 -0.116 0

8 1 0.291 -0.052 -0.028 -0.105 0 0 -0.331 0.108 -0.012 0.149 -0.088 -0.173 -0.071 -0.111 0.034 -0.029 0.124 0 -0.125 -0.042 0.047 -0.111 -0.128 -0.116 0

8 1 0.291 -0.052 -0.028 -0.12 0.109 0 -0.25 0.039 -0.012 0.157 -0.088 -0.173 0.07 -0.052 0.034 -0.029 0.124 0 0 0 0.047 -0.111 -0.128 -0.116 0

8 1 0.291 -0.066 -0.028 -0.105 0 0 -0.331 0.108 -0.012 0.179 -0.088 -0.173 0.07 -0.034 0.034 -0.029 0.17 0 0 0 0.047 -0.111 -0.266 -0.116 0

8 1 0.291 -0.052 -0.028 -0.105 0 0 -0.25 0.134 -0.012 0.179 -0.088 -0.129 0.07 -0.111 0.034 -0.029 0.053 0 0 -0.035 0.047 -0.154 -0.128 -0.116 0

8 1 0.291 -0.052 -0.005 -0.12 0 0 -0.25 0.108 -0.012 0.157 -0.088 -0.173 0.07 -0.052 0.034 -0.029 0.124 0 0 0 0.047 -0.111 -0.128 -0.116 0

8 1 0.291 -0.052 -0.028 -0.12 0 0 -0.25 -0.016 -0.012 0.179 -0.088 -0.173 0.07 -0.111 0.034 0.196 0.124 0 -0.052 0 0.061 -0.111 -0.087 -0.116 0

8 1 0.291 -0.052 -0.028 -0.105 0 0 -0.25 0.134 -0.012 0.119 -0.088 -0.129 0.07 -0.111 0.034 -0.029 0.053 0 0 -0.035 0.047 -0.137 -0.102 -0.116 0

9 481 0.031 -0.028 0 0.061 0.017 -0.149 -0.05 -0.016 0.013 0.106 0.123 -0.006 -0.012 0.064 0.108 -0.047 0.073 0.02 0 0.151 -0.045 0.088 0.021 -0.074 0

9 351 0.031 -0.028 0 0.061 0.017 -0.149 -0.05 -0.016 0.013 0.106 0.123 -0.142 -0.012 0.064 0.108 -0.047 0.073 0.02 0 0.151 -0.045 0.088 0.021 -0.074 0

9 544 0.031 -0.028 0 0.061 0.017 -0.152 -0.05 -0.016 0 0.106 0.123 0 -0.012 0.064 0.108 -0.069 0.073 0.02 0 0.036 -0.045 0.039 0.021 -0.074 0

9 203 0.031 -0.028 -0.032 0.061 0.017 -0.152 -0.05 -0.016 0 0.106 0.123 0 -0.012 0.064 0.108 -0.069 0.073 -0.029 0 0.036 -0.045 0.039 0.021 -0.074 0

9 180 0.031 -0.028 0 0.061 0.017 -0.152 -0.05 -0.016 0 0.106 0.123 0 -0.012 0.064 0.108 -0.069 0.073 0.02 0 0.036 -0.154 0.039 0.021 -0.074 0

9 338 0.031 -0.028 0 0.061 0.017 -0.149 -0.05 -0.016 0.013 0.106 0.123 -0.006 -0.012 0.064 0.108 -0.047 0.073 0.02 0 0.151 -0.045 0.138 0.021 -0.074 0

9 144 0.031 -0.028 0 0.061 0.017 -0.149 -0.05 -0.016 0.013 0.076 0.123 -0.006 -0.012 0.064 0.108 -0.047 0.073 0.02 0 0.151 -0.045 0.138 0.021 -0.074 0

9 134 0.031 -0.028 0 0.061 0.017 -0.152 -0.05 -0.016 -0.014 0.106 0.123 0 -0.012 0.064 0.108 -0.069 0.073 0.02 0 0.036 -0.045 0.039 0.021 -0.074 0

9 134 0.031 -0.028 0 0.061 0.017 -0.152 -0.05 -0.016 0 -0.057 0.123 0 -0.012 0.064 0.108 -0.069 0.073 0.02 0 0.036 -0.045 0.039 0.021 0.128 0

9 156 0.031 -0.028 0 0.076 0.017 -0.149 -0.05 -0.016 0.013 0.106 0.123 -0.006 -0.012 0.064 0.108 -0.047 0.073 0.02 0 0.151 -0.045 0.088 0.021 -0.074 0

9 103 0.031 -0.028 0 0.061 0.017 -0.152 -0.05 -0.016 0 0.106 0.123 0 -0.012 0.064 0.108 -0.069 0.073 -0.167 0 0.036 -0.154 0.039 0.021 -0.074 0

9 67 0.031 -0.028 0 0.061 0.017 -0.152 -0.05 -0.016 0 0.106 0.123 0 -0.012 0.064 0.108 -0.069 0.073 -0.029 0 0.036 -0.045 0.039 0.021 -0.074 0

9 66 0.031 -0.028 0 0.076 0.017 -0.149 -0.05 -0.016 0.013 0.106 0.123 -0.006 -0.012 0.064 0.108 -0.047 0.073 0.02 0 0.151 -0.045 0.11 0.021 -0.074 0

9 60 0.031 -0.028 0 0.061 0.017 -0.149 -0.05 -0.016 0.013 0.106 0.123 -0.006 -0.012 0.064 0.108 -0.101 0.021 0.02 0 0.151 -0.045 0.088 0.021 -0.074 0

9 53 0.031 -0.028 0 0.061 0.017 -0.149 -0.05 -0.016 0.013 0.076 0.123 -0.006 0.029 0.064 0.108 -0.047 0.073 0.02 0 0.151 -0.045 0.138 0.021 -0.074 0

9 40 0.031 -0.028 0 0.061 0.017 -0.149 -0.05 -0.016 0.013 0.106 0.123 -0.006 -0.012 0.064 0.108 -0.047 0.073 0.02 0 0.392 -0.045 0.088 0.021 -0.074 0

9 28 0.031 -0.028 0 0.061 0.017 -0.152 -0.05 -0.016 0 -0.057 0.123 0 -0.012 0.064 0.108 -0.069 0.122 0.02 0 0.036 -0.045 0.039 0.021 -0.074 0

9 27 0.031 -0.028 0 0.061 0.017 -0.152 -0.05 -0.016 -0.014 0.106 0.123 0 -0.012 0.064 0.108 -0.069 0.073 -0.12 0 0.036 -0.045 0.039 0.021 -0.074 0

9 25 0.031 -0.028 0 0.061 0.017 -0.152 -0.05 -0.016 0 -0.057 0.123 0 -0.012 0.064 0.108 -0.069 0.073 0.02 0 0.036 -0.045 0.039 0.021 -0.074 0

9 23 0.031 -0.028 0 0.061 0.017 -0.152 -0.05 -0.016 0 -0.057 0.123 0 -0.012 0.064 0.108 -0.069 0.051 0.02 0 0.036 -0.045 0.039 0.021 0.128 0

9 19 0.031 -0.028 0 0.061 0.017 -0.152 -0.05 -0.016 -0.014 0.106 0.123 0 -0.012 0.064 0.108 -0.069 0.073 0.02 0 0.036 -0.045 0.039 0.077 -0.074 0

9 18 0.031 -0.028 0 0.061 0.017 -0.152 0.055 -0.016 0 0.106 0.123 0 -0.012 0.064 0.108 -0.069 0.073 -0.029 0 0.036 -0.045 0.039 0.021 -0.074 0

9 18 0.031 -0.028 -0.032 0.061 0.017 -0.152 -0.05 -0.016 0 0.106 0.123 0 0.086 0.064 0.108 -0.069 0.073 -0.029 0 0.036 -0.045 0.039 0.021 -0.074 0

9 10 0.031 -0.028 0 0.061 0.017 -0.149 -0.05 -0.016 0.013 0.106 0.123 -0.006 -0.012 0.064 0.108 -0.047 0.073 0.02 0 0.151 -0.045 0.088 0.021 -0.056 0

9 9 0.031 -0.028 0 0.061 0.017 -0.149 -0.05 -0.016 0.013 0.106 0.123 -0.006 -0.012 0.064 0.108 -0.047 0.092 0.02 0 0.151 -0.045 0.088 0.021 -0.074 0

9 8 0.031 -0.028 0 0.061 0.017 -0.149 -0.05 -0.045 0.013 0.106 0.123 -0.006 -0.012 0.064 0.108 -0.047 0.073 0.02 0 0.151 -0.045 0.088 0.021 -0.074 0

9 7 0.031 -0.028 0 0.061 0.017 -0.152 -0.05 -0.016 -0.046 0.106 0.123 0 -0.012 0.064 0.108 -0.069 0.073 0.02 0 0.036 -0.045 0.039 0.021 -0.074 0

9 5 0.031 -0.028 0 0.061 0.017 -0.149 -0.05 -0.016 0.013 0.106 0.123 -0.006 -0.079 0.064 0.108 -0.047 0.073 0.02 0 0.151 -0.045 0.088 0.021 -0.074 0

9 4 0.031 -0.028 0 0.061 0.017 -0.152 -0.05 -0.016 0 0.106 0.123 0.034 -0.012 0.064 0.108 -0.069 0.073 0.02 0 0.036 -0.045 0.039 0.021 -0.074 0

9 4 0.031 -0.028 -0.069 0.061 0.017 -0.152 -0.05 -0.016 0 0.106 0.123 0 -0.012 0.064 0.108 -0.069 0.073 0.02 0 0.036 -0.045 0.039 0.021 -0.074 0

9 4 0.031 -0.028 0 0.061 0.017 -0.152 -0.05 -0.03 0 0.106 0.123 0 -0.012 0.064 0.108 -0.069 0.073 0.02 0 0.036 -0.045 0.039 0.021 -0.074 0

9 4 0.031 -0.028 0 0.061 0.017 -0.152 -0.05 -0.016 0 0.106 0.123 0 -0.012 0.064 0.108 -0.069 0.253 0.02 0 0.036 -0.045 0.039 0.021 -0.074 0

9 4 0.031 -0.028 0 0.061 0.017 -0.152 -0.05 -0.016 0 0.106 0.123 0 -0.012 0.064 0.108 0.14 0.073 0.02 0 0.036 -0.045 0.039 0.021 -0.074 0

9 4 0.031 -0.028 0 0.061 0.017 -0.149 -0.05 -0.016 0.013 0.106 0.123 0.071 -0.012 0.064 0.108 -0.047 0.073 0.02 0 0.151 -0.045 0.088 0.021 -0.074 0

9 4 0.031 -0.028 0 -0.095 0.017 -0.149 -0.05 -0.016 0.013 0.106 0.123 -0.006 -0.012 0.064 0.108 -0.047 0.073 0.02 0 0.392 -0.045 0.088 0.021 -0.074 0

9 3 -0.036 -0.028 0 0.061 0.017 -0.152 -0.05 -0.016 0 -0.057 0.123 0 -0.012 0.064 0.108 -0.069 0.073 0.02 0 0.036 -0.045 0.039 0.021 -0.074 0

9 4 0.031 -0.028 0 0.061 0.017 -0.149 0 -0.016 0.013 0.106 0.123 -0.142 -0.012 0.064 0.108 -0.047 0.073 0.02 0 0.151 -0.045 0.088 0.021 -0.074 0

9 3 0.014 -0.028 0 0.061 0.017 -0.152 -0.05 -0.016 0 0.106 0.123 0 -0.012 0.064 0.108 -0.069 0.073 0.02 0 0.036 -0.045 0.039 0.021 -0.074 0

9 3 0.031 -0.028 0 0.061 0.017 -0.149 -0.05 -0.016 0.013 0.106 0.123 -0.006 -0.012 0.064 0.108 -0.047 0.073 0.02 0 0.151 -0.045 0.228 0.021 -0.074 0

9 3 0.031 -0.028 0 0.061 0.017 -0.149 -0.05 -0.016 0.013 0.106 0.123 -0.006 -0.012 0.064 0.108 -0.047 0.121 0.02 0 0.151 -0.045 0.088 0.021 -0.074 0

9 3 0.031 -0.028 0 0.076 0.017 -0.149 -0.05 -0.016 0.013 0.106 0.123 -0.006 -0.012 0.064 0.293 -0.047 0.073 0.02 0 0.151 -0.045 0.088 0.021 -0.074 0

9 3 0.031 -0.028 0 0.061 0.017 -0.152 -0.05 -0.016 0 0.106 0.123 0 -0.012 0.064 0.108 -0.069 0.073 0.02 0 0.114 -0.154 0.039 0.021 -0.074 0

9 3 0.031 -0.028 0 0.061 0.017 -0.149 -0.05 -0.016 0.013 0.106 0.123 -0.142 -0.012 0.064 0.108 -0.047 0.073 -0.022 0 0.151 -0.045 0.088 0.021 -0.074 0

9 2 0.031 -0.028 0 0.061 0.017 -0.149 -0.05 -0.016 0.013 0.106 0.123 -0.006 -0.012 0.064 0.108 -0.201 0.073 0.02 0 0.151 -0.045 0.138 0.021 -0.074 0

9 2 0.031 -0.028 0 0.061 0.017 -0.152 -0.05 -0.016 0 0.106 0.123 0 -0.012 0.064 0.108 -0.069 0.073 0.02 0 0.036 -0.045 0.039 0.021 0.09 0

9 2 -0.036 -0.028 0 0.061 0.017 -0.152 -0.05 -0.016 0 -0.057 0.123 0 0.071 0.064 0.108 -0.069 0.073 0.02 0 0.036 -0.045 0.039 0.021 -0.074 0

9 2 0.031 -0.028 0 0.061 0.017 -0.149 -0.05 -0.016 0.074 0.106 0.123 -0.006 -0.012 0.064 0.108 -0.047 0.092 0.02 0 0.151 -0.045 0.088 0.021 -0.074 0

9 2 0.031 -0.028 0 0.061 0.017 -0.289 -0.05 -0.016 0 -0.057 0.123 0 -0.012 0.064 0.108 -0.069 0.073 0.02 0 0.036 -0.045 0.039 0.021 -0.074 0

9 2 0.031 -0.028 -0.041 0.061 0.017 -0.152 -0.05 -0.016 0 0.106 0.123 0 -0.012 0.064 0.108 -0.069 0.073 0.02 0 0.036 -0.045 0.039 0.021 -0.074 0

9 2 0.031 -0.028 0 0.089 0.017 -0.152 -0.05 -0.016 0 0.106 0.123 0 -0.012 0.064 0.108 -0.069 0.073 0.02 0 0.036 -0.045 0.039 0.021 -0.074 0

9 2 0.031 -0.01 0 0.061 0.017 -0.152 -0.05 -0.016 0 0.106 0.123 0 -0.012 0.064 0.108 -0.069 0.073 0.02 0 0.036 -0.154 0.039 0.021 -0.074 0

9 2 0.031 -0.028 0 0.061 0.017 -0.149 -0.05 -0.016 0.013 0.076 0.123 -0.006 0.029 0.064 0.108 -0.047 0.073 0.02 0 0.151 -0.045 0.138 0.021 -0.104 0

9 2 0.031 -0.028 0 0.061 0.017 -0.152 -0.05 -0.016 0 0.106 0.123 0 -0.028 0.064 0.108 -0.069 0.073 0.02 0 0.036 -0.045 0.039 0.021 -0.074 0

9 2 0.031 -0.028 0 0.061 0.017 -0.149 -0.05 -0.016 0.013 0.076 -0.016 -0.006 0.029 0.064 0.108 -0.047 0.073 0.02 0 0.151 -0.045 0.138 0.021 -0.074 0

9 2 0.031 -0.028 0 0.061 0.017 -0.149 -0.05 -0.016 0.013 0.106 0.089 -0.006 -0.012 0.064 0.108 -0.047 0.073 0.02 0 0.151 -0.045 0.138 0.021 -0.074 0

9 2 0.031 -0.028 0 0.061 0.017 -0.152 -0.05 -0.016 0 0.106 0.123 0 -0.012 0.064 0.108 -0.069 0.073 -0.074 0 0.036 -0.045 0.039 0.021 -0.074 0

9 2 0.031 -0.028 0 0.061 0.017 -0.152 -0.05 -0.016 0 0.106 0.123 0 0.055 0.064 0.108 -0.069 0.073 0.02 0 0.036 -0.045 0.039 0.021 -0.074 0

9 2 0.031 -0.028 0 0.061 0.017 -0.086 -0.05 -0.016 0 -0.038 0.123 0 -0.012 0.064 0.108 -0.069 0.073 0.02 0 0.036 -0.154 0.039 0.021 -0.074 0

9 2 0.031 -0.028 0 0.061 0.017 -0.152 -0.05 -0.016 0 0.106 0.123 0 -0.012 0.064 0.108 0.066 0.073 0.02 0 0.036 -0.045 0.039 0.021 -0.074 0

9 2 0.031 -0.028 0 0.061 0.017 -0.052 -0.05 -0.016 0.013 0.106 0.123 -0.006 -0.012 0.064 0.108 -0.047 0.073 0.02 0 0.392 -0.045 0.088 0.008 -0.074 0

9 2 0.031 -0.028 0 0.061 0.017 -0.149 -0.05 -0.016 0.013 0.106 0.123 -0.006 -0.012 0.064 0.108 -0.047 0.089 0.02 0 0.151 -0.045 0.138 0.021 -0.074 0

9 2 0.031 -0.028 0 0.061 0.017 -0.149 -0.05 -0.016 0.013 0.106 0.123 -0.243 -0.012 0.064 0.108 -0.047 0.073 0.02 0 0.151 -0.045 0.088 0.021 -0.074 0

9 2 0.031 -0.028 0 0.076 0.017 -0.149 -0.05 -0.016 0.013 0.106 0.123 -0.006 -0.012 0.064 0.108 -0.179 0.073 0.02 0 0.151 -0.045 0.11 0.021 -0.074 0

9 2 0.031 -0.028 0 0.061 0.017 -0.149 -0.05 -0.016 0.013 0.106 0.123 -0.142 -0.012 0.064 0.108 -0.047 0.073 0.02 0 0.151 -0.045 0.088 0.021 -0.02 0

9 2 0.031 -0.028 0 0.061 0.017 -0.152 -0.05 -0.016 0 0.106 0.123 0 -0.012 0.064 0.108 -0.069 0.073 0.02 0 0.036 -0.045 0.039 0.061 -0.074 0

9 2 0.031 -0.028 -0.032 0.061 0.017 -0.152 -0.05 -0.016 0 0.106 0.123 0 -0.012 0.064 0.108 -0.069 0.106 -0.029 0 0.036 -0.045 0.039 0.021 -0.074 0

9 1 0.031 -0.028 0 0.061 0.017 -0.152 -0.05 -0.016 0 0.106 0.123 0.037 -0.012 0.064 0.108 -0.069 0.073 0.02 0 0.036 -0.154 0.039 -0.014 -0.074 0

9 2 0.031 -0.028 0 0.061 0.017 -0.149 -0.05 -0.016 0.013 0.106 0.123 -0.006 -0.15 0.064 0.108 -0.201 0.073 0.02 0 0.151 -0.045 0.138 0.021 -0.074 0

9 1 0.031 -0.028 0 0.061 0.017 -0.152 -0.05 -0.016 0 0.106 0.123 0.037 -0.012 0.024 0.108 -0.069 0.073 0.02 0 0.036 -0.154 0.039 -0.014 -0.074 0

9 1 0.031 -0.028 0.095 0.061 0.017 -0.149 -0.05 -0.016 0.013 0.106 0.123 -0.006 -0.012 0.064 0.108 -0.047 0.073 0.02 0 0.151 -0.045 0.088 0.021 -0.074 0

9 1 0.031 -0.028 0 0.061 0.017 -0.149 -0.05 -0.016 0.013 0.106 0.123 -0.006 -0.012 0.064 0.108 -0.047 0.073 0.02 0 0.105 -0.045 0.138 0.021 -0.074 0

9 1 0.031 -0.028 0 0.061 0.017 -0.149 -0.05 -0.016 0.013 0.106 0.123 -0.006 -0.012 0.064 0.108 -0.047 0.073 0.02 -0.012 0.151 -0.045 0.088 0.021 -0.074 0

9 1 0.031 -0.028 0 0.061 0.017 -0.149 -0.05 0.164 0.013 0.106 0.123 -0.006 -0.012 0.064 0.108 -0.047 0.073 0.02 0 0.151 -0.045 0.138 0.021 -0.074 0

9 1 0.031 -0.028 0 0.061 0.017 -0.149 -0.05 -0.016 0.013 0.106 0.123 -0.142 -0.012 0.064 0.108 -0.047 0.073 0.02 0.017 0.151 -0.045 0.088 0.021 -0.074 0

9 1 0.031 -0.028 0 0.061 0.017 -0.149 -0.05 -0.016 0.013 0.106 0.123 -0.006 -0.012 0.064 0.108 -0.047 0.073 0.02 0 0.151 -0.045 0.138 0.021 -0.074 -0.004

9 1 0.031 -0.028 0 0.061 0.017 -0.152 -0.05 -0.016 0 0.106 0.123 0 -0.012 0.064 0.108 -0.069 0.073 0.02 0 0.036 -0.045 0.039 0.021 -0.074 -0.213

9 1 0.031 -0.028 0 0.061 0.017 -0.149 -0.05 -0.016 0.013 0.106 0.123 -0.006 -0.012 0.064 0.108 -0.047 0.073 0.02 0 0.175 -0.045 0.088 0.021 -0.074 0

9 1 0.031 -0.028 0 0.061 0.017 -0.149 -0.05 -0.016 0.013 0.106 0.123 -0.142 -0.012 0.064 0.108 -0.047 0.073 0.02 0 0.151 -0.045 0.088 0.021 -0.012 0

9 1 0.031 -0.028 0 0.061 0.017 -0.149 -0.05 -0.016 0.013 0.106 0.123 -0.006 -0.012 0.064 0.108 0.016 0.073 0.02 0 0.151 -0.045 0.138 0.021 -0.074 0

9 1 0.031 -0.028 0 0.061 0.017 -0.152 -0.05 -0.016 -0.014 0.106 0.123 0 -0.012 0.064 0.108 -0.069 0.073 0.02 0 0.036 -0.045 0.082 0.021 -0.074 0

9 1 0.031 -0.028 0 0.061 0.017 -0.152 -0.05 -0.016 0 -0.038 0.123 0 -0.012 0.064 0.108 -0.069 0.073 0.02 0 0.036 -0.154 0.039 0.021 -0.074 0

9 1 0.031 -0.028 0 0.061 0.017 -0.149 -0.05 -0.016 0.013 0.106 0.123 -0.006 -0.012 0.064 0.108 -0.047 0.073 0.02 0 0.151 -0.045 0.138 0.021 -0.074 0.011

9 1 0.031 -0.028 0.092 0.061 0.017 -0.152 -0.05 -0.016 -0.014 0.106 0.123 0 -0.012 0.064 0.108 -0.069 0.073 0.02 0 0.036 -0.045 0.039 0.021 -0.074 0

9 1 0.031 -0.028 0 0.061 0.017 -0.149 -0.05 -0.016 0.013 -0.011 0.123 -0.006 -0.012 0.064 0.108 -0.047 0.073 0.02 0 0.151 -0.045 0.138 0.021 -0.074 0

9 1 0.031 -0.028 0 0.061 0.017 -0.149 -0.05 -0.016 0.013 0.106 0.123 -0.006 -0.012 0.064 0.108 -0.047 0.092 0.02 -0.038 0.151 -0.045 0.088 0.021 -0.074 0

9 1 0.031 -0.028 0 0.061 0.017 -0.152 -0.05 -0.016 0 0.106 0.123 0 -0.012 0.064 0.108 -0.069 0.073 0.02 0 0.036 -0.045 0.039 0.021 -0.074 -0.039

9 1 0.031 -0.028 0 0.153 0.017 -0.152 -0.05 -0.016 0 0.106 0.123 0 -0.012 0.064 0.108 -0.069 0.073 0.02 0 0.036 -0.045 0.039 0.021 -0.074 0

9 1 0.031 -0.028 0 0.061 0.017 -0.152 -0.05 -0.016 0.125 0.106 0.123 0 -0.012 0.064 0.108 -0.069 0.073 0.02 0 0.036 -0.154 0.039 0.021 -0.074 0

9 1 0.031 -0.028 0 0.061 0.017 -0.149 -0.05 -0.016 0.013 0.106 0.123 -0.006 -0.012 0.064 0.108 -0.047 0.073 0.02 0 0.392 -0.045 0.088 0.021 -0.074 -0.06

9 1 0.031 -0.028 0 0.061 0.017 -0.152 -0.05 -0.016 0 0.106 0.123 0 -0.012 0.064 0.108 -0.069 0.073 0.02 0 0.036 -0.045 0.039 -0.159 -0.074 0

9 1 0.031 -0.028 0 0.061 0.017 -0.152 -0.05 -0.016 0 -0.021 0.123 0 -0.012 0.064 0.108 -0.069 0.073 0.02 0 0.036 -0.045 0.039 0.021 -0.074 0

9 1 0.031 -0.028 0 0.061 0.017 -0.152 -0.05 -0.016 0 0.106 0.267 0 -0.012 0.064 0.108 -0.069 0.073 0.02 0 0.036 -0.045 0.039 0.021 -0.074 0

9 1 0.031 -0.028 0.152 0.061 0.017 -0.152 -0.05 -0.016 0 0.106 0.123 0 -0.012 0.064 0.108 -0.069 0.073 0.02 0 0.036 -0.045 0.039 0.021 -0.074 0

9 1 0.031 -0.028 0 0.061 0.017 -0.152 -0.05 -0.016 -0.014 0.106 0.123 0 -0.012 0.064 0.108 -0.069 0.073 0.02 0 0.036 -0.131 0.039 0.021 -0.074 0

9 1 0.031 -0.028 0 0.061 0.017 -0.149 -0.05 -0.016 0.013 0.106 0.123 -0.142 -0.012 0.064 0.108 -0.047 0.073 0.02 0 0.151 -0.045 0.137 0.021 -0.074 0

9 1 0.031 -0.028 0 0.061 0.017 -0.149 -0.05 -0.195 0.013 0.106 0.123 -0.006 -0.012 0.064 0.108 -0.047 0.073 0.02 0 0.151 -0.045 0.138 0.021 -0.074 0

9 1 0.031 -0.028 0 0.061 0.017 -0.152 -0.05 -0.016 0 0.106 0.123 0 -0.012 0.064 0.108 -0.069 0.073 0.02 0 0.036 -0.045 0.039 0.021 -0.074 -0.066

9 1 0.031 -0.028 0 0.076 0.017 -0.149 -0.05 -0.016 0.013 0.106 0.123 -0.006 -0.012 0.064 0.108 -0.151 0.073 0.02 0 0.151 -0.045 0.088 0.021 -0.074 0

9 1 0.031 -0.183 0 0.061 0.017 -0.149 -0.05 -0.016 0.013 0.106 0.123 -0.006 -0.012 0.064 0.108 -0.047 0.073 0.02 0 0.151 -0.045 0.138 0.021 -0.074 0

9 1 0.031 -0.028 0 0.061 0.017 -0.152 -0.05 -0.016 0 -0.057 0.123 0 -0.012 0.064 0.108 -0.069 0.073 0.004 0 0.036 -0.045 0.039 0.021 0.128 0

9 1 0.031 -0.028 0 0.061 0.017 -0.149 -0.05 -0.016 0.013 0.106 0.123 -0.006 -0.012 0.064 0.108 -0.047 0.073 0.02 0 0.151 -0.179 0.088 0.021 -0.074 0

9 1 0.031 -0.028 -0.032 0.061 0.017 -0.152 -0.05 -0.016 0 0.106 0.123 0 -0.012 0.064 0.108 -0.069 0.073 -0.029 0 0.036 -0.045 0.039 0.021 -0.074 -0.073

9 1 0.031 -0.028 0 0.061 0.017 -0.149 -0.05 -0.016 0.013 0.106 0.123 -0.006 -0.012 0.064 0.108 -0.047 0.073 0.02 0 0.151 -0.045 0.088 0.021 -0.074 -0.089

9 1 0.031 0.077 0 0.061 0.017 -0.149 -0.05 -0.016 0.013 0.106 0.123 -0.142 -0.012 0.064 0.108 -0.047 0.073 0.02 0 0.151 -0.045 0.088 0.021 -0.074 0

9 1 0.031 -0.028 -0.065 0.061 0.017 -0.152 -0.05 -0.016 0 -0.057 0.123 0 -0.012 0.064 0.108 -0.069 0.073 0.02 0 0.036 -0.045 0.039 0.021 0.128 0

9 1 0.031 -0.028 -0.032 0.061 0.017 -0.152 -0.177 -0.016 0 0.106 0.123 0 -0.012 0.064 0.108 -0.069 0.073 -0.029 0 0.036 -0.045 0.039 0.021 -0.074 0

9 1 0.031 -0.028 0 0.061 0.017 -0.152 -0.05 -0.016 0 -0.067 0.123 0 -0.012 0.064 0.108 -0.069 0.073 0.02 0 0.036 -0.045 0.039 0.021 0.128 0

9 1 0.031 -0.028 0 0.061 0.017 -0.124 -0.05 -0.016 -0.014 0.106 0.123 0 -0.012 0.064 0.108 -0.069 0.073 -0.12 0 0.036 -0.045 0.039 0.021 -0.074 0

9 1 0.031 -0.028 0 0.061 0.017 -0.152 -0.05 -0.016 -0.014 0.106 0.127 0 -0.012 0.064 0.108 -0.069 0.073 0.02 0 0.036 -0.045 0.039 0.021 -0.074 0

9 1 0.031 -0.028 0 0.061 0.017 -0.152 -0.05 -0.016 0.073 0.106 0.123 0 -0.012 0.064 0.108 -0.069 0.073 0.02 0 0.036 -0.045 0.039 0.021 -0.074 0

9 1 0.031 -0.028 0 0.061 0.017 -0.152 -0.05 -0.016 -0.014 0.106 0.123 0 -0.012 0.064 0.108 -0.069 0.073 0.02 0 0.036 -0.045 0.039 0.021 -0.074 0.196

9 1 0.031 -0.028 0 0.061 0.017 -0.149 -0.05 -0.016 0.013 0.106 0.123 -0.142 -0.012 0.064 0.082 -0.047 0.073 0.02 0 0.151 -0.045 0.088 0.021 -0.074 0

9 1 0.031 -0.028 0 0.061 0.017 -0.149 -0.05 -0.016 0.013 0.106 0.123 -0.006 -0.012 0.064 0.108 -0.047 0.073 0.02 0 0.104 -0.045 0.088 0.021 -0.074 0

9 1 0.031 -0.028 0 0.061 -0.167 -0.149 -0.05 -0.016 0.013 0.106 0.123 -0.142 -0.012 0.064 0.108 -0.047 0.073 0.02 0 0.151 -0.045 0.088 0.021 -0.074 0

9 1 0.031 -0.145 0 0.061 0.017 -0.152 -0.05 -0.016 0 0.106 0.123 0 -0.012 0.064 0.108 -0.069 0.073 0.02 0 0.036 -0.154 0.039 0.021 -0.074 0

9 1 0.031 -0.028 0 0.061 0.017 -0.149 -0.05 -0.016 0.013 0.106 0.123 -0.142 -0.012 0.064 0.202 -0.047 0.073 0.02 0 0.151 -0.045 0.088 0.021 -0.074 0

9 1 0.031 -0.171 0 0.061 0.017 -0.149 -0.05 -0.016 0.013 0.106 0.123 -0.142 -0.012 0.064 0.108 -0.047 0.073 0.02 0 0.151 -0.045 0.088 0.021 -0.074 0

9 1 0.031 -0.028 0 0.061 0.017 -0.152 -0.05 -0.016 0 0.106 0.123 0 -0.012 0.064 0.108 -0.069 -0.063 0.02 0 0.036 -0.154 0.039 0.021 -0.074 0

9 1 0.031 -0.028 0 0.061 0.017 -0.152 -0.05 -0.016 -0.014 0.106 0.123 0 -0.012 0.064 0.108 -0.069 0.073 0.02 0 0.055 -0.045 0.039 0.021 -0.074 0

9 1 0.031 -0.028 0 0.061 0.017 -0.149 -0.05 -0.016 0.013 0.076 0.123 -0.006 -0.012 0.064 0.108 -0.129 0.073 0.02 0 0.151 -0.045 0.138 0.021 -0.074 0

9 1 0.031 -0.028 0 0.061 0.017 -0.149 -0.05 -0.016 0.013 0.106 0.123 -0.006 -0.012 0.064 0.108 -0.047 0.073 0.02 0 0.205 -0.045 0.088 0.021 -0.074 0

9 1 0.031 -0.028 0 0.061 0.017 -0.149 -0.05 -0.016 0.013 0.106 0.123 -0.006 -0.012 0.064 0.108 -0.047 0.073 0.02 0 0.151 -0.045 0.228 0.021 -0.26 0

9 1 0.031 -0.028 0 0.184 0.017 -0.149 -0.05 -0.016 0.013 0.106 0.123 -0.142 -0.012 0.064 0.108 -0.047 0.073 0.02 0 0.151 -0.045 0.088 0.021 -0.074 0

9 1 0.031 -0.028 0 0.061 0.017 -0.152 -0.05 -0.016 0 0.106 0.123 0 0.148 0.064 0.108 0.066 0.073 0.02 0 0.036 -0.045 0.039 0.021 -0.074 0

9 1 0.031 -0.028 0 0.061 0.017 -0.152 -0.093 -0.016 0 0.106 0.123 0 -0.012 0.064 0.108 -0.069 0.073 0.02 0 0.036 -0.045 0.039 0.021 -0.074 0

9 1 0.031 -0.028 0 0.061 0.017 -0.149 -0.05 -0.016 0.013 0.106 0.123 -0.006 -0.012 0.064 0.108 -0.047 0.073 0.02 0 0.257 -0.045 0.088 0.021 -0.074 0

9 1 0.031 -0.028 0 0.061 0.017 -0.152 -0.05 -0.03 0 0.106 0.123 0 -0.012 0.064 0.108 -0.069 0.073 0.238 0 0.036 -0.045 0.039 0.021 -0.074 0

9 1 0.031 -0.028 0 0.061 0.017 -0.165 -0.05 -0.016 0 0.106 0.123 0 -0.012 0.064 0.108 -0.069 0.073 0.02 0 0.036 -0.154 0.039 0.021 -0.074 0

9 1 0.031 -0.028 0 0.061 0.017 -0.152 -0.05 -0.016 0 0.106 0.123 0 0.123 0.064 0.108 -0.069 0.073 0.02 0 0.036 -0.045 0.039 0.021 -0.074 0

9 1 0.031 -0.063 0 0.061 0.017 -0.152 -0.05 -0.016 0 0.106 0.123 0 -0.012 0.064 0.108 -0.069 0.073 0.02 0 0.036 -0.045 0.039 0.021 -0.074 0

9 1 0.031 -0.028 0 0.061 0.017 -0.152 -0.05 -0.016 0 -0.057 0.123 0 -0.012 0.064 0.108 -0.069 0.073 0.02 0 0.036 -0.105 0.039 0.021 0.128 0

9 1 0.031 -0.028 0 0.061 0.017 -0.173 -0.05 -0.016 0 0.106 0.123 0 -0.012 0.064 0.108 -0.069 0.073 0.02 0 0.114 -0.154 0.039 0.021 -0.074 0

9 1 -0.04 -0.028 0 0.061 0.017 -0.149 -0.05 -0.016 0.013 0.106 0.123 -0.006 -0.012 0.064 0.108 -0.047 0.073 0.02 0 0.151 -0.045 0.138 0.021 -0.074 0

9 1 0.031 -0.028 0 0.061 0.017 -0.152 -0.05 -0.016 0 0.106 0.123 0 -0.012 0.064 0.108 -0.069 0.183 0.02 0 0.036 -0.045 0.039 0.021 -0.074 0

9 1 0.031 -0.028 0 -0.032 0.017 -0.149 -0.05 -0.016 0.013 0.106 0.123 -0.006 -0.012 0.064 0.108 -0.101 0.021 0.02 0 0.151 -0.045 0.088 0.021 -0.074 0

9 1 0.031 -0.028 0 0.061 0.017 -0.152 0.055 -0.016 0 0.106 0.123 0 -0.012 0.064 0.108 -0.069 0.073 -0.029 0 -0.048 -0.045 0.039 0.021 -0.074 0

10 817 0.292 0.13 -0.095 0.043 -0.1 0 -0.187 -0.086 0 0.222 -0.136 0.313 -0.035 0.073 -0.114 0.087 0 0.001 -0.053 -0.058 0.167 -0.171 -0.151 0 0

10 398 0.257 0.13 -0.095 0.043 -0.1 0 -0.187 -0.107 0 0.164 -0.136 0.313 -0.035 0.039 -0.114 0.087 0 0.001 -0.134 0 0.167 -0.171 -0.151 0 0

10 366 0.292 0.13 -0.095 0.043 -0.1 0 -0.187 -0.107 0 0.222 -0.136 0.313 -0.035 0.039 -0.114 0.087 0 0.001 -0.053 0 0.167 -0.171 -0.151 0 0

10 240 0.292 0.13 -0.095 0.043 -0.1 0 -0.187 -0.107 0 0.164 -0.118 0.313 -0.035 0.039 -0.114 0.087 -0.159 0.001 -0.053 -0.17 0.167 -0.171 -0.151 0 0

10 143 0.292 0.13 -0.095 0.043 -0.1 0 -0.187 -0.107 0 0.222 -0.136 0.313 -0.035 0.048 -0.114 0.087 0 0.001 -0.053 0 0.167 -0.171 -0.139 -0.027 0

10 121 0.292 0.13 -0.095 0.043 -0.1 0 -0.187 -0.107 0 0.164 -0.136 0.313 -0.035 0.039 -0.114 0.087 0 0.001 -0.053 0 0.167 -0.171 -0.151 0 0

10 105 0.292 0.13 -0.095 0.043 -0.1 0 -0.187 -0.107 0 0.164 -0.118 0.313 -0.035 0.039 -0.114 0.087 0 0.001 -0.053 -0.17 0.167 -0.171 -0.151 0 0

10 85 0.257 0.13 -0.095 0.043 -0.1 0 -0.187 -0.177 0 0.164 -0.136 0.329 -0.035 0.039 -0.114 0.087 0 0.001 -0.134 0 0.167 -0.171 -0.151 0 0

10 83 0.257 0.13 -0.095 0.043 -0.1 0 -0.187 -0.107 0 0.164 -0.136 0.313 -0.035 0.039 -0.114 0.087 0 0.001 -0.053 0 0.167 -0.171 -0.151 0 0

10 77 0.292 0.13 -0.095 0.043 -0.1 0 -0.187 -0.107 0 0.164 -0.118 0.313 -0.035 0.039 -0.114 0.09 0 0.001 -0.053 0 0.167 -0.171 -0.151 0 0

10 62 0.292 0.13 -0.095 0.043 -0.1 0 -0.187 -0.086 0 0.222 -0.136 0.313 -0.035 0.073 -0.114 0.087 0 0.055 -0.053 -0.058 0.167 -0.171 -0.151 0 0

10 65 0.292 0.13 -0.095 0.043 -0.1 0 -0.187 -0.107 0 0.222 -0.136 0.313 -0.035 0.039 -0.114 0.087 0.104 0.001 -0.053 0 0.167 -0.171 -0.151 0 0

10 55 0.292 0.13 -0.095 0.043 -0.1 0 -0.187 -0.107 0 0.164 -0.118 0.313 -0.035 0.109 -0.114 0.087 0 0.001 -0.053 -0.17 0.167 -0.171 -0.151 0 0

10 40 0.292 0.13 -0.095 0.043 -0.1 0 -0.187 -0.107 0 0.164 -0.136 0.313 -0.035 0.124 -0.114 0.087 0 0.001 -0.053 0 0.167 -0.171 -0.151 0 0

10 40 0.292 0.13 -0.095 0.043 -0.1 0 -0.187 -0.107 -0.117 0.222 -0.136 0.313 -0.035 0.039 -0.114 0.087 0 0.001 -0.053 0 0.167 -0.171 -0.151 0 0

10 39 0.292 0.13 -0.095 0.043 -0.1 0 -0.187 -0.086 0 0.222 -0.136 0.313 -0.035 0.073 -0.114 0.012 0 0.001 -0.053 -0.058 0.167 -0.171 -0.151 0 0

10 32 0.257 0.13 -0.095 0.043 -0.1 0 -0.187 -0.107 0 0.164 -0.136 0.329 -0.035 0.039 -0.114 0.087 0 0.001 -0.134 0 0.167 -0.171 -0.151 0 0

10 30 0.257 0.13 -0.095 0.043 -0.1 0 -0.187 -0.107 -0.029 0.164 -0.136 0.313 -0.035 0.039 -0.114 0.087 0 0.001 -0.134 0 0.167 -0.171 -0.151 0 0

10 22 0.292 0.13 -0.095 0.043 -0.1 0 -0.187 -0.107 0 0.164 -0.136 0.313 0.02 0.039 -0.114 0.087 0 0.001 -0.053 0 0.167 -0.171 -0.151 0 0

10 22 0.292 0.13 -0.095 0.043 -0.1 0 -0.187 -0.209 0 0.164 -0.118 0.313 -0.035 0.039 -0.114 0.087 -0.159 0.001 -0.053 -0.17 0.167 -0.171 -0.151 0 0

10 20 0.292 0.155 -0.095 0.043 -0.1 0 -0.187 -0.086 0 0.222 -0.136 0.313 -0.035 0.073 -0.114 0.087 0 0.001 -0.053 -0.058 0.167 -0.171 -0.151 0 0

10 23 0.292 0.13 -0.095 0.043 -0.1 0 -0.191 -0.107 0 0.164 -0.136 0.313 -0.035 0.039 -0.114 0.087 0 0.001 -0.053 0 0.167 -0.171 -0.151 0 0

10 17 0.292 0.13 -0.095 0.043 -0.1 0 -0.187 -0.107 0 0.164 -0.118 0.313 -0.035 0.039 -0.114 0.087 0 0.001 -0.053 0 0.167 -0.171 -0.151 0 0

10 12 0.292 0.13 -0.095 0.043 -0.1 0 -0.187 -0.086 0 0.222 -0.136 0.313 -0.035 0.073 -0.114 0.087 0 0.068 -0.053 -0.058 0.167 -0.171 -0.151 0 0

10 12 0.257 0.13 -0.095 0.043 -0.1 0 -0.248 -0.107 0 0.164 -0.136 0.313 -0.035 0.039 -0.114 0.087 0 0.001 -0.134 0 0.167 -0.171 -0.151 0 0

10 10 0.292 0.13 -0.095 0.043 -0.1 0 -0.187 -0.107 -0.105 0.222 -0.136 0.313 -0.035 0.039 -0.114 0.087 0 0.001 -0.053 0 0.167 -0.171 -0.151 0 0

10 10 0.292 0.13 -0.095 0.069 -0.1 0 -0.187 -0.107 0 0.222 -0.136 0.313 -0.035 0.039 -0.114 0.087 0 0.001 -0.053 0 0.167 -0.171 -0.151 0 0

10 9 0.334 0.12 -0.113 0.043 -0.1 0 -0.187 -0.107 0 0.164 -0.136 0.364 -0.074 0.039 -0.114 0.145 0 0.001 -0.053 0 0.167 -0.171 -0.151 0 0

10 9 0.257 0.13 -0.095 0.043 -0.1 0 -0.187 -0.107 0 0.164 -0.136 0.24 -0.035 0.039 -0.114 0.087 0 0.001 -0.134 0 0.167 -0.171 -0.151 0 0

10 9 0.292 0.13 -0.095 0.043 -0.179 0 -0.187 -0.107 0 0.164 -0.136 0.313 -0.035 0.039 -0.114 0.087 0 0.001 -0.053 0 0.167 -0.171 -0.151 0 0

10 8 0.257 0.13 -0.116 0.043 -0.1 0 -0.187 -0.107 0 0.164 -0.136 0.313 -0.035 0.039 -0.114 0.087 0 0.001 -0.053 0 0.167 -0.171 -0.151 0 0

10 8 0.292 0.13 -0.095 0.043 -0.1 0 -0.187 -0.086 0 0.222 -0.136 0.313 -0.035 0.073 -0.114 0.087 0 0.001 -0.053 -0.058 0.167 -0.171 -0.151 -0.008 0

10 7 0.292 -0.138 -0.095 0.043 -0.1 0 -0.187 -0.086 0 0.222 -0.136 0.313 -0.035 0.073 -0.114 0.087 0 0.001 -0.053 -0.058 0.167 -0.171 -0.151 0 0

10 6 0.292 0.13 -0.095 0.043 -0.1 0 -0.187 -0.107 0 0.164 -0.118 0.313 -0.035 0.039 -0.114 0.087 0.075 0.001 -0.053 -0.17 0.167 -0.171 -0.151 0 0

10 6 0.292 0.13 -0.095 0.043 -0.1 0 -0.187 -0.209 0 0.2 -0.118 0.313 -0.035 0.039 -0.114 0.087 -0.159 0.001 -0.053 -0.17 0.167 -0.171 -0.151 0 0

10 5 0.292 0.13 -0.095 0.043 -0.1 0 -0.187 -0.107 0 0.222 -0.136 0.313 -0.035 0.039 -0.114 0.087 0 0.008 -0.053 0 0.167 -0.171 -0.151 0 0

10 5 0.292 0.13 -0.095 0.043 -0.1 0 -0.187 -0.086 0 0.222 -0.136 0.313 -0.035 0.202 -0.114 0.087 0 0.001 -0.053 -0.058 0.167 -0.171 -0.151 0 0

10 5 0.292 0.13 -0.095 0.043 -0.1 0 -0.187 -0.107 0 0.133 -0.136 0.313 -0.035 0.039 -0.114 0.087 0 0.001 -0.053 0 0.167 -0.171 -0.151 0 0

10 5 0.292 0.171 -0.095 0.043 -0.1 0 -0.187 -0.107 0 0.164 -0.136 0.313 -0.035 0.039 -0.114 0.087 0 0.001 -0.053 0 0.167 -0.171 -0.151 0 0

10 7 0.292 0.13 -0.095 0.043 -0.1 0 -0.187 -0.107 0 0.222 -0.136 0.313 -0.035 0.039 -0.114 0.087 0 0.001 -0.053 -0.085 0.167 -0.171 -0.151 0 0

10 4 0.292 0.13 -0.095 0.157 -0.1 0 -0.187 -0.086 0 0.222 -0.136 0.313 -0.035 0.073 -0.082 0.087 0 0.001 -0.053 -0.058 0.167 -0.171 -0.151 0 0

10 4 0.292 0.13 -0.095 0.043 -0.011 0 -0.187 -0.107 0 0.164 -0.136 0.313 -0.035 0.039 -0.114 0.087 0 0.001 -0.053 0 0.167 -0.171 -0.151 0 0

10 4 0.292 0.13 -0.095 0.043 -0.1 0 -0.187 -0.086 0 0.222 -0.136 0.313 -0.035 0.073 -0.114 0.087 0 0.001 -0.053 -0.058 0.167 -0.171 -0.151 0 0.005

10 4 0.292 0.13 -0.194 0.043 -0.1 0 -0.187 -0.086 0 0.222 -0.136 0.313 -0.035 0.073 -0.114 0.087 0 0.001 -0.053 -0.058 0.167 -0.171 -0.151 0 0

10 4 0.292 0.13 -0.095 0.043 -0.1 0 -0.187 0.014 0 0.222 -0.136 0.313 -0.035 0.073 -0.114 0.087 0 0.055 -0.053 -0.058 0.167 -0.171 -0.151 0 0

10 4 0.292 0.155 -0.095 0.043 -0.1 0 -0.187 -0.086 0 0.222 -0.136 0.313 -0.035 0.073 -0.114 0.087 0 -0.119 -0.053 -0.058 0.167 -0.171 -0.151 0 0

10 4 0.292 0.13 -0.136 0.043 -0.1 0 -0.187 -0.107 0 0.222 -0.136 0.313 -0.035 0.048 -0.114 0.087 0 0.001 -0.053 0 0.167 -0.171 -0.139 -0.027 0

10 4 0.292 0.13 -0.095 0.043 -0.1 0 -0.187 -0.086 0 0.222 -0.136 0.313 -0.035 0.073 -0.114 0.087 0 0.001 -0.053 -0.271 0.167 -0.171 -0.151 0 0

10 3 0.292 0.13 -0.095 0.043 -0.1 0 -0.187 -0.107 0 0.222 -0.136 0.313 -0.035 0.048 -0.114 0.087 0 0.001 -0.053 0 0.167 -0.164 -0.139 -0.027 0

10 4 0.292 0.13 -0.095 0.043 -0.1 0 -0.187 -0.107 0 0.164 -0.136 0.313 -0.035 -0.037 -0.114 0.087 0 0.001 -0.053 0 0.167 -0.171 -0.151 0 0

10 3 0.292 0.13 -0.095 0.043 -0.1 0 -0.187 -0.107 0 0.164 -0.118 0.313 -0.035 0.109 -0.114 0.087 0 -0.157 -0.053 -0.17 0.167 -0.171 -0.151 0 0

10 3 0.257 0.13 -0.095 0.043 -0.1 0 -0.187 -0.107 0 0.164 -0.136 0.313 -0.035 0.039 -0.114 0.087 0 0.001 -0.098 0 0.167 -0.171 -0.151 0 0

10 2 0.257 0.13 -0.095 0.043 -0.1 0 -0.187 -0.107 0 0.164 -0.136 0.329 -0.035 0.039 -0.114 0.087 0 0.033 -0.134 0 0.167 -0.171 -0.151 0 0

10 2 0.292 0.13 -0.095 0.043 -0.1 0 -0.187 -0.107 0 0.222 -0.136 0.313 -0.035 0.039 -0.114 0.087 -0.051 0.008 -0.053 0 0.167 -0.171 -0.151 0 0

10 2 0.292 0.13 -0.095 0.043 -0.1 0 -0.187 -0.107 0 0.222 -0.136 0.313 -0.035 0.039 -0.114 0.087 0 0.001 -0.053 0 0.167 -0.171 -0.151 0 0.06

10 2 0.292 0.13 -0.095 0.043 -0.1 0 -0.187 -0.086 0 0.222 -0.136 0.313 -0.035 0.073 -0.114 0.087 0 -0.143 -0.053 -0.058 0.167 -0.171 -0.151 0 0

10 2 0.292 0.13 -0.095 0.043 -0.091 0 -0.187 -0.107 0 0.164 -0.118 0.313 -0.035 0.039 -0.114 0.087 0 0.001 -0.053 0 0.167 -0.171 -0.151 0 0

10 2 0.292 0.13 -0.095 0.043 -0.1 0 -0.187 -0.107 0 0.222 -0.136 0.313 -0.035 0.039 -0.114 0.116 0 0.001 -0.053 0 0.167 -0.171 -0.151 0 0

10 2 0.257 0.13 -0.095 0.043 -0.056 0 -0.187 -0.107 0 0.164 -0.136 0.313 -0.035 0.039 -0.114 0.087 0 0.001 -0.053 0 0.167 -0.171 -0.151 0 0

10 2 0.292 0.13 -0.095 0.043 -0.1 0 -0.187 -0.086 0 0.222 -0.136 0.313 -0.035 0.073 -0.114 0.087 0 0.001 -0.053 -0.058 0.167 -0.093 -0.151 -0.008 0

10 2 0.292 0.13 -0.095 0.043 -0.1 0 -0.187 -0.086 0 0.222 0.106 0.313 -0.035 0.073 -0.114 0.087 0 0.001 -0.053 -0.058 0.167 -0.171 -0.151 0 0

10 2 0.292 0.13 -0.095 0.043 -0.1 0 -0.187 -0.107 0 0.222 -0.136 0.313 -0.035 -0.066 -0.114 0.087 0 0.001 -0.053 0 0.167 -0.171 -0.139 -0.027 0

10 2 0.292 0.13 -0.095 0.043 -0.1 0 -0.187 -0.107 0 0.164 -0.136 0.313 -0.035 0.039 -0.114 -0.023 0 0.001 -0.053 0 0.167 -0.171 -0.151 0 0

10 2 0.292 0.13 -0.095 0.043 -0.1 0 -0.187 -0.107 0 0.164 -0.136 0.313 -0.035 0.124 -0.114 0.085 0 0.001 -0.053 0 0.167 -0.171 -0.151 0 0

10 2 0.292 0.13 -0.095 0.043 -0.1 0 -0.187 -0.086 0 0.222 -0.136 0.313 -0.035 0.073 -0.114 0.087 0 0.001 -0.053 -0.058 0.167 -0.433 -0.151 0 0

10 2 0.292 0.13 -0.095 0.043 -0.1 0 -0.187 -0.107 0 0.164 -0.118 0.313 0.05 0.039 -0.114 0.087 -0.159 0.001 -0.053 -0.17 0.167 -0.171 -0.151 0 0

10 2 0.454 0.13 -0.095 0.043 -0.1 0 -0.187 -0.107 0 0.164 -0.136 0.313 -0.035 0.039 -0.114 0.087 0 0.001 -0.134 0 0.167 -0.171 -0.151 0 0

10 2 0.257 0.13 -0.095 0.043 -0.1 0 -0.187 -0.107 0 0.164 -0.136 0.313 -0.035 0.039 -0.09 0.087 0 0.001 -0.134 0 0.167 -0.171 -0.151 0 0

10 2 0.292 0.13 -0.095 0.043 -0.1 0 -0.187 -0.107 -0.059 0.222 -0.136 0.313 -0.035 0.039 -0.114 0.087 0 0.001 -0.053 0 0.167 -0.171 -0.151 0 0

10 2 0.292 0.13 -0.095 0.043 -0.1 0 -0.187 -0.107 0 0.164 -0.118 0.378 -0.035 0.039 -0.114 0.087 0 0.001 -0.053 -0.17 0.167 -0.171 -0.151 0 0

10 2 0.292 0.13 -0.095 0.043 -0.1 0 -0.187 -0.086 0 0.222 -0.136 0.313 -0.035 0.073 -0.114 0.087 0.124 0.001 -0.053 -0.058 0.167 -0.171 -0.151 0 0

10 2 0.292 0.13 -0.095 0.043 -0.1 0 -0.187 -0.102 0 0.164 -0.118 0.313 -0.035 0.039 -0.114 0.09 0 0.001 -0.053 0 0.167 -0.171 -0.151 0 0

10 2 0.292 0.13 -0.217 0.043 -0.1 0 -0.187 -0.086 0 0.222 -0.136 0.313 -0.035 0.073 -0.114 0.012 0 0.001 -0.053 -0.058 0.167 -0.171 -0.151 0 0

10 1 0.292 0.13 -0.095 0.043 -0.1 0 -0.187 -0.107 0 0.164 -0.118 0.313 -0.035 0.039 -0.114 0.087 -0.159 0.001 -0.053 -0.17 0.167 -0.171 -0.151 0 -0.111

10 1 0.292 0.13 0.056 0.043 -0.1 0 -0.187 -0.107 0 0.164 -0.136 0.313 0.02 0.039 -0.114 0.087 0 0.001 -0.053 0 0.167 -0.171 -0.151 0 0

10 1 0.292 0.13 -0.095 0.157 -0.1 0 -0.187 -0.086 0 0.222 -0.136 0.313 -0.035 0.073 0.004 0.087 0 0.001 -0.053 -0.058 0.167 -0.171 -0.151 0 0

10 1 0.257 0.13 -0.095 0.043 -0.1 0 -0.187 -0.107 0 0.164 -0.136 0.367 -0.035 0.039 -0.114 0.087 0 0.001 -0.134 0 0.167 -0.171 -0.151 0 0

10 1 0.292 0.13 -0.095 0.043 -0.1 0 -0.187 -0.086 0 0.222 -0.136 0.313 -0.035 0.073 -0.114 0.139 0 0.001 -0.053 -0.058 0.167 -0.171 -0.151 0 0

10 1 0.292 0.13 -0.095 0.043 -0.1 0 -0.187 -0.107 0 0.164 -0.118 0.313 -0.035 0.039 -0.114 0.087 0 0.001 -0.053 -0.17 0.167 -0.171 -0.151 0 -0.039

10 1 0.257 0.13 -0.095 0.18 -0.1 0 -0.187 -0.107 0 0.164 -0.136 0.329 -0.035 0.039 -0.114 0.087 0 0.033 -0.134 0 0.167 -0.171 -0.151 0 0

10 1 0.257 0.13 -0.095 0.043 -0.1 0 -0.187 -0.107 0.057 0.164 -0.136 0.313 -0.035 0.039 -0.114 0.087 0 0.001 -0.053 0 0.167 -0.171 -0.151 0 0

10 1 0.292 0.13 -0.095 0.043 -0.1 0 -0.187 -0.107 0 0.222 -0.136 0.313 -0.035 0.075 -0.114 0.087 0 0.001 -0.053 0 0.167 -0.171 -0.151 0 0

10 1 0.292 0.13 -0.095 0.043 -0.1 0 -0.187 -0.107 0 0.164 -0.118 0.313 -0.035 0.039 -0.114 0.087 0 0.001 -0.053 -0.17 0.167 -0.227 -0.151 0 0

10 1 0.292 0.13 -0.095 0.043 -0.1 0.125 -0.187 -0.107 0 0.222 -0.136 0.313 -0.035 0.039 -0.114 0.087 0.104 0.001 -0.053 0 0.167 -0.171 -0.151 0 0

10 1 0.257 0.15 -0.095 0.043 -0.1 0 -0.187 -0.107 0 0.164 -0.136 0.313 -0.035 0.039 -0.114 0.087 0 0.001 -0.053 0 0.167 -0.171 -0.151 0 0

10 1 0.257 0.13 -0.028 0.043 -0.1 0 -0.187 -0.107 0 0.164 -0.136 0.313 -0.035 0.039 -0.114 0.087 0 0.001 -0.134 0 0.167 -0.171 -0.151 0 0

10 1 0.292 0.13 -0.095 0.043 -0.1 0 -0.187 -0.107 0 0.222 -0.136 0.313 -0.035 0.048 -0.308 0.087 0 0.001 -0.053 0 0.167 -0.171 -0.139 -0.027 0

10 1 0.292 0.13 -0.095 0.043 -0.018 0 -0.187 -0.107 0 0.222 -0.136 0.313 -0.035 0.039 -0.114 0.087 0 0.001 -0.053 0 0.167 -0.171 -0.151 0 0

10 1 0.292 0.13 -0.033 0.043 -0.1 0 -0.187 -0.086 0 0.222 -0.136 0.313 -0.035 0.073 -0.114 0.087 0 0.001 -0.053 -0.058 0.167 -0.171 -0.151 0 0

10 1 0.257 0.13 -0.095 0.043 -0.1 0 -0.187 -0.107 0 0.164 -0.136 0.313 -0.035 -0.148 -0.114 0.087 0 0.001 -0.134 0 0.167 -0.171 -0.151 0 0

10 1 0.292 0.13 -0.095 0.043 -0.1 0 -0.187 -0.086 0 0.222 -0.136 0.313 -0.035 0.073 -0.114 0.087 0 0.001 -0.053 -0.058 0.167 -0.171 -0.151 0.073 0

10 1 0.368 0.13 -0.095 0.043 -0.1 0 -0.187 -0.086 0 0.222 -0.136 0.313 -0.035 0.202 -0.114 0.087 0 0.001 -0.053 -0.058 0.167 -0.171 -0.151 0 0

10 1 0.292 0.216 -0.095 0.043 -0.1 0 -0.187 -0.107 0 0.222 -0.136 0.313 -0.035 0.039 -0.114 0.087 0 0.001 -0.053 0 0.167 -0.171 -0.151 0 0

10 1 0.292 0.13 -0.029 0.043 -0.1 0 -0.187 -0.107 0 0.164 -0.118 0.313 -0.035 0.039 -0.114 0.09 0 0.001 -0.053 0 0.167 -0.171 -0.151 0 0

10 1 0.257 0.13 -0.095 0.043 -0.1 0.109 -0.187 -0.107 0 0.164 -0.136 0.313 -0.035 0.039 -0.114 0.087 0 0.001 -0.134 0 0.167 -0.171 -0.151 0 0

10 1 0.292 0.13 -0.095 0.043 -0.1 0 -0.187 -0.107 0 0.222 -0.136 0.313 -0.035 0.039 -0.114 0.087 0 0.001 0.084 0 0.167 -0.171 -0.151 0 0

10 1 0.257 0.13 -0.095 0.043 -0.1 0 -0.187 -0.107 0 0.164 -0.136 0.313 -0.035 0.039 -0.114 0.087 0 0.001 -0.134 0.03 0.167 -0.171 -0.151 0 0

10 1 0.292 0.13 -0.078 0.043 -0.1 0 -0.187 -0.107 0 0.164 -0.118 0.313 -0.035 0.039 -0.114 0.087 -0.159 0.001 -0.053 -0.17 0.167 -0.171 -0.151 0 0

10 1 0.292 0.13 -0.095 0.043 -0.1 0 -0.187 -0.107 0 0.222 -0.167 0.313 -0.035 0.039 -0.114 0.087 0 0.001 -0.053 0 0.167 -0.171 -0.151 0 0

10 1 0.257 0.13 -0.095 0.043 -0.1 0 -0.187 -0.107 0 0.164 -0.136 0.313 -0.035 0.039 -0.114 0.087 0 0.001 -0.134 0 0.078 -0.171 -0.151 0 0

10 1 0.257 0.13 -0.095 0.043 -0.1 0 -0.187 -0.107 0 0.164 -0.136 0.313 -0.035 0.039 -0.114 0.087 0 0.001 -0.134 0 0.167 -0.171 -0.151 0 0.099

10 1 0.292 0.13 -0.095 0.043 -0.1 0.148 -0.187 -0.107 0 0.164 -0.118 0.313 -0.035 0.039 -0.114 0.087 -0.159 0.001 -0.053 -0.17 0.167 -0.171 -0.151 0 0

10 1 0.189 0.13 -0.095 0.043 -0.1 0 -0.187 -0.107 0 0.164 -0.118 0.313 -0.035 0.039 -0.114 0.087 0 0.001 -0.053 -0.17 0.167 -0.171 -0.151 0 0

10 1 0.292 0.13 -0.095 0.043 -0.1 0 -0.187 -0.079 0 0.222 -0.136 0.313 -0.035 0.073 -0.114 0.087 0 0.001 -0.053 -0.058 0.167 -0.171 -0.151 0 0

10 1 0.292 0.13 -0.095 0.043 -0.1 -0.018 -0.187 -0.086 0 0.222 -0.136 0.313 -0.035 0.073 -0.114 0.087 0 0.001 -0.053 -0.058 0.167 -0.171 -0.151 0 0

10 1 0.292 0.13 -0.095 0.043 -0.1 0 -0.187 -0.086 0 0.222 -0.136 0.313 -0.035 0.25 -0.114 0.087 0 0.055 -0.053 -0.058 0.167 -0.171 -0.151 0 0

10 1 0.292 0.13 -0.095 0.043 -0.1 0 -0.187 -0.086 0 0.222 -0.136 0.313 -0.035 0.129 -0.114 0.087 0 0.001 -0.053 -0.058 0.167 -0.171 -0.151 0 0

10 1 0.292 0.13 -0.095 0.043 -0.1 0 -0.187 -0.086 0 0.222 -0.136 0.313 -0.035 0.073 -0.114 0.087 0 -0.053 -0.053 -0.058 0.167 -0.171 -0.151 0 0

10 1 0.292 0.13 -0.095 0.043 -0.1 0 -0.187 -0.107 0 0.164 -0.118 0.159 -0.035 0.039 -0.114 0.087 -0.159 0.001 -0.053 -0.17 0.167 -0.171 -0.151 0 0

10 1 0.292 0.13 -0.095 0.043 -0.1 0 -0.187 -0.107 0 0.164 -0.118 0.313 -0.035 0.039 -0.114 0.087 -0.012 0.001 -0.053 -0.17 0.167 -0.171 -0.151 0 0

10 1 0.292 0.13 -0.095 0.043 -0.1 0 -0.187 -0.107 -0.117 0.222 -0.136 0.2 -0.035 0.039 -0.114 0.087 0 0.001 -0.053 0 0.167 -0.171 -0.151 0 0

10 1 0.292 0.13 -0.095 0.043 -0.1 0 -0.187 -0.107 0.085 0.222 -0.136 0.313 -0.035 0.048 -0.114 0.087 0 0.001 -0.053 0 0.167 -0.171 -0.139 -0.027 0

10 1 0.292 0.13 -0.083 0.043 -0.1 0 -0.187 -0.086 0 0.222 -0.136 0.313 -0.035 0.073 -0.114 0.012 0 0.001 -0.053 -0.058 0.167 -0.171 -0.151 0 0

10 1 0.292 0.13 -0.095 0.043 -0.1 0 -0.187 -0.107 0 0.164 -0.118 0.313 -0.035 0.039 -0.004 0.087 -0.159 0.001 -0.053 -0.17 0.167 -0.171 -0.151 0 0

10 1 0.292 0.13 -0.095 -0.013 -0.1 0 -0.187 -0.086 0 0.222 -0.136 0.313 -0.035 0.073 -0.114 0.087 0 0.001 -0.053 -0.058 0.167 -0.171 -0.151 0 0

10 1 0.292 0.13 -0.095 0.043 -0.1 0 -0.187 -0.086 0 0.222 -0.136 0.313 -0.035 0.073 -0.114 0.087 0 -0.112 -0.053 -0.058 0.167 -0.171 -0.151 0 0

10 1 0.292 0.13 -0.095 0.043 -0.1 0 -0.187 -0.086 0 0.222 -0.136 0.313 -0.035 0.073 -0.114 0.087 0 -0.084 -0.053 -0.058 0.167 -0.171 -0.151 0 0

10 1 0.257 0.13 -0.095 0.043 -0.1 0 -0.187 -0.107 0 0.164 -0.136 0.313 -0.035 0.039 -0.114 0.087 0 0.062 -0.134 0 0.167 -0.171 -0.151 0 0

10 1 0.292 0.13 -0.095 0.043 -0.1 0 -0.187 -0.086 0 0.222 -0.136 0.313 -0.035 0.073 -0.046 0.087 0 0.001 -0.053 -0.058 0.167 -0.171 -0.151 0 0

10 1 0.292 0.13 -0.095 0.043 -0.1 0 -0.187 -0.107 0.11 0.164 -0.118 0.313 -0.035 0.039 -0.114 0.087 0 0.001 -0.053 -0.17 0.167 -0.171 -0.151 0 0

10 1 0.292 0.13 -0.095 0.043 -0.1 -0.092 -0.187 -0.086 0 0.222 -0.136 0.313 -0.035 0.073 -0.114 0.087 0 0.001 -0.053 -0.058 0.167 -0.171 -0.151 0 0

10 1 0.292 0.13 -0.095 0.043 -0.1 0 -0.187 -0.086 0 0.222 -0.136 0.313 -0.035 0.202 -0.114 0.087 0 0.001 -0.053 -0.058 0.167 -0.171 -0.151 0 -0.051

10 1 0.292 0.13 -0.095 0.043 -0.1 0 -0.187 -0.086 0 0.222 -0.136 0.313 -0.035 0.073 -0.203 0.087 0 0.001 -0.053 -0.058 0.167 -0.171 -0.151 0 0

10 1 0.292 0.13 -0.095 0.043 -0.1 0 -0.187 -0.107 0 0.164 -0.118 0.201 -0.035 0.109 -0.114 0.087 0 0.001 -0.053 -0.17 0.167 -0.171 -0.151 0 0

10 1 0.292 0.13 -0.065 0.043 -0.1 0 -0.187 -0.107 0 0.164 -0.118 0.313 -0.035 0.039 -0.114 0.087 -0.159 0.001 -0.053 -0.17 0.167 -0.171 -0.151 0 0

10 1 0.257 0.13 -0.095 0.043 -0.1 0 -0.187 -0.177 0 0.164 -0.136 0.329 -0.035 0.039 -0.114 0.13 0 0.001 -0.134 0 0.167 -0.171 -0.151 0 0

10 1 0.292 0.13 -0.095 0.163 -0.1 0 -0.187 -0.086 0 0.222 -0.136 0.313 -0.035 0.073 -0.114 0.087 0 0.001 -0.053 -0.058 0.167 -0.171 -0.151 0 0

10 1 0.292 0.13 -0.095 0.043 -0.1 0 -0.187 -0.107 0 0.222 -0.136 0.313 -0.035 0.048 -0.114 0.087 0 0.001 -0.053 0 0.167 -0.171 -0.139 -0.104 0

10 1 0.292 0.13 -0.095 0.043 -0.1 0 -0.187 -0.086 0 0.222 -0.136 0.313 -0.035 0.073 -0.114 0.087 0 0.001 -0.053 -0.058 0.167 -0.171 -0.223 0 0

10 1 0.292 0.13 -0.095 0.043 -0.1 -0.059 -0.187 -0.107 0 0.164 -0.118 0.313 -0.035 0.039 -0.114 0.087 -0.159 0.001 -0.053 -0.17 0.167 -0.171 -0.151 0 0

11 633 0.179 -0.171 0.102 -0.016 0.148 -0.03 0.109 -0.1 0 0.037 0.404 -0.108 0.056 -0.064 -0.151 -0.626 -0.016 0.103 0.072 -0.024 -0.174 0.071 -0.367 0.006 0.004

11 621 0.179 -0.171 0.102 -0.016 0.148 -0.03 0.109 -0.1 0 -0.007 0.31 -0.108 0.056 -0.064 -0.151 -0.518 0 0.052 0.072 -0.024 -0.174 0.071 -0.427 0.006 0.004

11 406 0.179 -0.171 0.102 -0.016 0.148 -0.03 0.109 -0.1 0 -0.123 0.189 -0.108 0.056 -0.064 -0.151 -0.518 -0.016 0.035 0.072 -0.024 -0.181 0.071 -0.367 0.006 0.004

11 365 0.179 -0.171 0.102 -0.016 0.148 -0.03 0.109 -0.1 0 -0.007 0.31 -0.108 0.056 -0.064 -0.151 -0.518 -0.139 0.052 0.072 -0.024 -0.174 0.071 -0.427 0.006 0.004

11 341 0.179 -0.162 0.102 -0.016 0.148 -0.03 0.109 -0.1 0 -0.007 0.31 -0.108 0.056 -0.064 -0.151 -0.605 -0.401 0.052 0.072 0.111 -0.174 0.071 -0.427 0.006 0.004

11 280 0.179 -0.171 0.102 -0.016 0.148 -0.03 0.109 -0.1 0 -0.007 0.189 -0.108 0.056 -0.05 -0.151 -0.505 -0.016 0.035 0.072 -0.024 -0.181 0.071 -0.367 0.006 0.004

11 202 0.179 -0.171 0.102 -0.016 0.148 -0.03 0.109 -0.1 0 0.108 0.31 -0.108 0.056 -0.064 -0.151 -0.518 0 0.052 0.072 -0.024 -0.174 0.071 -0.427 0.006 0.004

11 192 0.179 -0.171 0.102 -0.016 0.148 -0.03 0.109 -0.1 0 -0.007 0.31 -0.108 0.056 -0.064 -0.151 -0.518 0 0.052 0.072 -0.024 -0.297 0.071 -0.427 0.006 0.004

11 159 0.179 -0.171 0.102 -0.016 0.191 -0.03 0.109 -0.1 0 -0.007 0.189 -0.108 0.056 -0.05 -0.151 -0.505 -0.016 0.035 0.072 -0.024 -0.164 0.071 -0.367 0.006 0.004

11 125 0.179 -0.171 0.102 -0.016 0.148 -0.03 0.109 -0.1 0 -0.007 0.31 -0.108 0.056 -0.064 -0.151 -0.59 0 0.052 0.072 -0.024 -0.174 0.071 -0.427 0.006 0.004

11 120 0.179 -0.171 0.102 -0.016 0.117 -0.03 0.109 -0.1 0 0.037 0.404 -0.108 0.056 -0.064 -0.151 -0.626 -0.016 0.103 0.072 -0.024 -0.174 0.071 -0.367 0.006 0.004

11 96 0.179 -0.171 0.038 -0.016 0.148 -0.03 0.109 -0.1 0 -0.007 0.189 -0.108 0.056 -0.05 -0.151 -0.505 -0.016 0.035 0.072 -0.024 -0.181 0.071 -0.367 0.006 0.004

11 74 0.179 -0.171 0.102 -0.016 0.148 0.01 0.109 -0.1 0 -0.007 0.31 -0.108 0.056 -0.064 -0.151 -0.518 0 0.052 0.072 -0.024 -0.174 0.071 -0.427 0.006 0.004

11 49 0.179 -0.171 0.102 -0.016 0.148 -0.03 0.109 -0.1 0 0.037 0.404 -0.108 0.056 -0.064 -0.151 -0.518 -0.016 0.103 0.072 -0.024 -0.174 0.071 -0.367 0.006 0.004

11 48 0.179 -0.162 0.102 -0.016 0.148 -0.03 0.109 -0.1 0 -0.007 0.31 -0.108 0.056 -0.064 -0.151 -0.518 -0.401 0.052 0.072 0.111 -0.174 0.071 -0.427 0.006 0.004

11 49 0.179 -0.171 0.102 -0.016 0.148 -0.03 0.109 -0.1 0 -0.007 0.189 -0.108 0.056 -0.05 -0.151 -0.505 -0.016 0.035 0.072 -0.024 -0.181 0.071 -0.373 0.006 0.004

11 21 0.179 -0.171 0.102 -0.016 0.148 -0.03 0.109 -0.1 0 0.133 0.31 -0.108 0.056 -0.064 -0.151 -0.518 0 0.052 0.072 -0.024 -0.174 0.071 -0.427 0.006 0.004

11 16 0.179 -0.171 0.102 -0.016 0.148 -0.03 0.109 -0.1 0 0.037 0.404 -0.108 0.056 -0.045 -0.151 -0.626 -0.016 0.103 0.072 -0.024 -0.174 0.071 -0.367 0.006 0.004

11 15 0.179 -0.171 0.056 -0.016 0.148 -0.03 0.109 -0.1 0 -0.007 0.31 -0.108 0.056 -0.064 -0.151 -0.518 0 0.052 0.072 -0.024 -0.297 0.071 -0.427 0.006 0.004

11 14 0.179 -0.171 0.102 -0.016 0.148 -0.03 0.109 -0.1 0 -0.007 0.36 -0.108 0.056 -0.064 -0.151 -0.518 0 0.052 0.072 -0.024 -0.174 0.071 -0.427 0.006 0.004

11 13 0.179 -0.171 0.102 -0.016 0.148 -0.03 0.109 -0.118 0 0.037 0.404 -0.108 0.056 -0.064 -0.151 -0.626 -0.016 0.103 0.072 -0.024 -0.174 0.071 -0.367 0.006 0.004

11 9 0.179 -0.162 0.102 -0.016 0.148 -0.03 0.109 -0.1 0 -0.007 0.328 -0.108 0.056 -0.064 -0.151 -0.605 -0.401 0.052 0.072 0.111 -0.174 0.071 -0.427 0.006 0.004

11 9 0.179 -0.171 0.102 -0.016 0.11 -0.03 0.109 -0.1 0 -0.007 0.31 -0.108 0.056 -0.064 -0.151 -0.518 0 0.052 0.072 -0.024 -0.174 0.071 -0.427 0.006 0.004

11 8 0.179 -0.171 0.102 -0.016 0.117 -0.03 0.109 -0.273 0 0.037 0.404 -0.108 0.056 -0.064 -0.151 -0.626 -0.016 0.103 0.072 -0.024 -0.174 0.071 -0.367 0.006 0.004

11 8 0.179 -0.171 0.102 -0.016 0.148 -0.03 0.109 -0.013 0 -0.007 0.31 -0.108 0.056 -0.064 -0.151 -0.59 0 0.052 0.072 -0.024 -0.174 0.071 -0.427 0.006 0.004

11 7 0.179 -0.171 0.102 -0.048 0.148 -0.03 0.109 -0.1 0 0.037 0.404 -0.108 0.056 -0.064 -0.151 -0.626 -0.016 0.103 0.072 -0.024 -0.174 0.071 -0.367 0.006 0.004

11 7 0.179 -0.171 0.102 -0.016 0.148 -0.03 0.109 -0.1 0 0 0.189 -0.108 0.056 -0.05 -0.151 -0.505 -0.016 0.035 0.072 -0.024 -0.181 0.071 -0.373 0.006 0.004

11 7 0.179 -0.171 0.102 -0.016 0.148 -0.03 0.109 -0.1 0 0.037 0.404 -0.108 0.064 -0.064 -0.151 -0.626 -0.016 0.103 0.072 -0.024 -0.174 0.071 -0.367 0.006 0.004

11 7 0.179 -0.171 0.102 -0.016 0.117 -0.03 0.109 -0.1 0 0.037 0.404 -0.108 0.056 -0.064 -0.151 -0.626 -0.016 0.166 0.072 -0.024 -0.174 0.071 -0.367 0.006 0.004

11 6 0.179 -0.171 0.102 -0.016 0.148 -0.03 0.109 -0.1 0 0.037 0.404 -0.108 0.056 -0.064 -0.151 -0.626 -0.016 0.103 0.072 -0.024 -0.174 0.071 -0.367 0.006 -0.049

11 6 0.179 -0.171 0.102 -0.016 0.148 -0.03 0.109 -0.1 0 -0.007 0.31 -0.108 0.056 -0.064 -0.151 -0.518 0 0.052 0.072 -0.024 -0.174 0.071 -0.427 0.006 -0.069

11 6 0.179 -0.171 0.194 -0.016 0.148 -0.03 0.109 -0.1 0 0.037 0.404 -0.108 0.056 -0.064 -0.151 -0.626 -0.016 0.103 0.072 -0.024 -0.174 0.071 -0.367 0.006 0.004

11 6 0.179 -0.171 0.102 -0.016 0.191 -0.03 0.109 -0.002 0 -0.007 0.189 -0.108 0.056 -0.05 -0.151 -0.479 -0.016 0.035 0.072 -0.024 -0.164 0.071 -0.367 0.006 0.004

11 6 0.179 -0.204 0.102 -0.016 0.148 -0.03 0.109 -0.1 0 0.037 0.404 -0.108 0.056 -0.064 -0.151 -0.626 -0.016 0.103 0.072 -0.024 -0.174 0.071 -0.367 0.006 0.004

11 7 0.179 -0.171 0.102 -0.016 0.148 -0.03 0.109 -0.1 0 0.037 0.404 -0.108 0.056 -0.064 -0.151 -0.626 -0.016 0.028 0.072 -0.024 -0.174 0.071 -0.367 0.006 0.004

11 6 0.179 -0.171 0.102 -0.016 0.148 -0.03 0.055 -0.1 0 0.037 0.404 -0.108 0.056 -0.064 -0.151 -0.626 -0.016 0.103 0.072 -0.024 -0.174 0.071 -0.367 0.006 0.004

11 5 0.179 -0.162 0.102 -0.016 0.148 -0.03 0.109 -0.1 0 -0.007 0.421 -0.108 0.056 -0.064 -0.151 -0.518 -0.401 0.052 0.072 0.111 -0.174 0.071 -0.427 0.006 0.004

11 4 0.179 -0.162 0.102 -0.016 -0.025 -0.03 0.109 -0.1 0 -0.007 0.31 -0.108 0.056 -0.064 -0.151 -0.605 -0.401 0.052 0.072 0.111 -0.174 0.071 -0.427 0.006 0.004

11 4 0.179 -0.171 0.102 -0.016 0.148 -0.03 0.109 -0.1 0 -0.007 0.31 -0.108 0.056 -0.064 -0.151 -0.518 0 0.052 0.072 -0.024 -0.174 0.071 -0.427 -0.147 0.004

11 4 0.179 -0.171 0.102 -0.016 0.191 -0.03 0.109 -0.1 0 -0.007 0.189 -0.108 0.056 -0.05 -0.151 -0.479 -0.016 0.035 0.072 -0.024 -0.164 0.071 -0.367 0.006 0.004

11 4 0.179 -0.171 0.102 -0.016 0.148 -0.03 0.109 -0.1 0 -0.007 0.31 -0.108 0.056 -0.064 -0.099 -0.518 0 0.052 0.072 -0.024 -0.174 0.071 -0.427 0.006 0.004

11 4 0.179 -0.171 0.102 -0.016 0.148 -0.03 0.109 -0.1 0 -0.007 0.31 -0.1 0.056 -0.064 -0.151 -0.518 0 0.052 0.072 -0.024 -0.297 0.071 -0.427 0.006 0.004

11 4 0.179 -0.171 0.102 -0.016 0.148 -0.03 0.109 -0.1 0 0.133 0.31 -0.108 0.056 -0.064 -0.151 -0.518 0 0.052 0.072 -0.024 -0.174 0.071 -0.427 -0.081 0.004

11 4 0.179 -0.171 0.102 -0.016 0.148 -0.03 0.109 -0.1 0 -0.007 0.189 -0.108 0.056 -0.05 -0.151 -0.505 -0.016 0.035 0.072 -0.024 -0.181 0.071 -0.367 0.22 0.004

11 4 0.179 -0.171 0.102 -0.016 0.148 -0.03 0.109 -0.1 0 0.037 0.404 -0.108 0.029 -0.064 -0.151 -0.626 -0.016 0.103 0.072 -0.024 -0.174 0.071 -0.367 0.006 0.004

11 4 0.179 -0.162 0.102 -0.016 0.148 -0.03 0.109 -0.1 0 0.002 0.31 -0.108 0.056 -0.064 -0.151 -0.605 -0.401 0.052 0.072 0.111 -0.174 0.071 -0.427 0.006 0.004

11 4 0.179 -0.171 0.102 -0.016 0.148 -0.03 0.109 -0.1 0 -0.007 0.31 -0.11 0.056 -0.064 -0.151 -0.518 0 0.052 0.072 -0.024 -0.297 0.071 -0.427 0.006 0.004

11 4 0.179 -0.171 0.102 -0.016 0.117 -0.03 0.109 -0.1 0 0.037 0.404 -0.108 0.056 -0.064 -0.151 -0.626 -0.016 0.103 0.072 -0.024 -0.174 0.071 -0.367 0.006 -0.008

11 4 0.179 -0.171 0.038 -0.016 0.148 -0.03 0.109 -0.1 0 -0.007 0.189 -0.108 0.056 -0.05 -0.151 -0.505 -0.016 0.035 0.072 -0.024 -0.181 0.071 -0.367 -0.02 0.004

11 4 0.179 -0.171 0.056 -0.016 0.148 -0.03 0.001 -0.1 0 -0.007 0.31 -0.108 0.056 -0.064 -0.151 -0.518 0 0.052 0.072 -0.024 -0.297 0.071 -0.427 0.006 0.004

11 4 0.179 -0.171 0.102 -0.016 0.148 -0.03 0.109 -0.1 0 -0.007 0.189 -0.108 0.056 -0.05 -0.151 -0.505 -0.016 0.035 0.072 -0.024 -0.181 0.071 -0.48 0.006 0.004

11 4 0.179 -0.171 0.102 -0.113 0.148 0.01 0.109 -0.1 0 -0.007 0.31 -0.108 0.056 -0.064 -0.151 -0.518 0 0.052 0.072 -0.024 -0.174 0.071 -0.427 0.006 0.004

11 3 0.179 -0.171 0.102 -0.016 0.148 -0.03 0.109 0.017 0 -0.007 0.31 -0.108 0.056 -0.064 -0.151 -0.518 0 0.052 0.072 -0.024 -0.297 0.071 -0.427 0.006 0.004

11 3 0.179 -0.171 0.102 -0.016 0.148 -0.03 0.109 -0.1 0 0.037 0.404 -0.108 0.056 -0.064 -0.151 -0.626 -0.016 0.103 0.072 -0.024 -0.174 0.071 -0.298 0.006 0.004

11 3 0.179 -0.162 0.118 -0.016 0.148 -0.03 0.109 -0.1 0 -0.007 0.31 -0.108 0.056 -0.064 -0.151 -0.518 -0.401 0.052 0.072 0.111 -0.174 0.071 -0.427 0.006 0.004

11 3 0.179 -0.171 0.102 -0.016 0.148 -0.03 0.109 -0.1 0 -0.123 0.189 -0.108 0.056 -0.064 -0.151 -0.518 -0.016 0.035 0.072 -0.024 -0.181 0.071 -0.314 0.006 0.004

11 3 0.179 -0.35 0.102 -0.016 0.148 -0.03 0.109 -0.1 0 0.037 0.404 -0.108 0.056 -0.064 -0.151 -0.626 -0.016 0.103 0.072 -0.024 -0.174 0.071 -0.367 0.006 0.004

11 3 0.179 -0.171 0.102 -0.016 0.148 -0.03 0.109 -0.1 0 -0.007 0.475 -0.108 0.056 -0.064 -0.151 -0.518 0 0.052 0.072 -0.024 -0.174 0.071 -0.427 0.006 0.004

11 3 0.179 -0.171 0.102 -0.016 0.148 -0.03 0.109 -0.1 0 -0.007 0.31 -0.108 0.056 -0.064 -0.151 -0.518 0 0.052 0.072 -0.024 -0.234 0.071 -0.427 0.006 0.004

11 3 0.179 -0.171 0.102 -0.016 0.148 -0.03 0.109 -0.1 0 -0.007 0.31 -0.108 0.056 -0.064 -0.151 -0.518 0 0.052 0.072 -0.024 -0.174 0.071 -0.571 0.006 0.004

11 2 0.179 -0.171 0.102 -0.016 0.148 -0.03 0.132 -0.1 0 -0.007 0.31 -0.108 0.056 -0.064 -0.151 -0.518 0 0.052 0.072 -0.024 -0.174 0.071 -0.427 0.006 0.004

11 2 0.179 -0.171 0.102 -0.016 0.148 -0.03 0.109 -0.1 0 -0.123 0.189 -0.108 0.056 0.053 -0.151 -0.518 -0.016 0.035 0.072 -0.024 -0.181 0.071 -0.367 0.006 0.004

11 2 0.179 -0.171 0.102 -0.016 0.148 -0.03 0.109 -0.1 0 -0.123 0.189 -0.108 0.056 -0.064 -0.151 -0.518 -0.016 0.004 0.072 -0.024 -0.181 0.071 -0.367 0.006 -0.054

11 2 0.179 -0.171 0.102 -0.016 0.148 -0.03 0.109 -0.118 0 0.037 0.404 -0.108 0.056 0.06 -0.151 -0.626 -0.016 0.103 0.072 -0.024 -0.174 0.071 -0.367 0.006 0.004

11 2 0.179 -0.171 0.102 -0.016 0.148 -0.03 0.136 -0.1 0 0.108 0.31 -0.108 0.056 -0.064 -0.151 -0.649 0 0.052 0.072 -0.024 -0.174 0.071 -0.427 0.006 0.004

11 2 0.179 -0.171 0.102 -0.016 0.148 -0.03 0.109 -0.1 0 -0.007 0.189 -0.108 0.056 -0.05 -0.151 -0.505 -0.016 0.048 0.072 -0.024 -0.181 0.071 -0.373 0.006 0.004

11 2 0.179 -0.171 0.102 -0.016 0.148 -0.03 0.109 -0.172 0 -0.007 0.31 -0.108 0.056 -0.064 -0.151 -0.518 0 0.052 0.072 -0.024 -0.174 0.071 -0.427 0.006 0.004

11 2 0.179 -0.288 0.102 -0.016 0.148 -0.03 0.109 -0.1 0 0.037 0.404 -0.108 0.056 -0.064 -0.151 -0.626 -0.016 0.103 0.072 -0.024 -0.174 0.071 -0.367 0.006 0.004

11 2 0.179 -0.171 0.102 -0.016 0.191 -0.03 0.109 -0.1 0 -0.007 0.346 -0.108 0.056 -0.05 -0.151 -0.505 -0.016 0.035 0.072 -0.024 -0.164 0.071 -0.367 0.006 0.004

11 2 0.179 -0.171 0.102 -0.016 0.148 -0.03 0.109 -0.1 0 -0.007 0.31 -0.108 0.056 -0.064 -0.151 -0.518 0 0.052 0.072 -0.024 -0.174 0.071 -0.427 0.025 0.004

11 2 0.179 -0.171 0.102 -0.016 0.148 -0.03 0.099 -0.1 0 -0.007 0.31 -0.108 0.056 -0.064 -0.151 -0.518 0 0.052 0.072 -0.024 -0.174 0.071 -0.427 0.006 0.004

11 2 0.179 -0.171 0.102 -0.016 0.148 -0.03 0.109 -0.1 0 0.037 0.404 -0.108 0.056 -0.064 -0.151 -0.518 -0.016 0.103 0.072 -0.024 -0.174 0.071 -0.367 0.006 -0.02

11 2 0.179 -0.162 0.102 -0.016 0.148 -0.03 0.109 -0.1 0 -0.007 0.31 -0.108 0.056 -0.064 -0.151 -0.605 -0.401 0.052 0.072 0.111 -0.174 0.071 -0.427 0.092 0.004

11 2 0.179 -0.171 0.102 -0.016 0.148 -0.03 0.109 -0.1 0 -0.007 0.31 -0.104 0.056 -0.064 -0.151 -0.518 -0.139 0.052 0.072 -0.024 -0.174 0.071 -0.427 0.006 0.004

11 2 0.179 -0.171 0.102 -0.016 0.148 -0.03 0.109 -0.1 0 -0.007 0.31 -0.108 0.056 -0.064 -0.151 -0.518 -0.139 0.052 0.072 -0.025 -0.174 0.071 -0.427 0.006 0.004

11 2 0.179 -0.162 0.102 -0.016 0.148 -0.03 0.109 -0.1 0 -0.007 0.31 -0.108 0.056 -0.064 -0.151 -0.605 -0.401 0.052 0.072 -0.025 -0.174 0.071 -0.427 0.006 0.004

11 2 0.179 -0.171 0.102 -0.016 0.148 -0.03 0.109 -0.1 0 0.108 0.31 -0.108 0.056 -0.064 -0.151 -0.518 0 0.052 0.12 -0.024 -0.174 0.071 -0.427 0.006 0.004

11 2 0.179 -0.171 0.102 -0.016 0.148 -0.03 0.109 -0.1 0 0.037 0.404 -0.108 0.056 -0.165 -0.151 -0.518 -0.016 0.103 0.072 -0.024 -0.174 0.071 -0.367 0.006 -0.02

11 2 0.179 -0.171 0.102 -0.016 0.148 -0.03 0.109 -0.1 0 0.037 0.404 -0.108 0.056 -0.064 -0.151 -0.518 -0.016 0.059 0.072 -0.024 -0.174 0.071 -0.367 0.006 0.004

11 2 0.179 -0.171 0.102 -0.016 0.148 -0.03 0.109 -0.1 0 -0.123 0.189 -0.108 0.056 -0.061 -0.151 -0.518 -0.016 0.035 0.072 -0.024 -0.181 0.071 -0.367 0.006 0.004

11 2 0.179 -0.171 0.102 -0.016 0.148 -0.03 0.109 -0.1 0 -0.007 0.31 -0.108 0.056 -0.064 -0.151 -0.518 0 0.052 0.072 -0.024 -0.174 0.071 -0.427 0.027 0.004

11 2 0.179 -0.173 0.102 -0.016 0.148 -0.03 0.109 -0.1 0 -0.007 0.31 -0.108 0.056 -0.064 -0.151 -0.59 0 0.052 0.072 -0.024 -0.174 0.071 -0.427 0.006 0.004

11 2 0.179 -0.171 0.102 -0.016 0.148 -0.03 0.109 -0.1 0 0.108 0.31 -0.108 0.056 -0.064 -0.151 -0.518 0 0.052 0.072 -0.024 -0.174 0.071 -0.427 0.006 0.023

11 1 0.179 -0.171 0.102 -0.016 0.191 -0.03 0.109 -0.1 -0.106 -0.007 0.189 -0.108 0.056 -0.05 -0.151 -0.505 -0.016 0.035 0.072 -0.024 -0.164 0.071 -0.367 0.006 0.004

11 1 0.179 -0.171 0.102 -0.016 0.148 -0.03 0.109 -0.1 0 -0.007 0.31 -0.108 0.056 -0.064 -0.151 -0.518 -0.139 0.052 0.072 -0.024 -0.174 0.071 -0.362 0.006 0.004

11 1 0.179 -0.171 0.078 -0.016 0.148 -0.03 0.109 -0.1 0 -0.007 0.31 -0.108 0.056 -0.064 -0.151 -0.518 -0.139 0.052 0.072 -0.024 -0.174 0.071 -0.427 0.006 0.004

11 1 0.179 -0.171 0.102 -0.016 0.148 -0.03 0.109 -0.1 0 -0.123 0.189 -0.108 0.056 -0.064 -0.151 -0.459 -0.016 0.035 0.072 -0.024 -0.181 0.071 -0.367 0.006 0.004

11 1 0.208 -0.171 0.102 -0.016 0.148 -0.03 0.109 -0.1 0 0.037 0.404 -0.108 0.056 -0.064 -0.151 -0.626 -0.016 0.103 0.072 -0.024 -0.174 0.071 -0.367 0.006 0.004

11 1 0.179 -0.171 0.102 -0.016 0.148 -0.03 0.109 -0.1 0 0.037 0.404 -0.108 0.056 -0.064 -0.151 -0.626 -0.016 -0.047 0.072 -0.024 -0.174 0.071 -0.367 0.006 -0.049

11 1 0.179 -0.171 0.102 -0.016 0.148 -0.03 0.109 -0.1 0 0.037 0.404 -0.108 0.056 -0.064 -0.151 -0.626 -0.016 0.103 0.072 -0.094 -0.174 0.071 -0.367 0.006 -0.049

11 1 0.179 -0.171 0.102 0.023 0.148 -0.03 0.109 -0.1 0 -0.123 0.189 -0.108 0.056 -0.064 -0.151 -0.518 -0.016 0.035 0.072 -0.024 -0.181 0.071 -0.367 0.006 0.004

11 1 0.179 -0.171 0.102 -0.016 0.148 -0.03 0.109 -0.1 -0.082 -0.123 0.189 -0.108 0.056 -0.064 -0.151 -0.518 -0.016 0.035 0.072 -0.024 -0.181 0.071 -0.367 0.006 0.004

11 1 0.179 -0.171 0.102 -0.016 0.148 -0.03 0.109 -0.1 0 0.037 0.404 -0.108 0.056 -0.064 -0.151 -0.626 -0.016 0.122 0.072 -0.024 -0.174 0.071 -0.367 0.006 0.004

11 1 0.179 -0.171 0.038 -0.016 0.148 -0.03 0.109 -0.1 0 -0.007 0.189 -0.108 0.056 -0.05 -0.151 -0.475 -0.016 0.035 0.072 -0.024 -0.181 0.071 -0.367 0.006 0.004

11 1 0.179 -0.171 0.102 -0.016 0.148 -0.03 0.109 -0.1 0 -0.123 0.189 -0.108 0.056 -0.064 -0.151 -0.518 -0.031 0.035 0.072 -0.024 -0.181 0.071 -0.367 0.006 0.004

11 1 0.179 -0.171 -0.057 -0.016 0.148 -0.03 0.109 -0.1 0 -0.123 0.189 -0.108 0.056 -0.064 -0.151 -0.518 -0.016 0.035 0.072 -0.024 -0.181 0.071 -0.367 0.006 0.004

11 1 0.179 -0.171 0.102 -0.016 0.148 -0.03 0.109 -0.1 0 -0.007 0.31 -0.108 0.056 -0.064 -0.151 -0.518 0 0.052 0.072 -0.024 -0.174 0.071 -0.427 0.179 0.004

11 1 0.179 -0.162 0.102 -0.016 0.148 -0.03 0.109 -0.1 0 -0.007 0.31 -0.108 0.056 0.005 -0.151 -0.518 -0.401 0.052 0.072 0.111 -0.174 0.071 -0.427 0.006 0.004

11 1 0.179 -0.171 0.102 -0.016 0.148 -0.03 0.109 -0.1 0 0.133 0.31 -0.108 0.056 -0.064 -0.151 -0.518 0 0.052 0.056 -0.024 -0.174 0.071 -0.427 0.006 0.004

11 1 0.179 -0.171 0.102 -0.016 0.148 -0.03 0.109 -0.1 0 0.037 0.404 -0.108 0.122 -0.064 -0.151 -0.626 -0.016 0.103 0.072 -0.024 -0.174 0.071 -0.367 0.006 0.004

11 1 0.179 -0.171 0.102 -0.016 0.148 -0.03 0.109 -0.1 0 -0.123 0.189 -0.108 0.056 -0.064 -0.097 -0.518 -0.016 0.035 0.072 -0.024 -0.181 0.071 -0.367 0.006 0.004

11 1 0.179 -0.171 0.102 -0.016 0.148 -0.03 0.109 -0.1 0 -0.123 0.189 -0.108 0.056 -0.064 -0.151 -0.518 -0.016 0.035 0.178 -0.024 -0.181 0.071 -0.367 0.006 0.004

11 1 0.179 -0.345 0.038 -0.016 0.148 -0.03 0.109 -0.1 0 -0.007 0.189 -0.108 0.056 -0.05 -0.151 -0.505 -0.016 0.035 0.072 -0.024 -0.181 0.071 -0.367 0.006 0.004

11 1 0.179 -0.171 0.102 -0.016 0.117 -0.033 0.109 -0.1 0 0.037 0.404 -0.108 0.056 -0.064 -0.151 -0.626 -0.016 0.103 0.072 -0.024 -0.174 0.071 -0.367 0.006 0.004

11 1 0.179 -0.171 0.102 -0.016 0.087 -0.03 0.109 -0.1 0 -0.007 0.189 -0.108 0.056 -0.05 -0.151 -0.505 -0.016 0.035 0.072 -0.024 -0.181 0.071 -0.367 0.006 0.004

11 1 0.179 -0.171 0.145 -0.016 0.148 -0.03 0.109 -0.1 0 -0.007 0.189 -0.108 0.056 -0.05 -0.151 -0.505 -0.016 0.035 0.072 -0.024 -0.181 0.071 -0.367 0.006 0.004

11 1 0.179 -0.171 0.102 -0.016 0.148 -0.03 0.109 -0.1 0 0.037 0.404 -0.108 0.056 0.013 -0.151 -0.626 -0.016 0.103 0.072 -0.024 -0.174 0.071 -0.367 0.006 0.004

11 1 0.179 -0.171 0.102 -0.016 0.148 -0.03 0.109 -0.1 0 -0.007 0.38 -0.108 0.056 -0.064 -0.151 -0.518 0 0.052 0.072 -0.024 -0.174 0.071 -0.427 0.006 0.004

11 1 0.179 -0.171 0.102 -0.016 0.191 -0.03 0.109 -0.1 0 -0.007 0.189 -0.108 0.056 -0.05 -0.151 -0.505 -0.016 0.035 0.072 0.123 -0.164 0.071 -0.367 0.006 0.004

11 1 0.179 -0.171 0.102 -0.016 0.148 -0.03 0.109 -0.1 0 -0.007 0.182 -0.108 0.056 -0.05 -0.151 -0.505 -0.016 0.035 0.072 -0.024 -0.181 0.071 -0.373 0.006 0.004

11 1 0.179 -0.162 0.102 -0.016 0.148 -0.03 0.109 -0.1 0 -0.007 0.28 -0.108 0.056 -0.064 -0.151 -0.605 -0.401 0.052 0.072 0.111 -0.174 0.071 -0.427 0.006 0.004

11 1 0.179 -0.171 0.102 -0.016 0.148 -0.03 0.109 -0.1 0 -0.007 0.189 -0.108 0.03 -0.05 -0.151 -0.505 -0.016 0.035 0.072 -0.024 -0.181 0.071 -0.367 0.006 0.004

11 1 0.179 -0.171 0.102 -0.016 0.148 -0.03 0.109 -0.1 0 0.037 0.404 -0.108 0.056 -0.064 -0.151 -0.626 -0.016 0.103 0.214 -0.024 -0.174 0.071 -0.367 0.006 0.004

11 1 0.179 -0.171 0.102 -0.016 0.148 -0.03 0.109 -0.1 -0.078 0.037 0.404 -0.108 0.056 -0.064 -0.151 -0.626 -0.016 0.103 0.072 -0.024 -0.174 0.071 -0.367 0.006 0.004

11 1 0.179 -0.171 0.102 -0.016 0.148 -0.03 0.109 -0.1 0 -0.007 0.31 -0.108 0.056 -0.064 -0.151 -0.518 -0.139 0.052 0.04 -0.024 -0.174 0.071 -0.427 0.006 0.004

11 1 0.179 -0.171 0.102 -0.016 0.11 -0.03 0.109 -0.1 0 -0.007 0.31 -0.108 0.056 -0.064 -0.151 -0.518 -0.061 0.052 0.072 -0.024 -0.174 0.071 -0.427 0.006 0.004

11 1 0.179 -0.171 0.102 -0.016 0.148 -0.03 0.288 -0.1 0 -0.123 0.189 -0.108 0.056 -0.064 -0.151 -0.518 -0.016 0.035 0.072 -0.024 -0.181 0.071 -0.367 0.006 0.004

11 1 0.179 -0.171 0.102 -0.016 0.191 -0.03 0.109 -0.1 0 -0.007 0.189 -0.108 0.056 -0.05 -0.322 -0.505 -0.016 0.035 0.072 -0.024 -0.164 0.071 -0.367 0.006 0.004

11 1 0.179 -0.171 0.102 -0.016 0.148 -0.03 0.109 -0.1 0 0.108 0.31 -0.108 0.013 -0.064 -0.151 -0.518 0 0.052 0.072 -0.024 -0.174 0.071 -0.427 0.006 0.004

11 1 0.179 -0.171 0.102 -0.016 0.148 -0.03 0.109 -0.1 0 -0.007 0.31 -0.108 0.056 -0.064 -0.151 -0.318 0 0.052 0.072 -0.024 -0.297 0.071 -0.427 0.006 0.004

11 1 0.179 -0.171 0.102 -0.016 0.148 -0.03 0.109 -0.1 0 0.037 0.404 -0.108 0.056 -0.064 -0.151 -0.626 -0.016 0.103 0.072 -0.024 -0.174 0.071 -0.354 0.006 0.004

11 1 0.179 -0.171 0.102 -0.016 0.148 -0.03 0.109 -0.1 0 0.037 0.404 -0.108 0.056 -0.064 -0.151 -0.626 -0.016 0.103 0.072 -0.087 -0.174 0.071 -0.367 0.006 0.004

11 1 0.179 -0.171 0.102 -0.016 0.148 -0.03 0.109 -0.1 0 0.108 0.31 -0.108 0.056 -0.064 -0.151 -0.518 0 0.052 0.148 -0.024 -0.174 0.071 -0.427 0.006 0.004

11 1 0.179 -0.171 0.102 -0.016 0.148 -0.03 0.109 -0.1 0 -0.007 0.31 -0.108 0.056 -0.064 -0.151 -0.518 0 0.052 0.072 -0.024 -0.368 0.071 -0.427 0.006 0.004

11 1 0.179 -0.171 0.102 -0.016 0.117 -0.03 0.109 -0.1 0 0.037 0.404 -0.108 0.128 -0.064 -0.151 -0.626 -0.016 0.103 0.072 -0.024 -0.174 0.071 -0.367 0.006 0.004

11 1 0.179 -0.171 0.093 -0.016 0.148 -0.03 0.109 -0.1 0 0.108 0.31 -0.108 0.056 -0.095 -0.151 -0.518 0 0.052 0.072 -0.024 -0.174 0.071 -0.427 0.006 0.004

11 1 0.179 -0.171 0.102 -0.016 0.148 -0.03 0.109 -0.1 0 0.037 0.404 -0.108 0.056 -0.064 -0.151 -0.626 0.016 0.103 0.072 -0.024 -0.174 0.071 -0.367 0.006 0.004

11 1 0.179 -0.171 0.102 -0.016 0.148 0.01 0.109 -0.1 -0.136 -0.007 0.31 -0.108 0.056 -0.064 -0.151 -0.518 0 0.052 0.072 -0.024 -0.174 0.071 -0.427 0.006 0.004

11 1 0.179 -0.171 0.102 -0.016 0.148 -0.03 0.109 -0.1 0 0.108 0.31 -0.108 0.056 -0.052 -0.151 -0.518 0 0.052 0.072 -0.024 -0.174 0.071 -0.427 0.006 0.004

11 1 0.179 -0.171 0.102 -0.016 0.148 -0.03 0.109 -0.1 0 -0.007 0.189 -0.108 0.056 -0.05 -0.151 -0.505 -0.016 0.035 0.072 -0.024 -0.236 0.071 -0.367 0.006 0.004

11 1 0.179 -0.171 0.102 -0.016 0.148 -0.03 0.109 -0.1 0 -0.123 0.092 -0.108 0.056 -0.064 -0.151 -0.518 -0.016 0.035 0.072 -0.024 -0.181 0.071 -0.367 0.006 0.004

11 1 0.179 -0.162 0.102 -0.016 0.148 -0.03 0.156 -0.1 0 -0.007 0.31 -0.108 0.056 -0.064 -0.151 -0.605 -0.401 0.052 0.072 0.111 -0.174 0.071 -0.427 0.006 0.004

11 1 0.179 -0.171 0.102 -0.016 0.148 -0.03 0.109 -0.1 0 0.108 0.31 -0.108 0.056 -0.064 -0.151 -0.518 0 0.052 0.072 -0.024 -0.174 0.071 -0.427 0.006 -0.104

11 1 0.179 -0.171 0.102 -0.016 0.148 -0.03 0.109 -0.1 0 0.037 0.404 -0.108 0.056 -0.064 -0.151 -0.626 -0.016 0.103 0.072 -0.024 -0.174 0.089 -0.367 0.006 0.004

11 1 0.179 -0.171 0.102 -0.016 0.117 -0.03 0.109 -0.1 0 0.037 0.404 -0.108 0.056 -0.064 -0.151 -0.626 -0.016 0.103 -0.032 -0.024 -0.174 0.071 -0.367 0.006 0.004

11 1 0.179 -0.171 0.102 -0.016 0.148 -0.03 0.109 -0.1 0 0 0.189 -0.108 0.056 -0.14 -0.151 -0.505 -0.016 0.035 0.072 -0.024 -0.181 0.071 -0.373 0.006 0.004

11 1 0.179 -0.171 0.102 -0.016 0.148 -0.03 0.109 -0.1 0 0.037 0.404 -0.108 0.056 -0.064 -0.151 -0.626 -0.016 0.103 0.047 -0.024 -0.174 0.071 -0.367 0.006 0.004

11 1 0.179 -0.171 0.102 -0.158 0.148 -0.03 0.109 -0.1 0 0.037 0.404 -0.108 0.056 -0.064 -0.151 -0.626 -0.016 0.103 0.072 -0.024 -0.174 0.071 -0.367 0.006 0.004

11 1 0.179 -0.171 0.102 -0.016 0.148 -0.03 0.109 -0.1 0 0.037 0.404 -0.108 0.056 -0.064 -0.151 -0.626 -0.016 0.103 0.041 -0.024 -0.174 0.071 -0.367 0.006 0.004

11 1 0.179 -0.171 0.102 -0.016 0.148 -0.03 0.109 -0.1 0 0.037 0.404 -0.108 0.056 -0.064 -0.151 -0.626 -0.016 0.103 0.072 -0.024 -0.174 0.071 -0.367 0.006 0.068

11 1 0.179 -0.171 0.102 -0.016 0.148 -0.03 0.109 -0.1 0 0.07 0.31 -0.108 0.056 -0.064 -0.151 -0.554 -0.139 0.052 0.072 -0.024 -0.174 0.071 -0.427 0.006 0.004

11 1 0.179 -0.171 0.102 -0.016 0.148 -0.03 0.109 -0.252 0 -0.007 0.31 -0.108 0.056 -0.064 -0.151 -0.518 0 0.052 0.072 -0.024 -0.174 0.071 -0.427 0.006 0.004

11 1 0.179 -0.171 0.102 -0.016 0.148 -0.03 0.109 -0.1 0 -0.007 0.31 -0.108 0.056 -0.064 -0.151 -0.518 0 -0.017 0.072 -0.024 -0.174 0.071 -0.427 0.006 0.004

11 1 0.179 -0.171 0.102 -0.016 0.148 -0.03 0.109 -0.1 0 0.037 0.404 -0.108 0.056 -0.064 -0.151 -0.626 -0.016 0.103 0.072 -0.073 -0.174 0.071 -0.367 0.006 0.004

11 1 0.179 -0.171 0.102 -0.016 0.148 -0.03 0.109 -0.1 0 -0.007 0.31 -0.108 0.056 -0.064 -0.151 -0.518 0 0.052 0.072 -0.024 -0.082 0.071 -0.427 0.006 0.004

11 1 0.179 -0.171 0.102 -0.016 0.148 0.017 0.109 -0.1 0 -0.007 0.31 -0.108 0.056 -0.064 -0.151 -0.518 0 0.052 0.072 -0.024 -0.297 0.071 -0.427 0.006 0.004

11 1 0.179 -0.171 0.102 -0.016 0.148 -0.03 0.109 -0.1 0 -0.007 0.31 -0.108 0.056 -0.064 -0.171 -0.518 0 0.052 0.072 -0.024 -0.174 0.071 -0.427 0.006 0.004

11 1 0.179 -0.211 0.102 -0.016 0.148 -0.03 0.109 -0.1 0 0.037 0.404 -0.108 0.056 -0.064 -0.151 -0.626 -0.016 0.103 0.072 -0.024 -0.174 0.071 -0.367 0.006 0.004

11 1 0.179 -0.171 0.102 -0.016 0.148 -0.03 0.109 -0.1 0 -0.123 0.189 -0.108 0.056 -0.064 -0.151 -0.518 -0.016 0.035 0.02 -0.024 -0.181 0.071 -0.367 0.006 0.004

11 1 0.179 -0.171 0.102 -0.016 0.148 -0.03 0.109 -0.1 0 -0.007 0.31 -0.108 0.056 -0.064 -0.151 -0.518 -0.139 0.052 0.072 -0.024 -0.289 0.071 -0.427 0.006 0.004

11 1 0.179 -0.171 0.102 -0.016 0.148 -0.03 0.109 -0.1 0 -0.007 0.303 -0.108 0.056 -0.064 -0.151 -0.518 0 0.052 0.072 -0.024 -0.297 0.071 -0.427 0.006 0.004

11 1 0.179 -0.171 0.102 -0.016 0.148 -0.03 0.109 -0.1 0 -0.007 0.31 -0.108 0.056 -0.064 -0.151 -0.518 0 0.052 0.072 -0.024 -0.174 0.071 -0.427 0.094 0.004

11 1 0.179 -0.171 0.102 -0.016 0.148 -0.03 0.109 -0.1 0 0.123 0.31 -0.108 0.056 -0.064 -0.151 -0.518 -0.139 0.052 0.072 -0.024 -0.174 0.071 -0.427 0.006 0.004

11 1 0.179 -0.171 0.102 -0.016 0.148 -0.03 0.109 -0.1 0 -0.007 0.31 -0.108 0.056 -0.064 -0.151 -0.518 -0.139 0.052 0.072 -0.024 -0.164 0.071 -0.427 0.006 0.004

11 1 0.179 -0.162 0.102 -0.016 0.148 -0.03 0.109 -0.1 0 -0.007 0.31 -0.108 0.056 -0.064 -0.151 -0.605 -0.482 0.052 0.072 0.111 -0.174 0.071 -0.427 0.006 0.004

11 1 0.179 -0.171 0.102 -0.016 0.148 -0.03 0.109 -0.133 0 0.037 0.404 -0.108 0.056 -0.064 -0.151 -0.626 -0.016 0.103 0.072 -0.024 -0.174 0.071 -0.367 0.006 0.004

11 1 0.179 -0.171 0.102 -0.016 0.148 -0.03 0.109 -0.1 0 -0.007 0.31 -0.108 0.056 -0.064 -0.151 -0.518 -0.139 0.052 0.072 -0.024 -0.174 0.047 -0.427 0.006 0.004

11 1 0.179 -0.171 0.102 -0.016 0.148 -0.03 0.109 -0.186 0 -0.007 0.31 -0.108 0.056 -0.064 -0.151 -0.518 0 0.052 0.072 -0.024 -0.174 0.071 -0.427 0.006 0.004

11 1 0.179 -0.171 0.102 -0.016 0.127 0.01 0.109 -0.1 0 -0.007 0.31 -0.108 0.056 -0.064 -0.151 -0.518 0 0.052 0.072 -0.024 -0.174 0.071 -0.427 0.006 0.004

11 1 0.179 -0.171 0.102 -0.016 0.148 -0.107 0.109 -0.1 0 0.037 0.404 -0.108 0.056 -0.064 -0.151 -0.626 -0.016 0.103 0.072 -0.024 -0.174 0.071 -0.367 0.006 0.004

11 1 0.179 -0.171 0.102 -0.016 0.045 -0.03 0.109 -0.1 0 0.037 0.404 -0.108 0.056 -0.064 -0.151 -0.626 -0.016 0.103 0.072 -0.024 -0.174 0.071 -0.367 0.006 0.004

11 1 0.179 -0.171 0.102 -0.016 0.148 -0.03 0.109 -0.1 0 0.037 0.404 -0.108 0.056 -0.064 -0.151 -0.626 -0.016 0.103 0.072 -0.024 -0.13 0.071 -0.367 0.006 0.004

11 1 0.179 -0.171 0.056 -0.016 0.148 -0.03 0.16 -0.1 0 -0.007 0.31 -0.108 0.056 -0.064 -0.151 -0.518 0 0.052 0.072 -0.024 -0.297 0.071 -0.427 0.006 0.004

11 1 0.179 -0.171 0.056 -0.016 0.057 -0.03 0.109 -0.1 0 -0.007 0.31 -0.108 0.056 -0.064 -0.151 -0.518 0 0.052 0.072 -0.024 -0.297 0.071 -0.427 0.006 0.004

11 1 0.179 -0.171 0.102 -0.016 0.148 -0.03 0.109 -0.1 0 0.037 0.404 -0.108 0.064 0.022 -0.151 -0.626 -0.016 0.103 0.072 -0.024 -0.174 0.071 -0.367 0.006 0.004

11 1 0.179 -0.171 0.102 -0.016 0.148 -0.03 0.109 -0.1 0 0.108 0.31 -0.108 0.056 -0.064 -0.151 -0.518 0 0.052 0.189 -0.024 -0.174 0.071 -0.427 0.006 0.004

11 1 0.179 -0.171 0.102 -0.016 0.148 -0.03 0.109 -0.1 0 -0.007 0.31 -0.108 0.056 -0.064 -0.151 -0.518 0 0.052 0.072 -0.024 -0.095 0.071 -0.427 0.006 0.004

11 1 0.179 -0.171 0.102 -0.016 0.148 -0.03 0.109 -0.1 0 -0.007 0.31 -0.13 0.056 -0.064 -0.151 -0.518 0 0.052 0.072 -0.024 -0.174 0.071 -0.427 0.006 0.004

12 340 0 0 0.087 0.033 0.003 -0.076 -0.228 -0.047 0 0 -0.075 0.15 0 -0.019 -0.226 0.155 -0.051 0.041 -0.068 0 -0.086 0 -0.195 0 -0.056

12 598 0 0 0.087 0.033 0.003 -0.076 -0.228 -0.047 0 0 -0.075 0 0 -0.019 -0.226 0.155 -0.051 0.041 -0.068 0 -0.086 0 -0.195 0 -0.056

12 326 0 0 0.087 0.033 0.003 -0.076 -0.228 -0.047 0 0 -0.075 0.15 0 -0.019 -0.226 0.155 -0.051 0.041 -0.068 0 -0.086 0 -0.236 0 -0.056

12 281 0 0 0.087 0.033 0.003 -0.076 -0.228 -0.047 0 0 -0.075 0 0 -0.019 -0.226 0.189 -0.051 0.041 -0.068 0 -0.086 0 -0.195 0 -0.056

12 129 0 0 0.087 0.033 0.003 -0.076 -0.228 -0.047 0 0 -0.075 0 0.074 -0.019 -0.226 0.083 -0.051 0.041 -0.068 0 -0.086 0 -0.195 0 -0.056

12 154 0 0 0.087 0.033 0.003 -0.081 -0.228 -0.047 0 0 -0.075 0 0 -0.019 -0.226 0.155 -0.051 0.041 -0.068 0 -0.086 0 -0.195 0 -0.056

12 66 0 0 0.087 0.033 0.003 -0.076 -0.228 -0.047 0 0 -0.075 0 0 -0.019 -0.226 0.155 -0.051 0.041 -0.068 0 -0.08 0 -0.195 0 -0.056

12 99 0 0 0.087 0.033 0.033 -0.076 -0.228 -0.047 0 0 -0.075 0 0.074 -0.084 -0.226 0.083 -0.051 0.041 -0.068 0 -0.086 0 -0.195 0 -0.056

12 63 0 0 0.087 0.033 0.098 -0.081 -0.228 -0.047 0 -0.059 0 0 0 -0.019 -0.226 0.155 -0.051 0.041 -0.068 0 -0.086 0 -0.195 0 -0.056

12 51 0 0 0.087 0.033 0.033 -0.076 -0.228 -0.047 0 0 -0.075 0 0.074 -0.019 -0.226 0.083 -0.051 0.041 -0.068 0 -0.086 0 -0.195 0 -0.056

12 49 0 0 0.087 0.033 0.003 -0.076 -0.228 -0.047 0 0 -0.075 0 0 -0.019 -0.226 0.083 -0.051 0.041 -0.068 0 -0.086 0 -0.195 0 -0.056

12 42 0 0 0.087 0.033 0.003 -0.076 -0.224 -0.047 0 0 -0.075 0.15 0 -0.019 -0.226 0.155 -0.051 0.041 -0.068 0 -0.086 0 -0.236 0 -0.056

12 39 0 0 0.087 0.033 0.003 -0.076 -0.228 -0.047 0 0 -0.075 0.15 0 -0.019 -0.226 0.155 -0.051 0.041 -0.068 0 -0.086 0 -0.195 0.032 -0.056

12 34 0 0 0.087 0.033 0.003 -0.076 -0.228 -0.047 0.035 0 -0.075 0.15 0 -0.019 -0.226 0.155 -0.051 0.041 -0.068 0 -0.086 0 -0.236 0 -0.056

12 28 0 0 0.087 0.033 0.003 -0.076 -0.228 -0.047 0 0 -0.075 0.15 0 -0.019 -0.226 0.155 -0.051 0.041 -0.068 0 -0.086 0 -0.236 -0.227 -0.056

12 27 0 0 0.087 0.033 0.003 -0.076 -0.228 -0.047 0 0 -0.075 0 0 -0.019 -0.226 0.155 -0.051 0.041 -0.068 0 -0.086 0 -0.195 -0.198 -0.056

12 17 0 0 0.165 0.033 0.003 -0.076 -0.228 -0.047 0 0 -0.075 0.15 0 -0.019 -0.226 0.155 -0.051 0.041 -0.068 0 -0.086 0 -0.195 0.032 -0.056

12 16 0 -0.093 0.087 0.033 0.003 -0.076 -0.228 -0.047 0 0 -0.075 0.15 0 -0.019 -0.226 0.155 -0.051 0.041 -0.068 0 -0.086 0 -0.236 0 -0.056

12 16 0 0 0.087 0.033 0.003 -0.076 -0.228 -0.047 0 0 -0.075 0.15 0 -0.019 -0.226 0.155 -0.051 -0.159 -0.068 0 -0.086 0 -0.236 0 -0.056

12 16 0 0 0.087 0.033 0.003 -0.076 -0.228 -0.047 0 0 -0.075 0 0 -0.019 -0.226 0.155 -0.051 0.041 -0.068 0 -0.086 0 -0.085 0 -0.056

12 15 0 0 0.087 0.033 0.003 -0.076 -0.228 -0.047 0 0 -0.075 0 0 -0.019 -0.226 0.155 -0.051 0.041 -0.068 0 -0.086 -0.077 -0.195 0 -0.056

12 11 0 0 0.087 0.157 0.003 -0.076 -0.228 -0.047 0 0 -0.075 0 0 -0.019 -0.226 0.155 -0.051 0.041 -0.068 0 -0.086 0 -0.085 0 -0.056

12 10 0 0 0.087 0.033 0.003 -0.076 -0.228 -0.047 0 0 -0.075 0 0 -0.019 -0.226 0.155 -0.051 0.041 -0.068 0 -0.086 0.147 -0.195 0 -0.056

12 10 0 0 0.087 0.033 0.033 0.07 -0.228 -0.047 0 0 -0.075 0 0.074 -0.019 -0.226 0.083 -0.051 0.041 -0.068 0 -0.086 0 -0.195 0 -0.056

12 12 0 0 0.087 0.033 0.003 -0.076 -0.228 -0.047 0 -0.094 -0.075 0 0.074 -0.019 -0.226 0.083 -0.051 0.041 -0.068 0 -0.086 0 -0.195 0 -0.056

12 8 0 0 0.087 0.033 0.033 -0.076 -0.228 -0.047 0 0 -0.075 0 0.074 -0.019 -0.226 0.083 -0.051 0.078 -0.068 0 -0.086 0 -0.195 0 -0.056

12 8 0 0 0.087 0.033 0.003 -0.076 -0.098 -0.047 0 0 -0.075 0 0.074 -0.019 -0.226 0.083 -0.051 0.041 -0.068 0 -0.086 0 -0.195 0 -0.056

12 8 0 0 0.087 0.033 0.003 -0.076 -0.228 -0.047 0 0 -0.075 0.15 0.161 -0.019 -0.226 0.155 -0.051 0.041 -0.068 0 -0.086 0 -0.195 0 -0.056

12 7 0 0 0.087 0.033 0.003 -0.076 -0.228 -0.047 0 0 -0.075 0 0 -0.019 -0.226 0.311 -0.051 0.041 -0.068 0 -0.086 0 -0.195 -0.198 -0.056

12 7 0 0 0.087 0.033 0.003 -0.076 -0.228 -0.047 0 0 -0.075 0 -0.012 -0.019 -0.226 0.083 -0.051 0.041 -0.068 0 -0.086 0 -0.195 0 -0.056

12 7 0 0 0.087 0.033 0.003 -0.076 -0.228 -0.047 0 0 -0.01 0 0 -0.019 -0.226 0.155 -0.051 0.041 -0.068 0 -0.086 0 -0.195 0 -0.056

12 6 0 0 0.087 0.033 0.003 -0.192 -0.228 -0.047 0 0 0 0 0 -0.019 -0.226 0.155 -0.051 0.041 -0.068 0 -0.086 0 -0.195 0 -0.056

12 6 0 0 0.087 0.033 0.003 -0.076 -0.228 -0.047 0 0 -0.075 0 0 -0.019 -0.226 0.155 -0.01 0.041 -0.068 0 -0.08 0 -0.195 0 -0.056

12 6 0 0 0.087 0.033 0.003 -0.076 -0.228 -0.047 0 0 -0.075 0.15 0.025 -0.019 -0.226 0.155 -0.051 0.041 -0.068 0 -0.086 0 -0.195 0 -0.056

12 6 0 0 0.087 0.033 0.003 -0.076 -0.228 -0.047 0 0 -0.075 0 0 -0.019 -0.226 0.155 -0.11 0.041 -0.068 0 -0.086 0 -0.195 0 -0.056

12 6 0 0 0.087 0.033 0.003 -0.076 -0.228 -0.047 0 0 -0.075 0 0 -0.019 0.016 0.155 -0.051 0.041 -0.068 0 -0.086 0 -0.195 0 -0.056

12 5 0 0 0.122 0.033 0.003 -0.076 -0.228 -0.047 0 0 -0.075 0 0 -0.019 -0.226 0.083 -0.051 0.041 -0.068 0 -0.086 0 -0.195 0 -0.056

12 5 0 0 0.087 0.033 0.003 -0.076 -0.228 -0.152 0 0 -0.075 0.15 0 -0.019 -0.226 0.155 -0.051 0.041 -0.068 0 -0.086 0 -0.236 0 -0.056

12 5 0 0 0.087 0.033 0.003 -0.081 -0.228 -0.047 0 0 -0.075 0 0.056 -0.019 -0.226 0.155 -0.051 0.041 -0.068 0 -0.086 0 -0.195 0 -0.056

12 4 0 0 0.087 0.033 0.033 -0.076 -0.228 -0.047 0 0 -0.075 0 0.074 -0.019 -0.213 0.083 -0.051 0.041 -0.068 0 -0.086 0 -0.195 0 -0.056

12 4 0 0 0.087 0.033 0.003 -0.076 -0.333 -0.047 0 -0.037 -0.075 0.15 0 -0.019 -0.199 0.155 -0.051 0.041 -0.068 0 -0.086 0 -0.236 0 -0.056

12 4 0 -0.093 0.087 0.033 0.033 -0.076 -0.228 -0.047 0 0 -0.075 0 0.074 -0.019 -0.226 0.083 -0.051 0.041 -0.068 0 -0.086 0 -0.195 0 -0.056

12 4 0 0 0.087 0.033 0.003 -0.076 -0.228 -0.047 0.002 0 -0.075 0.15 0 -0.019 -0.226 0.155 -0.051 0.041 -0.068 0 -0.086 0 -0.236 0 -0.056

12 4 0 0 0.087 0.033 0.003 -0.076 -0.228 -0.047 0 0 -0.075 0 0 -0.019 -0.226 0.145 -0.051 0.041 -0.068 0 -0.086 0 -0.195 0 -0.056

12 4 0 0 0.087 0.033 0.003 -0.076 -0.228 -0.047 0 0 -0.075 0 0 -0.019 -0.226 0.155 -0.051 0.041 -0.068 0 -0.086 0 -0.195 -0.04 -0.056

12 4 0 0 0.087 0.033 0.003 -0.076 -0.228 -0.047 0 0 -0.075 0 0 -0.019 -0.226 0.155 -0.051 0.041 -0.068 0 -0.086 0 -0.195 -0.198 0.011

12 3 0 0 0.087 0.033 0.003 -0.076 -0.228 -0.047 0 0 -0.075 0 0 -0.019 -0.226 0.155 -0.051 0.041 -0.05 0 -0.086 0 -0.195 0 -0.056

12 3 0 0 0.122 0.033 0.003 -0.076 -0.228 -0.047 0 0 -0.075 0 0 -0.019 -0.226 0.083 -0.051 0.176 -0.068 0 -0.086 0 -0.195 0 -0.056

12 3 0 0 0.087 0.033 0.003 -0.076 -0.228 -0.047 0 0 -0.085 0 0 -0.019 -0.226 0.155 -0.051 0.041 -0.068 0 -0.086 0 -0.195 0 -0.056

12 3 0 0 0.087 0.033 0.003 -0.076 -0.375 -0.047 0 0 -0.075 0 0 -0.019 -0.226 0.189 -0.051 0.041 -0.068 0 -0.086 0 -0.195 0 -0.056

12 2 0 0 0.087 0.033 0.003 -0.076 -0.228 -0.047 0 0 -0.075 0 0 -0.019 -0.226 0.155 -0.017 0.041 -0.068 0 -0.086 0 -0.195 0 -0.056

12 2 0 0 0.087 0.033 0.003 -0.076 -0.228 -0.047 0 0 -0.075 0 0 -0.019 -0.278 0.155 -0.051 0.041 -0.068 0 -0.086 0 -0.195 0 -0.056

12 2 0 0 0.087 0.033 0.003 -0.076 -0.228 -0.047 0 0 -0.075 0 0 0.022 -0.226 0.155 -0.051 0.041 -0.068 0 -0.086 0 -0.195 0 -0.056

12 2 0 0 0.087 0.033 0.003 -0.076 -0.228 -0.047 0 0 -0.075 0 0 -0.019 -0.226 0.155 -0.051 0.041 -0.068 -0.048 -0.086 0.147 -0.195 0 -0.056

12 2 0 0 0.087 0.033 0.003 -0.076 -0.228 -0.047 0 0 -0.075 0.15 0 -0.019 -0.226 0.155 -0.051 0.041 -0.068 0 -0.086 0.155 -0.195 0 -0.056

12 2 0 0 0.087 0.033 0.003 -0.076 -0.259 -0.047 0 0 -0.075 0.15 0 -0.019 -0.226 0.155 -0.051 0.041 -0.068 0 -0.086 0 -0.236 0 -0.056

12 2 0 0 0.087 0.033 -0.05 -0.076 -0.228 -0.047 0 0 -0.075 0 0 -0.019 -0.226 0.155 -0.051 0.041 -0.068 0 -0.086 0 -0.195 0 -0.056

12 2 0 0 0.087 0.033 0.003 -0.076 -0.228 -0.047 0 0.171 -0.075 0 0 -0.019 -0.226 0.083 -0.051 0.041 -0.068 0 -0.086 0 -0.195 0 -0.056

12 2 0 0 0.087 0.033 0.003 -0.076 -0.228 -0.047 0 0 -0.075 0.15 0 -0.019 -0.226 0.155 -0.051 0.041 -0.068 0 -0.086 0 0.017 0 -0.056

12 2 0 0 0.087 0.033 0.003 -0.081 -0.228 -0.047 0 0 -0.075 0 0 -0.019 -0.112 0.155 -0.051 0.041 -0.068 0 -0.086 0 -0.195 0 -0.056

12 2 0 0 0.087 0.033 0.003 -0.076 -0.228 -0.047 0 0 -0.075 0 0 -0.019 -0.226 0.155 -0.051 0.041 -0.068 0 -0.086 0 -0.201 0 -0.056

12 2 0 -0.202 0.087 0.033 0.003 -0.076 -0.228 -0.047 0 0 -0.075 0.15 0 -0.019 -0.226 0.155 -0.051 0.041 -0.068 0 -0.086 0 -0.195 0 -0.056

12 2 0 0 0.087 0.033 0.003 -0.081 -0.228 -0.047 0 0 -0.075 0 0 -0.019 -0.226 0.155 -0.051 0.041 -0.053 0 -0.086 0 -0.195 0 -0.056

12 2 0 0 0.087 0.033 0.003 -0.076 -0.228 -0.047 0 0 -0.075 0.15 0 -0.019 -0.226 0.155 -0.051 -0.015 -0.068 0 -0.086 0 -0.236 0 -0.056

12 2 0 0 0.087 0.033 0.069 -0.076 -0.228 -0.047 0 0 -0.075 0 0 -0.019 -0.226 0.155 -0.051 0.041 -0.068 0 -0.086 0 -0.195 0 -0.056

12 2 0 0 0.087 0.033 0.003 -0.076 -0.228 -0.047 0 0 -0.075 0 0.074 0.035 -0.226 0.083 -0.051 0.041 -0.068 0 -0.086 0 -0.195 0 -0.056

12 2 0 0 0.087 0.033 0.003 -0.076 -0.228 -0.047 0 0 -0.075 0 0 -0.019 -0.226 0.155 -0.123 0.041 -0.068 0 -0.086 0 -0.195 0 -0.056

12 3 0 0 0.087 0.033 0.003 -0.076 -0.228 -0.047 0 0 -0.024 0.15 0 -0.019 -0.226 0.155 -0.051 0.041 -0.068 0 -0.086 0 -0.195 0 -0.056

12 2 0 0 0.087 0.033 0.003 -0.076 -0.228 -0.047 0 -0.041 -0.075 0 0.074 -0.019 -0.226 0.083 -0.051 0.041 -0.068 0 -0.086 0 -0.195 0 -0.056

12 2 0 0 0.087 0.033 0.003 -0.076 -0.228 -0.047 0 0 -0.075 0 0 -0.019 -0.226 0.189 -0.051 0.041 -0.068 0 -0.086 0 -0.153 0 -0.056

12 2 -0.061 0 0.087 0.033 0.003 -0.076 -0.228 -0.047 0 0 -0.075 0 0 -0.019 -0.226 0.189 -0.051 0.041 -0.068 0 -0.086 0 -0.195 0 -0.056

12 2 0 0 0.087 0.033 0.003 -0.081 -0.228 -0.047 0 -0.063 -0.075 0 0 -0.019 -0.226 0.155 -0.051 0.041 -0.068 0 -0.086 0 -0.195 0 -0.056

12 2 0 0 0.087 0.033 0.003 -0.076 -0.228 -0.047 0 0 -0.075 0 0 -0.019 -0.226 0.155 -0.051 0.041 -0.068 0 -0.086 0 -0.195 0.062 -0.056

12 2 0 0 0.087 0.033 0.003 -0.076 -0.228 -0.047 0 0.104 -0.075 0.15 0 -0.019 -0.226 0.155 -0.051 0.041 -0.068 0 -0.086 0 -0.236 0 -0.056

12 1 0 0 0.087 0.033 0.003 -0.076 -0.228 -0.047 0 0 -0.075 0 0 -0.019 -0.226 0.155 -0.051 0.041 -0.068 0 -0.086 0 -0.227 0 -0.056

12 1 0 0 0.122 0.033 0.003 -0.076 -0.228 -0.088 0 0 -0.075 0 0 -0.019 -0.226 0.083 -0.051 0.041 -0.068 0 -0.086 0 -0.195 0 -0.056

12 1 0 0 0.087 0.033 0.033 -0.076 -0.228 -0.047 0 0 -0.075 0 0.074 -0.097 -0.226 0.083 -0.051 0.041 -0.068 0 -0.086 0 -0.195 0 -0.056

12 1 0 0 0.087 0.033 0.003 -0.051 -0.228 -0.047 0 0 -0.075 0 0 -0.019 -0.226 0.155 -0.051 0.041 -0.068 0 -0.086 0.147 -0.195 0 -0.056

12 1 0 0 0.087 0.033 0.003 -0.076 -0.228 -0.047 0 0 -0.075 0 0 -0.019 -0.226 0.155 -0.051 0.041 -0.068 0 -0.086 0 -0.195 0 -0.235

12 1 0 0 0.087 0.033 0.003 -0.076 -0.198 -0.047 0 0 -0.075 0 0 -0.019 -0.226 0.155 -0.051 0.041 -0.068 0 -0.086 0 -0.195 0 -0.056

12 1 0 0 0.087 0.033 0.003 -0.076 -0.228 -0.047 0 0 -0.075 0 0.074 -0.019 -0.226 0.083 -0.051 0.041 -0.068 0 -0.086 0 -0.195 0.141 -0.056

12 1 0 0 0.087 0.033 0.003 -0.076 -0.228 -0.047 -0.143 0 -0.075 0 0.074 -0.019 -0.226 0.083 -0.051 0.041 -0.068 0 -0.086 0 -0.195 0 -0.056

12 1 0 0 0.087 0.033 0.003 -0.124 -0.228 -0.047 0 0 -0.075 0 0 -0.019 -0.226 0.155 -0.051 0.041 -0.068 0 -0.086 0 -0.195 0 -0.056

12 1 0 0 0.087 0.033 0.003 -0.076 -0.228 -0.047 0 0 -0.075 0 0 -0.019 -0.226 0.189 -0.051 0.041 -0.068 0 -0.086 0 -0.195 -0.054 -0.056

12 1 0 0 0.087 0.033 0.003 -0.076 -0.228 -0.047 0 0 -0.075 0 0.074 -0.019 -0.226 0.083 -0.051 0.041 -0.068 0 -0.086 0 -0.195 0.025 -0.056

12 1 0 0 0.087 0.033 0.003 -0.076 -0.228 -0.047 0 0 -0.075 0.15 0 -0.019 -0.226 0.155 -0.051 0.041 -0.068 0 -0.072 0 -0.195 0 -0.056

12 1 0 0 0.122 0.033 0.003 -0.076 -0.228 -0.047 0 0 -0.075 0 0 -0.019 -0.226 0.083 -0.051 0.176 -0.068 0 -0.086 0 -0.167 0 -0.056

12 1 0 0 0.087 0.033 0.003 -0.076 -0.228 -0.047 0 0 -0.075 0.15 0 -0.019 -0.226 0.155 -0.051 -0.159 -0.068 0 -0.086 0 -0.096 0 -0.056

12 1 0 0 0.087 0.033 0.003 -0.076 -0.228 -0.047 0 0 0.003 0 0.074 -0.019 -0.226 0.083 -0.051 0.041 -0.068 0 -0.086 0 -0.195 0 -0.056

12 1 0 0 0.087 0.033 0.003 -0.076 -0.228 -0.047 0 0 -0.075 0 0 -0.019 -0.226 0.189 -0.051 0.041 -0.068 0 0.024 0 -0.195 0 -0.056

12 1 0 0 0.087 0.033 0.003 -0.076 -0.228 -0.047 0 0 -0.075 0 0 -0.019 -0.264 0.189 -0.051 0.041 -0.068 0 -0.086 0 -0.195 0 -0.056

12 1 0 0 0.087 0.033 0.003 -0.299 -0.228 -0.047 0 0 -0.075 0 0 -0.019 -0.226 0.083 -0.051 0.041 -0.068 0 -0.086 0 -0.195 0 -0.056

12 1 0 0 0.087 0.033 0.003 -0.081 -0.228 -0.047 0 0 -0.075 0 -0.103 -0.019 -0.226 0.155 -0.051 0.041 -0.068 0 -0.086 0 -0.195 0 -0.056

12 1 0 0 0.087 0.033 0.003 -0.076 -0.228 -0.047 0 -0.126 -0.075 0.15 0 -0.019 -0.226 0.155 -0.051 -0.159 -0.068 0 -0.086 0 -0.236 0 -0.056

12 1 0 0 0.087 0.033 0.003 -0.076 -0.224 -0.047 0 0 -0.075 0.15 0 -0.019 -0.226 0.155 -0.051 0.041 -0.068 0 -0.086 -0.129 -0.236 0 -0.056

12 1 0 0 0.087 0.033 0.003 -0.076 -0.228 -0.047 0 0 -0.075 0.15 0 -0.019 -0.226 0.155 -0.051 0.041 -0.068 0 -0.086 0 -0.195 0 -0.251

12 1 0 0 0.087 0.033 0.003 -0.076 -0.228 -0.047 0 0 -0.075 0 0.074 -0.019 -0.226 0.083 -0.051 0.041 -0.068 0 -0.086 0.076 -0.195 -0.095 -0.056

12 1 0 0 0.087 0.033 0.003 -0.076 -0.228 -0.047 0 0 -0.075 0.083 0 -0.019 -0.226 0.155 -0.051 0.041 -0.068 0 -0.086 0 -0.195 0 -0.056

12 1 0 0 0.087 0.033 0.003 -0.076 -0.228 -0.047 0 0 -0.075 0 0 -0.019 -0.226 0.189 -0.051 0.041 -0.068 0 -0.086 0 -0.322 0 -0.056

12 1 0 0 0.087 0.033 0.003 -0.076 -0.228 -0.047 0 0 -0.075 0 0.074 -0.019 -0.226 0.303 -0.051 0.041 -0.068 0 -0.086 0 -0.195 0 -0.056

12 1 0 0 0.087 0.033 0.003 -0.076 -0.228 -0.047 0 0 -0.075 0 0 -0.019 -0.226 0.155 -0.051 0.041 -0.068 0 -0.086 0 -0.195 0 -0.018

12 1 0 0 0.087 0.033 0.033 -0.076 -0.228 -0.047 0 0 -0.075 0 0.074 -0.084 -0.226 0.083 -0.051 0.041 -0.068 -0.003 -0.086 0 -0.195 0 -0.056

12 1 0 0 -0.013 0.033 0.003 -0.076 -0.228 -0.047 0 0 -0.075 0 0 -0.019 -0.226 0.155 -0.051 0.041 -0.068 0 -0.086 0 -0.195 0 -0.056

12 1 0 0 0.087 0.033 -0.036 -0.076 -0.224 -0.047 0 0 -0.075 0.15 0 -0.019 -0.226 0.155 -0.051 0.041 -0.068 0 -0.086 0 -0.236 0 -0.056

12 1 0 0 0.087 0.033 0.003 -0.076 -0.228 -0.047 0 0 -0.075 0 0 -0.019 -0.226 0.111 -0.051 0.041 -0.068 0 -0.086 0 -0.195 0 -0.056

12 1 0 0 0.087 0.033 0.033 0.07 -0.228 -0.047 0 0 -0.075 0 0.074 -0.057 -0.226 0.083 -0.051 0.041 -0.068 0 -0.086 0 -0.195 0 -0.056

12 1 0 0 0.087 0.033 0.033 -0.076 -0.228 -0.047 -0.143 0 -0.075 0 0.074 -0.084 -0.226 0.083 -0.051 0.041 -0.068 -0.179 -0.086 0 -0.195 0 -0.056

12 1 0 0 -0.008 0.033 0.003 -0.076 -0.228 -0.047 0 0 -0.075 0 0 -0.019 -0.226 0.189 -0.051 0.041 -0.068 0 -0.086 0 -0.195 0 -0.056

12 1 0 0 0.087 0.033 0.003 -0.076 -0.224 -0.047 0 0 -0.075 0.15 0 -0.019 -0.226 0.155 -0.051 0.041 -0.068 0 -0.086 0.036 -0.236 0 -0.056

12 1 0 0 0.087 0.033 0.003 -0.076 -0.228 -0.047 0 0 -0.075 0 0 -0.019 -0.226 0.189 -0.051 0.09 -0.068 0 -0.086 0 -0.195 0 -0.056

12 1 0.258 0 0.087 0.033 0.003 -0.076 -0.228 -0.047 0 0 -0.075 0.15 0 -0.019 -0.226 0.155 -0.051 0.041 -0.068 0 -0.086 0 -0.195 0 -0.056

12 1 0 0 0.087 0.033 0.003 -0.076 -0.228 -0.047 0.016 0 -0.075 0 0 -0.019 -0.226 0.189 -0.051 0.041 -0.068 0 -0.086 0 -0.195 0 -0.056

12 1 0 0 0.087 0.033 0.003 -0.076 -0.228 -0.047 0 0 -0.075 0 0 -0.019 -0.226 0.155 -0.051 0.041 -0.068 0 -0.116 0 -0.195 0 -0.056

12 1 0 -0.004 0.087 0.033 0.003 -0.076 -0.228 -0.047 0 0 -0.075 0 0 -0.019 -0.226 0.155 -0.051 0.041 -0.068 0 -0.086 0 -0.195 0 -0.056

12 1 0 0 0.087 0.033 0.003 -0.076 -0.228 -0.047 0.099 0 -0.075 0 0 -0.019 -0.226 0.155 -0.051 0.041 -0.068 0 -0.086 0 -0.195 0 -0.056

12 1 0 0 0.087 0.078 0.003 -0.076 -0.228 -0.047 0 0 -0.075 0.15 0 -0.019 -0.226 0.155 -0.051 0.041 -0.068 0 -0.086 0 -0.195 0 -0.056

12 1 0 0 0.087 0.033 0.003 -0.076 -0.228 -0.047 0 0 -0.075 0.279 0 -0.019 -0.226 0.155 -0.051 0.041 -0.068 0 -0.086 0 -0.236 0 -0.056

12 1 0 0 0.087 0.033 0.003 -0.076 -0.228 -0.08 0 0 -0.075 0.15 0 -0.019 -0.226 0.155 -0.051 0.041 -0.068 0 -0.086 0 -0.236 0 -0.056

12 1 0 0 0.087 0.033 0.003 -0.076 -0.228 -0.047 0 0 -0.075 0 0 -0.019 -0.226 0.155 -0.051 0.041 -0.068 0 0.01 0 -0.195 0 -0.056

12 1 0 0 0.087 0.033 0.003 -0.076 -0.213 -0.047 0 0 -0.075 0 0.074 -0.019 -0.226 0.083 -0.051 0.041 -0.068 0 -0.086 0 -0.195 0 -0.056

12 1 0 0 0.087 0.033 0.003 -0.076 -0.228 -0.047 0 0 -0.075 0 0 -0.107 -0.226 0.155 -0.051 0.041 -0.068 0 -0.086 0 -0.195 0 -0.056

12 1 0 0 0.087 0.033 0.033 -0.076 -0.228 -0.047 0 0 -0.075 0 0.074 -0.084 -0.226 0.083 -0.051 0.041 -0.068 0 -0.1 0 -0.195 0 -0.056

12 1 -0.042 0 0.087 0.033 0.003 -0.076 -0.228 -0.047 0 0 -0.075 0.15 0 -0.019 -0.226 0.155 -0.051 0.041 -0.068 0 -0.086 0 -0.236 0 -0.056

12 1 0 0 0.087 0.033 0.003 -0.076 -0.228 -0.047 -0.031 0 -0.075 0.15 0 -0.019 -0.226 0.155 -0.051 0.041 -0.068 0 -0.086 0 -0.236 0 -0.056

12 1 0 0 0.087 0.033 0.003 -0.076 -0.228 -0.047 -0.043 0 -0.075 0 0 -0.019 -0.226 0.155 -0.051 0.041 -0.068 0 -0.086 0 -0.195 0 -0.056

12 1 0 0 0.087 0.033 0.003 -0.076 -0.228 -0.047 0 0 -0.075 0.15 0 -0.121 -0.226 0.155 -0.051 0.041 -0.068 0 -0.086 0 -0.195 0 -0.056

12 1 0 0 0.087 0.033 0.003 -0.076 -0.228 -0.047 0 0 -0.075 0 0 -0.019 -0.226 0.189 -0.051 -0.156 -0.068 0 -0.086 0 -0.195 0 -0.056

13 1504 -0.021 -0.015 -0.086 0 0 0.116 0 -0.124 -0.063 0 0 0 0.145 0.09 0 0 0.208 0.093 0.061 -0.023 0 0 -0.049 -0.16 0.003

13 513 -0.021 -0.015 0 0 0 0.116 0 -0.124 -0.063 0 0 0 0.145 0.123 0 0 0.216 0.093 0.061 -0.023 0 0.009 -0.02 -0.16 0.003

13 513 -0.021 -0.015 0 0 0 0.116 0 -0.124 -0.063 0 0 0 0.145 0.123 0 0 0.216 0.093 0.061 -0.023 0 0 -0.02 -0.16 0.003

13 245 -0.021 -0.015 0 0 0 0.116 0 -0.124 -0.166 0 0 0 0.145 0.123 -0.008 -0.02 0.216 0.133 0.061 -0.023 0 0 -0.02 -0.197 0.003

13 212 -0.021 -0.015 0 0 0 0.116 0 -0.124 -0.166 0 0 0 0.145 0.123 0.15 -0.02 0.216 0.133 0.111 -0.023 0 0 -0.02 -0.197 0.003

13 111 -0.021 -0.015 0 0 0 0.116 0 -0.124 -0.166 0 0.025 0 0.145 0.123 -0.008 -0.02 0.216 0.133 0.061 -0.023 0 0 -0.02 -0.294 0.003

13 99 -0.021 -0.015 0 0 0 0.116 0 -0.124 -0.166 0 0 0 0.145 0.123 -0.008 -0.02 0.216 0.211 0.061 -0.023 0 0 -0.02 -0.197 0.003

13 100 -0.021 -0.015 0 0.02 0 0.116 0 -0.124 -0.166 0 0.025 0 0.145 0.123 -0.008 -0.02 0.216 0.133 0.061 -0.023 0 0 -0.02 -0.294 0.003

13 62 -0.021 -0.015 0 0 0 0.116 0 -0.124 -0.063 0 0 0 0.145 0.123 0 0 0.216 0.093 0.061 -0.023 0 0.009 -0.02 -0.26 0.003

13 31 -0.021 -0.015 -0.086 0 0 0.116 0 -0.124 -0.063 0 0 0 0.145 0.09 0 0 0.208 0.093 0.061 -0.023 0.065 0 -0.049 -0.16 0.003

13 15 -0.021 -0.015 0 0 0 0.116 -0.179 -0.124 -0.063 0 0 0 0.145 0.123 0 0 0.216 0.093 0.061 -0.023 0 0.009 -0.02 -0.16 0.003

13 13 -0.021 -0.015 0 0 0 0.116 0 -0.124 -0.166 0 0.025 0 0.145 0.123 -0.008 -0.02 0.216 0.133 0.061 -0.023 0 0 -0.02 -0.197 0.003

13 13 -0.021 -0.015 -0.086 0 0 0.116 0 -0.124 -0.063 0 0 0 0.145 0.09 0 0 0.208 0.093 0.08 -0.023 0 0 -0.049 -0.16 0.003

13 11 -0.021 -0.015 -0.086 0 0 0.116 0 -0.124 -0.063 0 0 0 0.145 0.09 0 0 0.249 0.093 0.061 -0.023 0 0 -0.049 -0.16 0.003

13 10 -0.021 -0.015 -0.086 0 0 -0.036 0 -0.124 -0.063 0 0 0 0.145 0.09 0 0 0.208 0.093 0.061 -0.023 0 0 -0.049 -0.16 0.003

13 8 -0.021 -0.015 0 0 0 0.116 0 -0.232 -0.063 0 0 0 0.145 0.123 0 0 0.216 0.093 0.061 -0.023 0 0.009 -0.02 -0.16 0.003

13 7 -0.021 -0.015 0 0 0 0.116 0 -0.124 -0.166 0 0.025 0 0.145 0.05 -0.008 -0.02 0.216 0.133 0.061 -0.023 0 0 -0.02 -0.294 0.003

13 7 -0.021 -0.015 0 0 0 0.116 0 -0.124 -0.166 0 0.025 0 0.145 0.123 -0.008 -0.02 0.216 0.133 0.061 -0.023 0 0 0.023 -0.294 0.003

13 7 -0.021 -0.015 -0.086 0 0 0.116 0 -0.124 -0.063 0 0 0 0.145 0.09 0 0 0.208 0.093 0.061 -0.023 0 0 -0.049 -0.16 0.126

13 6 -0.021 -0.015 -0.086 0 0 0.116 0 -0.124 -0.063 0 0.171 0 0.145 0.09 0 0 0.208 0.093 0.061 -0.023 0 0 -0.049 -0.16 0.003

13 6 -0.021 -0.015 -0.086 0 0 0.116 0 -0.075 -0.063 0 0 0 0.145 0.09 0 0 0.208 0.093 0.061 -0.023 0 0 -0.049 -0.16 0.003

13 6 -0.021 -0.015 -0.086 0 0 0.116 0 -0.124 -0.063 0 0 0 0.145 0.09 0 0 0.208 0.093 0.061 -0.023 0.099 0 -0.049 -0.16 0.003

13 5 -0.021 -0.015 -0.086 0 0 0.116 0 -0.124 -0.063 0 0 0 0.145 0.09 0 0 0.208 0.093 0.061 -0.023 0 0 -0.049 -0.193 0.003

13 4 -0.021 -0.015 -0.086 -0.014 0 0.116 0 -0.124 -0.063 0 0 0 0.145 0.09 0 0 0.208 0.093 0.061 -0.023 -0.017 0 -0.049 -0.16 0.003

13 4 -0.021 -0.015 0 0 0 0.116 0 -0.124 -0.166 0 -0.048 0 0.145 0.123 -0.008 -0.02 0.216 0.133 0.061 -0.023 0 0 -0.02 -0.083 0.003

13 4 -0.021 -0.015 -0.086 0 0 0.116 0 -0.124 -0.063 0 0 0 0.145 0.09 0 0 0.208 0.093 0.061 -0.023 0.056 0.039 -0.049 -0.16 0.003

13 4 -0.021 -0.015 0 0 0 0.116 0 -0.124 -0.063 0 -0.05 0 0.145 0.123 0 0 0.216 0.093 0.061 -0.023 0 0.009 -0.02 -0.16 0.003

13 4 -0.021 -0.015 0 0 0 0.116 0 -0.124 -0.166 0 0 0 0.145 0.123 0.15 -0.02 0.216 0.133 0.111 -0.023 0 0 0.12 -0.197 0.003

13 4 -0.021 -0.015 -0.086 0 0 0.116 0 -0.124 -0.063 0 0 0 0.145 0.09 0 0 0.208 0.093 0.061 -0.023 0 0.159 -0.049 -0.16 0.003

13 8 -0.021 -0.015 0 0 0 0.116 0 -0.124 -0.166 0 0.025 0 0.145 0.123 -0.008 -0.02 0.216 0.133 0.061 -0.023 -0.108 0 -0.02 -0.294 0.003

13 4 -0.021 -0.015 0 0 -0.142 0.116 0 -0.124 -0.063 0 0 0 0.145 0.123 0 0 0.216 0.093 0.061 -0.023 0 0.009 -0.02 -0.16 0.003

13 4 -0.021 -0.015 0 0 0 0.116 0 -0.124 -0.063 0 0 0 0.145 0.123 0 -0.042 0.216 0.093 0.061 -0.023 0 0.009 -0.02 -0.16 0.003

13 4 -0.021 -0.015 0 0 0 0.116 0 -0.124 -0.166 0 0.025 0 0.145 0.123 -0.008 -0.02 0.216 0.133 0.061 -0.025 0 0 -0.02 -0.294 0.003

13 4 -0.021 -0.015 -0.086 0 0 0.116 0 -0.124 -0.063 0 0 0 0.145 0.09 0 0 0.208 0.093 0.061 -0.023 0 0.135 -0.049 -0.16 0.003

13 4 -0.021 -0.015 -0.086 0 0 0.116 0 -0.124 -0.063 0 0 0 0.145 0.09 0 0 0.208 0.093 0.061 -0.023 0 0 0.058 -0.16 0.003

13 4 -0.021 -0.015 0 0 0.016 0.116 0 -0.124 -0.063 0 0 0 0.145 0.123 0 0 0.216 0.093 0.061 -0.023 0 0 -0.02 -0.16 0.003

13 4 -0.021 -0.015 0 0 0 0.116 0 -0.124 -0.063 0 -0.073 0 0.145 0.123 0 0 0.216 0.093 0.061 -0.023 0 0 -0.02 -0.16 0.003

13 3 -0.021 -0.015 -0.086 0 0 0.116 0 -0.124 -0.063 0 -0.03 0 0.145 0.09 0 0 0.208 0.093 0.061 -0.023 0 0 -0.049 -0.16 0.003

13 3 -0.021 -0.015 0 0 0 0.116 0 -0.124 -0.199 0 0 0 0.145 0.123 0 0 0.216 0.093 0.061 -0.023 0 0 -0.02 -0.16 0.003

13 3 -0.021 -0.015 0 0 0 0.116 0 -0.124 -0.166 0 0 0 0.145 0.123 -0.008 -0.02 0.216 0.211 0.061 -0.023 -0.123 0 -0.02 -0.197 0.003

13 2 -0.021 -0.015 -0.086 0 0 0.116 0 -0.124 -0.063 0 0 0 0.145 0.09 0 0 0.208 0.093 0.061 -0.023 0 0 -0.049 -0.263 0.003

13 2 -0.021 -0.015 0 0 0 0.116 0 -0.124 -0.063 0 0 0 0.145 0.123 0 0 0.216 0.093 0.061 -0.052 0 0.009 -0.02 -0.16 0.003

13 2 -0.021 -0.015 0 0 0 0.116 0 -0.124 -0.063 0 0 0 0.145 0.092 0 0 0.216 0.093 0.061 -0.023 0 0.009 -0.02 -0.16 0.003

13 3 -0.021 -0.015 0 0.101 0 0.116 0 -0.124 -0.063 0 0 0 0.145 0.123 0 0 0.216 0.093 0.061 -0.023 0 0.009 -0.02 -0.26 0.003

13 2 -0.021 -0.015 0 0 0 0.116 0 -0.124 -0.063 -0.114 0 0 0.145 0.123 0 0 0.216 0.093 0.061 -0.023 0 0.009 -0.02 -0.16 0.003

13 2 -0.021 -0.015 -0.086 0 0 0.116 0 -0.124 -0.063 0 0 0 0.145 0.09 0 -0.02 0.208 0.093 0.061 -0.023 0 0 -0.049 -0.16 0.003

13 2 -0.021 -0.015 -0.086 0 0 0.116 0 -0.124 -0.063 0 0 0 0.145 0.09 0 0 0.208 0.093 0.061 -0.023 -0.07 0 -0.049 -0.16 0.003

13 2 -0.021 -0.015 -0.086 0 0 0.116 0 -0.124 -0.063 0 0 0 0.145 0.09 0 0 0.208 0.093 0.061 -0.023 0 0 -0.049 -0.16 0.037

13 2 -0.021 -0.015 0 0 0 0.116 0 -0.124 -0.166 0 0 0 0.145 0.123 -0.008 -0.02 0.216 0.133 0.061 -0.023 0 0.028 -0.02 -0.197 0.003

13 2 -0.021 -0.015 0 0 0 0.116 0 -0.124 -0.166 0 0 0 0.145 0.123 0.15 -0.02 0.216 0.133 0.04 -0.023 0 0 -0.02 -0.197 0.003

13 2 -0.021 -0.015 0 0 0 0.116 0 -0.124 -0.063 0 0 0 0.145 0.123 0 0 0.216 0.093 0.061 -0.023 0 0.009 -0.02 -0.159 0.003

13 2 -0.021 -0.015 0 0 0 0.116 0 -0.124 -0.063 0 0 0.114 0.145 0.123 0 0 0.216 0.093 0.061 -0.023 0 0 -0.02 -0.16 0.003

13 2 -0.021 -0.015 0 0 0 0.116 0 -0.124 -0.063 0 0 0 0.145 0.123 0 0 0.216 0.093 0.061 -0.023 0 -0.041 -0.02 -0.16 0.003

13 2 -0.021 -0.015 0 0 0 0.116 0 -0.124 -0.063 0 0 0 0.145 0.123 0 0 0.216 0.093 0.061 -0.023 0 0 -0.02 -0.067 0.003

13 2 -0.021 -0.015 0 0 0 0.116 0 -0.124 -0.063 0 0 0 0.145 0.123 0 0 0.216 0.093 0.061 -0.023 0 0 -0.02 -0.16 0.027

13 2 -0.021 -0.015 0 0 0 0.066 0 -0.124 -0.063 0 0 0 0.145 0.123 0 0 0.216 0.093 0.061 -0.023 0 0.009 -0.02 -0.16 0.003

13 2 -0.021 -0.015 0 0.02 0 0.258 0 -0.124 -0.166 0 0.025 0 0.145 0.123 -0.008 -0.02 0.216 0.133 0.061 -0.023 0 0 -0.02 -0.294 0.003

13 2 -0.021 -0.015 -0.086 0 0 0.116 0 -0.124 -0.063 0 0 0 0.145 0.09 0 0 0.208 0.093 0.061 -0.023 0 0 -0.049 -0.052 0.003

13 2 -0.021 -0.015 0 0 0 0.116 0 -0.124 -0.063 0 0 0 0.145 0.123 0.091 0 0.216 0.093 0.061 -0.023 0 0.009 -0.02 -0.16 0.003

13 2 -0.021 -0.015 -0.086 0 0 0.116 0 -0.124 -0.063 0 0 0 0.145 0.09 0 0 0.208 0.093 0.061 -0.023 0 0 -0.049 -0.249 0.003

13 2 -0.021 -0.015 -0.086 0 0.064 0.116 0 -0.124 -0.063 0 0 0 0.145 0.09 0 0 0.208 0.093 0.061 -0.023 0 0 -0.049 -0.16 0.003

13 1 -0.021 -0.015 0 0 0 0.116 0 -0.124 -0.063 0 0 -0.191 0.145 0.123 0 0 0.216 0.093 0.061 -0.023 0 0.009 -0.02 -0.16 0.003

13 1 -0.021 -0.015 -0.086 0.073 0 0.116 0 -0.124 -0.063 0 0 0 0.145 0.09 0 0 0.208 0.093 0.061 -0.023 0 0 -0.049 -0.16 0.003

13 1 -0.021 -0.015 0 0 0 0.116 0 -0.02 -0.063 0 0 0 0.145 0.123 0 0 0.216 0.093 0.061 -0.023 0 0.009 -0.02 -0.16 0.003

13 1 -0.021 -0.015 0 0 0 0.116 0 -0.124 -0.166 0 0 0 0.145 0.123 0.15 -0.02 0.216 0.133 0.364 -0.023 0 0 -0.02 -0.197 0.003

13 1 -0.021 -0.015 -0.086 0 0 0.116 0 -0.124 -0.063 0 0 0 0.145 0.214 0 0 0.208 0.093 0.061 -0.023 0 0 -0.049 -0.16 0.003

13 1 -0.021 -0.015 -0.086 0 -0.019 0.116 0 -0.124 -0.063 0 0 0 0.145 0.09 0 0 0.208 0.093 0.061 -0.023 0.065 0 -0.049 -0.16 0.003

13 1 -0.021 -0.015 0 0 0 0.116 0 -0.124 -0.166 0 0.025 0 0.145 0.123 -0.008 -0.02 0.241 0.133 0.061 -0.023 0 0 -0.02 -0.294 0.003

13 1 -0.021 -0.015 -0.086 0 0 0.237 0 -0.124 -0.063 0 0 0 0.145 0.09 0 0 0.208 0.093 0.061 -0.023 0 0 -0.049 -0.16 0.003

13 1 -0.021 -0.015 -0.086 0 0 0.116 0 -0.124 -0.063 0 0 0 0.145 0.09 0 0 0.208 0.093 0.087 -0.023 0 0 -0.049 -0.16 0.003

13 1 -0.021 -0.015 0 0 0 0.116 0 -0.124 -0.166 0 0.025 0 0.145 0.123 -0.008 -0.02 0.216 0.133 0.031 -0.023 0 0 -0.02 -0.294 0.003

13 1 -0.021 0.098 0 0 0 0.116 0 -0.124 -0.166 0 0 0 0.145 0.123 -0.008 -0.02 0.216 0.133 0.061 -0.023 0 0 -0.02 -0.197 0.003

13 1 -0.021 -0.015 -0.086 0 0 0.116 0 -0.124 -0.063 -0.226 0 0 0.145 0.09 0 0 0.208 0.093 0.061 -0.023 0 0 -0.049 -0.16 0.003

13 1 -0.021 -0.015 0 0 0 0.003 0 -0.124 -0.166 0 0 0 0.145 0.123 -0.008 -0.02 0.216 0.133 0.061 -0.023 0 0 -0.02 -0.197 0.003

13 1 -0.021 -0.015 0 0.02 0 0.116 0 -0.124 -0.166 0 0.025 0 0.145 0.123 -0.038 -0.02 0.216 0.133 0.061 -0.023 0 0 -0.02 -0.294 0.003

13 1 -0.021 -0.015 0 0 0 0.116 0 -0.109 -0.166 0 0 0 0.145 0.123 0.15 -0.02 0.216 0.133 0.111 -0.023 0 0 -0.02 -0.197 0.003

13 1 -0.021 -0.015 -0.086 0 0 0.116 0 -0.124 -0.063 0 0 0 0.145 0.09 0 0 0.208 0.093 0.061 -0.023 0 -0.178 -0.049 -0.16 0.003

13 1 -0.021 -0.015 0 0 0 0.116 0 -0.124 -0.166 0 0 0 0.145 0.123 0.15 -0.02 0.216 0.133 0.111 -0.023 0 0 -0.02 -0.037 0.003

13 1 -0.021 -0.015 0 0.02 0 0.116 0.142 -0.124 -0.166 0 0.025 0 0.145 0.123 -0.008 -0.02 0.216 0.133 0.061 -0.023 0 0 -0.02 -0.294 0.003

13 1 -0.021 -0.015 0 0 0 0.058 0 -0.124 -0.166 0 0.025 0 0.145 0.123 -0.008 -0.02 0.216 0.133 0.061 -0.023 0 0 -0.02 -0.294 0.003

13 1 -0.021 -0.015 0 0 0 0.116 0 -0.124 -0.063 0 0 0 0.145 0.123 0 0 0.216 0.181 0.061 -0.023 0 0 -0.02 -0.16 0.003

13 1 -0.021 -0.141 0 0 0 0.116 0 -0.124 -0.063 0 0 0 0.145 0.123 0 0 0.216 0.093 0.061 -0.023 0 0 -0.02 -0.16 0.003

13 1 -0.021 -0.015 -0.086 0 0 0.116 0 -0.124 -0.063 0 -0.148 0 0.145 0.09 0 0 0.208 0.093 0.061 -0.023 0 0 -0.049 -0.16 0.003

13 1 -0.021 -0.015 0 0.02 0 0.116 0 -0.124 -0.166 0 0.025 0 0.145 0.123 -0.038 -0.02 0.216 0.051 0.061 -0.023 0 0 -0.02 -0.294 0.003

13 1 -0.021 -0.015 -0.086 0 0 0.116 0 -0.124 -0.063 0 0 0 0.145 -0.175 0 0 0.208 0.093 0.061 -0.023 0 0 -0.049 -0.16 0.003

13 1 -0.021 -0.015 -0.086 0 0 0.116 0 -0.124 -0.063 0 0 0 0.145 0.09 0 0 0.208 0.093 0.061 -0.023 0 0 -0.049 -0.16 -0.033

13 1 -0.021 -0.015 0 0 0 0.116 0 -0.124 -0.063 0 0 0 0.145 0.123 0 0 0.275 0.093 0.061 -0.023 0 0 -0.02 -0.16 0.003

13 1 -0.021 -0.015 0 0 0 0.116 0 -0.124 -0.063 0 0 0 0.145 0.123 0 0 0.216 0.093 0.061 -0.023 0 0.009 -0.02 -0.16 0.221

13 1 -0.021 -0.015 0 -0.086 0 0.116 0 -0.124 -0.063 0 0 0 0.145 0.123 0 0 0.216 0.093 0.061 -0.023 0 0 -0.02 -0.16 0.003

13 1 -0.021 -0.015 0 0 0 0.116 0 -0.124 -0.166 0 -0.087 0 0.145 0.123 -0.008 -0.02 0.216 0.133 0.061 -0.023 0 0 -0.02 -0.197 0.003

13 1 -0.021 -0.015 0 0 0 0.116 -0.179 -0.124 -0.063 0 0 0 0.145 0.123 0 0 0.166 0.093 0.061 -0.023 0 0.009 -0.02 -0.16 0.003

13 1 -0.021 -0.015 0 0 0 0.116 0 -0.124 -0.063 0 0 0 0.145 0.123 0.139 0 0.216 0.093 0.061 -0.023 0 0 -0.02 -0.16 0.003

13 1 -0.021 -0.015 0 0 0 0.116 0 0.011 -0.063 0 0 0 0.145 0.123 0 0 0.216 0.093 0.061 -0.023 0 0.009 -0.02 -0.16 0.003

13 1 -0.021 -0.015 -0.086 -0.126 0 0.116 0 -0.124 -0.063 0 0 0 0.145 0.09 0 0 0.249 0.093 0.061 -0.023 0 0 -0.049 -0.16 0.003

13 1 -0.021 -0.015 0 0 0 0.116 -0.072 -0.124 -0.166 0 0 0 0.145 0.123 -0.008 -0.02 0.216 0.211 0.061 -0.023 0 0 -0.02 -0.197 0.003

13 1 -0.021 -0.015 -0.086 0 0 0.116 0 -0.112 -0.063 0 0 0 0.145 0.09 0 0 0.208 0.093 0.061 -0.023 0 0 -0.049 -0.16 0.003

13 1 -0.021 -0.015 0 0 0 0.116 0 -0.124 -0.063 0 0 0 0.145 0.123 0 0 0.216 0.093 0.061 -0.023 0 0 -0.02 -0.282 0.003

13 1 -0.021 -0.015 0 0 0 0.116 0 -0.124 -0.063 0 0 0 0.145 0.123 0 0 0.216 0.093 0.061 -0.023 0 0.009 -0.02 -0.16 -0.242

13 1 -0.021 -0.015 0 0 0 0.116 0 -0.124 -0.063 0 0 0 0.054 0.123 0 0 0.216 0.093 0.061 -0.023 0 0.009 -0.02 -0.16 0.003

13 1 -0.021 -0.015 -0.086 0 0 0.116 0 -0.124 -0.063 0.251 0 0 0.145 0.09 0 0 0.208 0.093 0.061 -0.023 0 0 -0.049 -0.16 0.003

13 1 -0.021 -0.015 -0.086 0 0 0.116 0 -0.124 -0.063 0 0 0 0.269 0.09 0 0 0.208 0.093 0.061 -0.023 0 0 -0.049 -0.16 0.003

13 1 -0.021 -0.015 -0.083 0 0 0.116 0 -0.124 -0.063 0 0 0 0.145 0.123 0 0 0.216 0.093 0.061 -0.023 0 0.009 -0.02 -0.26 0.003

13 1 -0.021 -0.015 -0.086 0 0 0.116 0 -0.124 -0.063 0 0 0 0.145 0.09 0 0 0.208 0.093 0.061 -0.023 0 0 -0.049 -0.16 0.158

13 1 -0.021 -0.015 -0.158 0 0 0.116 0 -0.124 -0.063 0 0 0 0.145 0.09 0 0 0.208 0.093 0.061 -0.023 0 0 -0.049 -0.16 0.003

13 1 -0.021 -0.015 0 0 0 0.116 0 -0.124 -0.063 0 0 0 0.145 0.123 0 0 0.216 0.093 0.061 -0.023 0.054 0 -0.02 -0.16 0.003

13 1 -0.021 -0.015 0 0 0 0.116 0 -0.124 -0.063 0 0 0 0.145 0.123 0 0 0.216 0.093 0.061 -0.023 0 0.009 -0.02 -0.16 -0.122

13 1 -0.021 -0.015 0 0.02 0 0.116 0 -0.124 -0.166 0 0.025 0 0.145 0.123 0.053 -0.02 0.216 0.133 0.061 -0.023 0 0 -0.02 -0.294 0.003

13 1 -0.021 -0.015 0 0 0 0.116 0 -0.124 -0.166 0 0 0 0.145 0.123 0.15 -0.02 0.216 0.133 0.111 -0.023 0 -0.207 -0.02 -0.197 0.003

13 1 -0.021 -0.015 0 0.02 0 0.116 0 -0.124 -0.166 0 0.025 0 0.145 0.123 -0.008 -0.02 0.216 0.133 -0.04 -0.023 0 0 -0.02 -0.294 0.003

13 1 -0.021 -0.015 0 0 0 0.116 0 0.045 -0.166 0 0 0 0.145 0.123 0.15 -0.02 0.216 0.133 0.111 -0.023 0 0 -0.02 -0.197 0.003

13 1 -0.021 -0.015 0 0 0 0.116 0 -0.124 -0.039 0 0 0 0.145 0.123 0 0 0.216 0.093 0.061 -0.023 0 0.009 -0.02 -0.16 0.003

13 1 -0.021 -0.015 0.028 0 0 0.116 0 -0.124 -0.063 0 0 0 0.145 0.09 0 0 0.249 0.093 0.061 -0.023 0 0 -0.049 -0.16 0.003

13 1 -0.021 -0.015 0 0 0 0.116 0 -0.124 -0.063 0 0 0 0.145 0.123 0 0 0.216 0.051 0.061 -0.023 0 0 -0.02 -0.16 0.003

13 1 -0.021 -0.045 -0.086 0 0 0.116 0 -0.124 -0.063 0 0 0 0.145 0.09 0 0 0.208 0.093 0.061 -0.023 0 0 -0.049 -0.16 0.003

13 1 -0.021 -0.015 0 0 0 0.116 0 -0.124 -0.166 0 0.025 0 0.145 0.123 -0.008 -0.02 0.216 -0.005 0.061 -0.023 0 0 -0.02 -0.294 0.003

13 1 -0.021 -0.015 0 0 0 0.116 0 -0.124 -0.063 0 0 0 0.176 0.123 0 0 0.216 0.093 0.061 -0.023 0 0.009 -0.02 -0.16 0.003

13 1 -0.021 -0.015 0 0 0 0.116 0 -0.134 -0.063 0 0 0 0.145 0.123 0 0 0.216 0.093 0.061 -0.023 0 0 -0.02 -0.16 0.003

13 1 -0.021 -0.015 -0.086 0 0 0.116 0 -0.124 -0.063 0 0 0 0.145 0.09 0 0 0.208 0.093 0.061 -0.023 0.004 0 -0.049 -0.16 0.003

13 1 -0.021 -0.015 0 0 0 0.116 0 -0.124 -0.166 0 -0.012 0 0.145 0.123 -0.008 -0.02 0.216 0.133 0.061 -0.023 0 0 0.023 -0.294 0.003

13 1 -0.021 -0.015 0 0 0.098 0.116 0 -0.124 -0.063 0 0 0 0.145 0.123 0 0 0.216 0.093 0.061 -0.023 0 0.009 -0.02 -0.16 0.003

13 1 -0.021 -0.015 0 0 0 0.116 0 -0.124 -0.166 0 0.025 0 0.145 0.123 -0.008 -0.02 0.216 0.133 0.061 -0.023 -0.112 0 -0.02 -0.294 0.003

13 1 -0.021 -0.015 -0.086 0 0 0.116 0 -0.124 -0.063 0 0 0 0.145 0.09 0 0 0.208 0.093 0.061 -0.023 0.065 0.053 -0.049 -0.16 0.003

13 1 -0.021 -0.015 0 0 0 0.116 0 -0.124 -0.166 0 0 0 0.145 0.014 -0.008 -0.02 0.216 0.211 0.061 -0.023 0 0 -0.02 -0.197 0.003

13 1 -0.021 -0.015 -0.086 0 0 0.116 0 -0.124 -0.063 0 0 0 0.145 0.09 0 0 0.177 0.093 0.061 -0.023 0 0 -0.049 -0.16 0.003

13 1 -0.021 -0.015 0 0 0 0.116 0 -0.124 -0.063 0 0 0 0.145 0.123 0 0 0.216 0.093 0.061 -0.023 0 0 0.015 -0.16 0.003

13 1 -0.021 -0.06 0 0 0 0.116 0 -0.124 -0.063 0 0 0 0.145 0.123 0 0 0.216 0.093 0.061 -0.023 0 0 -0.02 -0.16 0.003

13 1 -0.021 -0.015 0 0 0 -0.033 0 -0.124 -0.063 0 0 0 0.145 0.123 0 0 0.216 0.093 0.061 -0.023 0 0.009 -0.02 -0.16 0.003

13 1 -0.021 -0.015 0 0 0 0.116 0 -0.124 -0.063 0 0 0 0.072 0.123 0 0 0.216 0.093 0.061 -0.023 0 0 -0.02 -0.16 0.003

13 1 -0.021 -0.015 -0.049 0 0 0.116 0 -0.124 -0.063 0 0 0 0.145 0.09 0 0 0.208 0.093 0.061 -0.023 0 0 -0.049 -0.16 0.003

13 1 -0.021 -0.015 -0.086 0.012 0 0.116 0 -0.124 -0.063 0 0 0 0.145 0.09 0 0 0.208 0.093 0.061 -0.023 0 0 -0.049 -0.16 0.003

13 1 -0.021 0.032 0 0 0.025 0.116 -0.094 -0.124 -0.166 0 0 0 0.145 0.123 -0.008 -0.02 0.216 0.133 0.061 -0.023 0 0 -0.02 -0.197 0.003

13 1 -0.021 -0.015 0 0 0 0.116 0 -0.124 -0.063 0 0 0 0.145 0.123 0 0 0.12 0.093 0.061 -0.023 0 0.009 -0.02 -0.16 0.003

14 741 0 -0.044 0 0 0.056 -0.023 0.014 0 0.032 0.139 -0.088 0.081 -0.175 0 -0.054 -0.132 -0.092 0.031 -0.156 0.109 -0.368 0.028 -0.072 0.145 0

14 652 0 -0.044 0 0 0.056 -0.023 0.014 0 0.032 0.139 -0.088 0.081 -0.175 0 -0.054 -0.132 -0.092 0.031 -0.156 0.109 -0.136 0.028 -0.072 0.145 0

14 560 0 -0.044 0 0 0.056 -0.023 0.014 0 0.032 0.139 -0.088 0.081 -0.175 0 -0.054 -0.132 -0.092 0.031 -0.062 0.109 -0.368 0.028 -0.072 0.145 0

14 184 0 -0.044 0 0 0.056 -0.023 0.014 0 0.032 0.139 -0.088 0.081 -0.175 0 -0.054 -0.132 -0.109 0.031 -0.156 0.109 -0.368 0.028 -0.072 0.145 0

14 210 0 -0.044 0 0 0.056 -0.023 0.014 0 0.032 0.139 -0.088 0.081 -0.175 0 -0.054 -0.132 -0.092 0.031 -0.156 0.042 -0.368 0.028 -0.072 0.145 0

14 126 0 -0.034 0 0 0.056 -0.023 0.014 0 0.032 0.139 -0.088 0.081 -0.175 0 -0.054 -0.196 -0.092 0.031 -0.156 0.109 -0.368 0.028 -0.072 0.145 0

14 57 -0.028 -0.044 0 0 0.056 -0.023 0.014 0 0.032 0.139 -0.088 0.081 -0.175 0 -0.054 -0.132 -0.109 0.031 -0.156 0.109 -0.368 0.028 -0.072 0.145 0

14 38 0 -0.044 0 0 0.056 -0.023 0.014 -0.001 0.032 0.139 -0.088 0.081 -0.175 0 -0.054 -0.132 -0.109 0.031 -0.156 0.109 -0.368 0.028 -0.072 0.145 0

14 28 0 -0.044 0 0 0.056 -0.023 0.014 0 0.032 0.139 -0.088 0.081 -0.175 -0.085 -0.054 -0.132 -0.092 0.031 -0.156 0.109 -0.368 0.028 -0.072 0.145 0

14 20 0 -0.033 0 0 0.056 0.184 0.014 0 0.032 0.139 -0.088 0.081 -0.175 0 -0.054 -0.132 -0.092 0.031 -0.156 0.042 -0.368 0.028 -0.072 0.145 0

14 14 0 -0.044 0 -0.029 0.056 -0.023 0.014 0 0.032 0.139 -0.088 0.081 -0.175 0 -0.054 -0.132 -0.092 0.031 -0.156 0.109 -0.368 0.028 -0.072 0.145 0

14 13 0 -0.044 0 0 0.056 -0.023 0.014 0 0.032 0.139 -0.088 0.081 -0.175 0 -0.139 -0.132 -0.092 0.031 -0.156 0.109 -0.368 0.028 -0.072 0.145 0

14 12 0 -0.044 0 0 0.056 -0.023 0.014 0 0.032 0.139 -0.088 0.081 -0.175 0 -0.054 -0.132 -0.092 0.031 -0.156 0.025 -0.368 0.028 -0.072 0.145 0

14 11 0 -0.044 0 0 0.056 -0.023 0.014 0 0.032 0.147 -0.088 0.081 -0.175 0 -0.054 -0.132 -0.092 0.031 -0.156 0.109 -0.368 0.028 -0.072 0.145 0

14 9 0 -0.044 0 0 0.056 -0.023 0.014 0 0.032 0.139 -0.088 0.081 -0.175 0 -0.054 -0.132 -0.092 -0.078 -0.156 0.042 -0.368 0.028 -0.072 0.145 0

14 8 0 -0.034 0 0 0.056 0.094 0.014 0 0.032 0.139 -0.088 0.081 -0.175 0 -0.054 -0.196 -0.092 0.031 -0.156 0.109 -0.368 0.028 -0.072 0.145 0

14 8 0 -0.044 0 0 0.056 -0.023 0.014 0 0.032 0.139 -0.088 0.081 -0.175 0 -0.054 -0.132 -0.092 0.031 -0.062 0.101 -0.368 0.028 -0.072 0.145 0

14 8 0 -0.044 0 0 0.056 -0.023 0.014 0 0.032 0.139 -0.088 0.081 -0.175 0 -0.054 -0.132 -0.092 0.031 -0.156 -0.098 -0.368 0.028 -0.072 0.145 0

14 7 0.159 -0.044 0 0 0.056 -0.023 0.014 0 0.032 0.139 -0.088 0.081 -0.175 0 -0.054 -0.132 -0.092 0.031 -0.062 0.109 -0.368 0.028 -0.072 0.145 0

14 6 0 -0.044 0 0 0.056 -0.023 0.014 0 0.032 0.139 -0.088 0.081 -0.282 0 -0.054 -0.132 -0.092 0.031 -0.062 0.109 -0.368 0.028 -0.072 0.145 0

14 6 0 -0.044 0 0 0.053 -0.023 0.014 0 0.032 0.139 -0.088 0.081 -0.175 0 -0.054 -0.132 -0.092 0.031 -0.062 0.109 -0.368 0.028 -0.072 0.145 0

14 6 0 -0.044 0 0 0.056 -0.023 0.014 0 0.032 0.139 -0.088 0.096 -0.175 0 -0.054 -0.132 -0.092 0.031 -0.062 0.109 -0.368 0.028 -0.072 0.145 0

14 4 0 -0.044 0 0 0.056 -0.023 0.014 0 0.032 0.139 -0.088 0.081 -0.175 0 -0.054 -0.282 -0.092 0.031 -0.156 0.042 -0.368 0.028 -0.072 0.145 0

14 4 0 -0.044 0 0 0.056 -0.023 0.014 0 0.032 0.139 -0.088 0.081 -0.175 0 -0.202 -0.132 -0.092 0.031 -0.156 0.042 -0.368 0.028 -0.072 0.145 0

14 4 0 -0.044 0 0 0.056 -0.023 0.014 0 0.247 0.139 -0.088 0.081 -0.175 0 -0.054 -0.132 -0.092 0.031 -0.062 0.109 -0.368 0.028 -0.072 0.145 0

14 4 0 -0.044 -0.056 0 0.056 -0.023 0.014 0 0.032 0.139 -0.088 0.081 -0.175 0 -0.054 -0.132 -0.092 0.031 -0.062 0.109 -0.368 0.028 -0.072 0.145 0

14 4 0 -0.044 0 0 0.056 -0.023 0.014 0 0.032 0.139 -0.006 0.081 -0.175 0 -0.054 -0.132 -0.092 0.031 -0.156 0.109 -0.368 0.028 -0.072 0.145 0

14 4 0 -0.044 0 0 0.056 -0.023 0.014 0 0.032 0.139 -0.088 0.081 -0.175 0 -0.054 -0.132 -0.092 0.031 -0.156 0.109 -0.136 0.028 0.061 0.145 0

14 3 -0.099 -0.044 0 0 0.056 -0.023 0.014 0 0.032 0.139 -0.088 0.081 -0.175 0 -0.054 -0.132 -0.092 0.031 -0.156 0.109 -0.368 0.028 -0.072 0.145 0

14 3 0 -0.033 0 0 0.056 -0.023 0.014 0 0.032 0.139 -0.088 0.081 -0.175 0 -0.054 -0.132 -0.092 0.031 -0.156 0.042 -0.368 0.028 -0.072 0.145 0

14 3 0 -0.034 0 0 0.056 -0.023 0.014 0 0.032 0.139 -0.247 0.081 -0.175 0 -0.054 -0.196 -0.092 0.031 -0.156 0.109 -0.368 0.028 -0.072 0.145 0

14 3 0 -0.044 0 0 0.056 -0.023 0.014 -0.042 0.032 0.139 -0.088 0.081 -0.175 0 -0.054 -0.132 -0.092 0.031 -0.156 0.109 -0.368 0.028 -0.072 0.145 0

14 3 0 -0.16 0 0 0.056 -0.023 0.014 0 0.032 0.139 -0.088 0.081 -0.175 0.179 -0.054 -0.132 -0.092 0.031 -0.156 0.109 -0.136 0.028 -0.009 0.145 0

14 3 0 -0.044 0 0 0.056 -0.023 0.014 0 0.032 0.139 -0.088 0.081 -0.175 0 -0.054 -0.132 -0.092 0.031 -0.156 -0.008 -0.368 0.028 -0.072 0.145 0

14 3 0 -0.044 0 0.002 0.056 -0.023 0.014 0 0.032 0.139 -0.088 0.081 -0.175 0 -0.054 -0.132 -0.092 0.031 -0.156 0.109 -0.368 0.028 -0.072 0.145 0

14 3 0 -0.044 0 0 0.056 -0.023 0.014 0 0.032 0.139 -0.088 0.081 -0.175 0 -0.054 -0.132 -0.092 0.031 -0.156 0.109 -0.136 0.028 -0.072 -0.051 0

14 2 0 -0.044 0 0 0.056 -0.023 0.014 0 0.032 0.071 -0.088 0.081 -0.175 0 -0.054 -0.132 -0.092 0.031 -0.156 0.109 -0.136 0.028 -0.072 0.145 0

14 2 0 -0.044 0 0 0.056 -0.023 0.014 0 0.032 0.139 -0.088 0.081 -0.175 0 -0.054 -0.034 -0.092 0.031 -0.156 0.109 -0.136 0.028 -0.072 0.145 0

14 2 0 -0.044 0 0 0.056 -0.023 0.014 0 0.032 0.139 -0.088 0.081 -0.175 0 -0.054 -0.132 -0.109 0.031 -0.156 0.109 -0.368 0.028 -0.072 0.145 -0.12

14 2 0 -0.044 0 0 0.056 -0.023 0.014 0 0.032 0.139 -0.088 0.081 -0.175 0 -0.054 -0.132 -0.092 0.031 -0.172 0.109 -0.136 0.028 -0.072 0.145 0

14 2 0 -0.044 0 0 0.056 -0.023 0.014 0 0.032 0.094 -0.088 0.081 -0.175 0 -0.054 -0.132 -0.092 0.031 -0.062 0.109 -0.368 0.028 -0.072 0.145 0

14 2 0 -0.033 0 0 0.056 0.184 0.014 0 0.032 0.139 -0.088 0.081 -0.175 0.038 -0.054 -0.132 -0.092 0.031 -0.156 0.042 -0.368 0.028 -0.072 0.145 0

14 2 0 -0.044 0 0 0.056 -0.023 0.014 0 0.032 0.139 -0.088 0.277 -0.175 0 -0.054 -0.132 -0.109 0.031 -0.156 0.109 -0.368 0.028 -0.072 0.145 0

14 2 0 -0.044 0 0 0.056 -0.023 0.014 0 0.032 0.139 -0.088 0.081 -0.175 0 -0.054 -0.132 -0.092 0.031 -0.156 0.109 -0.136 0.028 -0.009 0.145 0

14 2 0 -0.033 0 -0.038 0.056 0.184 0.014 0 0.032 0.139 -0.088 0.081 -0.175 0.038 -0.054 -0.132 -0.092 0.031 -0.156 0.042 -0.368 0.028 -0.072 0.145 0

14 2 0 -0.044 0 0 0.056 -0.023 0.014 0 0.032 0.139 -0.088 0.081 -0.245 0 -0.054 -0.132 -0.092 0.031 -0.156 0.109 -0.368 0.028 -0.072 0.145 0

14 2 0.02 -0.044 0 0 0.056 -0.023 0.014 0 0.032 0.139 -0.088 0.081 -0.175 0 -0.054 -0.132 -0.092 0.031 -0.062 0.109 -0.273 0.028 -0.072 0.145 0

14 2 0 -0.044 0 0 0.056 -0.023 0.014 -0.013 0.032 0.139 -0.088 0.081 -0.175 0 -0.054 -0.132 -0.092 0.031 -0.156 0.109 -0.368 0.028 -0.072 0.145 0

14 2 0 -0.044 0 0 0.056 -0.023 0.014 0 0.032 0.139 -0.088 0.081 -0.175 0 -0.054 -0.132 -0.092 0.031 -0.156 0.109 -0.136 -0.026 -0.072 0.145 0

14 2 -0.152 -0.044 0 0 0.056 -0.023 0.014 0 0.032 0.139 -0.088 0.081 -0.175 0 -0.054 -0.132 -0.092 0.031 -0.156 0.109 -0.368 0.028 -0.072 0.145 0

14 2 0 -0.044 0 0 0.056 -0.023 0.014 0 0.032 0.139 -0.088 0.081 -0.175 0 -0.054 -0.061 -0.092 0.031 -0.062 0.109 -0.368 0.028 -0.072 0.145 0

14 2 0 -0.044 0 0 0.056 -0.023 0.014 0 0.032 0.139 -0.088 0.081 -0.175 0 -0.054 -0.132 -0.092 0.031 -0.062 0.109 -0.368 0.028 -0.017 0.145 0

14 2 0 -0.044 0 0 0.056 -0.023 0.014 0 0.032 0.139 -0.088 0.081 -0.175 0 -0.054 -0.132 -0.092 0.031 -0.062 0.109 -0.368 0.028 -0.072 0.145 0.117

14 2 0 -0.044 0 -0.005 0.056 -0.023 0.014 0 0.032 0.139 -0.088 0.081 -0.175 0 -0.054 -0.132 -0.092 0.031 -0.156 0.109 -0.136 0.028 -0.072 0.145 0

14 2 0 -0.044 -0.333 0 0.056 -0.023 0.014 0 0.032 0.139 -0.088 0.081 -0.175 0 -0.054 -0.132 -0.092 0.031 -0.156 0.109 -0.136 0.028 -0.072 0.145 0

14 2 0 -0.044 0 0 0.056 -0.023 0.014 0 0.032 0.139 -0.088 0.081 -0.175 0 -0.054 -0.132 -0.092 0.031 -0.156 0.109 -0.136 -0.03 -0.072 0.145 0

14 2 0 -0.044 0 -0.084 0.056 -0.023 0.014 0 0.032 0.139 -0.088 0.081 -0.175 0 -0.054 -0.132 -0.092 0.031 -0.156 0.109 -0.368 0.028 -0.072 0.145 0

14 2 0 -0.044 0 0 0.056 -0.023 0.014 0 0.032 0.139 -0.088 0.081 -0.175 0 -0.054 -0.132 -0.092 0.031 -0.156 0.109 0.08 0.028 -0.072 0.145 0

14 2 0 -0.012 0 0 0.056 -0.023 0.014 0 0.032 0.139 -0.088 0.081 -0.175 0 -0.054 -0.132 -0.092 0.031 -0.156 0.109 -0.368 0.028 -0.072 0.145 0

14 2 0 -0.044 0 0 0.056 -0.023 0.014 0 0.032 0.139 -0.088 0.081 -0.175 0.064 -0.054 -0.132 -0.092 0.031 -0.062 0.109 -0.368 0.028 -0.072 0.145 0

14 2 0 -0.044 0 0 0.056 -0.023 0.014 0.053 0.032 0.139 -0.088 0.081 -0.175 0 -0.054 -0.132 -0.092 0.031 -0.156 0.109 -0.136 0.028 -0.072 0.145 0

14 1 -0.099 -0.044 0 0 0.056 -0.023 0.014 0 0.032 0.139 -0.088 0.064 -0.175 0 -0.054 -0.132 -0.092 0.031 -0.156 0.109 -0.368 0.028 -0.072 0.145 0

14 1 0 -0.044 0 0 0.056 -0.023 0.014 0.143 0.032 0.139 -0.088 0.081 -0.175 0 -0.054 -0.132 -0.092 0.031 -0.156 0.109 -0.136 0.028 -0.072 0.145 0

14 1 0 -0.044 0 0 0.056 -0.023 0.014 0 0.032 0.139 -0.088 0.081 -0.175 0 -0.054 -0.208 -0.092 0.031 -0.156 0.109 -0.136 0.028 -0.072 0.145 0

14 1 0 -0.044 0 0 0.056 -0.023 0.014 0 0.032 0.139 -0.088 0.081 -0.175 0 -0.054 -0.132 -0.092 0.031 -0.103 0.109 -0.136 0.028 -0.072 0.145 0

14 1 0 -0.044 0 0 0.056 -0.023 0.014 0 0.032 0.139 -0.088 0.081 -0.175 0 -0.054 -0.132 -0.092 0.031 -0.161 0.042 -0.368 0.028 -0.072 0.145 0

14 1 0 -0.044 0 0 0.056 -0.023 0.014 0.066 0.032 0.139 -0.088 0.081 -0.175 0 -0.054 -0.132 -0.092 0.031 -0.156 0.109 -0.136 0.028 -0.072 0.145 0

14 1 0 -0.044 0 0 0.056 -0.023 -0.065 0 0.032 0.139 -0.088 0.081 -0.175 0 -0.054 -0.132 -0.092 0.031 -0.156 0.109 -0.136 0.028 -0.072 0.145 0

14 1 0 -0.044 0 0 0.076 -0.023 0.014 0 0.032 0.139 -0.088 0.081 -0.175 0 -0.054 -0.132 -0.092 0.031 -0.156 0.109 -0.136 0.028 -0.072 0.145 0

14 1 -0.028 -0.044 0 0 0.056 -0.023 0.014 0 0.032 0.133 -0.088 0.081 -0.175 0 -0.054 -0.132 -0.109 0.031 -0.156 0.109 -0.368 0.028 -0.072 0.145 0

14 1 0 -0.044 0 0 0.056 -0.023 0.014 0 0.032 0.139 -0.088 0.081 -0.175 0 -0.054 -0.132 -0.092 0.031 -0.156 0.109 -0.438 0.028 -0.072 0.145 0

14 1 0 -0.044 0 0 0.056 -0.023 0.014 0 0.032 0.139 -0.088 0.081 -0.175 0 -0.054 -0.132 -0.092 0.031 -0.156 0.042 -0.368 0.028 -0.072 0.145 -0.07

14 1 0 0.114 0 0 0.056 -0.023 0.014 0 0.032 0.139 -0.088 0.081 -0.175 0 -0.054 -0.196 -0.092 0.031 -0.156 0.109 -0.368 0.028 -0.072 0.145 0

14 1 0 -0.044 0 0 0.056 -0.023 0.014 0 0.032 0.139 -0.088 0.081 -0.175 0.113 -0.054 -0.132 -0.092 0.031 -0.156 0.109 -0.136 0.028 -0.072 0.145 0

14 1 0 -0.044 0 0 0.056 -0.023 0.014 0 0.032 0.139 -0.088 0.081 -0.175 0 -0.054 -0.132 -0.092 0.031 -0.156 0.109 -0.136 0.212 -0.072 0.145 0

14 1 0 -0.044 0 0 0.056 -0.023 0.014 0 0.032 0.139 -0.088 0.081 -0.175 0 -0.054 -0.132 -0.092 0.031 -0.156 0.109 -0.368 0.028 -0.069 0.145 0

14 1 0 -0.044 0 0 0.056 -0.023 0.014 0 0.032 0.139 -0.088 0.081 -0.219 0 -0.054 -0.132 -0.109 0.031 -0.156 0.109 -0.368 0.028 -0.072 0.145 0

14 1 0 -0.044 0 0 0.056 -0.023 0.014 0 0.032 0.139 -0.088 0.081 -0.336 0 -0.054 -0.132 -0.092 0.031 -0.156 0.109 -0.136 0.028 -0.072 0.145 0

14 1 0 0.012 0 0 0.056 -0.023 0.014 0 0.032 0.139 -0.088 0.081 -0.175 0 -0.054 -0.196 -0.092 0.031 -0.156 0.109 -0.368 0.028 -0.072 0.145 0

14 1 0 -0.044 0 0 0.056 -0.023 0.014 0 0.032 0.139 -0.088 0.081 -0.175 0 -0.054 -0.132 -0.092 0.031 -0.062 0.109 -0.331 0.028 -0.072 0.145 0

14 1 0 -0.044 0 0 0.056 -0.023 0.014 0 0.032 0.139 -0.088 0.081 -0.175 0 -0.054 -0.132 -0.092 0.031 -0.156 0.109 -0.136 0.028 0.076 0.145 0

14 1 0 -0.044 0 0 0.056 -0.023 0.014 0 0.032 0.139 -0.088 0.081 -0.175 0 -0.054 -0.132 -0.092 0.031 -0.156 0.109 -0.368 0.028 0.001 0.145 0

14 1 0 -0.034 0.072 0 0.056 -0.023 0.014 0 0.032 0.139 -0.088 0.081 -0.175 0 -0.054 -0.196 -0.092 0.031 -0.156 0.109 -0.368 0.028 -0.072 0.145 0

14 1 0 -0.167 0 0 0.056 -0.023 0.018 0 0.032 0.139 -0.088 0.081 -0.175 0 -0.054 -0.132 -0.092 0.031 -0.062 0.109 -0.368 0.028 -0.072 0.145 0

14 1 0 -0.044 0 0 0.056 -0.023 0.014 0.127 0.032 0.139 -0.088 0.081 -0.175 0 -0.054 -0.132 -0.092 0.031 -0.156 0.109 -0.368 0.028 -0.072 0.145 0

14 1 0 -0.044 0 0 0.056 -0.023 0.014 0 0.032 0.139 -0.088 0.081 -0.175 0 -0.054 -0.132 -0.092 0.031 -0.156 0.109 -0.136 0.028 -0.072 0.145 -0.076

14 1 0 -0.044 0 0 0.056 -0.023 0.014 -0.194 0.032 0.139 -0.088 0.081 -0.175 0 -0.054 -0.132 -0.092 0.031 -0.156 0.109 -0.232 0.028 -0.072 0.145 0

14 1 0 -0.044 0 0 0.056 -0.023 0.014 0.144 0.032 0.139 -0.088 0.081 -0.175 0 -0.054 -0.132 -0.092 0.031 -0.156 0.109 -0.368 0.028 -0.069 0.145 0

14 1 0 -0.044 0 0 0.056 -0.023 0.014 0.05 0.032 0.139 -0.088 0.081 -0.175 0 -0.054 -0.132 -0.092 0.031 -0.156 0.109 -0.368 0.028 -0.072 0.145 0

14 1 0 -0.044 0 0 0.056 -0.023 0.014 0 0.032 0.139 -0.088 0.081 -0.175 0 -0.054 -0.132 -0.092 0.031 -0.102 0.109 -0.136 0.028 -0.072 0.145 0

14 1 0 -0.044 0 0 0.215 -0.023 0.014 0 0.032 0.139 -0.088 0.081 -0.175 0 -0.054 -0.132 -0.092 0.031 -0.156 0.109 -0.368 0.028 -0.072 0.145 0

14 1 0 -0.044 0 0 0.056 -0.023 0.014 0 0.032 0.308 -0.088 0.081 -0.175 0 -0.054 -0.132 -0.092 0.031 -0.156 0.042 -0.368 0.028 -0.072 0.145 0

14 1 0 -0.044 0 0 0.056 0.017 0.014 0 0.032 0.139 -0.088 0.081 -0.175 0 -0.054 -0.132 -0.092 0.031 -0.156 0.109 -0.368 0.028 -0.072 0.145 0

14 1 0 -0.044 0 0 0.056 -0.023 0.014 0 0.032 0.139 -0.088 0.081 -0.175 0 -0.054 -0.132 -0.092 0.031 -0.156 0.109 -0.136 0.028 -0.072 0.064 0

14 1 0 -0.044 0 0 0.056 -0.023 0.014 0 0.032 0.059 -0.088 0.081 -0.175 0 -0.054 -0.132 -0.092 0.031 -0.062 0.109 -0.368 0.028 -0.072 0.145 0

14 1 -0.028 -0.044 0 0 0.056 -0.023 0.014 0 0.032 0.139 -0.088 0.081 -0.175 0 -0.054 -0.132 -0.109 0.031 -0.18 0.109 -0.368 0.028 -0.072 0.145 0

14 1 0 -0.044 0 0 0.056 -0.023 0.014 0 0.032 0.139 -0.088 0.081 -0.175 -0.109 -0.054 -0.132 -0.092 0.031 -0.156 0.109 -0.136 0.028 -0.072 0.145 0

14 1 0 -0.044 0 0 0.056 -0.023 0.014 0 0.032 0.139 -0.088 0.081 -0.175 0 -0.054 -0.059 -0.092 0.031 -0.156 0.109 -0.136 0.028 -0.072 0.145 0

14 1 -0.028 -0.044 0 0 0.056 -0.023 0.014 0 0.032 0.139 -0.088 0.081 -0.175 0 -0.054 -0.132 -0.109 0.031 -0.156 0.109 -0.368 0.028 -0.24 0.145 0

14 1 0 -0.044 0 0 0.056 -0.023 0.014 0 0.032 0.139 -0.088 0.081 -0.175 0 -0.054 -0.201 -0.092 0.031 -0.156 0.109 -0.136 0.028 -0.072 0.145 0

14 1 0 -0.044 0.016 0 0.056 -0.023 0.014 0 0.032 0.139 -0.088 0.081 -0.175 0 -0.054 -0.132 -0.092 0.031 -0.062 0.109 -0.368 0.028 -0.072 0.145 0

14 1 0 -0.044 0 0 0.056 -0.023 0.014 0 0.032 0.139 -0.088 0.081 0.048 0 -0.054 -0.132 -0.092 0.031 -0.156 0.109 -0.136 0.028 -0.072 0.145 0

14 1 0 -0.044 0 0 0.056 -0.023 0.014 0 0.032 0.139 -0.088 0.081 -0.175 0 -0.054 -0.132 -0.092 0.126 -0.062 0.109 -0.368 0.028 -0.072 0.145 0

14 1 0 -0.044 0 0 0.056 -0.023 0.014 0 0.032 0.139 -0.088 0.081 -0.222 0 -0.054 -0.132 -0.092 0.031 -0.156 0.109 -0.368 0.028 -0.072 0.145 0

14 1 0 -0.044 0 0 0.056 -0.023 0.014 0 0.032 0.139 -0.088 0.081 -0.175 0 -0.054 -0.132 -0.092 0.031 -0.156 0.109 -0.008 0.028 -0.072 0.145 0

14 1 0 -0.044 0 0 0.056 -0.023 0.014 0 0.032 0.139 -0.088 0.081 -0.175 0 -0.054 -0.132 -0.092 0.031 -0.062 0.109 -0.368 0.028 -0.002 0.145 0

14 1 0 -0.044 0 0 0.056 -0.023 0.014 0 0.032 0.144 -0.088 0.081 -0.175 0 -0.054 -0.132 -0.109 0.031 -0.156 0.109 -0.368 0.028 -0.072 0.145 0

14 1 0 -0.044 0 0 0.056 -0.023 0.014 0 0.032 0.139 -0.088 0.081 -0.417 0 -0.054 -0.132 -0.092 0.031 -0.062 0.109 -0.368 0.028 -0.072 0.145 0

14 1 0 -0.044 0 0 0.056 -0.023 0.014 0 0.032 0.139 -0.088 0.081 -0.175 0 -0.054 -0.132 -0.092 0.031 -0.062 0.109 -0.368 0.028 -0.072 0.145 -0.078

14 1 0.056 -0.044 0 0 0.056 -0.023 0.014 0 0.032 0.139 -0.088 0.081 -0.175 0 -0.054 -0.132 -0.092 0.031 -0.156 0.109 -0.136 0.028 -0.072 0.145 0

14 1 0 -0.044 0 0 0.056 -0.023 0.014 -0.001 0.032 0.139 -0.088 0.098 -0.175 0 -0.054 -0.132 -0.109 0.031 -0.156 0.109 -0.368 0.028 -0.072 0.145 0

15 694 0.057 0 -0.039 0 0.068 0 -0.074 -0.113 0 0.251 -0.07 0.135 -0.225 0 -0.287 -0.01 -0.019 0.017 0 0 0.011 0.004 0 0 0

15 431 0.057 0 -0.039 0 0.068 0 -0.074 -0.113 0 0.251 -0.07 0.135 -0.225 0 -0.287 -0.01 -0.019 0.017 0 -0.08 0.011 0.004 0 0 0

15 389 0.057 -0.009 -0.039 0 0.068 0 -0.074 -0.187 0 0.251 0 0.135 -0.225 0 -0.287 -0.01 -0.019 0.017 0 0 0.128 0.004 0 0 0.001

15 329 0.057 -0.009 -0.039 0 0.068 0 -0.074 -0.187 0 0.251 0 0.135 -0.225 0 -0.287 -0.01 -0.019 0.017 0 0 0.128 0.004 0 0.008 0.001

15 189 0.057 -0.009 -0.039 0 0.068 0 -0.074 -0.187 0 0.251 0 0.135 -0.225 0 -0.26 -0.01 -0.019 0.017 0 0 0.128 0.004 0 0 0.001

15 164 0.057 -0.009 -0.039 0 0.068 0 -0.074 -0.092 0 0.251 0 0.135 -0.225 0 -0.21 -0.01 -0.019 0.017 0 0 0.128 0.004 0 0 0

15 143 0.057 -0.009 -0.039 0 0.068 0 -0.074 -0.187 0 0.085 0 0.135 -0.225 0 -0.287 -0.01 -0.019 0.017 0 0 0.128 0.004 0 0 0.001

15 115 0.057 0 -0.039 0 0.068 0 -0.074 -0.113 0 0.251 -0.07 0.135 -0.225 0.051 -0.287 -0.01 -0.019 0.017 0 0 0.011 0.004 0 0 0

15 113 0.057 0 -0.039 0 0.068 0 -0.074 -0.113 0 0.251 -0.07 0.135 -0.225 0 -0.287 -0.01 -0.019 0.021 0 0 0.011 0.004 0 0 0

15 110 0.057 0 -0.039 0 0.068 0 -0.074 -0.113 0 0.251 -0.133 0.135 -0.225 0 -0.287 -0.01 -0.019 0.017 0 -0.08 0.011 0.004 0 0 0

15 100 0.057 -0.009 -0.039 0 0.068 -0.089 -0.074 -0.187 0 0.251 0 0.135 -0.225 0.138 -0.287 -0.01 -0.019 0.017 0 0 0.128 0.004 0 0 0.001

15 94 0.057 0 -0.039 0 0.068 0 -0.074 -0.113 0 0.251 -0.07 0.135 -0.225 0.004 -0.287 -0.01 -0.019 0.017 0 -0.08 0.011 0.004 0 0 0

15 91 0.057 0 -0.039 0 0.068 0 -0.074 -0.113 0 0.251 -0.07 0.135 -0.225 0 -0.419 -0.01 -0.019 0.017 0 0 0.011 0.004 0 0 0

15 70 0.057 0 -0.039 0 0.068 0 -0.074 -0.113 0 0.394 -0.07 0.135 -0.225 0 -0.287 -0.01 -0.019 0.017 0 0 0.011 0.004 0 0 0

15 65 0.057 -0.009 -0.039 0.097 0.068 0 -0.074 -0.092 0 0.251 0 0.21 -0.225 0 -0.287 -0.01 -0.019 0.017 0 0 0.128 0.004 0 0 0

15 56 0.057 -0.009 -0.039 0 0.068 0.009 -0.074 -0.187 0 0.251 0 0.135 -0.225 0.138 -0.287 -0.01 -0.019 0.017 0 0 0.128 0.004 0 0 0.001

15 53 0.057 -0.009 -0.039 0 0.068 0 -0.074 -0.187 0 0.251 0 0.135 -0.225 0 -0.287 -0.01 -0.019 0.017 0 0 0.128 0.004 0.183 0 0.001

15 41 0.057 -0.009 -0.039 0 0.068 0 -0.074 -0.187 0 0.251 0 0.135 -0.225 0 -0.364 -0.01 -0.019 0.017 0 0 0.128 0.004 0 0 0.001

15 36 0.057 0 -0.039 0 0.068 0 -0.074 -0.113 -0.032 0.251 -0.07 0.135 -0.225 0.004 -0.287 -0.01 -0.019 0.017 0 -0.08 0.011 0.004 0 0 0

15 36 0.057 -0.009 -0.039 0 0.068 0 -0.074 -0.187 0 0.251 0 0.147 -0.225 0 -0.287 -0.01 -0.019 0.017 0 0 0.128 0.004 0.183 0 0.001

15 30 0.057 0 -0.039 0 0.068 0 -0.074 -0.113 0 0.139 -0.133 0.135 -0.225 0 -0.287 -0.01 -0.019 0.017 0 -0.08 0.011 0.004 0 0 0

15 30 0.057 0 -0.039 0 0.068 0 -0.074 -0.113 0 0.251 -0.07 0.135 -0.225 0 -0.419 -0.01 -0.019 0.017 0 0 -0.03 0.004 0 0 0

15 30 0.057 0 -0.039 0 0.068 0 -0.074 -0.113 0 0.251 -0.07 0.135 -0.225 0 -0.287 0.104 -0.019 0.021 0 0 0.011 0.004 0 0 0

15 25 0.057 0 -0.039 0 0.068 0 -0.074 -0.113 0 0.394 -0.07 0.091 -0.225 0 -0.287 -0.01 -0.019 0.017 0 0 0.011 0.004 0 0 0

15 24 0.057 0 -0.039 0 0.068 0 -0.074 -0.113 0 0.251 -0.07 0.135 -0.225 0 -0.287 -0.01 -0.019 0.017 0 0 0.011 -0.09 0 0 0

15 23 0.057 0 -0.039 0 0.068 0 -0.074 -0.113 0 0.251 -0.07 0.135 -0.225 0.004 -0.287 -0.01 -0.019 0.017 0 -0.08 0.011 0.004 0 0 0.097

15 20 0.057 0 -0.039 0 0.068 0 -0.074 -0.113 0 0.251 -0.07 0.135 -0.225 0 -0.287 -0.01 -0.019 0.017 0 0 0.011 -0.057 0 0 0

15 25 0.057 0 -0.039 0 0.068 0 -0.074 -0.113 0 0.251 -0.07 0.135 -0.225 0 -0.287 -0.01 -0.019 0.017 0 -0.158 0.011 0.004 0 0 0

15 17 0.057 -0.009 -0.039 0 0.068 0.009 -0.074 -0.187 0 0.251 0 0.135 -0.225 0.138 -0.287 -0.01 -0.019 0.017 0 0 0.128 0.004 0 0.056 0.001

15 15 0.057 -0.009 -0.039 0 -0.084 0 -0.074 -0.187 0 0.251 0 0.135 -0.225 0 -0.287 -0.01 -0.019 0.017 0 0 0.128 0.004 0 0 0.001

15 12 0.057 0 -0.039 0 0.068 0 -0.074 -0.113 -0.022 0.251 -0.07 0.135 -0.225 0 -0.287 -0.01 -0.019 0.017 0 0 0.011 0.004 0 0 0

15 12 0.057 0 -0.039 0 0.068 0 -0.074 -0.113 0 0.251 -0.133 0.135 -0.193 0 -0.287 -0.01 -0.019 0.017 0 -0.08 0.011 0.004 0 0 0

15 11 0.057 0 -0.039 0 0.068 0 -0.074 -0.113 0 0.251 -0.07 0.135 -0.225 0 -0.207 -0.01 -0.019 0.017 0 -0.08 0.011 0.004 0 0 0

15 10 0.057 -0.009 -0.039 0 0.068 0 -0.074 -0.092 0 0.385 0 0.135 -0.225 0 -0.21 -0.01 -0.019 0.017 0 0 0.128 0.004 0 0 0

15 10 0.057 0.007 -0.039 0.097 0.068 0 -0.074 -0.092 0 0.251 0 0.21 -0.225 0 -0.287 -0.01 -0.019 0.017 0 0 0.128 0.004 0 0 0

15 9 0.057 -0.009 -0.039 0 0.068 0 -0.074 -0.187 0 0.251 0 0.135 -0.225 0 -0.311 -0.01 -0.019 0.017 0 0 0.128 0.004 0.183 0 0.001

15 8 0.057 0 -0.039 0 0.068 0 -0.074 -0.113 0 0.251 -0.07 0.001 -0.225 0 -0.287 -0.01 -0.019 0.017 0 -0.08 0.011 0.004 0 0 0

15 8 0.057 0 -0.039 0 0.068 0 -0.074 -0.113 0 0.251 -0.07 0.135 -0.225 0 -0.287 -0.01 -0.019 0.017 0 -0.08 0.011 0.004 0.001 0 0

15 7 0.057 -0.009 -0.039 0 0.068 0 -0.074 -0.092 0 0.251 0 0.135 -0.385 0 -0.276 -0.01 -0.019 0.017 0 0 0.128 0.004 0 0 0

15 7 0.057 -0.009 -0.039 0 0.068 -0.089 -0.074 -0.187 0 0.251 0 0.135 -0.225 0.123 -0.287 -0.01 -0.019 0.017 0 0 0.128 0.004 0 0 0.001

15 7 0.057 0 -0.039 -0.021 0.068 0 -0.074 -0.113 0 0.251 -0.07 0.135 -0.225 0 -0.287 -0.01 -0.019 0.017 0 -0.08 0.011 0.004 0 0 0

15 7 0.057 0 -0.039 0 0.068 0 -0.074 -0.113 0 0.251 -0.07 0.135 -0.225 0 -0.287 -0.01 -0.019 0.021 0 0 -0.117 0.004 0 0 0

15 6 0.057 -0.009 -0.039 0 0.068 0 -0.074 -0.187 0 0.251 0 0.135 -0.225 0 -0.287 -0.01 -0.019 0.017 0 0 0.128 0.004 -0.078 0 0.001

15 6 0.057 -0.009 -0.039 0 0.068 0 -0.074 -0.187 0 0.251 0.043 0.135 -0.225 0 -0.287 -0.01 -0.019 0.017 0 0 0.128 0.004 0 0.008 0.001

15 6 0.057 -0.009 -0.039 0 0.068 0 -0.074 -0.187 0 0.325 0 0.135 -0.225 0 -0.287 -0.01 -0.019 0.017 0 0 0.128 0.004 0 0.008 0.001

15 5 0.057 0 -0.039 0 0.068 0 -0.074 -0.113 0 0.251 0.064 0.135 -0.225 0 -0.287 -0.01 -0.019 0.017 0 0 0.011 0.004 0 0 0

15 5 0.057 -0.009 -0.039 0 0.068 0 -0.074 -0.187 0 0.251 0 0.135 -0.225 0 -0.287 -0.01 -0.019 -0.073 0 0 0.128 0.004 0 0.008 0.001

15 5 -0.032 -0.009 -0.039 0 0.068 0 -0.074 -0.187 0 0.251 0 0.135 -0.225 0 -0.287 -0.01 -0.019 0.017 0 0 0.128 0.004 0 0.008 0.001

15 4 0.057 -0.009 -0.039 0 0.068 0 -0.074 -0.092 0 0.385 0 0.135 -0.225 0 -0.21 -0.01 0.011 0.017 0 0 0.128 0.004 0 0 0

15 4 0.057 -0.009 -0.039 0.099 0.068 0 -0.074 -0.187 0 0.085 0 0.135 -0.225 0 -0.287 -0.01 -0.019 0.017 0 0 0.128 0.004 0 0 0.001

15 4 0.057 0 -0.039 0 0.068 0 -0.074 -0.113 -0.113 0.251 -0.07 0.135 -0.225 0 -0.287 -0.01 -0.019 0.017 0 0 0.011 0.004 0 0 0

15 4 0.057 -0.009 -0.039 0 0.068 0 -0.074 -0.187 0 0.251 0 0.135 -0.161 0 -0.287 -0.01 -0.019 0.017 0 0 0.363 0.004 0 0 0.001

15 4 0.057 -0.009 -0.039 0 0.068 0 -0.074 -0.187 0 0.085 0 0.135 -0.225 0 -0.287 -0.01 -0.019 0.017 0 -0.014 0.128 0.004 0 0 0.001

15 4 0.057 0 -0.039 -0.037 0.068 0 -0.074 -0.113 0 0.251 -0.07 0.135 -0.225 0 -0.287 -0.01 -0.019 0.017 0 0 0.011 0.004 0 0 0

15 4 0.057 -0.009 -0.039 0 0.065 0 -0.074 -0.187 0 0.251 0 0.135 -0.225 0 -0.287 -0.01 -0.019 0.017 0 0 0.128 0.004 0 0.008 0.001

15 4 0.057 -0.009 -0.039 0 0.068 0 -0.074 -0.187 0 0.251 0 0.135 -0.387 0 -0.287 -0.01 -0.019 0.017 0 0 0.128 0.004 0.183 0 0.001

15 3 0.057 0 -0.039 0 0.068 0 -0.074 -0.113 0 0.251 -0.07 0.135 -0.225 0 -0.287 -0.01 -0.019 0.017 0 0 0.011 0.004 0 -0.039 0

15 3 0.057 0 -0.039 0 0.068 0 -0.074 -0.113 0 0.251 -0.07 0.135 -0.225 0 -0.287 -0.01 -0.019 0.048 0 -0.08 0.011 0.004 0 0 0

15 3 0.057 0 -0.039 0 0.068 0 -0.074 -0.113 0 0.251 0.064 0.135 -0.225 0 -0.287 -0.01 -0.019 0.017 0 0 0.067 0.004 0 0 0

15 3 0.057 0 -0.039 0 0.068 0 -0.074 -0.113 0 0.251 -0.07 0.135 -0.329 0 -0.287 -0.01 -0.019 0.017 0 -0.08 0.011 0.004 0 0 0

15 3 0.057 0 -0.039 0 0.068 0 -0.074 -0.113 0 0.251 -0.07 0.135 -0.225 0 -0.287 -0.028 -0.019 0.021 0 0 0.011 0.004 0 0 0

15 3 0.057 -0.009 -0.039 0 0.068 0 -0.074 -0.092 0 0.251 0 0.135 -0.225 0 -0.337 -0.01 -0.019 0.017 0 0 0.128 0.004 0 0 0

15 3 0.057 0 -0.039 0 0.068 0 -0.074 -0.113 0 0.251 -0.07 0.135 -0.225 -0.026 -0.287 -0.01 -0.019 0.017 0 -0.08 0.011 0.004 0 0 0

15 3 0.057 0 -0.039 0 0.054 0 -0.074 -0.113 0 0.251 -0.07 0.135 -0.225 0 -0.287 -0.01 -0.019 0.017 0 0 0.011 0.004 0 0 0

15 2 0.057 -0.009 -0.039 0 0.068 0 0.063 -0.187 0 0.085 0 0.135 -0.225 0 -0.287 -0.01 -0.019 0.017 0 0 0.128 0.004 0 0 0.001

15 2 0.057 0 -0.039 0 0.068 0 -0.074 -0.113 0 0.251 -0.07 0.223 -0.225 0 -0.419 -0.01 -0.019 0.017 0 0 0.011 0.004 0 0 0

15 2 0.057 0 -0.039 0 0.068 0 -0.074 -0.113 0 0.251 -0.07 0.135 -0.225 0 -0.287 -0.01 -0.019 0.017 0 -0.08 0.011 0.004 0.066 0 0

15 2 0.057 -0.009 -0.039 0 0.068 0 -0.074 -0.187 0 0.251 0 0.135 -0.225 0 -0.287 -0.01 -0.019 0.017 0 0 0.128 0.004 0.183 0.061 0.001

15 2 0.057 0 -0.039 0 0.068 0 -0.074 -0.113 -0.022 0.251 -0.07 0.135 -0.225 0 -0.287 -0.01 -0.019 0.143 0 0 0.011 0.004 0 0 0

15 2 0.057 -0.009 -0.039 0 0.068 0 -0.167 -0.092 0 0.251 0 0.135 -0.225 0 -0.21 -0.01 -0.019 0.017 0 0 0.128 0.004 0 0 0

15 2 0.057 -0.009 -0.039 0 0.068 0 -0.074 -0.187 0 0.251 0 0.135 -0.225 0 -0.364 -0.01 -0.019 0.017 0 0 0.128 0.004 0 0 0.203

15 2 0.057 0.117 -0.039 0 0.068 0 -0.074 -0.113 0 0.251 -0.07 0.223 -0.225 0 -0.419 -0.01 -0.019 0.017 0 0 0.011 0.004 0 0 0

15 2 0.063 0 -0.039 0 0.068 0 -0.074 -0.113 0 0.251 -0.07 0.135 -0.225 0 -0.287 -0.01 -0.019 0.017 0 -0.08 0.011 0.004 0 0 0

15 2 0.057 -0.009 -0.039 0 0.068 0 -0.074 -0.187 0 0.251 0 0.135 -0.225 0 -0.26 -0.01 -0.019 0.017 0 0 0.128 0.004 0 0.141 0.001

15 2 0.057 0 -0.039 0 0.068 0 -0.074 -0.113 0 0.251 -0.133 0.135 -0.225 0 -0.287 -0.01 -0.019 0.017 0 -0.08 -0.096 0.004 0 0 0

15 2 0.057 -0.009 -0.039 0 0.068 0 -0.074 -0.092 0 0.251 0 0.135 -0.225 0 -0.21 -0.01 -0.019 0.017 0 0 0.128 -0.103 0 0 0

15 2 0.057 0 -0.039 0 0.068 0 -0.074 -0.113 0 0.251 -0.07 0.135 -0.225 0 -0.287 -0.01 -0.019 0.017 0 -0.08 0.011 0.004 0 0.009 0

15 2 0.057 -0.009 -0.039 0 0.068 0 -0.074 -0.187 0 0.251 0 0.135 -0.225 0 -0.287 -0.01 -0.019 0.017 0 0 0.128 0.004 0.01 0.008 0.001

15 2 0.057 0 -0.039 0 0.068 0 -0.074 -0.113 0 0.251 -0.07 0.135 -0.225 0 -0.287 0.004 -0.019 0.017 0 0 0.011 0.004 0 0 0

15 2 0.057 -0.009 -0.039 0 0.068 0 -0.074 -0.123 0 0.251 0 0.135 -0.225 0 -0.287 -0.01 -0.019 0.017 0 0 0.128 0.004 0 0 0.001

15 2 0.057 -0.009 -0.039 0 0.068 0 -0.074 -0.187 0 0.251 0 0.135 -0.225 0.073 -0.26 -0.01 -0.019 0.017 0 0 0.128 0.004 0 0 0.001

15 2 0.057 -0.009 -0.039 0 0.068 0 -0.074 -0.092 0 0.251 0 0.135 -0.225 0 -0.21 -0.01 -0.019 0.017 0 0 0.128 0.004 0 0 0.124

15 2 0.057 -0.176 -0.039 0 0.068 0 -0.074 -0.113 0 0.251 -0.07 0.135 -0.225 0 -0.287 -0.01 -0.019 0.017 0 -0.08 0.011 0.004 0 0 0

15 2 0.057 -0.009 -0.039 0 0.068 0 -0.074 -0.128 0 0.085 0 0.135 -0.225 0 -0.287 -0.01 -0.019 0.017 0 0 0.128 0.004 0 0 0.001

15 2 0.057 0 -0.039 0 0.068 -0.016 -0.074 -0.113 0 0.251 -0.07 0.135 -0.225 0 -0.287 -0.01 -0.019 0.017 0 -0.08 0.011 0.004 0 0 0

15 2 0.057 -0.021 -0.039 0 0.068 0 -0.074 -0.187 0 0.251 0 0.135 -0.225 0 -0.287 -0.01 -0.019 0.017 0 0 0.128 0.004 0 0 0.001

15 2 0.057 -0.009 -0.039 0 0.068 0 -0.074 -0.187 0 0.251 0 0.135 -0.225 0 -0.384 -0.01 -0.019 0.017 0 0 0.128 0.004 0 0 0.001

15 2 0.057 0 -0.039 0 0.068 0 -0.074 -0.113 -0.06 0.251 -0.07 0.135 -0.225 0 -0.287 -0.01 -0.019 0.017 0 -0.08 0.011 0.004 0 0 0

15 2 0.057 0 -0.039 0 0.068 0 -0.074 -0.113 0 0.251 -0.07 0.135 -0.225 0 -0.287 -0.01 -0.019 0.017 0 0 0.011 -0.041 0 0 0

15 2 0.057 -0.009 -0.039 0 0.068 0 -0.074 -0.187 0 0.251 0 0.135 -0.225 0 -0.287 -0.01 -0.019 0.017 -0.109 0 0.128 0.004 0 0 0.001

15 2 0.057 -0.009 -0.039 0 0.068 0 -0.074 -0.187 0 0.251 0 0.135 -0.225 -0.028 -0.287 -0.01 -0.019 0.017 0 0 0.128 0.004 0 0 0.001

15 2 0.057 0 -0.039 0 0.016 0 -0.074 -0.113 0 0.251 -0.07 0.135 -0.225 0 -0.287 -0.01 -0.019 0.017 0 0 0.011 0.004 0 0 0

15 2 0.057 -0.009 -0.039 0 0.068 0 -0.074 -0.187 0 0.251 0 0.135 -0.225 0 -0.287 -0.01 -0.019 0.017 0 0 0.128 0.004 -0.114 0.008 0.001

15 2 0.057 -0.009 -0.039 0 0.068 0 -0.074 -0.187 0 0.251 0 0.135 -0.225 0 -0.287 -0.01 -0.035 0.017 0 0 0.128 0.004 0 0.008 0.001

15 2 0.057 -0.009 -0.039 -0.065 0.068 0 -0.074 -0.187 0 0.251 0 0.135 -0.225 0 -0.287 -0.01 -0.019 0.017 0 0 0.128 0.004 0 0 0.001

15 1 0.057 -0.009 0.027 0 0.068 0 -0.074 -0.187 0 0.085 0 0.135 -0.225 0 -0.287 -0.01 -0.019 0.017 0 0 0.128 0.004 0 0 0.001

15 1 0.057 0 -0.039 0 0.068 0 -0.074 -0.113 0.105 0.251 -0.07 0.135 -0.225 0 -0.287 -0.01 -0.019 0.017 0 0 0.011 -0.057 0 0 0

15 1 0.057 0 -0.039 0 0.068 0 -0.074 -0.113 0 0.251 -0.162 0.135 -0.225 0 -0.287 -0.01 -0.019 0.017 0 0 0.011 -0.09 0 0 0

15 1 0.057 -0.009 -0.039 0 0.068 0 -0.074 -0.187 0.104 0.251 0 0.135 -0.161 0 -0.287 -0.01 -0.019 0.017 0 0 0.363 0.004 0 0 0.001

15 1 0.057 0 -0.039 0 0.068 0 -0.074 -0.113 0 0.251 -0.07 0.135 -0.225 0 -0.419 -0.01 -0.019 0.017 0 0.039 0.011 0.004 0 0 0

15 1 0.057 0 -0.039 0 0.068 0 -0.074 -0.113 0.024 0.251 -0.07 0.135 -0.225 0 -0.419 -0.01 -0.019 0.017 0 0 0.011 0.004 0 0 0

15 1 0.057 -0.009 0.108 0 0.068 0 -0.074 -0.187 0 0.251 0 0.135 -0.225 0 -0.287 -0.01 -0.019 0.017 0 0 0.128 0.004 0 0.008 0.001

15 1 0.057 -0.009 -0.039 0 0.068 -0.089 -0.074 -0.187 0 0.251 0 0.135 -0.225 0.138 -0.287 -0.01 -0.019 0.017 0 0 0.128 0.004 0 0 -0.179

15 1 0.057 0 -0.039 0 0.068 0 -0.074 -0.113 0 0.251 0.064 0.135 -0.225 0 -0.287 -0.01 -0.019 0.017 0 0 0.011 0.004 0 -0.015 0

15 1 0.057 0 -0.039 0 0.068 0 -0.074 -0.113 0 0.251 -0.07 0.135 -0.225 0 -0.287 -0.01 -0.019 0.017 0 -0.172 0.011 0.004 0 0 0

15 1 0.057 -0.009 -0.039 0 0.068 0 -0.074 -0.187 0 0.251 0 0.135 -0.225 0 -0.364 -0.01 -0.019 0.017 0 0 0.128 -0.083 0 0 0.001

15 1 0.057 -0.009 -0.039 0 0.068 0 -0.074 -0.092 0 0.251 0 0.334 -0.225 0 -0.21 -0.01 -0.019 0.017 0 0 0.128 0.004 0 0 0

15 1 0.057 0 -0.039 0 0.068 0 -0.074 -0.113 0 0.251 -0.07 0.135 -0.314 0 -0.287 -0.01 -0.019 0.017 0 -0.08 0.011 0.004 0 0 0

15 1 0.057 -0.009 -0.039 0 0.068 0 -0.074 -0.187 0 0.251 0 0.135 -0.225 0 -0.26 -0.01 -0.019 0.017 0 0 0.315 0.004 0 0 0.001

15 1 0.057 0 -0.039 0 0.068 0 -0.074 -0.113 0 0.251 -0.07 0.135 -0.225 0 -0.287 -0.01 -0.033 0.017 0 0 0.011 0.004 0 0 0

15 1 0.057 -0.009 -0.039 0 0.068 0 -0.074 -0.092 0 0.251 0 0.135 -0.385 -0.057 -0.276 -0.01 -0.019 0.017 0 0 0.128 0.004 0 0 0

15 1 0.057 0 -0.039 0 0.068 0 -0.074 -0.113 0 0.251 -0.07 0.135 -0.225 0 -0.419 -0.01 -0.019 0.017 0 0 0.011 0.004 0 0 -0.025

15 1 0.057 0 -0.039 0 0.068 0 -0.074 -0.113 0 0.251 -0.07 0.135 -0.225 0 -0.287 -0.01 -0.019 0.017 0 0 0.011 0.004 0 0 -0.06

15 1 0.057 0 -0.039 0 0.068 0 -0.074 -0.151 0 0.251 -0.133 0.135 -0.225 0 -0.287 -0.01 -0.019 0.017 0 -0.08 0.011 0.004 0 0 0

15 1 0.057 -0.009 -0.039 0 0.068 0 -0.074 -0.187 0 0.251 0 0.135 -0.225 0 -0.287 -0.016 -0.019 0.017 0 0 0.128 0.004 0 0 0.001

15 1 0.057 -0.009 -0.039 0 0.068 0 -0.074 -0.187 0 0.251 0 0.135 -0.225 0 -0.26 -0.01 -0.019 0.017 0 0 0.182 0.004 0 0 0.001

15 1 0.057 -0.009 -0.039 0.097 0.068 0 -0.074 -0.092 0 0.251 0.003 0.21 -0.225 0 -0.287 -0.01 -0.019 0.017 0 0 0.128 0.004 0 0 0

15 1 0.057 -0.009 -0.039 0 0.068 0 -0.074 -0.092 0 0.251 0 0.135 -0.225 0 -0.21 -0.01 -0.019 0.017 0.018 0 0.128 0.004 0 0 0

15 1 0.057 -0.009 -0.039 -0.005 0.068 0 -0.074 -0.187 0 0.251 0 0.135 -0.225 0 -0.287 -0.01 -0.019 0.017 0 0 0.128 0.004 0 0 0.001

15 1 0.057 0 -0.039 0 0.068 0 -0.074 -0.113 0 0.251 -0.07 0.135 -0.225 0 -0.287 -0.01 -0.16 0.017 0 0 0.011 0.004 0 0 0

15 1 0.057 -0.009 -0.018 0 0.068 0 -0.074 -0.187 0 0.251 0 0.135 -0.225 0 -0.287 -0.01 -0.019 0.017 0 0 0.128 0.004 0 0.008 0.001

15 1 0.057 -0.009 -0.039 0 0.068 0 -0.074 -0.187 0 0.251 0.185 0.135 -0.225 0 -0.287 -0.01 -0.019 0.017 0 0 0.128 0.004 0 0 0.001

15 1 0.057 -0.009 -0.039 0 0.077 0 -0.074 -0.187 0 0.251 0 0.135 -0.225 0 -0.287 -0.01 -0.019 0.017 0 0 0.128 0.004 0 0.008 0.001

15 1 0.057 -0.009 -0.039 0 0.068 0 -0.074 -0.187 0 0.085 0 0.135 -0.225 0 -0.287 -0.01 -0.019 0.017 0 0 -0.024 0.004 0 0 0.001

15 1 0.057 0 -0.039 0 0.068 0 -0.074 -0.113 0 0.139 -0.133 0.135 -0.225 0 -0.287 -0.01 -0.019 0.017 0 -0.08 0.011 0.004 0 0 -0.125

15 1 0.057 0 -0.039 0 0.068 0 -0.074 -0.151 0 0.251 -0.133 0.135 -0.225 0 -0.287 -0.01 -0.019 0.017 0 -0.08 0.011 0.004 0 -0.099 0

15 1 0.057 0 -0.039 0 0.068 0 -0.074 -0.113 0 0.251 -0.07 0.135 -0.225 0 -0.287 -0.01 -0.019 0.017 0 -0.08 -0.033 0.004 0 0 0

15 1 0.057 -0.009 -0.039 0 0.068 0 -0.074 -0.187 0 0.251 0 0.135 -0.225 0 -0.287 -0.01 -0.019 0.017 0 0 0.128 0.004 0 0.021 0.001

15 1 0.057 0 -0.039 0 0.068 0 -0.074 -0.113 0 0.251 -0.07 0.135 -0.225 0 -0.287 -0.01 -0.019 0.017 0 -0.08 0.107 0.004 0 0 0

15 1 0.057 0 0.054 0 0.068 0 -0.074 -0.113 0 0.251 -0.07 0.135 -0.225 0 -0.287 -0.01 -0.019 0.017 0 0 0.011 0.004 0 0 0

15 1 0.057 -0.009 -0.039 0 0.068 0 -0.074 -0.187 0 0.251 0 0.135 -0.225 0 -0.287 -0.01 -0.103 0.017 0 0 0.128 0.004 0 0 0.001

15 1 0.057 -0.009 -0.039 0 0.068 0 -0.074 -0.187 0 0.251 0 0.135 -0.225 0 -0.26 -0.01 -0.019 0.017 0 0 0.128 0.004 0 0 -0.019

15 1 0.057 -0.009 -0.039 0 0.068 0 -0.074 -0.187 0 0.251 0 0.135 -0.225 0 -0.287 -0.01 -0.019 0.119 0 0 0.128 0.004 0 0.008 0.001

15 1 0.057 0 -0.039 0 0.068 0 -0.074 -0.113 0 0.251 -0.07 0.135 -0.225 0 -0.287 -0.01 -0.019 0.017 0 0 0.011 0.004 -0.079 0 0

15 1 0.057 -0.009 -0.039 0 0.068 0.009 -0.074 -0.187 0 0.251 0 0.135 -0.225 0.138 -0.287 -0.01 -0.019 0.017 0 0 0.128 0.004 0 0.056 -0.018

15 1 0.057 0 -0.039 0 0.068 0 -0.074 -0.113 0 0.251 -0.07 0.135 -0.225 -0.001 -0.287 -0.01 -0.019 0.017 0 0 0.011 0.004 0 0 0

15 1 0.057 -0.009 -0.039 0 0.068 0 -0.074 -0.187 0 0.251 0 0.135 -0.225 0 -0.287 -0.01 -0.019 0.017 0.017 0 0.128 0.004 0 0 0.001

15 1 0.057 0 -0.039 0 0.068 0 -0.074 -0.113 0 0.251 -0.191 0.135 -0.225 0.004 -0.287 -0.01 -0.019 0.017 0 -0.08 0.011 0.004 0 0 0

15 1 0.057 0.119 -0.039 0 0.068 0 -0.074 -0.187 0 0.251 0 0.135 -0.225 0 -0.287 -0.01 -0.019 0.017 0 0 0.128 0.004 0 0 0.001

15 1 0.057 0 -0.039 0 0.068 0 -0.074 -0.113 0 0.251 -0.07 0.135 -0.225 0 -0.287 -0.01 -0.019 0.017 0 -0.048 0.011 0.004 0 0 0.01

15 1 0.057 0 -0.039 0 0.068 0 -0.074 -0.113 0 0.244 -0.07 0.135 -0.225 0 -0.287 -0.01 -0.019 0.017 0 -0.08 0.011 0.004 0 0 0

15 1 0.057 0 -0.039 0 0.068 0 -0.074 -0.113 0 0.251 -0.07 0.135 -0.225 0.051 -0.287 -0.01 -0.019 0.017 0 0 0.011 0.004 -0.019 0 0

15 1 0.057 -0.009 -0.039 0 0.068 0 -0.074 -0.187 0 0.251 0 0.135 -0.225 0 -0.287 -0.01 -0.019 0.017 0.091 0 0.128 0.004 0 0 0.001

15 1 0.057 0 -0.039 0 0.068 0 -0.074 -0.113 0 0.251 -0.07 0.135 -0.225 0.052 -0.287 -0.01 -0.019 0.021 0 0 0.011 0.004 0 0 0

15 1 0.093 -0.009 -0.039 0 0.068 0 -0.074 -0.187 0 0.251 0 0.135 -0.225 0 -0.287 -0.01 -0.019 0.017 0 0 0.128 0.004 0 0.008 0.001

15 1 0.057 0 -0.039 0 0.068 0 -0.074 -0.113 0 0.251 -0.07 0.135 -0.08 0 -0.287 -0.01 -0.019 0.017 0 0 0.011 0.004 0 0 0

15 1 0.057 -0.009 -0.039 0 0.068 0 -0.074 -0.187 0 0.251 0 0.135 -0.225 0 -0.287 -0.01 -0.08 0.017 0 -0.105 0.128 0.004 0.183 0 0.001

15 1 0.057 0 -0.039 0 0.068 0 -0.074 -0.113 -0.032 0.251 -0.07 0.135 -0.225 0.085 -0.287 -0.01 -0.019 0.017 0 -0.08 0.011 0.004 0 0 0

15 1 0.057 0.115 -0.039 0 0.068 0 -0.074 -0.187 0 0.251 0 0.135 -0.225 0 -0.287 -0.01 -0.019 0.017 0 0 0.128 0.004 0 0.008 0.001

15 1 0.057 -0.009 -0.039 0 0.068 0 -0.074 -0.187 0 0.205 0 0.135 -0.225 0 -0.287 -0.01 -0.019 0.017 0 0 0.128 0.004 0 0 0.001

15 1 0.057 -0.009 -0.039 0 0.068 0 -0.074 -0.187 0 0.21 0 0.135 -0.225 0 -0.287 -0.01 -0.019 0.017 0 0 0.128 0.004 0 0.021 0.001

15 1 0.057 0 -0.039 0 0.141 0 -0.074 -0.113 0 0.394 -0.07 0.135 -0.225 0 -0.287 -0.01 -0.019 0.017 0 0 0.011 0.004 0 0 0

15 1 0.057 0 -0.039 0 0.068 0 -0.074 -0.113 0 0.251 -0.07 0.135 -0.225 0 -0.419 -0.01 -0.019 0.017 0 0 0.047 0.004 0 0 0

15 1 0.057 0 -0.039 0 0.068 0 -0.074 -0.113 0 0.251 -0.07 0.135 -0.225 0 -0.287 -0.01 -0.019 0.017 0 -0.08 0.011 -0.052 0 0 0

15 1 0.057 -0.009 -0.039 0 0.068 0 -0.074 -0.187 0 0.251 0 0.135 -0.225 0 -0.287 -0.01 -0.019 0.017 0 0 0.128 0.108 0 0 0.001

15 1 0.057 -0.009 -0.039 0 0.065 0 -0.074 -0.187 0 0.251 0 0.135 -0.225 0 -0.194 -0.01 -0.019 0.017 0 0 0.128 0.004 0 0.008 0.001

15 1 0.057 -0.009 -0.039 0 0.068 0 -0.074 -0.158 0 0.251 0 0.135 -0.225 0 -0.287 -0.01 -0.019 0.017 0 0 0.128 0.004 0 0 0.001

15 1 0.057 -0.009 -0.039 0 0.068 0 -0.074 -0.187 0 0.251 0 0.175 -0.225 0 -0.287 -0.01 -0.019 0.017 0 0 0.128 0.004 0 0 0.001

15 1 0.057 0 -0.039 0 0.068 0 -0.074 -0.113 -0.085 0.251 -0.07 0.135 -0.225 0 -0.287 -0.01 -0.019 0.017 0 0 0.011 0.004 0 0 0

15 1 0.057 0 -0.039 0 0.068 0 -0.074 -0.113 0 0.251 -0.07 0.199 -0.225 0.051 -0.287 -0.01 -0.019 0.017 0 0 0.011 0.004 0 0 0

15 1 0.057 -0.009 -0.039 0 0.068 -0.057 -0.074 -0.187 0 0.251 0 0.135 -0.225 0 -0.287 -0.01 -0.019 0.017 0 0 0.128 0.004 0 0 0.001

15 1 0.057 -0.009 -0.039 0.028 0.068 0 -0.074 -0.187 0 0.251 0 0.135 -0.225 0 -0.287 -0.01 -0.019 0.017 0 0 0.128 0.004 0 0.008 0.001

15 1 0.057 -0.009 -0.039 0 0.068 0 -0.074 -0.187 0 0.251 0 0.143 -0.225 0 -0.26 -0.01 -0.019 0.017 0 0 0.128 0.004 0 0 0.001

15 1 0.057 -0.009 -0.039 0 0.068 -0.089 -0.074 -0.187 0 0.251 0 0.048 -0.225 0.138 -0.287 -0.01 -0.019 0.017 0 0 0.128 0.004 0 0 0.001

15 1 0.057 0 -0.039 0 0.068 -0.139 -0.074 -0.113 0 0.251 -0.07 0.135 -0.225 0 -0.287 -0.01 -0.019 0.017 0 -0.08 0.011 0.004 0 0 0

15 1 0.057 -0.009 -0.039 0 0.068 0 -0.074 -0.187 0 0.251 0 0.135 -0.225 0 -0.26 -0.01 -0.019 0.017 0.021 0 0.128 0.004 0 0 0.001

15 1 0.057 0 -0.039 0 0.068 0 -0.074 -0.113 0 0.251 -0.12 0.135 -0.225 0 -0.287 -0.01 -0.019 0.017 0 0 0.011 0.004 0 0 0

15 1 0.057 -0.009 -0.039 0 0.068 0 -0.074 -0.187 0 0.251 0 0.135 -0.225 0 -0.287 -0.01 -0.019 0.017 0 0 0.101 0.004 0 0 0.001

15 1 0.057 -0.009 -0.039 0 0.068 0 -0.074 -0.187 0 0.251 0 0.135 -0.225 0 -0.26 -0.01 -0.019 -0.034 0 0 0.167 0.004 0 0 0.001

15 1 0.057 0 -0.039 0 0.068 0 -0.074 -0.113 0 0.251 -0.07 0.172 -0.225 0 -0.287 -0.01 -0.019 0.021 0 0 0.011 0.004 0 0 0

15 1 0.057 0 -0.039 0 0.068 0 -0.074 -0.113 0 0.251 -0.07 0.135 -0.225 0 -0.287 -0.01 -0.019 -0.099 0 -0.08 0.011 0.004 0 0 0

15 1 0.057 0 -0.039 0 0.068 0 -0.074 -0.113 0 0.251 -0.07 0.221 -0.225 0 -0.287 -0.01 -0.019 0.017 0 0 0.011 0.004 0 0 0

15 1 0.057 0 -0.039 0 0.068 0 -0.074 -0.113 0 0.251 -0.133 0.146 -0.225 0 -0.287 -0.01 -0.019 0.017 0 -0.08 0.011 0.004 0 0 0

15 1 0.057 -0.009 -0.17 0 0.068 0 -0.074 -0.187 0 0.251 0 0.135 -0.225 0 -0.287 -0.01 -0.019 0.017 0 0 0.128 0.004 0 0 0.001

15 1 0.057 -0.009 -0.039 0 0.068 0 -0.074 -0.092 0 0.251 0 0.091 -0.225 0 -0.21 -0.01 -0.019 0.017 0 0 0.128 0.004 0 0 0

15 1 0.057 0 -0.039 0 0.054 0 -0.074 -0.113 0 0.251 -0.07 0.135 -0.225 0 -0.287 -0.01 -0.019 0.017 0 0 0.011 0.004 0 0 0.109

15 1 0.057 0 -0.039 0 0.068 0 -0.074 -0.113 0 0.251 0.093 0.135 -0.225 0 -0.287 -0.01 -0.019 0.017 0 0 0.011 0.004 0 0 0

15 1 0.057 0 -0.039 0 -0.057 0 -0.074 -0.113 0 0.251 -0.07 0.135 -0.225 0.004 -0.287 -0.01 -0.019 0.017 0 -0.08 0.011 0.004 0 0 0

15 1 0.057 -0.009 -0.039 0 0.068 0 -0.074 -0.187 0 0.251 0 0.135 -0.225 0 -0.287 -0.01 0.063 0.017 0 0 0.128 0.004 0 0.008 0.001

15 1 0.057 -0.009 -0.039 0 0.068 0 -0.074 -0.187 0 0.227 0 0.135 -0.225 0 -0.287 -0.01 -0.019 0.017 0 0 0.128 0.004 0 0.008 0.001

15 1 0.057 0 -0.039 0 0.068 0 -0.074 -0.25 0 0.251 -0.07 0.135 -0.225 0 -0.419 -0.01 -0.019 0.017 0 0 -0.03 0.004 0 0 0

15 1 0.057 0 -0.039 0 0.068 0 -0.074 -0.113 0 0.251 -0.07 0.135 -0.225 0 -0.287 -0.01 0.043 0.017 0 -0.08 0.011 0.004 0 0 0

15 1 0.057 0 -0.039 -0.058 0.068 0 -0.074 -0.113 0 0.251 -0.07 0.135 -0.225 0 -0.287 -0.01 -0.019 0.017 0 0 0.011 0.004 0 0 0

16 1030 -0.162 0.294 0 0.093 0 -0.033 -0.073 -0.02 0.131 0 0.082 0 -0.065 -0.095 0 0.316 0.186 0.054 0.095 -0.062 0 -0.203 0.269 0.09 -0.045

16 691 -0.162 0.294 0 0.093 0 -0.033 -0.073 -0.02 0.131 0 0 0 -0.065 -0.131 0 0.5 0.186 0.054 0.095 -0.085 0 -0.203 0 0.09 -0.001

16 457 -0.162 0.294 0 0.093 0 -0.033 -0.073 -0.02 0.131 0 0.082 0 -0.065 -0.088 0 0.316 0.186 0.054 0.095 -0.062 0 -0.203 0.269 0.09 -0.045

16 420 -0.162 0.294 0 0.093 0 -0.033 -0.073 -0.02 0.131 0 0.082 -0.003 -0.065 -0.044 0 0.316 0.186 0.054 0.095 -0.062 0 -0.203 0.269 0.09 -0.045

16 328 -0.162 0.294 0 0.093 0 -0.033 -0.073 -0.02 0.131 0 0.082 0 -0.065 -0.044 0 0.316 0.035 0.054 0.095 -0.062 0 -0.203 0.269 0.09 -0.045

16 182 -0.162 0.294 0 0.093 0 -0.033 -0.073 -0.02 0.131 0 0.082 0 -0.065 -0.131 0 0.316 0.186 0.054 0.095 -0.062 0 -0.203 0.269 0.09 -0.045

16 125 -0.149 0.294 0.085 0.126 0.036 -0.033 -0.073 -0.02 0.131 0 -0.022 0 -0.065 -0.131 0 0.316 0.186 0.054 0.095 -0.062 0 -0.203 0.114 0.09 -0.001

16 117 -0.136 0.294 0 0.093 0 -0.033 -0.073 -0.02 0.131 0 0 0 -0.065 -0.131 0 0.5 0.186 0.054 0.095 -0.085 0 -0.203 0 0.09 -0.001

16 91 -0.162 0.294 0 0.195 0 -0.033 -0.073 -0.02 0.131 0 0.082 -0.003 -0.065 -0.044 0 0.316 0.186 0.054 0.095 -0.062 0 -0.203 0.269 0.09 -0.045

16 54 -0.162 0.294 0 0.093 0 -0.033 -0.073 -0.02 0.131 0 0 0 -0.065 -0.125 0 0.5 0.186 0.054 0.095 -0.085 0 -0.203 0 0.09 -0.001

16 44 -0.162 0.294 0 0.093 0 -0.033 -0.073 -0.02 0.131 0 0.082 0 -0.117 -0.088 0 0.316 0.186 0.054 0.095 -0.062 0 -0.203 0.269 0.09 -0.045

16 35 -0.162 0.294 0 0.093 0 -0.033 -0.073 -0.02 0.131 0 0 0 -0.065 -0.131 -0.042 0.5 0.186 0.054 0.095 -0.085 0 -0.203 0 0.09 -0.001

16 32 -0.162 0.294 0 0.093 0 -0.033 -0.073 -0.02 0.131 0 0.082 0 -0.065 -0.095 0 0.316 0.186 0.054 0.095 -0.062 0 -0.203 0.269 -0.076 -0.045

16 19 -0.162 0.294 0 0.195 0 -0.033 -0.073 -0.02 0.131 0 0.082 -0.003 -0.065 -0.044 0.021 0.316 0.186 0.054 0.095 -0.062 0 -0.203 0.269 0.09 -0.045

16 17 -0.162 0.294 0 0.093 0 -0.033 -0.073 -0.02 0.131 0 0 0 -0.065 -0.131 -0.07 0.5 0.186 0.054 0.095 -0.085 0 -0.203 0 0.09 -0.001

16 19 -0.162 0.294 0 0.093 0 -0.033 -0.073 -0.02 0.131 0 0.057 0 -0.065 -0.131 0 0.316 0.186 0.054 0.095 -0.062 0 -0.203 0.269 0.09 -0.045

16 16 -0.162 0.294 0 0.093 0.081 -0.033 -0.073 -0.02 0.131 0 0 0 -0.065 -0.131 0 0.5 0.186 0.054 0.095 -0.085 0 -0.203 0 0.09 -0.001

16 15 -0.162 0.294 0 0.093 0 -0.033 -0.073 -0.02 0.131 0 0.082 0 -0.065 -0.131 0 0.316 0.186 0.054 0.095 -0.062 0 -0.131 0.269 0.09 -0.045

16 12 -0.162 0.294 0 0.093 0 -0.033 -0.073 -0.02 0.131 0 0 0 -0.065 -0.131 -0.042 0.5 0.186 0.054 0.095 -0.128 0 -0.203 0 0.09 -0.001

16 12 -0.162 0.294 0.007 0.093 0 -0.033 -0.073 -0.02 0.131 0 0.082 0 -0.065 -0.095 0 0.316 0.186 0.054 0.095 -0.062 0 -0.203 0.269 0.09 -0.045

16 11 -0.301 0.294 0 0.093 0 -0.033 -0.073 -0.02 0.131 0 0.082 0 -0.065 -0.095 0 0.316 0.186 0.054 0.095 -0.062 0 -0.203 0.269 0.09 -0.045

16 11 -0.162 0.294 0 0.093 0 -0.033 -0.073 -0.02 0.131 0 0.082 0 -0.065 -0.095 0 0.316 0.186 0.054 0.095 -0.104 0 -0.203 0.269 0.09 -0.045

16 10 -0.162 0.294 0 0.093 0 -0.033 -0.073 -0.02 0.131 0 0 0 -0.065 -0.131 0 0.297 0.186 0.054 0.095 -0.085 0 -0.203 0 0.09 -0.001

16 8 -0.104 0.294 0 0.093 0 -0.033 -0.073 -0.02 0.131 0 0.082 0 -0.077 -0.044 0 0.316 0.035 0.054 0.095 -0.062 0 -0.203 0.269 0.09 -0.045

16 8 -0.162 0.294 0 0.093 0 -0.033 -0.073 -0.02 0.131 0 0 0 -0.065 -0.131 0 0.5 0.186 0.054 0.095 -0.132 0 -0.203 0 0.09 -0.001

16 9 -0.162 0.294 0 0.093 0 -0.033 -0.073 -0.02 0.131 0 0.082 -0.003 -0.065 -0.044 0 0.316 0.186 0.054 0.442 -0.062 0 -0.203 0.269 0.09 -0.045

16 7 -0.162 0.294 0 0.13 0 -0.033 -0.073 -0.02 0.131 0 0 0 -0.065 -0.131 -0.042 0.5 0.186 0.054 0.095 -0.085 0 -0.203 0 0.09 -0.001

16 6 -0.162 0.294 0 0.093 0 -0.033 -0.073 -0.02 0.131 0 0.082 0 -0.065 -0.131 0 0.316 0.186 0.148 0.095 -0.062 0 -0.203 0.269 0.09 -0.045

16 6 -0.162 0.294 0 0.093 0 -0.033 -0.073 -0.02 0.131 0 0.082 0 -0.027 -0.088 0 0.316 0.186 0.054 0.095 -0.062 0 -0.203 0.269 0.09 -0.045

16 5 -0.162 0.294 0 0.093 0 -0.033 -0.073 -0.02 0.131 0 0.082 0 -0.192 -0.095 0 0.316 0.186 0.054 0.095 -0.062 0 -0.203 0.269 0.09 -0.045

16 4 -0.162 0.294 0 0.093 0 -0.033 -0.073 -0.02 0.131 0 0.082 0 -0.065 0.034 0 0.316 0.186 0.054 0.095 -0.062 0 -0.203 0.269 0.09 -0.045

16 4 -0.149 0.294 0 0.126 0.036 -0.033 -0.073 -0.02 0.131 0 -0.022 0 -0.065 -0.131 0 0.316 0.186 0.054 0.095 -0.062 0 -0.203 0.114 0.09 -0.001

16 4 -0.162 0.294 0 0.093 0 -0.055 -0.073 -0.02 0.131 0 0 0 -0.065 -0.131 0 0.5 0.186 0.054 0.095 -0.085 0 -0.203 0 0.09 -0.001

16 4 -0.162 0.294 0 0.093 0 -0.033 -0.073 -0.02 0.131 0 0.082 0 -0.065 -0.057 0 0.316 0.186 0.054 0.095 -0.062 0 -0.203 0.269 0.09 -0.045

16 4 -0.162 0.294 0 0.093 0 -0.033 -0.073 -0.02 0.131 -0.196 0.082 0 -0.065 -0.131 0 0.316 0.186 0.054 0.095 -0.062 0 -0.203 0.269 0.09 -0.045

16 4 -0.162 0.294 0 0.093 0 -0.033 -0.073 -0.02 0.131 0 0 0 -0.065 -0.131 -0.042 0.5 0.186 0.054 0.095 -0.128 -0.065 -0.203 0 0.09 -0.001

16 4 -0.162 0.294 -0.06 0.093 0 -0.033 -0.073 -0.02 0.131 0 0.082 0 -0.065 -0.095 0 0.316 0.186 0.054 0.095 -0.062 0 -0.203 0.269 0.09 -0.045

16 4 -0.162 0.294 0 0.093 0 -0.033 -0.141 -0.02 0.131 0 0.082 0 -0.065 -0.131 0 0.316 0.186 0.054 0.095 -0.062 0 -0.203 0.269 0.09 -0.045

16 7 -0.162 0.294 -0.051 0.093 0 -0.033 -0.073 -0.02 0.131 0 0 0 -0.065 -0.131 0 0.5 0.186 0.054 0.095 -0.085 0 -0.203 0 0.09 -0.001

16 3 -0.162 0.294 0 0.098 0 -0.033 -0.073 -0.02 0.131 0 0 0 -0.065 -0.131 0 0.5 0.186 0.054 0.095 -0.085 0 -0.203 0 0.09 -0.001

16 3 -0.162 0.398 0 0.093 0 -0.033 -0.073 -0.02 0.131 0 0.082 -0.003 -0.065 -0.044 0 0.316 0.186 0.054 0.095 -0.062 0 -0.203 0.269 0.09 -0.045

16 3 -0.149 0.294 0.085 0.126 0.036 -0.033 -0.187 -0.02 0.131 0 -0.022 0 -0.065 -0.131 0 0.316 0.186 0.054 0.095 -0.062 0 -0.203 0.114 0.09 -0.001

16 3 -0.149 0.294 0.085 0.126 0.1 -0.033 -0.073 -0.02 0.131 0 -0.022 0 -0.065 -0.131 0 0.316 0.186 0.054 0.095 -0.062 0 -0.203 0.114 0.09 -0.001

16 3 -0.162 0.294 0 0.099 0 -0.033 -0.073 -0.02 0.131 0 0.082 0 -0.065 -0.131 0 0.316 0.186 0.054 0.095 -0.062 0 -0.203 0.269 0.109 -0.045

16 3 -0.162 0.294 0 0.093 0 -0.033 -0.073 -0.02 0.131 0 0.082 -0.003 -0.065 -0.019 0 0.316 0.186 0.054 0.095 -0.062 0 -0.203 0.269 0.09 -0.045

16 3 -0.162 0.294 0 0.093 -0.064 -0.033 -0.073 -0.02 0.131 0 0 0 -0.065 -0.125 0 0.5 0.186 0.054 0.095 -0.085 0 -0.203 0 0.09 -0.001

16 3 -0.162 0.294 0 0.093 0 -0.033 -0.073 -0.02 0.131 0 0.082 0 -0.065 -0.095 0 0.316 0.186 0.054 0.245 -0.062 0 -0.203 0.269 0.09 -0.045

16 3 -0.118 0.294 0 0.093 0 -0.033 -0.073 -0.02 0.131 0 0 0 -0.065 -0.125 0 0.5 0.186 0.054 0.095 -0.085 0 -0.203 0 0.09 -0.001

16 2 -0.162 0.294 0 0.093 0 -0.033 -0.073 -0.02 0.131 0 0.082 0 -0.065 -0.095 0 0.316 0.186 0.014 0.095 -0.062 0 -0.203 0.269 0.09 -0.045

16 2 -0.162 0.294 0 0.093 0 -0.033 -0.073 -0.02 0.131 0 0 0 -0.065 -0.131 0 0.5 0.186 0.054 0.095 -0.085 0 -0.203 0.015 0.09 -0.001

16 2 -0.162 0.294 0 0.093 0 -0.033 -0.073 -0.02 0.131 0 0.082 -0.003 -0.072 -0.044 0 0.316 0.186 0.054 0.095 -0.062 0 -0.203 0.269 0.09 -0.045

16 2 -0.162 0.294 0 0.093 0 -0.033 -0.073 -0.02 0.131 0 0 0 -0.065 -0.131 0 0.694 0.186 0.054 0.095 -0.085 0 -0.203 0 0.09 -0.001

16 2 -0.162 0.294 0 0.093 0 -0.033 -0.073 -0.02 0.131 0 0.082 0 -0.076 -0.131 0 0.316 0.186 0.054 0.095 -0.062 0 -0.203 0.269 0.09 -0.045

16 4 -0.162 0.294 0 0.093 0 -0.033 -0.073 -0.02 0.131 0 0.082 -0.079 -0.065 -0.095 0 0.316 0.186 0.054 0.095 -0.062 0 -0.203 0.269 0.09 -0.045

16 2 -0.162 0.323 0 0.093 0 -0.033 -0.073 -0.02 0.131 0 0.082 0 -0.065 -0.044 0 0.316 0.035 0.054 0.095 -0.062 0 -0.203 0.269 0.09 -0.045

16 2 -0.162 0.294 0 0.093 0 -0.033 -0.073 -0.02 0.131 0 0.082 0 -0.065 -0.131 0 0.316 0.186 0.032 0.095 -0.062 0 -0.203 0.269 0.09 -0.045

16 2 -0.162 0.294 0 0.093 0 -0.033 -0.011 -0.02 0.131 0 0.082 0 -0.065 -0.044 0 0.316 0.035 0.054 0.095 -0.062 0 -0.203 0.269 0.09 -0.045

16 2 -0.162 0.294 0 0.093 0 -0.033 -0.073 -0.02 0.131 0 0 0 -0.065 -0.125 0 0.5 0.186 0.041 0.095 -0.085 0 -0.203 0 0.09 -0.001

16 2 -0.162 0.294 0 0.121 0 -0.033 -0.073 -0.02 0.131 0 0 0 -0.065 -0.131 0 0.5 0.186 0.054 0.095 -0.085 0 -0.203 0 0.09 -0.001

16 2 -0.162 0.177 0 0.093 0 -0.033 -0.073 -0.02 0.131 0 0.082 0 -0.065 -0.088 0 0.316 0.186 0.054 0.095 -0.062 0 -0.203 0.269 0.09 -0.045

16 2 -0.162 0.294 0 0.093 0 -0.049 -0.073 -0.02 0.131 0 0 0 -0.065 -0.131 0 0.5 0.186 0.054 0.095 -0.085 0 -0.203 0 0.09 -0.001

16 2 -0.162 0.294 0 0.093 0 0.148 -0.073 -0.02 0.131 0 0.082 0 -0.065 -0.095 0 0.316 0.186 0.054 0.095 -0.062 0 -0.203 0.269 0.09 -0.045

16 2 -0.162 0.294 0 0.257 0 -0.033 -0.073 -0.02 0.131 0 0 0 -0.065 -0.131 -0.042 0.5 0.186 0.054 0.095 -0.085 0 -0.203 0 0.09 -0.001

16 2 -0.162 0.294 0 0.093 0 -0.033 -0.073 -0.02 0.131 0 0.082 0 -0.065 -0.095 0 0.316 0.186 0.054 0.154 -0.062 0 -0.203 0.269 0.09 -0.045

16 2 -0.162 0.294 0 0.093 0 -0.033 -0.073 -0.02 0.131 0.072 0.082 0 -0.065 -0.095 0 0.316 0.186 0.054 0.095 -0.062 0 -0.203 0.269 0.09 -0.045

16 2 -0.162 0.294 0 0.093 0 -0.033 -0.073 -0.02 0.131 0 0.082 0 -0.065 -0.131 0 0.316 0.186 0.054 0.08 -0.062 0 -0.203 0.269 0.09 -0.045

16 2 -0.162 0.294 0 0.093 0 -0.033 -0.073 -0.02 0.131 0 0.082 0 -0.065 -0.088 0 0.316 0.186 0.029 0.095 -0.062 0 -0.203 0.269 0.09 -0.045

16 2 -0.162 0.294 0 0.093 0 -0.033 -0.073 -0.02 0.131 0 0.077 0 -0.065 -0.044 0 0.316 0.035 0.054 0.095 -0.062 0 -0.203 0.269 0.09 -0.045

16 2 -0.162 0.294 0 0.093 0 -0.033 -0.073 -0.02 0.131 0 0.082 0 -0.065 -0.095 0 0.316 0.186 0.054 0.095 -0.062 0 -0.203 0.269 0.244 -0.045

16 2 -0.162 0.294 0 0.195 0 -0.033 -0.073 -0.02 0.131 0 0.082 -0.003 -0.065 -0.044 0 0.316 0.186 0.054 0.107 -0.062 0 -0.203 0.269 0.09 -0.045

16 2 -0.149 0.294 0.085 0.126 0.036 -0.324 -0.073 -0.02 0.131 0 -0.022 0 -0.065 -0.131 0 0.316 0.186 0.054 0.095 -0.062 0 -0.203 0.114 0.09 -0.001

16 2 -0.162 0.294 0 0.093 0 -0.033 -0.073 -0.02 0.131 0 0.082 0 -0.062 -0.095 0 0.316 0.186 0.054 0.095 -0.062 0 -0.203 0.269 0.09 -0.045

16 2 -0.149 0.294 0.085 0.126 0.036 -0.033 -0.073 -0.02 0.131 0 -0.022 0 -0.071 -0.131 0 0.316 0.186 0.054 0.095 -0.062 0 -0.203 0.114 0.09 -0.001

16 2 -0.162 0.294 0 0.093 0 -0.033 -0.073 -0.02 0.131 0 0.082 -0.034 -0.065 -0.095 0 0.316 0.186 0.054 0.095 -0.062 0 -0.203 0.269 0.09 -0.045

16 2 -0.162 0.294 0 0 0 -0.033 -0.073 -0.02 0.131 0 0.082 0 -0.065 -0.095 0 0.316 0.186 0.054 0.095 -0.062 0 -0.203 0.269 0.09 -0.045

16 2 -0.162 0.294 0 0.195 0 -0.033 -0.073 -0.02 0.131 0 0.141 -0.003 -0.065 -0.044 0 0.316 0.186 0.054 0.095 -0.062 0 -0.203 0.269 0.09 -0.045

16 2 -0.162 0.294 0 0.093 0 -0.033 -0.073 -0.02 0.131 0 0.082 0 0.024 -0.095 0 0.316 0.186 0.054 0.095 -0.062 0 -0.203 0.269 0.09 -0.045

16 2 -0.162 0.294 0 0.093 0 -0.033 -0.073 -0.02 0.131 0 0.082 0 -0.065 -0.044 0 0.316 0.035 0.054 0.095 -0.074 0 -0.203 0.269 0.09 -0.045

16 2 -0.162 0.294 0 0.093 0 -0.033 -0.073 -0.02 0.131 0 0.082 0 -0.065 -0.044 -0.043 0.316 0.035 0.054 0.095 -0.062 0 -0.203 0.269 0.09 -0.045

16 2 -0.162 0.294 0 0.093 0 -0.033 -0.073 -0.02 0.131 0.009 0 0 -0.065 -0.131 0 0.5 0.186 -0.085 0.095 -0.085 0 -0.203 0 0.09 -0.001

16 1 -0.162 0.294 0 0.19 0 -0.033 -0.073 -0.02 0.131 0 0.082 0 -0.065 -0.131 0 0.316 0.186 0.054 0.095 -0.062 0 -0.203 0.269 0.09 -0.045

16 1 -0.162 0.294 0 0.093 0 -0.033 -0.073 -0.02 0.131 0 0 0 -0.065 -0.131 0 0.5 0.186 0.005 0.095 -0.085 0 -0.203 0 0.09 -0.001

16 1 -0.162 0.294 0 0.093 0 -0.033 -0.073 0.087 0.131 0 0 0 -0.065 -0.131 0 0.5 0.186 0.054 0.095 -0.085 0 -0.203 0.015 0.09 -0.001

16 1 -0.162 0.294 0 0.093 0 -0.033 -0.073 -0.02 0.131 0 0.082 0 -0.065 -0.095 0 0.316 0.186 0.054 0.095 -0.062 0 -0.203 0.269 -0.076 -0.101

16 1 -0.162 0.294 0 0.093 0 -0.033 -0.073 -0.02 0.131 0 0.082 0 -0.065 -0.131 0 0.316 0.186 0.054 0.095 -0.062 -0.006 -0.131 0.269 0.09 -0.045

16 1 -0.162 0.331 0 0.093 0 -0.033 -0.073 -0.02 0.131 0 0.082 0 -0.065 -0.044 0 0.316 0.035 0.054 0.095 -0.062 0 -0.203 0.269 0.09 -0.045

16 1 -0.162 0.294 0.166 0.093 0 -0.033 -0.073 -0.02 0.131 0 0.082 0 -0.065 -0.095 0 0.316 0.186 0.054 0.095 -0.062 0 -0.203 0.269 0.09 -0.045

16 1 -0.162 0.294 0 0.093 0 -0.033 -0.073 -0.02 0.131 0 0.082 0 -0.027 -0.088 0 0.316 0.186 0.054 0.095 -0.062 0 -0.203 0.269 0.09 0.018

16 1 -0.162 0.294 0 0.093 0 -0.033 -0.073 -0.02 0.131 0 0 0 -0.065 -0.131 0.136 0.5 0.186 0.054 0.095 -0.085 0 -0.203 0 0.09 -0.001

16 1 -0.162 0.294 -0.096 0.093 0 -0.033 -0.073 -0.02 0.131 0 0.057 0 -0.065 -0.131 0 0.316 0.186 0.054 0.095 -0.062 0 -0.203 0.269 0.09 -0.045

16 1 -0.162 0.335 0 0.093 0 -0.033 -0.073 -0.02 0.131 0 0.082 0 -0.065 -0.131 0 0.316 0.186 0.054 0.095 -0.062 0 -0.203 0.269 0.09 -0.045

16 1 -0.162 0.294 0 0.13 0 -0.033 -0.073 -0.02 0.131 0 0 0 -0.065 -0.131 -0.042 0.5 0.186 0.054 0.095 -0.047 0 -0.203 0 0.09 -0.001

16 1 -0.162 0.294 0 0.093 0 -0.033 -0.048 -0.02 0.131 0 0 0 -0.065 -0.131 0 0.5 0.186 0.054 0.095 -0.085 0 -0.203 0 0.09 -0.001

16 1 -0.149 0.294 0.085 0.126 0.036 -0.033 -0.073 -0.02 0.131 0 -0.022 0 -0.065 -0.026 0 0.316 0.186 0.054 0.095 -0.062 0 -0.203 0.114 0.09 -0.001

16 1 -0.162 0.294 0 0.093 0 -0.033 -0.073 -0.02 0.131 0 0.082 -0.003 -0.065 -0.044 0 0.316 0.186 0.054 0.095 -0.062 0 -0.203 0.269 0.09 -0.107

16 1 -0.162 0.294 0 0.093 0 -0.033 -0.073 -0.02 0.131 0 0.057 0 -0.065 -0.131 0 0.317 0.186 0.054 0.095 -0.062 0 -0.203 0.269 0.09 -0.045

16 1 0.028 0.294 0 0.093 0 -0.033 -0.073 -0.02 0.131 0 0.082 0 -0.065 -0.095 0 0.316 0.186 0.054 0.095 -0.062 0 -0.203 0.269 0.09 -0.045

16 1 -0.162 0.294 0 0.093 0 -0.033 -0.073 -0.02 0.131 0 0.082 0 -0.065 -0.095 0.056 0.316 0.186 0.054 0.095 -0.062 0 -0.203 0.269 -0.076 -0.045

16 1 -0.162 0.294 0 0.093 0 -0.033 -0.073 -0.02 0.131 0 0.082 0 -0.065 -0.095 0 0.294 0.186 0.054 0.095 -0.062 0 -0.203 0.269 0.09 -0.045

16 1 -0.162 0.294 0 0.195 0 -0.033 -0.073 -0.02 0.131 0 0.082 -0.003 -0.065 -0.044 0 0.316 0.186 0.054 0.095 0.036 0 -0.203 0.269 0.09 -0.045

16 1 -0.162 0.294 0 0.093 0 -0.033 -0.073 -0.02 0.131 0 0.082 -0.003 -0.065 -0.044 0 0.316 0.186 0.054 0.095 -0.062 0 -0.203 0.269 0.09 -0.092

16 1 -0.162 0.294 0 0.093 0 -0.033 -0.073 -0.02 0.131 0 0.082 -0.003 -0.065 -0.044 0 0.316 0.186 0.054 0.095 -0.062 0 -0.203 0.269 0.09 -0.137

16 1 -0.162 0.294 0 0.093 0 -0.033 -0.073 -0.02 0.131 0 0 0 -0.036 -0.131 0 0.5 0.186 0.054 0.095 -0.085 0 -0.203 0 0.09 -0.001

16 1 -0.162 0.294 0 0.093 0 -0.033 -0.073 -0.02 0.131 -0.079 0.082 -0.003 -0.065 -0.044 0 0.316 0.186 0.054 0.095 -0.062 0 -0.203 0.269 0.09 -0.045

16 1 -0.162 0.294 0 0.093 0 -0.033 -0.073 -0.02 0.131 0 0.181 0 -0.065 -0.131 0 0.5 0.186 0.054 0.095 -0.085 0 -0.203 0 0.09 -0.001

16 1 -0.162 0.294 0 0.093 0 -0.033 -0.171 -0.02 0.131 0 0.082 0 -0.065 -0.095 0 0.316 0.186 0.054 0.095 -0.062 0 -0.203 0.269 0.09 -0.045

16 1 -0.162 0.294 0 0.093 0 -0.033 -0.073 -0.02 0.131 0 0.082 -0.003 -0.065 -0.044 0 0.316 0.186 0.054 0.095 -0.137 0 -0.203 0.269 0.09 -0.045

16 1 -0.162 0.294 0 0.093 0 -0.033 -0.073 -0.02 0.131 0 0.082 -0.003 -0.065 -0.044 0 0.387 0.186 0.054 0.095 -0.062 0 -0.203 0.269 0.09 -0.045

16 1 -0.162 0.294 0 0.093 0 -0.033 -0.073 -0.02 0.131 0 0.082 0 -0.065 -0.095 0 0.316 0.186 0.054 0.095 -0.062 0 -0.203 0.269 0.09 0.045

16 1 -0.224 0.294 0 0.093 0 -0.033 -0.073 -0.02 0.131 0 0.082 0 -0.065 -0.095 0 0.316 0.186 0.054 0.095 -0.062 0 -0.203 0.269 0.09 -0.045

16 1 -0.162 0.294 0 0.093 0 0.044 -0.073 -0.02 0.131 0 0.082 0 -0.065 -0.095 0 0.316 0.186 0.054 0.095 -0.062 0 -0.203 0.269 0.09 -0.045

16 1 -0.162 0.294 0 0.093 0 -0.033 -0.073 -0.02 0.131 0 0 0 -0.065 -0.131 0 0.5 0.186 0.054 0.095 0.03 0 -0.203 0 0.09 -0.001

16 1 -0.162 0.294 0 0.093 0 -0.033 -0.073 -0.02 0.131 0 0.082 0 -0.065 -0.044 0 0.405 0.035 0.054 0.095 -0.062 0 -0.203 0.269 0.09 -0.045

16 1 -0.162 0.294 0 0.093 0 -0.033 -0.083 -0.02 0.131 0 0.082 0 -0.065 -0.095 0 0.316 0.186 0.054 0.095 -0.062 0 -0.203 0.269 0.09 -0.045

16 1 -0.162 0.294 0 0.093 0 -0.033 -0.073 -0.02 0.131 0 0.082 0.05 -0.065 -0.131 0 0.316 0.186 0.054 0.095 -0.062 0 -0.203 0.269 0.09 -0.045

16 1 -0.162 0.294 0 0.093 0 -0.033 -0.073 -0.02 0.064 -0.008 0.082 0 -0.065 -0.095 0 0.316 0.186 0.054 0.095 -0.062 0 -0.203 0.269 0.09 -0.045

16 1 -0.162 0.294 0 0.093 0 -0.033 -0.073 -0.02 0.131 0 0.082 0 -0.065 -0.095 -0.146 0.316 0.186 0.054 0.095 -0.062 0 -0.203 0.269 0.09 -0.045

16 1 -0.162 0.294 0 0.093 0 -0.033 -0.073 -0.097 0.131 0 0 0 -0.065 -0.131 -0.042 0.5 0.186 0.054 0.095 -0.085 -0.179 -0.203 0 0.09 -0.001

16 1 -0.22 0.294 0 0.093 0 -0.033 -0.073 -0.02 0.131 0 0 0 -0.065 -0.131 0 0.5 0.066 0.054 0.095 -0.085 0 -0.203 0 0.09 -0.001

16 1 -0.162 0.294 0 0.093 0 -0.033 -0.073 -0.022 0.131 0 0 0 -0.065 -0.131 0 0.5 0.186 0.054 0.095 -0.085 0 -0.203 0 0.09 -0.001

16 1 -0.162 0.294 0 0.093 0 -0.033 -0.184 -0.02 0.131 0 0.082 0 -0.065 -0.044 0 0.316 0.035 0.054 0.095 -0.062 0 -0.203 0.269 0.09 -0.045

16 1 -0.162 0.294 0.025 0.093 0 -0.033 -0.073 -0.02 0.131 0 0 0 -0.065 -0.131 0 0.5 0.186 0.054 0.095 -0.085 0 -0.203 0 0.09 -0.001

16 1 -0.162 0.294 0 0.093 0 -0.033 -0.137 -0.02 0.131 0 0.082 0 -0.065 -0.088 0 0.316 0.186 0.054 0.095 -0.062 0 -0.203 0.269 0.09 -0.045

16 1 -0.162 0.294 0 0.093 0 0.004 -0.073 -0.02 0.131 0 0.082 0 -0.065 -0.088 0 0.316 0.186 0.054 0.095 -0.062 0 -0.203 0.269 0.09 -0.045

16 1 -0.162 0.294 0 0.016 0 -0.033 -0.073 -0.02 0.131 0 0.082 0 -0.065 -0.088 0 0.316 0.186 0.054 0.095 -0.062 0 -0.203 0.269 0.09 -0.045

16 1 -0.162 0.294 0 0.093 0 -0.033 -0.073 -0.02 0.131 0 0.082 0 -0.065 -0.044 0 0.316 0.035 0.054 0.149 -0.062 0 -0.203 0.269 0.09 -0.045

16 1 -0.162 0.215 0 0.093 0 -0.033 -0.073 -0.02 0.131 0 0.082 0 -0.065 -0.095 0 0.316 0.186 0.054 0.095 -0.062 0 -0.203 0.269 0.09 -0.045

16 1 -0.162 0.294 0 0.093 0.01 -0.049 -0.073 -0.02 0.131 0 0 0 -0.065 -0.131 0 0.5 0.186 0.054 0.095 -0.085 0 -0.203 0 0.09 -0.001

16 1 -0.162 0.294 0 0.093 0 -0.049 -0.073 -0.02 0.131 0 0 0 -0.065 -0.131 0 0.5 0.186 0.054 0.095 -0.085 0 -0.203 0 0.09 0.116

16 1 -0.162 0.294 0 0.188 0 -0.033 -0.073 -0.02 0.131 0 0 0 -0.065 -0.131 0 0.5 0.186 0.054 0.095 -0.085 0 -0.203 0 0.09 -0.001

16 1 -0.162 0.294 0 0.093 0.081 -0.033 -0.073 -0.02 0.131 0 0 0 -0.065 -0.264 0 0.5 0.186 0.054 0.095 -0.085 0 -0.203 0 0.09 -0.001

16 1 -0.162 0.294 -0.051 0.093 0 -0.033 -0.073 -0.02 0.131 0 0 0 -0.065 -0.131 0 0.5 0.186 0.054 0.095 -0.085 -0.073 -0.203 0 0.09 -0.001

16 1 -0.162 0.294 0 0.093 0 0.036 -0.073 -0.02 0.131 0 0 0 -0.065 -0.131 0 0.5 0.186 0.054 0.095 -0.085 0 -0.203 0 0.09 -0.001

16 1 -0.162 0.294 0 0.093 0 -0.033 -0.073 -0.02 0.131 0 0.082 0 -0.065 -0.044 0 0.316 0.035 0.054 0.095 -0.062 0 -0.203 0.112 0.09 -0.045

16 1 -0.162 0.294 0 0.093 0.014 -0.033 -0.073 -0.02 0.131 0 0.082 0 -0.065 -0.088 0 0.316 0.186 0.054 0.095 -0.062 0 -0.203 0.269 0.09 -0.045

16 1 -0.162 0.294 0 0.093 0 -0.033 -0.073 -0.02 0.131 0 0.082 -0.003 -0.065 -0.044 0 0.427 0.186 0.054 0.095 -0.062 0 -0.203 0.269 0.09 -0.045

16 1 -0.162 0.294 0 0.093 0 -0.033 -0.073 -0.02 0.131 0 0.082 0 -0.065 -0.095 0 0.316 0.186 0.054 0.095 -0.21 0 -0.203 0.269 0.09 -0.045

16 1 -0.162 0.294 0.126 0.093 0 -0.033 -0.073 -0.02 0.131 0 0.082 -0.003 -0.065 -0.044 0 0.316 0.186 0.054 0.095 -0.062 0 -0.203 0.269 0.09 -0.045

16 1 -0.162 0.294 0 0.195 0 -0.033 -0.073 -0.02 0.131 0 0.082 -0.003 -0.065 -0.044 0 0.316 0.186 0.054 0.095 -0.062 0 -0.207 0.269 0.09 -0.045

16 1 -0.162 0.294 0 0.093 -0.175 -0.033 -0.073 -0.02 0.131 0 0.082 0 -0.065 -0.088 0 0.316 0.186 0.054 0.095 -0.062 0 -0.203 0.269 0.09 -0.045

16 1 -0.162 0.294 0 0.093 0 -0.033 -0.073 -0.02 0.131 0 0.082 0 -0.065 -0.095 0 0.316 -0.031 0.054 0.095 -0.062 0 -0.203 0.269 0.09 -0.045

16 1 -0.162 0.426 0 0.093 0 -0.033 -0.073 -0.02 0.131 0 0.082 0 -0.065 -0.095 0 0.316 0.186 0.054 0.095 -0.062 0 -0.203 0.269 0.09 -0.045

16 1 -0.162 0.294 0 0.093 0 -0.033 -0.073 -0.02 0.131 0 0.082 -0.003 -0.065 -0.044 0 0.316 0.186 0.054 0.095 -0.062 0 -0.203 0.359 0.09 -0.045

16 1 -0.162 0.294 0 0.093 0 -0.033 -0.073 -0.02 0.131 0 0.082 0 -0.065 -0.095 0 0.316 0.186 0.054 0.095 -0.062 0 -0.203 0.334 0.09 -0.045

16 1 -0.162 0.294 0 0.093 0 -0.033 -0.073 -0.02 0.131 0 -0.028 0 -0.065 -0.131 0 0.5 0.186 0.054 0.095 -0.085 0 -0.203 0 0.09 -0.001

16 1 -0.136 0.294 0 0.093 0 -0.033 -0.073 -0.02 0.131 0 0 0 -0.065 -0.131 0 0.5 0.186 0.054 0.095 -0.085 0 -0.203 0 0.033 -0.001

16 1 -0.162 0.294 0 0.093 0 -0.033 -0.073 -0.02 0.131 0 0.082 0 -0.065 -0.095 0 0.316 0.186 0.054 0.095 -0.062 0 -0.203 0.269 0.09 0.085

16 1 -0.162 0.294 0 0.093 0 -0.033 -0.073 -0.02 0.131 0 0.082 0 -0.065 -0.044 0 0.316 0.035 0.054 0.095 -0.103 0 -0.203 0.269 0.09 -0.045

16 1 -0.162 0.294 0.021 0.195 0 -0.033 -0.073 -0.02 0.131 0 0.082 -0.003 -0.065 -0.044 0.021 0.316 0.186 0.054 0.095 -0.062 0 -0.203 0.269 0.09 -0.045

16 1 -0.136 0.294 0 0.093 0 -0.033 -0.073 -0.02 0.131 0 0 0 -0.065 -0.131 0 0.5 0.186 0.054 0.046 -0.085 -0.035 -0.203 0 0.09 -0.001

16 1 -0.162 0.294 0 0.093 0.076 -0.033 -0.073 -0.02 0.131 0 0 0 -0.065 -0.125 0 0.5 0.186 0.054 0.095 -0.085 0 -0.203 0 0.09 -0.001

17 436 -0.089 0.316 0.018 0.072 0 0 0.121 -0.086 0.008 0 0.252 -0.11 -0.15 -0.067 0.005 -0.002 -0.104 0 0 0.061 -0.153 0.112 0.071 0 0.028

17 747 -0.06 0.37 0.018 0.072 0 0 0.121 -0.086 0.008 0 0.252 -0.11 -0.15 -0.067 0.005 -0.002 -0.104 0 0 0.061 -0.153 0.112 0.093 0 0

17 242 -0.089 0.316 0.018 0.072 0 0 0.121 -0.086 0.008 0 0.252 -0.11 -0.15 -0.067 0.005 -0.002 -0.104 0 0 0.061 -0.153 0.119 0.071 0 0.028

17 235 -0.06 0.257 0.018 0.072 0.121 0 0.236 -0.086 0.008 0 0.252 -0.11 -0.15 -0.208 0.005 -0.002 -0.104 0 -0.031 0.061 -0.118 0.125 0.071 0 0

17 208 -0.06 0.316 0.018 0.072 0 0 0.121 -0.086 0.008 0 0.252 -0.11 -0.15 -0.125 0.005 -0.002 -0.104 0 0 0.061 -0.153 0.166 0.071 0 0

17 177 -0.089 0.316 0.018 0.072 0 0 0.121 -0.086 0.008 0 0.252 -0.11 -0.056 -0.067 0.005 -0.002 -0.104 0 0 0.061 -0.153 0.112 0.071 0 0.028

17 141 -0.055 0.31 0.018 0.072 0 0 0.121 -0.086 0.008 0 0.252 -0.11 -0.15 -0.067 0.005 -0.002 -0.104 0 0 0.061 -0.153 0.112 0.093 0 0

17 126 -0.089 0.316 0.018 0.072 0 0 0.121 -0.086 0.008 0 0.252 -0.11 -0.056 -0.067 0.005 -0.002 -0.104 0 0 0.061 -0.13 0.112 0.071 0 0.028

17 104 -0.055 0.31 0.018 0.078 0 0 0.121 -0.086 0.008 0 0.252 -0.11 -0.15 -0.067 0.005 -0.002 -0.104 0 0 0.061 -0.153 0.112 0.093 0 0

17 99 -0.089 0.316 0.018 0.072 0 0 0.121 -0.086 0.008 0.084 0.252 -0.11 -0.056 -0.067 0.005 -0.002 -0.104 0 0 0.061 -0.153 0.112 0.071 0 0.028

17 97 -0.06 0.37 0.018 0.072 0 0 0.121 -0.049 0.008 0 0.252 -0.11 -0.15 -0.067 0.005 -0.002 -0.104 0 0 0.061 -0.153 0.112 0.093 0 0.006

17 84 -0.06 0.37 0.018 0.072 0 0 0.121 -0.086 0.008 0.121 0.252 -0.11 -0.15 -0.067 0.005 -0.002 -0.104 0 0 0.061 -0.153 0.112 0.093 0 0

17 83 -0.089 0.316 0.018 0.072 -0.077 0 0.121 -0.086 0.008 0 0.252 -0.11 -0.105 -0.067 0.005 -0.002 -0.104 0 -0.028 0.061 -0.153 0.112 0.071 0 0.028

17 26 -0.06 0.37 0.018 0.072 0 0 0.121 -0.086 0.008 0 0.252 -0.046 -0.15 -0.067 0.005 -0.002 -0.104 0 0 0.061 -0.153 0.112 0.093 0 0

17 21 -0.06 0.37 0.018 0.072 0 0 0.121 -0.086 0.008 0 0.252 -0.11 -0.15 -0.067 0.159 -0.002 -0.104 0 0 0.061 -0.153 0.112 0.093 0 0

17 18 -0.089 0.316 0.018 0.072 0 0 0.121 -0.086 0.008 0 0.23 -0.11 -0.056 -0.067 0.005 -0.002 -0.104 0 0 0.061 -0.13 0.112 0.071 0 0.028

17 13 -0.055 0.31 0.018 0.072 0 0 0.121 -0.086 0.008 0 0.252 -0.11 -0.15 -0.067 0.005 -0.002 -0.104 0 0 0.061 -0.153 0.112 0.093 0 -0.037

17 9 -0.06 0.316 0.018 0.072 0 0 0.121 -0.086 0.008 -0.021 0.252 -0.11 -0.15 -0.125 0.005 -0.002 -0.104 0 0 0.061 -0.287 0.166 0.071 -0.082 0

17 9 -0.089 0.316 0.018 0.072 0 0 0.121 -0.193 0.008 0 0.252 -0.11 -0.056 -0.067 0.005 -0.002 -0.104 0 0 0.061 -0.153 0.112 0.071 0 0.028

17 8 -0.089 0.316 0.018 0.072 0 0 0.121 -0.086 0.008 0 0.23 -0.11 -0.056 -0.067 0.005 -0.017 -0.104 0 0 0.061 -0.13 0.112 0.071 0 0.028

17 8 -0.089 0.316 0.018 0.072 0 0 0.121 -0.086 0.008 0.041 0.252 -0.11 -0.15 -0.067 0.005 -0.002 -0.104 0 0 0.061 -0.153 0.112 0.071 0 0.028

17 7 -0.089 0.316 0.018 0.072 0 0 0.211 -0.086 0.008 0 0.252 -0.11 -0.15 -0.067 0.005 -0.002 -0.104 0 0 0.061 -0.153 0.112 0.071 0 0.028

17 7 -0.06 0.257 0.018 0.072 0.121 0 0.236 -0.086 0.008 0 0.252 -0.11 -0.15 -0.208 0.005 -0.002 -0.104 0 -0.031 0.061 -0.118 0.166 0.071 0 0

17 7 -0.089 0.316 0.018 0.072 0 0 0.121 -0.086 0.008 0 0.252 -0.11 -0.105 -0.067 0.005 -0.002 -0.104 0 -0.028 0.061 -0.153 0.112 0.071 0 0.028

17 6 -0.06 0.37 0.018 0.072 0 -0.068 0.121 -0.086 0.008 0 0.252 -0.11 -0.15 -0.067 0.005 -0.002 -0.104 0 0 0.061 -0.153 0.112 0.093 0 0

17 6 -0.06 0.358 0.018 0.072 0 0 0.121 -0.086 0.008 0 0.252 -0.11 -0.15 -0.067 0.005 -0.002 -0.104 0 0 0.061 -0.153 0.112 0.093 0 0

17 6 -0.06 0.316 0.018 0.072 0 0 0.017 -0.086 0.008 0 0.252 -0.11 -0.15 -0.125 0.005 -0.002 -0.104 0 0 0.061 -0.153 0.166 0.071 0 0

17 5 -0.089 0.316 0.018 0.072 0 0 0.121 -0.086 0.008 0 0.252 -0.11 -0.056 -0.067 0.005 -0.002 -0.104 0 0 0.149 -0.153 0.112 0.071 0 0.028

17 4 -0.089 0.316 0.018 0.053 0 0 0.121 -0.086 0.008 0 0.252 -0.11 -0.15 -0.067 0.005 -0.002 -0.104 0 0 0.061 -0.153 0.112 0.071 0 0.028

17 4 -0.06 0.316 0.018 0.072 0 0 0.121 -0.086 0.008 0 0.252 -0.11 -0.15 -0.125 0.005 -0.002 -0.104 0 0 0.061 -0.287 0.166 0.071 0 0

17 4 -0.089 0.43 0.018 0.072 0 0 0.121 -0.086 0.008 0 0.252 -0.11 -0.15 -0.067 0.005 -0.002 -0.104 0 0 0.061 -0.153 0.112 0.071 0 0.028

17 4 -0.142 0.31 0.018 0.078 0 0 0.121 -0.086 0.008 0 0.252 -0.11 -0.15 -0.067 0.005 -0.002 -0.104 0 0 0.061 -0.153 0.112 0.093 0 0

17 4 -0.089 0.316 0.079 0.072 0 0 0.121 -0.086 0.008 0.084 0.252 -0.11 -0.056 -0.067 0.005 -0.002 -0.104 0 0 0.061 -0.153 0.112 0.071 0 0.028

17 4 -0.06 0.37 0.018 0.072 0 0 0.121 -0.049 0.008 0 0.252 -0.11 -0.099 -0.067 0.005 -0.002 -0.104 0 0 0.061 -0.153 0.112 0.093 0 0.006

17 4 -0.089 0.316 0.018 0.072 -0.077 0 0.121 -0.086 0.008 0 0.252 -0.059 -0.105 -0.067 0.005 -0.002 -0.104 0 -0.028 0.061 -0.153 0.112 0.071 0 0.028

17 4 -0.089 0.316 0.018 0.072 0 0 0.121 -0.086 0.008 0 0.252 -0.11 -0.15 -0.067 0.005 -0.002 -0.104 0 -0.084 0.061 -0.153 0.112 0.071 0 0.028

17 3 -0.06 0.316 0.018 0.072 0 0 0.121 -0.086 0.008 -0.021 0.252 -0.11 -0.15 -0.125 0.005 -0.002 -0.104 0 0 0.061 -0.287 0.166 0.071 0 0

17 3 -0.055 0.31 0.018 0.072 0 0 0.121 -0.086 0.008 0 0.252 -0.11 -0.15 -0.067 0.005 0.005 -0.104 0 0 0.061 -0.153 0.112 0.093 0 0

17 3 -0.089 0.316 0.018 0.072 0 0 0.121 -0.086 0.008 0 0.252 -0.11 -0.056 -0.067 0.005 -0.002 -0.104 0 0 0.061 -0.153 0.112 0.071 0 -0.058

17 4 -0.06 0.316 0.018 0.072 0 0.064 0.121 -0.086 0.008 0 0.252 -0.11 -0.15 -0.125 0.005 -0.002 -0.104 0 0 0.061 -0.153 0.166 0.071 0 0

17 3 -0.06 0.37 0.018 0.072 0 0 0.121 -0.049 0.008 0 0.252 -0.11 -0.15 -0.067 0.005 -0.002 -0.104 0 0 0.061 -0.153 0.112 0.093 0 0.144

17 3 -0.06 0.37 0.018 0.072 0 0 0.121 -0.086 0.008 0 0.252 -0.11 -0.15 -0.069 0.005 -0.002 -0.104 0 0 0.071 -0.153 0.112 0.093 0 0

17 2 -0.055 0.31 0.018 0.072 0 0 0.225 -0.086 0.008 0 0.252 -0.11 -0.15 -0.067 0.005 -0.002 -0.104 0 0 0.061 -0.153 0.112 0.093 0 0

17 2 -0.06 0.37 0.018 0.072 0 0 0.121 -0.086 0.008 0 0.252 -0.11 -0.15 -0.067 0.005 -0.002 -0.104 0 0.023 0.061 -0.153 0.112 0.093 0 0

17 2 0.002 0.316 0.018 0.072 0 0 0.121 -0.086 0.008 0 0.252 -0.11 -0.15 -0.067 0.005 -0.002 -0.104 0 0 0.061 -0.153 0.112 0.071 0 0.028

17 2 -0.06 0.37 0.018 0.072 0 0 0.121 -0.086 0.191 0 0.252 -0.11 -0.15 -0.067 0.005 -0.002 -0.104 0 0 0.061 -0.153 0.112 0.093 0 0

17 2 -0.089 0.459 0.018 0.072 0 0 0.121 -0.086 0.008 0 0.252 -0.11 -0.15 -0.067 0.005 -0.002 -0.104 0 0 0.061 -0.153 0.112 0.071 0 0.028

17 2 -0.089 0.316 0.018 0.072 0 0 0.121 -0.086 0.008 0 0.252 -0.11 -0.15 -0.067 0.005 -0.002 -0.104 0 0 0.061 -0.153 0.112 0.071 -0.054 0.028

17 2 -0.06 0.37 0.018 0.072 0 0 0.121 -0.086 0.008 0 0.252 -0.11 -0.15 -0.067 0.005 -0.002 -0.104 0 0 0.057 -0.153 0.112 0.093 0 0

17 2 -0.06 0.316 0.018 0.072 0 0 0.121 -0.086 0.149 -0.021 0.252 -0.11 -0.15 -0.125 0.005 -0.002 -0.104 0 0 0.061 -0.287 0.166 0.071 -0.082 0

17 2 -0.06 0.37 0.018 0.072 0 0 0.121 -0.086 0.008 0 0.252 -0.11 -0.15 -0.067 0.005 -0.002 -0.19 0 0 0.061 -0.153 0.112 0.093 0 0

17 2 -0.06 0.37 0.018 0.072 0 0 0.121 -0.086 0.008 0 0.252 -0.11 -0.15 -0.067 0.159 -0.002 -0.104 -0.059 0 0.061 -0.153 0.112 0.093 0 0

17 2 -0.06 0.257 0.018 0.072 0.121 0 0.236 -0.086 0.008 -0.019 0.252 -0.11 -0.15 -0.208 0.005 -0.002 -0.104 0 -0.031 0.061 -0.118 0.125 0.011 0 0

17 2 -0.06 0.37 0.018 0.072 0 0 0.121 -0.086 0.008 0 0.251 -0.11 -0.15 -0.067 0.005 -0.002 -0.104 0 0 0.061 -0.153 0.112 0.093 0 0

17 2 -0.06 0.37 0.018 0.072 0 0 0.121 -0.086 0.008 0 0.252 -0.11 -0.15 -0.067 0.005 -0.002 -0.104 0 -0.075 0.061 -0.153 0.112 0.093 0 0

17 2 -0.089 0.316 0.018 0.072 0 0 0.121 -0.086 0.008 0.042 0.252 -0.11 -0.15 -0.067 0.005 -0.002 -0.104 0 0 0.061 -0.153 0.112 0.071 0 0.028

17 2 -0.089 0.316 0.018 0.072 0 0 0.121 -0.086 0.008 0 0.252 -0.11 -0.15 -0.067 0.005 -0.002 -0.104 0 0 0.061 -0.153 0.151 0.071 0 0.028

17 2 -0.06 0.37 0.018 0.072 0 0 0.121 -0.086 0.008 0 0.122 -0.11 -0.15 -0.067 0.005 -0.002 -0.104 0 0 0.061 -0.153 0.112 0.093 0 0

17 2 -0.089 0.316 0.018 0.072 0 0 0.121 -0.086 0.008 0 0.252 -0.11 -0.15 -0.067 0.098 -0.002 -0.104 0 0 0.061 -0.153 0.112 0.071 0 0.028

17 2 -0.089 0.316 0.018 0.072 0 0 0.121 -0.086 0.008 0 0.252 -0.11 -0.056 -0.067 0.005 -0.002 -0.104 0 0 0.061 -0.13 0.112 0.071 0 0.09

17 2 -0.055 0.31 0.018 0.072 0 0 0.121 -0.086 0.008 0 0.252 -0.11 -0.15 -0.067 0.005 -0.002 -0.104 -0.013 0 0.061 -0.153 0.112 0.093 0 0

17 2 -0.06 0.37 0.018 0.072 0.147 0 0.121 -0.086 0.008 0 0.252 -0.11 -0.15 -0.067 0.005 -0.002 -0.104 0 0 0.061 -0.153 0.112 0.093 0 0

17 2 -0.06 0.37 0.018 0.072 0 0 0.121 -0.049 0.008 0 0.252 -0.11 -0.15 -0.067 0.005 -0.002 -0.104 0 0 0.061 -0.153 0.112 0.093 0.023 0.006

17 2 -0.06 0.257 0.018 0.072 0.121 0 0.236 -0.086 0.008 0 0.252 -0.11 -0.15 -0.186 0.005 -0.002 -0.104 0 -0.031 0.061 -0.118 0.125 0.071 0 0

17 2 -0.089 0.316 0.018 0.072 0 0 0.121 -0.086 0.008 0 0.252 -0.11 -0.15 -0.067 0.005 -0.002 -0.104 0 -0.015 0.061 -0.153 0.119 0.071 0 0.028

17 2 -0.06 0.37 0.018 0.072 0 0 0.121 -0.049 0.008 0 0.252 -0.11 -0.119 -0.067 0.005 -0.002 -0.104 0 0 0.061 -0.153 0.112 0.093 0 0.006

17 2 -0.055 0.31 0.018 0.078 0 0 0.121 -0.086 0.008 0 0.252 -0.11 -0.15 -0.067 0.005 0.014 -0.104 0 0 0.061 -0.153 0.112 0.093 0 0

17 2 -0.06 0.37 0.018 0.072 0 0 0.121 -0.123 0.008 0 0.252 -0.11 -0.15 -0.067 0.005 -0.002 -0.104 0 0 0.061 -0.153 0.112 0.093 0 0

17 1 -0.055 0.31 0.018 0.072 0 0 0.121 -0.086 0.008 0 0.252 -0.11 -0.15 -0.067 0.005 -0.002 -0.104 0 0 0.061 -0.153 0.112 0.093 0.117 0

17 1 -0.06 0.37 0.018 0.072 0 0 0.121 -0.086 0.008 0 0.252 -0.11 -0.15 -0.067 0.005 -0.002 -0.104 0 0 0.061 -0.153 0.112 0.093 0.034 0

17 1 -0.06 0.37 0.018 0.072 0 0 0.121 -0.086 0.008 0 0.252 -0.11 -0.15 -0.067 0.005 -0.002 -0.104 0 0.015 0.061 -0.153 0.112 0.093 0 0

17 1 -0.06 0.316 0.018 0.072 0 0 0.121 -0.086 0.008 -0.021 0.252 -0.11 -0.15 -0.125 0.017 -0.002 -0.104 0 0 0.061 -0.287 0.166 0.071 0 0

17 1 -0.055 0.31 0.018 0.137 0 0 0.121 -0.086 0.008 0 0.252 -0.11 -0.15 -0.067 0.005 -0.002 -0.104 0 0 0.061 -0.153 0.112 0.093 0 0

17 1 -0.06 0.257 0.018 0.072 0.121 0 0.236 -0.086 0.008 0 0.252 -0.234 -0.15 -0.208 0.005 -0.002 -0.104 0 -0.031 0.061 -0.118 0.125 0.071 0 0

17 1 -0.06 0.316 0.018 0.072 0 0 0.121 -0.086 -0.039 0 0.252 -0.11 -0.15 -0.125 0.005 -0.002 -0.104 0 0 0.061 -0.153 0.166 0.071 0 0

17 1 -0.089 0.316 0.018 0.041 0 0 0.121 -0.086 0.008 0 0.252 -0.11 -0.15 -0.067 0.005 -0.002 -0.104 0 0 0.061 -0.153 0.119 0.071 0 0.028

17 1 -0.218 0.316 0.018 0.072 0 0 0.121 -0.086 0.008 0 0.252 -0.11 -0.15 -0.125 0.005 -0.002 -0.104 0 0 0.061 -0.153 0.166 0.071 0 0

17 1 -0.06 0.37 0.018 0.072 0 0 0.121 -0.086 0.008 0 0.252 -0.11 -0.15 -0.067 -0.057 -0.002 -0.104 0 0 0.061 -0.153 0.112 0.093 0 0

17 1 -0.06 0.37 0.018 0.072 0 0 0.121 -0.086 0.008 0 0.252 -0.046 -0.15 -0.067 0.005 -0.002 -0.104 0 0 0.061 -0.153 0.112 0.093 0.123 0

17 1 -0.06 0.316 0.018 0.072 0 0 0.121 -0.086 0.008 0 0.252 -0.11 -0.15 -0.125 0.005 -0.002 -0.104 0 0 0.061 -0.153 0.166 0.071 0.158 0

17 1 -0.06 0.37 0.018 0.072 0 0 0.121 -0.086 0.008 0 0.252 -0.11 -0.15 -0.067 0.005 -0.002 -0.104 0 0 0.061 -0.041 0.112 0.093 0 0

17 1 -0.06 0.316 0.018 0.072 0 0 0.121 -0.086 0.008 -0.004 0.252 -0.11 -0.15 -0.125 0.005 -0.002 -0.104 0 0 0.061 -0.153 0.166 0.071 0 0

17 1 -0.055 0.31 0.018 0.078 0 0 0.121 -0.115 0.008 0 0.252 -0.11 -0.15 -0.067 0.005 -0.002 -0.104 0 0 0.061 -0.153 0.112 0.093 0 0

17 1 -0.06 0.37 0.018 0.092 0 0 0.121 -0.086 0.008 0 0.252 -0.11 -0.15 -0.067 0.159 -0.002 -0.104 0 0 0.061 -0.153 0.112 0.093 0 0

17 1 -0.158 0.316 0.018 0.072 0 0 0.121 -0.086 0.008 0 0.252 -0.11 -0.056 -0.067 0.005 -0.002 -0.104 0 0 0.061 -0.153 0.112 0.071 0 0.028

17 1 -0.06 0.257 0.018 0.072 0.121 -0.066 0.236 -0.086 0.008 0 0.252 -0.11 -0.15 -0.208 0.005 -0.002 -0.104 0 -0.031 0.061 -0.118 0.125 0.071 0 0

17 1 -0.089 0.316 0.018 0.041 0 0 0.121 -0.086 0.008 0 0.252 -0.11 -0.15 -0.067 0.005 -0.002 -0.104 -0.067 0 0.061 -0.153 0.119 0.071 0 0.028

17 1 -0.06 0.37 0.018 0.072 0 0 0.121 -0.086 0.008 0 0.252 -0.11 -0.172 -0.067 0.005 -0.002 -0.104 0 0 0.061 -0.153 0.112 0.093 0 0

17 1 -0.089 0.316 0.018 0.072 0 0 0.121 -0.086 0.008 0 0.252 -0.11 -0.056 -0.067 0.005 -0.14 -0.104 0 0 0.061 -0.13 0.112 0.071 0 0.028

17 1 -0.089 0.316 0.018 0.072 0 0 0.121 -0.086 0.008 0 0.23 -0.11 -0.056 -0.067 0.005 -0.002 -0.104 0 0 0.061 -0.13 0.112 0.071 0.204 0.028

17 1 -0.06 0.257 0.018 0.072 0.121 0 0.236 -0.086 0.008 -0.05 0.252 -0.11 -0.15 -0.208 0.005 -0.002 -0.104 0 -0.031 0.061 -0.118 0.125 0.071 0 0

17 1 -0.06 0.37 0.018 0.072 0 0 0.121 -0.086 0.008 0 0.182 -0.11 -0.15 -0.067 0.005 -0.002 -0.104 0 0 0.061 -0.153 0.112 0.093 0 0

17 1 -0.06 0.37 0.018 0.072 0 0 0.121 -0.086 0.008 0 0.252 -0.11 -0.15 -0.067 0.005 -0.002 -0.104 0 0 0.061 -0.153 0.112 0.093 0 -0.16

17 1 -0.06 0.37 0.018 0.072 0 0 0.121 -0.049 0.008 0 0.252 -0.11 -0.15 -0.067 0.005 -0.002 -0.104 0 0 0.061 -0.153 0.112 0.228 0 0.006

17 1 -0.06 0.37 0.018 0.072 0 0 0.121 -0.049 0.008 0 0.252 -0.295 -0.15 -0.067 0.005 -0.002 -0.104 0 0 0.061 -0.153 0.112 0.093 0 0.006

17 1 -0.055 0.31 0.018 0.078 0 0 0.121 -0.086 0.008 0 0.252 -0.11 -0.199 -0.067 0.005 -0.002 -0.104 0 0 0.061 -0.153 0.112 0.01 0 0

17 1 -0.089 0.316 0.018 0.072 0 -0.179 0.121 -0.086 0.008 0 0.252 -0.11 -0.15 -0.067 0.005 -0.002 -0.104 0 0 0.061 -0.153 0.119 0.071 0 0.028

17 1 -0.06 0.257 0.018 0.072 0.121 0 0.236 -0.086 0.008 0 0.252 -0.198 -0.15 -0.208 0.005 -0.002 -0.104 0 -0.031 0.061 -0.118 0.125 0.071 0 0

17 1 -0.06 0.37 0.018 0.072 0 0 0.121 -0.086 0.008 0 0.252 -0.11 -0.15 -0.067 0.005 -0.002 -0.104 0 0 0.061 -0.153 0.112 0.156 0 0

17 1 -0.06 0.37 0.018 0.072 0 0 0.121 -0.086 0.008 0 0.252 -0.11 -0.15 -0.067 0.005 -0.002 -0.104 0 0 0.061 -0.153 0.112 0.093 -0.207 0

17 1 -0.089 0.316 0.018 0.072 0 -0.073 0.121 -0.086 0.008 0 0.252 -0.11 -0.15 -0.067 0.005 -0.002 -0.104 0 0 0.061 -0.153 0.119 0.071 0 0.028

17 1 -0.089 0.316 0.018 0.072 0 0 0.121 -0.086 0.008 0.084 0.252 -0.11 -0.056 -0.067 0.005 -0.002 -0.104 0 0 0.061 -0.153 0.144 0.071 0 0.028

17 1 -0.089 0.316 -0.077 0.072 0 0 0.121 -0.086 0.008 0 0.252 -0.11 -0.15 -0.067 0.005 -0.002 -0.104 0 0 0.061 -0.153 0.119 0.071 0 0.028

17 1 -0.06 0.37 0.018 0.072 0 0 0.121 -0.086 0.008 0 0.252 -0.11 -0.15 -0.067 0.005 -0.002 -0.104 0 0 0.061 -0.153 0.112 0.093 0 -0.116

17 1 -0.089 0.316 0.018 0.072 0 0 0.121 -0.086 0.008 0 0.252 -0.11 -0.035 -0.067 0.005 -0.002 -0.104 0 0 0.061 -0.13 0.112 0.071 0 0.028

17 1 -0.089 0.316 0.018 0.072 0 0 0.121 -0.086 0.008 0.084 0.252 -0.11 -0.056 -0.067 0.005 -0.002 -0.104 0 0 0.056 -0.153 0.112 0.071 0 0.028

17 1 -0.089 0.316 0.018 0.072 0 0 0.121 -0.086 0.008 0 0.252 -0.11 -0.15 -0.067 0.005 -0.002 -0.017 0 0 0.061 -0.153 0.119 0.071 0 0.028

17 1 -0.089 0.316 0.018 0.072 0 0 0.121 -0.086 0.008 0 0.252 -0.11 -0.15 -0.067 0.005 -0.002 -0.104 0 0 0.061 -0.153 0.112 0.07 0 0.028

17 1 -0.06 0.37 -0.107 0.072 0 0 0.121 -0.086 0.008 0 0.252 -0.11 -0.15 -0.067 0.005 -0.002 -0.104 0 0 0.061 -0.153 0.112 0.093 0 0

17 1 -0.089 0.316 0.018 0.072 0 0 0.121 -0.086 0.008 0 0.252 -0.11 -0.15 -0.067 0.005 -0.002 -0.104 0 0 0.061 -0.153 0.119 0.071 0 -0.086

17 1 -0.06 0.37 0.018 0.072 0 0 0.121 -0.086 0.008 0 0.252 -0.11 -0.15 -0.067 0.005 -0.002 -0.104 0 -0.119 0.061 -0.153 0.112 0.093 0 0

17 1 -0.089 0.316 0.018 0.072 0 0 0.121 -0.086 0.008 0 0.252 -0.11 -0.15 -0.067 0.005 -0.002 -0.104 -0.087 0 0.061 -0.153 0.112 0.071 0 0.028

17 1 -0.06 0.37 0.018 0.072 0 0 0.121 -0.086 0.008 0 0.252 -0.003 -0.15 -0.067 0.005 -0.002 -0.104 0 0 0.061 -0.153 0.112 0.093 0 0

17 1 -0.06 0.37 0.018 0.072 0 0 0.121 -0.086 0.008 0 0.252 -0.11 -0.15 -0.067 0.005 -0.002 -0.104 0 0 0.061 -0.153 -0.048 0.093 0 0

17 1 -0.089 0.316 0.018 0.072 0 0 0.121 -0.086 0.008 0.084 0.252 -0.11 -0.056 -0.067 0.005 -0.002 -0.104 0 0.068 0.061 -0.153 0.112 0.071 0 0.028

17 1 -0.06 0.316 0.018 0.072 0 0 0.121 -0.086 0.008 -0.021 0.252 -0.11 -0.238 -0.125 0.005 -0.002 -0.104 0 0 0.061 -0.287 0.166 0.071 -0.082 0

17 1 -0.06 0.37 0.018 0.072 0 0 0.121 -0.086 0.008 0 0.252 -0.145 -0.15 -0.067 0.005 -0.002 -0.104 0 0 0.061 -0.153 0.112 0.093 0 0

17 1 -0.055 0.31 0.018 0.078 0 0 0.121 -0.086 0.008 0 0.252 -0.11 -0.15 -0.067 0.005 -0.002 -0.104 0 0 0.061 -0.153 0.112 0.093 -0.002 0

17 1 -0.06 0.37 0.018 0.072 0 0 0.121 -0.086 -0.104 0 0.252 -0.11 -0.15 -0.067 0.005 -0.002 -0.104 0 0 0.061 -0.153 0.112 0.093 0 0

17 1 -0.089 0.316 0.018 0.072 0 0 0.121 -0.086 0.008 0 0.252 -0.11 -0.15 -0.067 0.005 0.002 -0.104 0 0 0.061 -0.153 0.112 0.071 0 0.028

17 1 -0.089 0.316 0.018 0.045 0 0 0.121 -0.086 0.008 0 0.252 -0.11 -0.15 -0.067 0.005 -0.002 -0.104 0 0 0.061 -0.153 0.119 0.071 0 0.028

17 1 -0.089 0.316 0.018 0.072 0 0 0.121 -0.086 0.008 0 0.252 -0.11 -0.15 -0.067 0.005 -0.002 -0.104 0 0 0.061 -0.153 0.269 0.071 0 0.028

17 1 -0.032 0.316 0.018 0.072 0 0 0.121 -0.086 0.008 0 0.252 -0.11 -0.056 -0.067 0.005 -0.002 -0.104 0 0 0.061 -0.153 0.112 0.071 0 0.028

17 1 -0.06 0.37 0.018 0.072 0 0 0.121 -0.086 0.008 0 0.252 -0.11 -0.15 -0.088 0.005 -0.002 -0.104 0 0 0.061 -0.153 0.112 0.093 0 0

18 340 0 -0.022 -0.129 0.022 0 0.158 -0.007 0 -0.054 -0.124 0.014 -0.044 -0.128 0 0 -0.119 0.046 0 -0.094 0.011 0.035 0 -0.042 -0.426 0.013

18 300 0 -0.022 -0.129 0.022 0 0.158 -0.007 0 -0.054 -0.008 0.014 -0.094 -0.128 0 0 -0.119 0.063 0 -0.094 0.011 0.035 0 -0.042 -0.426 0.013

18 223 0 -0.022 -0.156 0.022 0 0.158 -0.007 0 -0.054 0.106 0.014 -0.094 -0.073 0 0 -0.119 0.175 0 -0.094 0.011 0.035 0 -0.042 -0.426 0.013

18 197 0 -0.022 -0.129 0.022 0 0.158 -0.007 0 -0.129 -0.008 -0.054 -0.094 -0.128 0 0 -0.119 0.063 0 -0.094 0.011 0.035 0 -0.042 -0.426 0.013

18 178 0 -0.022 -0.129 0.022 0 0.158 -0.007 0 -0.129 -0.008 -0.096 -0.094 -0.128 0 0 -0.119 0.063 0 -0.094 0.011 0.035 0 -0.042 -0.426 0.013

18 168 0 -0.022 -0.129 0.022 0 0.158 -0.007 0 -0.054 -0.008 0.014 -0.094 -0.128 0.088 0 -0.119 0.063 0 -0.094 0.011 0.035 0 -0.042 -0.426 0.013

18 195 0 -0.022 -0.129 0.022 0 0.158 -0.007 0 -0.054 -0.008 0.014 -0.094 -0.128 0 0 -0.119 0.063 0 -0.094 0.011 0.035 0 -0.042 -0.338 0.013

18 109 0 -0.022 -0.129 0.022 0 0.158 -0.007 0 -0.129 -0.008 0.034 -0.094 -0.128 0 0 -0.119 0.063 0 -0.094 0.011 0.035 0 -0.042 -0.426 0.013

18 109 0 -0.022 -0.129 0.022 0 0.158 -0.007 0 -0.054 -0.124 0.014 0.036 -0.128 0 0 -0.119 0.046 0 -0.094 0.011 0.035 0 -0.042 -0.426 0.013

18 100 0 -0.022 -0.129 0.022 0 0.158 -0.007 0 -0.054 -0.008 0.014 -0.094 -0.128 0 0 -0.119 0.066 0 -0.094 0.011 0.035 0 -0.042 -0.338 0.013

18 91 0 -0.022 -0.129 -0.098 0 0.158 -0.007 0 -0.054 -0.008 0.014 -0.094 -0.128 0 0 -0.119 0.063 0 -0.094 0.011 0.035 0 -0.042 -0.338 0.013

18 85 0 -0.022 -0.129 0.022 0 0.158 -0.027 0 -0.129 -0.008 0.034 -0.094 -0.128 0 0 -0.119 0.063 0 -0.094 0.011 0.035 0 -0.042 -0.426 0.013

18 75 0 -0.022 -0.129 0.022 0 0.158 -0.007 0 -0.129 -0.008 -0.096 -0.094 -0.128 0 0 -0.119 0.063 -0.067 -0.094 0.011 0.035 0 -0.042 -0.426 0.013

18 39 0 -0.022 -0.129 0.022 0 0.158 -0.007 0.018 -0.054 -0.008 0.014 -0.094 -0.128 0 0 -0.119 0.063 0 -0.094 0.011 0.035 0 -0.042 -0.426 0.013

18 32 0 -0.022 -0.129 0.022 0 0.158 -0.007 0 -0.054 -0.008 0.014 -0.094 -0.128 0.114 0 -0.119 0.063 0 -0.094 0.011 0.035 0 -0.042 -0.426 0.013

18 32 0 -0.022 -0.129 0.022 0 0.158 -0.097 0 -0.129 -0.008 0.034 -0.094 -0.128 0 0 -0.119 0.063 0 -0.094 0.011 0.035 0 -0.042 -0.426 0.013

18 22 0 -0.022 -0.129 0.022 0 0.158 -0.007 0 -0.155 -0.008 0.014 -0.094 -0.128 0 0 -0.119 0.063 0 -0.094 0.011 -0.155 0 -0.042 -0.338 0.013

18 21 0 -0.022 -0.259 0.022 0 0.158 -0.063 0 -0.054 -0.016 0.014 -0.094 -0.128 0 0 -0.119 0.063 0 -0.094 0.011 0.035 0 -0.042 -0.338 0.013

18 18 0 -0.022 -0.173 0.022 0 0.158 -0.007 0 -0.129 -0.008 -0.096 -0.094 -0.128 0 0 -0.119 0.063 0 -0.094 0.011 0.035 -0.128 -0.042 -0.426 0.013

18 17 0 -0.022 -0.129 0.022 -0.08 0.158 -0.007 0 -0.054 -0.008 0.014 -0.094 -0.128 0 0 -0.119 0.063 0 -0.094 0.011 0.035 0 -0.042 -0.338 0.013

18 11 0 -0.022 -0.129 0.022 0 0.158 -0.007 0 -0.054 -0.008 0.014 -0.094 -0.01 0 0 -0.119 0.063 0 -0.094 0.011 0.035 0 -0.042 -0.338 0.013

18 11 0 -0.022 -0.129 0.053 0 0.158 -0.007 0 -0.129 -0.008 -0.096 -0.094 -0.128 0 0 -0.119 0.063 0 -0.094 0.011 0.035 0 -0.042 -0.426 0.013

18 9 0 -0.022 -0.129 0.022 0 0.158 -0.007 0 -0.054 -0.119 0.014 -0.094 -0.128 0 0 -0.119 0.066 0 -0.094 0.011 0.035 0 -0.042 -0.338 0.013

18 9 0 -0.022 -0.129 0.053 0 0.158 -0.007 0 -0.054 -0.008 0.014 -0.094 -0.128 0 0 -0.119 0.063 0 -0.094 0.011 0.035 0 -0.042 -0.426 0.013

18 9 0 -0.022 -0.108 0.022 0 0.158 -0.007 0 -0.054 -0.008 0.014 -0.094 -0.128 0 0 -0.119 0.063 0 -0.094 0.011 0.035 0 -0.042 -0.338 0.013

18 8 0 -0.022 -0.156 0.022 0 0.09 -0.007 0 -0.054 0.106 0.014 -0.094 -0.073 0 0 -0.119 0.175 0 -0.094 0.011 0.035 0 -0.042 -0.426 0.013

18 7 0 -0.022 -0.129 0.022 0 0.158 -0.007 0 -0.054 -0.008 0.014 -0.204 -0.128 0 0 -0.119 0.063 0 -0.094 0.011 0.035 0 -0.042 -0.426 0.013

18 7 0 -0.022 -0.129 0.022 0 0.158 -0.007 0 -0.054 -0.008 0.21 -0.094 -0.128 0 0 -0.119 0.063 0 -0.094 0.011 0.035 0 -0.042 -0.426 0.013

18 7 0 -0.022 -0.129 0.022 0 0.158 -0.007 0 -0.129 -0.008 -0.096 -0.094 -0.128 0 0 -0.119 0.063 -0.067 -0.094 0.011 0.035 0 -0.144 -0.426 0.013

18 6 0 -0.022 -0.129 0.022 0 0.158 -0.007 0 -0.054 -0.008 0.014 -0.094 -0.128 0.088 -0.058 -0.119 0.063 0 -0.094 0.011 0.035 0 -0.042 -0.426 0.013

18 6 -0.074 -0.022 -0.129 0.022 0 0.158 -0.007 0 -0.054 -0.008 0.014 -0.094 -0.128 0 0 -0.119 0.063 0 -0.094 0.011 0.035 0 -0.042 -0.338 0.013

18 6 0 -0.022 -0.129 0.022 0 0.158 -0.007 0.015 -0.054 -0.008 0.014 -0.094 -0.128 0 0 -0.119 0.063 0 -0.094 0.011 0.035 0 -0.042 -0.338 0.013

18 6 0 -0.022 -0.156 0.022 0 0.158 -0.007 0 -0.054 0.106 0.014 -0.094 -0.073 0 0 -0.119 0.175 0 -0.094 -0.078 0.035 0 -0.042 -0.426 0.013

18 6 0 -0.022 -0.129 -0.098 0 0.158 -0.007 0 -0.054 -0.008 0.014 -0.094 -0.128 -0.031 0 -0.119 0.063 0 -0.094 0.011 0.035 0 -0.042 -0.338 0.013

18 5 0 -0.022 -0.129 0.022 0 0.158 0.035 0 -0.129 -0.008 -0.096 -0.094 -0.128 0 0 -0.119 0.063 0 -0.094 0.011 0.035 0 -0.042 -0.426 0.013

18 5 0 -0.022 -0.129 0.022 0 0.158 -0.007 0 -0.155 -0.008 0.014 -0.094 -0.128 0 0 -0.119 0.063 0 -0.094 0.011 0.035 0 -0.042 -0.338 0.013

18 5 0 -0.022 -0.129 0.022 0 0.158 -0.007 0 -0.129 -0.008 -0.096 -0.094 -0.128 0 0 -0.119 0.063 0 -0.203 0.011 0.035 0 -0.042 -0.426 0.013

18 5 0 -0.022 -0.129 0.022 0 0.158 -0.007 0 -0.054 -0.008 0.014 -0.094 -0.128 0 0 -0.119 0.063 0 -0.121 0.011 0.035 0 -0.042 -0.338 0.013

18 5 0 -0.022 -0.167 0.022 0 0.158 -0.007 0 -0.054 -0.008 0.014 -0.094 -0.128 0 0 -0.119 0.063 0 -0.094 0.011 0.035 0 -0.042 -0.426 0.013

18 4 0 -0.022 -0.129 0.022 0 0.158 -0.007 0 -0.054 -0.008 0.014 -0.094 -0.128 0.088 0 -0.119 0.042 0 -0.094 0.011 0.035 0 -0.042 -0.426 0.013

18 4 0 -0.022 -0.129 0.122 0 0.158 -0.007 0 -0.054 -0.124 0.014 -0.044 -0.128 0 0 -0.119 0.046 0 -0.094 0.011 0.035 0 -0.042 -0.426 0.013

18 4 0 -0.022 -0.129 0.022 0 0.158 -0.007 0 -0.054 -0.008 0.014 -0.156 -0.128 0 0 -0.119 0.063 0 -0.094 0.011 0.035 0 -0.042 -0.426 0.013

18 4 0 -0.022 -0.129 0.022 0 0.158 -0.007 0 -0.129 -0.008 -0.054 -0.094 -0.128 0 0 -0.119 0.063 0 -0.094 0.011 0.035 0 -0.042 -0.426 0.049

18 4 0 -0.022 -0.129 0.022 0 0.158 -0.007 0 -0.054 -0.008 0.014 -0.094 -0.128 0.088 0 -0.119 0.063 0 0.005 0.011 0.035 0 -0.042 -0.426 0.013

18 4 0 -0.022 -0.156 0.022 0 0.158 -0.007 0 -0.054 0.103 0.014 -0.094 -0.073 0 0 -0.119 0.175 0 -0.094 0.011 0.035 0 -0.042 -0.426 0.013

18 3 0 -0.022 -0.129 0.022 0 0.158 -0.007 -0.043 -0.054 -0.124 0.014 0.036 -0.128 0 0 -0.119 0.046 0 -0.094 0.011 0.035 0 -0.042 -0.426 0.013

18 3 0 -0.022 -0.156 0.022 0 0.158 -0.007 0 -0.054 0.106 0.014 -0.094 -0.073 0 0 -0.119 0.175 0 -0.094 0.011 0.126 0 -0.042 -0.426 0.013

18 3 0 -0.022 -0.129 0.022 0 0.158 -0.007 0 -0.129 -0.008 -0.054 -0.094 -0.128 0 0 -0.119 0.063 0 -0.094 0.011 0.123 0 -0.042 -0.426 0.013

18 3 0 -0.022 -0.129 0.022 0 0.158 -0.007 0 -0.054 -0.008 0.014 -0.094 -0.128 -0.003 0 -0.119 0.063 0 -0.094 0.011 0.035 0 -0.042 -0.426 0.013

18 2 0 -0.022 -0.129 -0.047 0 0.158 -0.007 0 -0.054 -0.008 0.014 -0.094 -0.128 0 0 -0.119 0.063 0 -0.094 0.011 0.035 0 -0.042 -0.426 0.013

18 2 0 -0.022 -0.129 0.022 0 0.158 -0.007 0.018 -0.054 -0.008 0.014 -0.094 -0.128 0 0 -0.119 0.063 -0.042 -0.094 0.011 0.035 0 -0.042 -0.426 0.013

18 2 0 -0.022 -0.129 0.022 -0.049 0.158 -0.007 0 -0.129 -0.008 -0.096 -0.094 -0.128 0 0 -0.119 0.063 0 -0.094 0.011 0.035 0 -0.042 -0.426 0.013

18 2 0 -0.022 -0.129 0.022 0 0.158 0.035 -0.115 -0.129 -0.008 -0.096 -0.094 -0.128 0 0 -0.119 0.063 0 -0.094 0.011 0.035 0 -0.042 -0.426 0.013

18 2 0 -0.022 -0.129 0.022 0 0.158 -0.007 0 -0.129 -0.008 0.034 -0.094 -0.128 0 -0.078 -0.119 0.063 0 -0.094 0.011 0.035 0 -0.042 -0.426 0.013

18 2 0 -0.022 -0.156 0.022 0 0.158 -0.007 0 -0.054 0.106 0.014 -0.094 -0.073 0 0 -0.119 0.175 0 -0.094 0.011 0.171 0 -0.042 -0.426 0.013

18 2 0 0.078 -0.129 0.022 0 0.158 -0.007 0 -0.155 -0.008 0.014 -0.094 -0.128 0 0 -0.119 0.063 0 -0.094 0.011 0.035 0 -0.042 -0.338 0.013

18 2 0 -0.022 -0.156 0.022 0 0.158 -0.007 0 -0.054 0.106 0.014 -0.094 -0.073 0.026 0 -0.119 0.175 0 -0.094 0.011 0.035 0 -0.042 -0.426 0.013

18 2 0 -0.022 -0.129 0.022 0 0.158 -0.007 0 -0.054 -0.008 -0.197 -0.094 -0.128 0 0 -0.119 0.066 0 -0.094 0.011 0.035 0 -0.042 -0.338 0.013

18 2 0 -0.022 -0.129 0.022 0 0.158 -0.007 0 -0.054 -0.124 0.014 -0.044 -0.128 0 0 -0.119 0.046 0 -0.094 0.011 0.035 0 -0.042 -0.468 0.013

18 2 0 -0.022 -0.156 0.022 0 0.158 -0.007 0 -0.054 0.106 0.014 -0.091 -0.073 0 0 -0.119 0.175 0 -0.094 0.011 0.035 0 -0.042 -0.426 0.013

18 2 0 -0.022 -0.129 0.022 0 0.158 -0.007 0 -0.168 -0.124 0.014 -0.044 -0.128 0 0 -0.119 0.046 0 -0.094 0.011 0.035 0 -0.042 -0.426 0.013

18 2 0 0.01 -0.129 0.022 0 0.158 -0.007 0 -0.129 -0.008 -0.096 -0.094 -0.128 0 0 -0.119 0.063 -0.067 -0.094 0.011 0.035 0 -0.042 -0.426 0.013

18 2 0 -0.131 -0.129 0.022 0 0.158 -0.007 0 -0.054 -0.124 0.014 -0.044 -0.128 0 0 -0.119 0.046 0 -0.094 0.011 0.035 0 -0.042 -0.426 0.013

18 2 0 -0.022 -0.129 0.022 0 0.158 -0.007 0 -0.129 -0.052 -0.096 -0.094 -0.128 0 0 -0.119 0.063 0 -0.094 0.011 0.035 0 -0.042 -0.426 0.013

18 2 0 -0.075 -0.129 0.022 0 0.158 -0.007 0 -0.054 -0.008 0.014 -0.094 -0.128 0.114 0 -0.119 0.063 0 -0.094 0.011 0.035 0 -0.042 -0.426 0.013

18 2 0 -0.022 -0.129 0.022 0 0.158 0.069 0 -0.054 -0.008 0.014 -0.094 -0.128 0 0 -0.119 0.066 0 -0.094 0.011 0.035 0 -0.042 -0.338 0.013

18 2 0 -0.022 -0.129 0.022 0 0.158 -0.007 0 -0.054 -0.008 0.014 -0.094 -0.128 0 0 -0.119 0.063 -0.132 -0.094 0.011 0.035 0 -0.042 -0.426 0.013

18 2 0 -0.022 -0.129 0.022 -0.08 0.158 -0.007 0 -0.047 -0.008 0.014 -0.094 -0.128 0 0 -0.119 0.063 0 -0.094 0.011 0.035 0 -0.042 -0.338 0.013

18 2 0 -0.022 -0.153 0.022 0 0.158 -0.007 0 -0.129 -0.008 -0.096 -0.094 -0.128 0 0 -0.119 0.063 -0.067 -0.094 0.011 0.035 0 -0.042 -0.426 0.013

18 2 0 -0.022 -0.156 0.022 0 0.158 -0.007 0 -0.054 0.106 0.014 -0.094 -0.073 0 0 -0.119 0.175 0 -0.068 0.011 0.035 0 -0.042 -0.426 0.013

18 2 0 -0.022 -0.129 0.128 0 0.158 -0.007 0 -0.129 -0.008 -0.096 -0.094 -0.128 0 0 -0.119 0.063 0 -0.094 0.011 0.035 0 -0.042 -0.426 0.013

18 2 0 -0.075 -0.129 0.022 0 0.158 -0.007 0 -0.129 -0.008 -0.096 -0.094 -0.128 0 0 -0.119 0.063 0 -0.094 0.011 0.035 0 -0.042 -0.426 0.013

18 2 0 -0.022 -0.129 0.022 0 0.158 -0.007 0 -0.054 -0.008 0.014 -0.094 -0.128 0.088 0 -0.119 0.063 0 -0.094 0.011 0.035 0 -0.042 -0.543 0.013

18 2 0 -0.022 -0.129 0.022 0 0.158 -0.007 0 -0.054 -0.008 0.014 -0.094 -0.128 0 0 -0.119 0.063 0 -0.094 0.011 -0.045 0 -0.042 -0.338 0.013

18 2 0 -0.022 -0.129 0.022 0 0.158 -0.007 0 -0.129 -0.008 -0.054 -0.094 -0.128 0 0 -0.119 0.063 0.083 -0.094 0.011 0.035 0 -0.042 -0.426 0.013

18 2 0 -0.022 -0.129 0.022 0 0.158 -0.007 0 -0.054 -0.008 0.014 -0.094 -0.023 0.088 0 -0.119 0.063 0 -0.094 0.011 0.035 0 -0.042 -0.426 0.013

18 2 0.079 -0.022 -0.129 0.022 0 0.158 -0.007 0 -0.129 -0.008 -0.096 -0.094 -0.128 0 0 -0.119 0.063 0 -0.094 0.011 0.035 0 -0.042 -0.426 0.013

18 2 0 -0.022 -0.129 0.022 0 0.158 -0.007 0 -0.054 -0.008 0.014 -0.094 -0.128 0 0 -0.117 0.066 0 -0.094 0.011 0.035 0 -0.042 -0.338 0.013

18 2 0 -0.022 -0.129 0.022 0 0.158 -0.008 0 -0.054 -0.008 0.014 -0.094 -0.128 0.088 0 -0.119 0.063 0 -0.094 0.011 0.035 0 -0.042 -0.426 0.013

18 2 0 -0.022 -0.129 0.022 0 0.158 -0.007 0 -0.054 -0.124 0.014 -0.044 -0.128 0 0 -0.119 0.046 0 -0.094 0.011 0.035 0 -0.017 -0.426 0.013

18 2 0 -0.022 -0.156 0.022 0 0.158 -0.007 0 -0.054 0.106 0.014 -0.094 -0.073 0 0 -0.119 0.175 -0.095 -0.094 0.011 0.035 0 -0.042 -0.426 0.013

18 2 0 -0.022 -0.129 0.022 0 -0.042 -0.007 0 -0.054 -0.008 0.014 -0.094 -0.128 0 0 -0.119 0.066 0 -0.094 0.011 0.035 0 -0.042 -0.338 0.013

18 1 0 -0.022 -0.129 0.022 0 0.158 -0.007 0 -0.129 -0.008 0.034 -0.094 -0.128 0 0 -0.119 0.063 0.001 -0.094 0.011 0.035 0 -0.042 -0.426 0.013

18 1 0 -0.022 -0.129 0.022 0 0.158 -0.007 0 -0.054 -0.008 0.014 -0.094 -0.128 0.088 0 -0.119 0.063 0 -0.094 0.011 0.035 0 -0.042 -0.426 -0.117

18 1 0 -0.022 -0.129 0.022 0 0.158 -0.007 0 -0.054 -0.119 0.014 -0.094 -0.128 -0.238 0 -0.119 0.066 0 -0.094 0.011 0.035 0 -0.042 -0.338 0.013

18 1 0 -0.022 -0.129 0.022 0 0.158 -0.11 0 -0.129 -0.008 -0.096 -0.094 -0.128 0 0 -0.119 0.063 0 -0.094 0.011 0.035 0 -0.042 -0.426 0.013

18 1 0 -0.022 -0.129 0.022 0 0.158 -0.007 0 -0.054 -0.008 -0.13 -0.094 -0.128 0 0 -0.119 0.063 0 -0.094 0.011 0.035 0 -0.042 -0.426 0.013

18 1 0 -0.022 -0.129 0.022 0 0.158 -0.007 0 -0.054 -0.008 0.014 -0.094 0.04 0 0 -0.119 0.063 0 -0.094 0.011 0.035 0 -0.042 -0.338 0.013

18 1 0 -0.022 -0.129 0.022 0 0.158 -0.007 0 -0.054 -0.008 -0.13 -0.094 -0.128 0 0 -0.119 0.063 0 -0.094 0.011 0.035 0 -0.102 -0.426 0.013

18 1 0 -0.022 -0.156 0.022 0 0.158 -0.007 0 -0.054 0.106 0.014 -0.094 -0.073 0 0 -0.119 0.175 0 -0.094 0.011 0.074 0 -0.042 -0.426 0.013

18 1 0 -0.022 -0.129 0.022 0 0.158 -0.007 0 -0.054 -0.008 0.014 -0.094 -0.079 0 0 -0.119 0.063 0 -0.094 0.011 0.035 0 -0.042 -0.338 0.013

18 1 0 -0.022 -0.129 0.022 0.015 0.158 -0.007 0 -0.054 -0.124 0.014 -0.044 -0.128 0 0 -0.119 0.046 0 -0.094 0.011 0.035 0 -0.042 -0.426 0.013

18 1 0 -0.022 -0.129 0.022 -0.103 0.158 -0.007 0 -0.054 -0.008 0.014 -0.094 -0.128 0.114 0 -0.119 0.063 0 -0.094 0.011 0.035 0 -0.042 -0.426 0.013

18 1 0 -0.022 -0.129 0.022 0 0.158 -0.007 0 -0.054 -0.124 0.014 -0.044 -0.128 0 0 -0.189 0.046 0 -0.094 0.011 0.035 0 -0.042 -0.426 0.013

18 1 0 -0.022 -0.129 -0.187 0 0.158 -0.007 0 -0.054 -0.124 0.014 -0.044 -0.128 0 0 -0.119 0.046 0 -0.094 0.011 0.035 0 -0.042 -0.426 0.013

18 1 0 -0.022 -0.129 0.022 0 0.158 -0.007 0 -0.054 -0.008 0.014 -0.094 -0.128 0 0 -0.119 0.063 0 0.026 0.011 0.035 0 -0.042 -0.426 0.013

18 1 0 -0.022 -0.129 0.022 0 0.158 -0.007 0 -0.099 -0.008 -0.096 -0.094 -0.128 0 0 -0.119 0.063 0 -0.094 0.011 0.035 0 -0.042 -0.426 0.013

18 1 0 -0.022 -0.129 0.022 0 0.158 -0.007 0 -0.129 -0.185 -0.054 -0.094 -0.128 0 0 -0.119 0.063 0 -0.094 0.011 0.035 0 -0.042 -0.426 0.013

18 1 0 -0.022 -0.129 0.022 0 0.158 -0.007 0 -0.129 -0.008 -0.096 -0.094 -0.128 0 0 -0.119 0.063 -0.067 -0.094 0.011 0.035 0.067 -0.042 -0.426 0.013

18 1 0 -0.022 -0.129 0.022 0 0.158 -0.027 0 -0.129 -0.008 0.034 -0.094 -0.128 0 0 -0.119 0.063 0 -0.094 0.134 0.035 0 -0.042 -0.426 0.013

18 1 0 -0.022 -0.129 0.022 0 0.158 -0.007 0 -0.129 -0.008 -0.104 -0.094 -0.128 0 0 -0.119 0.063 -0.067 -0.094 0.011 0.035 0 -0.042 -0.426 0.013

18 1 0 -0.022 0.008 0.022 0 0.158 -0.007 0 -0.129 -0.008 -0.096 -0.094 -0.128 0 0 -0.119 0.063 -0.067 -0.094 0.011 0.035 0 -0.042 -0.426 0.013

18 1 0 -0.215 -0.129 0.022 0 0.158 -0.007 0 -0.054 -0.008 0.014 -0.094 -0.128 0 0 -0.119 0.063 0 -0.094 0.011 0.035 0 -0.042 -0.338 0.013

18 1 0 -0.022 -0.129 0.022 0 0.158 -0.007 0 -0.054 -0.008 0.014 -0.094 -0.128 0.004 0 -0.119 0.066 0 -0.094 0.011 0.035 0 -0.042 -0.338 0.013

18 1 0 -0.022 -0.129 0.022 0 0.158 -0.007 0 -0.092 -0.008 -0.054 -0.094 -0.128 0 0 -0.119 0.063 0 -0.094 0.011 0.035 0 -0.042 -0.426 0.013

18 1 0 -0.022 -0.129 0.022 0 0.158 -0.007 0 -0.054 0.001 0.014 -0.044 -0.128 0 0 -0.119 0.046 0 -0.094 0.011 0.035 0 -0.042 -0.426 0.013

18 1 0 -0.022 -0.129 0.022 0 0.158 -0.007 0 -0.129 -0.008 -0.054 -0.094 -0.128 0 -0.106 -0.119 0.063 0 -0.094 0.011 0.035 0 0.019 -0.426 0.013

18 1 0 -0.022 -0.129 0.022 0 0.158 -0.007 0 -0.054 -0.008 0.014 -0.094 -0.128 0 0 -0.119 0.063 -0.016 -0.094 0.011 0.035 0 -0.042 -0.426 0.013

18 1 0 -0.022 -0.129 0.022 0 0.158 0.005 0 -0.129 -0.008 0.034 -0.094 -0.128 0 0 -0.119 0.063 0 -0.094 0.011 0.035 0 -0.042 -0.426 0.013

18 1 0 -0.022 -0.129 0.022 0 0.158 -0.007 0.044 -0.054 -0.008 0.014 -0.094 -0.128 0 0 -0.119 0.063 0 -0.121 0.011 0.035 0 -0.042 -0.338 0.013

18 1 0 -0.022 -0.129 0.022 0 0.158 -0.007 0 -0.129 -0.008 0.034 -0.094 -0.128 0 0 -0.14 0.063 0 -0.094 0.011 0.035 0 -0.042 -0.426 0.013

18 1 0 -0.022 -0.129 0.022 0 0.158 0.065 0 -0.054 -0.008 0.014 -0.094 -0.128 0 0 -0.119 0.063 0 -0.094 0.011 0.035 0 -0.042 -0.426 0.013

18 1 -0.02 -0.022 -0.129 0.022 0 0.158 -0.027 0 -0.129 -0.008 0.034 -0.094 -0.128 0 0 -0.119 0.063 0 -0.094 0.011 0.035 0 -0.042 -0.426 0.013

18 1 0 -0.022 -0.129 0.022 0 0.158 -0.007 0.018 -0.054 -0.008 0.014 -0.094 -0.128 0 0 -0.119 0.063 0.045 -0.094 0.011 0.035 0 -0.042 -0.426 0.013

18 1 0 -0.022 -0.129 0.022 0 0.158 -0.027 0 -0.129 -0.008 0.034 -0.094 -0.128 0 0 -0.228 0.063 0 -0.094 0.011 0.035 0 -0.042 -0.426 0.013

18 1 0 -0.022 -0.129 0.022 0 0.158 -0.193 0 -0.054 -0.124 0.014 0.036 -0.128 0 0 -0.119 0.046 0 -0.094 0.011 0.035 0 -0.042 -0.426 0.013

18 1 0 -0.022 -0.129 0.022 0 0.158 -0.007 0 -0.129 -0.008 -0.096 -0.094 -0.128 0 0 -0.119 0.063 -0.067 -0.094 0.081 0.035 0 -0.042 -0.426 0.013

18 1 0 -0.022 -0.129 0.022 0 0.158 0.002 0 -0.054 -0.008 0.014 -0.094 -0.128 0 0 -0.119 0.066 0 -0.094 0.011 0.035 0 -0.042 -0.338 0.013

18 1 0 -0.173 -0.129 0.022 0 0.158 -0.007 0 -0.129 -0.008 -0.054 -0.094 -0.128 0 0 -0.119 0.063 0 -0.094 0.011 0.035 0 -0.042 -0.426 0.013

18 1 0 -0.022 -0.156 0.022 0 0.158 -0.007 0 -0.054 0.106 0.014 -0.094 -0.073 0 0.207 -0.119 0.175 0 -0.094 0.011 0.035 0 -0.042 -0.426 0.013

18 1 0 -0.022 -0.129 0.022 0 0.158 -0.007 0 -0.054 -0.124 -0.154 -0.044 -0.128 0 0 -0.119 0.046 0 -0.094 0.011 0.035 0 -0.042 -0.426 0.013

18 1 0 -0.022 -0.129 0.022 0 0.158 -0.007 0 -0.054 -0.008 0.014 -0.302 -0.128 0.088 0 -0.119 0.063 0 -0.094 0.011 0.035 0 -0.042 -0.426 0.013

18 1 0 -0.022 -0.185 0.022 0 0.158 -0.007 0 -0.054 0.106 0.014 -0.094 -0.073 0 0 -0.119 0.175 0 -0.094 0.011 0.035 0 -0.042 -0.426 0.013

18 1 0 -0.022 -0.156 0.022 0 0.158 -0.007 0 -0.054 0.106 0.014 -0.094 -0.073 0 0.035 -0.119 0.175 0 -0.094 0.011 0.035 0 -0.042 -0.426 0.013

18 1 0 -0.022 -0.129 0.022 0 0.158 -0.007 0 -0.129 -0.008 -0.054 -0.078 -0.128 0 0 -0.119 0.063 0 -0.094 0.011 0.035 0 -0.042 -0.426 0.013

18 1 0 -0.022 -0.129 0.022 0 0.158 -0.007 0 -0.054 -0.008 0.014 -0.094 -0.172 0 0 -0.119 0.066 0 -0.094 0.011 0.035 0 -0.042 -0.338 0.013

18 1 0 -0.022 -0.129 0.022 0 0.158 -0.007 0 -0.054 -0.008 0.014 -0.094 -0.128 0 0 -0.119 0.063 -0.008 -0.094 0.011 0.035 0 -0.042 -0.426 0.013

18 1 0 -0.022 -0.129 0.022 0 0.158 -0.007 0 -0.043 -0.008 -0.054 -0.094 -0.128 0 0 -0.119 0.063 0 -0.094 0.011 0.035 0 -0.042 -0.426 0.013

18 1 0 -0.022 -0.129 0.022 0 0.158 -0.007 0 -0.129 -0.008 0.034 -0.094 -0.128 -0.134 0 -0.119 0.063 0 -0.094 0.011 0.035 0 -0.042 -0.426 0.013

18 1 0 -0.022 -0.156 0.022 0 0.208 -0.007 0 -0.054 0.106 0.014 -0.094 -0.073 0 0 -0.119 0.175 0 -0.094 0.011 0.035 0 -0.042 -0.426 0.013

18 1 0 -0.022 -0.129 0.022 0 0.158 -0.007 0 -0.054 -0.008 0.014 -0.094 -0.128 0 0 -0.119 0.063 0 -0.094 0.013 0.035 0 -0.042 -0.338 0.013

19 655 0.028 -0.045 -0.019 -0.004 0 0 -0.05 -0.096 0 0.047 0 -0.047 -0.004 0.02 0.131 0.173 -0.041 0 0 -0.011 0.141 -0.066 -0.491 0 0

19 377 0.028 -0.045 -0.019 -0.039 0 0 -0.05 -0.106 -0.009 0.047 0 -0.047 -0.004 0.02 0.097 0.032 -0.041 0.101 0 -0.011 0.141 -0.028 -0.491 0 0

19 275 0.028 -0.045 -0.019 -0.039 0 0 -0.05 -0.106 0 0.047 0.072 -0.047 -0.004 0.02 0.097 0.032 -0.041 0.101 0 -0.011 0.141 -0.028 -0.491 0 0

19 228 0.028 -0.045 -0.019 -0.039 0 0 -0.05 -0.106 0 0.047 0 -0.047 -0.004 0.02 0.097 0.032 -0.041 0.101 0 -0.011 0.141 -0.028 -0.491 0 0

19 392 0.028 -0.045 -0.019 -0.004 0 0 -0.05 -0.096 0 0.047 0 -0.047 -0.004 0.02 0.131 0.045 -0.041 0 0 -0.011 0.141 -0.066 -0.491 0 0

19 211 0.028 -0.045 -0.019 -0.004 0 0 -0.05 -0.096 0 0.047 0 -0.047 -0.004 0.02 0.131 0.173 -0.041 0 0 -0.017 0.141 -0.066 -0.491 0 0

19 118 0.028 -0.054 -0.019 -0.004 0.048 -0.024 -0.05 -0.096 0 0.047 0 -0.047 0.066 0.02 0.131 0.032 0.069 0 0 -0.011 0.141 -0.066 -0.491 0 0

19 108 0.028 -0.045 -0.019 -0.039 0 0 -0.05 -0.106 0 0.047 0 -0.047 -0.004 0.02 0.097 0.032 -0.041 0.101 0 -0.011 0.141 -0.09 -0.491 0 0

19 98 0.028 -0.054 -0.019 -0.004 0.048 0 -0.05 -0.096 0 0.047 0 -0.047 0.066 0.02 0.131 0.032 0.069 0.066 0 -0.011 0.141 -0.066 -0.491 -0.007 0

19 96 0.028 -0.045 -0.019 -0.004 0 0 -0.05 -0.096 0 0.047 0 -0.047 -0.164 0.02 0.131 0.173 -0.041 0 0 -0.011 0.141 -0.066 -0.491 0 0

19 76 0.028 -0.045 -0.019 -0.039 0 0 -0.05 -0.106 -0.009 0.047 -0.073 -0.047 -0.004 0.02 0.097 0.032 -0.041 0.101 0 -0.011 0.141 -0.028 -0.491 0 0

19 19 0.012 -0.045 -0.019 -0.004 0 0 -0.05 -0.096 0 0.047 0 -0.047 -0.004 0.02 0.131 0.045 -0.041 0 0 -0.011 0.141 -0.066 -0.491 0 0

19 17 0.028 -0.045 -0.028 -0.004 0 0 -0.05 -0.096 0 0.047 0 -0.047 -0.004 0.02 0.131 0.173 -0.041 0 0 -0.011 0.141 -0.066 -0.491 0 0

19 14 0.028 -0.045 -0.028 0.175 0 0 -0.05 -0.096 0 0.047 0 -0.047 -0.004 0.02 0.131 0.173 -0.041 0 0 -0.011 0.141 -0.066 -0.491 0 0

19 13 -0.026 -0.045 -0.019 -0.004 0 0.002 -0.05 -0.096 0 0.047 0.06 -0.047 -0.004 0.02 0.131 0.045 -0.041 0 0 -0.011 0.141 -0.078 -0.491 0 0

19 12 -0.026 -0.045 -0.019 -0.004 0 0.002 -0.05 -0.096 0 0.047 0.06 -0.047 -0.004 0.02 0.131 0.045 -0.041 -0.051 0 -0.011 0.141 -0.078 -0.491 0 0

19 10 0.028 -0.045 -0.019 -0.039 0 0 -0.05 -0.106 0 0.047 0 -0.047 -0.004 0.02 0.097 0.032 -0.041 0.101 0 -0.093 0.141 -0.028 -0.491 0 0

19 8 0.028 -0.045 -0.019 -0.004 0 0 -0.05 -0.096 0 -0.005 0 -0.047 -0.164 0.02 0.131 0.173 -0.041 0 0 -0.011 0.141 -0.066 -0.491 0 0

19 8 0.028 -0.045 -0.019 -0.004 0 0 -0.05 -0.096 -0.079 0.047 0 -0.047 -0.004 0.02 0.131 0.045 -0.041 0 0 -0.011 0.141 -0.066 -0.491 0 0

19 8 0.028 -0.045 -0.019 -0.039 0 -0.09 -0.05 -0.106 -0.009 0.047 0 -0.047 -0.004 0.02 0.097 0.032 -0.041 0.101 0 -0.011 0.141 -0.028 -0.491 0 0

19 7 0.028 -0.054 -0.019 -0.004 0.048 0 -0.05 -0.096 0 0.047 0 -0.047 0.066 0.02 0.131 0.032 0.069 0 0 -0.011 0.141 -0.066 -0.491 0 0

19 7 0.028 -0.045 -0.106 -0.039 0 0 -0.05 -0.106 0 0.047 0.072 -0.047 -0.004 0.02 0.097 0.032 -0.041 0.101 0 -0.011 0.141 -0.028 -0.491 0 0

19 6 0.028 -0.045 -0.019 -0.004 0 0 -0.05 -0.096 0 0.047 -0.124 -0.047 -0.004 0.02 0.131 0.173 -0.041 0 0 -0.011 0.141 -0.066 -0.491 0 0

19 5 0.028 -0.045 -0.096 -0.004 0 0 -0.05 -0.096 0 0.047 0 -0.047 -0.164 0.02 0.131 0.173 -0.041 0 0 -0.011 0.141 -0.066 -0.491 0 0

19 5 0.028 -0.045 -0.019 -0.004 0 0 -0.05 -0.096 0 0.047 0 -0.047 -0.004 0.02 0.239 0.173 -0.041 0 0 -0.011 0.141 -0.066 -0.491 0 0

19 4 0.028 -0.045 -0.019 -0.039 0 0 -0.05 -0.106 0 0.047 0 -0.047 -0.147 0.02 0.097 0.032 -0.041 0.101 0 -0.011 0.141 -0.028 -0.504 0 0

19 4 0.028 -0.045 -0.019 -0.039 0 0 -0.05 -0.106 -0.009 0.047 0 -0.047 -0.155 0.02 0.097 0.032 -0.041 0.101 0 -0.011 0.141 -0.028 -0.491 0 0

19 4 0.028 -0.045 -0.019 -0.004 0 0 -0.05 -0.096 0 0.047 0 -0.047 -0.004 0.02 0.134 0.045 -0.041 0 0 -0.011 0.141 -0.066 -0.491 0 0

19 4 0.028 -0.045 -0.019 -0.004 0 0 -0.05 -0.096 0 0.047 0 -0.047 -0.004 0.02 0.131 0.173 -0.041 0 0 -0.011 0.141 -0.066 -0.347 0 0

19 6 0.028 -0.045 -0.019 -0.004 0 0 -0.05 -0.096 0 0.047 0.06 -0.047 -0.004 0.02 0.131 0.045 -0.041 0 0 -0.011 0.141 -0.078 -0.491 0 0

19 4 0.028 -0.045 -0.019 -0.06 0 0 -0.05 -0.096 0 0.047 0 -0.047 -0.164 0.02 0.131 0.173 -0.041 0 0 -0.011 0.141 -0.066 -0.491 0 0

19 4 0.028 -0.045 -0.019 -0.004 0 0 -0.05 -0.096 0 0.047 0 -0.047 -0.009 0.02 0.131 0.045 -0.041 0 0 -0.011 0.141 -0.066 -0.491 0 0

19 4 0.028 -0.045 -0.019 -0.039 0 0 -0.05 -0.106 0 0.047 0.072 -0.047 -0.004 0.02 0.097 -0.089 -0.041 0.101 0 -0.011 0.141 -0.028 -0.491 0 0

19 4 0.028 -0.054 -0.019 -0.004 0.048 -0.024 -0.05 -0.096 0 0.047 0 -0.047 0.066 0.02 0.131 0.032 0.069 0 0 -0.011 0.207 -0.066 -0.491 0 0

19 4 0.028 -0.045 -0.019 -0.004 -0.094 0 -0.05 -0.096 0 0.047 0 -0.047 -0.004 0.02 0.131 0.173 -0.041 0 0 -0.011 0.141 -0.066 -0.491 0 0

19 4 0.028 -0.045 -0.019 -0.004 0 0 -0.05 -0.096 0 0.047 0 -0.047 -0.004 0.02 0.131 0.285 -0.041 0 0 -0.011 0.141 -0.066 -0.491 0 0

19 4 0.028 -0.045 -0.019 -0.004 0 0 -0.05 -0.096 0 0.047 0 -0.047 -0.004 0.02 0.131 0.045 -0.041 0 -0.003 -0.011 0.141 -0.066 -0.491 0 0

19 4 0.028 -0.054 -0.019 -0.004 0.048 0 -0.05 -0.096 0 0.047 0 -0.047 0.114 0.02 0.131 0.032 0.069 0.066 0 -0.011 0.141 -0.066 -0.491 -0.007 0

19 4 0.028 -0.045 -0.019 -0.004 0 0 -0.05 -0.096 0 0.047 0 -0.047 -0.004 0.02 0.131 0.173 -0.041 0 -0.125 -0.011 0.141 -0.066 -0.491 0 0

19 4 0.028 -0.045 -0.019 -0.039 0 0 -0.05 -0.106 0 0.081 0 -0.047 -0.004 0.02 0.097 0.032 -0.041 0.101 0 -0.011 0.141 -0.028 -0.491 0 0

19 4 0.028 -0.045 -0.028 0.175 0 0 -0.05 -0.096 0 0.047 0 -0.047 -0.004 0.02 0.131 0.173 -0.063 0 0 -0.011 0.141 -0.066 -0.491 0 0

19 3 0.028 -0.045 -0.019 -0.004 0 0 -0.05 -0.096 0 0.047 0 -0.05 -0.164 0.02 0.131 0.173 -0.041 0 0 -0.011 0.141 -0.066 -0.491 0 0

19 3 0.028 -0.045 -0.019 -0.039 0 0 -0.05 -0.106 -0.009 0.047 0 -0.047 -0.004 0.02 0.097 0.032 -0.041 0.031 0 -0.011 0.141 -0.028 -0.491 0 0

19 3 0.028 -0.045 -0.019 -0.004 0 0 -0.05 -0.145 0 0.047 0 -0.047 -0.004 0.02 0.131 0.173 -0.041 0 0 -0.011 0.141 -0.066 -0.491 0 0

19 3 0.028 -0.063 -0.019 -0.004 0 0 -0.05 -0.096 0 0.047 0 -0.047 -0.164 0.02 0.131 0.173 -0.041 0 0 -0.011 0.141 -0.066 -0.491 0 0

19 3 0.028 -0.045 -0.019 -0.004 0 0 -0.05 -0.096 0 0.047 0 -0.047 -0.004 0.02 0.131 0.173 -0.041 0.015 0 -0.011 0.141 -0.066 -0.491 0 0

19 3 0.028 -0.054 -0.019 -0.004 0.048 -0.024 -0.05 -0.096 0 0.047 0 -0.047 0.066 0.02 0.131 0.032 0.069 0 0 -0.011 0.141 0.03 -0.491 0 0

19 2 -0.098 -0.045 -0.019 -0.039 0 0 -0.05 -0.106 -0.009 0.047 0 -0.047 -0.004 0.02 0.097 0.032 -0.041 0.101 0 -0.011 0.141 -0.028 -0.491 0 0

19 2 0.028 -0.045 -0.019 -0.004 0 0 0.004 -0.096 0 0.047 0 -0.047 -0.004 0.02 0.131 0.173 -0.041 0 0 -0.011 0.141 -0.066 -0.491 0 0

19 2 0.028 -0.045 -0.019 -0.039 0 0 -0.05 -0.106 -0.009 0.047 0 -0.047 -0.004 0.051 0.097 0.032 -0.041 0.101 0 -0.011 0.141 -0.028 -0.491 0 0

19 2 0.028 -0.045 -0.019 -0.004 0 0 -0.05 -0.096 0 0.047 0 -0.047 -0.004 0.02 0.131 0.173 -0.041 0.012 0 -0.011 0.141 -0.066 -0.491 0 0

19 2 0.028 -0.045 -0.019 -0.004 0 0 -0.05 -0.096 0 0.047 0 -0.047 -0.004 0.02 0.131 0.173 -0.041 0 0 -0.011 0.141 -0.093 -0.491 0 0

19 2 0.028 -0.054 -0.019 -0.004 0.048 -0.024 -0.05 -0.096 0 0.047 0 -0.047 0.066 -0.065 0.131 0.032 0.069 0 0 -0.011 0.141 -0.066 -0.491 0 0

19 2 0.028 -0.045 -0.019 -0.004 0 0 -0.05 -0.096 0 0.047 0 -0.047 -0.004 0.02 0.1 0.045 -0.041 0 0 -0.011 0.141 -0.066 -0.491 0 0

19 2 0.028 -0.045 -0.019 -0.039 0 0 -0.05 -0.106 0 0.047 0 -0.047 -0.004 -0.047 0.097 0.032 -0.041 0.101 0 -0.011 0.141 -0.028 -0.491 0 0

19 4 0.028 -0.054 -0.019 0.063 0.048 0 -0.05 -0.096 0 0.047 0 -0.047 0.066 0.02 0.131 0.032 0.069 0.066 0 -0.011 0.141 -0.066 -0.491 -0.007 0

19 2 0.028 -0.045 -0.019 -0.004 0 0 -0.05 -0.096 0 0.047 0 -0.047 -0.004 0.02 0.131 0.173 -0.084 0 0 -0.011 0.141 -0.066 -0.491 0 0

19 2 0.028 -0.045 -0.019 -0.004 0 0 -0.05 -0.096 0 0.047 0 -0.047 -0.004 0.02 0.131 0.045 -0.041 -0.039 0 -0.011 0.141 -0.066 -0.491 0 0

19 2 0.028 -0.045 -0.019 -0.004 0 -0.029 -0.05 -0.096 0 0.047 0 -0.047 -0.004 0.02 0.131 0.173 -0.041 0 0 -0.011 0.141 -0.066 -0.491 0 0

19 2 0.028 -0.045 -0.019 -0.004 0 0 -0.05 -0.096 0 0.047 0 -0.047 -0.004 0.02 -0.024 0.045 -0.041 0 0 -0.032 0.141 -0.066 -0.491 0 0

19 2 0.028 -0.045 -0.019 -0.039 0 0 -0.05 -0.106 0 0.047 0 -0.047 -0.004 0.02 0.097 0.032 0.09 0.101 0 -0.011 0.141 -0.028 -0.491 0 0

19 2 0.028 -0.045 -0.028 -0.004 0 0 -0.05 -0.096 0 0.047 0 -0.047 -0.004 0.02 0.131 0.173 -0.129 0 0 -0.011 0.141 -0.066 -0.491 0 0

19 2 0.028 -0.054 -0.019 -0.004 0.131 -0.024 -0.05 -0.096 0 0.047 0 -0.047 0.066 0.02 0.131 0.032 0.069 0 0 -0.011 0.141 -0.066 -0.491 0 0

19 2 0.028 -0.045 -0.019 0.053 0 0 -0.05 -0.096 0 0.047 -0.124 -0.047 -0.004 0.02 0.131 0.173 -0.041 0 0 -0.011 0.141 -0.066 -0.491 0 0

19 2 0.028 -0.045 -0.019 -0.004 0 0 -0.05 -0.096 0 0.047 0 -0.047 0.004 0.02 0.131 0.285 -0.041 0 0 -0.011 0.141 -0.066 -0.491 0 0

19 2 0.028 -0.045 -0.019 -0.039 0 0 -0.05 -0.106 -0.009 0.047 0 -0.047 -0.004 0.02 0.097 0.032 -0.041 0.101 0 -0.011 0.223 -0.028 -0.491 0 0

19 2 0.028 -0.045 -0.019 -0.004 0 0 -0.05 -0.096 0 0.047 0 -0.047 -0.004 0.02 0.131 0.173 -0.041 0 0 -0.011 0.141 -0.066 -0.617 0 0

19 2 0.046 -0.045 -0.019 -0.004 0 0 -0.05 -0.096 -0.079 0.047 0 -0.047 -0.004 0.02 0.131 0.045 -0.041 0 0 -0.011 0.141 -0.066 -0.491 0 0

19 2 0.028 -0.045 -0.019 -0.039 0 0 0.019 -0.106 0 0.047 0.072 -0.047 -0.004 0.02 0.097 0.032 -0.041 0.101 0 -0.011 0.141 -0.028 -0.491 0 0

19 2 0.028 -0.045 -0.019 -0.004 0 0 -0.05 -0.096 0 0.047 0 -0.047 -0.004 0.02 0.131 0.023 -0.041 0 0 -0.017 0.141 -0.066 -0.491 0 0

19 2 0.028 -0.045 -0.019 -0.004 0 0 -0.05 -0.096 0 0.047 0 -0.047 -0.004 0.02 0.035 0.173 -0.041 0 0 -0.011 0.141 -0.066 -0.491 0 0

19 2 -0.152 -0.045 -0.019 -0.01 0 0 -0.05 -0.096 0 0.047 0 -0.047 -0.004 0.02 0.131 0.045 -0.041 0 0 -0.011 0.141 -0.066 -0.491 0 0

19 1 0.028 -0.045 -0.019 -0.039 0 0 -0.05 -0.106 -0.009 0.047 0 -0.047 -0.004 0.02 0.097 0.032 -0.041 0.101 0 -0.011 0.141 -0.028 -0.491 0 -0.002

19 1 0.028 -0.045 -0.019 -0.004 0 0 -0.05 -0.096 0 0.047 0 -0.047 -0.004 0.02 0.131 0.173 -0.041 0 0.003 -0.011 0.141 -0.066 -0.491 0 0

19 1 0.028 -0.045 -0.019 -0.039 0 0 -0.05 -0.106 0 0.047 0.072 -0.047 -0.004 0.02 0.097 0.032 -0.041 0.094 0 -0.011 0.141 -0.028 -0.491 0 0

19 1 0.028 -0.045 -0.019 -0.039 0 0 -0.05 -0.106 -0.009 0.047 0 -0.047 -0.004 0.02 0.097 0.032 -0.041 0.101 0 -0.011 0.141 -0.028 -0.491 -0.003 0

19 1 0.028 -0.045 0.023 -0.004 0 0 -0.05 -0.096 0 0.047 0 -0.047 -0.004 0.02 0.131 0.173 -0.041 0 0 -0.011 0.141 -0.066 -0.491 0 0

19 1 0.028 -0.045 -0.019 -0.004 0 0 -0.05 -0.096 0 0.047 0 -0.047 -0.164 0.02 0.131 0.173 -0.041 0 0 -0.011 0.141 -0.066 -0.491 0 -0.076

19 1 0.028 -0.045 -0.019 -0.004 0 0 -0.05 -0.096 0 0.047 0 -0.047 -0.004 0.02 0.131 0.173 -0.041 0 0 -0.011 0.141 -0.066 -0.491 0 0.035

19 1 0.028 -0.045 -0.019 -0.004 0 0 -0.05 -0.096 0 0.047 0.056 -0.047 -0.004 0.02 0.131 0.045 -0.041 0 0 -0.011 0.141 -0.078 -0.491 0 0

19 1 0.028 -0.045 -0.019 -0.215 0 0 -0.05 -0.096 0 0.047 0 -0.047 -0.004 0.02 0.131 0.173 -0.041 0 0 -0.011 0.141 -0.066 -0.491 0 0

19 1 0.028 -0.045 -0.019 -0.039 0 0 -0.05 -0.106 0 0.047 0.072 -0.047 -0.004 0.02 0.097 0.032 -0.041 0.101 0 0.093 0.141 -0.028 -0.491 0 0

19 1 0.028 -0.045 -0.019 -0.039 0 0 -0.05 -0.106 -0.009 0.047 0 -0.047 -0.004 0.02 0.097 0.08 -0.041 0.101 0 -0.011 0.141 -0.028 -0.491 0 0

19 1 0.028 -0.045 -0.019 -0.039 0 0 -0.05 -0.106 0.087 0.047 0 -0.047 -0.004 0.02 0.097 0.032 -0.041 0.101 0 -0.011 0.141 -0.028 -0.491 0 0

19 1 0.028 -0.045 -0.019 -0.004 0 0 -0.05 -0.096 0 -0.047 0 -0.047 -0.004 0.02 0.131 0.173 -0.041 0 0 -0.011 0.141 -0.066 -0.491 0 0

19 1 0.028 -0.045 -0.019 -0.039 0 0 -0.05 -0.106 0 0.047 0 -0.047 -0.004 0.02 0.097 0.032 -0.041 0.101 0 -0.011 0.141 -0.04 -0.491 0 0

19 1 0.028 -0.045 -0.019 -0.004 0 0 -0.05 -0.096 0 0.047 0 -0.047 -0.004 0.02 0.131 0.173 -0.041 0 -0.021 -0.017 0.141 -0.066 -0.491 0 0

19 1 0.028 -0.045 0.024 -0.004 0 0 -0.05 -0.096 0 0.047 0 -0.047 -0.004 0.02 0.131 0.173 -0.041 0 0 -0.017 0.141 -0.066 -0.491 0 0

19 1 0.028 -0.045 -0.019 -0.039 0 0 -0.05 -0.106 0 0.047 0.077 -0.047 -0.004 0.02 0.097 0.032 -0.041 0.101 0 -0.011 0.141 -0.028 -0.491 0 0

19 1 0.028 -0.045 -0.019 -0.039 0 0 -0.05 -0.106 0.035 0.047 0 -0.047 -0.004 0.02 0.097 0.032 -0.041 0.101 0 -0.011 0.141 -0.028 -0.491 0 0

19 1 0.028 -0.045 -0.019 -0.004 0 0 -0.05 -0.096 0 0.047 0 -0.047 0.077 0.02 0.131 0.173 -0.041 0 0 -0.017 0.141 -0.066 -0.491 0 0

19 1 0.028 -0.045 -0.019 -0.039 0 0 -0.05 -0.106 0 0.047 0.072 -0.047 -0.004 0.02 0.097 0.009 -0.041 0.101 0 -0.011 0.141 -0.028 -0.491 0 0

19 1 0.028 -0.045 -0.019 -0.004 0 0 -0.05 -0.085 0 0.047 0 -0.047 -0.004 0.02 0.131 0.173 -0.041 0 0 -0.011 0.141 -0.066 -0.491 0 0

19 1 0.028 -0.045 -0.019 -0.039 0 0 -0.05 -0.091 -0.009 0.047 -0.073 -0.047 -0.004 0.02 0.097 0.032 -0.041 0.101 0 -0.011 0.141 -0.028 -0.491 0 0

19 1 0.028 -0.054 -0.019 -0.004 0.048 -0.024 -0.05 -0.096 0.092 0.047 0 -0.047 0.066 0.02 0.131 0.032 0.069 0 0 -0.011 0.141 -0.066 -0.491 0 0

19 1 0.028 -0.045 -0.019 -0.004 0 0 -0.05 -0.096 0 0.047 0 -0.047 -0.004 0.02 0.131 0.173 -0.041 0 0 -0.011 0.141 -0.062 -0.491 0 0

19 1 0.028 -0.045 -0.019 -0.039 0 0 -0.05 -0.106 0 0.047 0.072 -0.047 -0.004 0.138 0.097 0.032 -0.041 0.101 0 -0.011 0.141 -0.028 -0.491 0 0

19 1 0.028 -0.045 -0.019 -0.039 0 0 -0.05 -0.106 -0.009 0.047 0 -0.047 -0.004 0.02 0.097 0.032 -0.041 0.101 0 -0.011 0.244 -0.028 -0.491 0 0

19 1 0.028 -0.045 -0.019 -0.004 0 0 -0.05 -0.096 0 0.047 0 -0.047 -0.004 0.02 0.158 0.173 -0.041 0 0 -0.011 0.141 -0.066 -0.491 0 0

19 1 0.028 0 -0.019 -0.004 0 0 -0.05 -0.096 0 0.047 0 -0.047 -0.004 0.02 0.131 0.173 -0.041 0 0 -0.011 0.141 -0.066 -0.491 0 0

19 1 0.028 -0.045 -0.019 -0.004 0 0 -0.05 -0.096 0 0.047 0 -0.047 -0.004 0.02 0.131 0.045 -0.041 -0.02 0 -0.011 0.141 -0.066 -0.491 0 0

19 1 0.028 -0.045 -0.019 -0.039 0 0 -0.05 -0.106 0 0.047 0.072 -0.047 -0.004 0.02 0.097 0.032 -0.041 0.164 0 -0.011 0.141 -0.028 -0.491 0 0

19 1 0.028 -0.045 -0.019 -0.039 0 0 -0.05 -0.106 0 0.047 0.072 -0.047 -0.004 0.02 0.097 0.032 -0.041 0.101 0 -0.011 0.141 -0.028 -0.491 0 0.116

19 1 0.028 -0.045 -0.019 -0.039 0 0 -0.05 -0.096 0 0.047 0 -0.047 -0.004 0.02 0.097 0.032 -0.041 0.101 0 -0.011 0.141 -0.028 -0.491 0 0

19 1 -0.299 -0.045 -0.019 -0.004 0 0 -0.05 -0.096 0 0.047 0 -0.047 -0.004 0.02 0.131 0.045 -0.041 0 0 -0.011 0.141 -0.066 -0.491 0 0

19 1 0.028 -0.045 -0.019 -0.039 0 0 -0.05 -0.106 -0.009 0.047 0 -0.047 -0.004 0.016 0.097 0.032 -0.041 0.101 0 -0.011 0.141 -0.028 -0.491 0 0

19 1 0.028 -0.045 -0.019 -0.039 0 0 -0.05 -0.106 -0.009 0.047 0 -0.011 -0.004 0.02 0.097 0.032 -0.041 0.101 0 -0.011 0.141 -0.028 -0.491 0 0

19 1 0.028 -0.045 -0.019 -0.039 0 0 -0.05 -0.106 0 0.047 0.072 -0.054 -0.004 0.02 0.097 0.032 -0.041 0.101 0 -0.011 0.141 -0.028 -0.491 0 0

19 1 0.028 -0.045 -0.019 -0.004 0 0 -0.05 -0.096 0.085 0.047 0 -0.047 -0.004 0.02 0.131 0.045 -0.041 0 0 -0.011 0.141 -0.066 -0.491 0 0

19 1 0.028 -0.045 -0.019 -0.004 0 0 -0.05 -0.096 0 0.047 0 -0.047 -0.004 0.031 0.131 0.173 -0.041 0 0 -0.011 0.141 -0.066 -0.491 0 0

19 1 -0.046 -0.045 -0.019 -0.039 0 0 -0.05 -0.106 0 0.047 0 -0.047 -0.004 0.02 0.097 0.032 -0.041 0.101 0 -0.011 0.141 -0.09 -0.491 0 0

19 1 0.028 -0.045 0.073 -0.039 0 0 -0.05 -0.106 0 0.047 0.072 -0.047 -0.004 0.02 0.097 0.032 -0.041 0.101 0 -0.011 0.141 -0.028 -0.491 0 0

19 1 0.028 -0.045 -0.019 -0.039 0 0 -0.05 -0.106 0 0.047 0 -0.047 -0.004 -0.058 0.097 0.032 -0.041 0.101 0 -0.011 0.141 -0.028 -0.491 0 0

19 1 0.028 -0.045 -0.019 -0.004 0 0 -0.05 -0.096 0 0.047 0 -0.047 -0.004 0.02 0.131 0.173 -0.041 0 0 -0.011 0.141 -0.066 -0.491 0 0.08

19 1 0.028 -0.045 -0.019 -0.004 0 0 -0.05 -0.096 0 0.047 0.123 -0.047 -0.004 0.02 0.131 0.173 -0.041 0.015 0 -0.011 0.141 -0.066 -0.491 0 0

19 1 0.028 -0.045 -0.019 -0.039 0 0 -0.05 -0.106 -0.009 0.047 0 -0.047 -0.004 0.02 0.093 0.032 -0.041 0.101 0 -0.011 0.141 -0.028 -0.491 0 0

19 1 0.028 -0.045 -0.019 -0.039 0 0 -0.05 -0.106 0 0.047 0.072 -0.047 -0.004 0.02 0.195 0.032 -0.041 0.101 0 -0.011 0.141 -0.028 -0.491 0 0

19 1 0.028 -0.045 -0.019 -0.039 0 0 -0.05 -0.106 0 0.047 0 -0.047 -0.004 0.02 0.097 0.032 -0.041 0.101 0.066 -0.011 0.141 -0.028 -0.491 0 0

19 1 0.028 -0.045 -0.019 -0.004 0 0 -0.05 -0.096 0 0.047 0 -0.047 -0.004 0.02 0.131 0.157 -0.041 0 0 -0.011 0.141 -0.066 -0.491 0 0

19 1 0.028 -0.036 -0.019 -0.039 0 0 -0.05 -0.106 -0.009 0.047 0 -0.047 -0.004 0.02 0.097 0.032 -0.041 0.101 0 -0.011 0.141 -0.028 -0.491 0 0

19 1 0.028 -0.054 -0.019 -0.004 0.048 0 -0.05 -0.096 0 0.047 0 -0.047 0.066 0.02 0.131 0.032 0.069 0.119 0 -0.011 0.141 -0.066 -0.491 -0.007 0

19 1 0.028 -0.045 -0.019 -0.004 0 0 -0.05 -0.096 0 0.047 0 -0.047 -0.004 0.02 0.131 0.173 -0.041 0.041 0 -0.011 0.141 -0.066 -0.491 0 0

19 1 0.028 -0.045 -0.019 -0.004 0 0 -0.05 -0.096 0 0.047 0 -0.047 -0.004 0.02 0.131 0.045 -0.041 0 0 -0.011 0.141 -0.004 -0.491 0 0

19 1 0.028 -0.045 -0.019 -0.039 0 0 -0.05 -0.106 -0.009 0.047 0 -0.047 -0.004 0.175 0.097 0.032 -0.041 0.101 0 -0.011 0.141 -0.028 -0.491 0 0

19 1 0.028 -0.045 -0.019 -0.004 0 0 -0.05 -0.168 0 0.047 0 -0.047 -0.004 0.02 0.131 0.173 -0.041 0 0 -0.011 0.141 -0.066 -0.491 0 0

19 1 0.028 -0.045 -0.019 -0.039 0 0 -0.05 -0.106 0 0.047 0 -0.047 -0.004 0.02 0.097 0.032 -0.041 0.101 0 -0.011 0.192 -0.028 -0.491 0 0

19 1 0.028 -0.045 -0.019 -0.039 0.148 0 -0.05 -0.106 0 0.047 0.072 -0.047 -0.004 0.02 0.097 0.032 -0.041 0.101 0 -0.011 0.141 -0.028 -0.491 0 0

19 1 0.028 -0.054 -0.019 -0.004 0.048 -0.024 -0.05 -0.096 0 0.047 0 -0.047 0.066 0.132 0.131 0.032 0.069 0 0 -0.011 0.141 -0.066 -0.491 0 0

19 1 0.028 -0.045 -0.019 -0.039 0 0 -0.05 -0.106 -0.117 0.047 0 -0.047 -0.004 0.02 0.097 0.032 -0.041 0.101 0 -0.011 0.141 -0.028 -0.491 0 0

19 1 0.028 -0.045 -0.019 -0.039 0 0 -0.05 -0.106 0 0.047 0 -0.047 -0.004 0.02 0.097 0.032 -0.041 0.101 -0.068 -0.011 0.141 -0.028 -0.491 0 0

19 1 0.028 -0.045 -0.019 -0.039 0 0 -0.05 -0.106 0 0.047 -0.075 -0.047 -0.004 0.02 0.097 0.032 -0.041 0.101 0 -0.011 0.141 -0.09 -0.491 0 0

19 1 0.028 -0.045 -0.019 -0.039 0 0 -0.05 -0.106 0 0.047 0.09 -0.047 -0.004 0.02 0.097 0.032 -0.041 0.101 0 -0.011 0.141 -0.028 -0.491 0 0

19 1 0.028 -0.054 -0.019 -0.004 0.048 -0.024 -0.05 -0.096 0 0.047 0 -0.047 0.066 0.02 0.131 0.032 0.069 0 0 -0.011 0.221 -0.066 -0.491 0 0

19 1 0.028 -0.045 -0.019 -0.004 0 0 -0.05 -0.096 -0.052 0.047 0 -0.047 -0.004 0.02 0.131 0.173 -0.041 0 0 -0.017 0.141 -0.066 -0.491 0 0

19 1 0.028 -0.054 -0.019 -0.004 0.048 -0.024 -0.05 -0.096 0 0.047 -0.01 -0.047 0.066 0.02 0.131 0.032 0.069 0 0 -0.011 0.141 -0.066 -0.491 0 0

19 1 0.028 -0.054 -0.019 -0.004 0.048 -0.024 -0.05 -0.096 0 0.047 0 -0.047 0.066 0.02 0.131 0.032 0.069 0 0 -0.011 0.141 0.03 -0.491 0 -0.133

20 1562 -0.091 0.147 0.039 -0.036 0 0.049 0 -0.096 -0.184 0 0 -0.199 0.174 -0.045 -0.139 -0.272 0.172 0.016 0.321 -0.113 0 -0.19 -0.096 -0.247 0.107

20 527 -0.054 0.147 0.039 -0.036 0 0.049 0 -0.096 -0.184 0 0 -0.199 0.174 -0.045 -0.139 -0.272 0.172 0.016 0.321 -0.113 0 -0.19 -0.043 -0.247 0.107

20 321 -0.091 0.069 0.039 -0.036 0 0.039 0 0.257 -0.184 0.156 0 0 0.174 -0.045 -0.139 -0.272 0.172 -0.005 0.226 -0.113 0 -0.19 -0.096 -0.283 0.107

20 284 -0.091 0.147 0.039 -0.036 0 0.049 0 -0.096 -0.184 0 0 -0.246 0.174 -0.045 -0.139 -0.272 0.172 0.016 0.321 -0.113 0 -0.19 -0.096 -0.247 0.107

20 238 -0.091 0.147 0.039 -0.036 0 0.049 0 -0.096 -0.184 0 0 -0.199 0.174 -0.045 -0.139 -0.358 0.172 0.016 0.321 -0.113 0 -0.19 -0.096 -0.256 0.107

20 195 -0.091 0.147 0.039 -0.036 0 0.049 0 -0.096 -0.184 0 0 -0.199 0.174 -0.045 -0.139 -0.272 0.172 0.016 0.366 -0.113 0 -0.19 -0.013 -0.247 0.107

20 162 -0.091 0.147 0.039 -0.036 0 0.049 0 -0.096 -0.184 0.169 0 -0.199 0.174 -0.045 -0.139 -0.272 0.172 0.016 0.321 -0.113 -0.018 -0.19 -0.096 -0.247 0.107

20 131 -0.091 0.147 0.039 -0.036 0 0.049 0 -0.096 -0.184 0 0 -0.199 0.174 -0.045 -0.139 -0.272 0.172 0.016 0.321 -0.113 -0.068 -0.19 -0.096 -0.247 0.107

20 118 -0.091 0.147 0.039 -0.036 0 0.049 0 -0.096 -0.184 0.169 0 -0.199 0.174 -0.045 -0.139 -0.272 0.172 0.016 0.321 -0.113 -0.018 -0.228 -0.096 -0.247 0.107

20 85 -0.091 0.147 0.039 -0.036 0 0.049 0 -0.096 -0.184 0 0 -0.199 0.174 -0.045 -0.139 -0.272 0.172 0.061 0.321 -0.113 0 -0.19 -0.096 -0.247 0.107

20 68 -0.104 0.147 0.039 -0.036 0 0.049 0 -0.096 -0.184 0 0 -0.199 0.174 -0.045 -0.139 -0.272 0.172 0.061 0.321 -0.113 0 -0.19 -0.096 -0.247 0.107

20 66 -0.091 0.147 0.039 -0.036 0 0.049 0 -0.096 -0.184 0 0 -0.199 0.174 -0.045 -0.139 -0.272 0.172 0.016 0.366 -0.113 0 -0.19 -0.096 -0.247 0.107

20 23 -0.091 -0.028 0.039 -0.036 0 0.039 0 0.257 -0.184 0 0 0 0.174 -0.045 -0.139 -0.272 0.172 -0.005 0.226 -0.113 0.065 -0.19 -0.096 -0.283 0.107

20 22 -0.054 0.147 0.039 -0.036 0 0.049 0 -0.096 -0.184 0 0 -0.199 0.174 -0.045 -0.139 -0.272 0.172 0.016 0.321 -0.113 0 -0.19 -0.043 -0.247 0.123

20 41 -0.091 0.147 0.039 -0.036 0.017 0.049 0 -0.096 -0.184 0.169 0 -0.199 0.174 -0.045 -0.139 -0.272 0.172 0.016 0.321 -0.113 -0.018 -0.228 -0.096 -0.247 0.107

20 20 -0.091 0.147 0.039 -0.036 0 0.049 0 -0.096 -0.184 0 0 -0.199 0.174 -0.045 -0.139 -0.272 0.172 0.016 0.321 -0.113 0 -0.19 0.125 -0.247 0.107

20 15 -0.091 0.147 0.039 -0.036 0 0.049 0 -0.096 -0.184 0 0 -0.199 0.174 -0.045 -0.139 -0.272 0.172 0.016 0.321 -0.113 0 -0.19 -0.038 -0.247 0.117

20 15 -0.091 0.147 0.039 -0.036 0 0.049 0 -0.096 -0.184 0 0.035 -0.199 0.174 -0.045 -0.139 -0.272 0.172 0.016 0.321 -0.113 0 -0.19 -0.096 -0.247 0.107

20 15 -0.091 0.147 -0.101 -0.036 0 0.049 0 -0.096 -0.184 0 0 -0.199 0.174 -0.045 -0.139 -0.358 0.172 0.016 0.321 -0.113 0 -0.19 -0.096 -0.256 0.107

20 12 -0.091 0.147 0.039 -0.036 0 0.049 0 -0.096 -0.184 0 0 -0.199 0.174 -0.045 -0.139 -0.272 0.172 0.049 0.321 -0.113 0 -0.19 -0.096 -0.247 0.107

20 12 -0.091 0.147 0.039 -0.036 0.174 0.049 0 -0.096 -0.184 0 0 -0.199 0.174 -0.045 -0.139 -0.272 0.172 0.016 0.321 -0.113 0 -0.19 -0.096 -0.247 0.107

20 12 -0.091 0.147 0.039 -0.036 0 0.049 0 -0.096 -0.184 0 0 -0.199 0.174 -0.045 -0.139 -0.272 0.172 0.016 0.321 -0.113 -0.068 -0.19 -0.068 -0.247 0.107

20 10 -0.091 0.147 0.039 -0.036 0 0.049 0 -0.096 -0.184 0 0 -0.199 0.174 -0.045 -0.139 -0.272 0.172 0.016 0.321 -0.113 0 -0.213 -0.096 -0.247 0.107

20 9 -0.091 0.147 0.039 -0.036 0 0.049 0 -0.096 -0.184 0 0 -0.246 0.174 -0.279 -0.139 -0.272 0.172 0.016 0.321 -0.113 0 -0.19 -0.096 -0.247 0.107

20 8 -0.091 0.147 0.039 -0.036 0 0.049 0 -0.096 -0.184 0 -0.091 -0.199 0.174 -0.045 -0.139 -0.272 0.172 0.016 0.321 -0.113 -0.068 -0.19 -0.096 -0.247 0.107

20 7 -0.091 0.147 0.039 -0.036 0 0.049 0 -0.096 -0.184 0 0 -0.199 0.174 -0.045 -0.139 -0.272 0.172 0.016 0.321 -0.113 0 -0.185 -0.096 -0.247 0.107

20 7 -0.104 0.147 0.039 -0.036 0 0.049 0 -0.096 -0.184 0 0.023 -0.199 0.174 -0.045 -0.139 -0.272 0.172 0.061 0.321 -0.113 0 -0.19 -0.096 -0.247 0.107

20 7 -0.091 0.147 -0.06 -0.036 0 0.049 0 -0.096 -0.184 0 0 -0.199 0.174 -0.045 -0.139 -0.272 0.172 0.016 0.321 -0.113 0 -0.19 -0.096 -0.247 0.107

20 7 -0.054 0.147 0.039 -0.036 0 0.049 0 -0.096 -0.184 0 0 -0.199 0.174 -0.045 -0.056 -0.272 0.172 0.016 0.321 -0.113 0 -0.19 -0.043 -0.247 0.107

20 7 -0.091 0.147 0.039 -0.036 0 0.049 0 -0.096 -0.184 0 0 -0.199 0.174 -0.045 -0.139 -0.272 0.172 0.016 0.321 -0.113 -0.146 -0.19 -0.096 -0.247 0.107

20 7 -0.091 0.147 0.039 -0.036 0 0.049 0 -0.187 -0.184 0 0 -0.199 0.174 -0.045 -0.139 -0.272 0.172 0.016 0.321 -0.113 0 -0.19 -0.096 -0.247 0.107

20 6 -0.091 0.147 0.039 -0.036 0 0.049 0 -0.096 -0.184 0 0 -0.199 0.174 -0.045 -0.139 -0.272 0.172 0.016 0.321 -0.172 0 -0.19 -0.096 -0.247 0.107

20 6 -0.091 0.088 0.039 -0.036 0 0.049 0 -0.096 -0.184 0 0 -0.199 0.174 -0.045 -0.139 -0.358 0.172 0.016 0.321 -0.113 0 -0.19 -0.096 -0.256 0.107

20 6 -0.091 0.147 0.039 -0.036 0 0.049 0 -0.096 -0.184 0.169 0 -0.199 0.174 -0.045 -0.139 -0.272 0.298 0.016 0.321 -0.113 -0.018 -0.19 -0.096 -0.247 0.107

20 6 -0.054 0.147 0.039 -0.036 0 -0.02 0 -0.096 -0.184 0 0 -0.199 0.174 -0.045 -0.139 -0.272 0.172 0.016 0.321 -0.113 0 -0.19 -0.043 -0.247 0.107

20 5 -0.091 0.147 0.039 0.003 0 0.049 0 -0.096 -0.184 0 0 -0.199 0.174 -0.045 -0.139 -0.272 0.172 0.016 0.321 -0.113 0 -0.19 -0.096 -0.247 0.107

20 5 -0.104 0.147 0.039 -0.036 0 0.049 0 -0.096 -0.184 0 0 -0.199 0.174 -0.045 -0.139 -0.272 0.172 0.061 0.321 -0.113 0 -0.19 -0.096 -0.247 0.198

20 6 -0.054 0.147 0.039 -0.036 0 0.049 0 -0.096 -0.184 0 0 -0.199 0.174 -0.045 -0.139 -0.272 0.172 0.016 0.321 -0.113 0 -0.19 -0.193 -0.247 0.107

20 4 -0.091 0.147 0.039 -0.036 0 0.049 0 -0.096 -0.184 0 0 -0.199 0.174 -0.045 -0.139 -0.272 0.172 0.016 0.321 -0.113 -0.018 -0.19 -0.096 -0.247 0.107

20 4 -0.091 0.147 0.039 -0.036 -0.076 0.049 0 -0.096 -0.184 0 0 -0.199 0.174 -0.045 -0.139 -0.272 0.172 0.016 0.197 -0.113 0 -0.19 -0.096 -0.247 0.107

20 4 -0.091 0.147 0.039 -0.036 0 0.049 0 -0.096 -0.184 0 0 -0.199 0.174 -0.045 -0.139 -0.272 0.172 0.016 0.321 -0.113 0 -0.063 -0.096 -0.247 0.107

20 4 -0.091 0.147 0.039 -0.036 0 0.049 0 -0.096 -0.184 0 0 -0.246 0.174 -0.22 -0.139 -0.272 0.172 0.016 0.321 -0.113 0 -0.19 -0.096 -0.247 0.107

20 4 -0.131 0.147 0.039 -0.036 0 0.049 0 -0.096 -0.184 0 0 -0.199 0.174 -0.045 -0.139 -0.272 0.172 0.016 0.321 -0.113 -0.068 -0.19 -0.096 -0.247 0.107

20 4 -0.091 0.147 0.039 -0.036 0 0.049 0 -0.096 -0.184 0 0 -0.199 0.174 -0.045 -0.139 -0.272 0.172 0.016 0.436 -0.113 0 -0.19 -0.096 -0.247 0.107

20 4 -0.091 0.163 0.039 -0.036 0 0.049 0 -0.096 -0.184 0 0 -0.199 0.174 -0.045 -0.139 -0.272 0.172 0.016 0.321 -0.113 0 -0.19 -0.096 -0.247 0.107

20 4 -0.091 0.088 0.039 -0.036 0 0.049 0 -0.096 -0.213 0 0 -0.199 0.174 -0.045 -0.139 -0.358 0.172 0.016 0.321 -0.113 0 -0.19 -0.096 -0.256 0.107

20 4 -0.091 0.147 0.189 -0.036 0 0.049 0 -0.096 -0.184 0 0 -0.199 0.174 -0.045 -0.139 -0.272 0.172 0.016 0.321 -0.113 -0.068 -0.19 -0.096 -0.247 0.107

20 4 -0.054 0.053 0.039 -0.036 0 0.049 0 -0.096 -0.184 0 0 -0.199 0.174 -0.045 -0.139 -0.272 0.172 0.016 0.321 -0.113 0 -0.19 -0.043 -0.247 0.107

20 4 -0.091 0.147 0.039 -0.036 0 0.049 0 -0.181 -0.184 0 0 -0.199 0.174 -0.045 -0.139 -0.272 0.172 0.016 0.366 -0.113 0 -0.19 -0.013 -0.247 0.107

20 4 -0.091 0.147 0.039 -0.036 0 0.049 0 -0.096 -0.184 0 0 -0.199 0.174 -0.074 -0.139 -0.358 0.172 0.016 0.321 -0.113 0 -0.19 -0.096 -0.256 0.107

20 4 -0.091 0.147 0.039 -0.036 0 0.049 0 -0.096 -0.184 0 0 -0.246 0.174 -0.045 -0.139 -0.272 0.172 0.081 0.321 -0.113 0 -0.19 -0.096 -0.247 0.107

20 4 -0.091 0.147 0.039 -0.036 0.174 0.049 0 -0.096 -0.184 0 0 -0.199 0.174 -0.045 -0.139 -0.272 0.172 0.016 0.321 -0.113 0 -0.19 -0.096 -0.082 0.107

20 4 -0.091 0.147 0.039 -0.036 0 0.049 0 -0.096 -0.184 -0.033 0 -0.199 0.174 -0.045 -0.139 -0.272 0.172 0.016 0.321 -0.113 0 -0.19 -0.096 -0.247 0.107

20 4 -0.091 0.147 0.039 -0.036 0 0.049 0 -0.096 -0.184 0.169 0 -0.199 0.174 -0.045 -0.142 -0.272 0.172 0.016 0.321 -0.113 -0.018 -0.228 -0.096 -0.247 0.107

20 4 -0.091 0.147 0.039 -0.036 0 0.049 0 -0.096 -0.184 0.169 0 -0.199 0.174 -0.045 -0.139 -0.272 0.172 0.016 0.321 -0.113 -0.018 -0.19 -0.096 -0.247 0.019

20 4 -0.054 0.147 0.039 -0.036 0 0.049 0 -0.096 -0.336 0 0 -0.199 0.174 -0.045 -0.139 -0.272 0.172 0.016 0.321 -0.113 0 -0.19 -0.043 -0.247 0.107

20 4 -0.091 0.147 0.039 -0.036 0 0.049 0 -0.096 -0.184 0 0 -0.199 0.174 -0.045 -0.139 -0.272 0.172 0.016 0.321 -0.113 0.025 -0.19 -0.096 -0.247 0.107

20 3 -0.091 0.147 0.039 -0.036 0 0.049 0 -0.096 -0.11 0 0 -0.199 0.174 -0.045 -0.139 -0.272 0.172 0.016 0.321 -0.113 0 -0.19 -0.096 -0.247 0.107

20 3 -0.104 0.147 0.039 -0.036 0 0.049 0 -0.096 -0.184 0 0 -0.22 0.174 -0.045 -0.139 -0.272 0.172 0.061 0.321 -0.113 0 -0.19 -0.096 -0.247 0.107

20 3 -0.091 0.147 0.039 -0.036 0.2 0.049 0 -0.096 -0.184 0 0 -0.199 0.174 -0.045 -0.139 -0.272 0.172 0.016 0.321 -0.113 0 -0.19 -0.096 -0.247 0.107

20 3 -0.054 0.147 0.039 -0.036 0 0.049 0 -0.096 -0.184 0 0 -0.199 0.016 -0.045 -0.139 -0.272 0.172 0.016 0.321 -0.113 0 -0.19 -0.043 -0.247 0.107

20 2 -0.091 0.049 0.039 -0.036 0 0.049 0 -0.096 -0.184 0 0 -0.199 0.174 -0.045 -0.139 -0.272 0.172 0.061 0.321 -0.113 0 -0.19 -0.096 -0.247 0.107

20 2 -0.091 0.147 0.039 -0.036 -0.274 0.049 0 -0.096 -0.184 0 0 -0.199 0.174 -0.045 -0.139 -0.272 0.172 0.016 0.321 -0.113 0 -0.19 -0.096 -0.247 0.107

20 2 -0.091 0.147 0.039 -0.036 0 0.049 0 -0.096 -0.184 0 0.162 -0.199 0.174 -0.045 -0.139 -0.272 0.172 0.061 0.321 -0.113 0 -0.19 -0.096 -0.247 0.107

20 2 -0.091 0.147 0.039 -0.036 0 0.049 0 -0.096 -0.184 0 0 -0.199 0.222 -0.045 -0.139 -0.272 0.172 0.016 0.366 -0.113 0 -0.19 -0.013 -0.247 0.107

20 2 -0.091 0.147 0.039 -0.036 0 -0.056 0 -0.096 -0.184 0 0 -0.199 0.174 -0.045 -0.139 -0.272 0.172 0.016 0.321 -0.113 0 -0.19 -0.096 -0.247 0.107

20 2 -0.091 0.147 0.039 -0.036 0 0.049 0 -0.096 -0.184 0 0 -0.199 0.174 -0.045 -0.139 -0.272 0.172 0.016 0.341 -0.113 0 -0.19 -0.096 -0.247 0.107

20 2 -0.054 0.147 0.039 -0.036 0 0.049 0 -0.096 -0.184 0 0 -0.199 0.192 -0.045 -0.139 -0.272 0.172 0.016 0.321 -0.113 0 -0.19 -0.043 -0.247 0.107

20 2 -0.091 0.147 0.039 -0.036 0 0.049 0 -0.096 -0.184 0 0 -0.246 0.174 -0.279 -0.139 -0.272 0.172 0.016 0.321 -0.113 0 -0.19 -0.096 -0.165 0.107

20 2 -0.054 0.147 0.039 -0.036 0 0.049 0 -0.096 -0.184 0 -0.038 -0.199 0.174 -0.045 -0.139 -0.272 0.172 0.016 0.321 -0.113 0 -0.19 -0.043 -0.247 0.107

20 2 -0.091 0.147 0.039 -0.036 0 0.049 0 -0.096 -0.184 0 0.035 -0.199 0.174 -0.045 -0.139 -0.272 0.172 0.016 0.321 -0.113 0 -0.19 -0.096 -0.265 0.107

20 2 -0.091 0.147 0.039 -0.036 0 0.049 0 -0.096 -0.184 0 0 -0.199 0.174 -0.045 -0.139 -0.272 0.172 0.016 0.321 -0.113 0 -0.045 -0.096 -0.247 0.107

20 2 -0.091 0.147 0.039 -0.036 0 0.049 0 -0.096 -0.184 0 0 -0.313 0.174 -0.045 -0.139 -0.272 0.172 0.016 0.341 -0.113 0 -0.19 -0.096 -0.247 0.107

20 2 -0.091 0.147 0.039 -0.036 0 0.049 0 -0.096 -0.184 0 -0.022 -0.199 0.174 -0.045 -0.139 -0.272 0.172 0.016 0.321 -0.113 0 -0.19 -0.096 -0.247 0.107

20 2 -0.091 0.147 0.039 -0.036 0 0.049 -0.046 -0.096 -0.184 0.169 0 -0.199 0.174 -0.045 -0.139 -0.272 0.172 0.016 0.321 -0.113 -0.018 -0.19 -0.096 -0.247 0.107

20 2 -0.091 0.147 0.039 -0.036 0 0.049 0 -0.096 -0.184 0 0 -0.199 0.174 -0.045 -0.139 -0.234 0.172 0.016 0.321 -0.113 0 -0.19 -0.096 -0.247 0.107

20 2 -0.091 0.147 0.039 -0.036 0 0.049 0 -0.096 -0.184 0 0 -0.199 0.077 -0.045 -0.139 -0.272 0.172 0.016 0.366 -0.113 0 -0.19 -0.013 -0.247 0.05

20 2 -0.091 0.147 0.039 -0.036 0 0.049 0 -0.096 -0.184 0 0 -0.199 0.174 -0.045 -0.139 -0.272 0.172 0.016 0.321 -0.113 0 -0.301 -0.096 -0.247 0.107

20 2 -0.091 0.147 0.039 -0.036 0 0.049 0 -0.096 -0.184 0.169 0 -0.199 0.174 -0.045 -0.139 -0.272 0.172 0.016 0.321 -0.113 -0.018 -0.19 -0.002 -0.247 0.107

20 2 -0.091 0.147 0.039 -0.036 0 0.049 0 -0.096 -0.184 0 0 -0.199 0.174 -0.045 -0.139 -0.272 0.172 -0.158 0.321 -0.113 -0.068 -0.19 -0.096 -0.247 0.107

20 2 -0.091 0.147 0.039 -0.036 0 0.049 0 -0.096 -0.184 0 0 -0.199 0.174 -0.045 -0.139 -0.272 0.172 0.016 0.321 -0.113 0 -0.19 0.038 -0.247 0.107

20 2 -0.054 0.147 0.039 -0.036 0 0.049 0 -0.096 -0.184 0 0 -0.199 0.174 -0.045 -0.139 -0.272 0.205 0.016 0.321 -0.113 0 -0.19 -0.043 -0.247 0.107

20 2 -0.091 0.147 0.039 -0.036 0 0.049 0 -0.096 -0.184 0 0 -0.199 0.174 -0.045 -0.139 -0.179 0.172 0.016 0.321 -0.113 0 -0.19 -0.096 -0.247 0.107

20 2 -0.091 0.147 0.039 -0.036 0 0.049 0 -0.096 -0.184 0 0 -0.199 0.174 -0.045 -0.139 -0.272 0.172 0.016 0.321 -0.324 0 -0.19 -0.096 -0.247 0.107

20 2 -0.091 0.147 0.039 -0.036 0 0.049 0 -0.096 -0.184 0 0 -0.126 0.174 -0.045 -0.139 -0.272 0.172 0.016 0.321 -0.113 0 -0.19 -0.096 -0.247 0.107

20 2 -0.091 0.147 -0.009 -0.036 0 0.049 0 -0.096 -0.184 0 0 -0.199 0.174 -0.045 -0.139 -0.272 0.172 0.016 0.321 -0.113 0 -0.19 -0.096 -0.247 0.107

20 2 -0.054 0.147 0.039 -0.036 0 0.049 0 -0.096 -0.217 0 0 -0.199 0.174 -0.045 -0.139 -0.272 0.172 0.016 0.321 -0.113 0 -0.19 -0.043 -0.247 0.107

20 2 -0.091 0.147 0.039 -0.036 0 0.049 0 -0.304 -0.184 0 0 -0.199 0.174 -0.045 -0.139 -0.272 0.172 0.016 0.366 -0.113 0 -0.19 -0.013 -0.247 0.107

20 2 -0.091 0.069 0.039 -0.036 0 0.039 0 0.257 -0.184 0.156 0 0 0.174 -0.045 -0.139 -0.272 0.172 -0.005 0.226 -0.113 0 -0.19 -0.096 -0.283 0.135

20 2 -0.091 0.147 0.039 -0.036 0 0.049 0 -0.096 -0.184 0 0 -0.199 0.174 -0.045 -0.139 -0.272 0.172 0.016 0.321 -0.113 0 -0.19 -0.07 -0.247 0.107

20 2 -0.091 0.147 0.039 -0.036 0 0.049 0 -0.096 -0.184 0 0 -0.199 0.174 -0.045 -0.139 -0.416 0.172 0.016 0.366 -0.113 0 -0.19 -0.013 -0.247 0.107

20 2 -0.091 0.069 0.039 -0.036 0 0.039 0 0.257 -0.184 0.156 -0.128 0 0.174 -0.045 -0.139 -0.272 0.172 -0.005 0.226 -0.113 0 -0.19 -0.096 -0.283 0.107

20 2 -0.091 0.147 0.039 -0.036 0 0.049 0 -0.096 -0.184 0 0 -0.199 0.174 -0.045 -0.139 -0.272 -0.027 0.016 0.321 -0.113 0 -0.19 -0.096 -0.247 0.107

20 2 -0.091 0.147 0.039 -0.036 0 0.049 0 -0.096 -0.184 0 0 -0.199 0.174 0.004 -0.139 -0.358 0.172 0.016 0.321 -0.113 0 -0.19 -0.096 -0.256 0.107

20 2 -0.091 0.147 0.039 -0.036 0 0.049 0 -0.096 -0.184 0 0 -0.199 0.174 -0.045 -0.139 -0.272 0.172 0.016 0.223 -0.113 0 -0.19 -0.096 -0.247 0.107

20 2 -0.054 0.147 0.039 -0.036 0 0.049 0 -0.096 -0.184 0 0 -0.199 0.174 -0.045 -0.139 -0.272 0.172 0.016 0.356 -0.113 0 -0.19 -0.043 -0.247 0.107

20 2 -0.091 0.147 0.039 -0.036 0.114 0.049 0 -0.096 -0.184 0.169 0 -0.199 0.174 -0.045 -0.139 -0.272 0.172 0.016 0.321 -0.113 -0.018 -0.228 -0.096 -0.247 0.107

20 2 -0.091 0.147 0.039 -0.036 0 0.049 0 -0.17 -0.184 -0.033 0 -0.199 0.174 -0.045 -0.139 -0.272 0.172 0.016 0.321 -0.113 0 -0.19 -0.096 -0.247 0.107

20 2 -0.091 0.069 0.039 -0.036 0.246 0.039 0 0.257 -0.184 0.156 0 0 0.174 -0.045 -0.139 -0.272 0.172 -0.005 0.226 -0.113 0 -0.19 -0.096 -0.283 0.107

20 2 -0.091 0.147 0.039 -0.036 0 0.049 0 -0.096 -0.184 0 0 -0.235 0.174 -0.045 -0.139 -0.272 0.172 0.016 0.321 -0.113 0 -0.19 -0.096 -0.247 0.107

20 2 -0.091 0.147 0.039 -0.036 0 0.049 0 -0.096 -0.184 0 0 -0.284 0.174 -0.045 -0.139 -0.272 0.172 0.016 0.321 -0.113 0 -0.19 -0.096 -0.247 0.107

20 1 -0.091 0.147 0.039 -0.036 0 0.049 0.209 -0.096 -0.184 0 0 -0.199 0.174 -0.045 -0.139 -0.272 0.172 0.016 0.321 -0.113 0 -0.19 -0.096 -0.247 0.107

20 1 -0.091 0.147 0.039 0.116 0 0.049 0 -0.096 -0.184 0 0 -0.199 0.174 -0.045 -0.139 -0.272 0.172 0.016 0.321 -0.172 0 -0.19 -0.096 -0.247 0.107

20 1 -0.054 0.147 0.039 -0.036 0 0.049 0 -0.096 -0.184 0 0 -0.199 0.174 -0.045 -0.139 -0.272 0.172 0.016 0.321 -0.113 0 -0.19 -0.043 -0.247 -0.132

20 1 -0.091 0.147 0.039 -0.036 0 0.049 0 -0.096 -0.184 0 0 -0.199 0.292 -0.045 -0.139 -0.272 0.172 0.016 0.321 -0.113 0 -0.19 -0.096 -0.247 0.107

20 1 -0.091 0.147 0.039 -0.036 0 0.049 0 -0.096 -0.184 0 0 -0.246 0.174 -0.051 -0.139 -0.272 0.172 0.016 0.321 -0.113 0 -0.19 -0.096 -0.247 0.107

20 1 -0.091 0.147 0.039 -0.036 0 0.049 0 -0.096 -0.184 0 0 -0.199 0.174 -0.045 -0.139 -0.272 0.172 0.016 0.321 -0.113 0 -0.19 -0.19 -0.247 0.107

20 1 -0.104 0.147 0.039 -0.036 0 0.049 0 -0.096 -0.184 0 0.023 -0.199 0.174 -0.009 -0.139 -0.272 0.172 0.061 0.321 -0.113 0 -0.19 -0.096 -0.247 0.107

20 1 -0.091 0.147 0.039 -0.036 0 0.049 0 -0.096 -0.184 0.169 0 -0.199 0.174 -0.045 -0.137 -0.272 0.172 0.016 0.321 -0.113 -0.018 -0.228 -0.096 -0.247 0.107

20 1 -0.091 0.147 0.039 -0.036 0 0.049 0 -0.096 -0.184 0 0 -0.199 0.174 -0.045 -0.139 -0.272 0.097 0.016 0.366 -0.113 0 -0.19 -0.096 -0.247 0.107

20 1 -0.091 0.147 0.039 -0.036 0 0.049 0 -0.096 -0.184 0 -0.091 -0.199 0.174 -0.158 -0.139 -0.272 0.172 0.016 0.321 -0.113 -0.068 -0.19 -0.096 -0.247 0.107

20 1 0.012 0.147 0.039 -0.036 0 0.049 0 -0.096 -0.184 0 0 -0.199 0.174 -0.045 -0.139 -0.272 0.172 0.016 0.321 -0.113 0 -0.19 -0.096 -0.247 0.107

20 1 -0.091 0.147 0.039 -0.036 0 0.049 0.085 -0.096 -0.184 0 0 -0.199 0.174 -0.045 -0.139 -0.272 0.172 0.016 0.321 -0.113 0 -0.19 -0.096 -0.247 0.107

20 1 -0.006 0.147 0.039 -0.036 0 0.049 0 -0.096 -0.184 0 0 -0.199 0.174 -0.045 -0.139 -0.272 0.172 0.016 0.321 -0.113 0 -0.19 -0.096 -0.247 0.107

20 1 -0.091 0.147 0.039 -0.036 0 0.049 0 -0.096 -0.184 0 0 -0.199 0.174 -0.045 -0.139 -0.272 0.172 0.061 0.321 -0.113 0 -0.135 -0.096 -0.247 0.107

20 1 -0.091 0.147 0.039 -0.036 0 0.049 0 -0.096 -0.184 0 0 -0.199 0.174 -0.045 -0.139 -0.272 0.172 0.061 0.321 -0.113 0 -0.19 -0.106 -0.247 0.107

20 1 -0.031 0.147 0.039 -0.036 0 0.049 0 -0.096 -0.184 0 0 -0.199 0.174 -0.045 -0.139 -0.272 0.172 0.016 0.321 -0.113 0 -0.19 -0.096 -0.247 0.107

20 1 -0.091 0.147 0.039 -0.036 0 0.049 -0.065 -0.096 -0.184 0 0 -0.199 0.174 -0.045 -0.139 -0.272 0.172 0.016 0.321 -0.113 0 -0.19 -0.096 -0.247 0.107

20 1 -0.091 0.147 0.039 -0.054 0 0.049 0 -0.096 -0.184 0 0 -0.246 0.174 -0.045 -0.139 -0.272 0.172 0.016 0.321 -0.113 0 -0.19 -0.096 -0.247 0.107

20 1 -0.091 0.147 0.039 -0.036 0 0.049 0 -0.096 -0.184 0 0 -0.199 0.174 -0.045 -0.139 -0.272 0.172 -0.056 0.321 -0.113 0 -0.19 -0.096 -0.247 0.107

20 1 -0.054 0.147 0.039 -0.036 0 0.049 0 -0.096 -0.184 0.014 0 -0.199 0.174 -0.045 -0.139 -0.272 0.172 0.016 0.321 -0.113 0 -0.19 -0.043 -0.247 0.107

20 1 0.103 0.147 0.039 -0.036 0 0.049 0 -0.096 -0.184 0 0 -0.199 0.174 -0.045 -0.139 -0.272 0.172 0.016 0.321 -0.113 0 -0.19 -0.096 -0.247 0.107

20 1 -0.091 0.147 0.039 -0.036 0 0.049 0 -0.096 -0.184 0 0 -0.199 0.174 -0.045 -0.139 -0.272 0.076 0.016 0.321 -0.113 0 -0.19 -0.096 -0.247 0.107

20 1 -0.054 0.147 0.039 -0.036 0 0.049 0 -0.096 -0.184 0 0 -0.199 0.238 -0.045 -0.139 -0.272 0.172 0.016 0.321 -0.113 0 -0.19 -0.043 -0.247 0.107

20 1 -0.091 0.147 0.039 -0.036 0 0.049 0 -0.096 -0.184 0 0 -0.199 0.174 -0.045 -0.139 -0.272 0.335 0.016 0.321 -0.113 0 -0.19 -0.096 -0.247 0.107

20 1 -0.091 0.069 0.039 -0.036 0 0.039 0 0.257 -0.184 0.156 0 0 0.174 -0.045 -0.139 -0.272 0.366 -0.005 0.226 -0.113 0 -0.19 -0.096 -0.283 0.107

20 1 -0.054 0.147 0.039 -0.036 0 0.049 0 -0.063 -0.184 0 0 -0.199 0.174 -0.045 -0.139 -0.272 0.172 0.016 0.321 -0.113 0 -0.19 -0.043 -0.247 0.107

20 1 -0.091 0.147 0.039 -0.036 0 0.049 0 -0.096 -0.184 0 0 -0.199 0.174 -0.045 -0.195 -0.272 0.172 0.016 0.321 -0.113 0 -0.19 -0.096 -0.247 0.107

20 1 -0.091 0.147 0.039 -0.036 0 0.049 -0.004 -0.096 -0.184 0 0 -0.199 0.174 -0.045 -0.139 -0.358 0.172 0.016 0.321 -0.113 0 -0.19 -0.096 -0.256 0.107

20 1 -0.091 0.147 0.039 -0.036 0 0.049 0 -0.096 -0.184 0 0 -0.246 0.174 -0.045 -0.139 -0.272 0.152 0.016 0.321 -0.113 0 -0.19 -0.096 -0.247 0.107

20 1 -0.091 0.147 0.039 -0.036 0 0.224 0 -0.096 -0.184 0 0 -0.199 0.174 -0.045 -0.139 -0.272 0.172 0.016 0.321 -0.113 0 -0.19 -0.096 -0.247 0.107

20 1 0.056 0.147 0.039 -0.036 0 0.049 0 -0.096 -0.184 0 0 -0.199 0.174 -0.045 -0.139 -0.272 0.172 0.016 0.321 -0.113 0 -0.19 -0.096 -0.247 0.107

20 1 -0.091 0.147 0.039 -0.036 0 0.049 0.06 -0.096 -0.184 0 0 -0.199 0.174 -0.045 -0.139 -0.272 0.172 0.016 0.321 -0.113 -0.068 -0.19 -0.096 -0.247 0.107

20 1 -0.091 0.147 0.039 -0.036 0 0.049 -0.037 -0.096 -0.184 0 0 -0.199 0.174 -0.045 -0.139 -0.272 0.172 0.016 0.321 -0.113 0 -0.19 -0.096 -0.247 0.107

20 1 -0.091 0.147 0.039 -0.036 0 0.049 0 -0.096 -0.184 0 0.068 -0.246 0.174 -0.045 -0.139 -0.272 0.172 0.016 0.321 -0.113 0 -0.19 -0.096 -0.247 0.107

20 1 -0.091 0.147 0.039 -0.036 0 0.049 0 -0.096 -0.184 0 0 -0.246 0.174 -0.279 -0.139 -0.272 0.172 -0.096 0.321 -0.113 0 -0.19 -0.096 -0.247 0.107

20 1 -0.091 0.147 0.039 -0.036 0 0.049 0 -0.096 -0.184 0 0 -0.199 0.174 -0.045 -0.139 -0.272 0.172 0.016 0.321 -0.113 0 -0.19 -0.096 -0.247 0.094

20 1 -0.091 0.147 0.039 -0.036 0 0.138 0 -0.096 -0.184 0 0 -0.246 0.174 -0.045 -0.139 -0.272 0.172 0.016 0.321 -0.113 0 -0.19 -0.096 -0.247 0.107

20 1 -0.091 0.069 0.039 0.071 0 0.039 0 0.257 -0.184 0.156 0 0 0.174 -0.045 -0.139 -0.272 0.172 -0.005 0.226 -0.113 0 -0.19 -0.096 -0.283 0.107

20 1 -0.133 0.147 0.039 -0.036 0 0.049 0 -0.096 -0.184 0 0 -0.199 0.174 -0.045 -0.139 -0.272 0.172 0.016 0.321 -0.113 0 -0.19 -0.096 -0.247 0.107

20 1 -0.091 0.147 0.039 -0.036 0 0.049 0 -0.096 -0.184 -0.077 0 -0.199 0.174 -0.045 -0.139 -0.272 0.172 0.016 0.321 -0.113 -0.068 -0.19 -0.096 -0.247 0.107

20 1 -0.091 0.147 0.039 -0.036 0 0.049 0 -0.096 -0.184 0 0 -0.124 0.174 -0.045 -0.139 -0.272 0.172 0.081 0.321 -0.113 0 -0.19 -0.096 -0.247 0.107

20 1 -0.091 0.147 0.039 -0.036 0 0.049 -0.08 -0.096 -0.184 0 0 -0.199 0.174 -0.045 -0.139 -0.272 0.172 0.016 0.321 -0.113 0 -0.19 -0.096 -0.247 0.107

20 1 -0.091 0.147 0.039 -0.036 0 0.049 0 -0.096 -0.184 0 0 -0.193 0.174 -0.045 -0.139 -0.272 0.172 0.016 0.321 -0.113 -0.068 -0.19 -0.096 -0.247 0.107

20 1 -0.054 0.147 0.039 -0.036 0 0.049 0 -0.096 -0.184 0 0 -0.199 0.174 -0.045 -0.139 -0.272 0.172 0.016 0.321 -0.113 0 -0.19 0.054 -0.247 0.107

20 1 -0.091 0.147 0.039 -0.036 0 0.049 0 -0.096 -0.184 0 0 -0.199 0.174 -0.045 -0.139 -0.358 0.124 0.016 0.321 -0.113 0 -0.19 -0.096 -0.256 0.107

20 1 -0.091 0.147 0.039 -0.036 0 0.049 0 -0.096 -0.184 0 0 -0.199 0.174 -0.045 -0.139 -0.272 0.172 -0.061 0.321 -0.113 0 -0.19 -0.096 -0.247 0.107

20 1 -0.054 0.147 0.039 -0.036 0 -0.001 0 -0.096 -0.184 0 0 -0.199 0.174 -0.045 -0.139 -0.272 0.172 0.016 0.321 -0.113 0 -0.19 -0.043 -0.247 0.107

20 1 -0.091 0.139 0.039 -0.036 0 0.049 0 -0.096 -0.184 0 0 -0.199 0.174 -0.045 -0.139 -0.272 0.172 0.016 0.321 -0.113 0 -0.19 -0.096 -0.247 0.107

20 1 -0.091 0.147 0.039 -0.036 0 0.049 0 -0.096 -0.184 0.169 0 -0.199 0.174 -0.045 -0.139 -0.272 0.172 0.016 0.321 -0.113 -0.018 -0.399 -0.096 -0.247 0.107

20 1 -0.091 0.147 0.039 -0.036 0 0.049 0 -0.096 -0.184 0 0 -0.199 0.174 -0.045 0.023 -0.272 0.172 0.016 0.321 -0.113 0 -0.19 -0.096 -0.247 0.107

20 1 -0.091 0.147 0.039 -0.036 0 0.049 0 -0.096 -0.184 0 0 -0.199 0.076 -0.045 -0.139 -0.272 0.172 0.016 0.321 -0.113 0 -0.19 -0.096 -0.247 0.107

20 2 -0.091 0.147 0.039 -0.036 0 0.049 0 -0.096 -0.184 0 0 -0.246 0.174 -0.045 -0.139 -0.272 0.172 0.016 0.237 -0.113 0 -0.19 -0.096 -0.247 0.107

20 1 -0.091 0.147 0.039 -0.036 0 0.049 0 -0.096 -0.184 0 0 -0.199 0.174 -0.045 -0.139 -0.433 0.172 0.016 0.321 -0.113 0 -0.19 -0.096 -0.256 0.107

20 1 -0.091 0.147 0.028 -0.036 0 0.049 0 -0.096 -0.184 0 0 -0.199 0.174 -0.045 -0.139 -0.272 0.172 0.016 0.321 -0.113 0 -0.19 -0.096 -0.247 0.107

20 1 -0.091 0.147 0.039 -0.036 0 0.049 0 -0.096 -0.184 0 0 -0.246 0.174 -0.045 -0.139 -0.272 0.052 0.016 0.321 -0.113 0 -0.19 -0.096 -0.247 0.107

20 1 -0.091 0.147 0.039 -0.036 0 0.049 0 -0.096 -0.184 0 0 -0.199 0.174 -0.045 -0.139 -0.272 0.172 0.016 0.321 -0.113 0 -0.19 -0.096 -0.247 0.195

20 1 -0.054 0.147 0.039 -0.036 0 0.049 0 -0.096 -0.184 0 0 -0.154 0.174 -0.045 -0.139 -0.272 0.172 0.016 0.321 -0.113 0 -0.19 -0.043 -0.247 0.107

20 1 -0.054 0.147 0.049 -0.036 0 0.049 0 -0.096 -0.184 0 0 -0.199 0.174 -0.045 -0.139 -0.335 0.172 0.016 0.321 -0.113 0 -0.19 -0.043 -0.247 0.107

20 1 -0.091 0.147 0.039 -0.036 0 0.049 0 -0.117 -0.184 0 0 -0.199 0.174 -0.045 -0.139 -0.272 0.172 0.016 0.321 -0.113 0 -0.19 -0.096 -0.247 0.107

20 1 -0.091 0.147 0.039 -0.061 0 0.049 0 -0.096 -0.184 0 0 -0.199 0.174 -0.045 -0.139 -0.272 0.172 0.016 0.321 -0.113 0 -0.19 -0.096 -0.247 0.107

20 1 -0.091 0.147 0.039 -0.036 0 0.049 0 -0.096 -0.184 0 0.068 -0.246 0.174 -0.045 -0.139 -0.338 0.172 0.016 0.321 -0.113 0 -0.19 -0.096 -0.247 0.107

20 1 -0.054 0.147 0.039 -0.036 0 0.049 0 -0.096 -0.184 0 0 -0.199 0.174 -0.045 -0.139 -0.332 0.172 0.016 0.321 -0.113 0 -0.19 -0.043 -0.247 0.107

20 1 -0.091 0.147 0.039 -0.036 0 0.049 0 -0.096 -0.184 0 0 -0.199 0.06 -0.045 -0.139 -0.272 0.172 0.016 0.321 -0.113 0 -0.19 -0.096 -0.247 0.107

20 1 -0.054 0.147 0.039 -0.283 0 0.049 0 -0.096 -0.184 0 0 -0.199 0.174 -0.045 -0.139 -0.272 0.172 0.016 0.321 -0.113 0 -0.19 -0.043 -0.247 0.107

20 1 -0.091 0.069 0.039 -0.036 -0.013 0.039 0 0.257 -0.184 0.156 0 0 0.174 -0.045 -0.139 -0.272 0.172 -0.005 0.226 -0.113 0 -0.19 -0.096 -0.283 0.107

20 1 -0.091 0.147 0.039 -0.036 0 0.049 0 -0.096 -0.184 0 0 -0.199 0.174 -0.045 -0.139 -0.272 0.172 0.016 0.321 -0.113 -0.058 -0.19 -0.096 -0.247 0.107

20 1 -0.091 0.147 0.039 -0.036 0 0.049 0 -0.096 -0.184 0 0 -0.199 0.174 -0.045 -0.139 -0.358 0.172 0.016 0.207 -0.113 0 -0.19 -0.096 -0.256 0.107

20 1 -0.091 0.147 0.039 -0.036 0 0.049 0 -0.096 -0.184 0 0 -0.199 0.174 -0.045 -0.139 -0.272 0.172 0.016 0.321 -0.113 -0.146 -0.219 -0.096 -0.247 0.107

20 1 -0.091 0.147 0.039 -0.067 0 0.049 0 -0.096 -0.184 0 0 -0.246 0.174 -0.045 -0.139 -0.272 0.172 0.016 0.321 -0.113 0 -0.19 -0.096 -0.247 0.107

20 1 -0.091 0.147 0.088 -0.036 0 0.049 0 -0.096 -0.184 0 0 -0.199 0.174 -0.045 -0.139 -0.272 0.172 0.016 0.321 -0.113 0 -0.19 -0.096 -0.247 0.107

20 1 -0.091 0.147 0.039 -0.036 0 0.057 0 -0.096 -0.184 0 0 -0.199 0.174 -0.045 -0.139 -0.272 0.172 0.016 0.321 -0.113 0 -0.19 -0.096 -0.247 0.107

20 1 -0.091 0.147 0.039 -0.036 0 0.049 0 -0.096 -0.184 0.169 0 -0.199 0.174 -0.045 -0.139 -0.272 0.238 0.016 0.321 -0.113 -0.018 -0.19 -0.096 -0.247 0.107

20 1 -0.091 0.147 0.039 -0.036 0 0.049 0 -0.096 -0.201 0 0 -0.199 0.174 -0.045 -0.139 -0.272 0.172 0.016 0.321 -0.113 0 -0.19 -0.096 -0.247 0.107

20 1 -0.091 0.147 0.039 -0.036 0 0.049 0 -0.096 -0.184 0 0 -0.246 0.174 -0.045 -0.139 -0.272 0.172 0.016 0.321 -0.113 0 -0.19 -0.106 -0.247 0.107

20 1 -0.054 0.147 0.039 -0.036 0 0.049 0.008 -0.096 -0.184 0 0 -0.199 0.174 -0.045 -0.139 -0.272 0.172 0.016 0.321 -0.113 0 -0.19 -0.043 -0.247 0.107

20 1 -0.091 0.069 0.039 -0.036 0 0.039 0 0.095 -0.184 0.156 0 0 0.174 -0.045 -0.139 -0.272 0.172 -0.005 0.226 -0.113 0 -0.19 -0.096 -0.283 0.107

20 1 -0.091 0.147 0.039 -0.036 0 0.049 0 -0.096 -0.184 0 0 -0.199 0.174 -0.045 -0.139 -0.272 0.172 0.016 0.409 -0.113 0 -0.19 0.125 -0.247 0.107

20 1 -0.091 0.147 0.039 -0.036 0 0.049 0 -0.096 -0.184 0.169 0 -0.199 0.174 -0.045 -0.139 -0.272 0.172 -0.037 0.321 -0.113 -0.018 -0.228 -0.096 -0.247 0.107

20 1 -0.091 0.147 0.039 -0.036 0 0.049 0 -0.066 -0.184 0 0 -0.199 0.174 -0.045 -0.139 -0.272 0.172 0.016 0.321 -0.113 0 -0.19 -0.096 -0.247 0.107

20 1 -0.054 0.147 0.039 -0.036 0 0.049 0 -0.096 -0.184 0 -0.092 -0.199 0.174 -0.045 -0.139 -0.272 0.172 0.016 0.321 -0.113 0 -0.19 -0.043 -0.247 0.107

20 1 -0.091 0.147 0.039 -0.036 0 0.049 0 -0.096 -0.184 0 0 -0.199 0.174 -0.147 -0.139 -0.272 0.172 0.016 0.321 -0.113 0 -0.19 -0.096 -0.247 0.107

20 1 -0.091 0 0.039 -0.036 0 0.049 0 -0.096 -0.184 0 0 -0.199 0.174 -0.045 -0.195 -0.272 0.172 0.016 0.321 -0.113 0 -0.19 -0.096 -0.247 0.107

20 1 -0.091 0.147 0.039 -0.036 0 0.049 0 -0.096 -0.014 -0.033 0 -0.199 0.174 -0.045 -0.139 -0.272 0.172 0.016 0.321 -0.113 0 -0.19 -0.096 -0.247 0.107

20 1 -0.091 0.147 0.039 -0.036 0 0.049 0 -0.096 -0.184 0 0 -0.199 0.174 -0.045 0.008 -0.358 0.172 0.016 0.321 -0.113 0 -0.19 -0.096 -0.256 0.107

20 1 -0.091 0.147 0.019 -0.036 0 0.049 0 -0.096 -0.184 0.169 0 -0.199 0.174 -0.045 -0.139 -0.272 0.172 0.016 0.321 -0.113 -0.018 -0.399 -0.096 -0.247 0.107

20 1 -0.091 0.147 0.039 -0.036 0 0.049 0 -0.096 -0.184 0 0 -0.199 0.174 -0.045 0 -0.272 0.172 0.016 0.321 -0.113 0 -0.19 -0.096 -0.247 0.107

20 1 -0.091 0.147 0.039 -0.036 0 0.049 0 -0.096 -0.184 0 0 -0.199 0.174 -0.045 -0.062 -0.272 0.172 0.016 0.321 -0.113 0 -0.19 -0.096 -0.247 0.107
